# Identification of metabolites reproducibly associated with Parkinson’s Disease via meta-analysis and computational modelling

**DOI:** 10.1038/s41531-024-00732-z

**Published:** 2024-06-29

**Authors:** Xi Luo, Yanjun Liu, Alexander Balck, Christine Klein, Ronan M. T. Fleming

**Affiliations:** 1 School of Medicine, University of Galway, University Rd, Galway, Ireland; 2 Institute of Neurogenetics and Department of Neurology, University of Luebeck and University Hospital Schleswig-Holstein, Luebeck, Germany; 3 Division of Systems Biomedicine and Pharmacology, Leiden Academic Centre for Drug Research, Leiden University, Leiden, Netherlands

**Keywords:** Parkinson's disease, Diagnostic markers

## Abstract

Many studies have reported metabolomic analysis of different bio-specimens from Parkinson’s disease (PD) patients. However, inconsistencies in reported metabolite concentration changes make it difficult to draw conclusions as to the role of metabolism in the occurrence or development of Parkinson’s disease. We reviewed the literature on metabolomic analysis of PD patients. From 74 studies that passed quality control metrics, 928 metabolites were identified with significant changes in PD patients, but only 190 were replicated with the same changes in more than one study. Of these metabolites, 60 exclusively increased, such as 3-methoxytyrosine and glycine, 54 exclusively decreased, such as pantothenic acid and caffeine, and 76 inconsistently changed in concentration in PD versus control subjects, such as ornithine and tyrosine. A genome-scale metabolic model of PD and corresponding metabolic map linking most of the replicated metabolites enabled a better understanding of the dysfunctional pathways of PD and the prediction of additional potential metabolic markers from pathways with consistent metabolite changes to target in future studies.

## Introduction

Parkinson’s disease (PD) is the second most common neurodegenerative disorder. Primarily as a result of an increasing elderly population, from 1990 to 2019, the number of patients with PD increased to almost 4 million globally^1,2^. As PD is a multifactorial disease with various molecular mechanisms, a series of biomarkers derived from clinical factors, neuroimaging, genomics, and biomolecules have been proposed for PD diagnosis and progression^3,4^.

Metabolites are small endogenous molecules generated by various enzymes in metabolic reactions; they join in essential cellular functions, such as energy metabolism, signal transduction and apoptosis^5^. Evidence has indicated that metabolites may provide insight into the mechanisms of various pathophysiological processes through dysfunctional metabolic pathways^6^. Therefore, an increasing focus of research identifies metabolites as significant biomarkers to distinguish PD patients from asymptomatic controls, and to predict the disease progression or prognosis of PD patients^7^.

Several studies have reviewed metabolic biomarkers of PD. These studies briefly summarized significant metabolites of PD from clinical and experimental studies^8^, reported advancements of analytical platforms used in metabolomic studies^9^, and concisely discussed current PD metabolic biomarker discovery and validation methods^10^. However, currently identified PD metabolites are highly heterogeneous and segregated, these studies failed to identify the reproducibility of these metabolite changes in PD, and are not enough to comprehensively understand the significant metabolites and dysfunctional pathways of PD.

Human genome-scale metabolic modeling is increasingly used to understand normal cellular functions and disease states^11–14^. It provides molecular mechanistic framework for integrative analysis of segregated molecular data, and quantitative prediction of phenotypic states^12^. Such models can be used to simulate whole-body metabolism and perform functional predictions based on cell-specific metabolic models combined with omics data^14–16^. To comprehensively understand the dysfunctional metabolic pathways of PD, it is necessary to effectively integrate reliable PD metabolites with genome-scale computational modeling.

In this study, we systematically reviewed the literature on the metabolomic studies of Parkinson’s Disease patients. Then, we summarized the reported diagnosis-associated metabolites to reveal consistent and inconsistent metabolomic changes and analyzed possible reasons for inconsistency. Next, replicated metabolites were identified to explore the dysfunctional pathways of PD and generate a genome-scale metabolic model that is enriched with metabolic pathways containing PD-associated metabolites in order to put the metabolic perturbations associated with PD into a systems biochemical context. Consistently changed pathways and potential metabolic markers of PD were explored through the core map of the metabolic model.

## Results

### Study characteristics

**Fig. 1:**
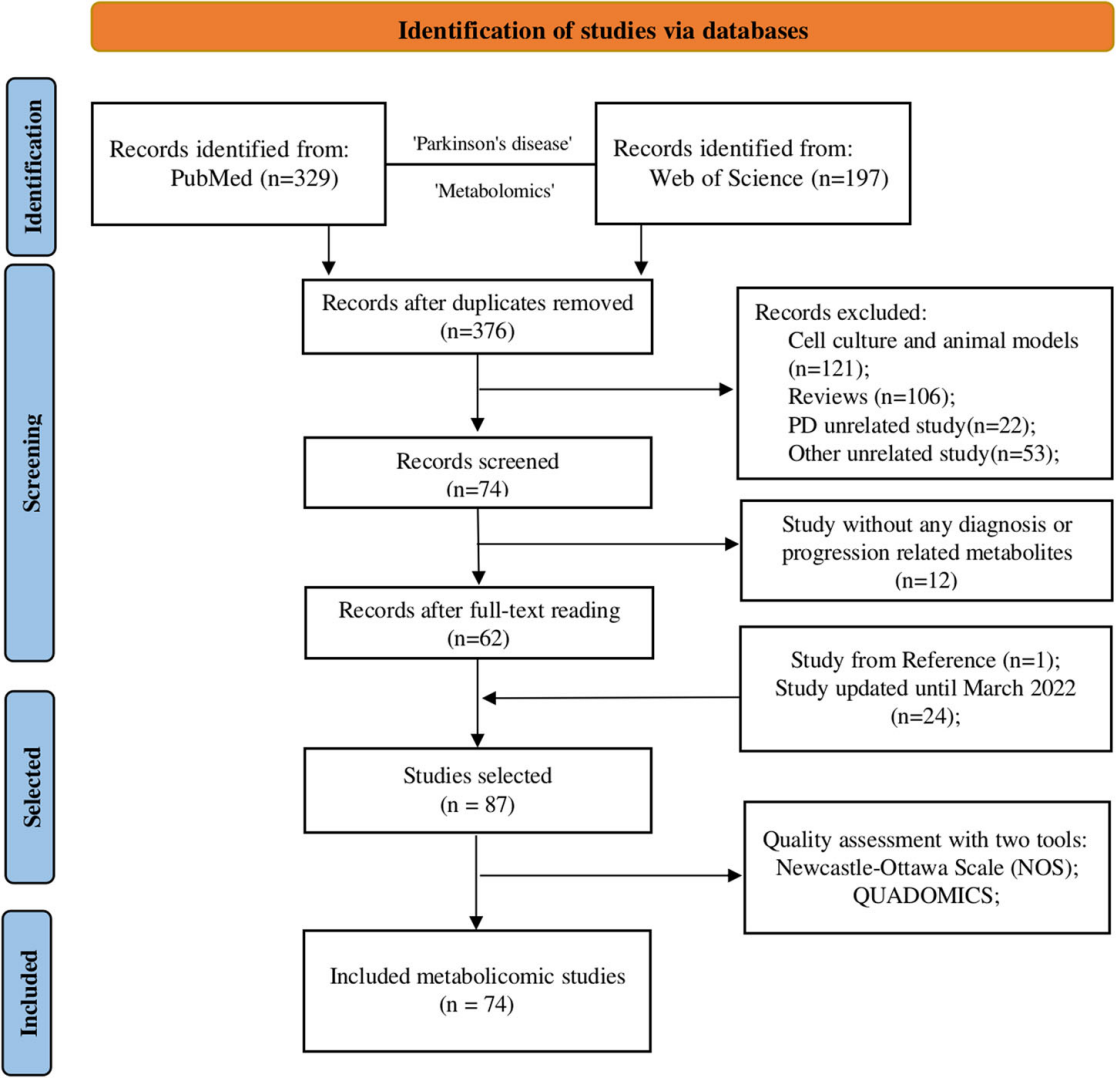
Study selection diagram. The target metabolomic studies of PD were searched from databases until March 2021 and updated until March 2022. In this study, the diagnosis-related metabolites were considered as the metabolites with different abundances between PD patients and asymptomatic controls. Due to the limited number of longitudinal metabolomic studies, our definition of progression-related metabolites has been expanded to include metabolites that are associated with PD severity, motor score, and disease duration, or metabolites with changed abundances in follow-up cohorts.

As shown in Fig. 1, a total of 87 metabolomic studies of PD patients were selected, 81 of which were case-control studies and the remaining six reported longitudinal metabolomic analysis. Out of these six, only three longitudinal metabolomic studies identified potential progression-related metabolites. Due to the limited longitudinal metabolomic studies, we extended the definition of progression-related metabolites to include metabolites associated with PD severity, motor score, and disease duration, or metabolites with changed abundances in follow-up cohorts. A comparison of progression-related metabolites between longitudinal and case-control studies was shown in Supplementary Figure 1. Details on each selected study is shown in Supplementary Table 1.

**Fig. 2:**
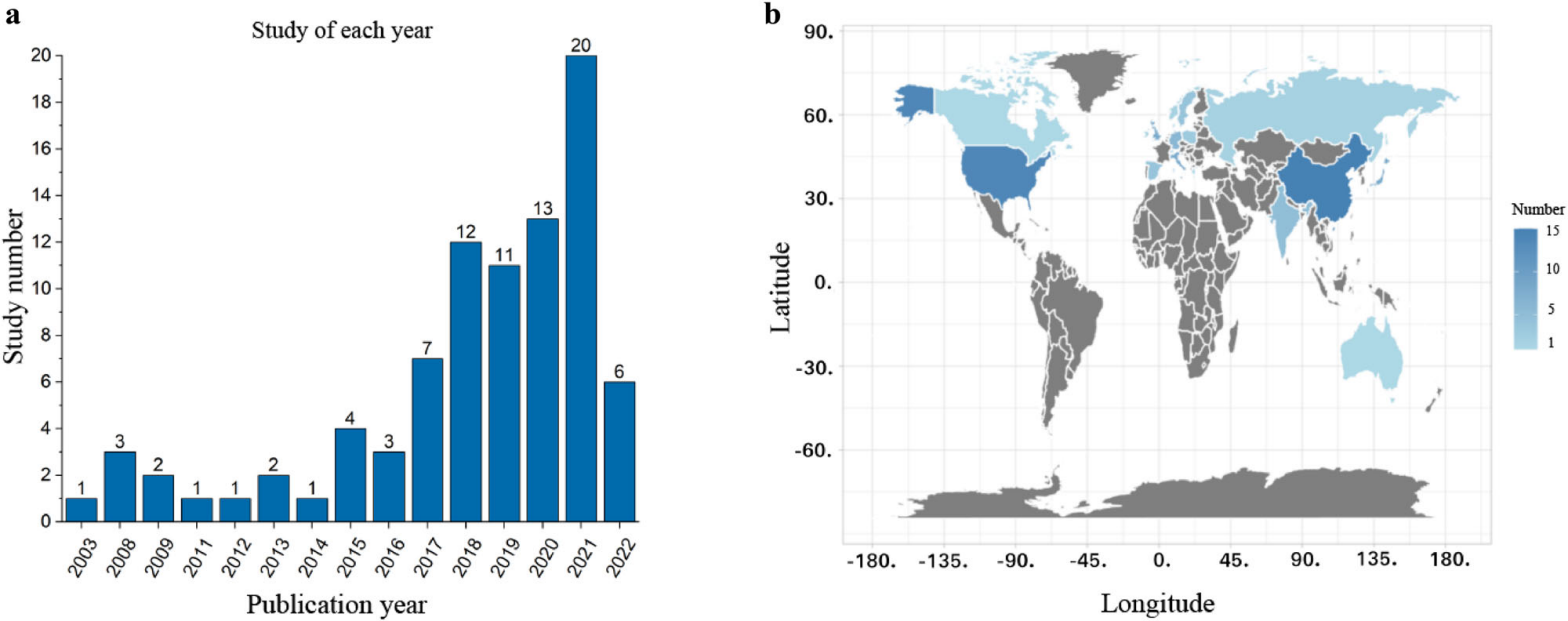
Study characteristics. **a** The number of studies of each year. **b** The involved countries of PD clinical studies.

All the selected studies were published from 2003 to March 2022, involving patients from 17 countries across the world (Fig. 2). These metabolomic studies utilized various bio-specimens, with plasma used in 39 studies, serum in 22 studies, cerebrospinal fluid (CSF) in 17 studies, brain tissue in six studies, urine in six studies, fecal in six studies, and other samples including sebum, saliva and human appendix in four studies.

**Fig. 3:**
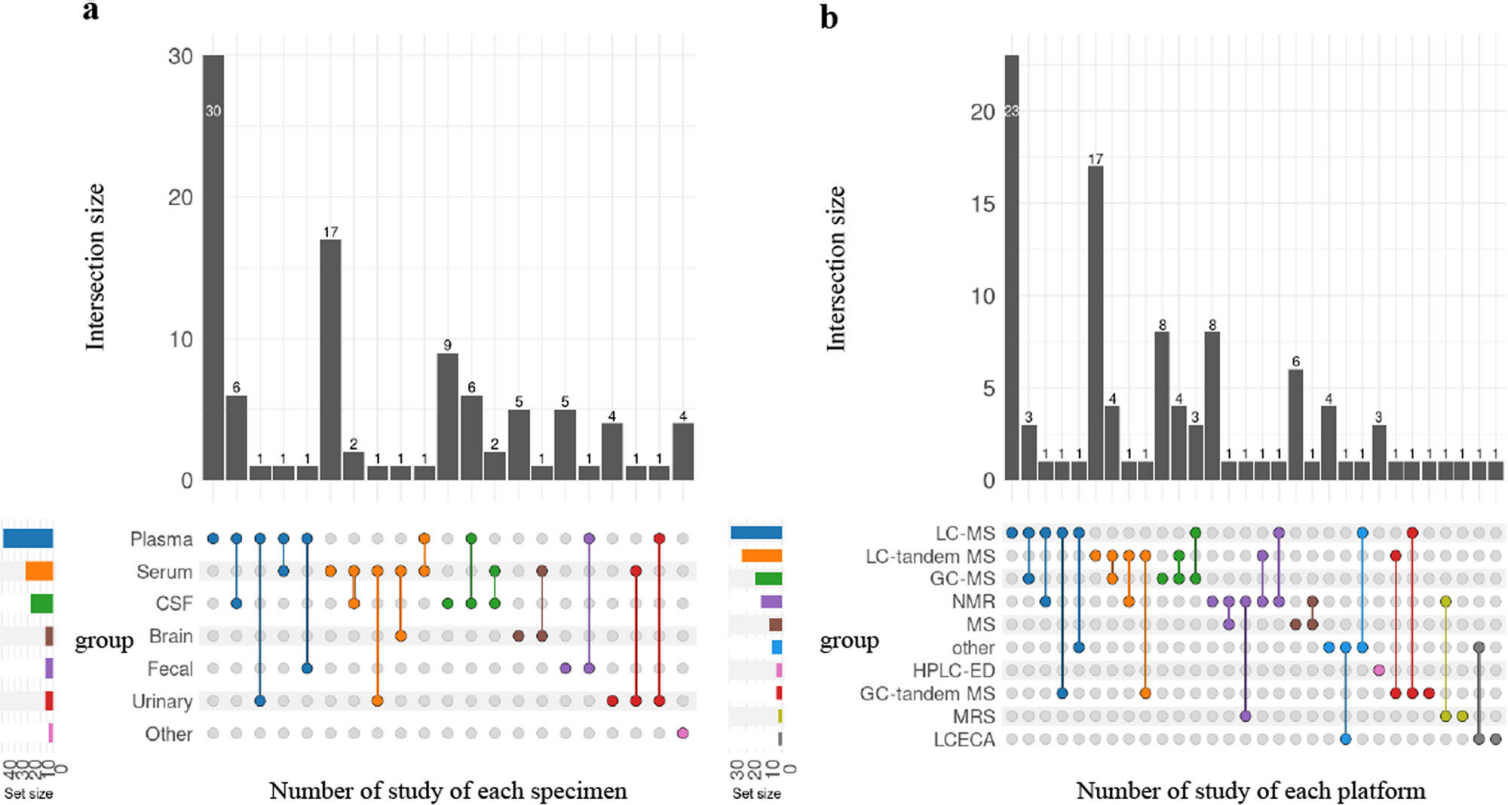
Detailed information on specimens and platforms. **a** Number of studies of each specimen. **b** Number of studies of each platform. The upset plot can visualize intersections between sets in the data matrix. Each specimen or platform corresponds to one set, and the study involved corresponds to the intersections. The size of the intersections is represented by bar charts aligned with the rows. The colored upset plot displays all the intersections involved in one set (specimen or platform) together, which facilitates the comparison between the sizes of individual sets, as the total study number of each set is easy to count, despite duplicating the columns. For example, the green color in the plots can easily help us to count the total study number involved in CSF or GC-MS. Abbreviations are as follows LC-MS liquid chromatography combined with mass spectrometry, LC-tandem MS liquid chromatography combined with tandem mass spectrometry, GS-MS gas chromatography combined with mass spectrometry, GC-tandem MS gas chromatography combined with tandem mass spectrometry, NMR nuclear magnetic resonance, HPLC-ED electrochemical detection coupled with high-performance liquid chromatography, LCECA liquid chromatography electrochemical array, MRS magnetic resonance spectroscopy, other immunoassay, electrode array, fluorescence detection and enzyme test.

The analytical platforms employed for metabolite measurement comprised liquid chromatography combined with mass spectrometry (LC-MS) in 29 studies, LC-tandem MS in 23 studies, gas chromatography combined with mass spectrometry (GC-MS) in 15 studies, GC-tandem MS in three studies, nuclear magnetic resonance (NMR) in 12 studies, only MS in seven studies, electrochemical detection coupled with high-performance liquid chromatography (HPLC-ED) in three studies, liquid chromatography electrochemical array (LCECA) in two studies, Magnetic Resonance Spectroscopy (MRS) in two studies, and other platforms including immunoassay, electrode array, fluorescence detection and enzyme test in six studies. The number of studies on each specimen and platform is shown in Fig. 3, most of these metabolomic studies chose to detect metabolomic profiles on blood samples, especially plasma, using liquid chromatography combined with mass spectrometry or tandem mass spectrometry platforms.

### Quality assessment

**Fig. 4:**
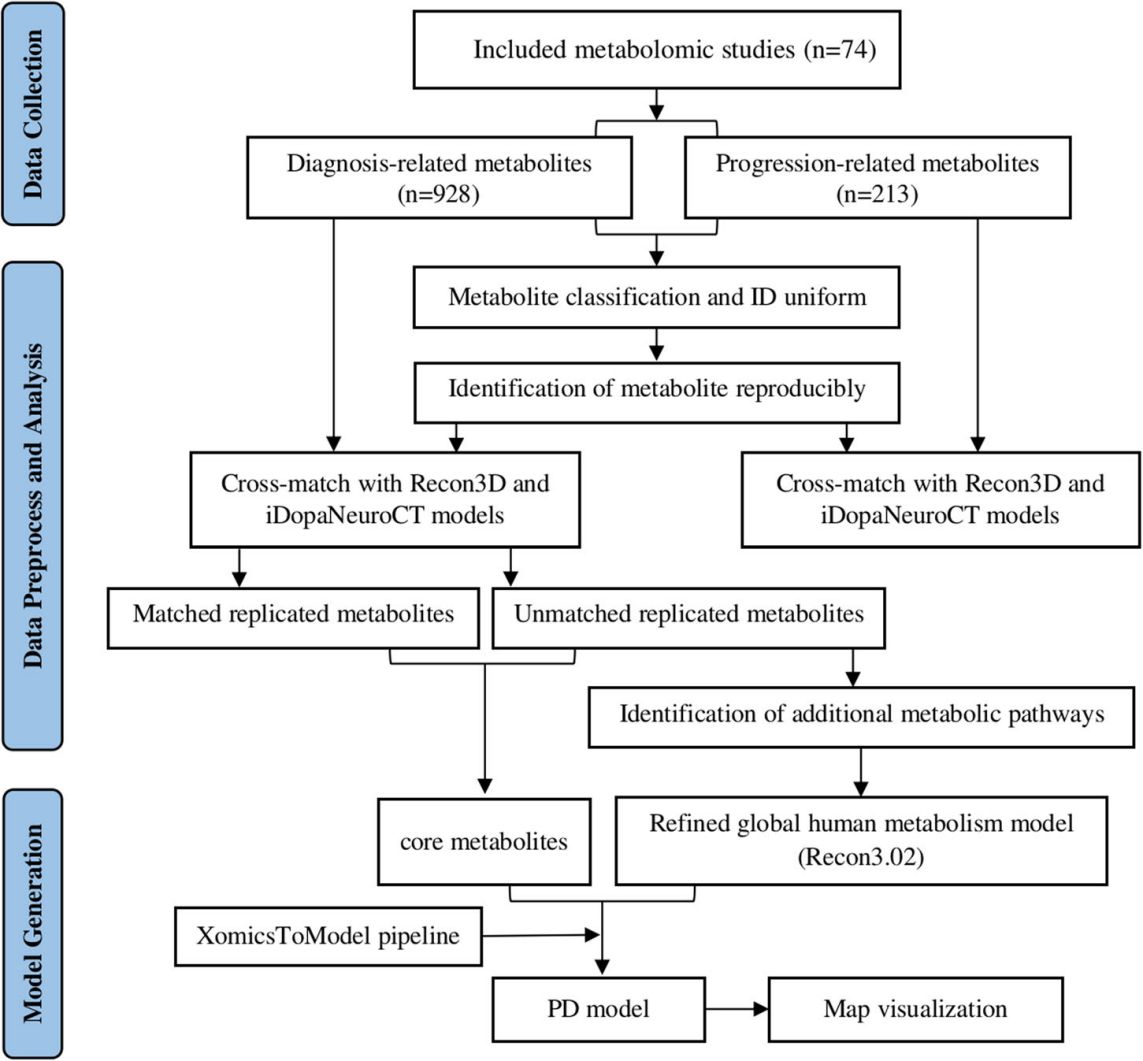
The schematic diagram of metabolite analysis in this study.

The quality assessment results of QUADOMICS and Newcastle-Ottawa Scale (NOS) performed on 84 diagnosis-related studies are shown in Supplementary Table 2. Three independent progression-related studies did not perform quality assessment because they were not qualified for the assessment of QUADOMICS. According to the QUADOMICS results, the scores of 76 studies were higher than 7, and eight studies had scores below 8. According to the NOS results, the scores of 79 studies were higher than 6. Five studies had scores below 7, three of which belonged to low quality studies both in the QUADOMICS and NOS assessments. Combining the results of two tools, a subset of 74 diagnosis-related studies were deemed high-quality studies. Therefore, the diagnosis-related metabolites from 74 studies and progression-related metabolites from 19/74 studies were used for further analysis. The schematic diagram of metabolites analysis in this study is shown in Fig. 4.

### Consistency of metabolites

**Fig. 5:**
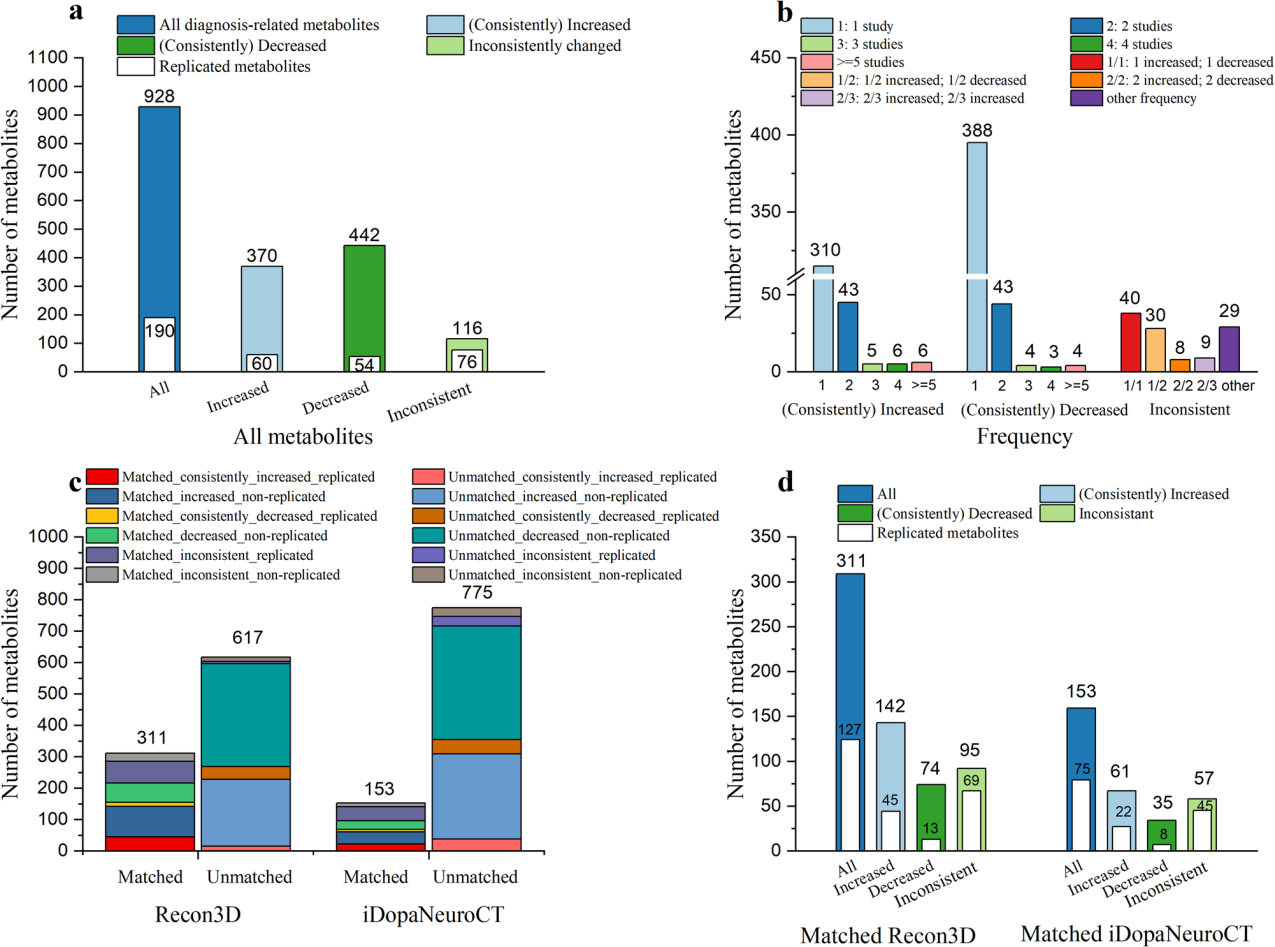
Diagnosis-related metabolites of PD. **a** All diagnosis-related metabolites with different changes. **b** The number of studies of each frequency in different changes. **c** Cross-matched results with Recon3D and the dopaminergic neuronal cell-type metabolism model (iDopaNeuroCT). **d** The number of studies of matched results in different changes.

As shown in Fig. 5a, a total of 928 diagnosis-related metabolites were identified, with 370 metabolites that were exclusively increased, 442 metabolites that were exclusively decreased, and 116 metabolites that were identified with inconsistent changes in different studies. A total of 190 diagnosis-related metabolites were identified as replicated metabolites, with 60 consistently increased, 54 consistently decreased, and 76 inconsistently changed metabolites. We considered a metabolite to be replicated if it was found to be increased or decreased in more than one study, irrespective of the bio-specimen analyzed. Conversely, a metabolite to be non-replicated if it was reported to be increased or decreased in only one study, irrespective of the bio-specimen analyzed in that study. All diagnosis-related metabolites are displayed in Supplementary Table 3, and the number of involved studies and bio-specimen of replicated diagnosis-related metabolites are given in Supplementary Table 8. Most of the replicated metabolites only appeared in two studies (Fig. 5b). A computer program, ClassyFire (http://classyfire.wishartlab.com/) was used to obtain the corresponding classification of metabolites^17^. The classification results indicated that the diagnosis-related metabolites of PD consisted of three inorganic compounds, including iodine, phosphate and potassium chloride, and 925 organic compounds. As shown in Fig. 5a, these organic compounds included 499 lipids, 69 of which were replicated; 189 organic acids, 55 of which were replicated; 66 organoheterocyclic compounds, 25 of which were replicated; 63 organic oxygen compounds, 16 of which were replicated; 45 benzenoids, 10 of which were replicated; 28 organic nitrogen compounds, eight of which were replicated; 35 other small class metabolites and seven of which were replicated. The progression-related metabolites of PD are shown in Supplementary Table 4. A total of 213 progression-related metabolites were identified, with 112 exclusively increased, 90 exclusively decreased, and 11 inconsistently changed metabolites. Only 14 metabolites were replicated, with eight consistently increased, five consistently decreased, and one inconsistently changed metabolites.

### Cross-matching with metabolic models

**Fig. 6:**
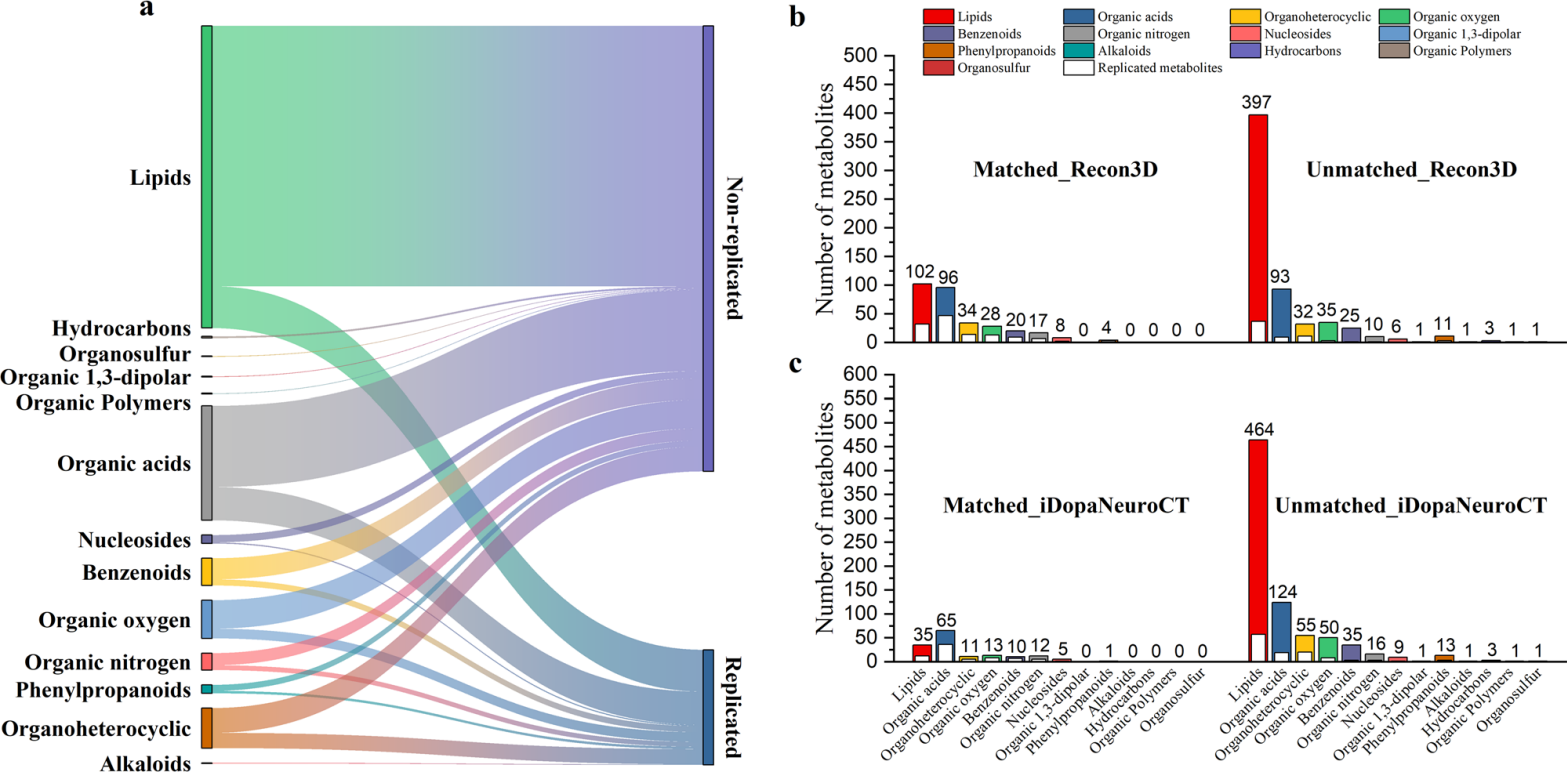
Organic compounds classification of PD diagnosis-related metabolites. **a** An overview of all organic compounds of PD diagnosis-related metabolites. **b** The organic compounds classification of the matched results with global human metabolism model (Recon3D). **c** The organic compounds classification of the matched results with the dopaminergic neuronal model (iDopaNeuroCT).

To explore dysfunctional pathways of PD, all diagnosis-related metabolites, including the replicated and non-replicated metabolites, were cross-matched with the metabolites in two established metabolic models, a global human metabolic network model Recon3D (Recon3D_3.01)^18^ and a cell-type specific model of dopaminergic neuronal metabolism (iDopaNeuroCT)^16^. Figure 5(c and d) displays the results of cross matching categorized by whether the metabolites were replicated or not, and whether they were consistently increased, consistently decreased, or inconsistently changed. The classification results of matched and unmatched metabolites are shown in Fig. 6.

A total of 311 metabolites were matched with metabolites in Recon3D, with 142 metabolites consistently increased, 74 metabolites consistently decreased, 95 matched metabolites with inconsistent changes. A total of 127 matched metabolites were replicated, with 45 consistently increased, 13 consistently decreased, and 69 inconsistently changed metabolites. Two inorganic compounds, iodine and phosphate, and 309 organic compounds can be matched. The matched organic compounds included 102 lipids, 32 of which were replicable; 96 organic acids, 47 of which were replicable; 34 organoheterocyclic compounds, 14 of which were replicable; 28 organic oxygen compounds, 13 of which were replicable; 20 benzenoids, 9 of which were replicable; 17 organic nitrogen compounds, 7 of which were replicable; 8 nucleosides and 4 phenylpropanoids. The matched results with the blood-brain barrier (BBB) metabolites shows that 34 of 43 BBB crossing metabolites and 13 of 240 BBB noncrossing metabolites could be matched. Of these matched metabolites, 27/34 BBB crossing metabolites were replicable and 3/13 noncrossing metabolites were replicable; the detail was shown in Supplementary Table 5.

A total of 153 metabolites were matched with the iDopaNeuroCT model, involving 65 organic acids and 35 lipids, with 61 consistently increased, 35 consistently decreased, and 57 inconsistently changed metabolites. A total of 75 matched metabolites were replicated metabolites, 22 of which were consistently increased, 8 of which were consistently decreased, and 45 replicated matched metabolites were inconsistently changed. All of these 153 metabolites could be found in the matched results with Recon3D.

The remaining 617 metabolites could not be matched with Recon3D, with 228 consistently increased, 368 consistently decreased, and 21 inconsistently changed metabolites. The presence of unmatched metabolites may be due to delays in updating the global human metabolism model. Therefore, it is necessary to add additional pathways to refine the global human metabolic network and better understand the metabolic perturbations of PD. The human biochemical metabolic reactions of the unmatched metabolites, especially non-lipid replicated metabolites, were manually searched in literature and public databases. Of the 617 metabolites, 37 replicated and 26 non-replicated metabolites could be found within at least one metabolic reaction, as shown in Supplementary Fig. 2a. However, most of these new biochemical reactions could not form metabolic pathways, only caffeine metabolism was identified as associated with PD. Therefore, caffeine metabolism was used to refine the global model.

As shown in Supplementary Fig. 2b, only 11 replicated progression-related replicated metabolites were matched with Recon3D, and eight replicated metabolites were matched with the iDopaNeuroCT model. Since only a few progression-related metabolites were replicable, more progression-related metabolic studies of PD are needed. Therefore, further analysis was only based on diagnosis-related metabolites of PD.

To generally understand the metabolic perturbations of PD, the most significantly dysfunctional pathways in Recon3D, including lipid (fatty acid, sphingolipid, and bile acid) metabolism, amino acids metabolism (ornithine, glutamine, glycine, tyrosine, tryptophan, and other significant amino acids), purine metabolism, organic oxygen metabolism, and other perturbed metabolic pathways that not in the Recon3D were reviewed before PD model generation based on the involved replicated metabolites and their corresponding changes.

### Lipid metabolic pathways perturbed in PD

Evidence has established that lipids function as structural components, energy molecules and signal messengers in living cells^19^, play important roles in many aspects of the pathogenesis of PD, ranging from oligomerisation and aggregation of α-Synuclein (α-Syn) to mitochondrial dysfunction^20^. Below, we summarized several lipid pathways of PD, including fatty acid metabolism, sphingolipid pathway and bile acid metabolism.

Accumulating evidence indicates that fatty acid metabolism is dysfunctional in PD patients^21–23^. Monounsaturated fatty acids, such as oleic acid, and polyunsaturated fatty acids, including linoleic acid, arachidonic acid, and docosahexaenoic acid, are essential for maintaining neuronal membrane permeability and regulating brain inflammation^24^. Four studies identified decreased levels of oleic acid in the blood of PD patients^22,23,25,26^, while seven studies identified increased levels of polyunsaturated fatty acids^22,27–32^, especially arachidonic acid^22,26,29–31^ in the plasma and CSF of PD patients. The weaker C-H bond at the bis-allylic position makes these fatty acids vulnerable to lipid peroxidation, and newly generated deleterious lipid peroxidation end products further influence cellular functions by altering membrane structure and circulating lipids^33^. The structure of α-Syn can immediately change to a α-helical conformation in the presence of both arachidonic acid and docosahexaenoic acid, which accelerates the aggregation of α-Syn and the formation of the oligomers that are harmful to neurons^24^.

The increase of acyl-carnitine metabolites^22,32,34,35^ in PD patients may result from the defects in the carnitine transport system^36,37^ and cause the dysregulation of fatty acid metabolism in PD patients^38^. For example, long-chain fatty acids (LCFAs) are required to transport into the mitochondrial matrix; this transport is carnitine-dependent and involves active translocation machinery^36^. Therefore, the accumulation of long-chain fatty acids in the blood of PD patients, such as hexadecanoic acid and octadecanoic acid^32,36,39^, may result from decreased fatty acid beta-oxidation due to the dysfunctional mitochondria. In addition, the metabolism of short-chain fatty acids (SCFAs), such as valeric acid^38,40,41^ and butyric acid^39–42^, has been reported to be changed in the plasma and feces of PD patients. SCFAs are the primary metabolites produced from dietary fiber by the fermentation of gut bacteria; they have part of the effects on maintaining intestinal barrier integrity and gut mucosal immunity^40^.

Perturbations of sphingolipid pathways have been identified in several studies of PD^27,28,43–45^. Sphingolipids are a kind of lipid containing a backbone of a sphingoid bases and aliphatic amino alcohols, including ceramides, sphingomyelins (SM), gangliosides, and cerebrosides; they are an integral part of cell signaling and regulation^21^. It has been reported that the perturbation of the sphingolipid pathway was an actual neurotoxic process following the aggregation of α-Syn^27,46^.

Sphingomyelin is a major myelin component that contains acyl chains with fatty acids; it is one of the constituents of the cellular membrane, and plays a role in inflammation, autophagy and cell death^43,45^. Several sphingomyelin molecules have been identified with increased^21,37,43,47^ or decreased levels^27,28,37,45,48–50^ in the CSF and plasma of PD patients. Besides, the genetic variants of PD, especially SMPD1, a gene that encodes sphingomyelin phosphodiesterase and leads to the accumulation of sphingomyelin, have been identified as associated with increased SM 26:0 blood levels^51^. However, three studies reported decreased levels of SM 26:0 in the CSF^37^ and plasma^44,45^ of PD patients.

Glycosphingolipids, one of the products in the sphingolipid pathway, have been shown to regulate fundamental cell properties and biological functions such as cell adhesion, cell growth, cell proliferation, autophagy, apoptosis and senescence^52^. The decreased levels of glycosphingolipids have been found in the plasma of PD patients^27,28^. Due to the bioactive role in cell membranes, glycosphingolipids can regulate blood-brain barrier permeability through the ordered regions in biological membranes, such as lipid rafts, which can serve as a potent inflammatory process regulator^24^.

Bile acids are mainly synthesized from cholesterol in the liver, and participate in cholesterol homeostasis^38^. These bile acids play critical roles in the digestion and absorption of other lipids in the small intestine; they are closely associated with intestinal hormones, microbiota, and energy balance^37^. Three studies identified the increased level of unconjugated bile acids in the plasma of PD patients^9,38,53^, and two studies identified the increased levels of secondary bile acids in the plasma of PD patients, such as lithocholic acid and deoxycholic acid^32,54^. These bile acids play critical roles in the digestion and absorption of other lipids in the small intestine; they are closely associated with intestinal hormones, microbiota, and energy balance^37^. Besides, ursodeoxycholic acid and its derivatives, such as tauroursodeoxycholic acid and glycoursodeoxycholic acid, have been reported with lower levels in PD animal models^55–57^. However, only one study reported decreased levels of glycoursodeoxycholic acid in the plasma of PD patients^38^, while another study reported increased levels of tauroursodeoxycholic acid in the plasma of PD patients^28^.

### Amino acids metabolic pathways perturbed in PD

Numerous amino acids significantly changed in different biospecimens of PD patients, and the most commonly changed amino acids are discussed, including ornithine, glutamine, glycine, tyrosine, tryptophan, and other significant amino acids metabolism.

Significantly increased levels of ornithine were observed in ten PD studies with different bio-fluids, including blood, urine and CSF^22,42,44,45,50,58–62^. Ornithine is derived from glutamine, synthesized from arginine and involved in the formation of urea^45^. The increased levels of ornithine may relate to the increased levels of urea^36,63^ through the urea cycle. Besides, the increased levels of proline were identified in the blood and urine in six studies^34,42,48,59,60,64^. Proline can also be generated from ornithine and glutamine^65^, establishing a tight association with these amino acids. The involved enzymes in proline metabolism, such as P5C reductases (PYCRs) and nicotinamide adenine dinucleotide (NAD)-dependent P5C dehydrogenase (P5CDH), have been reported to play important roles in neuronal function and brain structure^42^. In addition, the dysfunctional metabolism of ornithine was related to the polyamine synthesis in PD patients^60^. Polyamines, such as putrescine, spermidine, N1-acetylspermidine and spermine, participate in various biological processes such as cell proliferation, differentiation and division, and could act as antioxidant agents^66^. Increased metabolism of polyamines has been proposed to be due to high levels of oxidative stress in PD patients. Ornithine can be converted to putrescine by ornithine decarboxylase, and further affect the metabolism of polyamines in PD patients^67^. The increased ornithine may result in an increased level of putrescine^37,62^ and spermidine^35,62,68^.

Glutamine is the most abundant free amino acid; it has a critical role in mitochondria energy production, DNA damage response, apoptosis, and autophagy^69^. In PD patients, eight studies reported the increased levels of glutamine in the blood, urine and CSF^22,28,37,42,45,59,70,71^, while one reported decreased levels of glutamine in the CSF. Evidence indicates that glutamine can maintain the supply of neurotransmitter glutamate and gamma-aminobutyric acid (GABA) through the glutamine-glutamate-GABA metabolic cycle in the brain^70,72^. Six studies identified decreased levels of glutamate in the blood and feces^22,71,73–76^, while four studies identified increased levels of GABA in the plasma, urine and saliva of PD patients^34,35,73,77^. Normally, the released glutamate and GABA are taken up by astrocytes and rapidly converted to glutamine; these glutamines are then transported to the extracellular space and reabsorbed by glutamatergic and GABAergic neurons, where they are used to regenerate glutamate and GABA^78^. The inconsistenly changed levels of glutamine and glutamate in the brain may act as a protection mechanism for neurons from glutamate excitotoxic injury after striatal dopamine depletion^70^. In addition, GABA can be generated from glutamate by the enzyme glutamic acid decarboxylase (GAD) in the neuron, but this enzyme is also found in the insulin-secreting β cells in the pancreatic islets and further activating GABA receptors on the endocrine cells or immune cells^79^. Therefore, the increased levels of GABA in peripheral circulation may relate to inflammatory cell activation and immune dysregulation in PD patients^80^.

Six studies reported increased levels of glycine in the plasma, urine and CSF of PD patients^22,34,35,45,73,77^. As one of the inhibitory neurotransmitter amino acids^73^, glycine has been hypothesized to modulate the release of dopamine and glutamate^34,81^. Higher levels of glycine may indicate a neurotransmitter imbalance between dopaminergic and muscarinic cholinergic neurons^77^, and dopamine could induce glycine release from astrocytes to regulate neuronal excitability by reversing the function of astrocytic glycine transporter^81^. Moreover, glycine is involved in the synthesis of glutathione (GSH or gamma-glutamyl-cysteinyl-glycine) when combined with cysteine and glutamate^22^. Consistently increased glycine, glutamate and cysteine levels^22,59^ may indicate the dysfunction of glutathione synthesis in PD^77^. Furthermore, several glycine derivatives, as a kind of minor metabolites of fatty acids, such as hexanoylglycine^32,34,35^, phenylacetylglycine^34,35^, tiglylglycine^34,35^, furoylglycine^34,35^, were identified with increased levels in the urine of PD, indicating the dysfunctional fatty acid beta-oxidation in the PD patients^34^.

Perturbation of the phenylalanine/tyrosine/levodopa pathway has been identified in several PD metabolic studies. Tyrosine is derived from phenylalanine and converted to levodopa through phenylalanine hydroxylase and tyrosine hydroxylase, and then synthesized dopamine by aromatic amino acid decarboxylase^42^. Ten studies identified increased levels of tyrosine in the blood, saliva, urine, fecal and CSF of PD patients^22,34,42,45,48,61,62,71,77,82^. This increased changes was consistent with the increased levels of a primary tyrosine breakdown product, hydroxyphenylacetic acid, in five studies^27,28,34,35,83^. Eight studies reported increased levels of phenylalanine in the blood, saliva and urine of PD patients^34,38,42,50,71,77,84^, while three studies reported decreased levels of phenylalanine in the feces and serum of PD patients^22,74,85^. Both phenylalanine and tyrosine are precursors of dopamine^34^. Since phenylalanine and tyrosine can cross the blood-brain barrier, the increased levels of phenylalanine and tyrosine in peripheral circulation may indicate impairment in dopamine synthesis^42^. The increased precursors may indicate that the dopaminergic neurons in the brain affected by PD are trying to compensate for the depletion of dopaminergic neurons^62^. This compensation leads to an increased tyrosine lateral metabolism, ultimately resulting in higher levels of tyramine^21,82^. Besides, increased levels of levodopa^8^ and its metabolite, 3-methoxytyrosine (3-O-Methyldopa)^22,26,32,48,58,63,84^ and dopamine^37,45^, were affected by antiparkinsonian medications, such as levodopa^48^. However, only one study showed significantly changed levels of tyrosine in the cerebrospinal fluid of patients with PD^62^. More comprehensive studies of antiparkinsonian drugs are needed to explore the association with amino acids in the brain of PD patients. In addition, the increased levels of phenylacetyl-L-glutamine^22,34,35,49,86^, a phenylalanine derivative, was identified in the blood and urine of PD patients in five studies. Phenylacetyl-L-glutamine was derived from phenylalanine through gut microbiota by decarboxylation and then conjugated with glutamine^87,88^. Therefore, the increased levels of phenylacetyl-L-glutamine are highly associated with the metabolism of phenylalanine and glutamine in PD, and reflect the high activity of gut microbiota in PD patients.

Four studies reported decreased levels of tryptophan in the blood, feces and CSF of PD patients^25,74,85,89^, while one study reported that the level of urinary tryptophan was significantly increased in early-stage PD patients^34^. Tryptophan was reported to be involved in the generation of NAD + ^25^. The dysfunctional tryptophan metabolism has been found as associated with the impairment of brain energy metabolism^34,90^, which could induce increased oxidative stress in preclinical PD stage^25^. The increased or decreased tryptophan levels may be a result of the increased precursor degradation or excessive excretion of tryptophan in urine^48,74,89^. These changes can further lead to an increase or decrease in the corresponding catabolites, such as kynurenine^34,35,45^. Several studies have indicated that the imbalance of kynurenine metabolism plays an essential role in PD pathogenesis^45,83,91^. Kynurenine is a downstream metabolite of tryptophan, which can be further converted to 3-hydroxykynurenine or kynurenic acid^89^. Increased levels of kynurenic acid^89^ and 3-hydroxykynurenine^83,91^ have been identified in the blood of PD patients, indicating the increased kynurenine degradation. Besides, increased levels of 3-hydroxykynurenine may further lead to the depletion of dopamine neuons due to it is a neurotoxic compound that causes neuronal death^83^. In addition, based on the unmatched metabolites of PD, the products of kynurenine, 3- hydroxy-l-kynurenine^61^ and aminobenzoic acid^34^, were identified with increased levels, while the other products of tryptophan, indoleacetylglutamine^32^ and indolelactic acid^48^, were identified with decreased levels. These results emphasized the degradation of the kynurenine metabolism from tryptophan. Furthermore, tryptophan can also be degraded to 5-hydroxytryptophan^8,34,35,45^ by tryptophan hydroxylase^8^. Both 5-hydroxytryptophan and acetyl-serotonin are involved in serotonin pathways, which synthesize the monoamine neurotransmitters serotonin and melatonin^35^.

Elevated levels of alanine were observed in the plasma and CSF of PD patients in five clinical studies of PD^25,34,59,70,77^. Alanine can be synthesized from protein breakdown under fasting states, then transformed into pyruvate to synthesize glucose through gluconeogenesis in the liver^77^. The increased levels of alanine in PD patients may result from the dysfunction of the glucose-alanine cycle^59^, and affect the metabolism of Cori cycle^25,92^. The glucose-alanine cycle and the Cori cycle are crucial to gluconeogenesis and could increase glucose bioavailability in PD patients^92^. However, three studies identified the decreased levels of alanine in the plasma and CSF of PD patients^32,92,93^, two of which involved genetic PD patients, including PARK2^32^ and LRRK2^92^ PD patients. The identification of specific metabolic patterns is necessary for PD patients with different genetic mutations. In addition, increased levels of lysine were identified in the blood and urine of PD patients in four studies^34,44,59,71^, while decreased levels of lysine were identified in the plasma of PD patients in four studies^32,44,92,94^. The dysfunction of lysine in PD may relate to the dysfunction of polyubiquitin chain synthesis^95^ or reflect the imbalance of lysine acetylation^96^.

Increased levels of the branched-chain amino acids, such as histidine^35,37,59,70,77^ and isoleucine^23,34,35,37,42,59,70,77^ were identified in several studies of PD. Branched-chain amino acids play an important role in synthesizing neurotransmitters and maintaining the balance of nitrogen between astrocytes and neurons. Increased levels of histidine were related to neuronal damage in PD since it plays an integral part in neuronal transmission^70^. Metabolism of isoleucine was important in energy production and synthesis of the neurotransmitter glutamate, acetyl-CoA and proteins^42,70^.

Four studies indicated decreased levels of pipecolic acid in the blood and CSF of PD patients^26,83,91,94^. Pipecolic acid can act as a neurotransmitter to modulate gamma-amino butyric acid transmission; it could be taken up by cerebral mitochondria and induce neuronal apoptosis^97^. Besides, pipecolic acid is also involved in normal brain functions through the gut-brain axis^98^. The increased levels of trimethylamine N-oxide (TMAO)^34,35,77,99,100^ and p-Cresol sulfate^22,28–30,48,77^ in the blood, urine and CSF in PD patients may also reflect the dysfunction of gut-brain axis^99,101^.

### Purine metabolic pathways perturbed in PD

Metabolites involved in purine metabolism were identified with significant changes in different PD studies, including hypoxanthine, xanthine and uric acid. It was reported that uric acid is an endogenous antioxidant and end product of purine metabolism^102,103^, playing a role in protecting DNA from single-strand breaks caused by free radicals in alleviating oxidative damage in neurodegenerative diseases^104^. Hypoxanthine^34,35,53^ and xanthine^32,53,90^ are precursors of uric acid. Increased levels of hypoxanthine and xanthine may relate to the decreased levels of uric acid in PD patients^90,102^. The chronic impairment of mitochondrial function may contribute to the increased xanthine levels^105^. This impairment leads to increasing oxidative stress and energy dysfunction in PD patients, which can enhance the release and degradation of adenosine and ATP, and further increased levels of xanthine^105^. Besides, dopamine depletion in the brain of PD patients might also influence purine metabolism, resulting in increased levels of hypoxanthine and xanthine^106^. Besides, individuals consuming diets with increased serum uric acid have a lower risk of PD^107^. The inconsistent changes of purine metabolites may reflect the combined effects of endogenous and dietary factors^37,90^.

### Organic oxygen metabolic pathways perturbed in PD

The decreased levels of pantothenic acid in PD patients have been found in the plasma, serum, fecal and brain tissues of PD patients^23,48,58,83,108^. It was reported that pantothenic acid may associate with the neurodegenerative inflammation and oxidative stress in PD through the brain-gut axis^23^. However, metabolic modeling of the human gut microbiome predicted increased secretion of pantothenic acid in the human gut microbiota^13^, which is the converse of the changes in clinical studies of PD. Besides, pantothenic acid is a necessary precursor for coenzyme A (CoA) synthesis. CoA is a carbon transporter, required for pyruvate to be converted to acetyl-CoA before the citric acid cycle^23^. The decreased levels of pantothenic acid may reflect the energy dysfunction in PD patients^108^.

Energy dysfunction is highly related to the alterations in glucose metabolism in the pathogenesis of PD^109^. Increased levels of glucose^70,92,110^ were identified in the blood and CSF of PD patients in three studies, while decreased levels^58,91^ were identified in the plasma and CSF of PD patients in two studies. The energy requirements for metabolism in the brain are mainly glucose-dependent using NAD + . However, cellular NAD+ levels declines during the ageing process^111^ or due to the dysfunctional generation processes^25^, resulting in the decreased glucose utilization. Besides, increased glucose levels in blood are highly connected with type 2 diabetes mellitus (T2DM) in PD patients^112^. The treatment with dopaminergic medication, such as levodopa, may relate to glucose tolerance, hyperglycemia and hyperinsulinaemia but this association still remains controversial^112,113^.

Perturbations in glucose metabolism have been further identified through several significant metabolites in PD, including mannitol, glucitol, lactate, acetone, and pyruvate. The dysfunctional glucose levels may relate to the increased^34,114^ or decreased^59,75^ pyruvate levels through glycolysis, and the increased^22,37,70^ or decreased^92^ levels of lactate through the Cori cycle. Besides, the increased levels of mannitol^58,59^ and glucitol^59,114^ may imply the dysfunction of polyol metabolic pathways. The polyol pathway is a minor metabolic pathway of glucose running parallel to glycolysis, whose activity is altered in mitochondrial dysfunction^114^. Moreover, four studies found increased levels of acetone in serum, CSF and brain tissue of PD patients^37,50,70,71^. Acetone is a product formed from acetoacetic acid during fatty acid beta-oxidation^70^. Increased fatty acid beta-oxidation metabolism in the brain may be used as a compensatory energy metabolism in PD^71^.

### Other perturbed metabolic pathways not present in Recon3D

Based on the unmatched replicated metabolites, caffeine metabolism was identified as dysfunctional in several studies. Caffeine is a natural chemical compound with stimulant effects^115^. It is contained in coffee, tea, and cocoa, and is widely used in various energy drinks and medical products. Once the caffeine has been ingested, it is quickly absorbed by the gastrointestinal tract, and the blood concentrations of caffeine are highest 30 min after ingestion^116^. A similar trend in caffeine concentration is found in the brain, which means that caffeine can be transferred from blood to the brain through the rapid crossing of the blood-brain barrier due to its hydrophobic characteristics^115^.

Several studies have reported decreased levels of caffeine and its downstream metabolites^32,58,103,117,118^ in the blood of PD patients. One study^103^ indicated that reduced levels of caffeine in PD patients may partly result from PD patients consuming significantly less caffeine compared to healthy controls. However, in other studies^58,83,106,118^, the lower levels of caffeine and its decomposition products in PD patients were identified with no association with caffeine consumption in participants after adjusting for coffee intake^58,106^.

Caffeine is a known antagonist of adenosine A2A receptors^118^. Adenosine A2A receptors are highly expressed in the basal ganglia and the limbic brain region, which tightly interacts structurally and functionally with the dopamine D2 receptor to modulate the function of movement^119^. The activity of Adenosine A2A receptors may associate with pathogenic processes in neurodegenerative illnesses, such as excessive glutamate release, the aggregation of toxic protein species, activation of the indirect pathway of the cortico-striato-thalamo-cortical loop and disrupting the redox homeostasis^120,121^. The effects of caffeine in the substantia nigra could be competitive inhibition with adenosine binding to adenosine A2A receptors.

Moreover, the Cytochrome P450 enzymes, such as Cytochrome P450 Family 1 Subfamily A Member 2 (CYP1A2) and Cytochrome P450 Family 1 Subfamily E Member 1 (CYP2E1), are involved in the caffeine metabolic pathway, which can affect the efficiency of caffeine metabolism^32^. However, the prevalence of single-nucleotide polymorphism of CYP1A2 and CYP2E1 has no difference between PARK2 patients and control subjects^32^. Besides, it was difficult to exclude the possible influence of drug therapy, such as levodopa or other antiparkinsonian drugs that influence the collateral hypermetabolism of caffeine^117^. More independent studies are needed to explore the therapeutic potential of caffeine for PD treatment.

Other four unmatched replicated metabolites, including trigonelline, catechol sulfate, pyroglutamine and homovanillate sulfate, indicated that the metabolic changes of PD were also involved in drug metabolism, food metabolism, protein degradation, and gut microbiota metabolism in PD patients.

Trigonelline or N-methyl nicotinic acid, a non-purine constituent in coffee that serves to produce specific aroma compounds, was identified with lower levels of concentration in the plasma of PD^32,48,63,103^. The decreased levels of trigonelline indicated that there was a difference in coffee intake between PD patients and healthy controls. It was reported that trigonelline could be partially transformed into nicotinic acid during the roasting process, form into vitamins and be involved in human metabolism^122^. Besides, the pharmacological activities of trigonelline have been explored in central nervous system disease, especially its neuroprotective effects^123,124^. The biochemical reactions of trigonelline in humans need to be further explored.

Catechol sulfate is an end product of the metabolism of benzoate metabolism^125^. The biological pathway of catechol sulfate in PD patients is currently unclear. The decreased levels of catechol sulfate may be associated with abnormal gut microbiota in PD patients^32,58,125^; it has been reported to be involved in the combined activity of gut microbial metabolism and liver and kidney functions such as constipation and dysphagia^58^.

Pyroglutamine, a cyclic derivative of glutamine involving glutamine metabolism^126^, belongs to the class of organic compounds known as alpha amino acids and derivatives. Two studies reported increased levels of pyroglutamine in the blood of PD patients^22,58^, which may relate to the dysfunctional glutamine metabolism of PD. Besides, the research on the association between antihypertensive and lipid-lowering drugs with human metabolism indicated that the increased levels of pyroglutamine were positively associated with the concentration of beta-blockers, but negatively associated with fiber intake^127^.

Increased levels of homovanillate sulfate were reported in two studies^32,58^. Homovanillate sulfate or homovanillic acid sulfate, a primary metabolite of catecholamine metabolism, is located downstream of the levodopa metabolite, which was associated with levodopa intake and affected by the metabolism of levodopa medication^58^. Besides, some phenolic acids, such as caffeic acid, and vanillic acid, are found in the outer bran layer of wheat grains^128^. The increased levels of homovanillate sulfate may br associated with the metabolic conversion of wheat grains dietary by microbial enzymes or endogenous enzymes in the liver^128^.

### PD model

Considering the wide range of metabolism dysfunctions observed in PD patients, a PD model was then generated to gain a comprehensive understanding of these metabolic connections. To better describe the metabolic perturbations of PD, additional human metabolic reactions were added to generate a refined global model, Recon3.02, which contains 4,213 metabolites and 13,950 reactions, including all reactions from Recon3D, 277 new reactions from Human1^129^, 14 reactions from the iDopaNeuroCT model, 73 fatty acid oxidation reactions and 43 caffeine metabolism reactions, with all the additional metabolites and reactions listed in Supplementary Table 6 and 7. Starting with Recon3.02, a de-compartmentalized and thermodynamically flux-consistent global subset was generated to represent a generic model that not specific for any cell types or bio-specimens, containing 2828 metabolites and 5602 reactions. Of 137 replicated metabolites, 8 were excluded as they did not correspond to any thermodynamically consistent network flux. These excluded metabolites belonged to gut microbiota metabolites, such as trimethylamine N-oxide (TMAO), p-Cresol sulfate, p-Cresol, glutaric acid and indoxyl sulfate. The remaining 129 metabolites were designated as core metabolites to build a functional network around using an established model generation pipeline, XomicsToModel^130^.

**Fig. 7:**
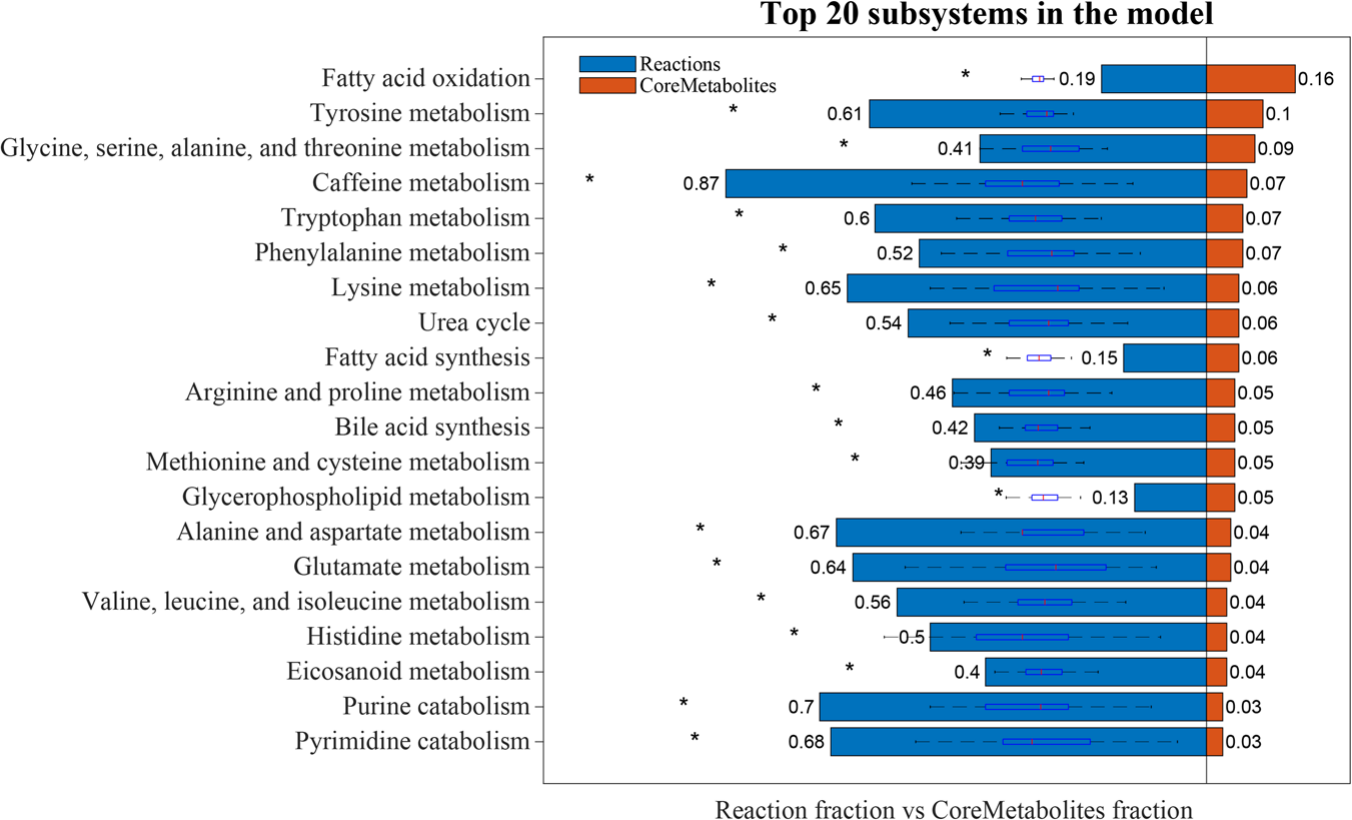
The top 20 subsystems in the PD model, ordered by the proportion of core metabolites involved in each subsystem. The decimal number on the reaction bars of each subsystem represents the fraction between the reaction number in the PD model and in the de-compartmentalized thermodynamically flux-consistent global subset; the decimal number on the core metabolite bars represents the proportion of the core metabolites involved in each subsystem out of the 137 core metabolites. The box plots represent the random distribution of reaction fraction in each subsystem, *P-value < 0.001 using Student *t*-test (*α* = 0.05, two side).

An ensemble of models was generated containing 1689 shared reactions (except for newly added exchange reactions) and 129 core metabolites. The heat maps for overlapped metabolites, reactions and genes between the randomly generated 10 models are shown in Supplementary Figure 2; the overlapped proportion for metabolites, reactions and genes between each pair of models can reach ˜90%. Then, the genome-scale metabolic PD model was extracted using the shared reactions, which contained 1689 reactions, 910 metabolites, and 808 metabolic genes, involving 82 subsystems. We ordered the subsystems according to the proportion of the core metabolites out of all 129 core metabolites contained in each subsystem, and the top 20 subsystems are shown in Fig. 7. Most of the significant pathways related to the replicated metabolites were visualized in a planar map ignoring the exchange reactions, which can help us to better understand the relationships between the replicated and non-replicated metabolic changes in PD, as shown in the Supplementary Figure 3. Non-diagnosis metabolites that are part of this PD metabolic model represent predictions of potential metabolites that may be relevant for PD but perhaps have not yet been measured due to the incomplete coverage of existing metabolomic platforms.

**Fig. 8:**
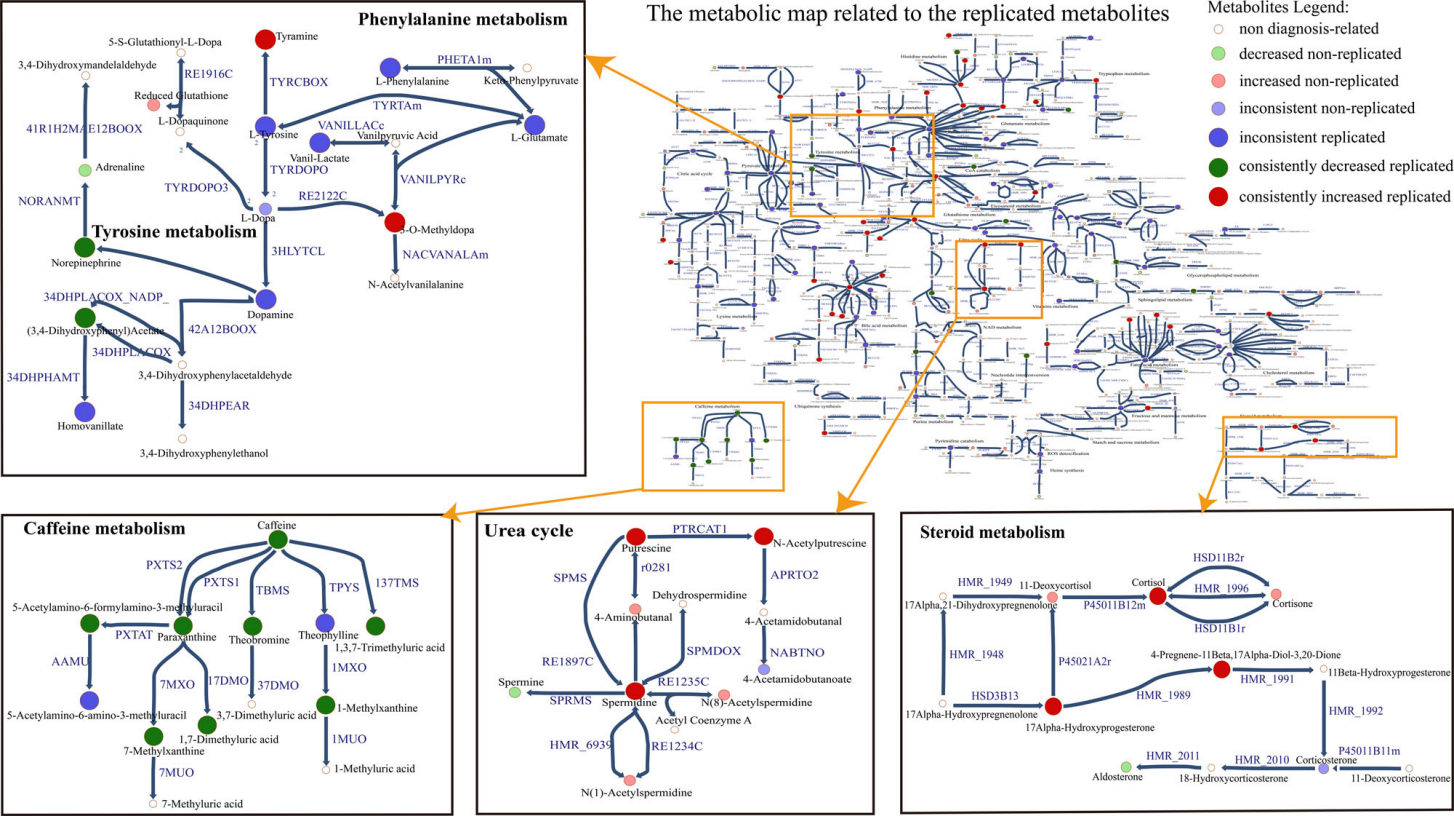
The overview map related to the replicated metabolites and four consistent pathways. Each disc represents a metabolite with non-white colors representing metabolites associated with PD diagnosis, with different changes and degree of replication, while white disks represent metabolites that have not been reported to be associated with PD diagnosis (non-diagnosis related metabolites). Non-diagnosis related metabolites are juxtaposed to many metabolites that are diagnosis-related and hypothesized to be relevant to the pathogenesis of PD, especially if they are not generally targeted by established metabolomic platforms. Zoom in to view details of additional metabolic pathways.

Since most of the metabolites were performed with inconsistent changes, the pathways with consistently changed metabolites were identified through the map and highlighted in the Fig. 8, including dopamine metabolism (tyrosine metabolism), polyamine metabolism (urea cycle), steroid metabolism and caffeine metabolism.

## Discussion

Of 84 studies reviewed in total, 10 diagnosis-related studies were omitted from further consideration as they scored below thresholds following externally specified quality control assessments, specifically QUADOMICS^131,132^ and the Newcastle-Ottawa Scale^133^. In the QUADOMICS results, most below-threshold studies did not provide a detailed description of sample inclusion and exclusion criteria; they failed to control potential factors affecting the metabolomic profiles during sample collection or they were unable to perform validation in an independent cohort. In the Newcastle-Ottawa Scale results, below-threshold studies were undermined by sample selection bias, such as hospital admission rate bias, failure to describe the source of control samples, or failure to control additional factors besides age and sex between cases and controls.

Metabolites reported to be changing in PD were matched with the metabolites of established human computational models^18^. However, it was difficult to match many of the reported lipids due to differences between the level of resolution and annotation of lipids in the human metabolic model and in the lipidomic data^134^. For example, triglycerides (TGs) are a lipid class that is composed of a large number of lipid molecules with different structures. For lipidomic data, classes such as TG (52:2), or species such as TG (17:0 17:2 18:0) or TG (17:1 17:1 18:0), could only be mapped to an entity in the model (’tag_hs’) that represents a lipid class rather than a lipid with a uniquely specified structure. Therefore, further refinement of lipid metabolism in the metabolic model and more detailed specifications of the lipids measured in clinical samples is necessary before computational modeling can be used to interpret PD-associated lipidomic changes.

According to the definitions of replicated and non-replicated metabolites, around 20% of PD diagnosis-related metabolites were replicated. Most non-replicated metabolites were lipids, which may be because lipids have complex structures that are difficult to analytically identify as the same metabolite with different platforms. In this study, the statistics of replicated metabolites are irrespective of the biospecimen being analyzed due to a considerable number of metabolites could be converted to non-replicated metabolites if we split biospecimens. Indeed, most studies employed different metabolomic platforms, which resulted in diverse metabolomic profiles. Besides, it is worth noting that most studies do not report measured but unchanged metabolites. Instead, they focus on reporting significantly changed metabolites identified through their statistical method. Consequently, a metabolite is not deemed non-replicated based on being only increased or decreased once in one specific biospecimen cause it could be reported with the same changes in different biospecimens. These altered metabolites are still considered to be replicated in this study and could be validated in future metabolomic studies.

Besides, almost one-third of replicated metabolites were inconsistently changed across different studies. One of the reasons for this inconsistency may be caused by various bio-specimens of included metabolomic studies. For example, taurocholic acid was considered a large molecule that cannot cross the blood-brain barrier (BBB). The inconsistent changes in taurocholic acid, including increased levels in plasma^28,48^ and decreased levels in CSF^37^, may be caused by the natural barrier. Besides, pathological changes in the permeability of the BBB may contribute to changes in metabolite levels, such as several triacylglycerols and derivatives, which are BBB impermeable metabolites but identified with increased levels in both plasma and CSF of PD patients^21,43,135^.

Inconsistency may also arise due to drug intake, as certain metabolites, such as ornithine and putrescine, can be influenced by antiparkinsonian drugs (Alexander B et al., in preparation). Genetic mutations are also known to be a significant contributing factor to this inconsistency, as the concentration of several long-chain fatty acids are known to be influenced by GBA mutations^25^. Use of different analytical platforms or different sample preparation procedures may also lead to reports of inconsistent changes in metabolite levels. Reduced and elevated levels of plasma lysine were identified in the same study using nuclear magnetic resonance and mass spectrometry platforms, respectively^44^. Pre-analytical variation in human plasma and serum may also contribute to inconsistencies^136^. For example, the level of several metabolites including hypoxanthine, guanosine, inosine and eicosanoids may change as a function of time interval between sampling and centrifugation^136^.

In addition, disease progression may also result in inconsistencies. For example, methionine and leucine were identified as significantly changed in concentration over time in the plasma of PD patients^84^; their concentrations were higher in PD compared to the controls at baseline but then decreased to be lower than the controls at follow-up^84^. This change may result from the changed levels of the precursor by oxidative stress or gut microbial pathways during PD progression^84^. Due to the limited progression-related studies, especially longitudinal metabolomic studies, we did not draw any conclusions on progression-related metabolites. However, we do conclude that additional longitudinal studies of PD metabolism are warranted.

In general, the low percentage of replication and consistency revealed that reported biomarkers are highly heterogeneous. This heterogeneous picture may result from complex factors affecting metabolite levels, such as different ethnicity, diet, exercise levels and medication intake, different metabolite annotation databases and statistical analysis methods used in different metabolic studies. Merging different subtypes of PD patients, different detection platforms and bio-specimens, may also contribute to such heterogeneity. To better understand common dysfunctional pathways of PD, we assumed that the replicated metabolites were more reliable biomarkers than other non-replicated metabolites in this study. As more precise PD metabolomic studies are conducted in the future, these replicated metabolites can be updated accordingly.

The identified replicated, diagnosis-related metabolites were used to specify core metabolites of the newly generated, PD-specific metabolic model. The semi-automated model generation process biased for inclusion of pathways that enabled steady-state flux involving PD-associated metabolites but relaxed this requirement for highly connected metabolites that were not associated with PD. Therefore, the PD model focuses on PD-associated pathways rather than pathways for synthesis and degradation of non-PD-related metabolites that are omnipresent in metabolism. As a result, pathways associated with PD were included in the model, while pathways for cofactor synthesis were excluded, unless that cofactor was associated with PD.

The metabolic subsystems enriched in the PD model were broadly reflective of the dysfunctional pathways summarized in the literature synthesis. Most significant metabolic pathways in PD model are primarily involved in six pathways: (1) Tyrosine metabolism. It contained 14 replicated metabolites, five of which are highly replicated. The highly replicated metabolites are metabolites that appeared in more than three studies with the same changes. These highly replicated metabolites included 3-O-Methyldopa, glycine, tyrosine, hydroxyphenylacetic acid and glutamine. (2) Caffeine metabolism. It contained 10 replicated metabolites, with six highly replicated metabolites, caffeine, paraxanthine^32,58,103,117,118^, 1-methylxanthine^32,83,103,117,118^, theophylline^32,58,83,93,103,117,118^, 1,7-dimethyluric acid^32,58,117,118^ and 5-acetylamino-6-amino-3-methyluracil^32,58,83,117,118,137^. (3) Tryptophan metabolism. It contained nine replicated metabolites, four of which were highly replicated, including 5-hydroxytryptophan, glutamate, tryptophan and alanine. (4) Phenylalanine metabolism. It contained nine replicated metabolites, six of which were highly replicated, including glycine, glutamine, phenylacetyl-L-glutamine, hydroxyphenylacetic acid, phenylalanine and glutamate. (5) Lysine metabolism. It contained eight replicated metabolites, five of which were highly replicated, including glycine, glutamate, pipecolic acid, acetate^40,42,71,77,100,113,114^ and lysine. (6) The urea cycle. It contained eight replicated metabolites, three of which were highly replicated, including ornithine, glycine and acetate. These pathways reflecting the dysfunctional metabolism in PD patients are mainly related to the neurotransmitter metabolism and caffeine metabolism.

Except for these pathways involving highly replicated metabolites, other pathways with consistent metabolic changes in the map, including steroid metabolism, polyamine metabolism and dopamine metabolism, could be used to explore potential metabolic markers further. Dysfunction of steroid metabolism was identified with cortisol and its related metabolites, including cortisone, 11-deoxycortisol, 17-hydroxyprogesterone and 21-Deoxycortisol, increased in PD patients^22,34,35,48^. Stress-sensitive cortisol, produced by the adrenal cortex, may affect motor and non-motor symptoms of PD^138^. Increased stress in the neurodegenerative process may induce the dysfunctional hypothalamic-pituitary-adrenal (HPA) axis and further influence cortisol levels; the increased cortisol levels may relate to mitochondrial dysfunction and the vulnerability of nigral neurons^139,140^. Besides, the dysfunction of sphingolipid metabolism may affect steroidogenesis metabolism^46^. Sphingolipids are strongly connected with sterol metabolism through the physical association of the planar ring of sterols with the acyl chains of sphingolipids; they may interact through the cross-talk on metabolic path- ways, resulting in increased steroid levels^141^. Therefore, cortisol and its related metabolites could have the potential to be the biomarkers to reflect dysfunctional lipid metabolism and the stress in the brain leading to the disruption of mitochondrial function and neuroinflammation^140^.

As shown in the map, putrescine and its downstream metabolites, including spermidine, 4-Aminobutanal^94^ and N-acetylputrescine^68,94^, were identified with increased levels in PD patients. Meanwhile, the lateral metabolism from spermidine to N1-acetylspermidine^68^, N8-acetylspermidine^68^ increased in PD patients, while the metabolism from spermidine to spermine was identified to be decreased^68^. These consistently changed polyamines could be used as potential biomarkers to reflect oxidative stress in PD; and the decreased metabolism from spermidine to spermine and its involved protein activity needs further exploration.

The PD model reflects the classical biosynthesis and degradation pathways of dopamine, where dopamine comes from phenylalanine/tyrosine/L-Dopa, and then degrades to norepinephrine or homovanillic acid^142^. One of the direct dopamine products, 3,4-dihydroxyphenylacetaldehyde (DOPAL), has been reported to accumulate in the brain of PD patients through the increased dopamine conversion to DOPAL by monoamine oxidase and decreased DOPAL degradation by aldehyde dehydrogenases, which may play an important role in the aberrant accumulation of α-Syn^143^. However, our study was limited to analyzing most of the metabolomic studies of PD patients. Hence, other relevant studies, such as the paper to explore the dysfunction of catechols in PD patients^144^ were not included, leading to the insignificance of DOPAL in our study. Therefore, more studies are needed to focus on the dopamine metabolites in the brain of PD patients. In contrast, the intermediate dihydroxyphenylacetic acid (DOPAC), which is also the product of DOPAL, was consistently reported with decreased changing levels, and plays an important role in several biological processes^145^. Both norepinephrine^73,82^ and dihydroxyphenylacetic acid (DOPAC)^73,146^ were consistently decreased in PD patients no matter whether the dopamine level was increased or decreased. Therefore, norepinephrine and dihydroxyphenylacetic acid could be used as potential markers to identify the loss of dopaminergic neurons^145^.

Except for the highly replicated metabolites in caffeine metabolism, other caffeine downstream metabolites, including theobromine, 1,3,7-trimethyluric acid, 7-methylxanthine and 5-acetylamino-6-formylamino-3-methyluracil, may also be considered as possible metabolic markers to reflect the dysfunction of caffeine metabolism as they are consistently decreased in PD patients.

In conclusion, our analysis limited metabolic perturbations associated with all PD patients, without distinguishing between genetic and idiopathic, treated versus untreated, or other subtypes of PD patients. Different subtypes of PD patients may result in perturbation to different subsets of the pathways associated with PD. Additional stratification analysis based on different subtypes of PD, different bio-specimens and metabolomic detection platforms would be possible with consistent access to raw data for each of the 74 studies that passed quality control assessments. A more detailed analysis of all of the individual raw metabolomic data and metadata from high-quality past and ongoing clinical metabolomic studies will be necessary to enable a paradigm shift from mono to multi-centric clinical metabolomic analysis of PD. Moreover, more longitudinal metabolomic studies are needed to explore the metabolic changes in PD progression.

Given that the metabolic biomarkers in PD patients are significantly impacted by various factors, it is necessary to avoid potential biases and control such factors in future PD metabolomic studies. When designing the metabolite detection method, careful consideration is required, as different detection platforms exhibit different sensitivity and separation power for different metabolites. Besides, inherent barriers may lead to inconsistent metabolic changes in PD patients across different bio-specimens, and dynamic metabolite changes may occur before sample centrifugation. Therefore, the adoption of standard analytical techniques and sample preparation operations are needed to be able to more precisely compare metabolomic studies. Additionally, it is essential to consider the different metabolomic changing patterns in various PD patient classifications, such as genetic or idiopathic PD patients, early- or late-stage PD patients, treated and untreated PD patients, due to some metabolites may change over time at different stages or be influenced by antiparkinsonian drugs. Also, the selection of an appropriate control group is crucial in obtaining accurate results, taking into account factors such as age onset or the impact of daily diet. Moreover, several metabolites have highlighted gut microbiota changes in PD, and further research is necessary to elucidate the association between the gut-brain axis and PD.

## Methods

### Study search and data collection

Metabolomic studies of PD were selected till March 2021 from two public databases, PubMed and Web of Science, with the combination of search keywords ’Parkinson’s disease’ and ’Metabolomics’ in all fields. The studies based on all PD patients, including genetic and idiopathic, treated and untreated PD patients, were included in this study. No limitations were placed on analytical platforms and bio-specimens used in PD metabolomic studies. The following studies were excluded: studies that only concerned cell culture, animal models and drug therapy; 2) review papers; 3) studies without any diagnosis-related or progression-related metabolites. Then, the target metabolomic studies of PD were updated from March 2021 to March 2022 to extend the study number.

In this study, the diagnosis-related metabolites were considered as the metabolites with different abundances between PD patients and asymptomatic controls. Due to the limited number of longitudinal metabolomic studies available, our definition of progression-related metabolites has been expanded to include metabolites that are associated with PD severity, motor score, and disease duration, or metabolites with changed abundances in follow-up cohorts. Basic information, including publication year, country, sample size, analytical platforms of metabolites, type of specimen, and possible diagnosis-related or progression-related metabolites with increased or decreased changing trends compared to controls were collected from each selected study.

### Quality assessment

To assess the reliability of the included results, the quality assessment of selected studies was applied by combining the tools Newcastle-Ottawa Scale (NOS)^133^ and QUADOMICS^132^. Newcastle-Ottawa Scale (NOS) is widely used for assessing the quality of non-randomized studies in meta-analyses^133^. It consisted of three parts: sample selection, comparability, and outcome with 9 questions. Each question can be awarded 1 score if it is ’yes’, and each study can get a maximum of 9 scores. The studies that scored greater than 6 were considered to be relative high-quality^147^.

QUADOMICS, a quality assessment panel specified for the ”omics”-based diagnostic research, was generated from Quality Assessment tool for Diagnostic Accuracy Studies (QUADAS)^131,132^. Except for the assessment of study design, this tool was also focused on sample collection, pre-analytical and analytical procedures, and statistical analysis. In this study, the 10/16 questions related to metabolomic were selected to assess the quality of PD metabolomic studies^148^. Each item is quantified as 1 score if it is ’yes’, and each study can get a maximum of 10 scores. The studies that scored greater than 7 were considered to be relatively high-quality. Any study that was assessed as high-quality by both tools was deemed a high-quality study. A random subset with eight studies was assessed by an independent clinical research fellow to validate the quality assessment consistency. Any disagreement on these assessment items was discussed to establish a consensus.

### Metabolite classification and matching to human metabolic databases

As collected metabolites were represented by different identifiers in different studies, for example, a metabolite may be reported as butyric acid in a clinical metabolomic study but butyrate in another study, a Virtual Metabolic Human identifier (VMH ID) was used to unify metabolite into a single namespace.

The Virtual Metabolic Human (VMH, https://www.vmh.life/) is a metabolic database linking genes, reactions and metabolites into human metabolic models, where each metabolite is identifiable with database dependent and independent identifiers^148^. It is necessary to identify the corresponding VMH ID for each metabolite to explore the metabolic pathways involved, as the VMH namespace is commonly used for each of the established metabolic models.

Uncertain metabolites, including fragments that could not be identified or annotated accurately, were excluded from this study. Then, the classification of each metabolite was obtained to identify the metabolite characteristics. A computer program, ClassyFire (http://classyfire.wishartlab.com/)^17^, which uses chemical structures and structural features to automatically assign all known chemical compounds to taxonomy, was used to obtain the corresponding classification through the InChI string and InChI key that was collected from the VMH database for each metabolite. The metabolites without InChI string and InChI key, especially lipids, were manually classified to the closest parent class.

Statistical analysis for the metabolites with different changes was performed to explore the consistency and inconsistency of PD metabolites. As the example shown in Table 1, we defined the replicated and non-replicated metabolites with consistent and inconsistent changes. A metabolite is considered to be *replicated* when it has been reported to be increased (or decreased) relative to controls in more than one study. We define a metabolite to be consistent when it has only been reported to increase (or decrease) in all involved studies, while a metabolite to be inconsistent when it has been reported to increase and decrease in different studies. Therefore, a metabolite is consistently replicated when it is only reported to increase (or decrease), while inconsistently replicated when it is reported to increase (or decrease) in more than one study but decrease (or increase) in at least one study. In this study, each of these definitions is irrespective of the type of biospecimen where the changes were reported, therefore a metabolite could be replicated if it was reported to increase in one study of plasma samples and another study of CSF samples.

**Table 1:** The example of the replicated and non-replicated metabolites in this study

Metabolites	VMH ID	Increased_Frequency	Decreased_Frequency	Replicated or Non-replicated
Tyramine	tym	2	0	Replicated (consistently)
Glycerophosphocholine	g3pc	0	2	Replicated (consistently)
Uridine	uri	1	3	Replicated (inconsistently)
Ubiquinone-1	q10	0	1	Non-replicated (consistently)
cholate	cholate	1	0	Non-replicated (consistently)
Sphinganine 1-phosphate	sph1p	1	1	Non-replicated (inconsistently)

As the blood-brain barrier (BBB) can prevent the transportation of some molecules, 43 known metabolites that can cross the BBB and 240 known metabolites that cannot pass the BBB, were matched with the collected metabolites of PD^14,135^. Besides, all collected metabolites were cross-matched with the metabolites in two established computational models using VMH ID, the global human metabolism model (Recon3D or Recon3D_3.01)^18^ and the dopaminergic neuronal cell-type metabolism model (iDopaNeuroCT model)^16^ to explore existed dysfunctional pathways of PD. The corresponding biochemical metabolic reactions in human beings of the unmatched metabolites, especially non-lipid replicated metabolites, were manually searched in literature and public databases to identify metabolic pathways associated with PD.

### Generating a PD model

A global genome-scale metabolic model, Recon3D (Recon3D_3.01, https://www.vmh.life/#downloadview)^18^, was used to generate the PD model. Recon3D is a genome-scale model of human metabolism, it contains 4,140 unique metabolites and 13,543 metabolic reactions, representing the activity of 3,697 metabolic genes, and provides information about gene-protein-reaction associations that connected metabolic genes with enzymes and metabolic reactions^18^. To better describe the metabolic perturbations of PD, additional metabolic reactions of human beings were added to refine the global model, including new human metabolic reactions from the human metabolic model, Human1^129^, and new neuronal reactions from a dopaminergic neuronal metabolic model (iDopaNeuroCT model)^16^. Besides, new reactions of fatty acid oxidation metabolism were added to extend lipidomic of the global model, and new reactions associated with PD pathways missing from Recon3D that were added before PD model generation.

An established context-specific metabolic model extraction pipeline ‘XomicsToModel’ was used to generate a thermo- dynamically flux-consistent model^130^. This pipeline has been used to generate the dopaminergic neuronal models^16^; it enables the integration of omics data, including genomic, transcriptomic, proteomic, metabolomic, and bibliomic data, and the extraction of a physicochemically consistent mechanistic model from a global metabolic network^130^. In the ‘XomicsToModel’ pipeline, the ’thermoKernel’ algorithm was used to extract the thermodynamically flux-consistent model, with the Gurobi v9.1.2 (Gurobi Optimization, LLC) as the linear optimization solver and with the ‘fastcc’ algorithm^149^ to check the flux consistency. The smallest non-zero flux was set to 1e-5 (parameter epsilon of XomicsToModel), and other parameters were set as default as described in the paper of German et al.^130^.

All the compartments were removed from the refined global model to explore the connection of metabolites better, and the reactions of ‘Artificial reactions’, ‘R group synthesis’ and ‘Pool reactions’ subsystems were removed from the global model due to these reactions cannot reflect the connection of specific metabolites. Then, a thermodynamically flux-consistent global subset of the extended Recon3D model, was extracted to shorten the running time before the PD model generation, and the default weight of all the metabolites and reactions in the subset was set to 0.01 at the beginning.

To get a compact model containing most of the pathways related to diagnosis-related metabolites of PD, the replicated metabolites were used as core metabolites. The weight of each core metabolite was calculated using the corresponding study number divided by 74 (total number of diagnosis-related studies), where the maximum study number was used if the metabolite changed in different changes. Then, the weight of core metabolites was converted to a negative value. The negative weight value promotes a metabolite or reaction to be present in the model, the positive value promotes a metabolite or reaction to be absent, and zero means ambivalence with respect to presence or absence^130^.

Since the PD model was only generated based on a limited input set of core metabolites, alternate optimal models resulted in the variability of replicate-generated models given the same input data. Therefore, we used a semi-steady state approach to identify relatively stable dysfunctional pathways of PD. A weight of zero was set for highly connected metabolites, except for the core metabolites, where the highly connected metabolites were defined as the metabolite connected with more than 9 reactions. Exchange reactions of these highly connected metabolites were added to relax them in the model if there were no corresponding exchange, demand or sink reactions in the global model. Then, the reaction weights were heuristically set through iterations: Ten models were generated without specifying reaction weights in the first iteration. Then, we identified overlapping reactions for these ten models and set their weight to −1.1, where newly added exchange reactions of which were excluded to avoid the model becoming too large. Next, the updated reaction weights were added to the next iteration to explore more overlapped reactions until the reaction weight was stable and all overlapped reactions were more than 85% of reactions from any randomly generated model.

The final relatively stable reactions with the weight were used to extract the core PD model, and the most significant pathways of replicated metabolites were visualized in the Escher map (https://escher.github.io/#/)^150^ to reflect the connectivity of the dysfunctional metabolites and further explore the possible PD metabolic markers. In the map, the metabolites were highlighted with their corresponding changes. To validate that the PD model was highly connected with core metabolites, we randomly generated extra 100 models with the same reaction size as the PD model from the global subset to identify the random distribution of reactions on each subsystem. One sample T-test was used to explore if there is any difference between the reaction proportion of random distribution and the reaction proportion of the PD model on each subsystem.

Constraint-based modeling process was implemented using the latest COBRA Toolbox^12^. The solver and algorithms were compatible with the COBRA Toolbox, and all of these calculations were conducted using MATLAB 2021a (MathWorks Inc.). The figures involved in this study were generated using OriginLab software (OriginLab Corporation, Northampton, MA, USA), MATLAB and R programming language.

### Supplementary information

supplementary information

## Data Availability

All data generated or analyzed during this study are included in this published article and its supplementary information files. The analysis codes used in this study is available in COBRA repository and can be accessed via this link: https://github.com/opencobra/COBRA.papers/2023_metaPD.
