# Supplementary material for: Identification of metabolites reproducibly associated with Parkinson’s Disease via meta-analysis and computational modelling
Source: NPJ Parkinsons Dis. 2024 Jun 29;10:126. doi: 10.1038/s41531-024-00732-z (PMC11217404; doi:10.1038/s41531-024-00732-z)
Supplement: Supplementary file 1 — supplementary information [file 41531_2024_732_MOESM1_ESM.pdf]

Content

Supplementary Table 1: Selected metabolomic studies of PD 2
Supplementary Table 2: Quality assessment results of selected studies using QUADOMICS and NOS tools. 5
Supplementary Table 3: All 928 diagnosis-related metabolites.(Note: the ID was represented by VMH ID or Pubchem ID or name) 14
Supplementary Table 4: All 213 progression-related metabolites. 87
Supplementary Table 5: Blood brain barrier metabolites. 99
Supplementary Table 6: The newly added metabolites in global model refinement. 108
Supplementary Table 7: The newly added reactions in global model refinement. 111
Supplementary Table 8: The replicated diagnosis-related metabolites. 130
Supplementary Figure 1. PD progression-related metabolites. 138
Supplementary Figure 2. Statistics of PD metabolites. 138
Supplementary Figure 3. The heat maps for overlapped metabolites, reactions and genes between the randomly generated 10 models. 139
Supplementary Figure 4. The raw PD map. 140

Supplementary Table 1: Selected metabolomic studies of PD

Type	Num	Author	Title	Year	Country	Case number	PD class	Control number	Control type	Biospecimens	Detection Method	Diagnosis-related (Y=yes)	Progression-related (Y=yes)	Excluded by quality assessment (Y=yes)
	1	Dong	Lipid metabolic dysregulation is involved in Parkinson's disease dementia	2021.03	China	36/32	PDND;PDD	41	Health control	Plasma	Untargeted LC-MS	Y		
	2	Dong	Metabolomics profiling reveals altered lipid metabolism and identifies a panel of lipid metabolites as biomarkers for Parkinson's disease related anxiety disorder	2021.02	China	35/35	PDNA;PDA	35	Health control	Plasma	Untargeted LC-MS	Y		
	3	Shao	Comprehensive metabolic profiling of Parkinson's disease by liquid chromatography-mass spectrometry	2021	China	223(three cohort)	PD	169/68	Health control and PD-unrelated neurological disease control (NDC)	Plasma	UPLC-MS	Y		
	4	Toczylowska	Changes in the metabolic profiles of the serum and putamen in Parkinson's disease patients - In vitro and in vivo NMR spectroscopy studies	2020	Poland	19	PD	21	Health control	Serum/putamen	NMR/MRS	Y		
	5	Hu	Integrated Metabolomics and Proteomics Analysis Reveals Plasma Lipid Metabolic Disturbance in Patients With Parkinson's Disease	2020	China	36	PD	36	Health control	Plasma	Untargeted LC-MS	Y		
	6	Yakhine-Diop	Metabolic alterations in plasma from patients with familial and idiopathic Parkinson's disease	2020	Spain	24	PD	8	Health control	Plasma	GC-MS/MS and UHPLC/MS	Y		Y
	7	Molsberry	Plasma Metabolomic Markers of Insulin Resistance and Diabetes and Rate of Incident Parkinson's Disease	2020	USA	349	PD	349	Health control	Plasma	U-HPLC-MS	Y		
	8	Chen	The Gut Metabolite Trimethylamine N-oxide Is Associated With Parkinson's Disease Severity and Progression	2020	China	60	PD	30	Health control	Plasma	LC-MS/MS	Y	Y	
	9	Saiki	A metabolic profile of polyamines in parkinson disease: A promising biomarker	2019	Japan	145/186	PD	45/49	Health control	Plasma/serum	HPLC-MS	Y	Y	
	10	Hertel	Integrated Analyses of Microbiome and Longitudinal Metabolome Data Reveal Microbial-Host Interactions on Sulfur Metabolism in Parkinson's Disease	2019	Ireland	30	PD	30	Health control	Plasma	UPLC-MS/MS;GC-MS	Y	Y	
	11	Glaab	Integrative analysis of blood metabolomics and PET brain neuroimaging data for Parkinson's disease	2019	Germany	60	PD	15	neurologically healthy controls	Plasma	GC-MS	Y	Y	
	12	Okuzumi	Metabolomics-based identification of metabolic alterations in PARK2	2019	Japan	15	PD	19	Health control	Plasma	UHPLC/MS	Y		
	13	Kim	Upgraded Methodology for the Development of Early Diagnosis of Parkinson's Disease Based on Searching Blood Markers in Patients and Experimental Models	2019	Russia	36	PD	52	Health control	Plasma	HPLC-ED	Y		
	14	Chang	Alternations of Metabolic Profile and Kynurenine Metabolism in the Plasma of Parkinson's Disease	2018	China	82	PD	82	Health control	Plasma	LC-TOFMS	Y	Y	
Diagnosis-related studies	15	Balashova	Plasma Metabolome Signature in Patients with Early-stage Parkinson Disease	2018	Russia	16	PD	20	controls without neurodegenerative disease	Plasma	DIMS	Y		
	16	Zhao	Potential biomarkers of Parkinson's disease revealed by plasma metabolic profiling	2018	China	28	PD	18	Health control	Plasma	UPLC-Q-TOF-MS	Y		Y
	17	Fiandaca	Potential Metabolomic Linkage in Blood between Parkinson's Disease and Traumatic Brain Injury	2018	USA	40	PD;PDD	20	Health control	Serum	HPLC-MS	Y		
	18	Saiki	Decreased long-chain acylcarnitines from insufficient β-oxidation as potential early diagnostic markers for Parkinson's disease	2017	Japan	109/145	PD	32/45	Health control	Plasma	CE-TOFMS; LC-TOFMS	Y		
	19	Hatano	Identification of novel biomarkers for Parkinson's disease by metabolomic technologies	2016	Japan	35	PD	15	Health control	Plasma	UPLC-MS/MS, GC-MS	Y		
	20	Johansen	Metabolomic Profiling in LRRK2-Related Parkinson's disease	2009	Norway	53	PD	31+15	Health control	Plasma	LCECA	Y		
	21	Ahmed	Metabolic profiling of Parkinson's disease: evidence of biomarker from gene expression analysis and rapid neural network detection	2009	India	43	PD	37	Health control	Plasma	NMR	Y		
	22	Bogdanov	Metabolomic profiling to develop blood biomarkers for Parkinson's disease	2008	USA	66	PD	25	Health control	Plasma	LCECA;HPLC	Y		
	23	Calvani	A novel multi-marker discovery approach identifies new serum biomarkers for Parkinson's disease in older people: an EXosomes in PArkiNson Disease (EXPAND) ancillary study	2020	Italy	20	PD	30	Health control	Serum	UPLC-MS	Y		
	24	Cirstea	Microbiota Composition and Metabolism Are Associated With Gut Function in Parkinson's Disease	2020	Canada	75	PD	50	Health control	Serum	UPLC-MS	Y		
	25	Picca	Circulating amino acid signature in older people with Parkinson's disease: A metabolic complement to the EXosomes in PArkiNson Disease (EXPAND) study	2019	Italy	20	PD	30	Health control	Serum	UPLC-MS	Y		
	26	Nagesh Babu	Serum metabolomics study in a group of Parkinson's disease patients from northern India	2018	India	17	PD	22	Health control	Serum	NMR	Y		
	27	Han	Profiling novel metabolic biomarkers for Parkinson's disease using in-depth metabolomic analysis	2017	China	43	PD	42	Health control/PDID	Serum	HPLC-TOFMS	Y	Y	
	28	Burté	metabolic profiling of Parkinson's disease and mild cognitive impairment	2017	UK	41	PD	40	asymptomatic matched controls	Serum	UHPLC-MS/MS	Y		Y
	29	Roede	Serum metabolomics of slow vs. rapid motor progression Parkinson's disease: a pilot study	2013	USA	80	PD	20	Health control	Serum	LC-Fourier transform MS	Y	Y	

30	Schulte	Alterations in Lipid and Inositol Metabolisms in Two Dopaminergic Disorders	2016	Germany	82	PD	1272	Health control	Serum	LC/GC-MS/MS	Y		
31	Crotty	Association of caffeine and related analytes with resistance to Parkinson disease among LRRK2 mutation carriers: A metabolomic study	2020	USA	188	PD	180	LRRK2+(115)/LRRK2-(65) UC.	Plasma/CSF	LC-MS	Y		
32	Stoessel	Promising Metabolite Profiles in the Plasma and CSF of Early Clinical Parkinson's Disease	2018	Germany	80/40	PD	76/37	Health control	Plasma/CSF	LC-MS/MS	Y		
33	Havelund	Changes in kynurenine pathway metabolism in Parkinson patients with L-DOPA-induced dyskinesia	2017	Denmark	26	PD	14	Health control	Plasma/CSF	LC-MS	Y		
34	Wuolikainen	Multi-platform mass spectrometry analysis of the CSF and plasma metabolomes of rigorously matched amyotrophic lateral sclerosis, Parkinson's disease and control subjects	2016	Sweden	22	PD	28	Control with other neurological conditions	Plasma/CSF	GC/MS, LC/MS	Y		
35	Trupp	Metabolite and peptide levels in plasma and CSF differentiating healthy controls from patients with newly diagnosed Parkinson's disease	2014	Sweden	20	PD	20	Health control	Plasma/CSF	GC-TOFMS	Y		
36	Bolner	Plasma and urinary HPLC-ED determination of the ratio of 8-OHdG/2-dG in Parkinson's disease	2011	Italy	21	PD	13	Health control	Plasma/urine	HPLC-ED	Y		Y
37	Abe	Alteration of 8-hydroxyguanosine concentrations in the cerebrospinal fluid and serum from patients with Parkinson's disease	2003	Japan	24	PD	15	controls were neurologically normal patients	Serum/CSF	HPLC-ED	Y	Y	Y
38	Michell	Metabolomic analysis of urine and serum in Parkinson's disease	2008	UK	23	PD	23	Health control	Serum/urine	GC-MS	Y		
39	Troisi	A metabolomic signature of treated and drug-naive patients with Parkinson disease: A pilot study	2018	Italy	100	PD	42	Health control	whole blood	GC-MS	Y	Y	
40	Yilmaz	Metabolic Profiling of CSF from People Suffering from Sporadic and LRRK2 Parkinson's Disease: A Pilot Study	2020	Norway	40	SPD; LRRK2 PD	40	Control; LRRK2 control	CSF	NMR; DI-LC-MS/MS	Y		
41	Lucio	Integrative Metabolomic and Metallomic Analysis in a Case-Control Cohort With Parkinson's Disease	2019	Germany	33	PD	101	Health control	CSF	PPE-FT-ICR-MS	Y		
42	Willkommen	Metabolomic investigations in cerebrospinal fluid of Parkinson's disease	2018	Germany	31	PD	95	Health control	CSF	PPE-FT-ICR-MS	Y		
43	Trezzi	Distinct metabolomic signature in cerebrospinal fluid in early parkinson's disease	2017	Germany	44	PD	43	Health control	CSF	GC-MS	Y		
44	Öhman	NMR metabonomics of cerebrospinal fluid distinguishes between Parkinson's disease and controls	2015	Sweden	10	PD	10	controls were patients where CSF was collected for analysis of different disorders other than neurodegenerative diseases	CSF	NMR	Y		
45	Lewitt	3-hydroxykynurenine and other Parkinson's disease biomarkers discovered by metabolomic analysis	2013	USA	48	PD	57	Control samples were from 57 brains lacking clinical or neuropathological evidence for CNS disorders	CSF	UHPLC-MS/MS2; GC/MS	Y		
46	LeWitt	CSF xanthine, homovanillic acid, and their ratio as biomarkers of Parkinson's disease	2012	USA	217	PD	26	Health control	CSF	HPLC-EAS	Y		
47	Tan	Gut Microbial Ecosystem in Parkinson Disease: New Clinicobiological Insights from Multi-Omics	2021	Malasiya	77	PD	77	Health control	Fecal	NMR and LC-MS	Y		
48	Vascellari	Gut Microbiota and Metabolome Alterations Associated with Parkinson's Disease	2020	Italy	64	PD	51	Health control	Fecal	GC-MS	Y		
49	Kumari	Identification of potential urine biomarkers in idiopathic parkinson's disease using NMR	2020	India	100	PD	50	Health control	urinary	NMR	Y	Y	
50	Luan	Comprehensive urinary metabolomic profiling and identification of potential noninvasive marker for idiopathic Parkinson's disease	2015.09	China	92	PD	65	normal control	urinary	GC-MS; LC/MS	Y	Y	
51	Luan	Elevated excretion of biopyrrin as a new marker for idiopathic Parkinson's disease	2015.11	China	92	PD	65	normal control	urinary	LC/MS;ELASA	Y		
52	Luan	LC-MS-based urinary metabolite signatures in idiopathic Parkinson's disease	2015.01	China	106	PD	104	Health control	urinary	UHPLC/MS	Y		
53	Sinclair	Metabolomics of sebum reveals lipid dysregulation in Parkinson's disease	2021	UK	218	PD	56	Health control	other:sebum	LC-MS	Y		
54	Trivedi	Discovery of Volatile Biomarkers of Parkinson's Disease from Sebum	2019	UK	43	PD	21	Health control	other:sebum	DHS–TD–GC–MS	Y		Y
55	Kumari	Quantitative metabolomics of saliva using proton NMR spectroscopy in patients with Parkinson's disease and healthy controls	2020	India	76	PD	37	Health control	other:saliva	NMR	Y		
56	Wood	Augmented frontal cortex diacylglycerol levels in Parkinson's disease and Lewy Body Disease	2018	USA	15	PD	43	Health Control	other:Frontal cortex	MS	Y		Y
57	Griffith	Reduced brain glutamate in patients with Parkinson's disease	2008	USA	12	PD	12	age-matched, neurologically normal control participants	other: posterior cingulate gyrus	MRS	Y		
58	D'Andrea	Different Circulating Trace Amine Profiles in De Novo and Treated Parkinson's Disease Patients	2019	Italy	48	PD	10	Health control	Plasma	UPLC-MS/MS	Y	Y	
59	Hamid	Gender specific decrease of a set of circulating N-acylphosphatidyl ethanolamines (NAPEs) in the plasma of Parkinson's disease patients	2019	Italy	114/149	PD	142/114	Health control	Plasma	LC–MS/MS	Y		
60	Takeshige-Amano	Shared Metabolic Profile of Caffeine in Parkinsonian Disorders	2019	Japan	111/160	PD	43/31	Health control	Serum	HPLC-MS	Y		
61	Kumari	Metabolomic analysis of serum using proton NMR in 6-OHDA experimental PD model and patients with PD	2020	India	99	PD	50	Health control	Serum	NMR	Y	Y	

	62	Ohmich	Biomarker repurposing: Therapeutic drug monitoring of serum theophylline offers a potential diagnostic biomarker of Parkinson’s disease	2018	Japan	31	PD	33	disease controls	Serum	in-vitro chemiluminescent microparticle immunoassay	Y		Y
	63	Fujimaki	Serum caffeine and metabolites are reliable biomarkers of early Parkinson disease	2018	Japan	108	PD	31	Health control	Serum	LC–MS	Y		Y
	64	Li	Gut Microbiota Dysbiosis Is Associated with Elevated Bile Acids in Parkinson's Disease	2021	USA	12	PD	16	Health control	Tissue:human appendix, ileum	UPLC-TQ-S-MS	Y		
	65	Fernández-Irigoyen	Alteration in the Cerebrospinal Fluid Lipidome in Parkinson's Disease: A Post-Mortem Pilot Study	2021	Spain	20	PD	10	Health control	CSF	UHPLC-TOF-MS	Y		
	66	Silvia	Plasma acyl-carnitines, bilirubin, tyramine and tetrahydro-21-deoxycortisol in Parkinson's disease and essential tremor. A case control biomarker study	2021	Spain					Plasma	UPLC-MS	Y	Y	
	67	Aaron	Human Brain Lipidomics: Pilot Analysis of the Basal Ganglia Sphingolipidome in Parkinson's Disease and Lewy Body Disease	2022.2	USA	7	PD	9	control	Brain tissue was obtained via autopsy of volunteer subjects	electrospray ionization (ESI)-MS/MS	Y		Y
	68	KH Chang	Alterations of Sphingolipid and Phospholipid Pathways and Ornithine Level in the Plasma as Biomarkers of Parkinson's Disease	2022.1	Taiwan,China	92	PD: 71 patients at early stage and 21 patients at advanced stage	60	60 sex- and age-matched HC	Plasma	LC/MS	Y	Y	
	69	Szu-Ju Chen	Association of Fecal and Plasma Levels of Short-Chain Fatty Acids With Gut Microbiota and Clinical Severity in Patients With Parkinson Diseas	2022.2	Taiwan,China	96	PD	85	normal control	fecal and plasma	LC-MS/MS ; GC-MS	Y	Y	
	70	Eduardo De Pablo-Fernandez	The faecal metabolome and mycobiome in Parkinson's disease	2022.2	UK	35	PD	35/15	primary/secondary control	Fecal	GC-MS	Y		
	71	Dong	Plasma Metabolite Signature Classifies Male LRRK2 Parkinson's Disease Patients	2022.2	USA	32	male PD with a LRRK2 G2019S mutation	42	male non-PD approximately age/sex-matched controls without LRRK2 mutations	Plasma	NMR	Y		
	72	Carolina Gonzalez-Riano	Prognostic biomarkers of Parkinson's disease in the Spanish EPIC cohort: a multiplatform metabolomics approach	2021.8	Spain	39	Pre-PD	39	healthy control	plasma	LC-MS; GC-MS	Y		
	73	Stephan Klatt	A six-metabolite panel as potential blood-based biomarkers for Parkinson's disease	2021.10.	Australia	103	iPD	93	control	serum	LC/MS	Y		
	74	Gaia Meoni	Metabolite and lipoprotein profiles reveal sex-related oxidative stress imbalance in de novo drug-naive Parkinson's disease patients	2022.2	Italy	228	de novo drug-naive PD patients (dn2PD)	79	healthy control	serum	NMR	Y		
	75	Meerakhan Pathan	Plasma Metabolite Markers of Parkinson's Disease and Atypical Parkinsonism	2021.12	Sweden	34	PD	31	control	plasma	NMR and MS	Y		
	76	Szymon Plewa	The Metabolomic Approach Reveals the Alteration in Human Serum and Cerebrospinal Fluid Composition in Parkinson's Disease Patients	2021.9	Poland	11	PD	10	control	serum/CSF	LC-MS/MS	Y		
	77	Dorines Rosario	Systematic analysis of gut microbiome reveals the role of bacterial folate and homocysteine metabolism in Parkinson's disease	2021.3	UK	26	PD	25	11 were healthy controls (COs) while 14 were diseased controls (DCs) with cardiovascular risk factors.	fecal	MS	Y		
	78	Melissa Scholefield	Severe and Regionally Widespread Increases in Tissue Urea in the Human Brain Represent a Novel Finding of Pathogenic Potential in Parkinson's Disease Dementia	2021.10.	UK	9	PDD	9	control	Brain tissue was obtained via autopsy of subjects	UHPLC-MS/MS	Y		
	79	Melissa Scholefield	Substantively Lowered Levels of Pantothenic Acid (Vitamin B5) in Several Regions of the Human Brain in Parkinson's Disease Dementia	2021.8	UK	9	PDD	9	control	Brain tissue was obtained via autopsy of subjects	UHPLC-MS/MS	Y		
	80	Yan	Alterations of gut microbiota and metabolome with Parkinson's disease	2021.11	China	20	PD	20	helathy control	Fecal	GC-MS	Y		
	81	Zhang	Targeted fatty acid metabolomics to discover Parkinson's disease associated metabolic alteration	2021.10.	China	42	PD	54	healthy volunteers	plasma	UPLS-MS	Y	Y	
	82	Chan	Elevated GM3 plasma concentration in idiopathic Parkinson's disease: A lipidomic analysis	2017	USA	150	PD	100	helathy control	Plasma	HPLC-MS	Y		
	83	Thomas Kremer	Longitudinal Analysis of Multiple Neurotransmitter Metabolites in Cerebrospinal Fluid in Early Parkinson's Disease	2021.08	Switzerland	95	PD	56	helathy control	CSF	LC-MS/MS	Y		
	84	Anastasia Bougea	Serum Uric Acid in LRRK2 Related Parkinson's Disease: Longitudinal Data from the PPMI Study	2021	Greece	144	LRRK2 + PD patients	199	helathy control	Serum	other: PPMI database	Y		
	85	LeWitt	Metabolomic biomarkers as strong correlates of Parkinson disease progression	2017	USA	49	PD	-	final line of PD	Plasma/CSF			Y	
Independent progression-related studies	86	Monika Figura	Serum amino acid profile in patients with Parkinson’s disease	2018	Poland	73	early PD/ late PD with dyskinesia/ late PD wihtout dyskinesia			Serum			Y	
	87	Hila Avisar	Lipidomics Prediction of Parkinson's Disease Severity: A Machine-Learning Analysis	2021	Israel	149	PD			plasma			Y	

Supplementary Table 2: Quality assessment results of selected studies using QUADOMICS and NOS tools.

	QUADOMICS											
	Item	1	3	4	5	6	8	10	11	15	16	Total
	Item question	Were selection criteria clearly described?	Was the type of sample used fully described?	Were the procedures and timing of biological sample collection with respect to clinical factors described with enough detail?(1. Clinical and physiological factors 2. Diagnostic and treatment procedures)	Were handling of specimens and pre-analytical procedures reported in sufficient detail and similar for the whole sample? And if differences in procedures were reported, was their effect on the results assessed?	Is the time period between reference standard and index test short enough to be reasonably sure that the target condition did not change between the two tests?	Did the whole sample or a random selection of the sample, receive verification using a reference standard?	Was the execution of the index test described in sufficient detail to permit replication of the test?	Was the execution of the reference standard described in sufficient detail to permit its replication?	Were uninterpretable/intermediate test results reported?	Is it likely that overfitting was avoided?	

	How to score (y, means 'yes'/n, means 'no/not clear')	sources of samples, selection criteria and a flow diagram are included along with a comparison between included and excluded patients	the report should present a detailed description of the type of sample (serum, plasma, other body fluids, tissue, etc.). Moreover, the authors should specifically list the type of plasma specimen (e.g., EDTA, heparin, citrate), since they could give different results.	These items would be scored as "yes" if the study includes an analysis of potential factors affecting the protein/metabolite/peptide profile (confounding factors), and a procedure to control biases that they may induce (for instance, stratification). Otherwise, these criteria should be scored as "no".	Any process related to the pre-analytical handling of the samples that could affect the results should be described, and a comparison of the results according to the different procedures be supplied (number of freezing cycles, type of anticoagulant, timing and storing of specimens, time from blood draw until centrifugation and storage, details on centrifugation conditions, etc.). Otherwise, authors should state that the whole set of samples has undergone the same pre-analytical process.	For such conditions this item should be scored "yes" if the delay between the performance of the index and reference standard is very short, a matter of hours or days. However, for chronic conditions disease status is unlikely to change in a week, or a month, or even longer. In such conditions longer delays between performance of the index and reference standard may be scored as "yes".	If it is clear from the study that all patients, or a random selection of patients, who received the index test went on to receive verification of their disease status using a reference standard then this item should be scored as "yes". This item should be scored as yes even if the reference standard was not the same for all patients. If some of the patients who received the index test did not receive verification of their true disease state, and the selection of patients to receive the reference standard was not random, then this item should be scored as "no". If this information is not reported by the study then it should be scored as "unclear".	Authors should follow the recommendations for reporting each technique, such as International standards for reporting metabolomic experimental results. Studies published before the availability of these guidelines should cover basic aspects as: Mass-spectrometry: Description of the use of particular technologies: column chromatography, capillary electrophoresis, the use of software to analyze MS data and gel electrophoresis (and its processing and analysis). It should also cover molecular interaction experiments and statistical analysis of data.	If the study reports sufficient details or citations to permit replication of the index test and reference standard then these items should be scored as "yes". In other cases these items should be scored as "no". In situations where details of test performance are partially reported and you feel that you do not have enough information to score this item as "yes", then it should be scored as "unclear".	If it is clear that all test results, including uninterpretable/indeterminate/intermediate are reported then this item should be scored as "yes". If the authors do not report any uninterpretable/indeterminate/intermediate results, and if results are reported for all patients who were described as having been entered into the study then this item should also be scored as "yes". If you think that such results occurred but have not been reported then this item should be scored as "no". If it is not clear whether all study results have been reported then this item should be scored as "unclear".	This item will be scored as "yes" if the authors performed a validation test in an independent set of samples or used some approach to deal with overfitting(cross-validation/resampling methods). However, if the study used the same sample for the test and training set, it should be scored 'no'.	
1	Dong	y	y	n	y	y	y	y	y	n	y	8
2	Dong	y	y	n	y	y	y	y	y	n	y	8
3	Shao	y	y	y	y	y	y	y	y	y	y	10
4	Toczylowska	y	y	n	y	y	y	y	y	y	y	9
5	Hu	y	y	n	y	y	y	y	y	n	y	8
6	Yakhine-Diop	n	y	y	y	y	y	y	y	n	n	7
7	Molsberry	y	y	y	y	y	y	y	y	y	n	9

8	Chen	y	y	y	y	y	y	y	y	y	n	9
9	Saiki	y	y	y	y	y	y	y	y	n	n	8
10	Hertel	y	y	y	y	y	y	y	y	y	n	9
11	Glaab	y	y	y	y	y	y	y	y	y	y	10
12	Okuzumi	y	y	n	y	y	y	y	y	y	n	8
13	Kim	y	y	n	y	y	y	y	y	y	n	8
14	Chang	y	y	y	y	y	y	y	y	y	y	10
15	Balashova	y	y	n	y	y	y	y	y	y	n	8
16	Zhao	y	y	n	y	y	y	y	y	n	n	7
17	Fiandaca	y	y	n	y	y	y	y	y	n	y	8
18	Saiki	y	y	y	y	y	y	y	y	y	y	10
19	Hatano	y	y	y	y	y	y	y	y	y	n	9
20	Johansen	y	y	y	y	y	y	y	y	y	y	10
21	Ahmed	y	y	n	y	y	y	y	y	y	n	8
22	Bogdanov	y	y	y	y	y	y	y	y	n	n	8
23	Calvani	y	y	y	y	y	y	y	y	y	y	10
24	Cirstea	y	y	y	y	y	y	y	y	y	n	9
25	Picca	y	y	n	y	y	y	y	y	y	y	9
26	Nagesh Babu	y	y	y	y	y	y	y	y	y	y	10
27	Han	y	y	n	y	y	y	y	y	y	y	9
28	Burté	n	y	n	y	y	y	y	y	y	y	8
29	Roede	y	y	n	y	y	y	y	y	y	n	8
30	Schulte	y	y	n	y	y	y	y	y	y	n	8
31	Crotty	y	y	y	y	y	y	y	y	y	n	9
32	Stoessel	y	y	y	y	y	y	y	y	y	y	10
33	Havelund	y	y	n	y	y	y	y	y	y	n	8
34	Wuolikainen	y	y	n	y	y	y	y	y	y	y	9
35	Trupp	y	y	n	y	y	y	y	y	y	n	8
36	Bolner	n	y	n	y	y	y	y	y	y	n	7
37	Abe	n	y	n	y	y	y	y	y	y	n	7
38	Michell	y	y	y	y	y	y	y	y	n	y	9
39	Troisi	y	y	y	y	y	y	y	y	n	y	9

40	Yilmaz	y	y	n	y	y	y	y	y	y	y	9
41	Lucio	y	y	y	y	y	y	y	y	y	y	10
42	Willkommen	y	y	y	y	y	y	y	y	y	y	10
43	Trezzi	y	y	y	y	y	y	y	y	y	y	10
44	Öhman	n	y	n	y	y	y	y	y	y	y	8
45	Lewitt	y	y	n	y	y	y	y	y	y	y	9
46	LeWitt	y	y	y	y	y	y	y	y	y	n	9
47	Tan	y	y	y	y	y	y	y	y	n	n	8
48	Vascellari	y	y	n	y	y	y	y	y	y	n	8
49	Kumari	y	y	y	y	y	y	y	y	n	y	9
50	Luan	y	y	y	y	y	y	y	y	n	y	9
51	Luan	y	y	n	y	y	y	y	y	y	n	8
52	Luan	y	y	y	y	y	y	y	y	n	y	9
53	Sinclair	n	y	y	y	y	y	y	y	n	y	8
54	Trivedi	n	y	n	y	y	y	y	y	n	y	7
55	Kumari	y	y	y	y	y	y	y	y	y	y	10
56	Wood	n	y	n	y	y	y	y	y	y	n	7
57	Griffith	y	y	n	y	y	y	y	y	y	n	8
58	D'Andrea	y	y	y	y	y	y	y	y	y	y	10
59	Hamid	y	y	y	y	y	y	y	y	y	y	10
60	Takeshige	y	y	y	y	y	y	y	y	y	y	10
61	Kumari	y	y	y	y	y	y	y	y	n	n	8
62	Ohmich	y	y	y	y	y	y	y	y	y	n	9
63	Fujimaki	n	y	n	y	y	y	y	y	y	n	7
64	Li	y	y	y	y	y	y	y	y	y	n	9
65	Fernández-Irigoyen	y	y	y	y	y	y	y	y	y	n	9
66	Silvia	y	y	y	y	y	y	y	y	y	y	10
67	Aaron	n	y	n	y	y	y	y	y	y	n	7
68	Chang	y	y	y	n	y	y	y	y	y	y	9
69	Chen	y	y	y	y	y	y	y	y	n	n	8
70	Eduardo	y	y	y	y	y	y	y	y	y	n	9
71	Dong	y	y	y	y	y	y	y	y	y	y	10

72	Carolina	y	y	n	y	y	y	y	y	y	n	8
73	Klatt	n	y	n	y	y	y	y	y	y	y	8
74	Meoni	y	y	y	y	y	y	y	y	y	y	10
75	Pathan	y	y	n	y	y	y	y	y	y	y	9
76	Plewa	y	y	n	y	y	y	y	y	y	n	8
77	Dorines	n	y	n	y	y	y	y	y	y	n	7
78	Scholefield	n	y	y	y	y	y	y	y	y	n	8
79	Scholefield	y	y	y	y	y	y	y	y	y	n	9
80	Yan	y	y	n	y	y	y	y	y	y	n	8
81	Zhang	y	y	y	y	y	y	y	y	y	y	10
82	Chan	n	y	y	y	y	y	y	y	y	y	9
83	Thomas	y	y	n	y	y	y	y	y	y	n	8
84	Anastasia	y	y	y	y	y	y	y	y	y	n	9

	NOS										
	**Iten num**	**Selection (4)**				**Comparability (2)**		**Exposure (3)**			**Total**
	Item question	case definition	Representativeness	Selection of Controls (hostipal.../no description)	Definition of Controls	Study controls for the most important factors	Study controls for any additional factor	Ascertainment of metabolites	Same method of metabolites ascertainment for cases and controls	Same in non-Response Rate	
	How to score (y, means 'yes'/n, means 'no/not clear')	If there is a clear case definition.	If the selected case can representative population	If there is a description about control source	If there is a clear control definition.	If the study controls the comparability of age and sex between cases and controls.	If the study controls the comparability of other factors between cases and controls.	If there is detailed description of metabolomics	Same method of metabolites ascertainment for cases and controls	Same in non-Response Rate	
1	Dong	y	n	y	y	y	y	y	y	y	8
2	Dong	y	n	y	y	y	y	y	y	y	8
3	Shao	y	n	y	y	y	n	y	y	y	7
4	Toczylowska	y	n	y	y	y	n	y	y	y	7
5	Hu	y	n	y	y	y	y	y	y	y	8
6	Yakhine-Diop	y	n	y	y	y	n	y	y	y	7
7	Molsberry	y	n	y	y	y	y	y	y	y	8
8	Chen	y	n	y	y	y	n	y	y	y	7
9	Saiki	y	n	y	y	y	y	y	y	y	8
10	Hertel	y	y	y	y	y	y	y	y	y	9
11	Glaab	y	n	y	y	y	y	y	y	y	8

12	Okuzumi	y	n	y	y	y	y	y	y	y	8
13	Kim	y	n	y	y	y	n	y	y	y	7
14	Chang	y	n	y	y	y	y	y	y	y	8
15	Balashova	y	n	y	y	y	n	y	y	y	7
16	Zhao	y	n	y	y	y	n	y	y	y	7
17	Fiandaca	y	n	y	y	y	n	y	y	y	7
18	Saiki	y	n	n	y	y	y	y	y	y	7
19	Hatano	y	n	y	y	y	y	y	y	y	8
20	Johansen	y	y	y	y	y	n	y	y	y	8
21	Ahmed	y	n	y	y	y	n	y	y	y	7
22	Bogdanov	y	n	y	y	y	n	y	y	y	7
23	Calvani	y	n	y	y	y	y	y	y	y	8
24	Cirstea	y	y	y	y	y	y	y	y	y	9
25	Picca	y	n	y	y	y	y	y	y	y	8
26	Nagesh Babu	y	n	y	y	y	y	y	y	y	8
27	Han	y	y	y	y	y	n	y	y	y	8
28	Burté	y	n	n	y	y	n	y	y	y	6
29	Roede	y	n	y	y	y	y	y	y	y	8
30	Schulte	y	y	y	n	y	n	y	y	y	7
31	Crotty	y	y	y	y	y	y	y	y	y	9
32	Stoessel	y	n	n	y	y	y	y	y	y	7
33	Havelund	y	n	y	y	y	y	y	y	y	8
34	Wuolikainen	y	n	y	y	y	n	y	y	y	7
35	Trupp	y	n	y	y	y	y	y	y	y	8
36	Bolner	y	n	n	y	y	n	y	y	y	6
37	Abe	y	n	n	y	y	n	y	y	y	6
38	Michell	y	n	y	y	y	y	y	y	y	8
39	Troisi	y	n	y	n	y	y	y	y	y	7
40	Yilmaz	y	n	y	y	y	n	y	y	y	7
41	Lucio	y	n	y	y	y	n	y	y	y	7
42	Willkommen	y	n	y	y	y	n	y	y	y	7
43	Trezzi	y	n	y	y	y	y	y	y	y	8

44	Öhman	y	n	y	y	n	n	y	y	y	6
45	Lewitt	y	n	y	y	y	n	y	y	y	7
46	LeWitt	y	y	y	y	y	y	y	y	y	9
47	Tan	y	n	y	y	y	y	y	y	y	8
48	Vascellari	y	n	y	y	y	n	y	y	y	7
49	Kumari	y	n	y	y	y	y	y	y	y	8
50	Luan	y	n	y	y	y	y	y	y	y	8
51	Luan	y	n	y	y	y	n	y	y	y	7
52	Luan	y	n	y	y	y	y	y	y	y	8
53	Sinclair	y	y	y	n	y	y	y	y	y	8
54	Trivedi	y	y	n	n	y	n	y	y	y	6
55	Kumari	y	n	y	y	y	y	y	y	y	8
56	Wood	y	n	y	y	y	n	y	y	y	7
57	Griffith	y	n	y	y	y	n	y	y	y	7
58	D'Andrea	y	n	n	y	y	y	y	y	y	7
59	Hamid	y	y	y	y	y	y	y	y	y	9
60	Takeshige	y	y	y	y	y	y	y	y	y	9
61	Kumari	y	n	n	y	y	y	y	y	y	7
62	Ohmich	y	n	y	y	y	y	y	y	y	8
63	Fujimaki	y	n	y	y	n	y	y	y	y	7
64	Li	y	n	y	y	y	y	y	y	y	8
65	Fernández-Iri goyen	y	n	y	y	y	y	y	y	y	8
66	Silvia	y	n	y	y	y	y	y	y	y	8
67	Aaron	y	n	y	n	y	y	y	y	y	7
68	Chang	y	n	y	y	y	y	y	y	y	8
69	Chen	y	n	y	y	y	y	y	y	y	8
70	Eduardo	y	n	y	y	y	y	y	y	y	8
71	Dong	y	n	y	y	y	n	y	y	y	7
72	Carolina	y	y	y	y	y	y	y	y	y	9
73	Klatt	y	n	y	y	y	n	y	y	y	7
74	Meoni	y	n	y	y	y	y	y	y	y	8
75	Pathan	y	n	y	y	y	n	y	y	y	7

76	Plewa	y	n	y	y	y	n	y	y	y	7
77	Dorines	y	n	y	y	y	n	y	y	y	7
78	Scholefield	y	n	y	y	y	y	y	y	y	8
79	Scholefield	y	n	y	y	y	y	y	y	n	7
80	Yan	y	n	y	y	y	y	y	y	y	8
81	Zhang	y	n	n	y	y	y	y	y	y	7
82	Chan	y	n	y	y	y	y	y	y	y	8
83	Thomas	y	n	y	y	y	y	y	y	y	8
84	Anastasia	y	y	y	y	y	y	y	y	y	9

Supplementary Table 3: All 928 diagnosis-related metabolites.(Note: the ID was represented by VMH ID or Pubchem ID or name)

ID	inchistring	kingdom	superclass	class	subclass	level5	level6	level7	level8	level9	crossmatchWithRecon3D	crossmatchWithiDNCT	VMH	increased	decreased	increased_highfreq	decreased_highfreq	increased_Realfrequency	decreased_Realfrequency	inconsistent	BBBcross	BBBnoncross
i	InChI=1S/HI/h1H/p-1	Inorganic compounds	Homogeneous non-metal compounds	Homogeneous halogens							TRUE	FALSE	TRUE	TRUE	FALSE	FALSE	FALSE	1	0	0	FALSE	FALSE
pi	InChI=1S/H3O4P/c1-5(2,3)4/h(H3,1,2,3,4)/p-2	Inorganic compounds	Homogeneous non-metal compounds	Non-metal oxoanionic compounds	Non-metal phosphates						TRUE	TRUE	TRUE	TRUE	FALSE	FALSE	FALSE	1	0	0	FALSE	FALSE
4873	InChI=1S/ClH.K/h1H;/q;+1/p-1	Inorganic compounds	Mixed metal/non-metal compounds	Alkali metal salts	Alkali metal chlorides						FALSE	FALSE	FALSE	TRUE	FALSE	FALSE	FALSE	1	0	0	FALSE	FALSE
5570	InChI=1S/C7H7NO2/c1-8-4-2-3-6(5-8)7(9)10/h2-5H,1H3	Organic compounds	Alkaloids and derivatives								FALSE	FALSE	FALSE	FALSE	TRUE	FALSE	TRUE	0	4	0	FALSE	FALSE
439616	InChI=1S/C8H8O3/c9-7(8(10)11)6-4-2-1-3-5-6/h1-5,7,9H,(H,10,11)/t7-/m0/s1	Organic compounds	Benzenoids	Benzene and substituted derivatives							FALSE	FALSE	FALSE	FALSE	TRUE	FALSE	FALSE	0	1	0	FALSE	FALSE
4775	InChI=1S/C10H12O2/c11-10(12)8-4-7-9-5-2-1-3-6-9/h1-3,5-6H,4,7-8H2,(H,11,12)	Organic compounds	Benzenoids	Benzene and substituted derivatives							FALSE	FALSE	FALSE	TRUE	FALSE	FALSE	FALSE	1	0	0	FALSE	FALSE
pac	InChI=1S/C8H8O2/c9-8(10)6-7-4-2-1-3-5-7/h1-5H,6H2,(H,9,10)/p-1	Organic compounds	Benzenoids	Benzene and substituted derivatives							TRUE	TRUE	TRUE	TRUE	FALSE	TRUE	FALSE	2	0	0	FALSE	FALSE
403	InChI=1S/C6H7NO/c7-5-1-3-6(8)4-2-5/h1-4,8H,7H2	Organic compounds	Benzenoids	Benzene and substituted derivatives	Aniline and substituted anilines						FALSE	FALSE	FALSE	TRUE	FALSE	FALSE	FALSE	1	0	0	FALSE	FALSE
anth	InChI=1S/C7H7NO2/c8-6-4-2-1-3-5(6)7(9)10/h1-4H,8H2,(H,9,10)/p-1	Organic compounds	Benzenoids	Benzene and substituted derivatives	Benzoic acids and derivatives	Aminobenzoic acids and derivatives		Aminobenzoic acids			TRUE	FALSE	TRUE	FALSE	TRUE	FALSE	FALSE	0	1	0	FALSE	FALSE
978	InChI=1S/C7H7NO2/c8-6-3-1-5(2-4-6)7(9)10/h1-4H,8H2,(H,9,10)	Organic compounds	Benzenoids	Benzene and substituted derivatives	Benzoic acids and derivatives	Aminobenzoic acids and derivatives	Aminobenzoic acids				FALSE	FALSE	FALSE	TRUE	FALSE	FALSE	FALSE	1	0	0	FALSE	FALSE
2148	InChI=1S/C9H10N2O3/c10-7-3-1-6(2-4-7)9(14)11-5-8(12)13/h1-4H,5,10H2,(H,11,14)(H,12,13)	Organic compounds	Benzenoids	Benzene and substituted derivatives	Benzoic acids and derivatives	Benzamides	Hippuric acids and derivatives	Hippuric acids			FALSE	FALSE	FALSE	TRUE	FALSE	FALSE	FALSE	1	0	0	FALSE	FALSE
450268	InChI=1S/C9H9NO4/c11-7-3-1-2-6(4-7)9(14)10-5-8(12)13/h1-4,11H,5H2,(H,10,14)(H,12,13)	Organic compounds	Benzenoids	Benzene and substituted derivatives	Benzoic acids and derivatives	Benzamides	Hippuric acids and derivatives	Hippuric acids			FALSE	FALSE	FALSE	FALSE	TRUE	FALSE	FALSE	0	1	0	FALSE	FALSE
bgly	InChI=1S/C9H9NO3/c11-8(12)6-10-9(13)7-4-2-1-3-5-7/h1-5H,6H2,(H,10,13)(H,11,12)/p-1	Organic compounds	Benzenoids	Benzene and substituted derivatives	Benzoic acids and derivatives	Benzamides	Hippuric acids and derivatives	Hippuric acids			TRUE	FALSE	TRUE	FALSE	TRUE	FALSE	FALSE	0	1	0	FALSE	FALSE
61156	InChI=1S/C24H20O6/c25-22(18-10-4-1-5-11-18)28-16-21(30-24(27)20-14-8-3-9-15-20)17-29-23(26)19-12-6-2-7-13-19/h1-15,21H,16-17H2	Organic compounds	Benzenoids	Benzene and substituted derivatives	Benzoic acids and derivatives	Benzoic acid esters					FALSE	FALSE	FALSE	TRUE	FALSE	FALSE	FALSE	1	0	0	FALSE	FALSE
349497	InChI=1S/C7H7NO3/c9-7(10)5-3-1-2-4-6(5)8-11/h1-4,8,11H,(H,9,10)	Organic compounds	Benzenoids	Benzene and substituted derivatives	Benzoic acids and derivatives	Benzoic acids					FALSE	FALSE	FALSE	TRUE	FALSE	TRUE	FALSE	2	0	0	FALSE	FALSE
4hbz	InChI=1S/C7H6O3/c8-6-3-1-5(2-4-6)7(9)10/h1-4,8H,(H,9,10)/p-1	Organic compounds	Benzenoids	Benzene and substituted derivatives	Benzoic acids and derivatives	Benzoic acids					TRUE	FALSE	TRUE	TRUE	TRUE	FALSE	FALSE	1	1	1	FALSE	FALSE
bz	InChI=1S/C7H6O2/c8-7(9)6-4-2-1-3-5-6/h1-5H,(H,8,9)/p-1	Organic compounds	Benzenoids	Benzene and substituted derivatives	Benzoic acids and derivatives	Benzoic acids					TRUE	FALSE	TRUE	TRUE	FALSE	FALSE	FALSE	1	0	0	FALSE	FALSE
3hanthrn	InChI=1S/C7H7NO3/c8-6-4(7(10)11)2-1-3-5(6)9/h1-3,9H,8H2,(H,10,11)	Organic compounds	Benzenoids	Benzene and substituted derivatives	Benzoic acids and derivatives	Hydroxybenzoic acid derivatives					TRUE	FALSE	TRUE	TRUE	FALSE	FALSE	FALSE	1	0	0	FALSE	FALSE
8468	InChI=1S/C8H8O4/c1-12-7-4-5(8(10)11)2-3-6(7)9/h2-4,9H,1H3,(H,10,11)	Organic compounds	Benzenoids	Benzene and substituted derivatives	Benzoic acids and derivatives	Methoxybenzoic acids and derivatives	M-methoxybenzoic acids and derivatives				FALSE	FALSE	FALSE	TRUE	FALSE	FALSE	FALSE	1	0	0	FALSE	FALSE

10703	InChI=1S/C10H14/c1-8(2)10-7-5-4-6-9(10)3/h4-8H,1-3H3	Organic compounds	Benzenoids	Benzene and substituted derivatives	Cumenes		FALSE	FALSE	FALSE	TRUE	FALSE	FALSE	FALSE	1	0	0	FALSE	FALSE
36159	InChI=1S/C12H5Br5O/c13-6-1-2-11(9(16)3-6)18-12-5-8(15)7(14)4-10(12)17/h1-5H	Organic compounds	Benzenoids	Benzene and substituted derivatives	Diphenylethers	Bromodiphenyl ethers	FALSE	FALSE	FALSE	TRUE	FALSE	FALSE	FALSE	1	0	0	FALSE	FALSE
ebastine	InChI=1S/C32H39NO2/c1-32(2,3)28-18-16-25(17-19-28)30(34)15-10-22-33-23-20-29(21-24-33)35-31(26-11-6-4-7-12-26)27-13-8-5-9-14-27/h4-9,11-14,16-19,29,31H,10,15,20-24H2,1-3H3	Organic compounds	Benzenoids	Benzene and substituted derivatives	Diphenylmethanes		TRUE	FALSE	TRUE	TRUE	FALSE	FALSE	FALSE	1	0	0	FALSE	FALSE
peamn	InChI=1S/C8H11N/c9-7-6-8-4-2-1-3-5-8/h1-5H,6-7,9H2/p+1	Organic compounds	Benzenoids	Benzene and substituted derivatives	Phenethylamines		TRUE	TRUE	TRUE	TRUE	TRUE	FALSE	FALSE	1	1	1	FALSE	FALSE
tym	InChI=1S/C8H11NO/c9-6-5-7-1-3-8(10)4-2-7/h1-4,10H,5-6,9H2/p+1	Organic compounds	Benzenoids	Benzene and substituted derivatives	Phenethylamines		TRUE	TRUE	TRUE	TRUE	FALSE	TRUE	FALSE	2	0	0	FALSE	FALSE
pacald	InChI=1S/C8H8O/c9-7-6-8-4-2-1-3-5-8/h1-5,7H,6H2	Organic compounds	Benzenoids	Benzene and substituted derivatives	Phenylacetaldehydes		TRUE	TRUE	TRUE	FALSE	TRUE	FALSE	FALSE	0	1	0	FALSE	FALSE
136810		Organic compounds	Benzenoids	Benzene and substituted derivatives	Phenylpropanes		FALSE	FALSE	FALSE	TRUE	FALSE	FALSE	FALSE	1	0	0	FALSE	FALSE
162265	InChI=1S/C9H13NO/c1-7(10)9(11)8-5-3-2-4-6-8/h2-7,9,11H,10H2,1H3/t7-,9+/m1/s1	Organic compounds	Benzenoids	Benzene and substituted derivatives	Phenylpropanes		FALSE	FALSE	FALSE	TRUE	FALSE	FALSE	FALSE	1	0	0	FALSE	FALSE
2762430	InChI=1S/C9H10O3/c1-6(10)4-7-2-3-8(11)9(12)5-7/h2-3,5,11-12H,4H2,1H3	Organic compounds	Benzenoids	Benzene and substituted derivatives	Phenylpropanes		FALSE	FALSE	FALSE	TRUE	FALSE	FALSE	FALSE	1	0	0	FALSE	FALSE
21124401	InChI=1S/C9H8O5/c10-6-2-1-5(3-7(6)11)4-8(12)9(13)14/h1-3,10-11H,4H2,(H,13,14)/p-1	Organic compounds	Benzenoids	Benzene and substituted derivatives	Phenylpyruvic acid derivatives		FALSE	FALSE	FALSE	FALSE	TRUE	FALSE	FALSE	0	1	0	FALSE	FALSE
62453	InChI=1S/C8H8O/c1-2-7-3-5-8(9)6-4-7/h2-6,9H,1H2	Organic compounds	Benzenoids	Benzene and substituted derivatives	Styrenes		FALSE	FALSE	FALSE	FALSE	TRUE	FALSE	FALSE	0	1	0	FALSE	FALSE
pcresol	InChI=1S/C7H8O/c1-6-2-4-7(8)5-3-6/h2-5,8H,1H3/p-1	Organic compounds	Benzenoids	Benzene and substituted derivatives	Toluenes		TRUE	FALSE	TRUE	TRUE	FALSE	TRUE	FALSE	2	0	0	FALSE	FALSE
Isovanillymandelic acid		Organic compounds	Benzenoids	Imidazopyrimidines	Purines and purine derivatives		FALSE	FALSE	FALSE	TRUE	FALSE	FALSE	FALSE	1	0	0	FALSE	FALSE
66886955	InChI=1S/C9H8O5/c10-6-1-3-7(4-2-6)14-9(13)5-8(11)12/h1-4,10H,5H2,(H,11,12)	Organic compounds	Benzenoids	Phenol esters			FALSE	FALSE	FALSE	TRUE	FALSE	FALSE	FALSE	1	0	0	FALSE	FALSE
4631	InChI=1S/C15H23NO3/c1-4-9-18-14-7-5-6-8-15(14)19-11-13(17)10-16-12(2)3/h4-8,12-13,16-17H,1,9-11H2,2-3H3	Organic compounds	Benzenoids	Phenol ethers			FALSE	FALSE	FALSE	FALSE	TRUE	FALSE	FALSE	0	1	0	FALSE	FALSE
4hphac	InChI=1S/C8H8O3/c9-7-3-1-6(2-4-7)5-8(10)11/h1-4,9H,5H2,(H,10,11)/p-1	Organic compounds	Benzenoids	Phenols	1-hydroxy-2-unsubstituted benzenoids		TRUE	FALSE	TRUE	TRUE	TRUE	TRUE	FALSE	5	1	1	FALSE	FALSE
phenol	InChI=1S/C6H6O/c7-6-4-2-1-3-5-6/h1-5,7H	Organic compounds	Benzenoids	Phenols	1-hydroxy-4-unsubstituted benzenoids		FALSE	FALSE	TRUE	FALSE	TRUE	FALSE	FALSE	0	1	0	FALSE	FALSE
34dhpha	InChI=1S/C8H8O4/c9-6-2-1-5(3-7(6)10)4-8(11)12/h1-3,9-10H,4H2,(H,11,12)/p-1	Organic compounds	Benzenoids	Phenols	Benzenediols	Catechols	TRUE	TRUE	TRUE	FALSE	TRUE	FALSE	TRUE	0	2	0	FALSE	FALSE
adrnl	InChI=1S/C9H13NO3/c1-10-5-9(13)6-2-3-7(11)8(12)4-6/h2-4,9-13H,5H2,1H3/p+1/t9-/m0/s1	Organic compounds	Benzenoids	Phenols	Benzenediols	Catechols	TRUE	TRUE	TRUE	FALSE	TRUE	FALSE	FALSE	0	1	0	FALSE	FALSE

nrpphr	InChI=1S/C8H11NO3/c9-4-8(12)5-1-2-6(10)7(11)3-5/h1-3,8,10-12H,4,9H2/p+1/t8-/m0/s1	Organic compounds	Benzenoids	Phenols	Benzenediols	Catechols		TRUE	TRUE	TRUE	FALSE	TRUE	FALSE	TRUE	0	2	0	FALSE	FALSE
dopa	InChI=1S/C8H11NO2/c9-4-3-6-1-2-7(10)8(11)5-6/h1-2,5,10-11H,3-4,9H2/p+1	Organic compounds	Benzenoids	Phenols	Benzenediols	Catechols	Catechol amines and derivatives	TRUE	TRUE	TRUE	TRUE	TRUE	TRUE	TRUE	2	2	1	FALSE	FALSE
3610	InChI=1S/C12H18O2/c1-2-3-4-5-6-10-7-8-11(13)9-12(10)14/h7-9,13-14H,2-6H2,1H3	Organic compounds	Benzenoids	Phenols	Benzenediols	Resorcinols		FALSE	FALSE	FALSE	FALSE	TRUE	FALSE	FALSE	0	1	0	FALSE	FALSE
335	InChI=1S/C7H8O/c1-6-4-2-3-5-7(6)8/h2-5,8H,1H3	Organic compounds	Benzenoids	Phenols	Cresols	Ortho cresols		FALSE	FALSE	FALSE	FALSE	TRUE	FALSE	FALSE	0	1	0	FALSE	FALSE
1183	InChI=1S/C8H8O3/c1-11-8-4-6(5-9)2-3-7(8)10/h2-5,10H,1H3	Organic compounds	Benzenoids	Phenols	Methoxyphenols			FALSE	FALSE	FALSE	TRUE	FALSE	FALSE	FALSE	1	0	0	FALSE	FALSE
1548943	InChI=1S/C18H27NO3/c1-14(2)8-6-4-5-7-9-18(21)19-13-15-10-11-16(20)17(12-15)22-3/h6,8,10-12,14,20H,4-5,7,9,13H2,1-3H3,(H,19,21)/b8-6+	Organic compounds	Benzenoids	Phenols	Methoxyphenols			FALSE	FALSE	FALSE	TRUE	FALSE	FALSE	FALSE	1	0	0	FALSE	FALSE
3mox4hoxm	InChI=1S/C9H10O5/c1-14-7-4-5(2-3-6(7)10)8(11)9(12)13/h2-4,8,10-11H,1H3,(H,12,13)/p-1	Organic compounds	Benzenoids	Phenols	Methoxyphenols			TRUE	TRUE	TRUE	TRUE	FALSE	TRUE	FALSE	2	0	0	FALSE	FALSE
homoval	InChI=1S/C9H10O4/c1-13-8-4-6(5-9(11)12)2-3-7(8)10/h2-4,10H,5H2,1H3,(H,11,12)/p-1	Organic compounds	Benzenoids	Phenols	Methoxyphenols			TRUE	FALSE	TRUE	TRUE	TRUE	TRUE	TRUE	3	2	1	FALSE	FALSE
mepi	InChI=1S/C10H15NO3/c1-11-6-9(13)7-3-4-8(12)10(5-7)14-2/h3-5,9,11-13H,6H2,1-2H3/p+1/t9-/m0/s1	Organic compounds	Benzenoids	Phenols	Methoxyphenols			TRUE	FALSE	TRUE	TRUE	FALSE	FALSE	FALSE	1	0	0	FALSE	FALSE
19933739	InChI=1S/C14H16O2/c1-10(15)2-3-11-4-5-13-9-14(16)7-6-12(13)8-11/h4-10,15-16H,2-3H2,1H3	Organic compounds	Benzenoids	phthalenes	phthols and derivatives			FALSE	FALSE	FALSE	FALSE	TRUE	FALSE	FALSE	0	1	0	FALSE	FALSE
M00538	InChI=1S/C10H8O/c11-10-7-3-5-8-4-1-2-6-9(8)10/h1-7,11H	Organic compounds	Benzenoids	phthalenes	phthols and derivatives			TRUE	FALSE	TRUE	TRUE	FALSE	FALSE	FALSE	1	0	0	FALSE	FALSE
356	InChI=1S/C8H18/c1-3-5-7-8-6-4-2/h3-8H2,1-2H3	Organic compounds	Hydrocarbons	Saturated hydrocarbons	Alkanes			FALSE	FALSE	FALSE	FALSE	TRUE	FALSE	FALSE	0	1	0	FALSE	FALSE
7461	InChI=1S/C10H16/c1-8(2)10-6-4-9(3)5-7-10/h4,7-8H,5-6H2,1-3H3	Organic compounds	Hydrocarbons	Unsaturated hydrocarbons	Branched unsaturated hydrocarbons			FALSE	FALSE	FALSE	TRUE	FALSE	FALSE	FALSE	1	0	0	FALSE	FALSE
74138	InChI=1S/C22H44/c1-3-5-7-9-11-13-15-17-19-21-22-20-18-16-14-12-10-8-6-4-2/h3H,1,4-22H2,2H3	Organic compounds	Hydrocarbons	Unsaturated hydrocarbons	Unsaturated aliphatic hydrocarbons			FALSE	FALSE	FALSE	FALSE	TRUE	FALSE	FALSE	0	1	0	FALSE	FALSE
FFA 16:1		Organic compounds	Lipids and lipid-like molecules	Fatty Acyls				FALSE	FALSE	FALSE	TRUE	FALSE	FALSE	FALSE	1	0	0	FALSE	FALSE
16061120	InChI=1S/C20H32O4/c1-2-18(21)19(22)16-14-12-10-8-6-4-3-5-7-9-11-13-15-17-20(23)24/h3,5-6,8-9,11-12,14,18-19,21-22H,2,4,7,10,13,15-17H2,1H3,(H,23,24)/b5-3-,8-6-,11-9-,14-12-	Organic compounds	Lipids and lipid-like molecules	Fatty Acyls	Eicosanoids	Hydroxyeicosatrienoic acids		FALSE	FALSE	FALSE	FALSE	TRUE	FALSE	FALSE	0	1	0	FALSE	FALSE
5283142	InChI=1S/C20H34O4/c1-2-3-4-5-6-7-8-9-10-11-12-13-15-18(21)19(22)16-14-17-20(23)24/h6-7,9-10,12-13,18-19,21-22H,2-5,8,11,14-17H2,1H3,(H,23,24)/b7-6-,10-9-,13-12-	Organic compounds	Lipids and lipid-like molecules	Fatty Acyls	Eicosanoids	Hydroxyeicosatrienoic acids		FALSE	FALSE	FALSE	FALSE	TRUE	FALSE	FALSE	0	1	0	FALSE	FALSE

5283146	InChI=1S/C20H34O4/c1-2-3-4-5-9-12-15-18(21)19(22)16-13-10-7-6-8-11-14-17-20(23)24/h6,8-10,12-13,18-19,21-22H,2-5,7,11,14-17H2,1H3,(H,23,24)/b8-6-,12-9-,13-10-	Organic compounds	Lipids and lipid-like molecules	Fatty Acyls	Eicosanoids	Hydroxyeicosatrienoic acids	FALSE	FALSE	FALSE	FALSE	TRUE	FALSE	FALSE	0	1	0	FALSE	FALSE
5283147	InChI=1S/C20H34O4/c1-2-3-12-15-18(21)19(22)16-13-10-8-6-4-5-7-9-11-14-17-20(23)24/h4,6-7,9-10,13,18-19,21-22H,2-3,5,8,11-12,14-17H2,1H3,(H,23,24)/b6-4-,9-7-,13-10-	Organic compounds	Lipids and lipid-like molecules	Fatty Acyls	Eicosanoids	Hydroxyeicosatrienoic acids	FALSE	FALSE	FALSE	FALSE	TRUE	FALSE	FALSE	0	1	0	FALSE	FALSE
5353272	InChI=1S/C20H32O3/c1-2-3-4-5-10-13-16-19(21)17-14-11-8-6-7-9-12-15-18-20(22)23/h7-11,13-14,17,19,21H,2-6,12,15-16,18H2,1H3,(H,22,23)/b9-7+,11-8+,13-10+,17-14+	Organic compounds	Lipids and lipid-like molecules	Fatty Acyls	Eicosanoids	Hydroxyeicosatrienoic acids	FALSE	FALSE	FALSE	FALSE	TRUE	FALSE	FALSE	0	1	0	FALSE	FALSE
6439476	InChI=1S/C20H34O4/c1-2-3-4-5-6-9-13-18(21)14-10-7-8-11-15-19(22)16-12-17-20(23)24/h7-8,10-11,14-15,18-19,21-22H,2-6,9,12-13,16-17H2,1H3,(H,23,24)/b8-7-,14-10+,15-11+/t18-,19-/m0/s1	Organic compounds	Lipids and lipid-like molecules	Fatty Acyls	Eicosanoids	Hydroxyeicosatrienoic acids	FALSE	FALSE	FALSE	TRUE	FALSE	FALSE	FALSE	1	0	0	FALSE	FALSE
5283107	InChI=1S/C26H38O5/c1-19(2)31-26(30)13-9-4-3-8-12-22-23(25(29)18-24(22)28)17-16-21(27)15-14-20-10-6-5-7-11-20/h3,5-8,10-11,16-17,19,21-25,27-29H,4,9,12-15,18H2,1-2H3/b8-3-,17-16+/t21-,22+,23+,24-,25+/m0/s1	Organic compounds	Lipids and lipid-like molecules	Fatty Acyls	Eicosanoids	Prostaglandins and related compounds	FALSE	FALSE	FALSE	TRUE	FALSE	FALSE	FALSE	1	0	0	FALSE	FALSE
9831415	InChI=1S/C22H34O2/c1-3-5-6-7-8-9-10-11-12-13-14-15-16-17-18-19-20-21-22(23)24-4-2/h5-6,8-9,11-12,14-15,17-18H,3-4,7,10,13,16,19-21H2,1-2H3/b6-5-,9-8-,12-11-,15-14-,18-17-	Organic compounds	Lipids and lipid-like molecules	Fatty Acyls	Fatty acid esters		FALSE	FALSE	FALSE	FALSE	TRUE	FALSE	FALSE	0	1	0	FALSE	FALSE
lnlncacrn	InChI=1S/C25H43NO4/c1-5-6-7-8-9-10-11-12-13-14-15-16-17-18-19-20-25(29)30-23(26(2,3)4)21-22-24(27)28/h6-7,9-10,12-13,23H,5,8,11,14-22H2,1-4H3/b7-6-,10-9-,13-12-/t23-/m0/s1	Organic compounds	Lipids and lipid-like molecules	Fatty Acyls	Fatty acid esters		TRUE	TRUE	TRUE	TRUE	FALSE	FALSE	FALSE	1	0	0	FALSE	FALSE
2-Hydroxyhexadecanoylcarnitine		Organic compounds	Lipids and lipid-like molecules	Fatty Acyls	Fatty acid esters	Acyl carnitines	FALSE	FALSE	FALSE	TRUE	TRUE	FALSE	FALSE	1	1	1	FALSE	FALSE
3ivcrn	InChI=1S/C12H23NO5/c1-12(2,17)7-11(16)18-9(6-10(14)15)8-13(3,4)5/h9,17H,6-8H2,1-5H3	Organic compounds	Lipids and lipid-like molecules	Fatty Acyls	Fatty acid esters	Acyl carnitines	TRUE	FALSE	TRUE	TRUE	FALSE	FALSE	FALSE	1	0	0	FALSE	FALSE
53481624	InChI=1S/C13H25NO5/c1-5-6-10(15)7-13(18)19-11(8-12(16)17)9-14(2,3)4/h10-11,15H,5-9H2,1-4H3/t10?,11-/m0/s1	Organic compounds	Lipids and lipid-like molecules	Fatty Acyls	Fatty acid esters	Acyl carnitines	FALSE	FALSE	FALSE	TRUE	FALSE	FALSE	FALSE	1	0	0	FALSE	FALSE
6426901	InChI=1S/C12H23NO4/c1-6-9(2)12(16)17-10(7-11(14)15)8-13(3,4)5/h9-10H,6-8	Organic compounds	Lipids and lipid-like molecules	Fatty Acyls	Fatty acid esters	Acyl carnitines	FALSE	FALSE	FALSE	FALSE	TRUE	FALSE	FALSE	0	1	0	FALSE	FALSE

	H2,1-5H3																	
71317118	InChI=1S/C12H21NO6/c1-13(2,3)8-9(7-11(16)17)19-12(18)6-4-5-10(14)15/h9H,4-8H2,1-3H3,(H-,14,15,16,17)/t9-/m1/s1	Organic compounds	Lipids and lipid-like molecules	Fatty Acyls	Fatty acid esters	Acyl carnitines	FALSE	FALSE	FALSE	TRUE	FALSE	FALSE	FALSE	1	0	0	FALSE	FALSE
71464474	InChI=1S/C12H23NO5/c1-12(2,17)7-11(16)18-9(6-10(14)15)8-13(3,4)5/h9,17H,6-8H2,1-5H3	Organic compounds	Lipids and lipid-like molecules	Fatty Acyls	Fatty acid esters	Acyl carnitines	FALSE	FALSE	FALSE	FALSE	TRUE	FALSE	FALSE	0	1	0	FALSE	FALSE
AC(12:0)		Organic compounds	Lipids and lipid-like molecules	Fatty Acyls	Fatty acid esters	Acyl carnitines	FALSE	FALSE	FALSE	FALSE	TRUE	FALSE	FALSE	0	1	0	FALSE	FALSE
AC(12:1)		Organic compounds	Lipids and lipid-like molecules	Fatty Acyls	Fatty acid esters	Acyl carnitines	FALSE	FALSE	FALSE	FALSE	TRUE	FALSE	FALSE	0	1	0	FALSE	FALSE
AC(14:0)		Organic compounds	Lipids and lipid-like molecules	Fatty Acyls	Fatty acid esters	Acyl carnitines	FALSE	FALSE	FALSE	FALSE	TRUE	FALSE	FALSE	0	1	0	FALSE	FALSE
AC(14:1)		Organic compounds	Lipids and lipid-like molecules	Fatty Acyls	Fatty acid esters	Acyl carnitines	FALSE	FALSE	FALSE	FALSE	TRUE	FALSE	FALSE	0	1	0	FALSE	FALSE
AC(14:2)		Organic compounds	Lipids and lipid-like molecules	Fatty Acyls	Fatty acid esters	Acyl carnitines	FALSE	FALSE	FALSE	FALSE	TRUE	FALSE	FALSE	0	1	0	FALSE	FALSE
AC(16:0)		Organic compounds	Lipids and lipid-like molecules	Fatty Acyls	Fatty acid esters	Acyl carnitines	FALSE	FALSE	FALSE	FALSE	TRUE	FALSE	FALSE	0	1	0	FALSE	FALSE
AC(16:1)		Organic compounds	Lipids and lipid-like molecules	Fatty Acyls	Fatty acid esters	Acyl carnitines	FALSE	FALSE	FALSE	FALSE	TRUE	FALSE	FALSE	0	1	0	FALSE	FALSE
C18:2-carnitine		Organic compounds	Lipids and lipid-like molecules	Fatty Acyls	Fatty acid esters	Acyl carnitines	FALSE	FALSE	FALSE	TRUE	FALSE	FALSE	FALSE	1	0	0	FALSE	FALSE
C8:0-carnitine		Organic compounds	Lipids and lipid-like molecules	Fatty Acyls	Fatty acid esters	Acyl carnitines	FALSE	FALSE	FALSE	TRUE	FALSE	FALSE	FALSE	1	0	0	FALSE	FALSE
HC10859	InChI=1S/C10H17NO6/c1-11(2,3)6-7(4-8(12)13)17-10(16)5-9(14)15/h7H,4-6H2,1-3H3,(H-,12,13,14,15)/t7-/m1/s1	Organic compounds	Lipids and lipid-like molecules	Fatty Acyls	Fatty acid esters	Acyl carnitines	TRUE	TRUE	TRUE	TRUE	FALSE	TRUE	FALSE	2	0	0	FALSE	FALSE
c4dc	InChI=1S/C11H19NO6/c1-12(2,3)7-8(6-10(15)16)18-11(17)5-4-9(13)14/h8H,4-7H2,1-3H3,(H-,13,14,15,16)	Organic compounds	Lipids and lipid-like molecules	Fatty Acyls	Fatty acid esters	Acyl carnitines	TRUE	FALSE	TRUE	FALSE	TRUE	FALSE	FALSE	0	1	0	FALSE	FALSE
c6dc	InChI=1S/C13H23NO6/c1-14(2,3)9-10(8-12(17)18)20-13(19)7-5-4-6-11(15)16/h10H,4-9H2,1-3H3,(H-,15,16,17,18)/p-1	Organic compounds	Lipids and lipid-like molecules	Fatty Acyls	Fatty acid esters	Acyl carnitines	TRUE	FALSE	TRUE	FALSE	TRUE	FALSE	FALSE	0	1	0	FALSE	FALSE
carnitine 10:1		Organic compounds	Lipids and lipid-like molecules	Fatty Acyls	Fatty acid esters	Acyl carnitines	FALSE	FALSE	FALSE	FALSE	TRUE	FALSE	FALSE	0	1	0	FALSE	FALSE
carnitine 12:0		Organic compounds	Lipids and lipid-like molecules	Fatty Acyls	Fatty acid esters	Acyl carnitines	FALSE	FALSE	FALSE	FALSE	TRUE	FALSE	FALSE	0	1	0	FALSE	FALSE
carnitine 12:1		Organic compounds	Lipids and lipid-like molecules	Fatty Acyls	Fatty acid esters	Acyl carnitines	FALSE	FALSE	FALSE	FALSE	TRUE	FALSE	FALSE	0	1	0	FALSE	FALSE
carnitine 14:2		Organic compounds	Lipids and lipid-like molecules	Fatty Acyls	Fatty acid esters	Acyl carnitines	FALSE	FALSE	FALSE	FALSE	TRUE	FALSE	FALSE	0	1	0	FALSE	FALSE
carnitine 14:3		Organic compounds	Lipids and lipid-like molecules	Fatty Acyls	Fatty acid esters	Acyl carnitines	FALSE	FALSE	FALSE	FALSE	TRUE	FALSE	FALSE	0	1	0	FALSE	FALSE
elaidcrn	InChI=1S/C25H47NO4/c1-5-6-7-8-9-10-11-12-13-14-15-16-17-18-19-20-25(29)30-23(21-24(27)28)22-26(2,3)4/h12-13,23H,5-11,14-22H2,1-4H3/b13-12+	Organic compounds	Lipids and lipid-like molecules	Fatty Acyls	Fatty acid esters	Acyl carnitines	TRUE	TRUE	TRUE	TRUE	FALSE	FALSE	FALSE	1	0	0	FALSE	FALSE
lneldccrn	InChI=1S/C25H45NO4/c1-5-6-7-8-9-10-11-12-13-14-15-16-17-18-19-20-25(29)30-23(21-24(27)28)22-26(2,3)4/h9-10,1	Organic compounds	Lipids and lipid-like molecules	Fatty Acyls	Fatty acid esters	Acyl carnitines	TRUE	TRUE	TRUE	TRUE	FALSE	FALSE	FALSE	1	0	0	FALSE	FALSE

	2-13,23H,5-8,11,14-22H2,1-4H3/b10-9+,13-12+																		
lnlccrn	InChI=1S/C25H45NO4/c1-5-6-7-8-9-10-11-12-13-14-15-16-17-18-19-20-25(29)30-23(21-24(27)28)22-26(2,3)4/h9-10,12-13,23H,5-8,11,14-22H2,1-4H3/b10-9-,13-12-/t23-/m1/s1	Organic compounds	Lipids and lipid-like molecules	Fatty Acyls	Fatty acid esters	Acyl carnitines		TRUE	TRUE	TRUE	FALSE	TRUE	FALSE	FALSE	0	1	0	FALSE	FALSE
odecrn	InChI=1S/C25H47NO4/c1-5-6-7-8-9-10-11-12-13-14-15-16-17-18-19-20-25(29)30-23(21-24(27)28)22-26(2,3)4/h12-13,23H,5-11,14-22H2,1-4H3/t23-/m0/s1	Organic compounds	Lipids and lipid-like molecules	Fatty Acyls	Fatty acid esters	Acyl carnitines		TRUE	TRUE	TRUE	TRUE	FALSE	FALSE	FALSE	1	0	0	FALSE	FALSE
pcrn	InChI=1S/C10H19NO4/c1-5-10(14)15-8(6-9(12)13)7-11(2,3)4/h8H,5-7H2,1-4H3	Organic compounds	Lipids and lipid-like molecules	Fatty Acyls	Fatty acid esters	Acyl carnitines		TRUE	TRUE	TRUE	TRUE	FALSE	FALSE	FALSE	1	0	0	FALSE	FALSE
pmtcrn	InChI=1S/C23H45NO4/c1-5-6-7-8-9-10-11-12-13-14-15-16-17-18-23(27)28-21(19-22(25)26)20-24(2,3)4/h21H,5-20H2,1-4H3	Organic compounds	Lipids and lipid-like molecules	Fatty Acyls	Fatty acid esters	Acyl carnitines		TRUE	TRUE	TRUE	TRUE	FALSE	FALSE	FALSE	1	0	0	FALSE	FALSE
stcrn	InChI=1S/C25H49NO4/c1-5-6-7-8-9-10-11-12-13-14-15-16-17-18-19-20-25(29)30-23(21-24(27)28)22-26(2,3)4/h23H,5-22H2,1-4H3	Organic compounds	Lipids and lipid-like molecules	Fatty Acyls	Fatty acid esters	Acyl carnitines		TRUE	TRUE	TRUE	TRUE	FALSE	TRUE	FALSE	2	0	0	FALSE	FALSE
56935945	InChI=1S/C34H62O2/c1-3-5-7-9-11-13-15-17-18-19-21-23-25-27-29-31-33-36-34(35)32-30-28-26-24-22-20-16-14-12-10-8-6-4-2/h5,7,11,13,17-18H,3-4,6,8-10,12,14-16,19-33H2,1-2H3/b7-5-,13-11-,18-17-	Organic compounds	Lipids and lipid-like molecules	Fatty Acyls	Fatty acid esters	Wax esters	Wax monoesters	FALSE	FALSE	FALSE	FALSE	TRUE	FALSE	TRUE	0	2	0	FALSE	FALSE
56935960	InChI=1S/C36H66O2/c1-3-5-7-9-11-13-15-17-19-21-23-25-27-29-31-33-35-38-36(37)34-32-30-28-26-24-22-20-18-16-14-12-10-8-6-4-2/h5,7,11,13,17,19H,3-4,6,8-10,12,14-16,18,20-35H2,1-2H3/b7-5-,13-11-,19-17-	Organic compounds	Lipids and lipid-like molecules	Fatty Acyls	Fatty acid esters	Wax esters	Wax monoesters	FALSE	FALSE	FALSE	FALSE	TRUE	FALSE	FALSE	0	1	0	FALSE	FALSE
2hb	InChI=1S/C4H8O3/c1-2-3(5)4(6)7/h3,5H,2H2,1H3,(H,6,7)/p-2	Organic compounds	Lipids and lipid-like molecules	Fatty Acyls	Fatty acids and conjugates			TRUE	TRUE	TRUE	TRUE	FALSE	FALSE	FALSE	1	0	0	FALSE	FALSE
ethmalac	InChI=1S/C5H8O4/c1-2-3(4(6)7)5(8)9/h3H,2H2,1H3,(H,6,7)(H,8,9)/p-2	Organic compounds	Lipids and lipid-like molecules	Fatty Acyls	Fatty acids and conjugates	Branched fatty acids		TRUE	TRUE	TRUE	TRUE	FALSE	FALSE	FALSE	1	0	0	FALSE	FALSE
2mbut	InChI=1S/C5H10O2/c1-3-4(2)5(6)7/h4H,3H2,1-2H3,(H,6,7)	Organic compounds	Lipids and lipid-like molecules	Fatty Acyls	Fatty acids and conjugates	Branched fatty acids	Methyl-branched fatty acids	FALSE	FALSE	TRUE	FALSE	TRUE	FALSE	FALSE	0	1	0	FALSE	FALSE
isoval	InChI=1S/C5H10O2/c1-4(2)3-5(6)7/h4H,3H2,1-2H3,(H,6,7)	Organic compounds	Lipids and lipid-like molecules	Fatty Acyls	Fatty acids and conjugates	Branched fatty acids	Methyl-branched fatty acids	FALSE	FALSE	TRUE	TRUE	FALSE	FALSE	FALSE	1	0	0	FALSE	FALSE
193530	InChI=1S/C6H10O5/c7-4(6(10)11)2-1-3-5(8)9/h4,7H,1-3H2,(H,8,9)(H,10,11)	Organic compounds	Lipids and lipid-like molecules	Fatty Acyls	Fatty acids and conjugates	Dicarboxylic acids		FALSE	FALSE	FALSE	FALSE	TRUE	FALSE	FALSE	0	1	0	FALSE	FALSE
52921867	InChI=1S/C21H38O4/c1-3-4-5-6-7-8-9-10-11-12-13-14-15-16-17-19(21(24)25)18(2)20(22)23/h19H,2-17H2,1H3,(H,22,23)(H,24,25)/t19-/m1/s1	Organic compounds	Lipids and lipid-like molecules	Fatty Acyls	Fatty acids and conjugates	Dicarboxylic acids		FALSE	FALSE	FALSE	FALSE	TRUE	FALSE	FALSE	0	1	0	FALSE	FALSE

9543654	InChI=1S/C13H24O4/c1-11(13(16)17)9-7-5-3-2-4-6-8-10-12(14)15/h11H,2-10H2,1H3,(H,14,15)(H,16,17)	Organic compounds	Lipids and lipid-like molecules	Fatty Acyls	Fatty acids and conjugates	Dicarboxylic acids	FALSE	FALSE	FALSE	FALSE	TRUE	FALSE	TRUE	0	2	0	FALSE	FALSE
pime	InChI=1S/C7H12O4/c8-6(9)4-2-1-3-5-7(10)11/h1-5H2,(H,8,9)(H,10,11)	Organic compounds	Lipids and lipid-like molecules	Fatty Acyls	Fatty acids and conjugates	Dicarboxylic acids	FALSE	FALSE	TRUE	TRUE	FALSE	FALSE	FALSE	1	0	0	FALSE	FALSE
123979	InChI=1S/C12H16O5/c1-3-4-8-7(2)11(12(15)16)9(17-8)5-6-10(13)14/h3-6H2,1-2H3,(H,13,14)(H,15,16)	Organic compounds	Lipids and lipid-like molecules	Fatty Acyls	Fatty acids and conjugates	Heterocyclic fatty acids	FALSE	FALSE	FALSE	TRUE	TRUE	FALSE	FALSE	1	1	1	FALSE	FALSE
5282889	InChI=1S/C22H36O6/c1-3-17(26-24)14-15-19-18(20-16-21(19)28-27-20)12-10-8-6-4-5-7-9-11-13-22(23)25-2/h10,12,14-15,17-21,24H,3-9,11,13,16H2,1-2H3/b12-10+,15-14+	Organic compounds	Lipids and lipid-like molecules	Fatty Acyls	Fatty acids and conjugates	Hydroperoxy fatty acids	FALSE	FALSE	FALSE	TRUE	FALSE	TRUE	FALSE	2	0	0	FALSE	FALSE
3hivac	InChI=1S/C5H10O3/c1-5(2,8)3-4(6)7/h8H,3H2,1-2H3,(H,6,7)/p-1	Organic compounds	Lipids and lipid-like molecules	Fatty Acyls	Fatty acids and conjugates	Hydroxy fatty acids	TRUE	FALSE	TRUE	FALSE	TRUE	FALSE	FALSE	0	1	0	FALSE	FALSE
677	InChI=1S/C5H10O4/c1-5(2,9)3(6)4(7)8/h3,6,9H,1-2H3,(H,7,8)	Organic compounds	Lipids and lipid-like molecules	Fatty Acyls	Fatty acids and conjugates	Hydroxy fatty acids	FALSE	FALSE	FALSE	FALSE	TRUE	FALSE	FALSE	0	1	0	FALSE	FALSE
92836	InChI=1S/C16H32O3/c1-2-3-4-5-6-7-8-9-10-11-12-13-14-15(17)16(18)19/h15,17H,2-14H2,1H3,(H,18,19)	Organic compounds	Lipids and lipid-like molecules	Fatty Acyls	Fatty acids and conjugates	Hydroxy fatty acids	FALSE	FALSE	FALSE	TRUE	FALSE	FALSE	FALSE	1	0	0	FALSE	FALSE
99823	InChI=1S/C5H10O3/c1-3(2)4(6)5(7)8/h3-4,6H,1-2H3,(H,7,8)	Organic compounds	Lipids and lipid-like molecules	Fatty Acyls	Fatty acids and conjugates	Hydroxy fatty acids	FALSE	FALSE	FALSE	TRUE	TRUE	FALSE	FALSE	1	1	1	FALSE	FALSE
10236635	InChI=1S/C18H34O4/c1-2-3-10-13-16(19)17(20)14-11-8-6-4-5-7-9-12-15-18(21)22/h8,11,16-17,19-20H,2-7,9-10,12-15H2,1H3,(H,21,22)/b11-8-	Organic compounds	Lipids and lipid-like molecules	Fatty Acyls	Fatty acids and conjugates	Long-chain fatty acids	FALSE	FALSE	FALSE	FALSE	TRUE	FALSE	FALSE	0	1	0	FALSE	FALSE
17903417	InChI=1S/C17H34O2/c1-16(2)14-12-10-8-6-4-3-5-7-9-11-13-15-17(18)19/h16H,3-15H2,1-2H3,(H,18,19)/p-1	Organic compounds	Lipids and lipid-like molecules	Fatty Acyls	Fatty acids and conjugates	Long-chain fatty acids	FALSE	FALSE	FALSE	FALSE	TRUE	FALSE	FALSE	0	1	0	FALSE	FALSE
21993177	InChI=1S/C18H36O3/c1-2-3-4-5-6-7-8-9-10-11-12-13-14-15-16-17(19)18(20)21/h17,19H,2-16H2,1H3,(H,20,21)/p-1	Organic compounds	Lipids and lipid-like molecules	Fatty Acyls	Fatty acids and conjugates	Long-chain fatty acids	FALSE	FALSE	FALSE	FALSE	TRUE	FALSE	FALSE	0	1	0	FALSE	FALSE
9966640	InChI=1S/C18H34O4/c1-2-3-4-5-7-10-13-16(19)17(20)14-11-8-6-9-12-15-18(21)22/h7,10,16-17,19-20H,2-6,8-9,11-15H2,1H3,(H,21,22)/b10-7-	Organic compounds	Lipids and lipid-like molecules	Fatty Acyls	Fatty acids and conjugates	Long-chain fatty acids	FALSE	FALSE	FALSE	FALSE	TRUE	FALSE	FALSE	0	1	0	FALSE	FALSE
CE2047	InChI=1S/C18H34O4/c1-2-3-4-5-7-10-13-16(19)17(20)14-11-8-6-9-12-15-18(21)22/h7,10,16-17,19-20H,2-6,8-9,11-15H2,1H3,(H,21,22)/p-1/b10-7-	Organic compounds	Lipids and lipid-like molecules	Fatty Acyls	Fatty acids and conjugates	Long-chain fatty acids	TRUE	FALSE	TRUE	TRUE	FALSE	FALSE	FALSE	1	0	0	FALSE	FALSE
CE2510	InChI=1S/C20H38O2/c1-2-3-4-5-6-7-8-9-10-11-12-13-14-15-16-17-18-19-20(21)22/h9-10H,2-8,11-19H2,1H3,(H,21,22)/p-1/b10-9-	Organic compounds	Lipids and lipid-like molecules	Fatty Acyls	Fatty acids and conjugates	Long-chain fatty acids	TRUE	FALSE	TRUE	TRUE	FALSE	FALSE	FALSE	1	0	0	FALSE	FALSE
CE2516	InChI=1S/C20H34O2/c1-2-3-4-5-6-7-8-9-10-11-12-13-14-15-16-17-18-19-20(21)	Organic compounds	Lipids and lipid-like molecules	Fatty Acyls	Fatty acids and conjugates	Long-chain fatty acids	TRUE	FALSE	TRUE	FALSE	TRUE	FALSE	FALSE	0	1	0	FALSE	FALSE

	22/h6-7,9-10,12-13H,2-5,8,11,14-19H2,1H3,(H,21,22)/p-1																	
FFA 14:1		Organic compounds	Lipids and lipid-like molecules	Fatty Acyls	Fatty acids and conjugates	Long-chain fatty acids	FALSE	FALSE	FALSE	FALSE	TRUE	FALSE	FALSE	0	1	0	FALSE	FALSE
FFA 16:2		Organic compounds	Lipids and lipid-like molecules	Fatty Acyls	Fatty acids and conjugates	Long-chain fatty acids	FALSE	FALSE	FALSE	FALSE	TRUE	FALSE	FALSE	0	1	0	FALSE	FALSE
FFA 18:0		Organic compounds	Lipids and lipid-like molecules	Fatty Acyls	Fatty acids and conjugates	Long-chain fatty acids	FALSE	FALSE	FALSE	FALSE	TRUE	FALSE	TRUE	0	2	0	FALSE	FALSE
FFA 18:1		Organic compounds	Lipids and lipid-like molecules	Fatty Acyls	Fatty acids and conjugates	Long-chain fatty acids	FALSE	FALSE	FALSE	FALSE	TRUE	FALSE	FALSE	0	1	0	FALSE	FALSE
FFA 18:2		Organic compounds	Lipids and lipid-like molecules	Fatty Acyls	Fatty acids and conjugates	Long-chain fatty acids	FALSE	FALSE	FALSE	FALSE	TRUE	FALSE	FALSE	0	1	0	FALSE	FALSE
FFA 18:3		Organic compounds	Lipids and lipid-like molecules	Fatty Acyls	Fatty acids and conjugates	Long-chain fatty acids	FALSE	FALSE	FALSE	FALSE	TRUE	FALSE	FALSE	0	1	0	FALSE	FALSE
FFA 19:1		Organic compounds	Lipids and lipid-like molecules	Fatty Acyls	Fatty acids and conjugates	Long-chain fatty acids	FALSE	FALSE	FALSE	FALSE	TRUE	FALSE	FALSE	0	1	0	FALSE	FALSE
FFA 20:0		Organic compounds	Lipids and lipid-like molecules	Fatty Acyls	Fatty acids and conjugates	Long-chain fatty acids	FALSE	FALSE	FALSE	FALSE	TRUE	FALSE	TRUE	0	2	0	FALSE	FALSE
FFA 20:2		Organic compounds	Lipids and lipid-like molecules	Fatty Acyls	Fatty acids and conjugates	Long-chain fatty acids	FALSE	FALSE	FALSE	FALSE	TRUE	FALSE	FALSE	0	1	0	FALSE	FALSE
FFA 20:3		Organic compounds	Lipids and lipid-like molecules	Fatty Acyls	Fatty acids and conjugates	Long-chain fatty acids	FALSE	FALSE	FALSE	FALSE	TRUE	FALSE	FALSE	0	1	0	FALSE	FALSE
FFA 20:4		Organic compounds	Lipids and lipid-like molecules	Fatty Acyls	Fatty acids and conjugates	Long-chain fatty acids	FALSE	FALSE	FALSE	FALSE	TRUE	FALSE	FALSE	0	1	0	FALSE	FALSE
FFA 20:5		Organic compounds	Lipids and lipid-like molecules	Fatty Acyls	Fatty acids and conjugates	Long-chain fatty acids	FALSE	FALSE	FALSE	FALSE	TRUE	FALSE	FALSE	0	1	0	FALSE	FALSE
FFA 22:5		Organic compounds	Lipids and lipid-like molecules	Fatty Acyls	Fatty acids and conjugates	Long-chain fatty acids	FALSE	FALSE	FALSE	FALSE	TRUE	FALSE	FALSE	0	1	0	FALSE	FALSE
FFA 22:6		Organic compounds	Lipids and lipid-like molecules	Fatty Acyls	Fatty acids and conjugates	Long-chain fatty acids	FALSE	FALSE	FALSE	FALSE	TRUE	FALSE	FALSE	0	1	0	FALSE	FALSE
arach	InChI=1S/C20H40O2/c1-2-3-4-5-6-7-8-9-10-11-12-13-14-15-16-17-18-19-20(21)22/h2-19H2,1H3,(H,21,22)/p-1	Organic compounds	Lipids and lipid-like molecules	Fatty Acyls	Fatty acids and conjugates	Long-chain fatty acids	TRUE	TRUE	TRUE	TRUE	FALSE	FALSE	FALSE	1	0	0	FALSE	FALSE
arachd	InChI=1S/C20H32O2/c1-2-3-4-5-6-7-8-9-10-11-12-13-14-15-16-17-18-19-20(21)22/h6-7,9-10,12-13,15-16H,2-5,8,11,14,17-19H2,1H3,(H,21,22)/p-1/b7-6-,10-9-,13-12-,16-15-	Organic compounds	Lipids and lipid-like molecules	Fatty Acyls	Fatty acids and conjugates	Long-chain fatty acids	TRUE	TRUE	TRUE	TRUE	TRUE	TRUE	TRUE	3	2	1	FALSE	FALSE
dlnlcg	InChI=1S/C20H34O2/c1-2-3-4-5-6-7-8-9-10-11-12-13-14-15-16-17-18-19-20(21)22/h6-7,9-10,12-13H,2-5,8,11,14-19H2,1H3,(H,21,22)/p-1/b7-6-,10-9-,13-12-	Organic compounds	Lipids and lipid-like molecules	Fatty Acyls	Fatty acids and conjugates	Long-chain fatty acids	TRUE	FALSE	TRUE	TRUE	FALSE	TRUE	FALSE	3	0	0	FALSE	FALSE
eidi1114ac	InChI=1S/C20H36O2/c1-2-3-4-5-6-7-8-9-10-11-12-13-14-15-16-17-18-19-20(21)22/h16-19H,2-15H2,1H3,(H,21,22)/b17-16+,19-18+	Organic compounds	Lipids and lipid-like molecules	Fatty Acyls	Fatty acids and conjugates	Long-chain fatty acids	TRUE	TRUE	TRUE	TRUE	TRUE	FALSE	FALSE	1	1	1	FALSE	FALSE
hdca	InChI=1S/C16H32O2/c1-2-3-4-5-6-7-8-9-10-11-12-13-14-15-16(17)18/h2-15H2,1H3,(H,17,18)/p-1	Organic compounds	Lipids and lipid-like molecules	Fatty Acyls	Fatty acids and conjugates	Long-chain fatty acids	TRUE	TRUE	TRUE	TRUE	TRUE	TRUE	TRUE	2	3	1	TRUE	FALSE
hdcea	InChI=1S/C16H30O2/c1-2-3-4-5-6-7-8-9-10-11-12-13-14-15-16(17)18/h7-8H,2-6,9-15H2,1H3,(H,17,18)/p-1/b8-7-	Organic compounds	Lipids and lipid-like molecules	Fatty Acyls	Fatty acids and conjugates	Long-chain fatty acids	TRUE	TRUE	TRUE	TRUE	TRUE	FALSE	TRUE	1	2	1	FALSE	FALSE

hpdca	InChI=1S/C17H34O2/c1-2-3-4-5-6-7-8-9-10-11-12-13-14-15-16-17(18)19/h2-16H2,1H3,(H,18,19)/p-1	Organic compounds	Lipids and lipid-like molecules	Fatty Acyls	Fatty acids and conjugates	Long-chain fatty acids	TRUE	TRUE	TRUE	TRUE	TRUE	FALSE	TRUE	1	2	1	FALSE	FALSE
leuktrB4wcooh	InChI=1S/C20H30O6/c21-17(11-6-2-1-3-9-15-19(23)24)12-7-4-5-8-13-18(22)14-10-16-20(25)26/h2,4-8,12-13,17-18,21-22H,1,3,9-11,14-16H2,(H,23,24)(H,25,26)/p-2/b5-4+,6-2-,12-7?,13-8-/t17-,18-/m1/s1	Organic compounds	Lipids and lipid-like molecules	Fatty Acyls	Fatty acids and conjugates	Long-chain fatty acids	TRUE	TRUE	TRUE	TRUE	FALSE	FALSE	FALSE	1	0	0	FALSE	FALSE
ocdca	InChI=1S/C18H36O2/c1-2-3-4-5-6-7-8-9-10-11-12-13-14-15-16-17-18(19)20/h2-17H2,1H3,(H,19,20)/p-1	Organic compounds	Lipids and lipid-like molecules	Fatty Acyls	Fatty acids and conjugates	Long-chain fatty acids	TRUE	FALSE	TRUE	TRUE	TRUE	TRUE	TRUE	2	3	1	FALSE	FALSE
ocdcea	InChI=1S/C18H34O2/c1-2-3-4-5-6-7-8-9-10-11-12-13-14-15-16-17-18(19)20/h9-10H,2-8,11-17H2,1H3,(H,19,20)/p-1/b10-9-	Organic compounds	Lipids and lipid-like molecules	Fatty Acyls	Fatty acids and conjugates	Long-chain fatty acids	TRUE	TRUE	TRUE	TRUE	TRUE	FALSE	TRUE	1	4	1	FALSE	FALSE
ptdca	InChI=1S/C15H30O2/c1-2-3-4-5-6-7-8-9-10-11-12-13-14-15(16)17/h2-14H2,1H3,(H,16,17)/p-1	Organic compounds	Lipids and lipid-like molecules	Fatty Acyls	Fatty acids and conjugates	Long-chain fatty acids	TRUE	FALSE	TRUE	TRUE	FALSE	FALSE	FALSE	1	0	0	FALSE	FALSE
tmndnc	InChI=1S/C20H30O2/c1-2-3-4-5-6-7-8-9-10-11-12-13-14-15-16-17-18-19-20(21)22/h3-4,6-7,9-10,12-13,15-16H,2,5,8,11,14,17-19H2,1H3,(H,21,22)/p-1/b4-3-,7-6-,10-9-,13-12-,16-15-	Organic compounds	Lipids and lipid-like molecules	Fatty Acyls	Fatty acids and conjugates	Long-chain fatty acids	TRUE	FALSE	TRUE	FALSE	TRUE	FALSE	FALSE	0	1	0	FALSE	FALSE
ttdca	InChI=1S/C14H28O2/c1-2-3-4-5-6-7-8-9-10-11-12-13-14(15)16/h2-13H2,1H3,(H,15,16)/p-1	Organic compounds	Lipids and lipid-like molecules	Fatty Acyls	Fatty acids and conjugates	Long-chain fatty acids	TRUE	FALSE	TRUE	TRUE	FALSE	TRUE	FALSE	2	0	0	TRUE	FALSE
6079438	InChI=1S/C8H12O4/c9-7(10)5-3-1-2-4-6-8(11)12/h3,5H,1-2,4,6H2,(H,9,10)(H,11,12)/b5-3+	Organic compounds	Lipids and lipid-like molecules	Fatty Acyls	Fatty acids and conjugates	Medium-chain fatty acids	FALSE	FALSE	FALSE	FALSE	TRUE	FALSE	FALSE	0	1	0	FALSE	FALSE
C01601	InChI=1S/C9H18O2/c1-2-3-4-5-6-7-8-9(10)11/h2-8H2,1H3,(H,10,11)/p-1	Organic compounds	Lipids and lipid-like molecules	Fatty Acyls	Fatty acids and conjugates	Medium-chain fatty acids	TRUE	FALSE	TRUE	TRUE	FALSE	FALSE	FALSE	1	0	0	FALSE	FALSE
FFA 10:0		Organic compounds	Lipids and lipid-like molecules	Fatty Acyls	Fatty acids and conjugates	Medium-chain fatty acids	FALSE	FALSE	FALSE	FALSE	TRUE	FALSE	FALSE	0	1	0	FALSE	FALSE
FFA 11:1		Organic compounds	Lipids and lipid-like molecules	Fatty Acyls	Fatty acids and conjugates	Medium-chain fatty acids	FALSE	FALSE	FALSE	FALSE	TRUE	FALSE	FALSE	0	1	0	FALSE	FALSE
FFA 12:0		Organic compounds	Lipids and lipid-like molecules	Fatty Acyls	Fatty acids and conjugates	Medium-chain fatty acids	FALSE	FALSE	FALSE	FALSE	TRUE	FALSE	FALSE	0	1	0	FALSE	FALSE
Octenoic acid		Organic compounds	Lipids and lipid-like molecules	Fatty Acyls	Fatty acids and conjugates	Medium-chain fatty acids	FALSE	FALSE	FALSE	FALSE	TRUE	FALSE	FALSE	0	1	0	FALSE	FALSE
dca	InChI=1S/C10H20O2/c1-2-3-4-5-6-7-8-9-10(11)12/h2-9H2,1H3,(H,11,12)/p-1	Organic compounds	Lipids and lipid-like molecules	Fatty Acyls	Fatty acids and conjugates	Medium-chain fatty acids	TRUE	FALSE	TRUE	TRUE	FALSE	TRUE	FALSE	3	0	0	FALSE	FALSE
ddca	InChI=1S/C12H24O2/c1-2-3-4-5-6-7-8-9-10-11-12(13)14/h2-11H2,1H3,(H,13,14)/p-1	Organic compounds	Lipids and lipid-like molecules	Fatty Acyls	Fatty acids and conjugates	Medium-chain fatty acids	TRUE	TRUE	TRUE	TRUE	FALSE	TRUE	FALSE	2	0	0	FALSE	FALSE
dodecanac	InChI=1S/C12H22O4/c13-11(14)9-7-5-3-1-2-4-6-8-10-12(15)16/h1-10H2,(H,13,14)(H,15,16)/p-2	Organic compounds	Lipids and lipid-like molecules	Fatty Acyls	Fatty acids and conjugates	Medium-chain fatty acids	TRUE	FALSE	TRUE	TRUE	FALSE	FALSE	FALSE	1	0	0	FALSE	FALSE
octa	InChI=1S/C8H16O2/c1-2-3-4-5-6-7-8(9)10/h2-7H2,1H3,(H,9,10)/p-1	Organic compounds	Lipids and lipid-like molecules	Fatty Acyls	Fatty acids and conjugates	Medium-chain fatty acids	TRUE	TRUE	TRUE	TRUE	FALSE	TRUE	FALSE	2	0	0	TRUE	FALSE

sebacid	InChI=1S/C10H18O4/c11-9(12)7-5-3-1-2-4-6-8-10(13)14/h1-8H2,(H,11,12)(H,13,14)/p-2	Organic compounds	Lipids and lipid-like molecules	Fatty Acyls	Fatty acids and conjugates	Medium-chain fatty acids		TRUE	FALSE	TRUE	TRUE	TRUE	FALSE	TRUE	1	2	1	FALSE	FALSE
subeac	InChI=1S/C8H14O4/c9-7(10)5-3-1-2-4-6-8(11)12/h1-6H2,(H,9,10)(H,11,12)/p-2	Organic compounds	Lipids and lipid-like molecules	Fatty Acyls	Fatty acids and conjugates	Medium-chain fatty acids		TRUE	FALSE	TRUE	TRUE	TRUE	FALSE	FALSE	1	1	1	FALSE	FALSE
3083779	InChI=1S/C19H38O2/c1-18(2)16-14-12-10-8-6-4-3-5-7-9-11-13-15-17-19(20)21/h18H,3-17H2,1-2H3,(H,20,21)	Organic compounds	Lipids and lipid-like molecules	Fatty Acyls	Fatty acids and conjugates	Methyl-branched fatty acids		FALSE	FALSE	FALSE	TRUE	FALSE	FALSE	FALSE	1	0	0	FALSE	FALSE
5282651	InChI=1S/C8H14O2/c1-3-4-5-6-7(2)8(9)10/h6H,3-5H2,1-2H3,(H,9,10)/b7-6+	Organic compounds	Lipids and lipid-like molecules	Fatty Acyls	Fatty acids and conjugates	Methyl-branched fatty acids		FALSE	FALSE	FALSE	TRUE	FALSE	FALSE	FALSE	1	0	0	FALSE	FALSE
5282655	InChI=1S/C13H24O2/c1-3-4-5-6-7-8-9-10-11-12(2)13(14)15/h11H,3-10H2,1-2H3,(H,14,15)/b12-11+	Organic compounds	Lipids and lipid-like molecules	Fatty Acyls	Fatty acids and conjugates	Methyl-branched fatty acids		FALSE	FALSE	FALSE	TRUE	FALSE	FALSE	FALSE	1	0	0	FALSE	FALSE
5282669	InChI=1S/C11H20O2/c1-4-9(2)7-5-6-8-10(3)11(12)13/h5,7,9-10H,4,6,8H2,1-3H3,(H,12,13)/b7-5+	Organic compounds	Lipids and lipid-like molecules	Fatty Acyls	Fatty acids and conjugates	Methyl-branched fatty acids		FALSE	FALSE	FALSE	TRUE	FALSE	FALSE	FALSE	1	0	0	FALSE	FALSE
52921765	InChI=1S/C27H54O3/c1-5-6-7-8-9-10-11-12-13-14-15-16-17-18-19-20-21-23(2)22-24(3)26(28)25(4)27(29)30/h23-26,28H,5-22H2,1-4H3,(H,29,30)/t23-,24-,25-,26-/m0/s1	Organic compounds	Lipids and lipid-like molecules	Fatty Acyls	Fatty acids and conjugates	Methyl-branched fatty acids		FALSE	FALSE	FALSE	FALSE	TRUE	FALSE	TRUE	0	2	0	FALSE	FALSE
151014	InChI=1S/C15H30O2/c1-14(2)12-10-8-6-4-3-5-7-9-11-13-15(16)17/h14H,3-13H2,1-2H3,(H,16,17)	Organic compounds	Lipids and lipid-like molecules	Fatty Acyls	Fatty acids and conjugates	Methyl-branched fatty acids	Fatty acids with an aliphatic tail that contains between 13 and 21 carbon atoms.	FALSE	FALSE	FALSE	TRUE	FALSE	FALSE	FALSE	1	0	0	FALSE	FALSE
5312916	InChI=1S/C19H36O3/c1-2-3-4-5-6-9-12-15-18(20)16-13-10-7-8-11-14-17-19(21)22/h2-17H2,1H3,(H,21,22)	Organic compounds	Lipids and lipid-like molecules	Fatty Acyls	Fatty acids and conjugates	Oxo fatty acids		FALSE	FALSE	FALSE	FALSE	TRUE	FALSE	FALSE	0	1	0	FALSE	FALSE
5312936	InChI=1S/C26H48O3/c1-2-3-4-5-13-16-19-22-25(27)23-20-17-14-11-9-7-6-8-10-12-15-18-21-24-26(28)29/h13,16H,2-12,14-15,17-24H2,1H3,(H,28,29)/b16-13-	Organic compounds	Lipids and lipid-like molecules	Fatty Acyls	Fatty acids and conjugates	Oxo fatty acids		FALSE	FALSE	FALSE	FALSE	TRUE	FALSE	FALSE	0	1	0	FALSE	FALSE
4tmeabutn	InChI=1S/C7H15NO2/c1-8(2,3)6-4-5-7(9)10/h4-6H2,1-3H3	Organic compounds	Lipids and lipid-like molecules	Fatty Acyls	Fatty acids and conjugates	Straight chain fatty acids		TRUE	FALSE	TRUE	TRUE	TRUE	TRUE	FALSE	2	1	1	FALSE	FALSE
M02613	InChI=1S/C19H38O2/c1-2-3-4-5-6-7-8-9-10-11-12-13-14-15-16-17-18-19(20)21/h2-18H2,1H3,(H,20,21)	Organic compounds	Lipids and lipid-like molecules	Fatty Acyls	Fatty acids and conjugates	Straight chain fatty acids		TRUE	FALSE	TRUE	TRUE	FALSE	FALSE	FALSE	1	0	0	FALSE	FALSE
M03134	InChI=1S/C5H10O2/c1-2-3-4-5(6)7/h2-4H2,1H3,(H,6,7)	Organic compounds	Lipids and lipid-like molecules	Fatty Acyls	Fatty acids and conjugates	Straight chain fatty acids		TRUE	FALSE	TRUE	TRUE	TRUE	FALSE	TRUE	1	3	1	FALSE	FALSE
but	InChI=1S/C4H8O2/c1-2-3-4(5)6/h2-3H2,1H3,(H,5,6)/p-1	Organic compounds	Lipids and lipid-like molecules	Fatty Acyls	Fatty acids and conjugates	Straight chain fatty acids		TRUE	TRUE	TRUE	TRUE	TRUE	TRUE	TRUE	2	3	1	TRUE	FALSE
131751124	InChI=1S/C10H8O3/c11-9-7-5-3-1-2-4-6-8-10(12)13/h5-8,11H,9H2,(H,12,13)/b7-5-,8-6+	Organic compounds	Lipids and lipid-like molecules	Fatty Acyls	Fatty acids and conjugates	Unsaturated fatty acids		FALSE	FALSE	FALSE	FALSE	TRUE	FALSE	FALSE	0	1	0	FALSE	FALSE

1449	InChI=1S/C18H32O2/c1-2-3-4-5-6-7-8-9-10-11-12-13-14-15-16-17-18(19)20/h1H,3-17H2,(H,19,20)	Organic compounds	Lipids and lipid-like molecules	Fatty Acyls	Fatty acids and conjugates	Unsaturated fatty acids	FALSE	FALSE	FALSE	TRUE	FALSE	FALSE	FALSE	1	0	0	FALSE	FALSE
5281119	InChI=1S/C14H26O2/c1-2-3-4-5-6-7-8-9-10-11-12-13-14(15)16/h5-6H,2-4,7-13H2,1H3,(H,15,16)/b6-5-	Organic compounds	Lipids and lipid-like molecules	Fatty Acyls	Fatty acids and conjugates	Unsaturated fatty acids	FALSE	FALSE	FALSE	TRUE	FALSE	FALSE	FALSE	1	0	0	FALSE	FALSE
52921835	InChI=1S/C21H36O2/c1-2-3-4-5-6-7-8-9-10-11-12-13-14-15-16-17-18-19-20-21(22)23/h4-5,7-8,16-17H,2-3,6,9-15,18-20H2,1H3,(H,22,23)/b5-4-,8-7-,17-16-	Organic compounds	Lipids and lipid-like molecules	Fatty Acyls	Fatty acids and conjugates	Unsaturated fatty acids	FALSE	FALSE	FALSE	TRUE	FALSE	TRUE	FALSE	2	0	0	FALSE	FALSE
5312378	InChI=1S/C12H22O2/c1-2-3-4-5-6-7-8-9-10-11-12(13)14/h7-8H,2-6,9-11H2,1H3,(H,13,14)/b8-7-	Organic compounds	Lipids and lipid-like molecules	Fatty Acyls	Fatty acids and conjugates	Unsaturated fatty acids	FALSE	FALSE	FALSE	TRUE	FALSE	FALSE	FALSE	1	0	0	FALSE	FALSE
5312513	InChI=1S/C19H36O2/c1-2-3-4-5-6-7-8-9-10-11-12-13-14-15-16-17-18-19(20)21/h9-10H,2-8,11-18H2,1H3,(H,20,21)/b10-9-	Organic compounds	Lipids and lipid-like molecules	Fatty Acyls	Fatty acids and conjugates	Unsaturated fatty acids	FALSE	FALSE	FALSE	TRUE	TRUE	FALSE	FALSE	1	1	1	FALSE	FALSE
HC01415	InChI=1S/C8H14O2/c1-2-3-4-5-6-7-8(9)10/h6-7H,2-5H2,1H3,(H,9,10)/b7-6+	Organic compounds	Lipids and lipid-like molecules	Fatty Acyls	Fatty acids and conjugates	Unsaturated fatty acids	TRUE	FALSE	TRUE	TRUE	FALSE	FALSE	FALSE	1	0	0	FALSE	FALSE
M00003	InChI=1S/C17H32O2/c1-2-3-4-5-6-7-8-9-10-11-12-13-14-15-16-17(18)19/h7-8H,2-6,9-16H2,1H3,(H,18,19)/b8-7-	Organic compounds	Lipids and lipid-like molecules	Fatty Acyls	Fatty acids and conjugates	Unsaturated fatty acids	TRUE	TRUE	TRUE	TRUE	TRUE	TRUE	FALSE	2	1	1	FALSE	FALSE
M00021	InChI=1S/C22H40O2/c1-2-3-4-5-6-7-8-9-10-11-12-13-14-15-16-17-18-19-20-21-22(23)24/h6-7,9-10H,2-5,8,11-21H2,1H3,(H,23,24)/b7-6+,10-9+	Organic compounds	Lipids and lipid-like molecules	Fatty Acyls	Fatty acids and conjugates	Unsaturated fatty acids	TRUE	FALSE	TRUE	TRUE	FALSE	FALSE	FALSE	1	0	0	FALSE	FALSE
M01235	InChI=1S/C20H38O2/c1-2-3-4-5-6-7-8-9-10-11-12-13-14-15-16-17-18-19-20(21)22/h11-12H,2-10,13-19H2,1H3,(H,21,22)/b12-11-	Organic compounds	Lipids and lipid-like molecules	Fatty Acyls	Fatty acids and conjugates	Unsaturated fatty acids	TRUE	FALSE	TRUE	TRUE	FALSE	FALSE	FALSE	1	0	0	FALSE	FALSE
M02457	InChI=1S/C20H34O2/c1-2-3-4-5-6-7-8-9-10-11-12-13-14-15-16-17-18-19-20(21)22/h9-10,12-13,15-16H,2-8,11,14,17-19H2,1H3,(H,21,22)/b10-9+,13-12+,16-15+	Organic compounds	Lipids and lipid-like molecules	Fatty Acyls	Fatty acids and conjugates	Unsaturated fatty acids	TRUE	FALSE	TRUE	TRUE	FALSE	FALSE	FALSE	1	0	0	FALSE	FALSE
adrn	InChI=1S/C22H36O2/c1-2-3-4-5-6-7-8-9-10-11-12-13-14-15-16-17-18-19-20-21-22(23)24/h6-7,9-10,12-13,15-16H,2-5,8,11,14,17-21H2,1H3,(H,23,24)/b7-6+,10-9+,13-12+,16-15+	Organic compounds	Lipids and lipid-like molecules	Fatty Acyls	Fatty acids and conjugates	Very long-chain fatty acids	TRUE	FALSE	TRUE	FALSE	TRUE	FALSE	FALSE	0	1	0	FALSE	FALSE
clpnd	InChI=1S/C22H34O2/c1-2-3-4-5-6-7-8-9-10-11-12-13-14-15-16-17-18-19-20-21-22(23)24/h3-4,6-7,9-10,12-13,15-16H,2,5,8,11,14,17-21H2,1H3,(H,23,24)/p-1/b4-3-,7-6-,10-9?,13-12-,16-15-	Organic compounds	Lipids and lipid-like molecules	Fatty Acyls	Fatty acids and conjugates	Very long-chain fatty acids	TRUE	FALSE	TRUE	TRUE	TRUE	FALSE	TRUE	1	2	1	FALSE	FALSE
crvnc	InChI=1S/C22H32O2/c1-2-3-4-5-6-7-8-9-10-11-12-13-14-15-16-17-18-19-20-21-22(23)24/h3-4,6-7,9	Organic compounds	Lipids and lipid-like molecules	Fatty Acyls	Fatty acids and conjugates	Very long-chain fatty acids	TRUE	FALSE	TRUE	TRUE	TRUE	FALSE	FALSE	1	1	1	FALSE	FALSE

	-10,12-13,15-16,18-19H,2,5,8,11,14,17,20-21H2,1H3,(H,23,24)/p-1/b4-3?,7-6-,10-9-,13-12-,16-15-,19-18-																	
doco13ac	InChI=1S/C22H42O2/c1-2-3-4-5-6-7-8-9-10-11-12-13-14-15-16-17-18-19-20-21-22(23)24/h9-10H,2-8,11-21H2,1H3,(H,23,24)/p-1/b10-9-	Organic compounds	Lipids and lipid-like molecules	Fatty Acyls	Fatty acids and conjugates	Very long-chain fatty acids	TRUE	FALSE	TRUE	TRUE	FALSE	FALSE	FALSE	1	0	0	FALSE	FALSE
docosac	InChI=1S/C22H44O2/c1-2-3-4-5-6-7-8-9-10-11-12-13-14-15-16-17-18-19-20-21-22(23)24/h2-21H2,1H3,(H,23,24)/p-1	Organic compounds	Lipids and lipid-like molecules	Fatty Acyls	Fatty acids and conjugates	Very long-chain fatty acids	TRUE	TRUE	TRUE	FALSE	TRUE	FALSE	FALSE	0	1	0	FALSE	FALSE
hexc	InChI=1S/C26H52O2/c1-2-3-4-5-6-7-8-9-10-11-12-13-14-15-16-17-18-19-20-21-22-23-24-25-26(27)28/h2-25H2,1H3,(H,27,28)/p-1	Organic compounds	Lipids and lipid-like molecules	Fatty Acyls	Fatty acids and conjugates	Very long-chain fatty acids	TRUE	FALSE	TRUE	FALSE	TRUE	FALSE	TRUE	0	3	0	FALSE	FALSE
lgnc	InChI=1S/C24H48O2/c1-2-3-4-5-6-7-8-9-10-11-12-13-14-15-16-17-18-19-20-21-22-23-24(25)26/h2-23H2,1H3,(H,25,26)/p-1	Organic compounds	Lipids and lipid-like molecules	Fatty Acyls	Fatty acids and conjugates	Very long-chain fatty acids	TRUE	FALSE	TRUE	FALSE	TRUE	FALSE	FALSE	0	1	0	FALSE	FALSE
443242	InChI=1S/C9H17O12P/c10-1-3-5(11)6(12)7(13)9(20-3)21-4(8(14)15)2-19-22(16,17)18/h3-7,9-13H,1-2H2,(H,14,15)(H2,16,17,18)/t3-,4?,5-,6+,7+,9-/m1/s1	Organic compounds	Lipids and lipid-like molecules	Fatty Acyls	Fatty acyl glycosides	Fatty acyl glycosides of mono- and disaccharides	FALSE	FALSE	FALSE	FALSE	TRUE	FALSE	FALSE	0	1	0	FALSE	FALSE
5460194	InChI=1S/C9H16O9/c10-1-3-5(12)6(13)7(14)9(17-3)18-4(2-11)8(15)16/h3-7,9-14H,1-2H2,(H,15,16)/t3-,4-,5-,6+,7+,9-/m1/s1	Organic compounds	Lipids and lipid-like molecules	Fatty Acyls	Fatty acyl glycosides	Fatty acyl glycosides of mono- and disaccharides	FALSE	FALSE	FALSE	TRUE	FALSE	TRUE	FALSE	2	0	0	FALSE	FALSE
3736	InChI=1S/C18H24I3N3O8/c1-24(4-9(28)6-26)18(31)12-13(19)11(17(30)22-3-8(27)5-25)14(20)16(15(12)21)23-10(29)7-32-2/h8-9,25-28H,3-7H2,1-2H3,(H,22,30)(H,23,29)	Organic compounds	Lipids and lipid-like molecules	Fatty Acyls	Fatty acyl thioesters	Acyl CoAs	FALSE	FALSE	FALSE	TRUE	FALSE	FALSE	FALSE	1	0	0	FALSE	FALSE
3mgcoa	InChI=1S/C27H42N7O19P3S/c1-14(8-17(36)37)9-18(38)57-7-6-29-16(35)4-5-30-25(41)22(40)27(2,3)11-50-56(47,48)53-55(45,46)49-10-15-21(52-54(42,43)44)20(39)26(51-15)34-13-33-19-23(28)31-12-32-24(19)34/h9,12-13,15,20-22,26,39-40H,4-8,10-11H2,1-3H3,(H,29,35)(H,30,41)(H,36,37)(H,45,46)(H,47,48)(H2,28,31,32)(H2,42,43,44)/b14-9+	Organic compounds	Lipids and lipid-like molecules	Fatty Acyls	Fatty acyl thioesters	Acyl CoAs	TRUE	TRUE	TRUE	TRUE	FALSE	FALSE	FALSE	1	0	0	FALSE	FALSE
phaccoa	InChI=1S/C29H42N7O17P3S/c1-29(2,24(40)27(41)32-9-8-19(37)31-10-11-57-20(38)12-17-6-4-3-5-7-17)14-50-56(47,48)53-55(45,46)49-13-18-23(52-54(42,43)44)22(39)28(51-18)36-16-35-21-25(30)33-15-34-26(21)36/h3-7,15-16,18,22-24,28,39-40H,8-14H2,1-2H3,(H,31,37)(H	Organic compounds	Lipids and lipid-like molecules	Fatty Acyls	Fatty acyl thioesters	Acyl CoAs	TRUE	FALSE	TRUE	TRUE	FALSE	FALSE	FALSE	1	0	0	FALSE	FALSE

	,32,41)(H,45,46)(H,47,48)(H2,30,33,34)(H2,42,43,44)/p-4/t18-,22-,23-,24+,28-/m1/s1																		
92153	InChI=1S/C25H40N7O18P3S/c1-13(33)8-16(35)54-7-6-27-15(34)4-5-28-23(38)20(37)25(2,3)10-47-53(44,45)50-52(42,43)46-9-14-19(49-51(39,40)41)18(36)24(48-14)32-12-31-17-21(26)29-11-30-22(17)32/h11-12,14,18-20,24,36-37H,4-10H2,1-3H3,(H,27,34)(H,28,38)(H,42,43)(H,44,45)(H2,26,29,30)(H2,39,40,41)/t14-,18-,19-,20+,24-/m1/s1	Organic compounds	Lipids and lipid-like molecules	Fatty Acyls	Fatty acyl thioesters	Acyl CoAs	3-oxo-acyl CoAs	FALSE	FALSE	FALSE	TRUE	FALSE	FALSE	FALSE	1	0	0	FALSE	FALSE
CE5126	InChI=1S/C40H72N7O17P3S/c1-25(2)11-8-12-26(3)13-9-14-27(4)15-10-16-28(5)39(52)68-20-19-42-30(48)17-18-43-37(51)34(50)40(6,7)22-61-67(58,59)64-66(56,57)60-21-29-33(63-65(53,54)55)32(49)38(62-29)47-24-46-31-35(41)44-23-45-36(31)47/h23-29,32-34,38,49-50H,8-22H2,1-7H3,(H,42,48)(H,43,51)(H,56,57)(H,58,59)(H2,41,44,45)(H2,53,54,55)/p-4/t26?,27?,28?,29?,32?,33?,34?,38-/m0/s1	Organic compounds	Lipids and lipid-like molecules	Fatty Acyls	Fatty acyl thioesters	Acyl CoAs	Long-chain fatty acyl CoAs	TRUE	FALSE	TRUE	TRUE	TRUE	TRUE	TRUE	4	2	1	FALSE	FALSE
CE2006	InChI=1S/C9H16O2/c1-2-3-4-6-9(11)7-5-8-10/h5,7-9,11H,2-4,6H2,1H3	Organic compounds	Lipids and lipid-like molecules	Fatty Acyls	Fatty alcohols			TRUE	FALSE	TRUE	TRUE	FALSE	FALSE	FALSE	1	0	0	FALSE	FALSE
5283300	InChI=1S/C20H40O/c1-2-3-4-5-6-7-8-9-10-11-12-13-14-15-16-17-18-19-20-21/h9-10,21H,2-8,11-20H2,1H3/b10-9-	Organic compounds	Lipids and lipid-like molecules	Fatty Acyls	Fatty alcohols	Long-chain fatty alcohols		FALSE	FALSE	FALSE	TRUE	FALSE	FALSE	FALSE	1	0	0	FALSE	FALSE
8221	InChI=1S/C18H38O/c1-2-3-4-5-6-7-8-9-10-11-12-13-14-15-16-17-18-19/h19H,2-18H2,1H3	Organic compounds	Lipids and lipid-like molecules	Fatty Acyls	Fatty alcohols	Long-chain fatty alcohols		FALSE	FALSE	FALSE	TRUE	FALSE	FALSE	FALSE	1	0	0	FALSE	FALSE
magste_hs	InChI=1S/C21H42O4/c1-2-3-4-5-6-7-8-9-10-11-12-13-14-15-16-17-19(23)21(25)20(24)18-22/h20-22,24-25H,2-18H2,1H3	Organic compounds	Lipids and lipid-like molecules	Fatty Acyls	Fatty alcohols	Long-chain fatty alcohols		TRUE	FALSE	TRUE	FALSE	TRUE	FALSE	FALSE	0	1	0	FALSE	FALSE
5283363	InChI=1S/C13H24O/c1-2-3-4-5-6-7-8-9-10-11-12-13-14/h11-13H,2-10H2,1H3/b12-11+	Organic compounds	Lipids and lipid-like molecules	Fatty Acyls	Fatty aldehydes			FALSE	FALSE	FALSE	FALSE	TRUE	FALSE	FALSE	0	1	0	FALSE	FALSE
5283365	InChI=1S/C13H16O/c1-2-3-4-5-6-7-8-9-10-11-12-13-14/h11-13H,2-6H2,1H3/b12-11+	Organic compounds	Lipids and lipid-like molecules	Fatty Acyls	Fatty aldehydes			FALSE	FALSE	FALSE	TRUE	FALSE	TRUE	FALSE	2	0	0	FALSE	FALSE
5283381	InChI=1S/C18H34O/c1-2-3-4-5-6-7-8-9-10-11-12-13-14-15-16-17-18-19/h9-10,18H,2-8,11-17H2,1H3/b10-9+	Organic compounds	Lipids and lipid-like molecules	Fatty Acyls	Fatty aldehydes			FALSE	FALSE	FALSE	FALSE	TRUE	FALSE	FALSE	0	1	0	FALSE	FALSE
5283385	InChI=1S/C24H46O/c1-2-3-4-5-6-7-8-9-10-11-12-13-14-15-16-17-18-19-20-21-22-23-24-25/h9-10,24H,2-8,11-23H2,1H3/b10-9-	Organic compounds	Lipids and lipid-like molecules	Fatty Acyls	Fatty aldehydes			FALSE	FALSE	FALSE	FALSE	TRUE	FALSE	TRUE	0	2	0	FALSE	FALSE

2mpdhl	InChI=1S/C12H23NO2S2/c1-9(2)12(15)17-8-7-10(16)5-3-4-6-11(13)14/h9-10,16H,3-8H2,1-2H3,(H2,13,14)	Organic compounds	Lipids and lipid-like molecules	Fatty Acyls	Fatty amides		TRUE	FALSE	TRUE	FALSE	TRUE	FALSE	FALSE	0	1	0	FALSE	FALSE
FFAD 20:0		Organic compounds	Lipids and lipid-like molecules	Fatty Acyls	Fatty amides		FALSE	FALSE	FALSE	FALSE	TRUE	FALSE	FALSE	0	1	0	FALSE	FALSE
FFAD 20:1		Organic compounds	Lipids and lipid-like molecules	Fatty Acyls	Fatty amides		FALSE	FALSE	FALSE	FALSE	TRUE	FALSE	FALSE	0	1	0	FALSE	FALSE
FFAD 22:0		Organic compounds	Lipids and lipid-like molecules	Fatty Acyls	Fatty amides		FALSE	FALSE	FALSE	FALSE	TRUE	FALSE	FALSE	0	1	0	FALSE	FALSE
FFAD 22:1		Organic compounds	Lipids and lipid-like molecules	Fatty Acyls	Fatty amides		FALSE	FALSE	FALSE	FALSE	TRUE	FALSE	FALSE	0	1	0	FALSE	FALSE
168274	InChI=1S/C20H41NO4S/c1-2-3-4-5-6-7-8-9-10-11-12-13-14-15-16-17-20(22)21-18-19-26(23,24)25/h2-19H2,1H3,(H,21,22)(H,23,24,25)	Organic compounds	Lipids and lipid-like molecules	Fatty Acyls	Fatty amides	N-acyl amines	FALSE	FALSE	FALSE	TRUE	FALSE	FALSE	FALSE	1	0	0	FALSE	FALSE
52922081	InChI=1S/C20H37NO4S/c1-2-3-4-5-6-7-8-9-10-11-12-13-14-15-16-17-20(22)21-18-19-26(23,24)25/h6-7,9-10H,2-5,8,11-19H2,1H3,(H,21,22)(H,23,24,25)/b7-6-,10-9-	Organic compounds	Lipids and lipid-like molecules	Fatty Acyls	Fatty amides	N-acyl amines	FALSE	FALSE	FALSE	TRUE	FALSE	TRUE	FALSE	2	0	0	FALSE	FALSE
6437033	InChI=1S/C20H39NO4S/c1-2-3-4-5-6-7-8-9-10-11-12-13-14-15-16-17-20(22)21-18-19-26(23,24)25/h9-10H,2-8,11-19H2,1H3,(H,21,22)(H,23,24,25)/b10-9-	Organic compounds	Lipids and lipid-like molecules	Fatty Acyls	Fatty amides	N-acyl amines	FALSE	FALSE	FALSE	TRUE	FALSE	FALSE	FALSE	1	0	0	FALSE	FALSE
10469728	InChI=1S/C18H30O3/c1-2-3-11-14-17(19)15-12-9-7-5-4-6-8-10-13-16-18(20)21/h3,7,9,11-12,15,17,19H,2,4-6,8,10,13-14,16H2,1H3,(H,20,21)/b9-7-,11-3-,15-12+	Organic compounds	Lipids and lipid-like molecules	Fatty Acyls	Lineolic acids and derivatives		FALSE	FALSE	FALSE	TRUE	FALSE	FALSE	FALSE	1	0	0	FALSE	FALSE
14275409	InChI=1S/C39H66O5/c1-3-5-7-9-11-13-15-17-19-21-23-25-27-29-31-33-38(41)43-36-37(35-40)44-39(42)34-32-30-28-26-24-22-20-18-16-14-12-10-8-6-4-2/h11-14,17-20,24,26,37,40H,3-10,15-16,21-23,25,27-36H2,1-2H3/b13-11-,14-12-,19-17-,20-18-,26-24-/t37-/m0/s1	Organic compounds	Lipids and lipid-like molecules	Fatty Acyls	Lineolic acids and derivatives		FALSE	FALSE	FALSE	FALSE	TRUE	FALSE	FALSE	0	1	0	FALSE	FALSE
5282856	InChI=1S/C18H32O4/c1-2-3-4-5-6-8-11-14-17(22-21)15-12-9-7-10-13-16-18(19)20/h6,8,11,14,17,21H,2-5,7,9-10,12-13,15-16H2,1H3,(H,19,20)/b8-6+,14-11+	Organic compounds	Lipids and lipid-like molecules	Fatty Acyls	Lineolic acids and derivatives		FALSE	FALSE	FALSE	FALSE	TRUE	FALSE	FALSE	0	1	0	FALSE	FALSE
53480978	InChI=1S/C21H36O4/c1-2-3-4-5-6-7-8-9-10-11-12-13-14-15-16-17-21(24)25-19-20(23)18-22/h6-7,9-10,12-13,20,22-23H,2-5,8,11,14-19H2,1H3/b7-6-,10-9-,13-12-/t20-/m0/s1	Organic compounds	Lipids and lipid-like molecules	Fatty Acyls	Lineolic acids and derivatives		FALSE	FALSE	FALSE	FALSE	TRUE	FALSE	FALSE	0	1	0	FALSE	FALSE
6437847	InChI=1S/C18H32O4/c1-2-3-11-14-17(22-21)15-12-9-7-5-4-6-8-10-13-16-18(19)20/h7,9,12,15,17,21H,2-6,8,10-11,13-14,16H2,1H3,(H,19,20	Organic compounds	Lipids and lipid-like molecules	Fatty Acyls	Lineolic acids and derivatives		FALSE	FALSE	FALSE	FALSE	TRUE	FALSE	FALSE	0	1	0	FALSE	FALSE

	)/b9-7-,15-12+																	
lnlc	InChI=1S/C18H32O2/c1-2-3-4-5-6-7-8-9-10-11-12-13-14-15-16-17-18(19)20/h6-7,9-10H,2-5,8,11-17H2,1H3,(H,19,20)/p-1/b7-6-,10-9-	Organic compounds	Lipids and lipid-like molecules	Fatty Acyls	Lineolic acids and derivatives		TRUE	TRUE	TRUE	TRUE	TRUE	TRUE	TRUE	2	4	1	TRUE	FALSE
lnlnca	InChI=1S/C18H30O2/c1-2-3-4-5-6-7-8-9-10-11-12-13-14-15-16-17-18(19)20/h3-4,6-7,9-10H,2,5,8,11-17H2,1H3,(H,19,20)/p-1/b4-3-,7-6-,10-9-	Organic compounds	Lipids and lipid-like molecules	Fatty Acyls	Lineolic acids and derivatives		TRUE	FALSE	TRUE	TRUE	TRUE	FALSE	FALSE	1	1	1	FALSE	FALSE
strdnc	InChI=1S/C18H28O2/c1-2-3-4-5-6-7-8-9-10-11-12-13-14-15-16-17-18(19)20/h3-4,6-7,9-10,12-13H,2,5,8,11,14-17H2,1H3,(H,19,20)/p-1/b4-3?,7-6?,10-9-,13-12-	Organic compounds	Lipids and lipid-like molecules	Fatty Acyls	Lineolic acids and derivatives		TRUE	FALSE	TRUE	TRUE	FALSE	FALSE	FALSE	1	0	0	FALSE	FALSE
119250	InChI=1S/C18H34O3/c1-2-3-4-5-7-10-13-16-17(21-16)14-11-8-6-9-12-15-18(19)20/h16-17H,2-15H2,1H3,(H,19,20)/t16-,17+/m1/s1	Organic compounds	Lipids and lipid-like molecules	Fatty Acyls	Octadecanoids	Other Octadecanoids	FALSE	FALSE	FALSE	TRUE	FALSE	FALSE	FALSE	1	0	0	FALSE	FALSE
16061057	InChI=1S/C18H30O5/c1-2-3-10-13-16(19)14-11-8-6-4-5-7-9-12-15-17(23-22)18(20)21/h3,6,8,10,14,17,19,22H,2,4-5,7,9,11-13,15H2,1H3,(H,20,21)/b8-6-,10-3-,16-14+/t17-/m1/s1	Organic compounds	Lipids and lipid-like molecules	Fatty Acyls	Octadecanoids	Other Octadecanoids	FALSE	FALSE	FALSE	FALSE	TRUE	FALSE	TRUE	0	2	0	FALSE	FALSE
16061067	InChI=1S/C18H32O4/c1-2-3-10-13-16(19)17(20)14-11-8-6-4-5-7-9-12-15-18(21)22/h3,8,10-11,16-17,19-20H,2,4-7,9,12-15H2,1H3,(H,21,22)/b10-3-,11-8-	Organic compounds	Lipids and lipid-like molecules	Fatty Acyls	Octadecanoids	Other Octadecanoids	FALSE	FALSE	FALSE	TRUE	FALSE	FALSE	FALSE	1	0	0	FALSE	FALSE
6446027	InChI=1S/C18H30O3/c1-2-3-11-14-17(19)15-12-9-7-5-4-6-8-10-13-16-18(20)21/h7,9,12,15H,2-6,8,10-11,13-14,16H2,1H3,(H,20,21)/b9-7-,15-12+	Organic compounds	Lipids and lipid-like molecules	Fatty Acyls	Octadecanoids	Other Octadecanoids	FALSE	FALSE	FALSE	FALSE	TRUE	FALSE	FALSE	0	1	0	FALSE	FALSE
6450029	InChI=1S/C18H30O4/c1-2-3-4-5-6-8-11-14-17(22-21)15-12-9-7-10-13-16-18(19)20/h3-4,6,8,11,14,17,21H,2,5,7,9-10,12-13,15-16H2,1H3,(H,19,20)/b4-3-,8-6-,14-11+/t17-/m1/s1	Organic compounds	Lipids and lipid-like molecules	Fatty Acyls	Octadecanoids	Other Octadecanoids	FALSE	FALSE	FALSE	TRUE	FALSE	FALSE	FALSE	1	0	0	FALSE	FALSE
9570127	InChI=1S/C18H36O3/c1-2-3-4-5-6-8-11-14-17(19)15-12-9-7-10-13-16-18(20)21/h17,19H,2-16H2,1H3,(H,20,21)	Organic compounds	Lipids and lipid-like molecules	Fatty Acyls	Octadecanoids	Other Octadecanoids	FALSE	FALSE	FALSE	TRUE	FALSE	FALSE	FALSE	1	0	0	FALSE	FALSE
9858729	InChI=1S/C18H34O5/c1-2-3-7-11-16(20)17(21)14-13-15(19)10-8-5-4-6-9-12-18(22)23/h13-17,19-21H,2-12H2,1H3,(H,22,23)/b14-13+/t15-,16-,17-/m0/s1	Organic compounds	Lipids and lipid-like molecules	Fatty Acyls	Octadecanoids	Other Octadecanoids	FALSE	FALSE	FALSE	TRUE	FALSE	FALSE	FALSE	1	0	0	FALSE	FALSE
14275352	InChI=1S/C35H64O5/c1-3-5-7-9-11-13-15-16-17-18-20-22-24-26-28-30-35(38)40-33(31-36)32-39-34(37)29-27-25-23-21-19-14-12-10-8-6-4-2/h11,13,16-17,33	Organic compounds	Lipids and lipid-like molecules	Glycerolipids	Diradylglycerols	Diacylglycerols	FALSE	FALSE	FALSE	FALSE	TRUE	FALSE	FALSE	0	1	0	FALSE	FALSE

	,36H,3-10,12,14-15,18-32H2,1-2H3/b13-11-,17-16-/t33-/m0/s1																		
14275369	InChI=1S/C36H66O5/c1-3-5-7-9-11-13-15-17-18-19-21-23-25-27-29-31-36(39)41-34(32-37)33-40-35(38)30-28-26-24-22-20-16-14-12-10-8-6-4-2/h11,13,17-18,34,37H,3-10,12,14-16,19-33H2,1-2H3/b13-11-,18-17-/t34-/m0/s1	Organic compounds	Lipids and lipid-like molecules	Glycerolipids	Diradylglycerols	Diacylglycerols		FALSE	FALSE	FALSE	FALSE	TRUE	FALSE	TRUE	0	2	0	FALSE	FALSE
56936328	InChI=1S/C34H62O5/c1-3-5-7-9-11-13-15-16-17-18-19-21-23-25-27-29-34(37)39-32(30-35)31-38-33(36)28-26-24-22-20-14-12-10-8-6-4-2/h11,13,16-17,32,35H,3-10,12,14-15,18-31H2,1-2H3/b13-11-,17-16-/t32-/m0/s1	Organic compounds	Lipids and lipid-like molecules	Glycerolipids	Diradylglycerols	Diacylglycerols		FALSE	FALSE	FALSE	FALSE	TRUE	FALSE	FALSE	0	1	0	FALSE	FALSE
DG 36:2/18:0		Organic compounds	Lipids and lipid-like molecules	Glycerolipids	Diradylglycerols	Diacylglycerols		FALSE	FALSE	FALSE	FALSE	TRUE	FALSE	FALSE	0	1	0	FALSE	FALSE
124835	InChI=1S/C19H36O5/c1-3-4-5-6-7-8-9-10-11-12-13-14-19(22)23-16-18(15-20)24-17(2)21/h18,20H,3-16H2,1-2H3	Organic compounds	Lipids and lipid-like molecules	Glycerolipids	Diradylglycerols	Diacylglycerols	1,2-diacylglycerols	FALSE	FALSE	FALSE	TRUE	FALSE	FALSE	FALSE	1	0	0	FALSE	FALSE
53477952	InChI=1S/C35H66O5/c1-3-5-7-9-11-13-15-16-17-18-20-22-24-26-28-30-35(38)40-33(31-36)32-39-34(37)29-27-25-23-21-19-14-12-10-8-6-4-2/h13,15,33,36H,3-12,14,16-32H2,1-2H3/b15-13-/t33-/m0/s1	Organic compounds	Lipids and lipid-like molecules	Glycerolipids	Diradylglycerols	Diacylglycerols	1,2-diacylglycerols	FALSE	FALSE	FALSE	FALSE	TRUE	FALSE	TRUE	0	2	0	FALSE	FALSE
53477967	InChI=1S/C39H66O5/c1-3-5-7-9-11-13-15-16-17-18-19-20-21-22-24-26-28-30-32-34-39(42)44-37(35-40)36-43-38(41)33-31-29-27-25-23-14-12-10-8-6-4-2/h11,13,16-17,19-20,22,24,28,30,37,40H,3-10,12,14-15,18,21,23,25-27,29,31-36H2,1-2H3/b13-11-,17-16-,20-19-,24-22-,30-28-/t37-/m0/s1	Organic compounds	Lipids and lipid-like molecules	Glycerolipids	Diradylglycerols	Diacylglycerols	1,2-diacylglycerols	FALSE	FALSE	FALSE	FALSE	TRUE	FALSE	FALSE	0	1	0	FALSE	FALSE
53478003	InChI=1S/C34H64O5/c1-3-5-7-9-11-13-15-17-19-21-23-25-27-29-34(37)39-32(30-35)31-38-33(36)28-26-24-22-20-18-16-14-12-10-8-6-4-2/h13,15,32,35H,3-12,14,16-31H2,1-2H3/b15-13-/t32-/m0/s1	Organic compounds	Lipids and lipid-like molecules	Glycerolipids	Diradylglycerols	Diacylglycerols	1,2-diacylglycerols	FALSE	FALSE	FALSE	FALSE	TRUE	FALSE	FALSE	0	1	0	FALSE	FALSE
53478004	InChI=1S/C36H68O5/c1-3-5-7-9-11-13-15-17-18-19-21-23-25-27-29-31-36(39)41-34(32-37)33-40-35(38)30-28-26-24-22-20-16-14-12-10-8-6-4-2/h13,15,34,37H,3-12,14,16-33H2,1-2H3/b15-13-/t34-/m0/s1	Organic compounds	Lipids and lipid-like molecules	Glycerolipids	Diradylglycerols	Diacylglycerols	1,2-diacylglycerols	FALSE	FALSE	FALSE	FALSE	TRUE	FALSE	TRUE	0	2	0	FALSE	FALSE
53478568	InChI=1S/C51H100O5/c1-3-5-7-9-11-13-15-17-19-21-23-25-27-29-31-33-35-37-39-41-43-45-50(53)55-48-49(47-52)56-	Organic compounds	Lipids and lipid-like molecules	Glycerolipids	Diradylglycerols	Diacylglycerols	1,2-diacylglycerols	FALSE	FALSE	FALSE	TRUE	FALSE	FALSE	FALSE	1	0	0	FALSE	FALSE

	51(54)46-44-42-40-38-36-34-32-30-28-26-24-22-20-18-16-14-12-10-8-6-4-2/h49,52H,3-48H2,1-2H3/t49-/m0/s1																		
MG(18:2)		Organic compounds	Lipids and lipid-like molecules	Glycerolipids	Monoradylglycerols	Monoacylglycerols		FALSE	FALSE	FALSE	TRUE	FALSE	FALSE	FALSE	1	0	0	FALSE	FALSE
14900	InChI=1S/C19H38O4/c1-2-3-4-5-6-7-8-9-10-11-12-13-14-15-19(22)23-17-18(21)16-20/h18,20-21H,2-17H2,1H3	Organic compounds	Lipids and lipid-like molecules	Glycerolipids	Monoradylglycerols	Monoacylglycerols	1-monoacylglycerols	FALSE	FALSE	FALSE	FALSE	TRUE	FALSE	FALSE	0	1	0	FALSE	FALSE
71598538	InChI=1S/C15H30O4/c1-2-3-4-5-6-7-8-9-10-11-15(18)19-13-14(17)12-16/h14,16-17H,2-13H2,1H3/t14-/m1/s1	Organic compounds	Lipids and lipid-like molecules	Glycerolipids	Monoradylglycerols	Monoacylglycerols	1-monoacylglycerols	FALSE	FALSE	FALSE	TRUE	FALSE	FALSE	FALSE	1	0	0	FALSE	FALSE
magole_hs	InChI=1S/C21H40O4/c1-2-3-4-5-6-7-8-9-10-11-12-13-14-15-16-17-21(24)25-19-20(23)18-22/h9-10,20,22-23H,2-8,11-19H2,1H3/b10-9-	Organic compounds	Lipids and lipid-like molecules	Glycerolipids	Monoradylglycerols	Monoacylglycerols	1-monoacylglycerols	TRUE	FALSE	TRUE	FALSE	TRUE	FALSE	FALSE	0	1	0	FALSE	FALSE
53480969	InChI=1S/C25H42O4/c1-2-3-4-5-6-7-8-9-10-11-12-13-14-15-16-17-18-19-20-21-25(28)29-24(22-26)23-27/h6-7,9-10,12-13,15-16,24,26-27H,2-5,8,11,14,17-23H2,1H3/b7-6-,10-9-,13-12-,16-15-	Organic compounds	Lipids and lipid-like molecules	Glycerolipids	Monoradylglycerols	Monoacylglycerols	2-monoacylglycerols	FALSE	FALSE	FALSE	TRUE	FALSE	FALSE	FALSE	1	0	0	FALSE	FALSE
TG 52:2/18:0		Organic compounds	Lipids and lipid-like molecules	Glycerolipids	Triradylglycerols			FALSE	FALSE	FALSE	FALSE	TRUE	FALSE	FALSE	0	1	0	FALSE	FALSE
TG 52:3/18:1		Organic compounds	Lipids and lipid-like molecules	Glycerolipids	Triradylglycerols			FALSE	FALSE	FALSE	FALSE	TRUE	FALSE	FALSE	0	1	0	FALSE	FALSE
TG 54:3/18:0		Organic compounds	Lipids and lipid-like molecules	Glycerolipids	Triradylglycerols			FALSE	FALSE	FALSE	FALSE	TRUE	FALSE	FALSE	0	1	0	FALSE	FALSE
TG 54:4/18:1		Organic compounds	Lipids and lipid-like molecules	Glycerolipids	Triradylglycerols			FALSE	FALSE	FALSE	FALSE	TRUE	FALSE	FALSE	0	1	0	FALSE	FALSE
TG 54:4/20:4		Organic compounds	Lipids and lipid-like molecules	Glycerolipids	Triradylglycerols			FALSE	FALSE	FALSE	FALSE	TRUE	FALSE	FALSE	0	1	0	FALSE	FALSE
TG 54:5/18:1		Organic compounds	Lipids and lipid-like molecules	Glycerolipids	Triradylglycerols			FALSE	FALSE	FALSE	FALSE	TRUE	FALSE	FALSE	0	1	0	FALSE	FALSE
TG 54:5/20:4		Organic compounds	Lipids and lipid-like molecules	Glycerolipids	Triradylglycerols			FALSE	FALSE	FALSE	FALSE	TRUE	FALSE	FALSE	0	1	0	FALSE	FALSE
TG 54:6/18:1		Organic compounds	Lipids and lipid-like molecules	Glycerolipids	Triradylglycerols			FALSE	FALSE	FALSE	FALSE	TRUE	FALSE	FALSE	0	1	0	FALSE	FALSE
TG 54:6/20:4		Organic compounds	Lipids and lipid-like molecules	Glycerolipids	Triradylglycerols			FALSE	FALSE	FALSE	FALSE	TRUE	FALSE	FALSE	0	1	0	FALSE	FALSE
TG 54:7/18:1		Organic compounds	Lipids and lipid-like molecules	Glycerolipids	Triradylglycerols			FALSE	FALSE	FALSE	FALSE	TRUE	FALSE	FALSE	0	1	0	FALSE	FALSE
TG 56:4/20:4		Organic compounds	Lipids and lipid-like molecules	Glycerolipids	Triradylglycerols			FALSE	FALSE	FALSE	FALSE	TRUE	FALSE	FALSE	0	1	0	FALSE	FALSE
TG 56:5/18:1		Organic compounds	Lipids and lipid-like molecules	Glycerolipids	Triradylglycerols			FALSE	FALSE	FALSE	FALSE	TRUE	FALSE	FALSE	0	1	0	FALSE	FALSE
TG 56:5/20:4		Organic compounds	Lipids and lipid-like molecules	Glycerolipids	Triradylglycerols			FALSE	FALSE	FALSE	FALSE	TRUE	FALSE	FALSE	0	1	0	FALSE	FALSE
TG 56:6/20:4		Organic compounds	Lipids and lipid-like molecules	Glycerolipids	Triradylglycerols			FALSE	FALSE	FALSE	FALSE	TRUE	FALSE	FALSE	0	1	0	FALSE	FALSE
TG 56:7/20:4		Organic compounds	Lipids and lipid-like molecules	Glycerolipids	Triradylglycerols			FALSE	FALSE	FALSE	FALSE	TRUE	FALSE	FALSE	0	1	0	FALSE	FALSE
TG 56:8/20:4		Organic compounds	Lipids and lipid-like molecules	Glycerolipids	Triradylglycerols			FALSE	FALSE	FALSE	FALSE	TRUE	FALSE	FALSE	0	1	0	FALSE	FALSE

TG 56:9/20:4		Organic compounds	Lipids and lipid-like molecules	Glycerolipids	Triradylglycerols		FALSE	FALSE	FALSE	FALSE	TRUE	FALSE	FALSE	0	1	0	FALSE	FALSE
TG 58:7/20:4		Organic compounds	Lipids and lipid-like molecules	Glycerolipids	Triradylglycerols		FALSE	FALSE	FALSE	FALSE	TRUE	FALSE	FALSE	0	1	0	FALSE	FALSE
131757896	InChI=1S/C60H118O5/c1-4-7-10-13-16-19-22-25-27-29-30-31-32-33-34-36-39-41-44-47-50-53-59(61)64-57-58(65-60(62)54-51-48-45-42-38-24-21-18-15-12-9-6-3)56-63-55-52-49-46-43-40-37-35-28-26-23-20-17-14-11-8-5-2/h58H,4-57H2,1-3H3/t58-/m1/s1	Organic compounds	Lipids and lipid-like molecules	Glycerolipids	Triradylglycerols	Alkyldiacylglycerols	FALSE	FALSE	FALSE	TRUE	FALSE	FALSE	FALSE	1	0	0	FALSE	FALSE
131762464	InChI=1S/C65H122O5/c1-4-7-10-13-16-19-22-25-28-31-33-35-37-40-43-46-49-52-55-58-64(66)69-62-63(61-68-60-57-54-51-48-45-42-39-30-27-24-21-18-15-12-9-6-3)70-65(67)59-56-53-50-47-44-41-38-36-34-32-29-26-23-20-17-14-11-8-5-2/h17,20,25-26,28-29,63H,4-16,18-19,21-24,27,30-62H2,1-3H3/b20-17-,28-25-,29-26-/t63-/m1/s1	Organic compounds	Lipids and lipid-like molecules	Glycerolipids	Triradylglycerols	Alkyldiacylglycerols	FALSE	FALSE	FALSE	TRUE	FALSE	FALSE	FALSE	1	0	0	FALSE	FALSE
131758566	InChI=1S/C45H82O6/c1-4-7-10-13-16-19-22-25-28-31-34-37-43(46)49-40-42(51-45(48)39-36-33-30-27-24-21-18-15-12-9-6-3)41-50-44(47)38-35-32-29-26-23-20-17-14-11-8-5-2/h13-14,16-17,42H,4-12,15,18-41H2,1-3H3/b16-13-,17-14-	Organic compounds	Lipids and lipid-like molecules	Glycerolipids	Triradylglycerols	Triacylglycerols	FALSE	FALSE	FALSE	TRUE	FALSE	FALSE	FALSE	1	0	0	FALSE	FALSE
131763034	InChI=1S/C65H106O6/c1-4-7-10-13-16-19-22-25-28-30-31-32-33-35-37-40-43-46-49-52-55-58-64(67)70-61-62(60-69-63(66)57-54-51-48-45-42-39-36-27-24-21-18-15-12-9-6-3)71-65(68)59-56-53-50-47-44-41-38-34-29-26-23-20-17-14-11-8-5-2/h8-9,11-12,17-18,20-21,25-29,36,38,41-42,45,47,50,62H,4-7,10,13-16,19,22-24,30-35,37,39-40,43-44,46,48-49,51-61H2,1-3H3/b11-8-,12-9-,20-17-,21-18-,28-25-,29-26-,36-27-,41-38-,45-42-,50-47-/t62-/m1/s1	Organic compounds	Lipids and lipid-like molecules	Glycerolipids	Triradylglycerols	Triacylglycerols	FALSE	FALSE	FALSE	TRUE	FALSE	FALSE	FALSE	1	0	0	FALSE	FALSE
131766033	InChI=1S/C59H88O6/c1-4-7-10-13-16-19-22-25-28-29-32-35-38-41-44-47-50-53-59(62)65-56(54-63-57(60)51-48-45-42-39-36-33-30-26-23-20-17-14-11-8-5-2)55-64-58(61)52-49-46-43-40-37-34-31-27-24-21-18-15-12-9-6-3/h7-12,16-21,25-28,30-32,35-37,39-41,44,56H,4-6,13-15,22-24,29,33-34,38,42-43,45-55H2,1-3H3/b10-7-,11-8-,12-9-,19-16-,20-17-,21	Organic compounds	Lipids and lipid-like molecules	Glycerolipids	Triradylglycerols	Triacylglycerols	FALSE	FALSE	FALSE	TRUE	FALSE	FALSE	FALSE	1	0	0	FALSE	FALSE

	-18-,28-25-,30-26-,31-27-,35-32-,39-36-,40-37-,44-41-																			
53480526	InChI=1S/C53H94O6/c1-4-7-10-13-16-19-22-24-26-28-31-33-36-39-42-45-51(54)57-48-50(59-53(56)47-44-41-38-35-30-21-18-15-12-9-6-3)49-58-52(55)46-43-40-37-34-32-29-27-25-23-20-17-14-11-8-5-2/h16-17,19-20,24-27,50H,4-15,18,21-23,28-49H2,1-3H3/b19-16-,20-17-,26-24-,27-25-	Organic compounds	Lipids and lipid-like molecules	Glycerolipids	Triradylcglycerols	Triacylglycerols			FALSE	FALSE	FALSE	TRUE	FALSE	FALSE	FALSE	1	0	0	FALSE	FALSE
56937262	InChI=1S/C53H92O6/c1-4-7-10-13-16-19-21-23-25-26-28-29-31-34-37-40-43-46-52(55)58-49-50(48-57-51(54)45-42-39-36-33-18-15-12-9-6-3)59-53(56)47-44-41-38-35-32-30-27-24-22-20-17-14-11-8-5-2/h16,19,23-25,27-29,34,37,50H,4-15,17-18,20-22,26,30-33,35-36,38-49H2,1-3H3/b19-16-,25-23-,27-24-,29-28-,37-34-/t50-/m1/s1	Organic compounds	Lipids and lipid-like molecules	Glycerolipids	Triradylcglycerols	Triacylglycerols			FALSE	FALSE	FALSE	FALSE	TRUE	FALSE	FALSE	0	1	0	FALSE	FALSE
TG C59:5		Organic compounds	Lipids and lipid-like molecules	Glycerolipids	Triradylcglycerols	Triacylglycerols			FALSE	FALSE	FALSE	FALSE	TRUE	FALSE	FALSE	0	1	0	FALSE	FALSE
pchol2ste_hs	InChI=1S/C26H55NO7P/c1-5-6-7-8-9-10-11-12-13-14-15-16-17-18-19-20-26(29)32-23-25(28)24-34-35(30,31)33-22-21-27(2,3)4/h25,28H,5-24H2,1-4H3,(H,30,31)	Organic compounds	Lipids and lipid-like molecules	Glycerophospholipids					TRUE	FALSE	TRUE	FALSE	TRUE	FALSE	FALSE	0	1	0	FALSE	TRUE
glyc2p	InChI=1S/C3H9O6P/c4-1-3(2-5)9-10(6,7)8/h3-5H,1-2H2,(H2,6,7,8)/p-2	Organic compounds	Lipids and lipid-like molecules	Glycerophospholipids	Glycerophosphates				TRUE	FALSE	TRUE	FALSE	TRUE	FALSE	FALSE	0	1	0	FALSE	FALSE
glyc3p	InChI=1S/C3H9O6P/c4-1-3(5)2-9-10(6,7)8/h3-5H,1-2H2,(H2,6,7,8)/p-2/t3-/m1/s1	Organic compounds	Lipids and lipid-like molecules	Glycerophospholipids	Glycerophosphates				TRUE	FALSE	TRUE	TRUE	TRUE	FALSE	FALSE	1	1	1	FALSE	FALSE
52929670	InChI=1S/C37H65O7P/c1-3-5-7-9-11-13-15-17-19-20-22-24-26-28-30-32-37(38)44-36(35-43-45(39,40)41)34-42-33-31-29-27-25-23-21-18-16-14-12-10-8-6-4-2/h5,7,11,13,17,19,22,24,31,33,36H,3-4,6,8-10,12,14-16,18,20-21,23,25-30,32,34-35H2,1-2H3,(H2,39,40,41)/b7-5-,13-11-,19-17-,24-22-,33-31-/t36-/m1/s1	Organic compounds	Lipids and lipid-like molecules	Glycerophospholipids	Glycerophosphates	1-(1Z-alkenyl),2-acyl-glycerophosphates			FALSE	FALSE	FALSE	FALSE	TRUE	FALSE	TRUE	0	2	0	FALSE	FALSE
52928935	InChI=1S/C36H67O8P/c1-3-5-7-9-11-13-15-17-18-19-21-22-24-26-28-30-35(37)42-32-34(33-43-45(39,40)41)44-36(38)31-29-27-25-23-20-16-14-12-10-8-6-4-2/h11,13,17-18,34H,3-10,12,14-16,19-33H2,1-2H3,(H2,39,40,41)/b13-11-,18-17-/t34-/m1/s1	Organic compounds	Lipids and lipid-like molecules	Glycerophospholipids	Glycerophosphates	Diacylglycerophosphates	1,2-diacylglycerol-3-phosphates		FALSE	FALSE	FALSE	TRUE	FALSE	FALSE	FALSE	1	0	0	FALSE	FALSE
44575059	InChI=1S/C22H45O7P/c1-2-3-4-5-6-7-8-9-10-11-12-13-14-15-16-17-18-22(24)28-19-21(23)20-29-3	Organic compounds	Lipids and lipid-like molecules	Glycerophospholipids	Glycerophosphates	Monoacylglycerophosphates	Lysophosphatidic acids	1-acylglycerol-3-phosphates	FALSE	FALSE	FALSE	TRUE	FALSE	FALSE	FALSE	1	0	0	FALSE	FALSE

	0(25,26)27/h21,23H,2-20H2,1H3,(H2,25,26,27)/t21-/m1/s1																			
6419701	InChI=1S/C19H39O7P/c1-2-3-4-5-6-7-8-9-10-11-12-13-14-15-19(21)25-16-18(20)17-26-27(22,23)24/h18,20H,2-17H2,1H3,(H2,22,23,24)/t18-/m1/s1	Organic compounds	Lipids and lipid-like molecules	Glycerophospholipids	Glycerophosphates	Monoacylglycerophosphates	Lysophosphatidic acids	1-acylglycerol-3-phosphates	FALSE	FALSE	FALSE	TRUE	FALSE	FALSE	FALSE	1	0	0	FALSE	FALSE
LPC 18:0		Organic compounds	Lipids and lipid-like molecules	Glycerophospholipids	Glycerophosphocholines				FALSE	FALSE	FALSE	FALSE	TRUE	FALSE	FALSE	0	1	0	FALSE	FALSE
LPC 20:2		Organic compounds	Lipids and lipid-like molecules	Glycerophospholipids	Glycerophosphocholines				FALSE	FALSE	FALSE	FALSE	TRUE	FALSE	FALSE	0	1	0	FALSE	FALSE
LPC 22:0		Organic compounds	Lipids and lipid-like molecules	Glycerophospholipids	Glycerophosphocholines				FALSE	FALSE	FALSE	FALSE	TRUE	FALSE	FALSE	0	1	0	FALSE	FALSE
LPC(16:1)		Organic compounds	Lipids and lipid-like molecules	Glycerophospholipids	Glycerophosphocholines				FALSE	FALSE	FALSE	TRUE	FALSE	FALSE	FALSE	1	0	0	FALSE	FALSE
LPC(17:1)		Organic compounds	Lipids and lipid-like molecules	Glycerophospholipids	Glycerophosphocholines				FALSE	FALSE	FALSE	TRUE	FALSE	FALSE	FALSE	1	0	0	FALSE	FALSE
LPC(22:6)		Organic compounds	Lipids and lipid-like molecules	Glycerophospholipids	Glycerophosphocholines				FALSE	FALSE	FALSE	TRUE	FALSE	FALSE	FALSE	1	0	0	FALSE	FALSE
LPC(P-16:0)		Organic compounds	Lipids and lipid-like molecules	Glycerophospholipids	Glycerophosphocholines				FALSE	FALSE	FALSE	FALSE	TRUE	FALSE	FALSE	0	1	0	FALSE	FALSE
LPC(P-18:0)		Organic compounds	Lipids and lipid-like molecules	Glycerophospholipids	Glycerophosphocholines				FALSE	FALSE	FALSE	TRUE	TRUE	FALSE	FALSE	1	1	1	FALSE	FALSE
PC aa C34:4		Organic compounds	Lipids and lipid-like molecules	Glycerophospholipids	Glycerophosphocholines				FALSE	FALSE	FALSE	TRUE	FALSE	FALSE	FALSE	1	0	0	FALSE	FALSE
PC aa C35:1_P		Organic compounds	Lipids and lipid-like molecules	Glycerophospholipids	Glycerophosphocholines				FALSE	FALSE	FALSE	TRUE	FALSE	FALSE	FALSE	1	0	0	FALSE	FALSE
PC aa C36:0		Organic compounds	Lipids and lipid-like molecules	Glycerophospholipids	Glycerophosphocholines				FALSE	FALSE	FALSE	FALSE	TRUE	FALSE	FALSE	0	1	0	FALSE	FALSE
PC aa C36:1		Organic compounds	Lipids and lipid-like molecules	Glycerophospholipids	Glycerophosphocholines				FALSE	FALSE	FALSE	FALSE	TRUE	FALSE	FALSE	0	1	0	FALSE	FALSE
PC aa C37:2_P		Organic compounds	Lipids and lipid-like molecules	Glycerophospholipids	Glycerophosphocholines				FALSE	FALSE	FALSE	TRUE	FALSE	FALSE	FALSE	1	0	0	FALSE	FALSE
PC aa C38:1		Organic compounds	Lipids and lipid-like molecules	Glycerophospholipids	Glycerophosphocholines				FALSE	FALSE	FALSE	FALSE	TRUE	FALSE	TRUE	0	2	0	FALSE	FALSE
PC aa C38:7		Organic compounds	Lipids and lipid-like molecules	Glycerophospholipids	Glycerophosphocholines				FALSE	FALSE	FALSE	FALSE	TRUE	FALSE	FALSE	0	1	0	FALSE	FALSE
PC aa C40:2		Organic compounds	Lipids and lipid-like molecules	Glycerophospholipids	Glycerophosphocholines				FALSE	FALSE	FALSE	FALSE	TRUE	FALSE	FALSE	0	1	0	FALSE	FALSE
PC aa C40:3		Organic compounds	Lipids and lipid-like molecules	Glycerophospholipids	Glycerophosphocholines				FALSE	FALSE	FALSE	FALSE	TRUE	FALSE	FALSE	0	1	0	FALSE	FALSE
PC aa C42:2		Organic compounds	Lipids and lipid-like molecules	Glycerophospholipids	Glycerophosphocholines				FALSE	FALSE	FALSE	FALSE	TRUE	FALSE	FALSE	0	1	0	FALSE	FALSE
PC ae C30:2		Organic compounds	Lipids and lipid-like molecules	Glycerophospholipids	Glycerophosphocholines				FALSE	FALSE	FALSE	FALSE	TRUE	FALSE	FALSE	0	1	0	FALSE	FALSE
PC ae C34:3		Organic compounds	Lipids and lipid-like molecules	Glycerophospholipids	Glycerophosphocholines				FALSE	FALSE	FALSE	FALSE	TRUE	FALSE	FALSE	0	1	0	FALSE	FALSE
PC ae C36:0		Organic compounds	Lipids and lipid-like molecules	Glycerophospholipids	Glycerophosphocholines				FALSE	FALSE	FALSE	FALSE	TRUE	FALSE	FALSE	0	1	0	FALSE	FALSE
PC ae C36:3		Organic compounds	Lipids and lipid-like molecules	Glycerophospholipids	Glycerophosphocholines				FALSE	FALSE	FALSE	FALSE	TRUE	FALSE	FALSE	0	1	0	FALSE	FALSE
PC ae C36:4		Organic compounds	Lipids and lipid-like molecules	Glycerophospholipids	Glycerophosphocholines				FALSE	FALSE	FALSE	FALSE	TRUE	FALSE	FALSE	0	1	0	FALSE	FALSE
PC ae C38:1		Organic compounds	Lipids and lipid-like molecules	Glycerophospholipids	Glycerophosphocholines				FALSE	FALSE	FALSE	FALSE	TRUE	FALSE	FALSE	0	1	0	FALSE	FALSE
PC ae C38:2		Organic compounds	Lipids and lipid-like molecules	Glycerophospholipids	Glycerophosphocholines				FALSE	FALSE	FALSE	FALSE	TRUE	FALSE	FALSE	0	1	0	FALSE	FALSE

PC ae C38:4		Organic compounds	Lipids and lipid-like molecules	Glycerophospholipids	Glycerophosphocholines		FALSE	FALSE	FALSE	FALSE	TRUE	FALSE	FALSE	0	1	0	FALSE	FALSE
PC ae C38:5		Organic compounds	Lipids and lipid-like molecules	Glycerophospholipids	Glycerophosphocholines		FALSE	FALSE	FALSE	FALSE	TRUE	FALSE	FALSE	0	1	0	FALSE	FALSE
PC ae C40:1		Organic compounds	Lipids and lipid-like molecules	Glycerophospholipids	Glycerophosphocholines		FALSE	FALSE	FALSE	TRUE	FALSE	FALSE	FALSE	1	0	0	FALSE	FALSE
PC ae C40:2		Organic compounds	Lipids and lipid-like molecules	Glycerophospholipids	Glycerophosphocholines		FALSE	FALSE	FALSE	FALSE	TRUE	FALSE	FALSE	0	1	0	FALSE	FALSE
PC ae C40:3		Organic compounds	Lipids and lipid-like molecules	Glycerophospholipids	Glycerophosphocholines		FALSE	FALSE	FALSE	FALSE	TRUE	FALSE	FALSE	0	1	0	FALSE	FALSE
PC ae C42:1		Organic compounds	Lipids and lipid-like molecules	Glycerophospholipids	Glycerophosphocholines		FALSE	FALSE	FALSE	TRUE	FALSE	FALSE	FALSE	1	0	0	FALSE	FALSE
PC ae C42:3		Organic compounds	Lipids and lipid-like molecules	Glycerophospholipids	Glycerophosphocholines		FALSE	FALSE	FALSE	FALSE	TRUE	FALSE	FALSE	0	1	0	FALSE	FALSE
PC ae C42:4		Organic compounds	Lipids and lipid-like molecules	Glycerophospholipids	Glycerophosphocholines		FALSE	FALSE	FALSE	FALSE	TRUE	FALSE	FALSE	0	1	0	FALSE	FALSE
PCe 34:2		Organic compounds	Lipids and lipid-like molecules	Glycerophospholipids	Glycerophosphocholines		FALSE	FALSE	FALSE	FALSE	TRUE	FALSE	FALSE	0	1	0	FALSE	FALSE
PCe 36:0		Organic compounds	Lipids and lipid-like molecules	Glycerophospholipids	Glycerophosphocholines		FALSE	FALSE	FALSE	FALSE	TRUE	FALSE	FALSE	0	1	0	FALSE	FALSE
PCe 36:1		Organic compounds	Lipids and lipid-like molecules	Glycerophospholipids	Glycerophosphocholines		FALSE	FALSE	FALSE	FALSE	TRUE	FALSE	FALSE	0	1	0	FALSE	FALSE
PCe 36:3		Organic compounds	Lipids and lipid-like molecules	Glycerophospholipids	Glycerophosphocholines		FALSE	FALSE	FALSE	FALSE	TRUE	FALSE	FALSE	0	1	0	FALSE	FALSE
PCe 36:4		Organic compounds	Lipids and lipid-like molecules	Glycerophospholipids	Glycerophosphocholines		FALSE	FALSE	FALSE	FALSE	TRUE	FALSE	FALSE	0	1	0	FALSE	FALSE
PCe 36:5		Organic compounds	Lipids and lipid-like molecules	Glycerophospholipids	Glycerophosphocholines		FALSE	FALSE	FALSE	FALSE	TRUE	FALSE	FALSE	0	1	0	FALSE	FALSE
PCe 38:1		Organic compounds	Lipids and lipid-like molecules	Glycerophospholipids	Glycerophosphocholines		FALSE	FALSE	FALSE	FALSE	TRUE	FALSE	FALSE	0	1	0	FALSE	FALSE
PCe 38:2		Organic compounds	Lipids and lipid-like molecules	Glycerophospholipids	Glycerophosphocholines		FALSE	FALSE	FALSE	FALSE	TRUE	FALSE	FALSE	0	1	0	FALSE	FALSE
PCe 38:6		Organic compounds	Lipids and lipid-like molecules	Glycerophospholipids	Glycerophosphocholines		FALSE	FALSE	FALSE	FALSE	TRUE	FALSE	FALSE	0	1	0	FALSE	FALSE
PCe 46:3		Organic compounds	Lipids and lipid-like molecules	Glycerophospholipids	Glycerophosphocholines		FALSE	FALSE	FALSE	FALSE	TRUE	FALSE	FALSE	0	1	0	FALSE	FALSE
PCp 36:4		Organic compounds	Lipids and lipid-like molecules	Glycerophospholipids	Glycerophosphocholines		FALSE	FALSE	FALSE	FALSE	TRUE	FALSE	FALSE	0	1	0	FALSE	FALSE
PCp 38:4		Organic compounds	Lipids and lipid-like molecules	Glycerophospholipids	Glycerophosphocholines		FALSE	FALSE	FALSE	FALSE	TRUE	FALSE	FALSE	0	1	0	FALSE	FALSE
Phospholipids fragment		Organic compounds	Lipids and lipid-like molecules	Glycerophospholipids	Glycerophosphocholines		FALSE	FALSE	FALSE	FALSE	TRUE	FALSE	FALSE	0	1	0	FALSE	FALSE
g3pc	InChI=1S/C8H20NO6P/c1-9(2,3)4-5-14-16(12,13)15-7-8(11)6-10/h8,10-11H,4-7H2,1-3H3/t8-/m1/s1	Organic compounds	Lipids and lipid-like molecules	Glycerophospholipids	Glycerophosphocholines		TRUE	FALSE	TRUE	FALSE	TRUE	FALSE	TRUE	0	2	0	FALSE	FALSE
52923834	InChI=1S/C46H88NO7P/c1-6-8-10-12-14-16-18-20-22-23-24-26-28-30-32-34-36-38-41-51-43-45(44-53-55(49,50)52-42-40-47(3,4)5)54-46(48)39-37-35-33-31-29-27-25-21-19-17-15-13-11-9-7-2/h15,17,21,25,29,31,45H,6-14,16,18-20,22-24,26-28,30,32-44H2,1-5H3/b17-15-,25-21-,31-29-/t45-/m1/s1	Organic compounds	Lipids and lipid-like molecules	Glycerophospholipids	Glycerophosphocholines	1-alkyl,2-acyl glycero-3-phosphocholines	FALSE	FALSE	FALSE	FALSE	TRUE	FALSE	FALSE	0	1	0	FALSE	FALSE
53481711	InChI=1S/C44H88NO7P/c1-6-8-10-12-14-16-18-20-22-23-2	Organic compounds	Lipids and lipid-like molecules	Glycerophospholipids	Glycerophosphocholines	1-alkyl,2-acyl glycero-3-phosphocholine	FALSE	FALSE	FALSE	FALSE	TRUE	FALSE	FALSE	0	1	0	FALSE	FALSE

	4-25-27-29-31-33-35-37-44(46)52-43(42-51-53(47,48)50-40-38-45(3,4)5)41-49-39-36-34-32-30-28-26-21-19-17-15-13-11-9-7-2/h17,19,43H,6-16,18,20-42H2,1-5H3/b19-17-/t43-/m1/s1					s												
53481727	InChI=1S/C46H92NO7P/c1-6-8-10-12-14-16-18-20-22-24-25-27-29-31-33-35-37-39-46(48)54-45(44-53-55(49,50)52-42-40-47(3,4)5)43-51-41-38-36-34-32-30-28-26-23-21-19-17-15-13-11-9-7-2/h21,23,45H,6-20,22,24-44H2,1-5H3/b23-21-/t45-/m1/s1	Organic compounds	Lipids and lipid-like molecules	Glycerophospholipids	Glycerophosphocholines	1-alkyl,2-acylglycero-3-phosphocholines	FALSE	FALSE	FALSE	FALSE	TRUE	FALSE	FALSE	0	1	0	FALSE	FALSE
53481745	InChI=1S/C48H92NO7P/c1-6-8-10-12-14-16-18-20-22-23-24-25-26-28-30-32-34-36-38-40-43-53-45-47(46-55-57(51,52)54-44-42-49(3,4)5)56-48(50)41-39-37-35-33-31-29-27-21-19-17-15-13-11-9-7-2/h15,17,21,27,31,33,47H,6-14,16,18-20,22-26,28-30,32,34-46H2,1-5H3/b17-15-,27-21-,33-31-/t47-/m1/s1	Organic compounds	Lipids and lipid-like molecules	Glycerophospholipids	Glycerophosphocholines	1-alkyl,2-acylglycero-3-phosphocholines	FALSE	FALSE	FALSE	FALSE	TRUE	FALSE	FALSE	0	1	0	FALSE	FALSE
53481767	InChI=1S/C52H96NO7P/c1-6-8-10-12-14-16-18-20-22-24-26-28-30-32-34-36-38-40-42-44-47-57-49-51(50-59-61(55,56)58-48-46-53(3,4)5)60-52(54)45-43-41-39-37-35-33-31-29-27-25-23-21-19-17-15-13-11-9-7-2/h14-17,20-23,27,29,51H,6-13,18-19,24-26,28,30-50H2,1-5H3/b16-14-,17-15-,22-20-,23-21-,29-27-/t51-/m1/s1	Organic compounds	Lipids and lipid-like molecules	Glycerophospholipids	Glycerophosphocholines	1-alkyl,2-acylglycero-3-phosphocholines	FALSE	FALSE	FALSE	TRUE	FALSE	FALSE	FALSE	1	0	0	FALSE	FALSE
6443070	InChI=1S/C44H86NO7P/c1-6-8-10-12-14-16-18-20-22-24-26-28-30-32-34-36-39-49-41-43(42-51-53(47,48)50-40-38-45(3,4)5)52-44(46)37-35-33-31-29-27-25-23-21-19-17-15-13-11-9-7-2/h15,17,21,23,43H,6-14,16,18-20,22,24-42H2,1-5H3/b17-15-,23-21-/t43-/m1/s1	Organic compounds	Lipids and lipid-like molecules	Glycerophospholipids	Glycerophosphocholines	1-alkyl,2-acylglycero-3-phosphocholines	FALSE	FALSE	FALSE	FALSE	TRUE	FALSE	FALSE	0	1	0	FALSE	FALSE
PC ae C35:2_P		Organic compounds	Lipids and lipid-like molecules	Glycerophospholipids	Glycerophosphocholines	1-alkyl,2-acylglycero-3-phosphocholines	FALSE	FALSE	FALSE	FALSE	TRUE	FALSE	FALSE	0	1	0	FALSE	FALSE
PE ae C36:2_N		Organic compounds	Lipids and lipid-like molecules	Glycerophospholipids	Glycerophosphocholines	1-alkyl,2-acylglycero-3-phosphocholines	FALSE	FALSE	FALSE	TRUE	FALSE	FALSE	FALSE	1	0	0	FALSE	FALSE
PG ae C33:2_N		Organic compounds	Lipids and lipid-like molecules	Glycerophospholipids	Glycerophosphocholines	1-alkyl,2-acylglycero-3-phosphocholines	FALSE	FALSE	FALSE	FALSE	TRUE	FALSE	FALSE	0	1	0	FALSE	FALSE
24778638	InChI=1S/C44H88NO8P/c1-6-8-10-12-14-16-18-19-20-21-22-23-24-25-27-29-31-33-35-37-44(47)53-42(41-52-54(48,49)51-39-38-45(3,4)5	Organic compounds	Lipids and lipid-like molecules	Glycerophospholipids	Glycerophosphocholines	Diacylglycerophosphocholines	FALSE	FALSE	FALSE	FALSE	TRUE	FALSE	TRUE	0	2	0	FALSE	FALSE

	)40-50-43(46)36-34-32-30-28-26-17-15-13-11-9-7-2/h42H,6-41H2,1-5H3/t42-/m1/s1																	
24778639	InChI=1S/C44H76NO8P/c1-6-8-10-12-14-16-18-19-20-21-22-23-24-25-27-29-31-33-35-37-44(47)53-42(41-52-54(48,49)51-39-38-45(3,4)5)40-50-43(46)36-34-32-30-28-26-17-15-13-11-9-7-2/h8,10,14,16,19-20,22-23,25,27,31,33,42H,6-7,9,11-13,15,17-18,21,24,26,28-30,32,34-41H2,1-5H3/b10-8-,16-14-,20-19-,23-22-,27-25-,33-31-/t42-/m1/s1	Organic compounds	Lipids and lipid-like molecules	Glycerophospholipids	Glycerophosphocholines	Diacylglycerophosphocholines	FALSE	FALSE	FALSE	FALSE	TRUE	FALSE	FALSE	0	1	0	FALSE	FALSE
24778699	InChI=1S/C42H78NO8P/c1-6-8-10-12-14-16-18-20-21-23-25-27-29-31-33-35-42(45)51-40(39-50-52(46,47)49-37-36-43(3,4)5)38-48-41(44)34-32-30-28-26-24-22-19-17-15-13-11-9-7-2/h14,16,20-21,25,27,40H,6-13,15,17-19,22-24,26,28-39H2,1-5H3/b16-14-,21-20-,27-25-/t40-/m1/s1	Organic compounds	Lipids and lipid-like molecules	Glycerophospholipids	Glycerophosphocholines	Diacylglycerophosphocholines	FALSE	FALSE	FALSE	TRUE	FALSE	FALSE	FALSE	1	0	0	FALSE	FALSE
24778972	InChI=1S/C42H80NO8P/c1-6-8-10-12-14-16-18-20-21-23-24-26-28-30-32-34-41(44)48-38-40(39-50-52(46,47)49-37-36-43(3,4)5)51-42(45)35-33-31-29-27-25-22-19-17-15-13-11-9-7-2/h14,16,20-21,40H,6-13,15,17-19,22-39H2,1-5H3/b16-14-,21-20-/t40-/m1/s1	Organic compounds	Lipids and lipid-like molecules	Glycerophospholipids	Glycerophosphocholines	Diacylglycerophosphocholines	FALSE	FALSE	FALSE	FALSE	TRUE	FALSE	FALSE	0	1	0	FALSE	FALSE
52922206	InChI=1S/C43H74NO8P/c1-6-8-10-12-14-16-18-19-20-21-22-23-24-25-26-28-30-32-34-36-43(46)52-41(40-51-53(47,48)50-38-37-44(3,4)5)39-49-42(45)35-33-31-29-27-17-15-13-11-9-7-2/h8,10,14,16,19-20,22-23,25-26,30,32,41H,6-7,9,11-13,15,17-18,21,24,27-29,31,33-40H2,1-5H3/b10-8-,16-14-,20-19-,23-22-,26-25-,32-30-/t41-/m1/s1	Organic compounds	Lipids and lipid-like molecules	Glycerophospholipids	Glycerophosphocholines	Diacylglycerophosphocholines	FALSE	FALSE	FALSE	FALSE	TRUE	FALSE	FALSE	0	1	0	FALSE	FALSE
52922244	InChI=1S/C36H70NO8P/c1-6-8-10-12-14-16-18-20-22-24-26-28-35(38)42-32-34(33-44-46(40,41)43-31-30-37(3,4)5)45-36(39)29-27-25-23-21-19-17-15-13-11-9-7-2/h12,14,34H,6-11,13,15-33H2,1-5H3/b14-12-/t34-/m1/s1	Organic compounds	Lipids and lipid-like molecules	Glycerophospholipids	Glycerophosphocholines	Diacylglycerophosphocholines	FALSE	FALSE	FALSE	TRUE	FALSE	FALSE	FALSE	1	0	0	FALSE	FALSE
52922294	InChI=1S/C44H82NO8P/c1-6-8-10-12-14-16-18-19-20-21-22-23-24-25-27-29-31-33-35-37-44(47)53-42(41-52-54(48,49)51-39-38-45(3,4)5	Organic compounds	Lipids and lipid-like molecules	Glycerophospholipids	Glycerophosphocholines	Diacylglycerophosphocholines	FALSE	FALSE	FALSE	TRUE	FALSE	FALSE	FALSE	1	0	0	FALSE	FALSE

	)40-50-43(46)36-34-32-30-28-26-17-15-13-11-9-7-2/h13-16,19-20,42H,6-12,17-18,21-41H2,1-5H3/b15-13-,16-14-,20-19-/t42-/m1/s1																		
52922783	InChI=1S/C44H80NO8P/c1-6-8-10-12-14-16-18-20-22-24-26-28-30-32-34-36-43(46)50-40-42(41-52-54(48,49)51-39-38-45(3,4)5)53-44(47)37-35-33-31-29-27-25-23-21-19-17-15-13-11-9-7-2/h14,16,20-23,26,28,42H,6-13,15,17-19,24-25,27,29-41H2,1-5H3/b16-14-,22-20-,23-21-,28-26-/t42-/m1/s1	Organic compounds	Lipids and lipid-like molecules	Glycerophospholipids	Glycerophosphocholines	Diacylglycerophosphocholines		FALSE	FALSE	FALSE	FALSE	TRUE	FALSE	FALSE	0	1	0	FALSE	FALSE
52922869	InChI=1S/C48H90NO8P/c1-6-8-10-12-14-16-18-20-22-23-24-25-27-29-31-33-35-37-39-41-48(51)57-46(45-56-58(52,53)55-43-42-49(3,4)5)44-54-47(50)40-38-36-34-32-30-28-26-21-19-17-15-13-11-9-7-2/h9,11,15,17,21,26,46H,6-8,10,12-14,16,18-20,22-25,27-45H2,1-5H3/b11-9-,17-15-,26-21-/t46-/m1/s1	Organic compounds	Lipids and lipid-like molecules	Glycerophospholipids	Glycerophosphocholines	Diacylglycerophosphocholines		FALSE	FALSE	FALSE	TRUE	FALSE	FALSE	FALSE	1	0	0	FALSE	FALSE
52923483	InChI=1S/C52H92NO8P/c1-6-8-10-12-14-16-18-20-22-24-26-28-30-32-34-36-38-40-42-44-51(54)58-48-50(49-60-62(56,57)59-47-46-53(3,4)5)61-52(55)45-43-41-39-37-35-33-31-29-27-25-23-21-19-17-15-13-11-9-7-2/h9,11,15,17,21,23,27,29,33,35,39,41,50H,6-8,10,12-14,16,18-20,22,24-26,28,30-32,34,36-38,40,42-49H2,1-5H3/b11-9-,17-15-,23-21-,29-27-,35-33-,41-39-/t50-/m1/s1	Organic compounds	Lipids and lipid-like molecules	Glycerophospholipids	Glycerophosphocholines	Diacylglycerophosphocholines		FALSE	FALSE	FALSE	TRUE	FALSE	FALSE	FALSE	1	0	0	FALSE	FALSE
lysoPC a C24:0		Organic compounds	Lipids and lipid-like molecules	Glycerophospholipids	Glycerophosphocholines	Lysophosphatidylcholines		FALSE	FALSE	FALSE	FALSE	TRUE	FALSE	FALSE	0	1	0	FALSE	FALSE
lysoPC a C26:0		Organic compounds	Lipids and lipid-like molecules	Glycerophospholipids	Glycerophosphocholines	Lysophosphatidylcholines		FALSE	FALSE	FALSE	TRUE	TRUE	FALSE	FALSE	1	1	1	FALSE	FALSE
lysoPC a C26:1		Organic compounds	Lipids and lipid-like molecules	Glycerophospholipids	Glycerophosphocholines	Lysophosphatidylcholines		FALSE	FALSE	FALSE	FALSE	TRUE	FALSE	FALSE	0	1	0	FALSE	FALSE
lysoPC a C28:0		Organic compounds	Lipids and lipid-like molecules	Glycerophospholipids	Glycerophosphocholines	Lysophosphatidylcholines		FALSE	FALSE	FALSE	TRUE	TRUE	FALSE	FALSE	1	1	1	FALSE	FALSE
lysoPC a C28:1		Organic compounds	Lipids and lipid-like molecules	Glycerophospholipids	Glycerophosphocholines	Lysophosphatidylcholines		FALSE	FALSE	FALSE	TRUE	TRUE	FALSE	FALSE	1	1	1	FALSE	FALSE
pcholhep_hs	InChI=1S/C25H52NO7P/c1-5-6-7-8-9-10-11-12-13-14-15-16-17-18-19-25(28)31-22-24(27)23-33-34(29,30)32-21-20-26(2,3)4/h24,27H,5-23H2,1-4H3/p+1/t24-/m0/s1	Organic compounds	Lipids and lipid-like molecules	Glycerophospholipids	Glycerophosphocholines	Lysophosphatidylcholines	1-acyl-sn-glycero-3-phosphocholines	TRUE	FALSE	TRUE	FALSE	TRUE	FALSE	FALSE	0	1	0	FALSE	TRUE
pcholpalm_hs	InChI=1S/C24H50NO7P/c1-5-6-7-8-9-10-11-12-13-14-15-16-17-18-23(26)22-32-33(28,29)31-20-19-25(2,3)4/h23,26H,5-22H2,1	Organic compounds	Lipids and lipid-like molecules	Glycerophospholipids	Glycerophosphocholines	Lysophosphatidylcholines	1-acyl-sn-glycero-3-phosphocholines	TRUE	FALSE	TRUE	FALSE	TRUE	FALSE	FALSE	0	1	0	FALSE	FALSE

	-4H3/p+1/t23-/m0/s1																		
pcholste_hs	InChI=1S/C26H54NO7P/c1-5-6-7-8-9-10-11-12-13-14-15-16-17-18-19-20-26(29)32-23-25(28)24-34-35(30,31)33-22-21-27(2,3)4/h25,28H,5-24H2,1-4H3/p+1/t25-/m1/s1	Organic compounds	Lipids and lipid-like molecules	Glycerophospholipids	Glycerophosphocholines	Lysophosphatidylcholines	1-acyl-sn-glycero-3-phosphocholines	TRUE	FALSE	TRUE	FALSE	TRUE	FALSE	FALSE	0	1	0	FALSE	TRUE
LysoPC a C15:1_P		Organic compounds	Lipids and lipid-like molecules	Glycerophospholipids	Glycerophosphocholines	Lysophosphatidylcholines	2-acyl-sn-glycero-3-phosphocholines	FALSE	FALSE	FALSE	FALSE	TRUE	FALSE	FALSE	0	1	0	FALSE	FALSE
pchol2palm_hs	InChI=1S/C23H48NO7P/c1-5-6-7-8-9-10-11-12-13-14-15-16-17-23(26)31-22(20-25)21-30-32(27,28)29-19-18-24(2,3)4/h22,25H,5-21H2,1-4H3/t22-/m1/s1	Organic compounds	Lipids and lipid-like molecules	Glycerophospholipids	Glycerophosphocholines	Lysophosphatidylcholines	2-acyl-sn-glycero-3-phosphocholines	TRUE	FALSE	TRUE	FALSE	TRUE	FALSE	FALSE	0	1	0	FALSE	FALSE
11005824	InChI=1S/C26H50NO7P/c1-5-6-7-8-9-10-11-12-13-14-15-16-17-18-19-20-26(29)32-23-25(28)24-34-35(30,31)33-22-21-27(2,3)4/h9-10,12-13,25,28H,5-8,11,14-24H2,1-4H3/b10-9-,13-12-/t25-/m1/s1	Organic compounds	Lipids and lipid-like molecules	Glycerophospholipids	Glycerophosphocholines	Monoacylglycerophosphocholines		FALSE	FALSE	FALSE	TRUE	FALSE	FALSE	FALSE	1	0	0	FALSE	FALSE
11757087	InChI=1S/C28H48NO7P/c1-5-6-7-8-9-10-11-12-13-14-15-16-17-18-19-20-21-22-28(31)34-25-27(30)26-36-37(32,33)35-24-23-29(2,3)4/h6-7,9-10,12-13,15-16,18-19,27,30H,5,8,11,14,17,20-26H2,1-4H3/b7-6-,10-9-,13-12-,16-15-,19-18-/t27-/m1/s1	Organic compounds	Lipids and lipid-like molecules	Glycerophospholipids	Glycerophosphocholines	Monoacylglycerophosphocholines		FALSE	FALSE	FALSE	FALSE	TRUE	FALSE	FALSE	0	1	0	FALSE	FALSE
24779461	InChI=1S/C24H48NO7P/c1-5-6-7-8-9-10-11-12-13-14-15-16-17-18-24(27)30-21-23(26)22-32-33(28,29)31-20-19-25(2,3)4/h10-11,23,26H,5-9,12-22H2,1-4H3/b11-10-/t23-/m1/s1	Organic compounds	Lipids and lipid-like molecules	Glycerophospholipids	Glycerophosphocholines	Monoacylglycerophosphocholines		FALSE	FALSE	FALSE	TRUE	FALSE	FALSE	FALSE	1	0	0	FALSE	FALSE
162127	InChI=1S/C24H52NO6P/c1-5-6-7-8-9-10-11-12-13-14-15-16-17-18-20-29-22-24(26)23-31-32(27,28)30-21-19-25(2,3)4/h24,26H,5-23H2,1-4H3/p+1/t24-/m1/s1	Organic compounds	Lipids and lipid-like molecules	Glycerophospholipids	Glycerophosphocholines	Monoalkylglycerophosphocholines		FALSE	FALSE	FALSE	FALSE	TRUE	FALSE	FALSE	0	1	0	FALSE	FALSE
3983	InChI=1S/C24H52NO6P/c1-5-6-7-8-9-10-11-12-13-14-15-16-17-18-20-29-22-24(26)23-31-32(27,28)30-21-19-25(2,3)4/h24,26H,5-23H2,1-4H3	Organic compounds	Lipids and lipid-like molecules	Glycerophospholipids	Glycerophosphocholines	Monoalkylglycerophosphocholines		FALSE	FALSE	FALSE	TRUE	FALSE	FALSE	FALSE	1	0	0	FALSE	FALSE
LPC(O-16:0)	InChI=1S/C24H52NO6P/c1-5-6-7-8-9-10-11-12-13-14-15-16-17-18-20-29-22-24(26)23-31-32(27,28)30-21-19-25(2,3)4/h24,26H,5-23H2,1-4H3/t24-/m1/s1	Organic compounds	Lipids and lipid-like molecules	Glycerophospholipids	Glycerophosphocholines	Monoalkylglycerophosphocholines		FALSE	FALSE	FALSE	FALSE	TRUE	FALSE	FALSE	0	1	0	FALSE	FALSE
131770064	InChI=1S/C52H92NO10P/c1-10-12-27-33-48-44(5)45(6)50(63-48)35-29-23-19-15-13-14-16-22-26-31-37-52(55)61-46(41-60-64(56,57)59-39-38-53(7,8)9)40-5	Organic compounds	Lipids and lipid-like molecules	Glycerophospholipids	Glycerophosphocholines	Phosphatidylcholines		FALSE	FALSE	FALSE	TRUE	FALSE	FALSE	FALSE	1	0	0	FALSE	FALSE

	8-51(54)36-30-25-21-18-17-20-24-28-34-49-43(4)42(3)47(62-49)32-11-2/h46H,10-41H2,1-9H3/p+1																	
53479327	InChI=1S/C52H82NO8P/c1-6-8-10-12-14-16-18-20-22-24-26-28-30-32-34-36-38-40-42-44-51(54)58-48-50(49-60-62(56,57)59-47-46-53(3,4)5)61-52(55)45-43-41-39-37-35-33-31-29-27-25-23-21-19-17-15-13-11-9-7-2/h9,11,14-17,20-23,26-29,32-35,38-41,50H,6-8,10,12-13,18-19,24-25,30-31,36-37,42-49H2,1-5H3/b11-9-,16-14-,17-15-,22-20-,23-21-,28-26-,29-27-,34-32-,35-33-,40-38-,41-39-/t50-/m1/s1	Organic compounds	Lipids and lipid-like molecules	Glycerophospholipids	Glycerophosphocholines	Phosphatidylcholines	FALSE	FALSE	FALSE	TRUE	FALSE	FALSE	FALSE	1	0	0	FALSE	FALSE
PC 28:1		Organic compounds	Lipids and lipid-like molecules	Glycerophospholipids	Glycerophosphocholines	Phosphatidylcholines	FALSE	FALSE	FALSE	FALSE	TRUE	FALSE	FALSE	0	1	0	FALSE	FALSE
PC 32:2		Organic compounds	Lipids and lipid-like molecules	Glycerophospholipids	Glycerophosphocholines	Phosphatidylcholines	FALSE	FALSE	FALSE	FALSE	TRUE	FALSE	FALSE	0	1	0	FALSE	FALSE
PC 32:3		Organic compounds	Lipids and lipid-like molecules	Glycerophospholipids	Glycerophosphocholines	Phosphatidylcholines	FALSE	FALSE	FALSE	FALSE	TRUE	FALSE	FALSE	0	1	0	FALSE	FALSE
PC 34:0		Organic compounds	Lipids and lipid-like molecules	Glycerophospholipids	Glycerophosphocholines	Phosphatidylcholines	FALSE	FALSE	FALSE	FALSE	TRUE	FALSE	FALSE	0	1	0	FALSE	FALSE
PC 34:2		Organic compounds	Lipids and lipid-like molecules	Glycerophospholipids	Glycerophosphocholines	Phosphatidylcholines	FALSE	FALSE	FALSE	FALSE	TRUE	FALSE	TRUE	0	2	0	FALSE	FALSE
PC 34:3		Organic compounds	Lipids and lipid-like molecules	Glycerophospholipids	Glycerophosphocholines	Phosphatidylcholines	FALSE	FALSE	FALSE	FALSE	TRUE	FALSE	FALSE	0	1	0	FALSE	FALSE
PC 34:4		Organic compounds	Lipids and lipid-like molecules	Glycerophospholipids	Glycerophosphocholines	Phosphatidylcholines	FALSE	FALSE	FALSE	FALSE	TRUE	FALSE	TRUE	0	2	0	FALSE	FALSE
PC 36:1b		Organic compounds	Lipids and lipid-like molecules	Glycerophospholipids	Glycerophosphocholines	Phosphatidylcholines	FALSE	FALSE	FALSE	FALSE	TRUE	FALSE	FALSE	0	1	0	FALSE	FALSE
PC 36:2		Organic compounds	Lipids and lipid-like molecules	Glycerophospholipids	Glycerophosphocholines	Phosphatidylcholines	FALSE	FALSE	FALSE	FALSE	TRUE	FALSE	FALSE	0	1	0	FALSE	FALSE
PC 36:4a		Organic compounds	Lipids and lipid-like molecules	Glycerophospholipids	Glycerophosphocholines	Phosphatidylcholines	FALSE	FALSE	FALSE	FALSE	TRUE	FALSE	FALSE	0	1	0	FALSE	FALSE
PC 36:6		Organic compounds	Lipids and lipid-like molecules	Glycerophospholipids	Glycerophosphocholines	Phosphatidylcholines	FALSE	FALSE	FALSE	FALSE	TRUE	FALSE	FALSE	0	1	0	FALSE	FALSE
PC 38:0		Organic compounds	Lipids and lipid-like molecules	Glycerophospholipids	Glycerophosphocholines	Phosphatidylcholines	FALSE	FALSE	FALSE	FALSE	TRUE	FALSE	FALSE	0	1	0	FALSE	FALSE
PC 38:2a		Organic compounds	Lipids and lipid-like molecules	Glycerophospholipids	Glycerophosphocholines	Phosphatidylcholines	FALSE	FALSE	FALSE	FALSE	TRUE	FALSE	FALSE	0	1	0	FALSE	FALSE
PC 38:4		Organic compounds	Lipids and lipid-like molecules	Glycerophospholipids	Glycerophosphocholines	Phosphatidylcholines	FALSE	FALSE	FALSE	FALSE	TRUE	FALSE	FALSE	0	1	0	FALSE	FALSE
PC 38:5b		Organic compounds	Lipids and lipid-like molecules	Glycerophospholipids	Glycerophosphocholines	Phosphatidylcholines	FALSE	FALSE	FALSE	FALSE	TRUE	FALSE	FALSE	0	1	0	FALSE	FALSE
PC 38:6a		Organic compounds	Lipids and lipid-like molecules	Glycerophospholipids	Glycerophosphocholines	Phosphatidylcholines	FALSE	FALSE	FALSE	FALSE	TRUE	FALSE	FALSE	0	1	0	FALSE	FALSE
PC 40:4b		Organic compounds	Lipids and lipid-like molecules	Glycerophospholipids	Glycerophosphocholines	Phosphatidylcholines	FALSE	FALSE	FALSE	FALSE	TRUE	FALSE	FALSE	0	1	0	FALSE	FALSE
PC 40:5		Organic compounds	Lipids and lipid-like molecules	Glycerophospholipids	Glycerophosphocholines	Phosphatidylcholines	FALSE	FALSE	FALSE	FALSE	TRUE	FALSE	FALSE	0	1	0	FALSE	FALSE
PC 40:6		Organic compounds	Lipids and lipid-like molecules	Glycerophospholipids	Glycerophosphocholines	Phosphatidylcholines	FALSE	FALSE	FALSE	FALSE	TRUE	FALSE	FALSE	0	1	0	FALSE	FALSE
PC 40:8		Organic compounds	Lipids and lipid-like molecules	Glycerophospholipids	Glycerophosphocholines	Phosphatidylcholines	FALSE	FALSE	FALSE	FALSE	TRUE	FALSE	FALSE	0	1	0	FALSE	FALSE

PC 46:2		Organic compounds	Lipids and lipid-like molecules	Glycerophospholipids	Glycerophosphocholines	Phosphatidylcholines	FALSE	FALSE	FALSE	FALSE	TRUE	FALSE	FALSE	0	1	0	FALSE	FALSE
PC(28:2)		Organic compounds	Lipids and lipid-like molecules	Glycerophospholipids	Glycerophosphocholines	Phosphatidylcholines	FALSE	FALSE	FALSE	FALSE	TRUE	FALSE	FALSE	0	1	0	FALSE	FALSE
PC(36:5)		Organic compounds	Lipids and lipid-like molecules	Glycerophospholipids	Glycerophosphocholines	Phosphatidylcholines	FALSE	FALSE	FALSE	FALSE	TRUE	FALSE	FALSE	0	1	0	FALSE	FALSE
53479728	InChI=1S/C43H82NO7P/c1-3-5-7-9-11-13-15-17-19-21-22-24-26-28-30-32-34-36-43(45)49-40-42(41-51-52(46,47)50-39-37-44)48-38-35-33-31-29-27-25-23-20-18-16-14-12-10-8-6-4-2/h14,16-17,19,35,38,42H,3-13,15,18,20-34,36-37,39-41,44H2,1-2H3,(H,46,47)/b16-14-,19-17-,38-35-/t42-/m1/s1	Organic compounds	Lipids and lipid-like molecules	Glycerophospholipids	Glycerophosphoethanolamines		FALSE	FALSE	FALSE	TRUE	FALSE	FALSE	FALSE	1	0	0	FALSE	FALSE
53480048	InChI=1S/C47H90NO7P/c1-3-5-7-9-11-13-15-17-19-21-22-23-24-25-26-28-30-32-34-36-38-40-47(49)53-44-46(45-55-56(50,51)54-43-41-48)52-42-39-37-35-33-31-29-27-20-18-16-14-12-10-8-6-4-2/h14,16-17,19,39,42,46H,3-13,15,18,20-38,40-41,43-45,48H2,1-2H3,(H,50,51)/b16-14-,19-17-,42-39-/t46-/m1/s1	Organic compounds	Lipids and lipid-like molecules	Glycerophospholipids	Glycerophosphoethanolamines		FALSE	FALSE	FALSE	FALSE	TRUE	FALSE	TRUE	0	2	0	FALSE	FALSE
PE 34:2		Organic compounds	Lipids and lipid-like molecules	Glycerophospholipids	Glycerophosphoethanolamines		FALSE	FALSE	FALSE	FALSE	TRUE	FALSE	FALSE	0	1	0	FALSE	FALSE
PEp 36:4		Organic compounds	Lipids and lipid-like molecules	Glycerophospholipids	Glycerophosphoethanolamines		FALSE	FALSE	FALSE	FALSE	TRUE	FALSE	FALSE	0	1	0	FALSE	FALSE
PEp 36:5		Organic compounds	Lipids and lipid-like molecules	Glycerophospholipids	Glycerophosphoethanolamines		FALSE	FALSE	FALSE	FALSE	TRUE	FALSE	FALSE	0	1	0	FALSE	FALSE
PEp 38:4		Organic compounds	Lipids and lipid-like molecules	Glycerophospholipids	Glycerophosphoethanolamines		FALSE	FALSE	FALSE	FALSE	TRUE	FALSE	FALSE	0	1	0	FALSE	FALSE
PEp 38:5		Organic compounds	Lipids and lipid-like molecules	Glycerophospholipids	Glycerophosphoethanolamines		FALSE	FALSE	FALSE	FALSE	TRUE	FALSE	FALSE	0	1	0	FALSE	FALSE
PEp 38:6		Organic compounds	Lipids and lipid-like molecules	Glycerophospholipids	Glycerophosphoethanolamines		FALSE	FALSE	FALSE	FALSE	TRUE	FALSE	FALSE	0	1	0	FALSE	FALSE
PEp 40:6		Organic compounds	Lipids and lipid-like molecules	Glycerophospholipids	Glycerophosphoethanolamines		FALSE	FALSE	FALSE	FALSE	TRUE	FALSE	FALSE	0	1	0	FALSE	FALSE
PEp 40:7		Organic compounds	Lipids and lipid-like molecules	Glycerophospholipids	Glycerophosphoethanolamines		FALSE	FALSE	FALSE	FALSE	TRUE	FALSE	FALSE	0	1	0	FALSE	FALSE
52925128	InChI=1S/C39H76NO7P/c1-3-5-7-9-11-13-15-17-19-20-22-24-26-28-30-32-39(41)47-38(37-46-48(42,43)45-35-33-40)36-44-34-31-29-27-25-23-21-18-16-14-12-10-8-6-4-2/h17,19,31,34,38H,3-16,18,20-30,32-33,35-37,40H2,1-2H3,(H,42,43)/b19-17-,34-31-/t38-/m1/s1	Organic compounds	Lipids and lipid-like molecules	Glycerophospholipids	Glycerophosphoethanolamines	1-(1Z-alkenyl),2-acylglycerophosphoethanolamines	FALSE	FALSE	FALSE	TRUE	FALSE	FALSE	FALSE	1	0	0	FALSE	FALSE
23425816	InChI=1S/C21H44NO6P/c1-2-3-4-5-6-7-8-9-10-11-12-13-14-15-17-26-19-21(23)20-28-29(24,25)27-18-16-22/h15,17,21,23H,2-14,16,18-20,22H2,1H3,(H,24,25)/b17-15+/t21-/m1/s1	Organic compounds	Lipids and lipid-like molecules	Glycerophospholipids	Glycerophosphoethanolamines	1-(1Z-alkenyl)-glycero-3-phosphoethanolamines	FALSE	FALSE	FALSE	FALSE	TRUE	FALSE	FALSE	0	1	0	FALSE	FALSE

52924085	InChI=1S/C39H76NO8P/c1-3-5-7-9-11-13-14-15-16-17-18-19-20-21-22-24-26-28-30-32-39(42)48-37(36-47-49(43,44)46-34-33-40)35-45-38(41)31-29-27-25-23-12-10-8-6-4-2/h17-18,37H,3-16,19-36,40H2,1-2H3,(H,43,44)/b18-17-/t37-/m1/s1	Organic compounds	Lipids and lipid-like molecules	Glycerophospholipids	Glycerophosphoethanolamines	Diacylglycerophosphocholines		FALSE	FALSE	FALSE	FALSE	TRUE	FALSE	FALSE	0	1	0	FALSE	FALSE
LysoPE C18:3		Organic compounds	Lipids and lipid-like molecules	Glycerophospholipids	Glycerophosphoethanolamines	Lysophosphatidylethanolamines	2-acyl-sn-glycero-3-phosphoethanolamines	FALSE	FALSE	FALSE	FALSE	TRUE	FALSE	FALSE	0	1	0	FALSE	FALSE
peole_hs	InChI=1S/C23H46NO7P/c1-2-3-4-5-6-7-8-9-10-11-12-13-14-15-16-17-23(26)29-20-22(25)21-31-32(27,28)30-19-18-24/h9-10,22,25H,2-8,11-21,24H2,1H3,(H,27,28)/b10-9-/t22-/m1/s1	Organic compounds	Lipids and lipid-like molecules	Glycerophospholipids	Glycerophosphoethanolamines	Monoacylglycerophosphocholines		TRUE	FALSE	TRUE	TRUE	FALSE	FALSE	FALSE	1	0	0	FALSE	FALSE
LPE 18:1		Organic compounds	Lipids and lipid-like molecules	Glycerophospholipids	Glycerophosphoethanolamines	Monoacylglycerophosphoethanolamines		FALSE	FALSE	FALSE	TRUE	FALSE	FALSE	FALSE	1	0	0	FALSE	FALSE
131770168	InChI=1S/C52H92NO10P/c1-6-8-26-32-46-40-43(3)48(61-46)33-28-22-18-14-10-13-17-21-25-31-37-52(55)62-47(42-60-64(56,57)59-39-38-53)41-58-51(54)36-30-24-20-16-12-11-15-19-23-29-35-50-45(5)44(4)49(63-50)34-27-9-7-2/h40,47H,6-39,41-42,53H2,1-5H3,(H,56,57)/t47-/m1/s1	Organic compounds	Lipids and lipid-like molecules	Glycerophospholipids	Glycerophosphoethanolamines	Phosphatidylethanolamines		FALSE	FALSE	FALSE	TRUE	FALSE	FALSE	FALSE	1	0	0	FALSE	FALSE
16:0-22:6-N18:0		Organic compounds	Lipids and lipid-like molecules	Glycerophospholipids	Glycerophosphoethanolamines	Phosphatidylethanolamines		FALSE	FALSE	FALSE	FALSE	TRUE	FALSE	FALSE	0	1	0	FALSE	FALSE
18:0-20:4-N18:0		Organic compounds	Lipids and lipid-like molecules	Glycerophospholipids	Glycerophosphoethanolamines	Phosphatidylethanolamines		FALSE	FALSE	FALSE	FALSE	TRUE	FALSE	FALSE	0	1	0	FALSE	FALSE
18:0-22:6-N16:0		Organic compounds	Lipids and lipid-like molecules	Glycerophospholipids	Glycerophosphoethanolamines	Phosphatidylethanolamines		FALSE	FALSE	FALSE	FALSE	TRUE	FALSE	FALSE	0	1	0	FALSE	FALSE
18:0-22:6-N18:0		Organic compounds	Lipids and lipid-like molecules	Glycerophospholipids	Glycerophosphoethanolamines	Phosphatidylethanolamines		FALSE	FALSE	FALSE	FALSE	TRUE	FALSE	FALSE	0	1	0	FALSE	FALSE
53479737	InChI=1S/C49H92NO8P/c1-3-5-7-9-11-13-15-17-19-21-22-23-24-26-28-30-32-34-36-38-40-42-49(52)58-47(46-57-59(53,54)56-44-43-50)45-55-48(51)41-39-37-35-33-31-29-27-25-20-18-16-14-12-10-8-6-4-2/h12,14,17-20,47H,3-11,13,15-16,21-46,50H2,1-2H3,(H,53,54)/b14-12-,19-17-,20-18-/t47-/m1/s1	Organic compounds	Lipids and lipid-like molecules	Glycerophospholipids	Glycerophosphoethanolamines	Phosphatidylethanolamines		FALSE	FALSE	FALSE	TRUE	FALSE	FALSE	FALSE	1	0	0	FALSE	FALSE
P16:0-22:6-N18:0		Organic compounds	Lipids and lipid-like molecules	Glycerophospholipids	Glycerophosphoethanolamines	Phosphatidylethanolamines		FALSE	FALSE	FALSE	FALSE	TRUE	FALSE	FALSE	0	1	0	FALSE	FALSE
P18:0-22:6-N16:0		Organic compounds	Lipids and lipid-like molecules	Glycerophospholipids	Glycerophosphoethanolamines	Phosphatidylethanolamines		FALSE	FALSE	FALSE	FALSE	TRUE	FALSE	FALSE	0	1	0	FALSE	FALSE
P18:0-22:6-N18:0		Organic compounds	Lipids and lipid-like molecules	Glycerophospholipids	Glycerophosphoethanolamines	Phosphatidylethanolamines		FALSE	FALSE	FALSE	FALSE	TRUE	FALSE	FALSE	0	1	0	FALSE	FALSE
24779550	InChI=1S/C42H75O10P/c1-3-5-7-9-11-13-15-17-18-19-20-22-24-26-28-30-32-3	Organic compounds	Lipids and lipid-like molecules	Glycerophospholipids	Glycerophosphoglycerols	Diacylglycerophosphocholines		FALSE	FALSE	FALSE	FALSE	TRUE	FALSE	FALSE	0	1	0	FALSE	FALSE

	4-42(46)52-40(38-51-53(47,48)50-36-39(44)35-43)37-49-41(45)33-31-29-27-25-23-21-16-14-12-10-8-6-4-2/h11,13,17-18,20,22,26,28,39-40,43-44H,3-10,12,14-16,19,21,23-25,27,29-38H2,1-2H3,(H,47,48)/b13-11-,18-17-,22-20-,28-26-/t39-,40+/m0/s1																		
52926742	InChI=1S/C47H89O10P/c1-3-5-7-9-11-13-15-17-19-21-22-23-25-27-29-31-33-35-37-39-47(51)57-45(43-56-58(52,53)55-41-44(49)40-48)42-54-46(50)38-36-34-32-30-28-26-24-20-18-16-14-12-10-8-6-4-2/h11,13,17,19,44-45,48-49H,3-10,12,14-16,18,20-43H2,1-2H3,(H,52,53)/b13-11-,19-17-/t44-,45+/m0/s1	Organic compounds	Lipids and lipid-like molecules	Glycerophospholipids	Glycerophosphoglycerols	Diacylglycerophosphocholines		FALSE	FALSE	FALSE	FALSE	TRUE	FALSE	TRUE	0	2	0	FALSE	FALSE
52926964	InChI=1S/C49H95O10P/c1-3-5-7-9-11-13-15-17-19-21-23-25-27-29-31-33-35-37-39-41-49(53)59-47(45-58-60(54,55)57-43-46(51)42-50)44-56-48(52)40-38-36-34-32-30-28-26-24-22-20-18-16-14-12-10-8-6-4-2/h21,23,46-47,50-51H,3-20,22,24-45H2,1-2H3,(H,54,55)/b23-21-/t46-,47+/m0/s1	Organic compounds	Lipids and lipid-like molecules	Glycerophospholipids	Glycerophosphoglycerols	Diacylglycerophosphocholines		FALSE	FALSE	FALSE	FALSE	TRUE	FALSE	TRUE	0	2	0	FALSE	FALSE
52928416	InChI=1S/C37H73O12P/c1-3-5-7-9-11-13-14-15-16-17-19-21-23-25-27-46-28-30(48-31(38)26-24-22-20-18-12-10-8-6-4-2)29-47-50(44,45)49-37-35(42)33(40)32(39)34(41)36(37)43/h30,32-37,39-43H,3-29H2,1-2H3,(H,44,45)/t30-,32?,33-,34?,35?,36?,37?/m1/s1	Organic compounds	Lipids and lipid-like molecules	Glycerophospholipids	Glycerophosphoinositols	1-alkyl,2-acylglycerophosphoinositols		FALSE	FALSE	FALSE	FALSE	TRUE	FALSE	FALSE	0	1	0	FALSE	FALSE
pailar_hs	InChI=1S/C29H49O12P/c1-2-3-4-5-6-7-8-9-10-11-12-13-14-15-16-17-18-19-23(31)39-20-22(30)21-40-42(37,38)41-29-27(35)25(33)24(32)26(34)28(29)36/h6-7,9-10,12-13,15-16,22,24-30,32-36H,2-5,8,11,14,17-21H2,1H3,(H,37,38)/p-1/b7-6?,10-9?,13-12?,16-15-/t22-,24-,25-,26+,27-,28-,29-/m1/s1	Organic compounds	Lipids and lipid-like molecules	Glycerophospholipids	Glycerophosphoinositols	Lysophosphatidylinositols	1-acyl-sn-glycerol-3-phosphoinositols	TRUE	FALSE	TRUE	FALSE	TRUE	FALSE	FALSE	0	1	0	FALSE	TRUE
52927512	InChI=1S/C44H77O13P/c1-3-5-7-9-11-13-15-16-17-18-19-20-21-22-23-25-27-29-31-33-38(46)56-36(34-54-37(45)32-30-28-26-24-14-12-10-8-6-4-2)35-55-58(52,53)57-44-42(50)40(48)39(47)41(49)43(44)51/h11,13,16-17,19-20,22-23,36,39-44,47-51H,3-10,12,14-15,18,21,24-35H2,1-2H3,(H,52,53	Organic compounds	Lipids and lipid-like molecules	Glycerophospholipids	Glycerophosphoinositols	Phosphatidylinositols		FALSE	FALSE	FALSE	FALSE	TRUE	FALSE	FALSE	0	1	0	FALSE	FALSE

	)/b13-11-,17-16-,20-19-,23-22-/t36-,39?,40-,41?,42?,43?,44?/m1/s1																	
52927667	InChI=1S/C48H89O13P/c1-3-5-7-9-11-13-15-17-19-20-21-22-23-25-27-29-31-33-35-37-42(50)60-40(39-59-62(56,57)61-48-46(54)44(52)43(51)45(53)47(48)55)38-58-41(49)36-34-32-30-28-26-24-18-16-14-12-10-8-6-4-2/h11,13,17,19,40,43-48,51-55H,3-10,12,14-16,18,20-39H2,1-2H3,(H,56,57)/b13-11-,19-17-/t40-,43?,44-,45?,46?,47?,48?/m1/s1	Organic compounds	Lipids and lipid-like molecules	Glycerophospholipids	Glycerophosphoinositols	Phosphatidylinositols	FALSE	FALSE	FALSE	FALSE	TRUE	FALSE	TRUE	0	2	0	FALSE	FALSE
PS 40:4		Organic compounds	Lipids and lipid-like molecules	Glycerophospholipids	Glycerophosphoserines		FALSE	FALSE	FALSE	FALSE	TRUE	FALSE	FALSE	0	1	0	FALSE	FALSE
52926102	InChI=1S/C42H80NO9P/c1-3-5-7-9-11-13-15-17-19-20-21-22-24-26-28-30-32-34-41(44)52-39(37-50-53(47,48)51-38-40(43)42(45)46)36-49-35-33-31-29-27-25-23-18-16-14-12-10-8-6-4-2/h11,13,17,19,39-40H,3-10,12,14-16,18,20-38,43H2,1-2H3,(H,45,46)(H,47,48)/b13-11-,19-17-/t39-,40+/m1/s1	Organic compounds	Lipids and lipid-like molecules	Glycerophospholipids	Glycerophosphoserines	1-alkyl,2-acyl-glycerol-3-phosphoserines	FALSE	FALSE	FALSE	FALSE	TRUE	FALSE	FALSE	0	1	0	FALSE	FALSE
52926118	InChI=1S/C42H78NO9P/c1-3-5-7-9-11-13-15-17-19-21-23-25-27-29-31-33-35-49-36-39(37-50-53(47,48)51-38-40(43)42(45)46)52-41(44)34-32-30-28-26-24-22-20-18-16-14-12-10-8-6-4-2/h12,14,18,20,24,26,39-40H,3-11,13,15-17,19,21-23,25,27-38,43H2,1-2H3,(H,45,46)(H,47,48)/b14-12-,20-18-,26-24-/t39-,40+/m1/s1	Organic compounds	Lipids and lipid-like molecules	Glycerophospholipids	Glycerophosphoserines	1-alkyl,2-acyl-glycerol-3-phosphoserines	FALSE	FALSE	FALSE	FALSE	TRUE	FALSE	FALSE	0	1	0	FALSE	FALSE
52925157	InChI=1S/C33H64NO10P/c1-3-5-7-9-11-13-14-15-17-19-21-23-25-32(36)44-29(27-42-45(39,40)43-28-30(34)33(37)38)26-41-31(35)24-22-20-18-16-12-10-8-6-4-2/h29-30H,3-28,34H2,1-2H3,(H,37,38)(H,39,40)/t29-,30+/m1/s1	Organic compounds	Lipids and lipid-like molecules	Glycerophospholipids	Glycerophosphoserines	Diacylglycerophosphocholines	FALSE	FALSE	FALSE	TRUE	FALSE	TRUE	FALSE	2	0	0	FALSE	FALSE
52925587	InChI=1S/C47H88NO10P/c1-3-5-7-9-11-13-15-17-19-21-22-23-25-27-29-31-33-35-37-39-46(50)58-43(41-56-59(53,54)57-42-44(48)47(51)52)40-55-45(49)38-36-34-32-30-28-26-24-20-18-16-14-12-10-8-6-4-2/h11,13,17,19,43-44H,3-10,12,14-16,18,20-42,48H2,1-2H3,(H,51,52)(H,53,54)/b13-11-,19-17-/t43-,44+/m1/s1	Organic compounds	Lipids and lipid-like molecules	Glycerophospholipids	Glycerophosphoserines	Diacylglycerophosphocholines	FALSE	FALSE	FALSE	FALSE	TRUE	FALSE	FALSE	0	1	0	FALSE	FALSE
52925637	InChI=1S/C48H86NO10P/c1-3-5-7-9-11-13-15-17-19-21-22-24-26-28-30-32-34-	Organic compounds	Lipids and lipid-like molecules	Glycerophospholipids	Glycerophosphoserines	Diacylglycerophosphocholines	FALSE	FALSE	FALSE	FALSE	TRUE	FALSE	TRUE	0	2	0	FALSE	FALSE

	36-38-40-47(51)59-44(42-57-60(54,55)58-43-45(49)48(52)53)41-56-46(50)39-37-35-33-31-29-27-25-23-20-18-16-14-12-10-8-6-4-2/h11,13,17,19,22,24,28,30,44-45H,3-10,12,14-16,18,20-21,23,25-27,29,31-43,49H2,1-2H3,(H,52,53)(H,54,55)/b13-11-,19-17-,24-22-,30-28-/t44-,45+/m1/s1																	
59720717	InChI=1S/C42H80NO10P/c1-3-5-7-9-11-13-15-17-19-21-23-25-27-29-31-33-40(44)50-35-38(36-51-54(48,49)52-37-39(43)42(46)47)53-41(45)34-32-30-28-26-24-22-20-18-16-14-12-10-8-6-4-2/h18,20,38-39H,3-17,19,21-37,43H2,1-2H3,(H,46,47)(H,48,49)/b20-18-/t38-,39+/m1/s1	Organic compounds	Lipids and lipid-like molecules	Glycerophospholipids	Glycerophosphoserines	Diacylglycerophosphocholines	FALSE	FALSE	FALSE	FALSE	TRUE	FALSE	FALSE	0	1	0	FALSE	FALSE
9547091	InChI=1S/C42H78NO10P/c1-3-5-7-9-11-13-15-17-19-21-23-25-27-29-31-33-40(44)50-35-38(36-51-54(48,49)52-37-39(43)42(46)47)53-41(45)34-32-30-28-26-24-22-20-18-16-14-12-10-8-6-4-2/h12,14,18,20,38-39H,3-11,13,15-17,19,21-37,43H2,1-2H3,(H,46,47)(H,48,49)/b14-12-,20-18-/t38-,39+/m1/s1	Organic compounds	Lipids and lipid-like molecules	Glycerophospholipids	Glycerophosphoserines	Diacylglycerophosphocholines	FALSE	FALSE	FALSE	FALSE	TRUE	FALSE	FALSE	0	1	0	FALSE	FALSE
53480355	InChI=1S/C46H80NO10P/c1-3-5-7-9-11-13-15-17-19-20-21-22-24-26-28-30-32-34-36-38-45(49)57-42(40-55-58(52,53)56-41-43(47)46(50)51)39-54-44(48)37-35-33-31-29-27-25-23-18-16-14-12-10-8-6-4-2/h5,7,11,13,17,19,21-22,26,28,42-43H,3-4,6,8-10,12,14-16,18,20,23-25,27,29-41,47H2,1-2H3,(H,50,51)(H,52,53)/b7-5-,13-11-,19-17-,22-21-,28-26-/t42-,43+/m1/s1	Organic compounds	Lipids and lipid-like molecules	Glycerophospholipids	Glycerophosphoserines	Phosphatidylserines	FALSE	FALSE	FALSE	FALSE	TRUE	FALSE	FALSE	0	1	0	FALSE	FALSE
Dioxo-octodecanoic acid		Organic compounds	Lipids and lipid-like molecules	Organonitrogen compounds	Amines	Long-chain fatty acids	FALSE	FALSE	FALSE	TRUE	FALSE	FALSE	FALSE	1	0	0	FALSE	FALSE
85072490	InChI=1S/C27H42O8/c1-6-32-18-12-19-25(5,9-7-24(4)8-10-26(15(2)3)27(19,24)35-26)20(11-16(18)13-28)34-23-22(31)21(30)17(29)14-33-23/h11,13,15,17-23,29-31H,6-10,12,14H2,1-5H3	Organic compounds	Lipids and lipid-like molecules	Prenol lipids	Diterpenoids		FALSE	FALSE	FALSE	TRUE	FALSE	FALSE	FALSE	1	0	0	FALSE	FALSE
25245114	InChI=1S/C20H41O4P/c1-17(2)9-6-10-18(3)11-7-12-19(4)13-8-14-20(5)15-16-24-25(21,22)23/h15,17-19H,6-14,16H2,1-5H3,(H2,21,22,23)/b20-15+/t18-,19-/m1/s1	Organic compounds	Lipids and lipid-like molecules	Prenol lipids	Diterpenoids	Acyclic diterpenoids	FALSE	FALSE	FALSE	FALSE	TRUE	FALSE	FALSE	0	1	0	FALSE	FALSE

5281976	InChI=1S/C5H12O8P2/c1-5(4-6)2-3-12-15(10,11)13-14(7,8)9/h2,6H,3-4H2,1H3,(H,10,11)(H2,7,8,9)/b5-2+	Organic compounds	Lipids and lipid-like molecules	Prenol lipids	Isoprenoid phosphates		Prenol lipids containing a phosphate group linked to an isoprene (2-methylbuta-1,3-diene) unit.	FALSE	FALSE	FALSE	FALSE	TRUE	FALSE	FALSE	0	1	0	FALSE	FALSE
6443726	InChI=1S/C40H50O4/c1-27(17-13-19-29(3)21-23-33-31(5)37(43)35(41)25-39(33,7)8)15-11-12-16-28(2)18-14-20-30(4)22-24-34-32(6)38(44)36(42)26-40(34,9)10/h11-21,23,35-36,41-42H,25-26H2,1-10H3/b12-11+,17-13+,18-14+,23-21+,27-15+,28-16+,29-19+,30-20+/t35-,36-/m0/s1	Organic compounds	Lipids and lipid-like molecules	Prenol lipids	Isoprenoids			FALSE	FALSE	FALSE	FALSE	TRUE	FALSE	TRUE	0	2	0	FALSE	FALSE
6654	InChI=1S/C10H16/c1-7-4-5-8-6-9(7)10(8,2)3/h4,8-9H,5-6H2,1-3H3	Organic compounds	Lipids and lipid-like molecules	Prenol lipids	Monoterpenoids	Bicyclic monoterpenoids		FALSE	FALSE	FALSE	TRUE	FALSE	FALSE	FALSE	1	0	0	FALSE	FALSE
440917	InChI=1S/C10H16/c1-8(2)10-6-4-9(3)5-7-10/h4,10H,1,5-7H2,2-3H3/t10-/m0/s1	Organic compounds	Lipids and lipid-like molecules	Prenol lipids	Monoterpenoids	Menthane monoterpenoids		FALSE	FALSE	FALSE	TRUE	FALSE	FALSE	FALSE	1	0	0	FALSE	FALSE
131752393	InChI=1S/C55H96O/c1-45(2)23-13-24-46(3)25-14-26-47(4)27-15-28-48(5)29-16-30-49(6)31-17-32-50(7)33-18-34-51(8)35-19-36-52(9)37-20-38-53(10)39-21-40-54(11)41-22-42-55(12)43-44-56/h29,31,33,35,37,39,41,43,45-47,56H,13-28,30,32,34,36,38,40,42,44H2,1-12H3/b48-29+,49-31-,50-33-,51-35-,52-37+,53-39+,54-41+,55-43-	Organic compounds	Lipids and lipid-like molecules	Prenol lipids	Polyprenols			FALSE	FALSE	FALSE	TRUE	FALSE	FALSE	FALSE	1	0	0	FALSE	FALSE
53481395	InChI=1S/C102H170N2O22P2/c1-70(2)35-20-36-71(3)37-21-38-72(4)39-22-40-73(5)41-23-42-74(6)43-24-44-75(7)45-25-46-76(8)47-26-48-77(9)49-27-50-78(10)51-28-52-79(11)53-29-54-80(12)55-30-56-81(13)57-31-58-82(14)59-32-60-83(15)61-33-62-84(16)63-34-64-85(17)65-66-119-127(115,116)126-128(117,118)125-101-92(104-87(19)109)95(112)98(90(69-107)122-101)123-100-91(103-86(18)108)94(111)99(89(68-106)121-100)124-102-97(114)96(113)93(110)88(67-105)120-102/h35,37,39,41,43,45,47,49,51,53,55,57,59,61,63,65,88-102,105-107,110-114H,20-34,36,38,40,42,44,46,48,50,52,54,56,58,60,62,64-69H2,1-19H3,(H,103,108)(H,104,109	Organic compounds	Lipids and lipid-like molecules	Prenol lipids	Polyprenols	Polyprenyl phospho carbohydrates		FALSE	FALSE	FALSE	TRUE	FALSE	FALSE	FALSE	1	0	0	FALSE	FALSE

	)(H,115,116)(H,117,118)/b71-37+,72-39+,73-41+,74-43+,75-45+,76-47+,77-49+,78-51+,79-53+,80-55+,81-57+,82-59+,83-61+,84-63+/t85?,88-,89-,90-,91-,92-,93-,94-,95-,96+,97+,98-,99-,100+,101+,102+/m1/s1																		
24892729	InChI=1S/C13H18O2/c1-8(2)5-6-11-7-12(14)9(3)10(4)13(11)15/h5,7,14-15H,6H2,1-4H3	Organic compounds	Lipids and lipid-like molecules	Prenol lipids	Quinone and hydroquinone lipids	Prenylquinones	Ubiquinones	FALSE	FALSE	FALSE	TRUE	FALSE	FALSE	FALSE	1	0	0	FALSE	FALSE
5280473	InChI=1S/C54H82O4/c1-40(2)22-14-23-41(3)24-15-25-42(4)26-16-27-43(5)28-17-29-44(6)30-18-31-45(7)32-19-33-46(8)34-20-35-47(9)36-21-37-48(10)38-39-50-49(11)51(55)53(57-12)54(58-13)52(50)56/h22,24,26,28,30,32,34,36,38H,14-21,23,25,27,29,31,33,35,37,39H2,1-13H3/b41-24+,42-26+,43-28+,44-30+,45-32+,46-34+,47-36+,48-38+	Organic compounds	Lipids and lipid-like molecules	Prenol lipids	Quinone and hydroquinone lipids	Prenylquinones	Ubiquinones	FALSE	FALSE	FALSE	FALSE	TRUE	FALSE	FALSE	0	1	0	FALSE	FALSE
5283544	InChI=1S/C39H58O4/c1-28(2)16-11-17-29(3)18-12-19-30(4)20-13-21-31(5)22-14-23-32(6)24-15-25-33(7)26-27-35-34(8)36(40)38(42-9)39(43-10)37(35)41/h16,18,20,22,24,26H,11-15,17,19,21,23,25,27H2,1-10H3/b29-18+,30-20+,31-22+,32-24+,33-26+	Organic compounds	Lipids and lipid-like molecules	Prenol lipids	Quinone and hydroquinone lipids	Prenylquinones	Ubiquinones	FALSE	FALSE	FALSE	FALSE	TRUE	FALSE	FALSE	0	1	0	FALSE	FALSE
q10	InChI=1S/C59H90O4/c1-44(2)24-15-25-45(3)26-16-27-46(4)28-17-29-47(5)30-18-31-48(6)32-19-33-49(7)34-20-35-50(8)36-21-37-51(9)38-22-39-52(10)40-23-41-53(11)42-43-55-54(12)56(60)58(62-13)59(63-14)57(55)61/h24,26,28,30,32,34,36,38,40,42H,15-23,25,27,29,31,33,35,37,39,41,43H2,1-14H3/b45-26+,46-28+,47-30+,48-32+,49-34+,50-36+,51-38+,52-40+,53-42+	Organic compounds	Lipids and lipid-like molecules	Prenol lipids	Quinone and hydroquinone lipids	Prenylquinones	Ubiquinones	TRUE	TRUE	TRUE	FALSE	TRUE	FALSE	FALSE	0	1	0	FALSE	TRUE
avite1	InChI=1S/C29H50O2/c1-20(2)12-9-13-21(3)14-10-15-22(4)16-11-18-29(8)19-17-26-25(7)27(30)23(5)24(6)28(26)31-29/h20-22,30H,9-19H2,1-8H3/t21-,22-,29-/m1/s1	Organic compounds	Lipids and lipid-like molecules	Prenol lipids	Quinone and hydroquinone lipids	Vitamin E compounds	Tocopherols	TRUE	FALSE	TRUE	FALSE	TRUE	FALSE	FALSE	0	1	0	FALSE	FALSE
101417891	InChI=1S/C15H24O/c1-12-7-5-9-13(2)14(16)11-15(3,4)10-6-8-12/h6-7,10,14,16H,2,5,8-9,11H2,1,3-4H3/b10-6-,12-7-	Organic compounds	Lipids and lipid-like molecules	Prenol lipids	Sesquiterpenoids			FALSE	FALSE	FALSE	TRUE	FALSE	FALSE	FALSE	1	0	0	FALSE	FALSE
6440940	InChI=1S/C15H22O2/c1-9-4-6-12(8-11(3)15(16)17)14-10(2)5-7-13(9)14/h8-9,12-13H,4-7H2,1-3H3,(H,16,17)/b11-8+/t9-,12+,13-/m1/s1	Organic compounds	Lipids and lipid-like molecules	Prenol lipids	Sesquiterpenoids			FALSE	FALSE	FALSE	TRUE	FALSE	TRUE	FALSE	2	0	0	FALSE	FALSE

92139	InChI=1S/C15H22/c1-12(2)6-5-7-14(4)15-10-8-13(3)9-11-15/h6,8-11,14H,5,7H2,1-4H3	Organic compounds	Lipids and lipid-like molecules	Prenol lipids	Sesquiterpenoids			FALSE	FALSE	FALSE	FALSE	TRUE	FALSE	FALSE	0	1	0	FALSE	FALSE
131751606	InChI=1S/C26H42O10/c1-11(9-34-21-19(30)18(29)17(28)14(8-27)35-21)13-7-15-22(2)10-25(32)24(13,4)26(15,33)20(36-25)16-12(22)5-6-23(16,3)31/h11-21,27-33H,5-10H2,1-4H3	Organic compounds	Lipids and lipid-like molecules	Prenol lipids	Terpene glycosides			FALSE	FALSE	FALSE	TRUE	FALSE	FALSE	FALSE	1	0	0	FALSE	FALSE
108062	InChI=1S/C48H78O19/c1-20-10-13-48(15-14-46(6)23(29(48)21(20)2)8-9-28-44(4)16-24(51)39(60)45(5,19-50)27(44)11-12-47(28,46)7)43(61)67-42-36(58)33(55)31(53)26(65-42)18-62-40-37(59)34(56)38(25(17-49)64-40)66-41-35(57)32(54)30(52)22(3)63-41/h8,20-22,24-42,49-60H,9-19H2,1-7H3	Organic compounds	Lipids and lipid-like molecules	Prenol lipids	Terpene glycosides	Triterpene glycosides	Triterpene saponins	FALSE	FALSE	FALSE	TRUE	FALSE	FALSE	FALSE	1	0	0	FALSE	FALSE
131751825	InChI=1S/C31H42O/c1-24(14-9-10-15-25(2)18-12-19-27(4)29(6)32)16-11-17-26(3)21-22-30-28(5)20-13-23-31(30,7)8/h9-12,14-19,21-22H,13,20,23H2,1-8H3/b10-9-,16-11+,18-12+,22-21+,24-14+,25-15+,26-17-,27-19+	Organic compounds	Lipids and lipid-like molecules	Prenol lipids	Triterpenoids			FALSE	FALSE	FALSE	TRUE	FALSE	FALSE	FALSE	1	0	0	FALSE	FALSE
N-palmitoyl-sphingadienine (d18:2/16:0)		Organic compounds	Lipids and lipid-like molecules	Purine nucleosides	Purine 2'-deoxyribonucleosides			FALSE	FALSE	FALSE	TRUE	FALSE	FALSE	FALSE	1	0	0	FALSE	FALSE
nervonoylcarnitine (C24:1)		Organic compounds	Lipids and lipid-like molecules	Purine nucleosides	Purine 2'-deoxyribonucleosides	Acyl carnitines		FALSE	FALSE	FALSE	TRUE	FALSE	FALSE	FALSE	1	0	0	FALSE	FALSE
Dihydro SM 14:0		Organic compounds	Lipids and lipid-like molecules	Sphingolipids				FALSE	FALSE	FALSE	FALSE	TRUE	FALSE	FALSE	0	1	0	FALSE	FALSE
Dihydro SM 16:0		Organic compounds	Lipids and lipid-like molecules	Sphingolipids				FALSE	FALSE	FALSE	FALSE	TRUE	FALSE	FALSE	0	1	0	FALSE	FALSE
Dihydro SM 20:0		Organic compounds	Lipids and lipid-like molecules	Sphingolipids				FALSE	FALSE	FALSE	FALSE	TRUE	FALSE	FALSE	0	1	0	FALSE	FALSE
Dihydro SM 22:0		Organic compounds	Lipids and lipid-like molecules	Sphingolipids				FALSE	FALSE	FALSE	FALSE	TRUE	FALSE	FALSE	0	1	0	FALSE	FALSE
Dihydro SM 24:0		Organic compounds	Lipids and lipid-like molecules	Sphingolipids				FALSE	FALSE	FALSE	FALSE	TRUE	FALSE	FALSE	0	1	0	FALSE	FALSE
Hexose sphingosine		Organic compounds	Lipids and lipid-like molecules	Sphingolipids				FALSE	FALSE	FALSE	FALSE	TRUE	FALSE	FALSE	0	1	0	FALSE	FALSE
dhSM d18:0/18:0		Organic compounds	Lipids and lipid-like molecules	Sphingolipids				FALSE	FALSE	FALSE	FALSE	TRUE	FALSE	FALSE	0	1	0	FALSE	FALSE
Cer d18:0/22:0		Organic compounds	Lipids and lipid-like molecules	Sphingolipids	Ceramides			FALSE	FALSE	FALSE	FALSE	TRUE	FALSE	FALSE	0	1	0	FALSE	FALSE
Cer d18:0/24:1		Organic compounds	Lipids and lipid-like molecules	Sphingolipids	Ceramides			FALSE	FALSE	FALSE	FALSE	TRUE	FALSE	FALSE	0	1	0	FALSE	FALSE
Cer d18:0/26:0		Organic compounds	Lipids and lipid-like molecules	Sphingolipids	Ceramides			FALSE	FALSE	FALSE	FALSE	TRUE	FALSE	FALSE	0	1	0	FALSE	FALSE
Cer d18:1/20:1		Organic compounds	Lipids and lipid-like molecules	Sphingolipids	Ceramides			FALSE	FALSE	FALSE	FALSE	TRUE	FALSE	FALSE	0	1	0	FALSE	FALSE
Cer d18:1/22:1		Organic compounds	Lipids and lipid-like molecules	Sphingolipids	Ceramides			FALSE	FALSE	FALSE	FALSE	TRUE	FALSE	FALSE	0	1	0	FALSE	FALSE

Cer(38:1)		Organic compounds	Lipids and lipid-like molecules	Sphingolipids	Ceramides		FALSE	FALSE	FALSE	TRUE	FALSE	FALSE	FALSE	1	0	0	FALSE	FALSE
Cer(40:0)		Organic compounds	Lipids and lipid-like molecules	Sphingolipids	Ceramides		FALSE	FALSE	FALSE	TRUE	FALSE	FALSE	FALSE	1	0	0	FALSE	FALSE
Cer(42:0)		Organic compounds	Lipids and lipid-like molecules	Sphingolipids	Ceramides		FALSE	FALSE	FALSE	TRUE	FALSE	FALSE	FALSE	1	0	0	FALSE	FALSE
Cer(d18:1/24:0)		Organic compounds	Lipids and lipid-like molecules	Sphingolipids	Ceramides		FALSE	FALSE	FALSE	FALSE	TRUE	FALSE	FALSE	0	1	0	FALSE	FALSE
5283584	InChI=1S/C38H76NO6P/c1-3-5-7-9-11-13-15-17-18-19-20-22-24-26-28-30-32-34-38(41)39-36(35-45-46(42,43)44)37(40)33-31-29-27-25-23-21-16-14-12-10-8-6-4-2/h31,33,36-37,40H,3-30,32,34-35H2,1-2H3,(H,39,41)(H2,42,43,44)/b33-31+/t36-,37+/m0/s1	Organic compounds	Lipids and lipid-like molecules	Sphingolipids	Ceramides	Ceramide 1-phosphates	FALSE	FALSE	FALSE	FALSE	TRUE	FALSE	TRUE	0	2	0	FALSE	FALSE
Cer(d18:0/14:0)	InChI=1S/C32H65NO3/c1-3-5-7-9-11-13-15-16-18-19-21-23-25-27-31(35)30(29-34)33-32(36)28-26-24-22-20-17-14-12-10-8-6-4-2/h30-31,34-35H,3-29H2,1-2H3,(H,33,36)/t30-,31+/m0/s1	Organic compounds	Lipids and lipid-like molecules	Sphingolipids	Ceramides	N-acylsphinganines	FALSE	FALSE	FALSE	FALSE	TRUE	FALSE	FALSE	0	1	0	FALSE	FALSE
5283564	InChI=1S/C34H67NO3/c1-3-5-7-9-11-13-15-17-19-21-23-25-27-29-33(37)32(31-36)35-34(38)30-28-26-24-22-20-18-16-14-12-10-8-6-4-2/h27,29,32-33,36-37H,3-26,28,30-31H2,1-2H3,(H,35,38)/b29-27+/t32-,33+/m0/s1	Organic compounds	Lipids and lipid-like molecules	Sphingolipids	Ceramides	N-acylsphingosines	FALSE	FALSE	FALSE	TRUE	FALSE	FALSE	FALSE	1	0	0	FALSE	FALSE
70698957	InChI=1S/C36H71NO4/c1-3-5-7-9-11-13-14-15-16-17-18-19-20-21-23-25-27-29-31-35(40)36(41)37-33(32-38)34(39)30-28-26-24-22-12-10-8-6-4-2/h28,30,33-35,38-40H,3-27,29,31-32H2,1-2H3,(H,37,41)/b30-28+/t33-,34+,35?/m0/s1	Organic compounds	Lipids and lipid-like molecules	Sphingolipids	Ceramides	N-acylsphingosines	FALSE	FALSE	FALSE	TRUE	FALSE	FALSE	FALSE	1	0	0	FALSE	FALSE
53481060	InChI=1S/C89H159N3O39/c1-6-8-10-12-14-16-18-20-21-22-23-24-25-27-29-31-33-35-37-39-64(107)92-54(55(102)38-36-34-32-30-28-26-19-17-15-13-11-9-7-2)49-120-83-72(113)71(112)76(62(47-97)123-83)126-85-74(115)81(131-89(87(118)119)42-57(104)66(91-52(5)101)79(129-89)68(109)59(106)44-94)77(63(48-98)124-85)127-82-53(40-50(3)99)75(69(110)60(45-95)121-82)125-84-73(114)80(70(111)61(46-96)122-84)130-88(86(116)117)41-56(103)65(90-51(4)100)78(128-88)67(108)58(105)43-93/h53-63,65-85,93-98,102-106,108-115H,6-49H2,1-5H3,(H,90,100)(H,91,101)(H,92,107)(H,1	Organic compounds	Lipids and lipid-like molecules	Sphingolipids	Glycosphingolipids		FALSE	FALSE	FALSE	TRUE	FALSE	FALSE	FALSE	1	0	0	FALSE	FALSE

	16,117)(H,118,119)/t53-,54+,55-,56+,57+,58-,59-,60-,61-,62-,63-,65-,66-,67-,68-,69+,70+,71-,72-,73-,74-,75-,76-,77+,78?,79?,80+,81-,82+,83-,84+,85+,88+,89+/m1/s1																	
53481135	InChI=1S/C70H127N3O29/c1-5-7-9-11-13-15-17-19-20-22-24-26-28-30-32-34-52(84)73-44(45(80)33-31-29-27-25-23-21-18-16-14-12-10-8-6-2)41-95-65-59(89)58(88)61(51(40-77)97-65)98-66-60(90)64(56(86)49(38-75)96-66)102-70(68(93)94)36-47(82)54(72-43(4)79)63(101-70)57(87)50(39-76)99-69(67(91)92)35-46(81)53(71-42(3)78)62(100-69)55(85)48(83)37-74/h44-51,53-66,74-77,80-83,85-90H,5-41H2,1-4H3,(H,71,78)(H,72,79)(H,73,84)(H,91,92)(H,93,94)/t44-,45+,46-,47-,48+,49+,50+,51+,53+,54+,55+,56-,57+,58+,59+,60+,61+,62?,63?,64-,65+,66-,69+,70-/m0/s1	Organic compounds	Lipids and lipid-like molecules	Sphingolipids	Glycosphingolipids		FALSE	FALSE	FALSE	TRUE	FALSE	FALSE	FALSE	1	0	0	FALSE	FALSE
53481196	InChI=1S/C63H118N2O21/c1-4-6-8-10-12-14-16-18-19-20-21-22-23-25-27-29-31-33-35-37-50(73)65-44(45(70)36-34-32-30-28-26-24-17-15-13-11-9-7-5-2)42-81-60-55(77)54(76)57(49(41-68)83-60)84-61-56(78)59(53(75)48(40-67)82-61)86-63(62(79)80)38-46(71)51(64-43(3)69)58(85-63)52(74)47(72)39-66/h44-49,51-61,66-68,70-72,74-78H,4-42H2,1-3H3,(H,64,69)(H,65,73)(H,79,80)/t44-,45+,46-,47+,48+,49+,51+,52+,53-,54+,55+,56+,57+,58?,59-,60+,61-,63-/m0/s1	Organic compounds	Lipids and lipid-like molecules	Sphingolipids	Glycosphingolipids		FALSE	FALSE	FALSE	FALSE	TRUE	FALSE	FALSE	0	1	0	FALSE	FALSE
6321370	InChI=1S/C50H99NO8/c1-3-5-7-9-11-13-15-17-18-19-20-21-22-23-24-25-26-28-30-32-34-36-38-40-46(54)51-43(42-58-50-49(57)48(56)47(55)45(41-52)59-50)44(53)39-37-35-33-31-29-27-16-14-12-10-8-6-4-2/h43-45,47-50,52-53,55-57H,3-42H2,1-2H3,(H,51,54)/t43-,44+,45+,47+,48?,49?,50+/m0/s1	Organic compounds	Lipids and lipid-like molecules	Sphingolipids	Glycosphingolipids		FALSE	FALSE	FALSE	FALSE	TRUE	FALSE	FALSE	0	1	0	FALSE	FALSE
HexCer(36:2)		Organic compounds	Lipids and lipid-like molecules	Sphingolipids	Glycosphingolipids		FALSE	FALSE	FALSE	TRUE	FALSE	FALSE	FALSE	1	0	0	FALSE	FALSE
53481357	InChI=1S/C89H156N4O37/c1-6-8-10-12-14-16-18-20-21-22-23-24-25-26-27-28-29-31-33-35-37-39-41-43-66(107)93-56(57(102)42-40-38-3	Organic compounds	Lipids and lipid-like molecules	Sphingolipids	Glycosphingolipids	Gangliosides	FALSE	FALSE	FALSE	TRUE	FALSE	FALSE	FALSE	1	0	0	FALSE	FALSE

	6-34-32-30-19-17-15-13-11-9-7-2)52-121-82-75(113)74(112)77(65(51-98)123-82)124-83-76(114)81(71(109)62(48-95)122-83)130-89(86(119)120)46-60(105)69(92-55(5)101)80(129-89)73(111)64(50-97)126-88(85(117)118)45-59(104)68(91-54(4)100)79(128-88)72(110)63(49-96)125-87(84(115)116)44-58(103)67(90-53(3)99)78(127-87)70(108)61(106)47-94/h20-21,40,42,56-65,67-83,94-98,102-106,108-114H,6-19,22-39,41,43-52H2,1-5H3,(H,90,99)(H,91,100)(H,92,101)(H,93,107)(H,115,116)(H,117,118)(H,119,120)/b21-20-,42-40+/t56-,57+,58-,59-,60-,61+,62+,63+,64+,65+,67+,68+,69+,70+,71-,72+,73+,74+,75+,76+,77+,78?,79?,80?,81-,82+,83-,87+,88+,89-/m0/s1																		
5431	InChI=1S/C83H145N3O34/c1-6-8-10-12-14-16-18-20-21-22-23-24-25-27-29-31-33-35-37-39-62(99)86-53(54(95)38-36-34-32-30-28-26-19-17-15-13-11-9-7-2)48-111-78-70(105)69(104)72(60(46-90)113-78)115-79-71(106)76(73(61(47-91)114-79)116-77-52(40-49(3)92)65(100)67(102)58(44-88)112-77)120-83(81(109)110)42-56(97)64(85-51(5)94)75(119-83)68(103)59(45-89)117-82(80(107)108)41-55(96)63(84-50(4)93)74(118-82)66(101)57(98)43-87/h20-21,36,38,52-61,63-79,87-91,95-98,100-106H,6-19,22-35,37,39-48H2,1-5H3,(H,84,93)(H,85,94)(H,86,99)(H,107,108)(H,109,110)/b21-20-,38-36+/t52-,53+,54-,55-,56-,57-,58-,59-,60-,61-,63-,64-,65-,66-,67+,68-,69-,70-,71-,72-,73+,74?,75?,76-,77+,78-,79+,82-,83+/m1/s1	Organic compounds	Lipids and lipid-like molecules	Sphingolipids	Glycosphingolipids	Gangliosides		FALSE	FALSE	FALSE	TRUE	FALSE	FALSE	FALSE	1	0	0	FALSE	FALSE
GM3 d18:1/24:1		Organic compounds	Lipids and lipid-like molecules	Sphingolipids	Glycosphingolipids	Gangliosides		FALSE	FALSE	FALSE	TRUE	FALSE	FALSE	FALSE	1	0	0	FALSE	FALSE
GM3 d18:1/26:0		Organic compounds	Lipids and lipid-like molecules	Sphingolipids	Glycosphingolipids	Gangliosides		FALSE	FALSE	FALSE	TRUE	FALSE	FALSE	FALSE	1	0	0	FALSE	FALSE
44260669	InChI=1S/C82H147N3O36/c1-7-9-11-13-15-17-19-21-22-24-26-28-30-32-34-36-54(94)85-47(48(93)35-33-31-29-27-25-23-20-18-16-14-12-10-8-2)42-108-78-68(106)65(103)70(52(40-89)115-78)116-81-69(107)73(60(98)50(38-87)112-81)1	Organic compounds	Lipids and lipid-like molecules	Sphingolipids	Glycosphingolipids	Neutral glycosphingolipids	Galb1-3 Glccb1-3 Galb1-4 Glc- (Lacto series)	FALSE	FALSE	FALSE	FALSE	TRUE	FALSE	TRUE	0	2	0	FALSE	FALSE

	19-77-56(84-46(6)92)72(71(53(41-90)114-77)117-79-66(104)63(101)57(95)43(3)109-79)118-82-75(121-80-67(105)64(102)58(96)44(4)110-80)74(61(99)51(39-88)113-82)120-76-55(83-45(5)91)62(100)59(97)49(37-86)111-76/h33,35,43-44,47-53,55-82,86-90,93,95-107H,7-32,34,36-42H2,1-6H3,(H,83,91)(H,84,92)(H,85,94)/b35-33+/t43?,44?,47-,48+,49?,50?,51?,52?,53?,55?,56?,57+,58+,59-,60-,61-,62+,63?,64?,65+,66-,67-,68?,69?,70+,71+,72+,73-,74-,75?,76+,77-,78+,79+,80+,81-,82-/m0/s1																			
ga2_hs	InChI=1S/C50H92N2O18/c1-4-6-8-10-12-14-15-16-17-19-20-22-24-26-34(57)33(52-38(58)27-25-23-21-18-13-11-9-7-5-2)31-65-49-44(63)42(61)47(36(29-54)67-49)70-50-45(64)43(62)46(37(30-55)68-50)69-48-39(51-32(3)56)41(60)40(59)35(28-53)66-48/h24,26,33-37,39-50,53-55,57,59-64H,4-23,25,27-31H2,1-3H3,(H,51,56)(H,52,58)/b26-24+/t33-,34+,35+,36+,37+,39+,40-,41+,42+,43+,44?,45+,46+,47+,48-,49+,50-/m0/s1	Organic compounds	Lipids and lipid-like molecules	Sphingolipids	Glycosphingolipids	Neutral glycosphingolipids	Galcb1-4 Galb1-4 Glc- (Ganglio series)		TRUE	FALSE	TRUE	TRUE	FALSE	FALSE	FALSE	1	0	0	FALSE	FALSE
11958364	InChI=1S/C42H81NO8/c1-3-5-7-9-11-13-15-17-18-20-22-24-26-28-30-32-38(46)43-35(34-50-42-41(49)40(48)39(47)37(33-44)51-42)36(45)31-29-27-25-23-21-19-16-14-12-10-8-6-4-2/h29,31,35-37,39-42,44-45,47-49H,3-28,30,32-34H2,1-2H3,(H,43,46)/b31-29+/t35-,36+,37+,39+,40-,41+,42+/m0/s1	Organic compounds	Lipids and lipid-like molecules	Sphingolipids	Glycosphingolipids	Neutral glycosphingolipids	Simple glycosylceramides	Glycosyl-N-acylsphingosines	FALSE	FALSE	FALSE	TRUE	FALSE	FALSE	FALSE	1	0	0	FALSE	FALSE
129884273	InChI=1S/C46H87NO13/c1-3-5-7-9-11-13-15-17-19-21-23-25-27-29-33(50)37(47-36(51)30-28-26-24-22-20-18-16-14-12-10-8-6-4-2)39(53)45-42(56)41(55)44(35(32-49)58-45)60-46-43(57)40(54)38(52)34(31-48)59-46/h27,29,33-35,37-46,48-50,52-57H,3-26,28,30-32H2,1-2H3,(H,47,51)/b29-27+/t33-,34-,35-,37-,38+,39?,40+,41-,42-,43-,44-,45?,46+/m1/s1	Organic compounds	Lipids and lipid-like molecules	Sphingolipids	Glycosphingolipids	Neutral glycosphingolipids	Simple glycosylceramides	Glycosyl-N-acylsphingosines	FALSE	FALSE	FALSE	TRUE	FALSE	FALSE	FALSE	1	0	0	FALSE	FALSE
131751332	InChI=1S/C40H77NO9/c1-3-5-7-9-11-13-15-17-19-20-22-24-26-28-33(43)32(31-49-40-38(47)37(46)36(45)35(30-42-50-40)41-39(48)34(44)29-27-25-23-21-18-	Organic compounds	Lipids and lipid-like molecules	Sphingolipids	Glycosphingolipids	Neutral glycosphingolipids	Simple glycosylceramides	Glycosyl-N-acylsphingosines	FALSE	FALSE	FALSE	TRUE	FALSE	FALSE	FALSE	1	0	0	FALSE	FALSE

	16-14-12-10-8-6-4-2/h26,28,32-38,40,42-47H,3-25,27,29-31H2,1-2H3,(H,41,48)/b28-26+																			
44260142	InChI=1S/C50H95NO13/c1-3-5-7-9-11-13-15-17-18-19-20-22-24-26-28-30-32-34-42(55)51-38(39(54)33-31-29-27-25-23-21-16-14-12-10-8-6-4-2)37-61-49-47(60)45(58)48(41(36-53)63-49)64-50-46(59)44(57)43(56)40(35-52)62-50/h31,33,38-41,43-50,52-54,56-60H,3-30,32,34-37H2,1-2H3,(H,51,55)/b33-31+/t38-,39+,40?,41?,43-,44-,45+,46?,47?,48+,49+,50-/m0/s1	Organic compounds	Lipids and lipid-like molecules	Sphingolipids	Glycosphingolipids	Neutral glycosphingolipids	Simple glycosylceramides	Glycosyl-N-acylsphingosines	FALSE	FALSE	FALSE	FALSE	TRUE	FALSE	FALSE	0	1	0	FALSE	FALSE
52931259	InChI=1S/C47H89NO8/c1-3-5-7-9-11-13-15-17-18-19-20-21-22-23-25-27-29-31-33-35-37-43(51)48-40(39-55-47-46(54)45(53)44(52)42(38-49)56-47)41(50)36-34-32-30-28-26-24-16-14-12-10-8-6-4-2/h8,10,34,36,40-42,44-47,49-50,52-54H,3-7,9,11-33,35,37-39H2,1-2H3,(H,48,51)/b10-8-,36-34+/t40-,41+,42+,44+,45?,46?,47+/m0/s1	Organic compounds	Lipids and lipid-like molecules	Sphingolipids	Glycosphingolipids	Neutral glycosphingolipids	Simple glycosylceramides	Glycosyl-N-acylsphingosines	FALSE	FALSE	FALSE	FALSE	TRUE	FALSE	FALSE	0	1	0	FALSE	FALSE
6321355	InChI=1S/C40H77NO8/c1-3-5-7-9-11-13-15-17-19-21-23-25-27-29-34(43)33(32-48-40-39(47)38(46)37(45)35(31-42)49-40)41-36(44)30-28-26-24-22-20-18-16-14-12-10-8-6-4-2/h27,29,33-35,37-40,42-43,45-47H,3-26,28,30-32H2,1-2H3,(H,41,44)/b29-27+/t33-,34+,35+,37+,38?,39?,40+/m0/s1	Organic compounds	Lipids and lipid-like molecules	Sphingolipids	Glycosphingolipids	Neutral glycosphingolipids	Simple glycosylceramides	Glycosyl-N-acylsphingosines	FALSE	FALSE	FALSE	FALSE	TRUE	FALSE	TRUE	0	2	0	FALSE	FALSE
53481402	InChI=1S/C38H73NO11S/c1-3-5-7-9-11-13-15-16-18-19-21-23-25-27-32(41)31(39-34(42)28-26-24-22-20-17-14-12-10-8-6-4-2)30-48-38-36(44)37(50-51(45,46)47)35(43)33(29-40)49-38/h25,27,31-33,35-38,40-41,43-44H,3-24,26,28-30H2,1-2H3,(H,39,42)(H,45,46,47)/b27-25+/t31-,32+,33+,35-,36?,37?,38+/m0/s1	Organic compounds	Lipids and lipid-like molecules	Sphingolipids	Glycosphingolipids	Sulfatides			FALSE	FALSE	FALSE	FALSE	TRUE	FALSE	FALSE	0	1	0	FALSE	FALSE
42608340	InChI=1S/C34H67NO5S/c1-3-5-7-9-11-13-15-17-19-21-23-25-27-29-33(36)32(31-41(38,39)40)35-34(37)30-28-26-24-22-20-18-16-14-12-10-8-6-4-2/h27,29,32-33,36H,3-26,28,30-31H2,1-2H3,(H,35,37)(H,38,39,40)/b29-27+/t32-,33+/m0/s1	Organic compounds	Lipids and lipid-like molecules	Sphingolipids	Other Sphingolipids				FALSE	FALSE	FALSE	FALSE	TRUE	FALSE	TRUE	0	2	0	FALSE	FALSE
138309803	InChI=1S/C44H89N2O6P/c1-6-8-10-12-14-16-18-20-21-22-23-24-26-28-30-32-	Organic compounds	Lipids and lipid-like molecules	Sphingolipids	Phosphosphingolipids				FALSE	FALSE	FALSE	FALSE	TRUE	FALSE	FALSE	0	1	0	FALSE	FALSE

	34-36-38-44(48)45-42(41-52-53(49,50)51-40-39-46(3,4)5)43(47)37-35-33-31-29-27-25-19-17-15-13-11-9-7-2/h35,37,42-43,47H,6-34,36,38-41H2,1-5H3,(H-,45,48,49,50)/b37-35+																	
53481791	InChI=1S/C47H93N2O7P/c1-6-8-10-12-14-16-18-20-21-22-23-24-25-27-28-30-32-34-36-38-44(50)42-47(52)48-45(43-56-57(53,54)55-41-40-49(3,4)5)46(51)39-37-35-33-31-29-26-19-17-15-13-11-9-7-2/h20-21,37,39,44-46,50-51H,6-19,22-36,38,40-43H2,1-5H3,(H-,48,52,53,54)/b21-20-,39-37+/t44?,45-,46+/m0/s1	Organic compounds	Lipids and lipid-like molecules	Sphingolipids	Phosphosphingolipids		FALSE	FALSE	FALSE	TRUE	FALSE	FALSE	FALSE	1	0	0	FALSE	FALSE
PE-Cer(d30:2)		Organic compounds	Lipids and lipid-like molecules	Sphingolipids	Phosphosphingolipids		FALSE	FALSE	FALSE	FALSE	TRUE	FALSE	FALSE	0	1	0	FALSE	FALSE
SM C20:2		Organic compounds	Lipids and lipid-like molecules	Sphingolipids	Phosphosphingolipids		FALSE	FALSE	FALSE	TRUE	FALSE	FALSE	FALSE	1	0	0	FALSE	FALSE
sph1p	InChI=1S/C18H40NO5P/c1-2-3-4-5-6-7-8-9-10-11-12-13-14-15-18(20)17(19)16-24-25(21,22)23/h17-18,20H,2-16,19H2,1H3,(H2,21,22,23)/p-1/t17-,18+/m0/s1	Organic compounds	Lipids and lipid-like molecules	Sphingolipids	Phosphosphingolipids		TRUE	FALSE	TRUE	TRUE	TRUE	FALSE	FALSE	1	1	1	FALSE	FALSE
52931153	InChI=1S/C40H83N2O6P/c1-6-8-10-12-14-16-18-20-22-24-26-28-30-32-34-40(44)41-38(37-48-49(45,46)47-36-35-42(3,4)5)39(43)33-31-29-27-25-23-21-19-17-15-13-11-9-7-2/h38-39,43H,6-37H2,1-5H3,(H-,41,44,45,46)/t38-,39+/m0/s1	Organic compounds	Lipids and lipid-like molecules	Sphingolipids	Phosphosphingolipids	Ceramide phosphocholines	FALSE	FALSE	FALSE	FALSE	TRUE	FALSE	TRUE	0	2	0	FALSE	FALSE
52931189	InChI=1S/C44H89N2O6P/c1-6-8-10-12-14-16-18-19-20-21-22-23-24-25-26-28-30-32-34-36-38-44(48)45-42(41-52-53(49,50)51-40-39-46(3,4)5)43(47)37-35-33-31-29-27-17-15-13-11-9-7-2/h35,37,42-43,47H,6-34,36,38-41H2,1-5H3,(H-,45,48,49,50)/b37-35+/t42-,43+/m0/s1	Organic compounds	Lipids and lipid-like molecules	Sphingolipids	Phosphosphingolipids	Ceramide phosphocholines	FALSE	FALSE	FALSE	FALSE	TRUE	FALSE	FALSE	0	1	0	FALSE	FALSE
52931211	InChI=1S/C46H93N2O6P/c1-6-8-10-12-14-16-18-19-20-21-22-23-24-25-26-27-28-30-32-34-36-38-40-46(50)47-44(43-54-55(51,52)53-42-41-48(3,4)5)45(49)39-37-35-33-31-29-17-15-13-11-9-7-2/h37,39,44-45,49H,6-36,38,40-43H2,1-5H3,(H-,47,50,51,52)/b39-37+/t44-,45+/m0/s1	Organic compounds	Lipids and lipid-like molecules	Sphingolipids	Phosphosphingolipids	Ceramide phosphocholines	FALSE	FALSE	FALSE	FALSE	TRUE	FALSE	TRUE	0	2	0	FALSE	FALSE
SM 12:0		Organic compounds	Lipids and lipid-like molecules	Sphingolipids	Phosphosphingolipids	Ceramide phosphocholines	FALSE	FALSE	FALSE	FALSE	TRUE	FALSE	FALSE	0	1	0	FALSE	FALSE
SM 13:0		Organic compounds	Lipids and lipid-like molecules	Sphingolipids	Phosphosphingolipids	Ceramide phosphocholines	FALSE	FALSE	FALSE	FALSE	TRUE	FALSE	FALSE	0	1	0	FALSE	FALSE

SM 14:0		Organic compounds	Lipids and lipid-like molecules	Sphingolipids	Phosphosphingolipids	Ceramide phosphocholines	FALSE	FALSE	FALSE	FALSE	TRUE	FALSE	FALSE	0	1	0	FALSE	FALSE
SM 14:1		Organic compounds	Lipids and lipid-like molecules	Sphingolipids	Phosphosphingolipids	Ceramide phosphocholines	FALSE	FALSE	FALSE	FALSE	TRUE	FALSE	FALSE	0	1	0	FALSE	FALSE
SM 15:0		Organic compounds	Lipids and lipid-like molecules	Sphingolipids	Phosphosphingolipids	Ceramide phosphocholines	FALSE	FALSE	FALSE	FALSE	TRUE	FALSE	FALSE	0	1	0	FALSE	FALSE
SM 16:1		Organic compounds	Lipids and lipid-like molecules	Sphingolipids	Phosphosphingolipids	Ceramide phosphocholines	FALSE	FALSE	FALSE	FALSE	TRUE	FALSE	FALSE	0	1	0	FALSE	FALSE
SM 21:0		Organic compounds	Lipids and lipid-like molecules	Sphingolipids	Phosphosphingolipids	Ceramide phosphocholines	FALSE	FALSE	FALSE	FALSE	TRUE	FALSE	FALSE	0	1	0	FALSE	FALSE
SM 22:0		Organic compounds	Lipids and lipid-like molecules	Sphingolipids	Phosphosphingolipids	Ceramide phosphocholines	FALSE	FALSE	FALSE	FALSE	TRUE	FALSE	FALSE	0	1	0	FALSE	FALSE
SM 22:1		Organic compounds	Lipids and lipid-like molecules	Sphingolipids	Phosphosphingolipids	Ceramide phosphocholines	FALSE	FALSE	FALSE	FALSE	TRUE	FALSE	FALSE	0	1	0	FALSE	FALSE
SM 23:0		Organic compounds	Lipids and lipid-like molecules	Sphingolipids	Phosphosphingolipids	Ceramide phosphocholines	FALSE	FALSE	FALSE	FALSE	TRUE	FALSE	FALSE	0	1	0	FALSE	FALSE
SM 23:1		Organic compounds	Lipids and lipid-like molecules	Sphingolipids	Phosphosphingolipids	Ceramide phosphocholines	FALSE	FALSE	FALSE	FALSE	TRUE	FALSE	FALSE	0	1	0	FALSE	FALSE
SM 24:0		Organic compounds	Lipids and lipid-like molecules	Sphingolipids	Phosphosphingolipids	Ceramide phosphocholines	FALSE	FALSE	FALSE	FALSE	TRUE	FALSE	FALSE	0	1	0	FALSE	FALSE
SM 26:0		Organic compounds	Lipids and lipid-like molecules	Sphingolipids	Phosphosphingolipids	Ceramide phosphocholines	FALSE	FALSE	FALSE	FALSE	TRUE	FALSE	TRUE	0	3	0	FALSE	FALSE
SM 32:2		Organic compounds	Lipids and lipid-like molecules	Sphingolipids	Phosphosphingolipids	Ceramide phosphocholines	FALSE	FALSE	FALSE	FALSE	TRUE	FALSE	FALSE	0	1	0	FALSE	FALSE
SM d18:1/20:1		Organic compounds	Lipids and lipid-like molecules	Sphingolipids	Phosphosphingolipids	Ceramide phosphocholines	FALSE	FALSE	FALSE	TRUE	FALSE	FALSE	FALSE	1	0	0	FALSE	FALSE
SM(d18:0/22:0)	InChI=1S/C45H93N2O6P/c1-6-8-10-12-14-16-18-20-21-22-23-24-25-27-29-31-33-35-37-39-45(49)46-43(42-53-54(50,51)52-41-40-47(3,4)5)44(48)38-36-34-32-30-28-26-19-17-15-13-11-9-7-2/h43-44,48H,6-42H2,1-5H3,(H-,46,49,50,51)/t43-,44+/m0/s1	Organic compounds	Lipids and lipid-like molecules	Sphingolipids	Phosphosphingolipids	Ceramide phosphocholines	FALSE	FALSE	FALSE	TRUE	TRUE	FALSE	TRUE	1	2	1	FALSE	FALSE
SM(d30:1)		Organic compounds	Lipids and lipid-like molecules	Sphingolipids	Phosphosphingolipids	Ceramide phosphocholines	FALSE	FALSE	FALSE	FALSE	TRUE	FALSE	FALSE	0	1	0	FALSE	FALSE
SM(d32:1)		Organic compounds	Lipids and lipid-like molecules	Sphingolipids	Phosphosphingolipids	Ceramide phosphocholines	FALSE	FALSE	FALSE	FALSE	TRUE	FALSE	FALSE	0	1	0	FALSE	FALSE
spc_hs	InChI=1S/C23H49N2O5P/c1-5-6-7-8-9-10-11-12-13-14-15-16-17-18-23(26)22(24)21-30-31(27,28)29-20-19-25(2,3)4/h17-18,22-23,26H,5-16,19-21,24H2,1-4H3/p+1/b18-17+/t22-,23+/m0/s1	Organic compounds	Lipids and lipid-like molecules	Sphingolipids	Phosphosphingolipids	Sphingosylphosphorylcholines	TRUE	FALSE	TRUE	FALSE	TRUE	FALSE	TRUE	0	2	0	FALSE	FALSE
9547289	InChI=1S/C25H40O4/c1-17-19(15-20(26)16-22(17)27)9-8-18-7-5-13-25(4)21(18)10-11-23(25)29-14-6-12-24(2,3)28/h8-9,20-23,26-28H,1,5-7,10-16H2,2-4H3/b18-8+,19-9-/t20-,21+,22+,23+,25+/m1/s1	Organic compounds	Lipids and lipid-like molecules	Steroids and steroid derivatives			FALSE	FALSE	FALSE	TRUE	FALSE	FALSE	FALSE	1	0	0	FALSE	FALSE
vitd3	InChI=1S/C27H44O/c1-19(2)8-6-9-21(4)25-15-16-26-22(10-7-17-27(25,26)5)12-13-23-18-24(28)14-11-20(23)3/h12-13,19,21,24-26,28H,3,6-11,14-18H2,1-2,4-5H3/b22-12+,23-13	Organic compounds	Lipids and lipid-like molecules	Steroids and steroid derivatives			TRUE	FALSE	TRUE	TRUE	FALSE	FALSE	FALSE	1	0	0	FALSE	FALSE

	-/t21-,24+,25-,26+, 27-/m1/s1																	
tststerone	InChI=1S/C19H28O2/c1-18-9-7-13(20)11-12(18)3-4-14-15-5-6-17(21)19(15,2)10-8-16(14)18/h11,14-17,21H,3-10H2,1-2H3/t14-,15-,16-,17-,18-,19-/m0/s1	Organic compounds	Lipids and lipid-like molecules	Steroids and steroid derivatives	Androstane steroids	Androgens and derivatives	TRUE	FALSE	TRUE	FALSE	TRUE	FALSE	FALSE	0	1	0	TRUE	FALSE
115245	InChI=1S/C26H43NO4/c1-16(4-9-23(29)27-15-24(30)31)20-7-8-21-19-6-5-17-14-18(28)10-12-25(17,2)22(19)11-13-26(20,21)3/h16-22,28H,4-15H2,1-3H3,(H,27,29)(H,30,31)/t16-,17-,18-,19+,20-,21+,22+,25+,26-/m1/s1	Organic compounds	Lipids and lipid-like molecules	Steroids and steroid derivatives	Bile acids, alcohols and derivatives	Glycinated bile acids and derivatives	FALSE	FALSE	FALSE	FALSE	TRUE	FALSE	FALSE	0	1	0	FALSE	FALSE
101602538	InChI=1S/C26H43NO8S/c1-15(4-9-23(29)27-14-24(30)31)19-7-8-20-18-6-5-16-12-17(35-36(32,33)34)10-11-25(16,2)21(18)13-22(28)26(19,20)3/h15-22,28H,4-14H2,1-3H3,(H,27,29)(H,30,31)(H,32,33,34)/t15-,16-,17-,18+,19-,20+,21+,22+,25+,26-/m1/s1	Organic compounds	Lipids and lipid-like molecules	Steroids and steroid derivatives	Bile acids, alcohols and derivatives	Glycited bile acids and derivatives	FALSE	FALSE	FALSE	TRUE	TRUE	FALSE	FALSE	1	1	1	FALSE	FALSE
145453482	InChI=1S/C26H41NO9S/c1-14(4-7-22(30)27-13-23(31)32)17-5-6-18-24-19(12-21(29)26(17,18)3)25(2)9-8-16(36-37(33,34)35)10-15(25)11-20(24)28/h4,7,14-21,24,28-29H,5-6,8-13H2,1-3H3,(H,27,30)(H,31,32)(H,33,34,35)/b7-4+/t14-,15+,16-,17-,18+,19+,20-,21+,24+,25+,26-/m1/s1	Organic compounds	Lipids and lipid-like molecules	Steroids and steroid derivatives	Bile acids, alcohols and derivatives	Glycited bile acids and derivatives	FALSE	FALSE	FALSE	FALSE	TRUE	FALSE	FALSE	0	1	0	FALSE	FALSE
HC02196	InChI=1S/C26H43NO5/c1-15(4-7-22(30)27-14-23(31)32)18-5-6-19-24-20(9-11-26(18,19)3)25(2)10-8-17(28)12-16(25)13-21(24)29/h15-21,24,28-29H,4-14H2,1-3H3,(H,27,30)(H,31,32)/t15-,16+,17-,18-,19+,20+,21+,24+,25+,26-/m1/s1	Organic compounds	Lipids and lipid-like molecules	Steroids and steroid derivatives	Bile acids, alcohols and derivatives	Glycited bile acids and derivatives	TRUE	FALSE	TRUE	FALSE	TRUE	FALSE	FALSE	0	1	0	FALSE	FALSE
HC02197	InChI=1S/C26H43NO7S/c1-16(4-9-23(28)27-15-24(29)30)20-7-8-21-19-6-5-17-14-18(34-35(31,32)33)10-12-25(17,2)22(19)11-13-26(20,21)3/h16-22H,4-15H2,1-3H3,(H,27,28)(H,29,30)(H,31,32,33)/t16-,17-,18-,19+,20-,21+,22+,25+,26-/m1/s1	Organic compounds	Lipids and lipid-like molecules	Steroids and steroid derivatives	Bile acids, alcohols and derivatives	Glycited bile acids and derivatives	TRUE	FALSE	TRUE	TRUE	FALSE	FALSE	FALSE	1	0	0	FALSE	TRUE
M01989	InChI=1S/C26H43NO5/c1-15(4-9-23(30)27-14-24(31)32)19-7-8-20-18-6-5-16-12-17(28)10-11-25(16,2)21(18)13-22(29)26(19,20)3/h15-22,28-29H,4-14H2,1-3H3,(H,27,30)(H,31,32)/t15-,16?,17-,18?,19?,20?,21?,22+,25+,26-/m0/s1	Organic compounds	Lipids and lipid-like molecules	Steroids and steroid derivatives	Bile acids, alcohols and derivatives	Glycited bile acids and derivatives	TRUE	FALSE	TRUE	TRUE	TRUE	TRUE	FALSE	2	1	1	FALSE	FALSE
dgchol	InChI=1S/C26H43NO5/c1-15(4-7-22(30)27-14-23(31)32)18-	Organic compounds	Lipids and lipid-like molecules	Steroids and steroid derivatives	Bile acids, alcohols and derivatives	Glycited bile acids and derivatives	TRUE	FALSE	TRUE	TRUE	TRUE	TRUE	FALSE	2	1	1	FALSE	FALSE

	5-6-19-24-20(9-11-26(18,19)3)25(2)10-8-17(28)12-16(25)13-21(24)29/h15-21,24,28-29H,4-14H2,1-3H3,(H,27,30)(H,31,32)/t15-,16+,17-,18-,19+,20+,21-,24+,25+,26-/m1/s1																		
gchola	InChI=1S/C26H43NO6/c1-14(4-7-22(31)27-13-23(32)33)17-5-6-18-24-19(12-21(30)26(17,18)3)25(2)9-8-16(28)10-15(25)11-20(24)29/h14-21,24,28-30H,4-13H2,1-3H3,(H,27,31)(H,32,33)/t14-,15+,16-,17-,18+,19+,20-,21+,24+,25+,26-/m1/s1	Organic compounds	Lipids and lipid-like molecules	Steroids and steroid derivatives	Bile acids, alcohols and derivatives	Glycited bile acids and derivatives		TRUE	FALSE	TRUE	TRUE	FALSE	TRUE	FALSE	2	0	0	FALSE	FALSE
dchac	InChI=1S/C24H40O4/c1-14(4-9-22(27)28)18-7-8-19-17-6-5-15-12-16(25)10-11-23(15,2)20(17)13-21(26)24(18,19)3/h14-21,25-26H,4-13H2,1-3H3,(H,27,28)/p-1/t14-,15-,16-,17+,18-,19+,20+,21+,23+,24-/m1/s1	Organic compounds	Lipids and lipid-like molecules	Steroids and steroid derivatives	Bile acids, alcohols and derivatives	Hydroxy bile acids, alcohols and derivatives	Dihydroxy bile acids, alcohols and derivatives	TRUE	FALSE	TRUE	TRUE	TRUE	TRUE	FALSE	2	1	1	FALSE	FALSE
165511	InChI=1S/C27H44O3/c1-17(6-5-7-18(2)25(29)30)22-10-11-23-21-9-8-19-16-20(28)12-14-26(19,3)24(21)13-15-27(22,23)4/h8,17-18,20-24,28H,5-7,9-16H2,1-4H3,(H,29,30)/t17-,18?,20+,21+,22-,23+,24+,26+,27-/m1/s1	Organic compounds	Lipids and lipid-like molecules	Steroids and steroid derivatives	Bile acids, alcohols and derivatives	Hydroxy bile acids, alcohols and derivatives	Monohydroxy bile acids, alcohols and derivatives	FALSE	FALSE	FALSE	TRUE	FALSE	FALSE	FALSE	1	0	0	FALSE	FALSE
3081085	InChI=1S/C27H42O4/c1-16(6-5-7-17(2)25(30)31)20-8-9-21-24-22(11-13-27(20,21)4)26(3)12-10-19(28)14-18(26)15-23(24)29/h14,16-17,20-24,29H,5-13,15H2,1-4H3,(H,30,31)/t16-,17?,20-,21+,22+,23-,24+,26+,27-/m1/s1	Organic compounds	Lipids and lipid-like molecules	Steroids and steroid derivatives	Bile acids, alcohols and derivatives	Hydroxy bile acids, alcohols and derivatives	Monohydroxy bile acids, alcohols and derivatives	FALSE	FALSE	FALSE	TRUE	FALSE	FALSE	FALSE	1	0	0	FALSE	FALSE
92997	InChI=1S/C24H38O3/c1-15(4-9-22(26)27)19-7-8-20-18-6-5-16-14-17(25)10-12-23(16,2)21(18)11-13-24(19,20)3/h5,15,17-21,25H,4,6-14H2,1-3H3,(H,26,27)/t15-,17+,18+,19-,20+,21+,23+,24-/m1/s1	Organic compounds	Lipids and lipid-like molecules	Steroids and steroid derivatives	Bile acids, alcohols and derivatives	Hydroxy bile acids, alcohols and derivatives	Monohydroxy bile acids, alcohols and derivatives	FALSE	FALSE	FALSE	TRUE	FALSE	FALSE	FALSE	1	0	0	FALSE	FALSE
HC02191	InChI=1S/C24H40O3/c1-15(4-9-22(26)27)19-7-8-20-18-6-5-16-14-17(25)10-12-23(16,2)21(18)11-13-24(19,20)3/h15-21,25H,4-14H2,1-3H3,(H,26,27)/p-1/t15-,16-,17-,18+,19-,20+,21+,23+,24-/m1/s1	Organic compounds	Lipids and lipid-like molecules	Steroids and steroid derivatives	Bile acids, alcohols and derivatives	Hydroxy bile acids, alcohols and derivatives	Monohydroxy bile acids, alcohols and derivatives	TRUE	FALSE	TRUE	TRUE	FALSE	FALSE	FALSE	1	0	0	FALSE	FALSE
131750961	InChI=1S/C28H40O7/c1-16-17(15-35-23(16)31)14-22(30)26(4,32)28(34)13-12-27(33)20-9-8-18-6-5-7-21(29)25(18,3)19(20)10-11-24(27,28)2/h5,7-8,16-17,19-22,30,32-34H,6,9-15H2,1-4H3	Organic compounds	Lipids and lipid-like molecules	Steroids and steroid derivatives	Bile acids, alcohols and derivatives	Hydroxy bile acids, alcohols and derivatives	Tetrahydroxy bile acids, alcohols and derivatives	FALSE	FALSE	FALSE	TRUE	FALSE	FALSE	FALSE	1	0	0	FALSE	FALSE

cholate	InChI=1S/C24H40O5/c1-13(4-7-21(28)29)16-5-6-17-22-18(12-20(27)24(16,17)3)23(2)9-8-15(25)10-14(23)11-19(22)26/h13-20,22,25-27H,4-12H2,1-3H3,(H,28,29)/p-1/t13-,14+,15-,16-,17+,18+,19-,20+,22+,23+,24-/m1/s1	Organic compounds	Lipids and lipid-like molecules	Steroids and steroid derivatives	Bile acids, alcohols and derivatives	Hydroxy bile acids, alcohols and derivatives	Trihydroxy bile acids, alcohols and derivatives	TRUE	FALSE	TRUE	TRUE	FALSE	FALSE	FALSE	1	0	0	FALSE	FALSE
12443252	InChI=1S/C26H45NO6S/c1-16(4-7-23(30)27-12-13-34(31,32)33)19-5-6-20-24-21(9-11-26(19,20)3)25(2)10-8-18(28)14-17(25)15-22(24)29/h16-22,24,28-29H,4-15H2,1-3H3,(H,27,30)(H,31,32,33)/t16-,17?,18-,19-,20+,21+,22+,24+,25+,26-/m1/s1	Organic compounds	Lipids and lipid-like molecules	Steroids and steroid derivatives	Bile acids, alcohols and derivatives	Taurited bile acids and derivatives		FALSE	FALSE	FALSE	TRUE	FALSE	FALSE	FALSE	1	0	0	FALSE	FALSE
145453483	InChI=1S/C26H43NO10S2/c1-15(4-7-23(30)27-10-11-38(31,32)33)18-5-6-19-24-20(14-22(29)26(18,19)3)25(2)9-8-17(37-39(34,35)36)12-16(25)13-21(24)28/h4,7,15-22,24,28-29H,5-6,8-14H2,1-3H3,(H,27,30)(H,31,32,33)(H,34,35,36)/b7-4+/t15-,16+,17-,18-,19+,20+,21-,22+,24+,25+,26-/m1/s1	Organic compounds	Lipids and lipid-like molecules	Steroids and steroid derivatives	Bile acids, alcohols and derivatives	Taurited bile acids and derivatives		FALSE	FALSE	FALSE	FALSE	TRUE	FALSE	FALSE	0	1	0	FALSE	FALSE
tca3s	InChI=1S/C26H45NO10S2/c1-15(4-7-23(30)27-10-11-38(31,32)33)18-5-6-19-24-20(14-22(29)26(18,19)3)25(2)9-8-17(37-39(34,35)36)12-16(25)13-21(24)28/h15-22,24,28-29H,4-14H2,1-3H3,(H,27,30)(H,31,32,33)(H,34,35,36)/t15-,16+,17-,18-,19+,20+,21-,22+,24+,25+,26-/m1/s1	Organic compounds	Lipids and lipid-like molecules	Steroids and steroid derivatives	Bile acids, alcohols and derivatives	Taurited bile acids and derivatives		TRUE	FALSE	TRUE	TRUE	FALSE	FALSE	FALSE	1	0	0	FALSE	FALSE
tchola	InChI=1S/C26H45NO7S/c1-15(4-7-23(31)27-10-11-35(32,33)34)18-5-6-19-24-20(14-22(30)26(18,19)3)25(2)9-8-17(28)12-16(25)13-21(24)29/h15-22,24,28-30H,4-14H2,1-3H3,(H,27,31)(H,32,33,34)/t15-,16?,17-,18?,19?,20?,21-,22+,24?,25+,26-/m1/s1	Organic compounds	Lipids and lipid-like molecules	Steroids and steroid derivatives	Bile acids, alcohols and derivatives	Taurited bile acids and derivatives		TRUE	FALSE	TRUE	TRUE	TRUE	TRUE	FALSE	2	1	1	FALSE	TRUE
tdchola	InChI=1S/C26H45NO6S/c1-16(4-7-23(30)27-12-13-34(31,32)33)19-5-6-20-24-21(9-11-26(19,20)3)25(2)10-8-18(28)14-17(25)15-22(24)29/h16-22,24,28-29H,4-15H2,1-3H3,(H,27,30)(H,31,32,33)/t16-,17?,18-,19?,20?,21?,22-,24?,25+,26-/m1/s1	Organic compounds	Lipids and lipid-like molecules	Steroids and steroid derivatives	Bile acids, alcohols and derivatives	Taurited bile acids and derivatives		TRUE	FALSE	TRUE	TRUE	FALSE	FALSE	FALSE	1	0	0	FALSE	FALSE
tdechola	InChI=1S/C26H45NO6S/c1-16(4-9-24(30)27-12-13-34(31,32)33)20-7-8-21-19-6-5-17-14-18(28)10-11-25(17,2)22(19)15-23(29)26(20,21)3/h16-23,28-29H,4-15H	Organic compounds	Lipids and lipid-like molecules	Steroids and steroid derivatives	Bile acids, alcohols and derivatives	Taurited bile acids and derivatives		TRUE	FALSE	TRUE	TRUE	TRUE	FALSE	FALSE	1	1	1	FALSE	FALSE

	2,1-3H3,(H,27,30)(H,31,32,33)/p-1/t16-,17-,18-,19+,20-,21+,22+,23+,25+,26-/m1/s1																	
44449966	InChI=1S/C28H44O6/c1-14(2)15(3)23-24(34-23)27(6,32)22-8-10-28(33)17-11-19(29)18-12-20(30)21(31)13-25(18,4)16(17)7-9-26(22,28)5/h11,14-16,18,20-24,30-33H,7-10,12-13H2,1-6H3/t15?,16-,18-,20+,21-,22-,23?,24+,25+,26+,27+,28+/m0/s1	Organic compounds	Lipids and lipid-like molecules	Steroids and steroid derivatives	Cholestane steroids	Cholesterols and derivatives	FALSE	FALSE	FALSE	TRUE	FALSE	FALSE	FALSE	1	0	0	FALSE	FALSE
52931344	InChI=1S/C29H48O5/c1-18(2)8-7-9-19(3)23-10-11-24(28(23,5)14-15-34-20(4)30)22-17-26(32)25-16-21(31)12-13-29(25,6)27(22)33/h17-19,21,23-26,31-32H,7-16H2,1-6H3/t19-,21+,23-,24+,25-,26+,28-,29+/m1/s1	Organic compounds	Lipids and lipid-like molecules	Steroids and steroid derivatives	Cholestane steroids	Cholesterols and derivatives	FALSE	FALSE	FALSE	FALSE	TRUE	FALSE	FALSE	0	1	0	FALSE	FALSE
chsterols	InChI=1S/C27H46O4S/c1-18(2)7-6-8-19(3)23-11-12-24-22-10-9-20-17-21(31-32(28,29)30)13-15-26(20,4)25(22)14-16-27(23,24)5/h9,18-19,21-25H,6-8,10-17H2,1-5H3,(H,28,29,30)/p-1/t19-,21+,22+,23-,24+,25+,26+,27-/m1/s1	Organic compounds	Lipids and lipid-like molecules	Steroids and steroid derivatives	Cholestane steroids	Cholesterols and derivatives	TRUE	TRUE	TRUE	TRUE	FALSE	FALSE	FALSE	1	0	0	FALSE	FALSE
11954106	InChI=1S/C25H33NO/c1-24-12-11-20-17(19(24)9-10-23(24)27)8-7-15-13-22-18(14-25(15,20)2)16-5-3-4-6-21(16)26-22/h3-6,15,17,19-20,23,26-27H,7-14H2,1-2H3/t15-,17-,19-,20-,23-,24-,25-/m0/s1	Organic compounds	Lipids and lipid-like molecules	Steroids and steroid derivatives	Estrane steroids		FALSE	FALSE	FALSE	FALSE	TRUE	FALSE	FALSE	0	1	0	FALSE	FALSE
11docrtsl	InChI=1S/C21H30O4/c1-19-8-5-14(23)11-13(19)3-4-15-16(19)6-9-20(2)17(15)7-10-21(20,25)18(24)12-22/h11,15-17,22,25H,3-10,12H2,1-2H3/t15-,16+,17+,19+,20+,21+/m1/s1	Organic compounds	Lipids and lipid-like molecules	Steroids and steroid derivatives	Hydroxysteroids	21-hydroxysteroids	TRUE	FALSE	TRUE	TRUE	FALSE	FALSE	FALSE	1	0	0	FALSE	FALSE
164838	InChI=1S/C21H32O5/c1-19-7-5-13(23)9-12(19)3-4-14-15-6-8-21(26,17(25)11-22)20(15,2)10-16(24)18(14)19/h12,14-16,18,22,24,26H,3-11H2,1-2H3/t12-,14+,15+,16+,18-,19+,20+,21+/m1/s1	Organic compounds	Lipids and lipid-like molecules	Steroids and steroid derivatives	Hydroxysteroids	21-hydroxysteroids	FALSE	FALSE	FALSE	TRUE	FALSE	TRUE	FALSE	2	0	0	FALSE	FALSE
5311364	InChI=1S/C21H28O4/c1-20-8-7-13(23)9-12(20)3-4-14-15-5-6-16(18(25)11-22)21(15,2)10-17(24)19(14)20/h9,14-16,19,22H,3-8,10-11H2,1-2H3/t14-,15-,16+,19+,20-,21-/m0/s1	Organic compounds	Lipids and lipid-like molecules	Steroids and steroid derivatives	Hydroxysteroids	21-hydroxysteroids	FALSE	FALSE	FALSE	FALSE	TRUE	FALSE	FALSE	0	1	0	FALSE	FALSE
aldstrn	InChI=1S/C21H28O5/c1-20-7-6-13(24)8-12(20)2-3-14-15-4-5-16(18(26)10-22)21(15,11-23)9-17(25)19(14)20/h8,11,14-17,19,22,25H,2-7,9-10H2,1H3/t14-,15-,16+,17-,19+,20-,21+/m0/s1	Organic compounds	Lipids and lipid-like molecules	Steroids and steroid derivatives	Hydroxysteroids	21-hydroxysteroids	TRUE	FALSE	TRUE	FALSE	TRUE	FALSE	FALSE	0	1	0	TRUE	FALSE

cortsn	InChI=1S/C21H28O5/c1-19-7-5-13(23)9-12(19)3-4-14-15-6-8-21(26,17(25)11-22)20(15,2)10-16(24)18(14)19/h9,14-15,18,22,26H,3-8,10-11H2,1-2H3/t14-,15-,18+,19-,20-,21-/m0/s1	Organic compounds	Lipids and lipid-like molecules	Steroids and steroid derivatives	Hydroxysteroids	21-hydroxysteroids	TRUE	FALSE	TRUE	TRUE	FALSE	FALSE	FALSE	1	0	0	TRUE	FALSE
crtsl	InChI=1S/C21H30O5/c1-19-7-5-13(23)9-12(19)3-4-14-15-6-8-21(26,17(25)11-22)20(15,2)10-16(24)18(14)19/h9,14-16,18,22,24,26H,3-8,10-11H2,1-2H3/t14-,15-,16-,18+,19-,20-,21-/m0/s1	Organic compounds	Lipids and lipid-like molecules	Steroids and steroid derivatives	Hydroxysteroids	21-hydroxysteroids	TRUE	FALSE	TRUE	TRUE	FALSE	TRUE	FALSE	4	0	0	TRUE	FALSE
crtstrn	InChI=1S/C21H30O4/c1-20-8-7-13(23)9-12(20)3-4-14-15-5-6-16(18(25)11-22)21(15,2)10-17(24)19(14)20/h9,14-17,19,22,24H,3-8,10-11H2,1-2H3/t14-,15-,16+,17-,19+,20-,21-/m0/s1	Organic compounds	Lipids and lipid-like molecules	Steroids and steroid derivatives	Hydroxysteroids	21-hydroxysteroids	TRUE	FALSE	TRUE	TRUE	TRUE	FALSE	FALSE	1	1	1	TRUE	FALSE
17ahprgstrn	InChI=1S/C21H30O3/c1-13(22)21(24)11-8-18-16-5-4-14-12-15(23)6-9-19(14,2)17(16)7-10-20(18,21)3/h12,16-18,24H,4-11H2,1-3H3/t16-,17+,18+,19+,20+,21+/m1/s1	Organic compounds	Lipids and lipid-like molecules	Steroids and steroid derivatives	Pregne steroids	Gluco/mineralocorticoids, progestogins and derivatives	TRUE	FALSE	TRUE	TRUE	FALSE	TRUE	FALSE	2	0	0	FALSE	FALSE
M00603	InChI=1S/C21H30O4/c1-12(22)21(25)9-7-16-15-5-4-13-10-14(23)6-8-19(13,2)18(15)17(24)11-20(16,21)3/h10,15-18,24-25H,4-9,11H2,1-3H3/t15?,16?,17-,18?,19-,20-,21-/m0/s1	Organic compounds	Lipids and lipid-like molecules	Steroids and steroid derivatives	Pregne steroids	Gluco/mineralocorticoids, progestogins and derivatives	TRUE	FALSE	TRUE	TRUE	FALSE	TRUE	FALSE	2	0	0	FALSE	FALSE
24779607	InChI=1S/C45H75NO4/c1-7-8-16-22-37(46(48)49)23-17-14-12-10-9-11-13-15-18-24-43(47)50-38-29-31-44(5)36(33-38)25-26-39-41-28-27-40(35(4)21-19-20-34(2)3)45(41,6)32-30-42(39)44/h12,14,23,25,34-35,38-42H,7-11,13,15-22,24,26-33H2,1-6H3/b14-12-,37-23+/t35-,38+,39+,40-,41+,42+,44+,45-/m1/s1	Organic compounds	Lipids and lipid-like molecules	Steroids and steroid derivatives	Steroid esters	Cholesteryl esters	FALSE	FALSE	FALSE	TRUE	FALSE	FALSE	FALSE	1	0	0	FALSE	FALSE
12314455	InChI=1S/C33H54O11/c1-16(2)6-7-25(37)32(5,41)24-9-11-33(42)18-12-20(35)19-13-22(43-29-28(40)27(39)26(38)23(15-34)44-29)21(36)14-30(19,3)17(18)8-10-31(24,33)4/h12,16-17,19,21-29,34,36-42H,6-11,13-15H2,1-5H3/t17-,19-,21-,22+,23+,24-,25+,26+,27-,28+,29+,30+,31+,32+,33+/m0/s1	Organic compounds	Lipids and lipid-like molecules	Steroids and steroid derivatives	Steroidal glycosides		FALSE	FALSE	FALSE	TRUE	FALSE	FALSE	FALSE	1	0	0	FALSE	FALSE
167918	InChI=1S/C27H40O11/c1-26-7-6-12(36-24-21(32)19(30)20(31)22(38-24)23(33)34)8-11(26)2-3-13-14-4-5-15(16(29)10-28)27(14)9-17(18(13)26)37-25(27)35/h11-15,17-22,24-25,2	Organic compounds	Lipids and lipid-like molecules	Steroids and steroid derivatives	Steroidal glycosides	Steroid glucuronide conjugates	FALSE	FALSE	FALSE	TRUE	FALSE	FALSE	FALSE	1	0	0	FALSE	FALSE

	8,30-32,35H,2-10H2,1H3,(H,33,34)/t11?,12?,13?,14?,15-,17+,18?,19-,20+,21-,22+,24-,25?,26+,27?/m1/s1																	
21252309	InChI=1S/C30H48O11/c1-13(4-7-21(33)34)16-5-6-17-22-18(12-20(32)30(16,17)3)29(2)9-8-15(10-14(29)11-19(22)31)40-28-25(37)23(35)24(36)26(41-28)27(38)39/h13-20,22-26,28,31-32,35-37H,4-12H2,1-3H3,(H,33,34)(H,38,39)/t13-,14+,15-,16-,17+,18+,19-,20+,22+,23+,24+,25-,26+,28-,29+,30-/m1/s1	Organic compounds	Lipids and lipid-like molecules	Steroids and steroid derivatives	Steroidal glycosides	Steroid glucuronide conjugates	FALSE	FALSE	FALSE	TRUE	FALSE	FALSE	FALSE	1	0	0	FALSE	FALSE
53477753	InChI=1S/C32H51NO11/c1-15(4-7-22(35)33-14-23(36)37)18-5-6-19-24-20(9-11-32(18,19)3)31(2)10-8-17(12-16(31)13-21(24)34)43-30-27(40)25(38)26(39)28(44-30)29(41)42/h15-21,24-28,30,34,38-40H,4-14H2,1-3H3,(H,33,35)(H,36,37)(H,41,42)/t15-,16+,17-,18?,19+,20+,21-,24+,25+,26+,27-,28+,30-,31+,32-/m1/s1	Organic compounds	Lipids and lipid-like molecules	Steroids and steroid derivatives	Steroidal glycosides	Steroid glucuronide conjugates	FALSE	FALSE	FALSE	TRUE	FALSE	FALSE	FALSE	1	0	0	FALSE	FALSE
20848950	InChI=1S/C19H28O6S/c1-18-7-5-12(25-26(22,23)24)9-11(18)3-4-13-14(18)6-8-19(2)15(13)10-16(20)17(19)21/h3,12-16,20H,4-10H2,1-2H3,(H,22,23,24)/p-1/t12-,13+,14-,15-,16+,18-,19-/m0/s1	Organic compounds	Lipids and lipid-like molecules	Steroids and steroid derivatives	Sulfated steroids		FALSE	FALSE	FALSE	FALSE	TRUE	FALSE	FALSE	0	1	0	FALSE	FALSE
5748360	InChI=1S/C21H36O8S2/c1-13(28-30(22,23)24)17-6-7-18-16-5-4-14-12-15(29-31(25,26)27)8-10-20(14,2)19(16)9-11-21(17,18)3/h13-19H,4-12H2,1-3H3,(H,22,23,24)(H,25,26,27)/t13-,14-,15-,16-,17+,18-,19-,20-,21+/m0/s1	Organic compounds	Lipids and lipid-like molecules	Steroids and steroid derivatives	Sulfated steroids		FALSE	FALSE	FALSE	TRUE	FALSE	FALSE	FALSE	1	0	0	FALSE	FALSE
CE6031	InChI=1S/C19H30O5S/c1-18-9-7-13(24-25(21,22)23)11-12(18)3-4-14-15-5-6-17(20)19(15,2)10-8-16(14)18/h12-16H,3-11H2,1-2H3,(H,21,22,23)/p-1/t12-,13+,14-,15-,16-,18-,19-/m0/s1	Organic compounds	Lipids and lipid-like molecules	Steroids and steroid derivatives	Sulfated steroids		TRUE	FALSE	TRUE	FALSE	TRUE	FALSE	FALSE	0	1	0	FALSE	FALSE
seasmet	InChI=1S/C15H22N6O5Se/c1-27(3-2-7(16)15(24)25)4-8-10(22)11(23)14(26-8)21-6-20-9-12(17)18-5-19-13(9)21/h5-8,10-11,14,22-23H,2-4,16H2,1H3,(H2-,17,18,19,24,25)/p+1/t7-,8+,10+,11+,14+,27?/m0/s1	Organic compounds	Nucleosides, nucleotides, and alogues	5'-deoxyribonucleosides			TRUE	FALSE	TRUE	FALSE	TRUE	FALSE	FALSE	0	1	0	FALSE	FALSE
15047	InChI=1S/C9H12N2O6/c12-2-4-5(13)6(14)7(17-4)3-1-10-9(16)11-8(3)15/h1,4-7,12-14H,2H2,(H2,10,11,15,16)/t4-,5-,6-,7+/m1/s1	Organic compounds	Nucleosides, nucleotides, and alogues	Nucleoside and nucleotide alogues			FALSE	FALSE	FALSE	TRUE	FALSE	FALSE	FALSE	1	0	0	FALSE	FALSE

1869	InChI=1S/C11H15N5O4/c1-12-9-6-10(14-3-13-9)16(4-15-6)11-8(19)7(18)5(2-17)20-11/h3-5,7-8,11,17-19H,2H2,1H3,(H,12,13,14)	Organic compounds	Nucleosides, nucleotides, and alogues	Purine nucleosides					FALSE	FALSE	FALSE	TRUE	FALSE	FALSE	FALSE	1	0	0	FALSE	FALSE
27476	InChI=1S/C11H15N5O4/c1-15-3-14-10-6(9(15)12)13-4-16(10)11-8(19)7(18)5(2-17)20-11/h3-5,7-8,11-12,17-19H,2H2,1H3/t5-,7-,8-,11-/m1/s1	Organic compounds	Nucleosides, nucleotides, and alogues	Purine nucleosides					FALSE	FALSE	FALSE	TRUE	FALSE	FALSE	FALSE	1	0	0	FALSE	FALSE
gsn	InChI=1S/C10H13N5O5/c11-10-13-7-4(8(19)14-10)12-2-15(7)9-6(18)5(17)3(1-16)20-9/h2-3,5-6,9,16-18H,1H2,(H3,11,13,14,19)/t3-,5-,6-,9-/m1/s1	Organic compounds	Nucleosides, nucleotides, and alogues	Purine nucleosides					TRUE	TRUE	TRUE	FALSE	TRUE	FALSE	FALSE	0	1	0	FALSE	FALSE
ins	InChI=1S/C10H12N4O5/c15-1-4-6(16)7(17)10(19-4)14-3-13-5-8(14)11-2-12-9(5)18/h2-4,6-7,10,15-17H,1H2,(H,11,12,18)/t4-,6-,7-,10-/m1/s1	Organic compounds	Nucleosides, nucleotides, and alogues	Purine nucleosides					TRUE	TRUE	TRUE	FALSE	TRUE	FALSE	TRUE	0	2	0	FALSE	FALSE
xtsn	InChI=1S/C10H12N4O6/c15-1-3-5(16)6(17)9(20-3)14-2-11-4-7(14)12-10(19)13-8(4)18/h2-3,5-6,9,15-17H,1H2,(H2,12,13,18,19)/t3-,5-,6-,9-/m1/s1	Organic compounds	Nucleosides, nucleotides, and alogues	Purine nucleosides					TRUE	TRUE	TRUE	FALSE	TRUE	FALSE	FALSE	0	1	0	FALSE	FALSE
135440064	InChI=1S/C10H13N5O5/c11-9-13-7-6(8(18)14-9)12-10(19)15(7)5-1-3(17)4(2-16)20-5/h3-5,16-17H,1-2H2,(H,12,19)(H3,11,13,14,18)/t3-,4+,5+/m0/s1	Organic compounds	Nucleosides, nucleotides, and alogues	Purine nucleosides	Purine 2'-deoxyribonucleosides				FALSE	FALSE	FALSE	TRUE	FALSE	FALSE	FALSE	1	0	0	FALSE	FALSE
din	InChI=1S/C10H12N4O4/c15-2-6-5(16)1-7(18-6)14-4-13-8-9(14)11-3-12-10(8)17/h3-7,15-16H,1-2H2,(H,11,12,17)/t5-,6+,7+/m0/s1	Organic compounds	Nucleosides, nucleotides, and alogues	Purine nucleosides	Purine 2'-deoxyribonucleosides				TRUE	FALSE	TRUE	TRUE	FALSE	FALSE	FALSE	1	0	0	FALSE	FALSE
coa	InChI=1S/C21H36N7O16P3S/c1-21(2,16(31)19(32)24-4-3-12(29)23-5-6-48)8-41-47(38,39)44-46(36,37)40-7-11-15(43-45(33,34)35)14(30)20(42-11)28-10-27-13-17(22)25-9-26-18(13)28/h9-11,14-16,20,30-31,48H,3-8H2,1-2H3,(H,23,29)(H,24,32)(H,36,37)(H,38,39)(H2,22,25,26)(H2,33,34,35)/p-4/t11-,14-,15-,16+,20-/m1/s1	Organic compounds	Nucleosides, nucleotides, and alogues	Purine nucleotides	Purine ribonucleotides	Purine ribonucleoside bisphosphates	Purine ribonucleoside 3',5'-bisphosphates	Coenzyme A and derivatives	TRUE	TRUE	TRUE	FALSE	TRUE	FALSE	FALSE	0	1	0	FALSE	TRUE
445408	InChI=1S/C10H14N2O6/c1-4-2-12(10(17)11-8(4)16)9-7(15)6(14)5(3-13)18-9/h2,5-7,9,13-15H,3H2,1H3,(H,11,16,17)/t5-,6-,7-,9-/m1/s1	Organic compounds	Nucleosides, nucleotides, and alogues	Pyrimidine nucleosides					FALSE	FALSE	FALSE	FALSE	TRUE	FALSE	FALSE	0	1	0	FALSE	FALSE
uri	InChI=1S/C9H12N2O6/c12-3-4-6(14)7(15)8(17-4)11-2-1-5(13)10-9(11)16/h1-2,4,6-8,12,14-15H,3H2,(H,10,13,16)/t4-,6-,7-,8-/m1/s1	Organic compounds	Nucleosides, nucleotides, and alogues	Pyrimidine nucleosides					TRUE	TRUE	TRUE	TRUE	TRUE	FALSE	TRUE	1	3	1	FALSE	FALSE
duri	InChI=1S/C9H12N2O5/c12-4-6-5(13)3-8(16-6)11-2-1-7(14)10-9(11)15/h1-2,5-6,8,12-13H,3-4H2,(H,	Organic compounds	Nucleosides, nucleotides, and alogues	Pyrimidine nucleosides	Pyrimidine 2'-deoxyribonucleosides				TRUE	TRUE	TRUE	TRUE	FALSE	FALSE	FALSE	1	0	0	FALSE	FALSE

	10,14,15)/t5-,6+,8+/m0/s1																			
53481030	InChI=1S/C9H12N3O7P/c10-5-1-2-12(9(14)11-5)8-7-6(4(3-13)17-8)18-20(15,16)19-7/h1-2,4,6-8,13H,3H2,(H,15,16)(H2,10,11,14)/t4-,6-,7-,8?/m1/s1	Organic compounds	Nucleosides, nucleotides, and alogues	Pyrimidine nucleotides	Cyclic pyrimidine nucleotides	2',3'-cyclic pyrimidine nucleotides			FALSE	FALSE	FALSE	TRUE	FALSE	FALSE	FALSE	1	0	0	FALSE	FALSE
2450	InChI=1S/C3H6BrNO4/c4-3(1-6,2-7)5(8)9/h6-7H,1-2H2	Organic compounds	Organic 1,3-dipolar compounds	Allyl-type 1,3-dipolar organic compounds	Organic nitro compounds	C-nitro compounds			FALSE	FALSE	FALSE	FALSE	TRUE	FALSE	FALSE	0	1	0	FALSE	FALSE
25081464	InChI=1S/C63H97N19O26/c1-29(2)19-37(57(102)73-32(6)53(98)76-35(15-17-48(91)92)55(100)70-24-43(85)68-23-42(84)69-25-46(88)82-51(30(3)4)61(106)77-36(62(107)108)13-10-18-67-63(65)66)79-58(103)38(20-33-11-8-7-9-12-33)80-59(104)39(21-49(93)94)75-45(87)27-71-54(99)34(14-16-47(89)90)74-44(86)26-72-56(101)41(28-83)81-60(105)40(22-50(95)96)78-52(97)31(5)64/h7-9,11-12,29-32,34-41,51,83H,10,13-28,64H2,1-6H3,(H,68,85)(H,69,84)(H,70,100)(H,71,99)(H,72,101)(H,73,102)(H,74,86)(H,75,87)(H,76,98)(H,77,106)(H,78,97)(H,79,103)(H,80,104)(H,81,105)(H,82,88)(H,89,90)(H,91,92)(H,93,94)(H,95,96)(H,107,108)(H4,65,66,67)	Organic compounds	Organic Polymers	Polypeptides					FALSE	FALSE	FALSE	TRUE	FALSE	FALSE	FALSE	1	0	0	FALSE	FALSE
pmeth	InChI=1S/C18H37NO2/c1-2-3-4-5-6-7-8-9-10-11-12-13-14-15-18(21)19-16-17-20/h20H,2-17H2,1H3,(H,19,21)	Organic compounds	Organic acids and derivatives	Carboximidic acids and derivatives	Carboximidic acids				TRUE	FALSE	TRUE	TRUE	FALSE	FALSE	FALSE	1	0	0	FALSE	FALSE
C02356	InChI=1S/C4H9NO2/c1-2-3(5)4(6)7/h3H,2,5H2,1H3,(H,6,7)/t3-/m0/s1	Organic compounds	Organic acids and derivatives	Carboxylic acids and derivatives	Amino acids, peptides, and alogues	Amino acids and derivatives	Alpha amino acids	L-alpha-amino acids	TRUE	TRUE	TRUE	TRUE	TRUE	FALSE	FALSE	1	1	1	FALSE	FALSE
134508	InChI=1S/C5H8N2O2/c6-3-1-2-4(8)7-5(3)9/h3H,1-2,6H2,(H,7,8,9)	Organic compounds	Organic acids and derivatives	Carboxylic acids and derivatives	Amino acids, peptides, and alogues	Amino acids and derivatives	Alpha amino acids and derivatives		FALSE	FALSE	FALSE	TRUE	FALSE	TRUE	FALSE	2	0	0	FALSE	FALSE
7074739	InChI=1S/C11H18N2O2/c1-7(2)6-8-11(15)13-5-3-4-9(13)10(14)12-8/h7-9H,3-6H2,1-2H3,(H,12,14)/t8-,9-/m0/s1	Organic compounds	Organic acids and derivatives	Carboxylic acids and derivatives	Amino acids, peptides, and alogues	Amino acids and derivatives	Alpha amino acids and derivatives		FALSE	FALSE	FALSE	FALSE	TRUE	FALSE	FALSE	0	1	0	FALSE	FALSE
CE1401	InChI=1S/C4H7NOS/c5-3-1-2-7-4(3)6/h3H,1-2,5H2/p+1/t3-/m0/s1	Organic compounds	Organic acids and derivatives	Carboxylic acids and derivatives	Amino acids, peptides, and alogues	Amino acids and derivatives	Alpha amino acids and derivatives		TRUE	TRUE	TRUE	TRUE	FALSE	FALSE	FALSE	1	0	0	FALSE	FALSE
crtn	InChI=1S/C4H7N3O/c1-7-2-3(8)6-4(7)5/h2H2,1H3,(H2,5,6,8)	Organic compounds	Organic acids and derivatives	Carboxylic acids and derivatives	Amino acids, peptides, and alogues	Amino acids and derivatives	Alpha amino acids and derivatives		TRUE	TRUE	TRUE	FALSE	TRUE	FALSE	FALSE	0	1	0	FALSE	FALSE
pcreat	InChI=1S/C4H10N3O5P/c1-7(2-3(8)9)4(5)6-13(10,11)12/h2H2,1H3,(H,8,9)(H4,5,6,10,11,12)/p-1	Organic compounds	Organic acids and derivatives	Carboxylic acids and derivatives	Amino acids, peptides, and alogues	Amino acids and derivatives	Alpha amino acids and derivatives		TRUE	TRUE	TRUE	TRUE	TRUE	FALSE	FALSE	1	1	1	FALSE	FALSE

ala_L	InChI=1S/C3H7NO2/c1-2(4)3(5)6/h2H,4H2,1H3,(H,5,6)/t2-/m0/s1	Organic compounds	Organic acids and derivatives	Carboxylic acids and derivatives	Amino acids, peptides, and alogues	Amino acids and derivatives	Alpha amino acids and derivatives	Alanine and derivatives		TRUE	TRUE	TRUE	TRUE	TRUE	TRUE	TRUE	5	2	1	TRUE	FALSE
440599	InChI=1S/C6H12N2O/c7-5-3-1-2-4-8-6(5)9/h5H,1-4,7H2,(H,8,9)/t5-/m0/s1	Organic compounds	Organic acids and derivatives	Carboxylic acids and derivatives	Amino acids, peptides, and alogues	Amino acids and derivatives	Alpha amino acids and derivatives	Alpha amino acid amides		FALSE	FALSE	FALSE	FALSE	TRUE	FALSE	FALSE	0	1	0	FALSE	FALSE
429581	InChI=1S/C20H40N2O8/c1-10(2)21(11(3)4)18(22(12(5)6)13(7)8)20(29)30-9-14(23)15(24)16(25)17(26)19(27)28/h10-18,23-26H,9H2,1-8H3,(H,27,28)	Organic compounds	Organic acids and derivatives	Carboxylic acids and derivatives	Amino acids, peptides, and alogues	Amino acids and derivatives	Alpha amino acids and derivatives	Alpha amino acid esters		FALSE	FALSE	FALSE	TRUE	FALSE	FALSE	FALSE	1	0	0	FALSE	FALSE
dmgly	InChI=1S/C4H9NO2/c1-5(2)3-4(6)7/h3H2,1-2H3,(H,6,7)	Organic compounds	Organic acids and derivatives	Carboxylic acids and derivatives	Amino acids, peptides, and alogues	Amino acids and derivatives	Alpha amino acids and derivatives	Alpha amino acids		TRUE	FALSE	TRUE	TRUE	TRUE	FALSE	FALSE	1	1	1	FALSE	FALSE
gly	InChI=1S/C2H5NO2/c3-1-2(4)5/h1,3H2,(H,4,5)	Organic compounds	Organic acids and derivatives	Carboxylic acids and derivatives	Amino acids, peptides, and alogues	Amino acids and derivatives	Alpha amino acids and derivatives	Alpha amino acids		TRUE	TRUE	TRUE	TRUE	FALSE	TRUE	FALSE	6	0	0	FALSE	FALSE
glyb	InChI=1S/C5H11NO2/c1-6(2,3)4-5(7)8/h4H2,1-3H3	Organic compounds	Organic acids and derivatives	Carboxylic acids and derivatives	Amino acids, peptides, and alogues	Amino acids and derivatives	Alpha amino acids and derivatives	Alpha amino acids		TRUE	FALSE	TRUE	FALSE	TRUE	FALSE	TRUE	0	3	0	FALSE	FALSE
hom_L	InChI=1S/C4H9NO3/c5-3(1-2-6)4(7)8/h3,6H,1-2,5H2,(H,7,8)	Organic compounds	Organic acids and derivatives	Carboxylic acids and derivatives	Amino acids, peptides, and alogues	Amino acids and derivatives	Alpha amino acids and derivatives	Alpha amino acids		TRUE	TRUE	TRUE	TRUE	FALSE	TRUE	FALSE	2	0	0	FALSE	FALSE
sarcs	InChI=1S/C3H7NO2/c1-4-2-3(5)6/h4H,2H2,1H3,(H,5,6)	Organic compounds	Organic acids and derivatives	Carboxylic acids and derivatives	Amino acids, peptides, and alogues	Amino acids and derivatives	Alpha amino acids and derivatives	Alpha amino acids		TRUE	FALSE	TRUE	TRUE	TRUE	FALSE	FALSE	1	1	1	FALSE	FALSE
439691	InChI=1S/C4H9NO2/c1-2-3(5)4(6)7/h3H,2,5H2,1H3,(H,6,7)/t3-/m1/s1	Organic compounds	Organic acids and derivatives	Carboxylic acids and derivatives	Amino acids, peptides, and alogues	Amino acids and derivatives	Alpha amino acids and derivatives	Alpha amino acids	D-alpha-amino acids	FALSE	FALSE	FALSE	FALSE	TRUE	FALSE	FALSE	0	1	0	FALSE	FALSE
164795	InChI=1S/C7H16N2O2/c1-9-5-3-2-4-6(8)7(10)11/h6,9H,2-5,8H2,1H3,(H,10,11)/t6-/m0/s1	Organic compounds	Organic acids and derivatives	Carboxylic acids and derivatives	Amino acids, peptides, and alogues	Amino acids and derivatives	Alpha amino acids and derivatives	Alpha amino acids	L-alpha-amino acids	FALSE	FALSE	FALSE	FALSE	TRUE	FALSE	FALSE	0	1	0	FALSE	FALSE
9085	InChI=1S/C7H16N4O2/c8-5(6(12)13)3-1-2-4-11-7(9)10/h5H,1-4,8H2,(H,12,13)(H4,9,10,11)/t5-/m0/s1	Organic compounds	Organic acids and derivatives	Carboxylic acids and derivatives	Amino acids, peptides, and alogues	Amino acids and derivatives	Alpha amino acids and derivatives	Alpha amino acids	L-alpha-amino acids	FALSE	FALSE	FALSE	FALSE	TRUE	FALSE	FALSE	0	1	0	FALSE	FALSE
92832	InChI=1S/C8H16N2O3/c1-6(11)10-5-3-2-4-7(9)8(12)13/h7H,2-5,9H2,1H3,(H,10,11)(H,12,13)/t7-/m0/s1	Organic compounds	Organic acids and derivatives	Carboxylic acids and derivatives	Amino acids, peptides, and alogues	Amino acids and derivatives	Alpha amino acids and derivatives	Alpha amino acids	L-alpha-amino acids	FALSE	FALSE	FALSE	TRUE	FALSE	FALSE	FALSE	1	0	0	FALSE	FALSE
L2aadp	InChI=1S/C6H11NO4/c7-4(6(10)11)2-1-3-5(8)9/h4H,1-3,7H2,(H,8,9)(H,10,11)/p-1/t4-/m0/s1	Organic compounds	Organic acids and derivatives	Carboxylic acids and derivatives	Amino acids, peptides, and alogues	Amino acids and derivatives	Alpha amino acids and derivatives	Alpha amino acids	L-alpha-amino acids	TRUE	TRUE	TRUE	TRUE	FALSE	FALSE	FALSE	1	0	0	FALSE	FALSE
Lpipecol	InChI=1S/C6H11NO2/c8-6(9)5-3-1-2-4-7-5/h5,7H,1-4H2,(H,8,9)/t5-/m0/s1	Organic compounds	Organic acids and derivatives	Carboxylic acids and derivatives	Amino acids, peptides, and alogues	Amino acids and derivatives	Alpha amino acids and derivatives	Alpha amino acids	L-alpha-amino acids	TRUE	TRUE	TRUE	FALSE	TRUE	FALSE	TRUE	0	4	0	FALSE	FALSE
arg_L	InChI=1S/C6H14N4O2/c7-4(5(11)12)2-1-3-10-6(8)9/h4H,1-	Organic compounds	Organic acids and derivatives	Carboxylic acids and derivatives	Amino acids, peptides, and alogues	Amino acids and derivatives	Alpha amino acids	Alpha amino acids	L-alpha-amino acids	TRUE	TRUE	TRUE	TRUE	TRUE	FALSE	FALSE	1	1	1	TRUE	FALSE

	3,7H2,(H,11,12)(H4,8,9,10)/p+1/t4-/m0/s1						and derivatives															
citr_L	InChI=1S/C6H13N3O3/c7-4(5(10)11)2-1-3-9-6(8)12/h4H,1-3,7H2,(H,10,11)(H3,8,9,12)/t4-/m0/s1	Organic compounds	Organic acids and derivatives	Carboxylic acids and derivatives	Amino acids, peptides, and alogues	Amino acids and derivatives	Alpha amino acids and derivatives	Alpha amino acids	L-alpha-amino acids		TRUE	TRUE	TRUE	FALSE	TRUE	FALSE	TRUE	0	3	0	FALSE	FALSE
gln_L	InChI=1S/C5H10N2O3/c6-3(5(9)10)1-2-4(7)8/h3H,1-2,6H2,(H2,7,8)(H,9,10)/t3-/m0/s1	Organic compounds	Organic acids and derivatives	Carboxylic acids and derivatives	Amino acids, peptides, and alogues	Amino acids and derivatives	Alpha amino acids and derivatives	Alpha amino acids	L-alpha-amino acids		TRUE	TRUE	TRUE	TRUE	TRUE	TRUE	FALSE	8	1	1	TRUE	FALSE
hcys_L	InChI=1S/C4H9NO2S/c5-3(1-2-8)4(6)7/h3,8H,1-2,5H2,(H,6,7)/t3-/m0/s1	Organic compounds	Organic acids and derivatives	Carboxylic acids and derivatives	Amino acids, peptides, and alogues	Amino acids and derivatives	Alpha amino acids and derivatives	Alpha amino acids	L-alpha-amino acids		TRUE	TRUE	TRUE	TRUE	FALSE	FALSE	FALSE	1	0	0	FALSE	FALSE
lys_L	InChI=1S/C6H14N2O2/c7-4-2-1-3-5(8)6(9)10/h5H,1-4,7-8H2,(H,9,10)/p+1/t5-/m0/s1	Organic compounds	Organic acids and derivatives	Carboxylic acids and derivatives	Amino acids, peptides, and alogues	Amino acids and derivatives	Alpha amino acids and derivatives	Alpha amino acids	L-alpha-amino acids		TRUE	TRUE	TRUE	TRUE	TRUE	TRUE	TRUE	4	4	1	TRUE	FALSE
metsox_S_L	InChI=1S/C5H11NO3S/c1-10(9)3-2-4(6)5(7)8/h4H,2-3,6H2,1H3,(H,7,8)/t4-,10?/m0/s1	Organic compounds	Organic acids and derivatives	Carboxylic acids and derivatives	Amino acids, peptides, and alogues	Amino acids and derivatives	Alpha amino acids and derivatives	Alpha amino acids	L-alpha-amino acids		FALSE	FALSE	TRUE	TRUE	TRUE	FALSE	FALSE	1	1	1	FALSE	FALSE
orn	InChI=1S/C5H12N2O2/c6-3-1-2-4(7)5(8)9/h4H,1-3,6-7H2,(H,8,9)/p+1/t4-/m0/s1	Organic compounds	Organic acids and derivatives	Carboxylic acids and derivatives	Amino acids, peptides, and alogues	Amino acids and derivatives	Alpha amino acids and derivatives	Alpha amino acids	L-alpha-amino acids		TRUE	TRUE	TRUE	TRUE	TRUE	TRUE	FALSE	10	1	1	TRUE	FALSE
441450	InChI=1S/C7H16N4O3/c8-5(6(13)14)3-4(12)1-2-11-7(9)10/h4-5,12H,1-3,8H2,(H,13,14)(H4,9,10,11)/t4-,5+/m1/s1	Organic compounds	Organic acids and derivatives	Carboxylic acids and derivatives	Amino acids, peptides, and alogues	Amino acids and derivatives	Alpha amino acids and derivatives	Alpha amino acids	L-alpha-amino acids	Alpha amino acids which have the L-configuration of the alpha-carbon atom.	FALSE	FALSE	FALSE	TRUE	FALSE	FALSE	FALSE	1	0	0	FALSE	FALSE
thr_L	InChI=1S/C4H9NO3/c1-2(6)3(5)4(7)8/h2-3,6H,5H2,1H3,(H,7,8)/t2-,3+/m1/s1	Organic compounds	Organic acids and derivatives	Carboxylic acids and derivatives	Amino acids, peptides, and alogues	Amino acids and derivatives	Alpha amino acids and derivatives	Alpha amino acids	L-alpha-amino acids	None	TRUE	TRUE	TRUE	TRUE	TRUE	TRUE	FALSE	5	1	1	FALSE	FALSE
dmlarg	InChI=1S/C8H18N4O2/c1-12(2)8(10)11-5-3-4-6(9)7(13)14/h6H,3-5,9H2,1-2H3,(H2,10,11)(H,13,14)/t6-/m0/s1	Organic compounds	Organic acids and derivatives	Carboxylic acids and derivatives	Amino acids, peptides, and alogues	Amino acids and derivatives	Alpha amino acids and derivatives	Arginine and derivatives			TRUE	FALSE	TRUE	TRUE	TRUE	FALSE	FALSE	1	1	1	FALSE	FALSE
22833646	InChI=1S/C10H19N3O5/c11-6(9(15)16)3-1-2-4-13-8(14)5-7(12)10(17)18/h6-7H,1-5,11-12H2,(H,13,14)(H,15,16)(H,17,18)/t6-,7-/m0/s1	Organic compounds	Organic acids and derivatives	Carboxylic acids and derivatives	Amino acids, peptides, and alogues	Amino acids and derivatives	Alpha amino acids and derivatives	Asparagine and derivatives			FALSE	FALSE	FALSE	TRUE	FALSE	FALSE	FALSE	1	0	0	FALSE	FALSE
asn_L	InChI=1S/C4H8N2O3/c5-2(4(8)9)1-3(6)7/h2H,1,5H2,(H2,6,7)(H,8,9)/t2-/m0/s1	Organic compounds	Organic acids and derivatives	Carboxylic acids and derivatives	Amino acids, peptides, and alogues	Amino acids and derivatives	Alpha amino acids and derivatives	Asparagine and derivatives			TRUE	TRUE	TRUE	TRUE	TRUE	TRUE	TRUE	2	2	1	TRUE	FALSE
24891369	InChI=1S/C13H13NO6/c15-9-4-1-8(2-5-9)3-6-11(16)14-10(13(19)20)7-12(17)18/h1-6,10,15H,7H2,(H,14,16)(H,17,18)(H,19,20)/b6-3+	Organic compounds	Organic acids and derivatives	Carboxylic acids and derivatives	Amino acids, peptides, and alogues	Amino acids and derivatives	Alpha amino acids and derivatives	Aspartic acid and derivatives			FALSE	FALSE	FALSE	FALSE	TRUE	FALSE	FALSE	0	1	0	FALSE	FALSE
439918	InChI=1S/C5H9N3O4/c6-5(7)8-2(4(11)12)1-3(9)10/h2H,1H2,(H,9,10)(H,11,12)(H	Organic compounds	Organic acids and derivatives	Carboxylic acids and derivatives	Amino acids, peptides, and alogues	Amino acids and derivatives	Alpha amino acids and	Aspartic acid and derivatives			FALSE	FALSE	FALSE	TRUE	FALSE	FALSE	FALSE	1	0	0	FALSE	FALSE

	4,6,7,8)/t2-/m0/s1						derivatives													
asp_L	InChI=1S/C4H7NO4/c5-2(4(8)9)1-3(6)7/h2H,1,5H2,(H,6,7)(H,8,9)/p-1/t2-/m0/s1	Organic compounds	Organic acids and derivatives	Carboxylic acids and derivatives	Amino acids, peptides, and alogues	Amino acids and derivatives	Alpha amino acids and derivatives	Aspartic acid and derivatives	TRUE	TRUE	TRUE	TRUE	TRUE	TRUE	FALSE	3	1	1	TRUE	FALSE
1080	InChI=1S/C5H9NO4S/c6-3(5(9)10)1-11-2-4(7)8/h3H,1-2,6H2,(H,7,8)(H,9,10)	Organic compounds	Organic acids and derivatives	Carboxylic acids and derivatives	Amino acids, peptides, and alogues	Amino acids and derivatives	Alpha amino acids and derivatives	Cysteine and derivatives	FALSE	FALSE	FALSE	FALSE	TRUE	FALSE	FALSE	0	1	0	FALSE	FALSE
Lcystin	InChI=1S/C6H12N2O4S2/c7-3(5(9)10)1-13-14-2-4(8)6(11)12/h3-4H,1-2,7-8H2,(H,9,10)(H,11,12)	Organic compounds	Organic acids and derivatives	Carboxylic acids and derivatives	Amino acids, peptides, and alogues	Amino acids and derivatives	Alpha amino acids and derivatives	Cysteine and derivatives	TRUE	TRUE	TRUE	TRUE	FALSE	FALSE	FALSE	1	0	0	FALSE	FALSE
cys_L	InChI=1S/C3H7NO2S/c4-2(1-7)3(5)6/h2,7H,1,4H2,(H,5,6)/t2-/m0/s1	Organic compounds	Organic acids and derivatives	Carboxylic acids and derivatives	Amino acids, peptides, and alogues	Amino acids and derivatives	Alpha amino acids and derivatives	Cysteine and derivatives	TRUE	TRUE	TRUE	TRUE	FALSE	TRUE	FALSE	2	0	0	TRUE	FALSE
cyst_L	InChI=1S/C7H14N2O4S/c8-4(6(10)11)1-2-14-3-5(9)7(12)13/h4-5H,1-3,8-9H2,(H,10,11)(H,12,13)	Organic compounds	Organic acids and derivatives	Carboxylic acids and derivatives	Amino acids, peptides, and alogues	Amino acids and derivatives	Alpha amino acids and derivatives	Cysteine and derivatives	TRUE	TRUE	TRUE	TRUE	FALSE	TRUE	FALSE	2	0	0	FALSE	FALSE
acglu	InChI=1S/C7H11NO5/c1-4(9)8-5(7(12)13)2-3-6(10)11/h5H,2-3H2,1H3,(H,8,9)(H,10,11)(H,12,13)/t5-/m0/s1	Organic compounds	Organic acids and derivatives	Carboxylic acids and derivatives	Amino acids, peptides, and alogues	Amino acids and derivatives	Alpha amino acids and derivatives	Glutamic acid and derivatives	TRUE	TRUE	TRUE	TRUE	FALSE	FALSE	FALSE	1	0	0	FALSE	FALSE
glu_L	InChI=1S/C5H9NO4/c6-3(5(9)10)1-2-4(7)8/h3H,1-2,6H2,(H,7,8)(H,9,10)/p-1/t3-/m0/s1	Organic compounds	Organic acids and derivatives	Carboxylic acids and derivatives	Amino acids, peptides, and alogues	Amino acids and derivatives	Alpha amino acids and derivatives	Glutamic acid and derivatives	TRUE	TRUE	TRUE	TRUE	TRUE	TRUE	TRUE	2	6	1	TRUE	FALSE
25200879	InChI=1S/C15H17N3O4/c16-13(19)6-5-12(15(21)22)18-14(20)7-9-8-17-11-4-2-1-3-10(9)11/h1-4,8,12,17H,5-7H2,(H2,16,19)(H,18,20)(H,21,22)	Organic compounds	Organic acids and derivatives	Carboxylic acids and derivatives	Amino acids, peptides, and alogues	Amino acids and derivatives	Alpha amino acids and derivatives	Glutamine and derivatives	FALSE	FALSE	FALSE	FALSE	TRUE	FALSE	FALSE	0	1	0	FALSE	FALSE
28880746	InChI=1S/C11H20N2O4/c1-2-3-4-5-10(15)13-8(11(16)17)6-7-9(12)14/h8H,2-7H2,1H3,(H2,12,14)(H,13,15)(H,16,17)/t8-/m0/s1	Organic compounds	Organic acids and derivatives	Carboxylic acids and derivatives	Amino acids, peptides, and alogues	Amino acids and derivatives	Alpha amino acids and derivatives	Glutamine and derivatives	FALSE	FALSE	FALSE	TRUE	FALSE	FALSE	FALSE	1	0	0	FALSE	FALSE
3032311	InChI=1S/C9H15N3O2S/c1-12(2,3)7(8(13)14)4-6-5-10-9(15)11-6/h5,7H,4H2,1-3H3,(H2-,10,11,13,14,15)	Organic compounds	Organic acids and derivatives	Carboxylic acids and derivatives	Amino acids, peptides, and alogues	Amino acids and derivatives	Alpha amino acids and derivatives	Histidine and derivatives	FALSE	FALSE	FALSE	FALSE	TRUE	FALSE	FALSE	0	1	0	FALSE	FALSE
3mhis	InChI=1S/C7H11N3O2/c1-10-4-9-3-5(10)2-6(8)7(11)12/h3-4,6H,2,8H2,1H3,(H,11,12)/t6-/m0/s1	Organic compounds	Organic acids and derivatives	Carboxylic acids and derivatives	Amino acids, peptides, and alogues	Amino acids and derivatives	Alpha amino acids and derivatives	Histidine and derivatives	TRUE	TRUE	TRUE	FALSE	TRUE	FALSE	TRUE	0	2	0	FALSE	FALSE
75619	InChI=1S/C8H11N3O3/c1-5(12)11-7(8(13)14)2-6-3-9-4-10-6/h3-4,7H,2H2,1H3,(H,9,10)(H,11,12)(H,13,14)/t7-/m0/s1	Organic compounds	Organic acids and derivatives	Carboxylic acids and derivatives	Amino acids, peptides, and alogues	Amino acids and derivatives	Alpha amino acids and derivatives	Histidine and derivatives	FALSE	FALSE	FALSE	FALSE	TRUE	FALSE	FALSE	0	1	0	FALSE	FALSE
his_L	InChI=1S/C6H9N3O2/c7-5(6(10)11)1-4-2-8-3-9-4/h2-3,5H,1,7H2,(H,8,9)(H,10,11)/t5-/m0/s1	Organic compounds	Organic acids and derivatives	Carboxylic acids and derivatives	Amino acids, peptides, and alogues	Amino acids and derivatives	Alpha amino acids and derivatives	Histidine and derivatives	TRUE	TRUE	TRUE	TRUE	FALSE	TRUE	FALSE	5	0	0	TRUE	FALSE
52922064	InChI=1S/C26H43NO3/c1-4-6-7-8-9-10-11-12-13-14-15-16-17-18-19-20-21-22-24(28)27-25(26(29)	Organic compounds	Organic acids and derivatives	Carboxylic acids and derivatives	Amino acids, peptides, and alogues	Amino acids and derivatives	Alpha amino acids and derivativ	Isoleucine and derivatives	FALSE	FALSE	FALSE	FALSE	TRUE	FALSE	FALSE	0	1	0	FALSE	FALSE

	30)23(3)5-2/h9-10,12-13,15-16,18-19,23,25H,4-8,11,14,17,20-22H2,1-3H3,(H,27,28)(H,29,30)/b10-9-,13-12-,16-15-,19-18-/t23-,25-/m0/s1						es														
ile_L	InChI=1S/C6H13NO2/c1-3-4(2)5(7)6(8)9/h4-5H,3,7H2,1-2H3,(H,8,9)/t4-,5-/m0/s1	Organic compounds	Organic acids and derivatives	Carboxylic acids and derivatives	Amino acids, peptides, and alogues	Amino acids and derivatives	Alpha amino acids and derivatives	Isoleucine and derivatives		TRUE	TRUE	TRUE	TRUE	TRUE	TRUE	FALSE	6	1	1	TRUE	FALSE
leu_L	InChI=1S/C6H13NO2/c1-4(2)3-5(7)6(8)9/h4-5H,3,7H2,1-2H3,(H,8,9)/t5-/m0/s1	Organic compounds	Organic acids and derivatives	Carboxylic acids and derivatives	Amino acids, peptides, and alogues	Amino acids and derivatives	Alpha amino acids and derivatives	Leucine and derivatives		TRUE	TRUE	TRUE	TRUE	TRUE	TRUE	TRUE	4	4	1	TRUE	FALSE
439750	InChI=1S/C6H11NO3S/c1-11-3-2-5(6(9)10)7-4-8/h4-5H,2-3H2,1H3,(H,7,8)(H,9,10)/t5-/m0/s1	Organic compounds	Organic acids and derivatives	Carboxylic acids and derivatives	Amino acids, peptides, and alogues	Amino acids and derivatives	Alpha amino acids and derivatives	Methionine and derivatives		FALSE	FALSE	FALSE	FALSE	TRUE	FALSE	FALSE	0	1	0	FALSE	FALSE
C02712	InChI=1S/C7H13NO3S/c1-5(9)8-6(7(10)11)3-4-12-2/h6H,3-4H2,1-2H3,(H,8,9)(H,10,11)/p-1/t6-/m0/s1	Organic compounds	Organic acids and derivatives	Carboxylic acids and derivatives	Amino acids, peptides, and alogues	Amino acids and derivatives	Alpha amino acids and derivatives	Methionine and derivatives		TRUE	TRUE	TRUE	FALSE	TRUE	FALSE	FALSE	0	1	0	FALSE	FALSE
met_L	InChI=1S/C5H11NO2S/c1-9-3-2-4(6)5(7)8/h4H,2-3,6H2,1H3,(H,7,8)/t4-/m0/s1	Organic compounds	Organic acids and derivatives	Carboxylic acids and derivatives	Amino acids, peptides, and alogues	Amino acids and derivatives	Alpha amino acids and derivatives	Methionine and derivatives		TRUE	TRUE	TRUE	TRUE	TRUE	TRUE	TRUE	3	2	1	TRUE	FALSE
23667628	InChI=1S/C19H17Cl2N3O5S.Na/c1-7-10(12(23-29-7)11-8(20)5-4-6-9(11)21)15(25)22-13-16(26)24-14(18(27)28)19(2,3)30-17(13)24;/h4-6,13-14,17H,1-3H3,(H,22,25)(H,27,28);/q;+1/p-1/t13-,14+,17-;/m1./s1	Organic compounds	Organic acids and derivatives	Carboxylic acids and derivatives	Amino acids, peptides, and alogues	Amino acids and derivatives	Alpha amino acids and derivatives	N-acyl-alpha amino acids and derivatives		FALSE	FALSE	FALSE	FALSE	TRUE	FALSE	FALSE	0	1	0	FALSE	FALSE
102807	InChI=1S/C8H16N4O3/c1-5(13)12-6(7(14)15)3-2-4-11-8(9)10/h6H,2-4H2,1H3,(H,12,13)(H,14,15)(H4,9,10,11)	Organic compounds	Organic acids and derivatives	Carboxylic acids and derivatives	Amino acids, peptides, and alogues	Amino acids and derivatives	Alpha amino acids and derivatives	N-acyl-alpha amino acids and derivatives	N-acyl-alpha amino acids	FALSE	FALSE	FALSE	TRUE	FALSE	FALSE	FALSE	1	0	0	FALSE	FALSE
151008	InChI=1S/C18H35NO3/c1-2-3-4-5-6-7-8-9-10-11-12-13-14-15-17(20)19-16-18(21)22/h2-16H2,1H3,(H,19,20)(H,21,22)	Organic compounds	Organic acids and derivatives	Carboxylic acids and derivatives	Amino acids, peptides, and alogues	Amino acids and derivatives	Alpha amino acids and derivatives	N-acyl-alpha amino acids and derivatives	N-acyl-alpha amino acids	FALSE	FALSE	FALSE	TRUE	FALSE	FALSE	FALSE	1	0	0	FALSE	FALSE
21863	InChI=1S/C7H7NO4/c9-6(10)4-8-7(11)5-2-1-3-12-5/h1-3H,4H2,(H,8,11)(H,9,10)	Organic compounds	Organic acids and derivatives	Carboxylic acids and derivatives	Amino acids, peptides, and alogues	Amino acids and derivatives	Alpha amino acids and derivatives	N-acyl-alpha amino acids and derivatives	N-acyl-alpha amino acids	FALSE	FALSE	FALSE	TRUE	TRUE	TRUE	FALSE	2	1	1	FALSE	FALSE
346152	InChI=1S/C14H27NO3/c1-2-3-4-5-6-7-8-9-10-11-13(16)15-12-14(17)18/h2-12H2,1H3,(H,15,16)(H,17,18)	Organic compounds	Organic acids and derivatives	Carboxylic acids and derivatives	Amino acids, peptides, and alogues	Amino acids and derivatives	Alpha amino acids and derivatives	N-acyl-alpha amino acids and derivatives	N-acyl-alpha amino acids	FALSE	FALSE	FALSE	FALSE	TRUE	FALSE	FALSE	0	1	0	FALSE	FALSE
440732	InChI=1S/C10H11NO4/c12-8-3-1-7(2-4-8)5-9(13)11-6-10(14)15/h1-4,12H,5-6H2,(H,11,13)(H,14,15)	Organic compounds	Organic acids and derivatives	Carboxylic acids and derivatives	Amino acids, peptides, and alogues	Amino acids and derivatives	Alpha amino acids and derivatives	N-acyl-alpha amino acids and derivatives	N-acyl-alpha amino acids	FALSE	FALSE	FALSE	TRUE	FALSE	FALSE	FALSE	1	0	0	FALSE	FALSE
M02723	InChI=1S/C10H11NO3/c12-9(11-7-10(13)14)6-8-4-2-1-3-5-8/h1-5H,6-7H2,(H,11,12)(H,13,14)	Organic compounds	Organic acids and derivatives	Carboxylic acids and derivatives	Amino acids, peptides, and alogues	Amino acids and derivatives	Alpha amino acids and derivatives	N-acyl-alpha amino acids and derivatives	N-acyl-alpha amino acids	TRUE	FALSE	TRUE	TRUE	FALSE	TRUE	FALSE	2	0	0	FALSE	FALSE
acgly	InChI=1S/C4H7NO3/c1-3(6)5-2-4(7)8/h2H2,1H3,(H,5,6)(H,7,8)	Organic compounds	Organic acids and derivatives	Carboxylic acids and derivatives	Amino acids, peptides, and alogues	Amino acids and derivatives	Alpha amino acids and derivativ	N-acyl-alpha amino acids and derivativ	N-acyl-alpha amino acids	TRUE	TRUE	TRUE	TRUE	FALSE	TRUE	FALSE	4	0	0	FALSE	FALSE

							es	es														
hexgly	InChI=1S/C8H15NO3/c1-2-3-4-5-7(10)9-6-8(11)12/h2-6H2,1H3,(H,9,10)(H,11,12)	Organic compounds	Organic acids and derivatives	Carboxylic acids and derivatives	Amino acids, peptides, and alogues	Amino acids and derivatives	Alpha amino acids and derivatives	N-acyl-alpha amino acids and derivatives	N-acyl-alpha amino acids		TRUE	FALSE	TRUE	TRUE	FALSE	TRUE	FALSE	3	0	0	FALSE	FALSE
pheacgln	InChI=1S/C13H16N2O4/c14-11(16)7-6-10(13(18)19)15-12(17)8-9-4-2-1-3-5-9/h1-5,10H,6-8H2,(H2,14,16)(H,15,17)(H,18,19)/t10-/m0/s1	Organic compounds	Organic acids and derivatives	Carboxylic acids and derivatives	Amino acids, peptides, and alogues	Amino acids and derivatives	Alpha amino acids and derivatives	N-acyl-alpha amino acids and derivatives	N-acyl-alpha amino acids		TRUE	FALSE	TRUE	TRUE	FALSE	TRUE	FALSE	5	0	0	FALSE	FALSE
tiggly	InChI=1S/C7H11NO3/c1-3-5(2)7(11)8-4-6(9)10/h3H,4H2,1-2H3,(H,8,11)(H,9,10)/b5-3+	Organic compounds	Organic acids and derivatives	Carboxylic acids and derivatives	Amino acids, peptides, and alogues	Amino acids and derivatives	Alpha amino acids and derivatives	N-acyl-alpha amino acids and derivatives	N-acyl-alpha amino acids		TRUE	FALSE	TRUE	TRUE	FALSE	TRUE	FALSE	2	0	0	FALSE	FALSE
65249	InChI=1S/C5H9NO4/c1-3(8)6-4(2-7)5(9)10/h4,7H,2H2,1H3,(H,6,8)(H,9,10)/t4-/m0/s1	Organic compounds	Organic acids and derivatives	Carboxylic acids and derivatives	Amino acids, peptides, and alogues	Amino acids and derivatives	Alpha amino acids and derivatives	N-acyl-alpha amino acids and derivatives	N-acyl-alpha amino acids	N-acyl-L-alpha-amino acids	FALSE	FALSE	FALSE	TRUE	TRUE	FALSE	FALSE	1	1	1	FALSE	FALSE
12314153	InChI=1S/C9H11NO3/c10-7(9(12)13)8(11)6-4-2-1-3-5-6/h1-5,7-8,11H,10H2,(H,12,13)/t7-,8?/m0/s1	Organic compounds	Organic acids and derivatives	Carboxylic acids and derivatives	Amino acids, peptides, and alogues	Amino acids and derivatives	Alpha amino acids and derivatives	Phenylalanine and derivatives			FALSE	FALSE	FALSE	FALSE	TRUE	FALSE	FALSE	0	1	0	FALSE	FALSE
52922060	InChI=1S/C31H41NO3/c1-2-3-4-5-6-7-8-9-10-11-12-13-14-15-16-17-18-19-23-26-30(33)32-29(31(34)35)27-28-24-21-20-22-25-28/h3-4,6-7,9-10,12-13,15-16,18-22,24-25,29H,2,5,8,11,14,17,23,26-27H2,1H3,(H,32,33)(H,34,35)/b4-3-,7-6-,10-9-,13-12-,16-15-,19-18-/t29-/m0/s1	Organic compounds	Organic acids and derivatives	Carboxylic acids and derivatives	Amino acids, peptides, and alogues	Amino acids and derivatives	Alpha amino acids and derivatives	Phenylalanine and derivatives			FALSE	FALSE	FALSE	TRUE	FALSE	FALSE	FALSE	1	0	0	FALSE	FALSE
74839	InChI=1S/C11H13NO3/c1-8(13)12-10(11(14)15)7-9-5-3-2-4-6-9/h2-6,10H,7H2,1H3,(H,12,13)(H,14,15)/t10-/m0/s1	Organic compounds	Organic acids and derivatives	Carboxylic acids and derivatives	Amino acids, peptides, and alogues	Amino acids and derivatives	Alpha amino acids and derivatives	Phenylalanine and derivatives			FALSE	FALSE	FALSE	TRUE	TRUE	TRUE	FALSE	2	1	1	FALSE	FALSE
phe_L	InChI=1S/C9H11NO2/c10-8(9(11)12)6-7-4-2-1-3-5-7/h1-5,8H,6,10H2,(H,11,12)/t8-/m0/s1	Organic compounds	Organic acids and derivatives	Carboxylic acids and derivatives	Amino acids, peptides, and alogues	Amino acids and derivatives	Alpha amino acids and derivatives	Phenylalanine and derivatives			TRUE	TRUE	TRUE	TRUE	TRUE	TRUE	TRUE	8	3	1	TRUE	FALSE
557	InChI=1S/C6H11NO2/c1-7-4-2-3-5(7)6(8)9/h5H,2-4H2,1H3,(H,8,9)	Organic compounds	Organic acids and derivatives	Carboxylic acids and derivatives	Amino acids, peptides, and alogues	Amino acids and derivatives	Alpha amino acids and derivatives	Proline and derivatives			FALSE	FALSE	FALSE	TRUE	FALSE	FALSE	FALSE	1	0	0	FALSE	FALSE
5810	InChI=1S/C5H9NO3/c7-3-1-4(5(8)9)6-2-3/h3-4,6-7H,1-2H2,(H,8,9)/t3-,4+/m1/s1	Organic compounds	Organic acids and derivatives	Carboxylic acids and derivatives	Amino acids, peptides, and alogues	Amino acids and derivatives	Alpha amino acids and derivatives	Proline and derivatives			FALSE	FALSE	FALSE	TRUE	FALSE	FALSE	FALSE	1	0	0	FALSE	FALSE
5oxpro	InChI=1S/C5H7NO3/c7-4-2-1-3(6-4)5(8)9/h3H,1-2H2,(H,6,7)(H,8,9)/p-1/t3-/m0/s1	Organic compounds	Organic acids and derivatives	Carboxylic acids and derivatives	Amino acids, peptides, and alogues	Amino acids and derivatives	Alpha amino acids and derivatives	Proline and derivatives			TRUE	TRUE	TRUE	TRUE	TRUE	TRUE	TRUE	4	2	1	FALSE	FALSE
pro_L	InChI=1S/C5H9NO2/c7-5(8)4-2-1-3-6-4/h4,6H,1-3H2,(H,7,8)/t4-/m0/s1	Organic compounds	Organic acids and derivatives	Carboxylic acids and derivatives	Amino acids, peptides, and alogues	Amino acids and derivatives	Alpha amino acids and derivatives	Proline and derivatives			TRUE	TRUE	TRUE	TRUE	FALSE	TRUE	FALSE	6	0	0	FALSE	FALSE
ser_L	InChI=1S/C3H7NO3/c4-2(1-5)3(6)7/h2,5H,1,4H2,(H,6,7)/t2-/m0/s1	Organic compounds	Organic acids and derivatives	Carboxylic acids and derivatives	Amino acids, peptides, and alogues	Amino acids and derivatives	Alpha amino acids and derivativ	Serine and derivatives			TRUE	TRUE	TRUE	TRUE	TRUE	TRUE	TRUE	2	2	1	FALSE	FALSE

							es													
34dphe	InChI=1S/C9H11NO4/c10-6(9(13)14)3-5-1-2-7(11)8(12)4-5/h1-2,4,6,11-12H,3,10H2,(H,13,14)/t6-/m0/s1	Organic compounds	Organic acids and derivatives	Carboxylic acids and derivatives	Amino acids, peptides, and alogues	Amino acids and derivatives	Alpha amino acids and derivatives	Tyrosine and derivatives	TRUE	TRUE	TRUE	TRUE	TRUE	FALSE	FALSE	1	1	1	FALSE	FALSE
94340	InChI=1S/C14H18N2O6/c15-10(13(19)20)5-6-12(18)16-11(14(21)22)7-8-1-3-9(17)4-2-8/h1-4,10-11,17H,5-7,15H2,(H,16,18)(H,19,20)(H,21,22)/t10-,11-/m0/s1	Organic compounds	Organic acids and derivatives	Carboxylic acids and derivatives	Amino acids, peptides, and alogues	Amino acids and derivatives	Alpha amino acids and derivatives	Tyrosine and derivatives	FALSE	FALSE	FALSE	TRUE	FALSE	FALSE	FALSE	1	0	0	FALSE	FALSE
CE2176	InChI=1S/C10H13NO4/c1-15-9-5-6(2-3-8(9)12)4-7(11)10(13)14/h2-3,5,7,12H,4,11H2,1H3,(H,13,14)	Organic compounds	Organic acids and derivatives	Carboxylic acids and derivatives	Amino acids, peptides, and alogues	Amino acids and derivatives	Alpha amino acids and derivatives	Tyrosine and derivatives	TRUE	FALSE	TRUE	TRUE	FALSE	TRUE	FALSE	8	0	0	FALSE	FALSE
actyr	InChI=1S/C11H13NO4/c1-7(13)12-10(11(15)16)6-8-2-4-9(14)5-3-8/h2-5,10,14H,6H2,1H3,(H,12,13)(H,15,16)/t10-/m0/s1	Organic compounds	Organic acids and derivatives	Carboxylic acids and derivatives	Amino acids, peptides, and alogues	Amino acids and derivatives	Alpha amino acids and derivatives	Tyrosine and derivatives	TRUE	TRUE	TRUE	TRUE	FALSE	FALSE	FALSE	1	0	0	FALSE	FALSE
tyr_L	InChI=1S/C9H11NO3/c10-8(9(12)13)5-6-1-3-7(11)4-2-6/h1-4,8,11H,5,10H2,(H,12,13)/t8-/m0/s1	Organic compounds	Organic acids and derivatives	Carboxylic acids and derivatives	Amino acids, peptides, and alogues	Amino acids and derivatives	Alpha amino acids and derivatives	Tyrosine and derivatives	TRUE	TRUE	TRUE	TRUE	TRUE	TRUE	TRUE	10	3	1	TRUE	FALSE
val_L	InChI=1S/C5H11NO2/c1-3(2)4(6)5(7)8/h3-4H,6H2,1-2H3,(H,7,8)/t4-/m0/s1	Organic compounds	Organic acids and derivatives	Carboxylic acids and derivatives	Amino acids, peptides, and alogues	Amino acids and derivatives	Alpha amino acids and derivatives	Valine and derivatives	TRUE	TRUE	TRUE	TRUE	TRUE	TRUE	FALSE	2	1	1	TRUE	FALSE
10932	InChI=1S/C4H9NO2/c1-3(5)2-4(6)7/h3H,2,5H2,1H3,(H,6,7)	Organic compounds	Organic acids and derivatives	Carboxylic acids and derivatives	Amino acids, peptides, and alogues	Amino acids and derivatives	Beta amino acids and derivatives		FALSE	FALSE	FALSE	TRUE	FALSE	FALSE	FALSE	1	0	0	FALSE	FALSE
ala_B	InChI=1S/C3H7NO2/c4-2-1-3(5)6/h1-2,4H2,(H,5,6)	Organic compounds	Organic acids and derivatives	Carboxylic acids and derivatives	Amino acids, peptides, and alogues	Amino acids and derivatives	Beta amino acids and derivatives		TRUE	TRUE	TRUE	TRUE	FALSE	FALSE	FALSE	1	0	0	FALSE	FALSE
16759346	InChI=1S/C26H39NO3/c1-2-3-4-5-6-7-8-9-10-11-12-13-14-15-16-17-18-19-20-22-25(28)27-24-21-23-26(29)30/h3-4,6-7,9-10,12-13,15-16,18-19H,2,5,8,11,14,17,20-24H2,1H3,(H,27,28)(H,29,30)/b4-3-,7-6-,10-9-,13-12-,16-15-,19-18-	Organic compounds	Organic acids and derivatives	Carboxylic acids and derivatives	Amino acids, peptides, and alogues	Amino acids and derivatives	Gamma amino acids and derivatives		FALSE	FALSE	FALSE	TRUE	FALSE	FALSE	FALSE	1	0	0	FALSE	FALSE
4aabutn	InChI=1S/C6H11NO3/c1-5(8)7-4-2-3-6(9)10/h2-4H2,1H3,(H,7,8)(H,9,10)/p-1	Organic compounds	Organic acids and derivatives	Carboxylic acids and derivatives	Amino acids, peptides, and alogues	Amino acids and derivatives	Gamma amino acids and derivatives		TRUE	TRUE	TRUE	TRUE	TRUE	FALSE	FALSE	1	1	1	FALSE	FALSE
4abut	InChI=1S/C4H9NO2/c5-3-1-2-4(6)7/h1-3,5H2,(H,6,7)	Organic compounds	Organic acids and derivatives	Carboxylic acids and derivatives	Amino acids, peptides, and alogues	Amino acids and derivatives	Gamma amino acids and derivatives		TRUE	TRUE	TRUE	TRUE	FALSE	TRUE	FALSE	4	0	0	FALSE	FALSE
101179	InChI=1S/C8H12N4O3/c9-6(1-5-2-10-4-12-5)8(15)11-3-7(13)14/h2,4,6H,1,3,9H2,(H,10,12)(H,11,15)(H,13,14)/t6-/m0/s1	Organic compounds	Organic acids and derivatives	Carboxylic acids and derivatives	Amino acids, peptides, and alogues	Peptides			FALSE	FALSE	FALSE	TRUE	FALSE	FALSE	FALSE	1	0	0	FALSE	FALSE
123804	InChI=1S/C15H23N5O4/c16-11(8-9-3-5-10(21)6-4-9)13(22)20-12(14(23)24)2-1-7-19-15(17)18/h3-6,11-12,21H,1-2,7-8,1	Organic compounds	Organic acids and derivatives	Carboxylic acids and derivatives	Amino acids, peptides, and alogues	Peptides			FALSE	FALSE	FALSE	TRUE	FALSE	FALSE	FALSE	1	0	0	FALSE	FALSE

	6H2,(H,20,22)(H,23,24)(H4,17,18,19)/t11-,12-/m0/s1																	
145454816	InChI=1S/C15H26N6O4S/c16-4-2-1-3-11(20-13(22)10(17)7-26)14(23)21-12(15(24)25)5-9-6-18-8-19-9/h6,8,10-12,26H,1-5,7,16-17H2,(H,18,19)(H,20,22)(H,21,23)(H,24,25)/t10-,11-,12-/m0/s1	Organic compounds	Organic acids and derivatives	Carboxylic acids and derivatives	Amino acids, peptides, and alogues	Peptides	FALSE	FALSE	FALSE	FALSE	TRUE	FALSE	FALSE	0	1	0	FALSE	FALSE
145456541	InChI=1S/C28H33N5O4/c1-16(2)11-21(29)26(34)32-24(12-17-14-30-22-9-5-3-7-19(17)22)27(35)33-25(28(36)37)13-18-15-31-23-10-6-4-8-20(18)23/h3-10,14-16,21,24-25,30-31H,11-13,29H2,1-2H3,(H,32,34)(H,33,35)(H,36,37)/t21-,24-,25-/m0/s1	Organic compounds	Organic acids and derivatives	Carboxylic acids and derivatives	Amino acids, peptides, and alogues	Peptides	FALSE	FALSE	FALSE	FALSE	TRUE	FALSE	FALSE	0	1	0	FALSE	FALSE
145456674	InChI=1S/C17H30N6O4S/c1-28-7-5-13(17(26)27)22-16(25)14(8-11-9-20-10-21-11)23-15(24)12(19)4-2-3-6-18/h9-10,12-14H,2-8,18-19H2,1H3,(H,20,21)(H,22,25)(H,23,24)(H,26,27)/t12-,13-,14-/m0/s1	Organic compounds	Organic acids and derivatives	Carboxylic acids and derivatives	Amino acids, peptides, and alogues	Peptides	FALSE	FALSE	FALSE	TRUE	FALSE	FALSE	FALSE	1	0	0	FALSE	FALSE
85328684	InChI=1S/C43H63N11O13/c1-21(2)12-30(39(62)52-32(43(66)67)13-22(3)4)51-42(65)34(19-56)53-36(59)23(5)48-41(64)33(18-55)54-38(61)29(10-11-35(57)58)49-40(63)31(14-24-16-46-28-9-7-6-8-26(24)28)50-37(60)27(44)15-25-17-45-20-47-25/h6-9,16-17,20-23,27,29-34,46,55-56H,10-15,18-19,44H2,1-5H3,(H,45,47)(H,48,64)(H,49,63)(H,50,60)(H,51,65)(H,52,62)(H,53,59)(H,54,61)(H,57,58)(H,66,67)	Organic compounds	Organic acids and derivatives	Carboxylic acids and derivatives	Amino acids, peptides, and alogues	Peptides	FALSE	FALSE	FALSE	FALSE	TRUE	FALSE	TRUE	0	2	0	FALSE	FALSE
93078	InChI=1S/C13H16N2O5/c14-9(7-11(16)17)12(18)15-10(13(19)20)6-8-4-2-1-3-5-8/h1-5,9-10H,6-7,14H2,(H,15,18)(H,16,17)(H,19,20)/t9-,10-/m0/s1	Organic compounds	Organic acids and derivatives	Carboxylic acids and derivatives	Amino acids, peptides, and alogues	Peptides	FALSE	FALSE	FALSE	FALSE	TRUE	FALSE	FALSE	0	1	0	FALSE	FALSE
CE2890	InChI=1S/C18H37N9O4/c1-3-10(2)13(16(30)31)27-15(29)12(7-5-9-25-18(22)23)26-14(28)11(19)6-4-8-24-17(20)21/h10-13H,3-9,19H2,1-2H3,(H,26,28)(H,27,29)(H,30,31)(H4,20,21,24)(H4,22,23,25)/p+2/t10-,11-,12+,13-/m1/s1	Organic compounds	Organic acids and derivatives	Carboxylic acids and derivatives	Amino acids, peptides, and alogues	Peptides	TRUE	FALSE	TRUE	TRUE	FALSE	FALSE	FALSE	1	0	0	FALSE	FALSE
gthox	InChI=1S/C20H32N6O12S2/c21-9(19(35)36)1-3-13(27)25-11(17(33)23-5-15(29)30)7-39-40-8-12(18(34)24-6-16(31)32)26-14(28)4-2-10(22)20(37)38/h9-12H,1-8,21-22H2,(H,23,33)(H,24,34)(H,25,27)(H,26,28)(H,29,30)(H,31,32)(H,35,36)(H,	Organic compounds	Organic acids and derivatives	Carboxylic acids and derivatives	Amino acids, peptides, and alogues	Peptides	TRUE	TRUE	TRUE	FALSE	TRUE	FALSE	FALSE	0	1	0	FALSE	TRUE

	37,38)/t9-,10-,11-,12-/m0/s1																		
leuleu	InChI=1S/C12H24N2O3/c1-7(2)5-9(13)11(15)14-10(12(16)17)6-8(3)4/h7-10H,5-6,13H2,1-4H3,(H,14,15)(H,16,17)	Organic compounds	Organic acids and derivatives	Carboxylic acids and derivatives	Amino acids, peptides, and alogues	Peptides		TRUE	TRUE	TRUE	FALSE	TRUE	FALSE	TRUE	0	2	0	FALSE	FALSE
valval	InChI=1S/C10H20N2O3/c1-5(2)7(11)9(13)12-8(6(3)4)10(14)15/h5-8H,11H2,1-4H3,(H,12,13)(H,14,15)/t7-,8+/m0/s1	Organic compounds	Organic acids and derivatives	Carboxylic acids and derivatives	Amino acids, peptides, and alogues	Peptides		TRUE	TRUE	TRUE	FALSE	TRUE	FALSE	FALSE	0	1	0	FALSE	FALSE
11902892	InChI=1S/C10H16N2O4/c13-6-4-8(10(15)16)12(5-6)9(14)7-2-1-3-11-7/h6-8,11,13H,1-5H2,(H,15,16)/t6-,7+,8+/m1/s1	Organic compounds	Organic acids and derivatives	Carboxylic acids and derivatives	Amino acids, peptides, and alogues	Peptides	Dipeptides	FALSE	FALSE	FALSE	TRUE	FALSE	FALSE	FALSE	1	0	0	FALSE	FALSE
151023	InChI=1S/C11H20N2O5/c1-6(2)5-8(11(17)18)13-9(14)4-3-7(12)10(15)16/h6-8H,3-5,12H2,1-2H3,(H,13,14)(H,15,16)(H,17,18)/t7-,8-/m0/s1	Organic compounds	Organic acids and derivatives	Carboxylic acids and derivatives	Amino acids, peptides, and alogues	Peptides	Dipeptides	FALSE	FALSE	FALSE	FALSE	TRUE	FALSE	FALSE	0	1	0	FALSE	FALSE
152264	InChI=1S/C14H18N2O4/c17-10-5-3-9(4-6-10)8-12(14(19)20)16-13(18)11-2-1-7-15-11/h3-6,11-12,15,17H,1-2,7-8H2,(H,16,18)(H,19,20)/t11-,12-/m0/s1	Organic compounds	Organic acids and derivatives	Carboxylic acids and derivatives	Amino acids, peptides, and alogues	Peptides	Dipeptides	FALSE	FALSE	FALSE	TRUE	FALSE	FALSE	FALSE	1	0	0	FALSE	FALSE
1549440	InChI=1S/C7H13N3O4/c1-3(8)6(12)10-4(7(13)14)2-5(9)11/h3-4H,2,8H2,1H3,(H2,9,11)(H,10,12)(H,13,14)/t3-,4-/m0/s1	Organic compounds	Organic acids and derivatives	Carboxylic acids and derivatives	Amino acids, peptides, and alogues	Peptides	Dipeptides	FALSE	FALSE	FALSE	TRUE	FALSE	FALSE	FALSE	1	0	0	FALSE	FALSE
18218185	InChI=1S/C7H13N3O5/c8-3(1-5(9)12)6(13)10-4(2-11)7(14)15/h3-4,11H,1-2,8H2,(H2,9,12)(H,10,13)(H,14,15)	Organic compounds	Organic acids and derivatives	Carboxylic acids and derivatives	Amino acids, peptides, and alogues	Peptides	Dipeptides	FALSE	FALSE	FALSE	FALSE	TRUE	FALSE	FALSE	0	1	0	FALSE	FALSE
22833544	InChI=1S/C8H15N3O5S2/c9-4(7(14)11-1-6(12)13)2-17-18-3-5(10)8(15)16/h4-5H,1-3,9-10H2,(H,11,14)(H,12,13)(H,15,16)/t4?,5-/m0/s1	Organic compounds	Organic acids and derivatives	Carboxylic acids and derivatives	Amino acids, peptides, and alogues	Peptides	Dipeptides	FALSE	FALSE	FALSE	FALSE	TRUE	FALSE	FALSE	0	1	0	FALSE	FALSE
259325	InChI=1S/C15H22N2O3/c1-10(2)8-12(16)14(18)17-13(15(19)20)9-11-6-4-3-5-7-11/h3-7,10,12-13H,8-9,16H2,1-2H3,(H,17,18)(H,19,20)	Organic compounds	Organic acids and derivatives	Carboxylic acids and derivatives	Amino acids, peptides, and alogues	Peptides	Dipeptides	FALSE	FALSE	FALSE	FALSE	TRUE	FALSE	FALSE	0	1	0	FALSE	FALSE
2724807	InChI=1S/C7H14N2O3/c1-4(2)6(7(11)12)9-5(10)3-8/h4,6H,3,8H2,1-2H3,(H,9,10)(H,11,12)/t6-/m0/s1	Organic compounds	Organic acids and derivatives	Carboxylic acids and derivatives	Amino acids, peptides, and alogues	Peptides	Dipeptides	FALSE	FALSE	FALSE	TRUE	FALSE	FALSE	FALSE	1	0	0	FALSE	FALSE
3613616	InChI=1S/C8H14N2O6/c9-4(3-11)7(14)10-5(8(15)16)1-2-6(12)13/h4-5,11H,1-3,9H2,(H,10,14)(H,12,13)(H,15,16)	Organic compounds	Organic acids and derivatives	Carboxylic acids and derivatives	Amino acids, peptides, and alogues	Peptides	Dipeptides	FALSE	FALSE	FALSE	TRUE	FALSE	FALSE	FALSE	1	0	0	FALSE	FALSE
4198036	InChI=1S/C9H19N5O3/c1-5(8(16)17)14-7(15)6(10)3-2-4-13-9(11)12/h5-6H,2-4,10H2,1H3,(H,14,15)(H,16,17)(H4,11,12,13)	Organic compounds	Organic acids and derivatives	Carboxylic acids and derivatives	Amino acids, peptides, and alogues	Peptides	Dipeptides	FALSE	FALSE	FALSE	TRUE	FALSE	FALSE	FALSE	1	0	0	FALSE	FALSE
435949	InChI=1S/C11H22N2O3/c1-5-7(4)8(12)10(14)13-9(6(2)3)11(15)16/h6-9H,5,12H2,1-4H3,(H,13,14)(H,15,16)	Organic compounds	Organic acids and derivatives	Carboxylic acids and derivatives	Amino acids, peptides, and alogues	Peptides	Dipeptides	FALSE	FALSE	FALSE	FALSE	TRUE	FALSE	FALSE	0	1	0	FALSE	FALSE

515717	InChI=1S/C14H18N2O6/c15-10(5-6-12(18)19)13(20)16-11(14(21)22)7-8-1-3-9(17)4-2-8/h1-4,10-11,17H,5-7,15H2,(H,16,20)(H,18,19)(H,21,22)/t10-,11-/m0/s1	Organic compounds	Organic acids and derivatives	Carboxylic acids and derivatives	Amino acids, peptides, and alogues	Peptides	Dipeptides	FALSE	FALSE	FALSE	TRUE	FALSE	FALSE	FALSE	1	0	0	FALSE	FALSE
5246009	InChI=1S/C9H18N2O3/c1-4-5(2)7(10)8(12)11-6(3)9(13)14/h5-7H,4,10H2,1-3H3,(H,11,12)(H,13,14)	Organic compounds	Organic acids and derivatives	Carboxylic acids and derivatives	Amino acids, peptides, and alogues	Peptides	Dipeptides	FALSE	FALSE	FALSE	FALSE	TRUE	FALSE	FALSE	0	1	0	FALSE	FALSE
65104	InChI=1S/C18H20N2O3/c19-15(11-13-7-3-1-4-8-13)17(21)20-16(18(22)23)12-14-9-5-2-6-10-14/h1-10,15-16H,11-12,19H2,(H,20,21)(H,22,23)	Organic compounds	Organic acids and derivatives	Carboxylic acids and derivatives	Amino acids, peptides, and alogues	Peptides	Dipeptides	FALSE	FALSE	FALSE	FALSE	TRUE	FALSE	FALSE	0	1	0	FALSE	FALSE
65254	InChI=1S/C11H21N3O5/c12-6-2-1-3-8(11(18)19)14-9(15)5-4-7(13)10(16)17/h7-8H,1-6,12-13H2,(H,14,15)(H,16,17)(H,18,19)/t7-,8-/m0/s1	Organic compounds	Organic acids and derivatives	Carboxylic acids and derivatives	Amino acids, peptides, and alogues	Peptides	Dipeptides	FALSE	FALSE	FALSE	FALSE	TRUE	FALSE	FALSE	0	1	0	FALSE	FALSE
7015683	InChI=1S/C10H18N2O5/c1-5(2)8(10(16)17)12-7(13)4-3-6(11)9(14)15/h5-6,8H,3-4,11H2,1-2H3,(H,12,13)(H,14,15)(H,16,17)/t6-,8-/m0/s1	Organic compounds	Organic acids and derivatives	Carboxylic acids and derivatives	Amino acids, peptides, and alogues	Peptides	Dipeptides	FALSE	FALSE	FALSE	FALSE	TRUE	FALSE	FALSE	0	1	0	FALSE	FALSE
7016084	InChI=1S/C9H17N3O5/c1-4(13)7(11)8(15)12-5(9(16)17)2-3-6(10)14/h4-5,7,13H,2-3,11H2,1H3,(H2,10,14)(H,12,15)(H,16,17)/t4-,5+,7+/m1/s1	Organic compounds	Organic acids and derivatives	Carboxylic acids and derivatives	Amino acids, peptides, and alogues	Peptides	Dipeptides	FALSE	FALSE	FALSE	TRUE	FALSE	FALSE	FALSE	1	0	0	FALSE	FALSE
7017195	InChI=1S/C11H16N4O5/c12-7(10(17)18)1-2-9(16)15-8(11(19)20)3-6-4-13-5-14-6/h4-5,7-8H,1-3,12H2,(H,13,14)(H,15,16)(H,17,18)(H,19,20)/t7-,8-/m0/s1	Organic compounds	Organic acids and derivatives	Carboxylic acids and derivatives	Amino acids, peptides, and alogues	Peptides	Dipeptides	FALSE	FALSE	FALSE	FALSE	TRUE	FALSE	FALSE	0	1	0	FALSE	FALSE
9813855	InChI=1S/C11H20N2O5/c1-3-6(2)9(11(17)18)13-10(16)7(12)4-5-8(14)15/h6-7,9H,3-5,12H2,1-2H3,(H,13,16)(H,14,15)(H,17,18)/t6-,7-,9-/m0/s1	Organic compounds	Organic acids and derivatives	Carboxylic acids and derivatives	Amino acids, peptides, and alogues	Peptides	Dipeptides	FALSE	FALSE	FALSE	FALSE	TRUE	FALSE	FALSE	0	1	0	FALSE	FALSE
gluala	InChI=1S/C8H14N2O5/c1-4(7(12)13)10-6(11)3-2-5(9)8(14)15/h4-5H,2-3,9H2,1H3,(H,10,11)(H,12,13)(H,14,15)/p-1/t4-,5-/m0/s1	Organic compounds	Organic acids and derivatives	Carboxylic acids and derivatives	Amino acids, peptides, and alogues	Peptides	Dipeptides	TRUE	FALSE	TRUE	FALSE	TRUE	FALSE	FALSE	0	1	0	FALSE	FALSE
glumet	InChI=1S/C10H18N2O5S/c1-18-5-4-7(10(16)17)12-8(13)3-2-6(11)9(14)15/h6-7H,2-5,11H2,1H3,(H,12,13)(H,14,15)(H,16,17)/t6-,7-/m0/s1	Organic compounds	Organic acids and derivatives	Carboxylic acids and derivatives	Amino acids, peptides, and alogues	Peptides	Dipeptides	TRUE	TRUE	TRUE	FALSE	TRUE	FALSE	FALSE	0	1	0	FALSE	FALSE
glypro	InChI=1S/C7H12N2O3/c8-4-6(10)9-3-1-2-5(9)7(11)12/h5H,1-4,8H2,(H,11,12)/t5-/m0/s1	Organic compounds	Organic acids and derivatives	Carboxylic acids and derivatives	Amino acids, peptides, and alogues	Peptides	Dipeptides	TRUE	TRUE	TRUE	FALSE	TRUE	FALSE	FALSE	0	1	0	FALSE	FALSE
10276355	InChI=1S/C23H36N6O4/c24-11-5-3-8-17(26)21(30)28-19(10-4-6-12-25)22(31)29-20(23(32)33)13-15-14-27-18-9-2-1-7-16(15)18/h1-2,7,9,14,17,19-20,27H,3-6,8,10-13,24-26H2,(H,28,30)(H,29,31)(H,32,33)/t17-,19-,20-/m	Organic compounds	Organic acids and derivatives	Carboxylic acids and derivatives	Amino acids, peptides, and alogues	Peptides	Oligopeptides	FALSE	FALSE	FALSE	TRUE	FALSE	FALSE	FALSE	1	0	0	FALSE	FALSE

	0/s1																		
105044	InChI=1S/C44H61N11O10/c45-29(15-7-19-48-44(46)47)40(61)55-22-10-18-35(55)42(63)54-21-8-16-33(54)38(59)49-25-36(57)50-30(23-27-11-3-1-4-12-27)37(58)52-32(26-56)41(62)53-20-9-17-34(53)39(60)51-31(43(64)65)24-28-13-5-2-6-14-28/h1-6,11-14,29-35,56H,7-10,15-26,45H2,(H,49,59)(H,50,57)(H,51,60)(H,52,58)(H,64,65)(H4,46,47,48)/t29-,30-,31-,32-,33-,34-,35-/m0/s1	Organic compounds	Organic acids and derivatives	Carboxylic acids and derivatives	Amino acids, peptides, and alogues	Peptides	Oligopeptides	FALSE	FALSE	FALSE	TRUE	FALSE	FALSE	FALSE	1	0	0	FALSE	FALSE
5489007	InChI=1S/C10H17N3O6/c1-5(9(17)12-4-8(15)16)13-7(14)3-2-6(11)10(18)19/h5-6H,2-4,11H2,1H3,(H,12,17)(H,13,14)(H,15,16)(H,18,19)/t5-,6-/m0/s1	Organic compounds	Organic acids and derivatives	Carboxylic acids and derivatives	Amino acids, peptides, and alogues	Peptides	Oligopeptides	FALSE	FALSE	FALSE	TRUE	FALSE	FALSE	FALSE	1	0	0	FALSE	FALSE
HC02121	InChI=1S/C11H19N3O7S/c12-6(11(20)21)1-2-8(16)14-7(4-22-5-15)10(19)13-3-9(17)18/h6-7,15H,1-5,12H2,(H,13,19)(H,14,16)(H,17,18)(H,20,21)/p-1/t6-,7-/m0/s1	Organic compounds	Organic acids and derivatives	Carboxylic acids and derivatives	Amino acids, peptides, and alogues	Peptides	Oligopeptides	TRUE	FALSE	TRUE	TRUE	FALSE	FALSE	FALSE	1	0	0	FALSE	FALSE
gthrd	InChI=1S/C10H17N3O6S/c11-5(10(18)19)1-2-7(14)13-6(4-20)9(17)12-3-8(15)16/h5-6,20H,1-4,11H2,(H,12,17)(H,13,14)(H,15,16)(H,18,19)/p-1/t5-,6-/m0/s1	Organic compounds	Organic acids and derivatives	Carboxylic acids and derivatives	Amino acids, peptides, and alogues	Peptides	Oligopeptides	TRUE	TRUE	TRUE	TRUE	FALSE	FALSE	FALSE	1	0	0	FALSE	FALSE
115015	InChI=1S/C3H7NO5S2/c4-2(3(5)6)1-10-11(7,8)9/h2H,1,4H2,(H,5,6)(H,7,8,9)/t2-/m0/s1	Organic compounds	Organic acids and derivatives	Carboxylic acids and derivatives	Amino acids, peptides, and analogues	Amino acids and derivatives		FALSE	FALSE	FALSE	TRUE	FALSE	FALSE	FALSE	1	0	0	FALSE	FALSE
169148	InChI=1S/C8H18N4O2/c1-10-8(11-2)12-5-3-4-6(9)7(13)14/h6H,3-5,9H2,1-2H3,(H,13,14)(H2,10,11,12)/	Organic compounds	Organic acids and derivatives	Carboxylic acids and derivatives	Amino acids, peptides, and analogues	Amino acids and derivatives		FALSE	FALSE	FALSE	TRUE	FALSE	FALSE	FALSE	1	0	0	FALSE	FALSE
189087	InChI=1S/C7H16N2O/c1-7(10)9-6-4-2-3-5-8/h2-6,8H2,1H3,(H,9,10)	Organic compounds	Organic acids and derivatives	Carboxylic acids and derivatives	Carboxylic acid derivatives	Carboxylic acid amides	Acetamides	FALSE	FALSE	FALSE	TRUE	FALSE	FALSE	FALSE	1	0	0	FALSE	FALSE
C03413	InChI=1S/C14H30N4O2/c1-13(19)17-11-5-9-15-7-3-4-8-16-10-6-12-18-14(2)20/h15-16H,3-12H2,1-2H3,(H,17,19)(H,18,20)/p+2	Organic compounds	Organic acids and derivatives	Carboxylic acids and derivatives	Carboxylic acid derivatives	Carboxylic acid amides	Acetamides	TRUE	FALSE	TRUE	TRUE	FALSE	FALSE	FALSE	1	0	0	FALSE	FALSE
CE1059	InChI=1S/C11H23N3O2.ClH/c1-10(15)13-8-4-3-6-12-7-5-9-14-11(2)16;/h12H,3-9H2,1-2H3,(H,13,15)(H,14,16);1H	Organic compounds	Organic acids and derivatives	Carboxylic acids and derivatives	Carboxylic acid derivatives	Carboxylic acid amides	Acetamides	TRUE	FALSE	TRUE	TRUE	FALSE	FALSE	FALSE	1	0	0	FALSE	FALSE
N1aspmd	InChI=1S/C9H21N3O/c1-9(13)12-8-4-7-11-6-3-2-5-10/h11H,2-8,10H2,1H3,(H,12,13)/p+2	Organic compounds	Organic acids and derivatives	Carboxylic acids and derivatives	Carboxylic acid derivatives	Carboxylic acid amides	Acetamides	TRUE	FALSE	TRUE	TRUE	FALSE	FALSE	FALSE	1	0	0	FALSE	FALSE
aprut	InChI=1S/C6H14N2O/c1-6(9)8-5-3-2-4-7/h2-5,7H2,1H3,(H,8,9)/p+1	Organic compounds	Organic acids and derivatives	Carboxylic acids and derivatives	Carboxylic acid derivatives	Carboxylic acid amides	Acetamides	TRUE	TRUE	TRUE	TRUE	FALSE	TRUE	FALSE	2	0	0	FALSE	FALSE
n8aspmd	InChI=1S/C9H21N3O/c1-9(13)12-8-3-2-6-11-7-4-5-10/h11H,2-8,10H2,1H3,(H,12	Organic compounds	Organic acids and derivatives	Carboxylic acids and derivatives	Carboxylic acid derivatives	Carboxylic acid amides	Acetamides	TRUE	FALSE	TRUE	TRUE	FALSE	FALSE	FALSE	1	0	0	FALSE	FALSE

	,13)/p+2																			
70143	InChI=1S/C10H13NO/c1-9(12)11-8-7-10-5-3-2-4-6-10/h2-6H,7-8H2,1H3,(H,11,12)	Organic compounds	Organic acids and derivatives	Carboxylic acids and derivatives	Carboxylic acid derivatives	Carboxylic acid amides	Acetamides	N-acetyl-2-arylethylamines	FALSE	FALSE	FALSE	TRUE	FALSE	FALSE	FALSE	1	0	0	FALSE	FALSE
dhcrm_hs	InChI=1S/C19H39NO3/c1-2-3-4-5-6-7-8-9-10-11-12-13-14-15-19(23)18(16-21)20-17-22/h17-19,21,23H,2-16H2,1H3,(H,20,22)/t18-,19+/m0/s1	Organic compounds	Organic acids and derivatives	Carboxylic acids and derivatives	Carboxylic acid derivatives	Carboxylic acid amides	Secondary carboxylic acid amides		TRUE	TRUE	TRUE	TRUE	FALSE	FALSE	FALSE	1	0	0	FALSE	FALSE
76406	InChI=1S/C5H9NO3/c1-4(7)6-3-2-5(8)9/h2-3H2,1H3,(H,6,7)(H,8,9)	Organic compounds	Organic acids and derivatives	Carboxylic acids and derivatives	Carboxylic acids				FALSE	FALSE	FALSE	FALSE	TRUE	FALSE	FALSE	0	1	0	FALSE	FALSE
ac	InChI=1S/C2H4O2/c1-2(3)4/h1H3,(H,3,4)/p-1	Organic compounds	Organic acids and derivatives	Carboxylic acids and derivatives	Carboxylic acids				TRUE	TRUE	TRUE	TRUE	TRUE	TRUE	TRUE	4	3	1	FALSE	FALSE
for	InChI=1S/CH2O2/c2-1-3/h1H,(H,2,3)/p-1	Organic compounds	Organic acids and derivatives	Carboxylic acids and derivatives	Carboxylic acids				TRUE	TRUE	TRUE	TRUE	TRUE	FALSE	FALSE	1	1	1	FALSE	FALSE
ppa	InChI=1S/C3H6O2/c1-2-3(4)5/h2H2,1H3,(H,4,5)/p-1	Organic compounds	Organic acids and derivatives	Carboxylic acids and derivatives	Carboxylic acids				TRUE	TRUE	TRUE	TRUE	TRUE	TRUE	TRUE	2	2	1	FALSE	FALSE
HC00319	InChI=1S/C3H4O4/c4-2(5)1-3(6)7/h1H2,(H,4,5)(H,6,7)/p-2	Organic compounds	Organic acids and derivatives	Carboxylic acids and derivatives	Dicarboxylic acids and derivatives				TRUE	TRUE	TRUE	TRUE	FALSE	FALSE	FALSE	1	0	0	FALSE	FALSE
HC00900	InChI=1S/C4H6O4/c1-2(3(5)6)4(7)8/h2H,1H3,(H,5,6)(H,7,8)/p-2	Organic compounds	Organic acids and derivatives	Carboxylic acids and derivatives	Dicarboxylic acids and derivatives				TRUE	TRUE	TRUE	FALSE	TRUE	FALSE	FALSE	0	1	0	FALSE	FALSE
fum	InChI=1S/C4H4O4/c5-3(6)1-2-4(7)8/h1-2H,(H,5,6)(H,7,8)/p-2/b2-1+	Organic compounds	Organic acids and derivatives	Carboxylic acids and derivatives	Dicarboxylic acids and derivatives				TRUE	TRUE	TRUE	FALSE	TRUE	FALSE	FALSE	0	1	0	FALSE	FALSE
glutar	InChI=1S/C5H8O4/c6-4(7)2-1-3-5(8)9/h1-3H2,(H,6,7)(H,8,9)/p-2	Organic compounds	Organic acids and derivatives	Carboxylic acids and derivatives	Dicarboxylic acids and derivatives				TRUE	FALSE	TRUE	TRUE	TRUE	FALSE	TRUE	1	2	1	FALSE	FALSE
oxa	InChI=1S/C2H2O4/c3-1(4)2(5)6/h(H,3,4)(H,5,6)/p-2	Organic compounds	Organic acids and derivatives	Carboxylic acids and derivatives	Dicarboxylic acids and derivatives				TRUE	TRUE	TRUE	FALSE	TRUE	FALSE	FALSE	0	1	0	FALSE	FALSE
succ	InChI=1S/C4H6O4/c5-3(6)1-2-4(7)8/h1-2H2,(H,5,6)(H,7,8)/p-2	Organic compounds	Organic acids and derivatives	Carboxylic acids and derivatives	Dicarboxylic acids and derivatives				TRUE	TRUE	TRUE	TRUE	TRUE	TRUE	TRUE	3	2	1	FALSE	FALSE
135438596	InChI=1S/C18H16N2O10/c21-14-7-9(6-13(30-14)18(28)29)5-10(15(22)23)19-2-1-8-3-11(16(24)25)20-12(4-8)17(26)27/h1-3,6-7,10,12,20H,4-5H2,(H,22,23)(H,24,25)(H,26,27)(H,28,29)/b8-1-,19-2?/t10-,12-/m0/s1	Organic compounds	Organic acids and derivatives	Carboxylic acids and derivatives	Tetracarboxylic acids and derivatives				FALSE	FALSE	FALSE	FALSE	TRUE	FALSE	FALSE	0	1	0	FALSE	FALSE
HC00342	InChI=1S/C6H6O6/c7-4(8)1-3(6(11)12)2-5(9)10/h1H,2H2,(H,7,8)(H,9,10)(H,11,12)/p-3/b3-1-	Organic compounds	Organic acids and derivatives	Carboxylic acids and derivatives	Tricarboxylic acids and derivatives				TRUE	TRUE	TRUE	TRUE	TRUE	FALSE	FALSE	1	1	1	FALSE	FALSE
cit	InChI=1S/C6H8O7/c7-3(8)1-6(13,5(11)12)2-4(9)10/h13H,1-2H2,(H,7,8)(H,9,10)(H,11,12)/p-3	Organic compounds	Organic acids and derivatives	Carboxylic acids and derivatives	Tricarboxylic acids and derivatives				TRUE	TRUE	TRUE	TRUE	TRUE	TRUE	TRUE	4	2	1	FALSE	FALSE
icit	InChI=1S/C6H8O7/c7-3(8)1-2(5(10)11)4(9)6(12)13/h2,4,9H,1H2,(H,7,8)(H,10,11)(H,12,13)/p-3	Organic compounds	Organic acids and derivatives	Carboxylic acids and derivatives	Tricarboxylic acids and derivatives				TRUE	TRUE	TRUE	FALSE	TRUE	FALSE	FALSE	0	1	0	FALSE	FALSE
3hmp	InChI=1S/C4H8O3/c1-3(2-5)4(6)7/h3,5H,2H2,1H3,(H,6,7)/p-1/t3-/m0/s1	Organic compounds	Organic acids and derivatives	Hydroxy acids and derivatives	Beta hydroxy acids and derivatives				TRUE	TRUE	TRUE	FALSE	TRUE	FALSE	FALSE	0	1	0	FALSE	FALSE
3hpp	InChI=1S/C3H6O3/c4-2-1-3(5)6/h4H,1-2H2,(H,5,6)/p-1	Organic compounds	Organic acids and derivatives	Hydroxy acids and derivatives	Beta hydroxy acids and derivatives				TRUE	TRUE	TRUE	TRUE	FALSE	FALSE	FALSE	1	0	0	FALSE	FALSE
hydroxylauroylcarnitine		Organic compounds	Organic acids and derivatives	Hydroxy acids and derivatives	Beta hydroxy acids and derivatives				FALSE	FALSE	FALSE	TRUE	FALSE	FALSE	FALSE	1	0	0	FALSE	FALSE

mal_L	InChI=1S/C4H6O5/c5-2(4(8)9)1-3(6)7/h2,5H,1H2,(H,6,7)(H,8,9)	Organic compounds	Organic acids and derivatives	Hydroxy acids and derivatives	Beta hydroxy acids and derivatives		TRUE	TRUE	TRUE	TRUE	TRUE	TRUE	TRUE	2	2	1	FALSE	FALSE
74300	InChI=1S/C10H20O3/c11-9-7-5-3-1-2-4-6-8-10(12)13/h11H,1-9H2,(H,12,13)	Organic compounds	Organic acids and derivatives	Hydroxy acids and derivatives	Medium-chain hydroxy acids and derivatives		FALSE	FALSE	FALSE	TRUE	FALSE	TRUE	FALSE	2	0	0	FALSE	FALSE
M00653	InChI=1S/C5H8O5/c6-3(5(9)10)1-2-4(7)8/h3,6H,1-2H2,(H,7,8)(H,9,10)	Organic compounds	Organic acids and derivatives	Hydroxy acids and derivatives	Short-chain hydroxy acids and derivatives		TRUE	FALSE	TRUE	FALSE	TRUE	FALSE	FALSE	0	1	0	FALSE	FALSE
pyr	InChI=1S/C3H4O3/c1-2(4)3(5)6/h1H3,(H,5,6)/p-1	Organic compounds	Organic acids and derivatives	Keto acids and derivatives	Alpha-keto acids and derivatives		TRUE	TRUE	TRUE	TRUE	TRUE	TRUE	TRUE	2	2	1	FALSE	FALSE
akg	InChI=1S/C5H6O5/c6-3(5(9)10)1-2-4(7)8/h1-2H2,(H,7,8)(H,9,10)/p-2	Organic compounds	Organic acids and derivatives	Keto acids and derivatives	Gamma-keto acids and derivatives		TRUE	TRUE	TRUE	TRUE	FALSE	FALSE	FALSE	1	0	0	FALSE	FALSE
3mob	InChI=1S/C5H8O3/c1-3(2)4(6)5(7)8/h3H,1-2H3,(H,7,8)/p-1	Organic compounds	Organic acids and derivatives	Keto acids and derivatives	Short-chain keto acids and derivatives		TRUE	TRUE	TRUE	TRUE	FALSE	FALSE	FALSE	1	0	0	FALSE	FALSE
4mop	InChI=1S/C6H10O3/c1-4(2)3-5(7)6(8)9/h4H,3H2,1-2H3,(H,8,9)/p-1	Organic compounds	Organic acids and derivatives	Keto acids and derivatives	Short-chain keto acids and derivatives		TRUE	TRUE	TRUE	TRUE	FALSE	TRUE	FALSE	2	0	0	FALSE	FALSE
5g2oxpt	InChI=1S/C6H11N3O3/c7-6(8)9-3-1-2-4(10)5(11)12/h1-3H2,(H,11,12)(H4,7,8,9)	Organic compounds	Organic acids and derivatives	Keto acids and derivatives	Short-chain keto acids and derivatives		TRUE	FALSE	TRUE	TRUE	FALSE	FALSE	FALSE	1	0	0	FALSE	FALSE
acac	InChI=1S/C4H6O3/c1-3(5)2-4(6)7/h2H2,1H3,(H,6,7)/p-1	Organic compounds	Organic acids and derivatives	Keto acids and derivatives	Short-chain keto acids and derivatives		TRUE	TRUE	TRUE	TRUE	FALSE	FALSE	FALSE	1	0	0	FALSE	FALSE
h2co3	InChI=1S/CH2O3/c2-1(3)4/h(H2,2,3,4)	Organic compounds	Organic acids and derivatives	Organic carbonic acids and derivatives	Organic carbonic acids		TRUE	FALSE	TRUE	TRUE	FALSE	FALSE	FALSE	1	0	0	FALSE	FALSE
urea	InChI=1S/CH4N2O/c2-1(3)4/h(H4,2,3,4)	Organic compounds	Organic acids and derivatives	Organic carbonic acids and derivatives	Ureas		TRUE	FALSE	TRUE	TRUE	TRUE	TRUE	TRUE	2	3	1	FALSE	FALSE
ethamp	InChI=1S/C2H8NO4P/c3-1-2-7-8(4,5)6/h1-3H2,(H2,4,5,6)/p-1	Organic compounds	Organic acids and derivatives	Organic phosphoric acids and derivatives	Phosphate esters	Phosphoethanolamines	TRUE	TRUE	TRUE	TRUE	FALSE	TRUE	FALSE	2	0	0	FALSE	FALSE
21252279	InChI=1S/C3H9NO3S2/c4-3(1-2-8)9(5,6)7/h3,8H,1-2,4H2,(H,5,6,7)/t3-/m1/s1	Organic compounds	Organic acids and derivatives	Organic sulfonic acids and derivatives	Organosulfonic acids and derivatives	Organosulfonic acids	FALSE	FALSE	FALSE	FALSE	TRUE	FALSE	FALSE	0	1	0	FALSE	FALSE
taur	InChI=1S/C2H7NO3S/c3-1-2-7(4,5)6/h1-3H2,(H,4,5,6)	Organic compounds	Organic acids and derivatives	Organic sulfonic acids and derivatives	Organosulfonic acids and derivatives	Organosulfonic acids	TRUE	TRUE	TRUE	TRUE	FALSE	FALSE	FALSE	1	0	0	FALSE	FALSE
22642043	InChI=1S/C5H5NO4S/c7-11(8,9)10-5-2-1-3-6-4-5/h1-4H,(H,7,8,9)	Organic compounds	Organic acids and derivatives	Organic sulfuric acids and derivatives	Arylsulfates		FALSE	FALSE	FALSE	FALSE	TRUE	FALSE	FALSE	0	1	0	FALSE	FALSE
inds	InChI=1S/C8H7NO4S/c10-14(11,12)13-8-5-9-7-4-2-1-3-6(7)8/h1-5,9H,(H,10,11,12)/p-1	Organic compounds	Organic acids and derivatives	Organic sulfuric acids and derivatives	Arylsulfates		TRUE	FALSE	TRUE	TRUE	FALSE	TRUE	FALSE	2	0	0	FALSE	FALSE
118431379	InChI=1S/C7H8O5S/c1-5-3-2-4-6(7(5)8)12-13(9,10)11/h2-4,8H,1H3,(H,9,10,11)	Organic compounds	Organic acids and derivatives	Organic sulfuric acids and derivatives	Arylsulfates	Phenylsulfates	FALSE	FALSE	FALSE	FALSE	TRUE	FALSE	FALSE	0	1	0	FALSE	FALSE
122164839	InChI=1S/C9H10O5S/c1-3-7-4-5-8(9(6-7)13-2)14-15(10,11)12/h3-6H,1H2,2H3,(H,10,11,12)/p-1	Organic compounds	Organic acids and derivatives	Organic sulfuric acids and derivatives	Arylsulfates	Phenylsulfates	FALSE	FALSE	FALSE	FALSE	TRUE	FALSE	FALSE	0	1	0	FALSE	FALSE
180632	InChI=1S/C10H12O5S/c1-3-4-8-5-6-9(10(7-8)14-2)15-16(11,12)13/h3,5-7H,1,4H2,2H3,(H,11,12,13)	Organic compounds	Organic acids and derivatives	Organic sulfuric acids and derivatives	Arylsulfates	Phenylsulfates	FALSE	FALSE	FALSE	FALSE	TRUE	FALSE	FALSE	0	1	0	FALSE	FALSE
181670	InChI=1S/C6H7NO4S/c7-5-3-1-2-4-6(5)11-12(8,9)10/h1-4H,7H2,(H,8,9,10)	Organic compounds	Organic acids and derivatives	Organic sulfuric acids and derivatives	Arylsulfates	Phenylsulfates	FALSE	FALSE	FALSE	FALSE	TRUE	FALSE	FALSE	0	1	0	FALSE	FALSE
187488	InChI=1S/C9H10O6S/c10-9(11)5-4-7-2-1-3-8(6-7)15-16(12,13)14/h1-3,6H,4-5H2,(H,10,11)(H,12,13,14)	Organic compounds	Organic acids and derivatives	Organic sulfuric acids and derivatives	Arylsulfates	Phenylsulfates	FALSE	FALSE	FALSE	FALSE	TRUE	FALSE	FALSE	0	1	0	FALSE	FALSE

193283	InChI=1S/C8H8O7S/c9-6-3-5(4-8(10)11)1-2-7(6)15-16(12,13)14/h1-3,9H,4H2,(H,10,11)(H,12,13,14)	Organic compounds	Organic acids and derivatives	Organic sulfuric acids and derivatives	Arylsulfates	Phenylsulfates	FALSE	FALSE	FALSE	TRUE	FALSE	FALSE	FALSE	1	0	0	FALSE	FALSE
22473	InChI=1S/C7H8O5S/c1-11-6-4-2-3-5-7(6)12-13(8,9)10/h2-5H,1H3,(H,8,9,10)	Organic compounds	Organic acids and derivatives	Organic sulfuric acids and derivatives	Arylsulfates	Phenylsulfates	FALSE	FALSE	FALSE	FALSE	TRUE	FALSE	FALSE	0	1	0	FALSE	FALSE
29981063	InChI=1S/C9H10O7S/c1-15-8-4-6(5-9(10)11)2-3-7(8)16-17(12,13)14/h2-4H,5H2,1H3,(H,10,11)(H,12,13,14)	Organic compounds	Organic acids and derivatives	Organic sulfuric acids and derivatives	Arylsulfates	Phenylsulfates	FALSE	FALSE	FALSE	TRUE	FALSE	TRUE	FALSE	2	0	0	FALSE	FALSE
3083879	InChI=1S/C6H6O5S/c7-5-3-1-2-4-6(5)11-12(8,9)10/h1-4,7H,(H,8,9,10)	Organic compounds	Organic acids and derivatives	Organic sulfuric acids and derivatives	Arylsulfates	Phenylsulfates	FALSE	FALSE	FALSE	FALSE	TRUE	FALSE	TRUE	0	2	0	FALSE	FALSE
54166459	InChI=1S/C9H13NO5S/c1-14-9-6-7(4-5-10)2-3-8(9)15-16(11,12)13/h2-3,6H,4-5,10H2,1H3,(H,11,12,13)	Organic compounds	Organic acids and derivatives	Organic sulfuric acids and derivatives	Arylsulfates	Phenylsulfates	FALSE	FALSE	FALSE	TRUE	FALSE	TRUE	FALSE	2	0	0	FALSE	FALSE
91799718	InChI=1S/C10H13NO7S/c1-10(11,9(13)14)5-6-2-3-7(12)8(4-6)18-19(15,16)17/h2-4,12H,5,11H2,1H3,(H,13,14)(H,15,16,17)/t10-/m0/s1	Organic compounds	Organic acids and derivatives	Organic sulfuric acids and derivatives	Arylsulfates	Phenylsulfates	FALSE	FALSE	FALSE	FALSE	TRUE	FALSE	FALSE	0	1	0	FALSE	FALSE
dopa4sf	InChI=1S/C8H11NO5S/c9-4-3-6-1-2-8(7(10)5-6)14-15(11,12)13/h1-2,5,10H,3-4,9H2,(H,11,12,13)	Organic compounds	Organic acids and derivatives	Organic sulfuric acids and derivatives	Arylsulfates	Phenylsulfates	TRUE	TRUE	TRUE	TRUE	FALSE	FALSE	FALSE	1	0	0	FALSE	FALSE
dopasf	InChI=1S/C8H11NO5S/c9-4-3-6-1-2-7(10)8(5-6)14-15(11,12)13/h1-2,5,10H,3-4,9H2,(H,11,12,13)	Organic compounds	Organic acids and derivatives	Organic sulfuric acids and derivatives	Arylsulfates	Phenylsulfates	TRUE	TRUE	TRUE	TRUE	FALSE	FALSE	FALSE	1	0	0	FALSE	FALSE
pcs	InChI=1S/C7H8O4S/c1-6-2-4-7(5-3-6)11-12(8,9)10/h2-5H,1H3,(H,8,9,10)/p-1	Organic compounds	Organic acids and derivatives	Organic sulfuric acids and derivatives	Arylsulfates	Phenylsulfates	TRUE	FALSE	TRUE	TRUE	FALSE	TRUE	FALSE	6	0	0	FALSE	FALSE
25058105	InChI=1S/C26H45NO7/c1-7-8-9-10-11-12-14-19(6)23(28)27-16-13-15-20(27)25(31)34-22(18(4)5)26(32)33-21(17(2)3)24(29)30/h17-22H,7-16H2,1-6H3,(H,29,30)/t19-,20-,21-,22+/m0/s1	Organic compounds	Organic acids and derivatives	Peptidomimetics	Depsipeptides		FALSE	FALSE	FALSE	FALSE	TRUE	FALSE	FALSE	0	1	0	FALSE	FALSE
132993922	InChI=1S/C60H92N18O25/c1-28(2)18-36(55(98)70-30(5)50(93)73-34(14-16-46(87)88)53(96)67-23-41(81)65-22-40(80)66-24-44(84)78-49(29(3)4)58(101)74-35(59(102)103)12-9-17-64-60(62)63)75-56(99)37(19-31-10-7-6-8-11-31)76-57(100)38(21-48(91)92)72-43(83)26-68-52(95)33(13-15-45(85)86)71-42(82)25-69-54(97)39(27-79)77-51(94)32(61)20-47(89)90/h6-8,10-11,28-30,32-39,49,79H,9,12-27,61H2,1-5H3,(H,65,81)(H,66,80)(H,67,96)(H,68,95)(H,69,97)(H,70,98)(H,71,82)(H,72,83)(H,73,93)(H,74,101)(H,75,99)(H,76,100)(H,77,94)(H,78,84)(H,85,86)(H,87,88)(H,89,90)(H,91,92)(H,102,103)(H4,62,63,64)	Organic compounds	Organic acids and derivatives	Peptidomimetics	Hybrid peptides		FALSE	FALSE	FALSE	TRUE	FALSE	FALSE	FALSE	1	0	0	FALSE	FALSE

132993923	InChI=1S/C63H98N19O29P/c1-29(2)19-37(57(101)73-32(6)53(97)76-35(15-17-48(90)91)55(99)70-24-43(84)68-23-42(83)69-25-46(87)82-51(30(3)4)61(105)77-36(62(106)107)13-10-18-67-63(65)66)79-58(102)38(20-33-11-8-7-9-12-33)80-59(103)39(21-49(92)93)75-45(86)27-71-54(98)34(14-16-47(88)89)74-44(85)26-72-56(100)41(28-111-112(108,109)110)81-60(104)40(22-50(94)95)78-52(96)31(5)64/h7-9,11-12,29-32,34-41,51H,10,13-28,64H2,1-6H3,(H,68,84)(H,69,83)(H,70,99)(H,71,98)(H,72,100)(H,73,101)(H,74,85)(H,75,86)(H,76,97)(H,77,105)(H,78,96)(H,79,102)(H,80,103)(H,81,104)(H,82,87)(H,88,89)(H,90,91)(H,92,93)(H,94,95)(H,106,107)(H4,65,66,67)(H2,108,109,110)	Organic compounds	Organic acids and derivatives	Peptidomimetics	Hybrid peptides			FALSE	FALSE	FALSE	TRUE	FALSE	FALSE	FALSE	1	0	0	FALSE	FALSE
9903482	InChI=1S/C11H16N4O4/c1-7(16)13-3-2-10(17)15-9(11(18)19)4-8-5-12-6-14-8/h5-6,9H,2-4H2,1H3,(H,12,14)(H,13,16)(H,15,17)(H,18,19)/t9-/m0/s1	Organic compounds	Organic acids and derivatives	Peptidomimetics	Hybrid peptides			FALSE	FALSE	FALSE	FALSE	TRUE	FALSE	FALSE	0	1	0	FALSE	FALSE
Tripeptide: Leu/Ile-Asp-Arg; Gln-Gln-Lys; Arg-Val-Glu; Val-Val-Trp		Organic compounds	Organic acids and derivatives	Peptidomimetics	Hybrid peptides			FALSE	FALSE	FALSE	FALSE	TRUE	FALSE	FALSE	0	1	0	FALSE	FALSE
Tripeptide: Leu/Ile-Lys-Gln		Organic compounds	Organic acids and derivatives	Peptidomimetics	Hybrid peptides			FALSE	FALSE	FALSE	FALSE	TRUE	FALSE	FALSE	0	1	0	FALSE	FALSE
Tripeptide: Leu/Ile-Tyr-Lys		Organic compounds	Organic acids and derivatives	Peptidomimetics	Hybrid peptides			FALSE	FALSE	FALSE	FALSE	TRUE	FALSE	FALSE	0	1	0	FALSE	FALSE
Tripeptide: Thr-Pro-Ala		Organic compounds	Organic acids and derivatives	Peptidomimetics	Hybrid peptides			FALSE	FALSE	FALSE	FALSE	TRUE	FALSE	FALSE	0	1	0	FALSE	FALSE
Bioactive amine		Organic compounds	Organic nitrogen compounds	Organonitrogen compounds	Amines			FALSE	FALSE	FALSE	TRUE	FALSE	FALSE	FALSE	1	0	0	FALSE	FALSE
9835868	InChI=1S/C18H35NO2/c1-2-3-4-5-6-7-8-9-10-11-12-13-14-15-18(21)19-16-17-20/h7-8,20H,2-6,9-17H2,1H3,(H,19,21)/b8-7-	Organic compounds	Organic nitrogen compounds	Organonitrogen compounds	Amines	Alkanolamines		FALSE	FALSE	FALSE	TRUE	FALSE	FALSE	FALSE	1	0	0	FALSE	FALSE
appl	InChI=1S/C3H9NO/c1-3(5)2-4/h3,5H,2,4H2,1H3	Organic compounds	Organic nitrogen compounds	Organonitrogen compounds	Amines	Alkanolamines	1,2-aminoalcohols	FALSE	FALSE	TRUE	TRUE	FALSE	FALSE	FALSE	1	0	0	FALSE	FALSE
etha	InChI=1S/C2H7NO/c3-1-2-4/h4H,1-3H2/p+1	Organic compounds	Organic nitrogen compounds	Organonitrogen compounds	Amines	Alkanolamines	1,2-aminoalcohols	TRUE	TRUE	TRUE	TRUE	TRUE	FALSE	TRUE	1	2	1	FALSE	FALSE
sphgn	InChI=1S/C18H39NO2/c1-2-3-4-5-6-7-8-9-10-11-12-13-14-15-18(21)17(19)16-20/h17-18,20-21H,2-16,19H2,1H3/p+1/t17-,18+/m0/s1	Organic compounds	Organic nitrogen compounds	Organonitrogen compounds	Amines	Alkanolamines	1,2-aminoalcohols	TRUE	TRUE	TRUE	TRUE	FALSE	FALSE	FALSE	1	0	0	FALSE	FALSE

sphings	InChI=1S/C18H37NO2/c1-2-3-4-5-6-7-8-9-10-11-12-13-14-15-18(21)17(19)16-20/h14-15,17-18,20-21H,2-13,16,19H2,1H3/p+1	Organic compounds	Organic nitrogen compounds	Organonitrogen compounds	Amines	Alkanolamines	1,2-aminoalcohols		TRUE	TRUE	TRUE	TRUE	FALSE	FALSE	FALSE	1	0	0	FALSE	FALSE
5283457	InChI=1S/C26H45NO2/c1-4-5-6-18-21-26(2,3)22-19-16-14-12-10-8-7-9-11-13-15-17-20-25(29)27-23-24-28/h7-8,11-14,19,22,28H,4-6,9-10,15-18,20-21,23-24H2,1-3H3,(H,27,29)/b8-7-,13-11-,14-12-,22-19-	Organic compounds	Organic nitrogen compounds	Organonitrogen compounds	Amines	Alkanolamines	1,2-aminoalcohols	N-acylethanolamines	FALSE	FALSE	FALSE	FALSE	TRUE	FALSE	FALSE	0	1	0	FALSE	FALSE
lineth	InChI=1S/C20H37NO2/c1-2-3-4-5-6-7-8-9-10-11-12-13-14-15-16-17-20(23)21-18-19-22/h6-7,9-10,22H,2-5,8,11-19H2,1H3,(H,21,23)/b7-6-,10-9-	Organic compounds	Organic nitrogen compounds	Organonitrogen compounds	Amines	Alkanolamines	1,2-aminoalcohols	N-acylethanolamines	TRUE	FALSE	TRUE	TRUE	FALSE	FALSE	FALSE	1	0	0	FALSE	FALSE
oleth	InChI=1S/C20H39NO2/c1-2-3-4-5-6-7-8-9-10-11-12-13-14-15-16-17-20(23)21-18-19-22/h9-10,22H,2-8,11-19H2,1H3,(H,21,23)/b10-9-	Organic compounds	Organic nitrogen compounds	Organonitrogen compounds	Amines	Alkanolamines	1,2-aminoalcohols	N-acylethanolamines	TRUE	FALSE	TRUE	TRUE	FALSE	FALSE	FALSE	1	0	0	FALSE	FALSE
14757418	InChI=1S/C18H37NO3/c1-2-3-4-5-6-7-8-9-10-11-12-13-14-17(21)18(22)16(19)15-20/h10-11,16-18,20-22H,2-9,12-15,19H2,1H3/b11-10+/t16-,17+,18-/m0/s1	Organic compounds	Organic nitrogen compounds	Organonitrogen compounds	Amines	Alkanolamines	1,3-aminoalcohols		FALSE	FALSE	FALSE	TRUE	FALSE	FALSE	FALSE	1	0	0	FALSE	FALSE
hista	InChI=1S/C5H9N3/c6-2-1-5-3-7-4-8-5/h3-4H,1-2,6H2,(H,7,8)/p+1	Organic compounds	Organic nitrogen compounds	Organonitrogen compounds	Amines	Aralkylamines			TRUE	TRUE	TRUE	TRUE	FALSE	FALSE	FALSE	1	0	0	FALSE	FALSE
ppbng	InChI=1S/C10H14N2O4/c11-4-8-7(3-10(15)16)6(5-12-8)1-2-9(13)14/h5,12H,1-4,11H2,(H,13,14)(H,15,16)/p-1	Organic compounds	Organic nitrogen compounds	Organonitrogen compounds	Amines	Aralkylamines			TRUE	TRUE	TRUE	TRUE	FALSE	FALSE	FALSE	1	0	0	FALSE	FALSE
15dap	InChI=1S/C5H14N2/c6-4-2-1-3-5-7/h1-7H2	Organic compounds	Organic nitrogen compounds	Organonitrogen compounds	Amines	Primary amines	Monoalkylamines		FALSE	FALSE	TRUE	TRUE	FALSE	FALSE	FALSE	1	0	0	FALSE	FALSE
mma	InChI=1S/CH5N/c1-2/h2H2,1H3/p+1	Organic compounds	Organic nitrogen compounds	Organonitrogen compounds	Amines	Primary amines	Monoalkylamines		TRUE	FALSE	TRUE	FALSE	TRUE	FALSE	FALSE	0	1	0	FALSE	FALSE
ptrc	InChI=1S/C4H12N2/c5-3-1-2-4-6/h1-6H2/p+2	Organic compounds	Organic nitrogen compounds	Organonitrogen compounds	Amines	Primary amines	Monoalkylamines		TRUE	TRUE	TRUE	TRUE	FALSE	TRUE	FALSE	2	0	0	FALSE	FALSE
dma	InChI=1S/C2H7N/c1-3-2/h3H,1-2H3	Organic compounds	Organic nitrogen compounds	Organonitrogen compounds	Amines	Secondary amines	Dialkylamines		FALSE	FALSE	TRUE	TRUE	FALSE	FALSE	FALSE	1	0	0	FALSE	FALSE
spmd	InChI=1S/C7H19N3/c8-4-1-2-6-10-7-3-5-9/h10H,1-9H2/p+3	Organic compounds	Organic nitrogen compounds	Organonitrogen compounds	Amines	Secondary amines	Dialkylamines		TRUE	TRUE	TRUE	TRUE	FALSE	TRUE	FALSE	3	0	0	FALSE	FALSE
sprm	InChI=1S/C10H26N4/c11-5-3-9-13-7-1-2-8-14-10-4-6-12/h13-14H,1-12H2/p+4	Organic compounds	Organic nitrogen compounds	Organonitrogen compounds	Amines	Secondary amines	Dialkylamines		TRUE	TRUE	TRUE	FALSE	TRUE	FALSE	FALSE	0	1	0	FALSE	FALSE
M03053	InChI=1S/C3H9N/c1-4(2)3/h1-3H3	Organic compounds	Organic nitrogen compounds	Organonitrogen compounds	Amines	Tertiary amines	Trialkylamines		TRUE	FALSE	TRUE	FALSE	TRUE	FALSE	TRUE	0	2	0	FALSE	FALSE
M03054	InChI=1S/C3H9NO/c1-4(2,3)5/h1-3H3	Organic compounds	Organic nitrogen compounds	Organonitrogen compounds	Aminoxides	Trialkyl amine oxides			TRUE	FALSE	TRUE	TRUE	TRUE	TRUE	FALSE	5	1	1	FALSE	FALSE
7965	InChI=1S/C6H13N/c7-6-4-2-1-3-5-6/h6H,1-5,7H2	Organic compounds	Organic nitrogen compounds	Organonitrogen compounds	Cyclohexylamines				FALSE	FALSE	FALSE	FALSE	TRUE	FALSE	FALSE	0	1	0	FALSE	FALSE
88299	InChI=1S/C7H10N4O3/c1-3(12)9-4-5(8)10-7(14)11(2)6(4)13/h8H2,1-2H3,(H,9,12)(H,10,14)	Organic compounds	Organic nitrogen compounds	Organonitrogen compounds	N-arylamides	N-acetylarylamines			FALSE	FALSE	FALSE	TRUE	TRUE	FALSE	TRUE	1	5	1	FALSE	FALSE
crn	InChI=1S/C7H15NO3/c1-8(2,3)5-6(9)4-7(10)11/h6,9H,4-5H2,1-3H3	Organic compounds	Organic nitrogen compounds	Organonitrogen compounds	Quaternary ammonium salts	Carnitines			TRUE	TRUE	TRUE	TRUE	TRUE	FALSE	TRUE	1	2	1	TRUE	FALSE

chol	InChI=1S/C5H14NO/c1-6(2,3)4-5-7/h7H,4-5H2,1-3H3/q+1	Organic compounds	Organic nitrogen compounds	Organonitrogen compounds	Quaterry ammonium salts	Cholines				TRUE	TRUE	TRUE	TRUE	TRUE	FALSE	TRUE	1	2	1	TRUE	FALSE
18025	InChI=1S/C12H26NO2/c1-5-6-7-8-9-12(14)15-11-10-13(2,3)4/h5-11H2,1-4H3/q+1	Organic compounds	Organic nitrogen compounds	Organonitrogen compounds	Quaterry ammonium salts	Cholines	Acyl cholines			FALSE	FALSE	FALSE	FALSE	TRUE	FALSE	FALSE	0	1	0	FALSE	FALSE
cholp	InChI=1S/C5H14NO4P/c1-6(2,3)4-5-10-11(7,8)9/h4-5H2,1-3H3,(H-,7,8,9)/p-1	Organic compounds	Organic nitrogen compounds	Organonitrogen compounds	Quaterry ammonium salts	Cholines	Phosphocholines			TRUE	TRUE	TRUE	FALSE	TRUE	FALSE	FALSE	0	1	0	FALSE	FALSE
betald	InChI=1S/C5H12NO/c1-6(2,3)4-5-7/h5H,4H2,1-3H3/q+1	Organic compounds	Organic nitrogen compounds	Organonitrogen compounds	Quaterry ammonium salts	Tetraalkylammonium salts				TRUE	FALSE	TRUE	TRUE	FALSE	FALSE	FALSE	1	0	0	FALSE	FALSE
86355	InChI=1S/C13H16N10O5S/c1-22-11(7(6-14-22)10-18-21-23(2)19-10)29(25,26)20-13(24)17-12-15-8(27-3)5-9(16-12)28-4/h5-6H,1-4H3,(H2,15,16,17,20,24)	Organic compounds	Organic nitrogen compounds	Organonitrogen compounds	Sulfonylureas	Pyrimidinyl-2-sulfonylureas				FALSE	FALSE	FALSE	FALSE	TRUE	FALSE	FALSE	0	1	0	FALSE	FALSE
636406	InChI=1S/C14H22N4O9P2S/c1-8-12(3-4-26-29(24,25)27-28(21,22)23)30-14(11(20)7-19)18(8)6-10-5-16-9(2)17-13(10)15/h5,19-20H,3-4,6-7H2,1-2H3,(H,24,25)(H2,15,16,17)(H2,21,22,23)	Organic compounds	Organic oxygen compounds	Organic oxoanionic compounds	Organic pyrophosphates					FALSE	FALSE	FALSE	FALSE	TRUE	FALSE	FALSE	0	1	0	FALSE	FALSE
439440	InChI=1S/C6H13NO5/c7-1-2(8)4(10)6(12)5(11)3(1)9/h1-6,8-12H,7H2/t1?,2-,3+,4+,5-,6?	Organic compounds	Organic oxygen compounds	Organooxygen compounds	Alcohols and polyols	Cyclic alcohols and derivatives	Cyclitols and derivatives	Aminocyclitols and derivatives	Aminocyclitols	FALSE	FALSE	FALSE	TRUE	FALSE	FALSE	FALSE	1	0	0	FALSE	FALSE
quint	InChI=1S/C7H12O6/c8-3-1-7(13,6(11)12)2-4(9)5(3)10/h3-5,8-10,13H,1-2H2,(H,11,12)/t3-,4-,5?,7?/m1/s1	Organic compounds	Organic oxygen compounds	Organooxygen compounds	Alcohols and polyols	Cyclic alcohols and derivatives	Cyclitols and derivatives	Quinic acids and derivatives		FALSE	FALSE	TRUE	TRUE	TRUE	TRUE	FALSE	2	1	1	FALSE	FALSE
12ppd_R	InChI=1S/C3H8O2/c1-3(5)2-4/h3-5H,2H2,1H3	Organic compounds	Organic oxygen compounds	Organooxygen compounds	Alcohols and polyols	Polyols	1,2-diols			TRUE	FALSE	TRUE	TRUE	FALSE	FALSE	FALSE	1	0	0	FALSE	FALSE
etoh	InChI=1S/C2H6O/c1-2-3/h3H,2H2,1H3	Organic compounds	Organic oxygen compounds	Organooxygen compounds	Alcohols and polyols	Primary alcohols				TRUE	FALSE	TRUE	TRUE	FALSE	FALSE	FALSE	1	0	0	FALSE	FALSE
94220	InChI=1S/C3H7NO2/c1-2(5)3(4)6/h2,5H,1H3,(H2,4,6)	Organic compounds	Organic oxygen compounds	Organooxygen compounds	Alcohols and polyols	Secondary alcohols				FALSE	FALSE	FALSE	FALSE	TRUE	FALSE	FALSE	0	1	0	FALSE	FALSE
lac_L	InChI=1S/C3H6O3/c1-2(4)3(5)6/h2,4H,1H3,(H,5,6)/p-1/t2-/m0/s1	Organic compounds	Organic oxygen compounds	Organooxygen compounds	Alcohols and polyols	Secondary alcohols				TRUE	TRUE	TRUE	TRUE	TRUE	TRUE	FALSE	3	1	1	TRUE	FALSE
pnto_R	InChI=1S/C9H17NO5/c1-9(2,5-11)7(14)8(15)10-4-3-6(12)13/h7,11,14H,3-5H2,1-2H3,(H,10,15)(H,12,13)/p-1	Organic compounds	Organic oxygen compounds	Organooxygen compounds	Alcohols and polyols	Secondary alcohols				TRUE	FALSE	TRUE	FALSE	TRUE	FALSE	TRUE	0	5	0	TRUE	FALSE
131769924	InChI=1S/C19H35NO/c21-18-11-9-16(10-12-18)19(15-6-2-1-3-7-15)14-17-8-4-5-13-20-17/h15-21H,1-14H2/t16?,17?,18?,19-/m0/s1	Organic compounds	Organic oxygen compounds	Organooxygen compounds	Alcohols and polyols	Secondary alcohols	Cyclohexanols			FALSE	FALSE	FALSE	TRUE	FALSE	FALSE	FALSE	1	0	0	FALSE	FALSE
inost	InChI=1S/C6H12O6/c7-1-2(8)4(10)6(12)5(11)3(1)9/h1-12H/t1-,2-,3-,4+,5-,6-	Organic compounds	Organic oxygen compounds	Organooxygen compounds	Alcohols and polyols	Secondary alcohols	Cyclohexanols			TRUE	TRUE	TRUE	TRUE	TRUE	TRUE	TRUE	2	2	1	FALSE	FALSE
M03165	InChI=1S/C7H14O7/c8-1-3-4(10)5(11)6(12)7(13,2-9)14-3/h3-6,8-13H,1-2H2/t3-,4-,5-,6+,7?/m1/s1	Organic compounds	Organic oxygen compounds	Organooxygen compounds	Carbohydrates and carbohydrate conjugates	Glycosyl compounds	C-glycosyl compounds			TRUE	FALSE	TRUE	TRUE	FALSE	FALSE	FALSE	1	0	0	FALSE	FALSE
131750952	InChI=1S/C18H22O10/c19-7-12-17(28-14(22)5-9-1-3-10(20)4-2-9)15(23)16(24)18(27-12)26-11-6-13(21)25-8-11/h1-4,11-12,15-20,23-24H,5-8H2	Organic compounds	Organic oxygen compounds	Organooxygen compounds	Carbohydrates and carbohydrate conjugates	Glycosyl compounds	O-glycosyl compounds			FALSE	FALSE	FALSE	TRUE	FALSE	FALSE	FALSE	1	0	0	FALSE	FALSE

sucr	InChI=1S/C12H22O11/c13-1-4-6(16)8(18)9(19)11(21-4)23-12(3-15)10(20)7(17)5(2-14)22-12/h4-11,13-20H,1-3H2/t4-,5-,6-,7-,8+,9-,10+,11-,12+/m1/s1	Organic compounds	Organic oxygen compounds	Organooxygen compounds	Carbohydrates and carbohydrate conjugates	Glycosyl compounds	O-glycosyl compounds			TRUE	FALSE	TRUE	TRUE	FALSE	FALSE	FALSE	1	0	0	FALSE	FALSE
154035	InChI=1S/C13H16O7/c1-6-2-4-7(5-3-6)19-13-10(16)8(14)9(15)11(20-13)12(17)18/h2-5,8-11,13-16H,1H3,(H,17,18)/t8-,9-,10+,11-,13+/m0/s1	Organic compounds	Organic oxygen compounds	Organooxygen compounds	Carbohydrates and carbohydrate conjugates	Glycosyl compounds	Phenolic glycosides			FALSE	FALSE	FALSE	TRUE	FALSE	FALSE	FALSE	1	0	0	FALSE	FALSE
64960	InChI=1S/C6H12O5/c7-1-4-6(10)5(9)3(8)2-11-4/h3-10H,1-2H2/t3-,4+,5+,6+/m0/s1	Organic compounds	Organic oxygen compounds	Organooxygen compounds	Carbohydrates and carbohydrate conjugates	Monosaccharides				FALSE	FALSE	FALSE	TRUE	TRUE	FALSE	FALSE	1	1	1	FALSE	FALSE
87141275	InChI=1S/C18H37NO2/c1-2-3-4-5-6-7-8-9-10-11-12-13-14-15-17(20)18(21)16-19/h18,21H,2-16,19H2,1H3	Organic compounds	Organic oxygen compounds	Organooxygen compounds	Carbohydrates and carbohydrate conjugates	Monosaccharides				FALSE	FALSE	FALSE	TRUE	FALSE	FALSE	FALSE	1	0	0	FALSE	FALSE
fru	InChI=1S/C6H12O6/c7-1-3-4(9)5(10)6(11,2-8)12-3/h3-5,7-11H,1-2H2/t3-,4-,5+,6-/m1/s1	Organic compounds	Organic oxygen compounds	Organooxygen compounds	Carbohydrates and carbohydrate conjugates	Monosaccharides				TRUE	TRUE	TRUE	TRUE	TRUE	FALSE	FALSE	1	1	1	FALSE	FALSE
srb_L	InChI=1S/C6H14O6/c7-1-3(9)5(11)6(12)4(10)2-8/h3-12H,1-2H2/t3-,4+,5-,6-/m0/s1	Organic compounds	Organic oxygen compounds	Organooxygen compounds	Carbohydrates and carbohydrate conjugates	Monosaccharides				TRUE	FALSE	TRUE	TRUE	FALSE	FALSE	FALSE	1	0	0	FALSE	FALSE
fuc_L	InChI=1S/C6H12O5/c1-2-3(7)4(8)5(9)6(10)11-2/h2-10H,1H3/t2-,3+,4+,5-,6?/m0/s1	Organic compounds	Organic oxygen compounds	Organooxygen compounds	Carbohydrates and carbohydrate conjugates	Monosaccharides	Hexoses			TRUE	FALSE	TRUE	TRUE	TRUE	TRUE	FALSE	2	1	1	FALSE	FALSE
glc_D	InChI=1S/C6H12O6/c7-1-2-3(8)4(9)5(10)6(11)12-2/h2-11H,1H2/t2-,3-,4+,5-,6?/m1/s1	Organic compounds	Organic oxygen compounds	Organooxygen compounds	Carbohydrates and carbohydrate conjugates	Monosaccharides	Hexoses			TRUE	TRUE	TRUE	TRUE	TRUE	TRUE	TRUE	3	2	1	TRUE	FALSE
man	InChI=1S/C6H12O6/c7-1-2-3(8)4(9)5(10)6(11)12-2/h2-11H,1H2/t2-,3-,4+,5+,6?/m1/s1	Organic compounds	Organic oxygen compounds	Organooxygen compounds	Carbohydrates and carbohydrate conjugates	Monosaccharides	Hexoses			TRUE	FALSE	TRUE	TRUE	TRUE	FALSE	FALSE	1	1	1	FALSE	FALSE
131752365	InChI=1S/C11H21NO10S2/c1-11(2,17)3-6(12-22-24(18,19)20)23-10-9(16)8(15)7(14)5(4-13)21-10/h5,7-10,13-17H,3-4H2,1-2H3,(H,18,19,20)/b12-6+	Organic compounds	Organic oxygen compounds	Organooxygen compounds	Carbohydrates and carbohydrate conjugates	Monosaccharides	Hexoses	Glucosinolates	Alkylglucosinolates	FALSE	FALSE	FALSE	TRUE	FALSE	FALSE	FALSE	1	0	0	FALSE	FALSE
151149	InChI=1S/C6H12O9S/c7-1-3(8)5(10)6(11)4(9)2-15-16(12,13)14/h1,3-6,8-11H,2H2,(H,12,13,14)/t3-,4+,5+,6+/m0/s1	Organic compounds	Organic oxygen compounds	Organooxygen compounds	Carbohydrates and carbohydrate conjugates	Monosaccharides	Monosaccharide sulfates			FALSE	FALSE	FALSE	TRUE	FALSE	FALSE	FALSE	1	0	0	FALSE	FALSE
arab_L	InChI=1S/C5H10O5/c6-2-1-10-5(9)4(8)3(2)7/h2-9H,1H2/t2-,3-,4+,5-/m0/s1	Organic compounds	Organic oxygen compounds	Organooxygen compounds	Carbohydrates and carbohydrate conjugates	Monosaccharides	Pentoses			TRUE	FALSE	TRUE	FALSE	TRUE	FALSE	FALSE	0	1	0	FALSE	FALSE
malttr	InChI=1S/C18H32O16/c19-1-4-7(22)8(23)12(27)17(31-4)34-15-6(3-21)32-18(13(28)10(15)25)33-14-5(2-20)30-16(29)11(26)9(14)24/h4-29H,1-3H2/t4-,5-,6-,7-,8+,9-,10-,11-,12-,13-,14-,15-,16+,17-,18-/m1/s1	Organic compounds	Organic oxygen compounds	Organooxygen compounds	Carbohydrates and carbohydrate conjugates	Oligosaccharides				TRUE	FALSE	TRUE	TRUE	FALSE	FALSE	FALSE	1	0	0	FALSE	TRUE
44	InChI=1S/C3H4O5/c4-1(2(5)6)3(7)8/h1,4H,(H,5,6)(H,7,8)/p-2	Organic compounds	Organic oxygen compounds	Organooxygen compounds	Carbohydrates and carbohydrate conjugates	Sugar acids and derivatives				FALSE	FALSE	FALSE	TRUE	FALSE	FALSE	FALSE	1	0	0	FALSE	FALSE
5460677	InChI=1S/C5H10O6/c6-1-2(7)3(8)4(9)5(10)11/h2-4,6-9H,1H2,(H,10,11)/t2-,3-,4-	Organic compounds	Organic oxygen compounds	Organooxygen compounds	Carbohydrates and carbohydrate conjugates	Sugar acids and derivatives				FALSE	FALSE	FALSE	TRUE	FALSE	FALSE	FALSE	1	0	0	FALSE	FALSE

	/m1/s1																				
glcn	InChI=1S/C6H12O7/c7-1-2(8)3(9)4(10)5(11)6(12)13/h2-5,7-11H,1H2,(H,12,13)/p-1/t2-,3-,4+,5-/m1/s1	Organic compounds	Organic oxygen compounds	Organooxygen compounds	Carbohydrates and carbohydrate conjugates	Sugar acids and derivatives				TRUE	TRUE	TRUE	FALSE	TRUE	FALSE	FALSE	0	1	0	FALSE	FALSE
glyc_R	InChI=1S/C3H6O4/c4-1-2(5)3(6)7/h2,4-5H,1H2,(H,6,7)/p-1/t2-/m0/s1	Organic compounds	Organic oxygen compounds	Organooxygen compounds	Carbohydrates and carbohydrate conjugates	Sugar acids and derivatives				TRUE	TRUE	TRUE	TRUE	TRUE	FALSE	TRUE	1	2	1	FALSE	FALSE
thrnt	InChI=1S/C4H8O5/c5-1-2(6)3(7)4(8)9/h2-3,5-7H,1H2,(H,8,9)/p-1/t2-,3+/m0/s1	Organic compounds	Organic oxygen compounds	Organooxygen compounds	Carbohydrates and carbohydrate conjugates	Sugar acids and derivatives				TRUE	FALSE	TRUE	TRUE	TRUE	TRUE	FALSE	2	1	1	FALSE	FALSE
Furosemide glucuronide		Organic compounds	Organic oxygen compounds	Organooxygen compounds	Carbohydrates and carbohydrate conjugates	Sugar acids and derivatives	Glucuronic acid derivatives			FALSE	FALSE	FALSE	FALSE	TRUE	FALSE	FALSE	0	1	0	FALSE	FALSE
152226	InChI=1S/C8H14O7/c1-2-14-8-5(11)3(9)4(10)6(15-8)7(12)13/h3-6,8-11H,2H2,1H3,(H,12,13)/t3-,4-,5+,6-,8?/m0/s1	Organic compounds	Organic oxygen compounds	Organooxygen compounds	Carbohydrates and carbohydrate conjugates	Sugar acids and derivatives	Glucuronic acid derivatives	Glucuronides	O-glucuronides	FALSE	FALSE	FALSE	FALSE	TRUE	FALSE	FALSE	0	1	0	FALSE	FALSE
168876	InChI=1S/C13H14O9/c14-6-4-2-1-3-5(6)12(20)22-13-9(17)7(15)8(16)10(21-13)11(18)19/h1-4,7-10,13-17H,(H,18,19)/t7-,8-,9+,10-,13-/m0/s1	Organic compounds	Organic oxygen compounds	Organooxygen compounds	Carbohydrates and carbohydrate conjugates	Sugar acids and derivatives	Glucuronic acid derivatives	Glucuronides	O-glucuronides	FALSE	FALSE	FALSE	FALSE	TRUE	FALSE	FALSE	0	1	0	FALSE	FALSE
169019	InChI=1S/C4H10O4/c5-1-3(7)4(8)2-6/h3-8H,1-2H2/t3-,4-/m1/s1	Organic compounds	Organic oxygen compounds	Organooxygen compounds	Carbohydrates and carbohydrate conjugates	Sugar alcohols				FALSE	FALSE	FALSE	TRUE	TRUE	FALSE	TRUE	1	2	1	FALSE	FALSE
C01507	InChI=1S/C6H14O6/c7-1-3(9)5(11)6(12)4(10)2-8/h3-12H,1-2H2/t3-,4-,5+,6+/m0/s1	Organic compounds	Organic oxygen compounds	Organooxygen compounds	Carbohydrates and carbohydrate conjugates	Sugar alcohols				TRUE	FALSE	TRUE	TRUE	FALSE	TRUE	FALSE	2	0	0	FALSE	FALSE
Sugar alcohol		Organic compounds	Organic oxygen compounds	Organooxygen compounds	Carbohydrates and carbohydrate conjugates	Sugar alcohols				FALSE	FALSE	FALSE	TRUE	FALSE	FALSE	FALSE	1	0	0	FALSE	FALSE
galt	InChI=1S/C6H14O6/c7-1-3(9)5(11)6(12)4(10)2-8/h3-12H,1-2H2/t3-,4+,5+,6-	Organic compounds	Organic oxygen compounds	Organooxygen compounds	Carbohydrates and carbohydrate conjugates	Sugar alcohols				TRUE	TRUE	TRUE	TRUE	TRUE	FALSE	FALSE	1	1	1	FALSE	FALSE
glyc	InChI=1S/C3H8O3/c4-1-3(6)2-5/h3-6H,1-2H2	Organic compounds	Organic oxygen compounds	Organooxygen compounds	Carbohydrates and carbohydrate conjugates	Sugar alcohols				TRUE	TRUE	TRUE	TRUE	TRUE	FALSE	TRUE	1	2	1	FALSE	FALSE
sbt_D	InChI=1S/C6H14O6/c7-1-3(9)5(11)6(12)4(10)2-8/h3-12H,1-2H2/t3-,4+,5-,6-/m0/s1	Organic compounds	Organic oxygen compounds	Organooxygen compounds	Carbohydrates and carbohydrate conjugates	Sugar alcohols				TRUE	TRUE	TRUE	TRUE	FALSE	TRUE	FALSE	2	0	0	FALSE	FALSE
xylt	InChI=1S/C5H12O5/c6-1-3(8)5(10)4(9)2-7/h3-10H,1-2H2/t3-,4+,5+	Organic compounds	Organic oxygen compounds	Organooxygen compounds	Carbohydrates and carbohydrate conjugates	Sugar alcohols				TRUE	TRUE	TRUE	FALSE	TRUE	FALSE	FALSE	0	1	0	FALSE	FALSE
actn_R	InChI=1S/C4H8O2/c1-3(5)4(2)6/h3,5H,1-2H3	Organic compounds	Organic oxygen compounds	Organooxygen compounds	Carbonyl compounds	Acyloins				FALSE	FALSE	TRUE	TRUE	FALSE	FALSE	FALSE	1	0	0	FALSE	FALSE
11552	InChI=1S/C5H10O/c1-5(2)3-4-6/h4-5H,3H2,1-2H3	Organic compounds	Organic oxygen compounds	Organooxygen compounds	Carbonyl compounds	Aldehydes	Alpha-hydrogen aldehydes			FALSE	FALSE	FALSE	TRUE	FALSE	FALSE	FALSE	1	0	0	FALSE	FALSE
18635	InChI=1S/C4H8OS/c1-6-4-2-3-5/h3H,2,4H2,1H3	Organic compounds	Organic oxygen compounds	Organooxygen compounds	Carbonyl compounds	Aldehydes	Alpha-hydrogen aldehydes			FALSE	FALSE	FALSE	TRUE	FALSE	FALSE	FALSE	1	0	0	FALSE	FALSE
4abutn	InChI=1S/C4H9NO/c5-3-1-2-4-6/h4H,1-3,5H2/p+1	Organic compounds	Organic oxygen compounds	Organooxygen compounds	Carbonyl compounds	Aldehydes	Alpha-hydrogen aldehydes			TRUE	TRUE	TRUE	TRUE	FALSE	FALSE	FALSE	1	0	0	FALSE	FALSE
4hbald	InChI=1S/C7H6O2/c8-5-6-1-3-7(9)4-2-6/h1-5,9H	Organic compounds	Organic oxygen compounds	Organooxygen compounds	Carbonyl compounds	Aldehydes	Aryl-aldehydes	Benzaldehydes	Hydroxybenzaldehydes	FALSE	FALSE	TRUE	TRUE	FALSE	FALSE	FALSE	1	0	0	FALSE	FALSE
8130	InChI=1S/C7H14O/c1-2-3-4-5-6-7-8/h7H,2-6H2,1H3	Organic compounds	Organic oxygen compounds	Organooxygen compounds	Carbonyl compounds	Aldehydes	Medium-chain aldehydes			FALSE	FALSE	FALSE	FALSE	TRUE	FALSE	FALSE	0	1	0	FALSE	FALSE

hxal	InChI=1S/C6H12O/c1-2-3-4-5-6-7/h6H,2-5H2,1H3	Organic compounds	Organic oxygen compounds	Organooxygen compounds	Carbonyl compounds	Aldehydes	Medium-chain aldehydes				FALSE	FALSE	TRUE	TRUE	FALSE	FALSE	FALSE	1	0	0	FALSE	FALSE
6561	InChI=1S/C4H8O/c1-4(2)3-5/h3-4H,1-2H3	Organic compounds	Organic oxygen compounds	Organooxygen compounds	Carbonyl compounds	Aldehydes	Short-chain aldehydes				FALSE	FALSE	FALSE	TRUE	FALSE	FALSE	FALSE	1	0	0	FALSE	FALSE
9839212	InChI=1S/C18H35NO2/c1-2-3-4-5-6-7-8-9-10-11-12-13-14-15-18(21)17(19)16-20/h14-15,17,20H,2-13,16,19H2,1H3/b15-14+/t17-/m0/s1	Organic compounds	Organic oxygen compounds	Organooxygen compounds	Carbonyl compounds	Alpha,beta-unsaturated carbonyl compounds	Alpha,beta-unsaturated ketones	b'-hydroxy-alpha,beta-unsaturated ketones			FALSE	FALSE	FALSE	TRUE	FALSE	TRUE	FALSE	2	0	0	FALSE	FALSE
13187	InChI=1S/C9H18O/c1-3-4-5-6-7-8-9(2)10/h3-8H2,1-2H3	Organic compounds	Organic oxygen compounds	Organooxygen compounds	Carbonyl compounds	Ketones					FALSE	FALSE	FALSE	FALSE	TRUE	FALSE	FALSE	0	1	0	FALSE	FALSE
28965	InChI=1S/C8H16O/c1-4-7(2)5-6-8(3)9/h7H,4-6H2,1-3H3	Organic compounds	Organic oxygen compounds	Organooxygen compounds	Carbonyl compounds	Ketones					FALSE	FALSE	FALSE	FALSE	TRUE	FALSE	FALSE	0	1	0	FALSE	FALSE
6569	InChI=1S/C4H8O/c1-3-4(2)5/h3H2,1-2H3	Organic compounds	Organic oxygen compounds	Organooxygen compounds	Carbonyl compounds	Ketones					FALSE	FALSE	FALSE	TRUE	FALSE	FALSE	FALSE	1	0	0	FALSE	FALSE
8051		Organic compounds	Organic oxygen compounds	Organooxygen compounds	Carbonyl compounds	Ketones					FALSE	FALSE	FALSE	FALSE	TRUE	FALSE	FALSE	0	1	0	FALSE	FALSE
acetone	InChI=1S/C3H6O/c1-3(2)4/h1-2H3	Organic compounds	Organic oxygen compounds	Organooxygen compounds	Carbonyl compounds	Ketones					TRUE	TRUE	TRUE	TRUE	FALSE	TRUE	FALSE	4	0	0	FALSE	FALSE
aact	InChI=1S/C3H7NO/c1-3(5)2-4/h2,4H2,1H3/p+1	Organic compounds	Organic oxygen compounds	Organooxygen compounds	Carbonyl compounds	Ketones	Alpha-amino ketones				TRUE	FALSE	TRUE	TRUE	FALSE	FALSE	FALSE	1	0	0	FALSE	FALSE
11811	InChI=1S/C10H12N2O4/c11-6(10(15)16)4-8(14)5-2-1-3-7(13)9(5)12/h1-3,6,13H,4,11-12H2,(H,15,16)/t6-/m0/s1	Organic compounds	Organic oxygen compounds	Organooxygen compounds	Carbonyl compounds	Ketones	Aryl ketones	Phenylketones	Alkyl-phenylketones		FALSE	FALSE	FALSE	TRUE	FALSE	FALSE	FALSE	1	0	0	FALSE	FALSE
2663	InChI=1S/C20H33N3O4/c1-7-23(8-2)19(26)22-15-9-10-18(17(11-15)14(3)24)27-13-16(25)12-21-20(4,5)6/h9-11,16,21,25H,7-8,12-13H2,1-6H3,(H,22,26)	Organic compounds	Organic oxygen compounds	Organooxygen compounds	Carbonyl compounds	Ketones	Aryl ketones	Phenylketones	Alkyl-phenylketones		FALSE	FALSE	FALSE	FALSE	TRUE	FALSE	FALSE	0	1	0	FALSE	FALSE
3-acetylphenol sulfate		Organic compounds	Organic oxygen compounds	Organooxygen compounds	Carbonyl compounds	Ketones	Aryl ketones	Phenylketones	Alkyl-phenylketones		FALSE	FALSE	FALSE	FALSE	TRUE	FALSE	FALSE	0	1	0	FALSE	FALSE
5hxkynam	InChI=1S/C9H12N2O2/c10-4-3-9(13)7-5-6(12)1-2-8(7)11/h1-2,5,12H,3-4,10-11H2/p+1	Organic compounds	Organic oxygen compounds	Organooxygen compounds	Carbonyl compounds	Ketones	Aryl ketones	Phenylketones	Alkyl-phenylketones		TRUE	FALSE	TRUE	TRUE	FALSE	FALSE	FALSE	1	0	0	FALSE	FALSE
Lkynr	InChI=1S/C10H12N2O3/c11-7-4-2-1-3-6(7)9(13)5-8(12)10(14)15/h1-4,8H,5,11-12H2,(H,14,15)	Organic compounds	Organic oxygen compounds	Organooxygen compounds	Carbonyl compounds	Ketones	Aryl ketones	Phenylketones	Alkyl-phenylketones		TRUE	TRUE	TRUE	TRUE	TRUE	TRUE	TRUE	3	3	1	FALSE	FALSE
hLkynr	InChI=1S/C10H12N2O4/c11-6(10(15)16)4-8(14)5-2-1-3-7(13)9(5)12/h1-3,6,13H,4,11-12H2,(H,15,16)/t6-/m0/s1	Organic compounds	Organic oxygen compounds	Organooxygen compounds	Carbonyl compounds	Ketones	Aryl ketones	Phenylketones	Alkyl-phenylketones		TRUE	FALSE	TRUE	TRUE	FALSE	TRUE	FALSE	2	0	0	FALSE	FALSE
51397980	InChI=1S/C26H38O4/c1-16(2)9-10-20-23(28)22(21(27)15-19(7)8)25(30)26(24(20)29,13-11-17(3)4)14-12-18(5)6/h9,11-12,19-20,30H,10,13-15H2,1-8H3	Organic compounds	Organic oxygen compounds	Organooxygen compounds	Carbonyl compounds	Ketones	Cyclic ketones	Quinones	Benzoquinones	M-benzoquinones	FALSE	FALSE	FALSE	TRUE	FALSE	FALSE	FALSE	1	0	0	FALSE	FALSE
1,2,3-benzenetriol sulfate (1)		Organic compounds	Organic oxygen compounds	Organooxygen compounds	ester	sulfuric ester	aryl sulfate				FALSE	FALSE	FALSE	TRUE	FALSE	FALSE	FALSE	1	0	0	FALSE	FALSE
im4ac	InChI=1S/C5H6N2O2/c8-5(9)1-4-2-6-3-7-4/h2-3H,1H2,(H,6,7)(H,8,9)/p-1	Organic compounds	Organoheterocyclic compounds	Azoles	Imidazoles	Substituted imidazoles	Imidazolyl carboxylic acids and derivatives				TRUE	TRUE	TRUE	TRUE	FALSE	TRUE	FALSE	2	0	0	FALSE	FALSE
urcan	InChI=1S/C6H6N2O2/c9-6(10)2-1-5-3-7-	Organic compound	Organoheterocyclic	Azoles	Imidazoles	Substituted imidazoles	Imidazolyl				TRUE	TRUE	TRUE	TRUE	FALSE	TRUE	FALSE	3	0	0	FALSE	FALSE

	4-8-5/h1-4H,(H,7,8)(H,9,10)/p-1	s	compounds				carboxylic acids and derivatives												
3mldz	InChI=1S/C6H8N2O/c1-8-4-6(2-3-9)7-5-8/h3-5H,2H2,1H3	Organic compounds	Organoheterocyclic compounds	Azoles	Imidazoles	Substituted imidazoles	N-substituted imidazoles	TRUE	TRUE	TRUE	FALSE	TRUE	FALSE	FALSE	0	1	0	FALSE	FALSE
155805	InChI=1S/C27H29FN4O/c28-22-9-5-21(6-10-22)19-32-26-4-2-1-3-25(26)30-27(32)29-23-14-17-31(18-15-23)16-13-20-7-11-24(33)12-8-20/h1-12,23,33H,13-19H2,(H,29,30)	Organic compounds	Organoheterocyclic compounds	Benzimidazoles				FALSE	FALSE	FALSE	FALSE	TRUE	FALSE	FALSE	0	1	0	FALSE	FALSE
CE1926	InChI=1S/C15H20O4/c1-9-10(2)14-11(8-12(9)16)4-6-15(3,19-14)7-5-13(17)18/h8,16H,4-7H2,1-3H3,(H,17,18)/p-1	Organic compounds	Organoheterocyclic compounds	Benzopyrans	1-benzopyrans			TRUE	FALSE	TRUE	FALSE	TRUE	FALSE	FALSE	0	1	0	FALSE	FALSE
biopyrrin		Organic compounds	Organoheterocyclic compounds	Carboxylic acids and derivatives	Amino acids, peptides, and alogues			FALSE	FALSE	FALSE	TRUE	FALSE	FALSE	FALSE	1	0	0	FALSE	FALSE
56dthm	InChI=1S/C5H8N2O2/c1-3-2-6-5(9)7-4(3)8/h3H,2H2,1H3,(H2,6,7,8,9)	Organic compounds	Organoheterocyclic compounds	Diazines	Pyrimidines and pyrimidine derivatives	Hydropyrimidines		TRUE	TRUE	TRUE	FALSE	TRUE	FALSE	FALSE	0	1	0	FALSE	FALSE
108214	InChI=1S/C8H10N4O4/c1-4(14)10-5-6(9-3-13)11-8(16)12(2)7(5)15/h3H,1-2H3,(H,9,13)(H,10,14)(H,11,16)	Organic compounds	Organoheterocyclic compounds	Diazines	Pyrimidines and pyrimidine derivatives	Hydroxypyrimidines		FALSE	FALSE	FALSE	FALSE	TRUE	FALSE	TRUE	0	2	0	FALSE	FALSE
thym	InChI=1S/C5H6N2O2/c1-3-2-6-5(9)7-4(3)8/h2H,1H3,(H2,6,7,8,9)	Organic compounds	Organoheterocyclic compounds	Diazines	Pyrimidines and pyrimidine derivatives	Hydroxypyrimidines		TRUE	FALSE	TRUE	TRUE	FALSE	FALSE	FALSE	1	0	0	FALSE	FALSE
1201482	InChI=1S/C4H6N4S/c5-2-1-3(6)8-4(9)7-2/h1H,(H5,5,6,7,8,9)	Organic compounds	Organoheterocyclic compounds	Diazines	Pyrimidines and pyrimidine derivatives	Pyrimidinethiones		FALSE	FALSE	FALSE	FALSE	TRUE	FALSE	FALSE	0	1	0	FALSE	FALSE
70122	InChI=1S/C6H8N2O2/c1-7-4-3-5(9)8(2)6(7)10/h3-4H,1-2H3	Organic compounds	Organoheterocyclic compounds	Diazines	Pyrimidines and pyrimidine derivatives	Pyrimidones		FALSE	FALSE	FALSE	TRUE	FALSE	FALSE	FALSE	1	0	0	FALSE	FALSE
ura	InChI=1S/C4H4N2O2/c7-3-1-2-5-4(8)6-3/h1-2H,(H2,5,6,7,8)	Organic compounds	Organoheterocyclic compounds	Diazines	Pyrimidines and pyrimidine derivatives	Pyrimidones		TRUE	TRUE	TRUE	TRUE	FALSE	FALSE	FALSE	1	0	0	FALSE	FALSE
ascb_L	InChI=1S/C6H8O6/c7-1-2(8)5-3(9)4(10)6(11)12-5/h2,5,7-10H,1H2/p-1/t2-,5+/m0/s1	Organic compounds	Organoheterocyclic compounds	Dihydrofurans	Furanones	Butenolides		TRUE	TRUE	TRUE	TRUE	TRUE	FALSE	FALSE	1	1	1	FALSE	FALSE
ade	InChI=1S/C5H5N5/c6-4-3-5(9-1-7-3)10-2-8-4/h1-2H,(H3,6,7,8,9,10)	Organic compounds	Organoheterocyclic compounds	Imidazopyrimidines	Purines and purine derivatives	6-aminopurines		TRUE	TRUE	TRUE	TRUE	FALSE	FALSE	FALSE	1	0	0	TRUE	FALSE
70315	InChI=1S/C6H7N5O/c1-11-5(12)3-4(9-2-8-3)10-6(11)7/h2H,1H3,(H2,7,10)(H,8,9)	Organic compounds	Organoheterocyclic compounds	Imidazopyrimidines	Purines and purine derivatives	Purinones	6-oxopurines	FALSE	FALSE	FALSE	TRUE	FALSE	FALSE	FALSE	1	0	0	FALSE	FALSE
135398679	InChI=1S/C6H7N5O/c1-11-2-8-4-3(11)5(12)10-6(7)9-4/h2H,1H3,(H3,7,9,10,12)	Organic compounds	Organoheterocyclic compounds	Imidazopyrimidines	Purines and purine derivatives	Purinones	Hypoxanthines	FALSE	FALSE	TRUE	TRUE	FALSE	FALSE	FALSE	1	0	0	FALSE	FALSE
135414099	InChI=1S/C5H4N4O2/c10-4-2-3(6-1-7-4)9-5(11)8-2/h1H,(H3,6,7,8,9,10,11)	Organic compounds	Organoheterocyclic compounds	Imidazopyrimidines	Purines and purine derivatives	Purinones	Hypoxanthines	FALSE	FALSE	FALSE	FALSE	TRUE	FALSE	FALSE	0	1	0	FALSE	FALSE
hxan	InChI=1S/C5H4N4O/c10-5-3-4(7-1-6-3)8-2-9-5/h1-2H,(H2,6,7,8,9,10)	Organic compounds	Organoheterocyclic compounds	Imidazopyrimidines	Purines and purine derivatives	Purinones	Hypoxanthines	TRUE	FALSE	TRUE	TRUE	TRUE	TRUE	FALSE	2	1	1	FALSE	FALSE
2153	InChI=1S/C7H8N4O2/c1-10-5-4(8-3-9-5)6(12)11(2)7(10)13/h3H,1-2H3,(H,8,9)	Organic compounds	Organoheterocyclic compounds	Imidazopyrimidines	Purines and purine derivatives	Xanthines		FALSE	FALSE	FALSE	TRUE	FALSE	FALSE	TRUE	1	6	1	FALSE	FALSE
2519	InChI=1S/C8H10N4O2/c1-10-4-9-6-5(10)7(13)12(3)8(14)11(6)2/h4H,1-3H3	Organic compounds	Organoheterocyclic compounds	Imidazopyrimidines	Purines and purine derivatives	Xanthines		FALSE	FALSE	FALSE	FALSE	TRUE	FALSE	TRUE	0	5	0	FALSE	FALSE

4687	InChI=1S/C7H8N4O2/c1-10-3-8-5-4(10)6(12)11(2)7(13)9-5/h3H,1-2H3,(H,9,13)	Organic compounds	Organoheterocyclic compounds	Imidazopyrimidines	Purines and purine derivatives	Xanthines	FALSE	FALSE	FALSE	FALSE	TRUE	FALSE	TRUE	0	5	0	FALSE	FALSE
5429	InChI=1S/C7H8N4O2/c1-10-3-8-5-4(10)6(12)9-7(13)11(5)2/h3H,1-2H3,(H,9,12,13)	Organic compounds	Organoheterocyclic compounds	Imidazopyrimidines	Purines and purine derivatives	Xanthines	FALSE	FALSE	FALSE	FALSE	TRUE	FALSE	TRUE	0	2	0	FALSE	FALSE
68374	InChI=1S/C6H6N4O2/c1-10-2-7-4-3(10)5(11)9-6(12)8-4/h2H,1H3,(H2,8,9,11,12)	Organic compounds	Organoheterocyclic compounds	Imidazopyrimidines	Purines and purine derivatives	Xanthines	FALSE	FALSE	FALSE	FALSE	TRUE	FALSE	TRUE	0	2	0	FALSE	FALSE
70346	InChI=1S/C7H8N4O3/c1-10-4-3(8-6(13)9-4)5(12)11(2)7(10)14/h1-2H3,(H2,8,9,13)	Organic compounds	Organoheterocyclic compounds	Imidazopyrimidines	Purines and purine derivatives	Xanthines	FALSE	FALSE	FALSE	FALSE	TRUE	FALSE	TRUE	0	2	0	FALSE	FALSE
70639	InChI=1S/C6H6N4O2/c1-10-4-3(7-2-8-4)5(11)9-6(10)12/h2H,1H3,(H,7,8)(H,9,11,12)	Organic compounds	Organoheterocyclic compounds	Imidazopyrimidines	Purines and purine derivatives	Xanthines	FALSE	FALSE	FALSE	FALSE	TRUE	FALSE	TRUE	0	2	0	FALSE	FALSE
79437	InChI=1S/C8H10N4O3/c1-10-4-5(9-7(10)14)11(2)8(15)12(3)6(4)13/h1-3H3,(H,9,14)	Organic compounds	Organoheterocyclic compounds	Imidazopyrimidines	Purines and purine derivatives	Xanthines	FALSE	FALSE	FALSE	FALSE	TRUE	FALSE	TRUE	0	2	0	FALSE	FALSE
80220	InChI=1S/C6H6N4O2/c1-10-5(11)3-4(8-2-7-3)9-6(10)12/h2H,1H3,(H,7,8)(H,9,12)	Organic compounds	Organoheterocyclic compounds	Imidazopyrimidines	Purines and purine derivatives	Xanthines	FALSE	FALSE	FALSE	FALSE	TRUE	FALSE	TRUE	0	5	0	FALSE	FALSE
91611	InChI=1S/C7H8N4O3/c1-10-3-4(8-6(10)13)9-7(14)11(2)5(3)12/h1-2H3,(H,8,13)(H,9,14)	Organic compounds	Organoheterocyclic compounds	Imidazopyrimidines	Purines and purine derivatives	Xanthines	FALSE	FALSE	FALSE	FALSE	TRUE	FALSE	TRUE	0	4	0	FALSE	FALSE
urate	InChI=1S/C5H4N4O3/c10-3-1-2(7-4(11)6-1)8-5(12)9-3/h(H4,6,7,8,9,10,11,12)	Organic compounds	Organoheterocyclic compounds	Imidazopyrimidines	Purines and purine derivatives	Xanthines	TRUE	FALSE	TRUE	TRUE	TRUE	FALSE	TRUE	1	3	1	FALSE	FALSE
xan	InChI=1S/C5H4N4O2/c10-4-2-3(7-1-6-2)8-5(11)9-4/h1H,(H3,6,7,8,9,10,11)	Organic compounds	Organoheterocyclic compounds	Imidazopyrimidines	Purines and purine derivatives	Xanthines	TRUE	TRUE	TRUE	TRUE	TRUE	TRUE	FALSE	2	1	1	FALSE	FALSE
170404	InChI=1S/C9H7N/c1-7-6-10-9-5-3-2-4-8(7)9/h2-6H,1H2	Organic compounds	Organoheterocyclic compounds	Indoles and derivatives			FALSE	FALSE	FALSE	TRUE	FALSE	FALSE	FALSE	1	0	0	FALSE	FALSE
CE5536	InChI=1S/C4H8O3/c1-3(5)2-4(6)7/h3,5H,2H2,1H3,(H,6,7)/p-1	Organic compounds	Organoheterocyclic compounds	Indoles and derivatives			TRUE	FALSE	TRUE	TRUE	FALSE	FALSE	FALSE	1	0	0	FALSE	FALSE
CE4888	InChI=1S/C8H7NO2/c10-7-3-5-1-2-9-6(5)4-8(7)11/h1-4,9-11H	Organic compounds	Organoheterocyclic compounds	Indoles and derivatives	Hydroxyindoles		TRUE	TRUE	TRUE	TRUE	FALSE	FALSE	FALSE	1	0	0	FALSE	FALSE
Nacsertn	InChI=1S/C12H14N2O2/c1-8(15)13-5-4-9-7-14-12-3-2-10(16)6-11(9)12/h2-3,6-7,14,16H,4-5H2,1H3,(H,13,15)	Organic compounds	Organoheterocyclic compounds	Indoles and derivatives	Hydroxyindoles		TRUE	FALSE	TRUE	TRUE	FALSE	FALSE	FALSE	1	0	0	FALSE	FALSE
indole	InChI=1S/C8H7N/c1-2-4-8-7(3-1)5-6-9-8/h1-6,9H	Organic compounds	Organoheterocyclic compounds	Indoles and derivatives	Indoles		TRUE	FALSE	TRUE	TRUE	TRUE	FALSE	FALSE	1	1	1	FALSE	FALSE
21775807	InChI=1S/C17H22N2O7/c18-9(17(24)25)5-8-7-3-1-2-4-10(7)19-12(8)16-15(23)14(22)13(21)11(6-20)26-16/h1-4,9,11,13-16,19-23H,5-6,18H2,(H,24,25)/t9-,11?,13?,14?,15?,16?/m0/s1	Organic compounds	Organoheterocyclic compounds	Indoles and derivatives	Indolyl carboxylic acids and derivatives		FALSE	FALSE	FALSE	TRUE	FALSE	FALSE	FALSE	1	0	0	FALSE	FALSE
92904	InChI=1S/C11H11NO3/c13-10(11(14)15)5-7-6-12-9-4-2-1-3-8(7)9/h1-4,6,10,12-13H,5H2,(H,14,15)	Organic compounds	Organoheterocyclic compounds	Indoles and derivatives	Indolyl carboxylic acids and derivatives		FALSE	FALSE	FALSE	TRUE	TRUE	FALSE	FALSE	1	1	1	FALSE	FALSE
trp_L	InChI=1S/C11H12N2O2/c12-9(11(14)15)5-7-6-13-10-4-2-1-3-8(7)10/h1-4,6,9,13H,5,12H2,(H,14,15)/t9-/m0/s1	Organic compounds	Organoheterocyclic compounds	Indoles and derivatives	Indolyl carboxylic acids and derivatives		TRUE	TRUE	TRUE	TRUE	FALSE	FALSE	TRUE	1	4	1	TRUE	FALSE

5hoxindoa	InChI=1S/C10H9NO3/c12-7-1-2-9-8(4-7)6(5-11-9)3-10(13)14/h1-2,4-5,11-12H,3H2,(H,13,14)/p-1	Organic compounds	Organoheterocyclic compounds	Indoles and derivatives	Indolyl carboxylic acids and derivatives	Indole-3-acetic acid derivatives		TRUE	FALSE	TRUE	FALSE	TRUE	FALSE	FALSE	0	1	0	FALSE	FALSE
CE2122	InChI=1S/C11H11NO2/c1-14-11(13)6-8-7-12-10-5-3-2-4-9(8)10/h2-5,7,12H,6H2,1H3	Organic compounds	Organoheterocyclic compounds	Indoles and derivatives	Indolyl carboxylic acids and derivatives	Indole-3-acetic acid derivatives		TRUE	FALSE	TRUE	FALSE	TRUE	FALSE	FALSE	0	1	0	FALSE	FALSE
ind3ac	InChI=1S/C10H9NO2/c12-10(13)5-7-6-11-9-4-2-1-3-8(7)9/h1-4,6,11H,5H2,(H,12,13)/p-1	Organic compounds	Organoheterocyclic compounds	Indoles and derivatives	Indolyl carboxylic acids and derivatives	Indole-3-acetic acid derivatives		TRUE	TRUE	TRUE	TRUE	TRUE	FALSE	TRUE	1	3	1	FALSE	FALSE
11781	InChI=1S/C9H9N/c1-10-7-6-8-4-2-3-5-9(8)10/h2-7H,1H3	Organic compounds	Organoheterocyclic compounds	Indoles and derivatives	N-alkylindoles			FALSE	FALSE	FALSE	TRUE	FALSE	FALSE	FALSE	1	0	0	FALSE	FALSE
355436	InChI=1S/C17H21N3/c1-2-5-14-12(4-1)13-7-11-20-15(17(13)18-14)8-10-19-9-3-6-16(19)20/h1-2,4-5,15-16,18H,3,6-11H2/t15-,16-/m0/s1	Organic compounds	Organoheterocyclic compounds	Indoles and derivatives	Pyridoindoles	Beta carbolines		FALSE	FALSE	FALSE	FALSE	TRUE	FALSE	FALSE	0	1	0	FALSE	FALSE
CE2152	InChI=1S/C11H12N2/c1-2-4-10-8(3-1)9-5-6-12-7-11(9)13-10/h1-4,12-13H,5-7H2/p+1	Organic compounds	Organoheterocyclic compounds	Indoles and derivatives	Pyridoindoles	Beta carbolines		TRUE	FALSE	TRUE	TRUE	FALSE	FALSE	FALSE	1	0	0	FALSE	FALSE
trypta	InChI=1S/C10H12N2/c11-6-5-8-7-12-10-4-2-1-3-9(8)10/h1-4,7,12H,5-6,11H2/p+1	Organic compounds	Organoheterocyclic compounds	Indoles and derivatives	Tryptamines and derivatives			TRUE	TRUE	TRUE	TRUE	TRUE	FALSE	FALSE	1	1	1	FALSE	FALSE
5htrp	InChI=1S/C11H12N2O3/c12-9(11(15)16)3-6-5-13-10-2-1-7(14)4-8(6)10/h1-2,4-5,9,13-14H,3,12H2,(H,15,16)	Organic compounds	Organoheterocyclic compounds	Indoles and derivatives	Tryptamines and derivatives	Serotonins		TRUE	TRUE	TRUE	TRUE	FALSE	TRUE	FALSE	4	0	0	FALSE	FALSE
srtn	InChI=1S/C10H12N2O/c11-4-3-7-6-12-10-2-1-8(13)5-9(7)10/h1-2,5-6,12-13H,3-4,11H2/p+1	Organic compounds	Organoheterocyclic compounds	Indoles and derivatives	Tryptamines and derivatives	Serotonins		TRUE	TRUE	TRUE	TRUE	TRUE	FALSE	TRUE	1	3	1	FALSE	FALSE
290378	InChI=1S/C9H7NO/c11-10-6-5-8-3-1-2-4-9(8)7-10/h1-7H	Organic compounds	Organoheterocyclic compounds	Isoquinolines and derivatives				FALSE	FALSE	FALSE	TRUE	FALSE	FALSE	FALSE	1	0	0	FALSE	FALSE
23672568	InChI=1S/C18H18N6O5S2.Na/c1-23-18(20-21-22-23)31-8-10-7-30-16-11(15(27)24(16)12(10)17(28)29)19-14(26)13(25)9-5-3-2-4-6-9;/h2-6,11,13,16,25H,7-8H2,1H3,(H,19,26)(H,28,29);/q;+1/p-1/t11-,13-,16-;/m1./s1	Organic compounds	Organoheterocyclic compounds	Lactams	Beta lactams	Cephems	Cephalosporins	FALSE	FALSE	FALSE	FALSE	TRUE	FALSE	FALSE	0	1	0	FALSE	FALSE
bile acid or Lovastatin		Organic compounds	Organoheterocyclic compounds	Lactones	Delta valerolactones			FALSE	FALSE	FALSE	FALSE	TRUE	FALSE	FALSE	0	1	0	FALSE	FALSE
122306	InChI=1S/C6H8O7/c7-1-2(8)6(12)13-4(1)3(9)5(10)11/h1-4,7-9H,(H,10,11)/t1-,2-,3+,4+/m1/s1	Organic compounds	Organoheterocyclic compounds	Lactones	Gamma butyrolactones			FALSE	FALSE	FALSE	TRUE	FALSE	FALSE	FALSE	1	0	0	FALSE	FALSE
dhdascb	InChI=1S/C6H6O6/c7-1-2(8)5-3(9)4(10)6(11)12-5/h2,5,7-8H,1H2/t2-,5+/m0/s1	Organic compounds	Organoheterocyclic compounds	Lactones	Gamma butyrolactones			TRUE	TRUE	TRUE	FALSE	TRUE	FALSE	FALSE	0	1	0	TRUE	FALSE
135403659	InChI=1S/C9H11N5O3/c1-3(15)6(16)4-2-11-7-5(12-4)8(17)14-9(10)13-7/h2-3,6,15-16H,1H3,(H3,10,11,13,14,17)/t3-,6-/m0/s1	Organic compounds	Organoheterocyclic compounds	Pteridines and derivatives	Pterins and derivatives	Biopterins and derivatives		FALSE	FALSE	FALSE	TRUE	FALSE	FALSE	FALSE	1	0	0	FALSE	FALSE
M00929	InChI=1S/C10H15N3O2/c1-13(12-15)7-3-5-10(14)9-4-2-6-11-8-9/h2,4,6,8,10,14H,3,5,7H2,1H3	Organic compounds	Organoheterocyclic compounds	Pyridines and derivatives				TRUE	FALSE	TRUE	FALSE	TRUE	FALSE	FALSE	0	1	0	FALSE	FALSE
4pyrdx	InChI=1S/C8H9NO4/c1-4-7(11)6(8(12)13)5(3-10)2-9-4/h2,10-11H,3H2,1H3,(H,12,	Organic compounds	Organoheterocyclic compounds	Pyridines and derivatives	Pyridinecarboxylic acids and derivatives	Pyridinecarboxylic acids		TRUE	FALSE	TRUE	TRUE	TRUE	TRUE	TRUE	2	2	1	FALSE	FALSE

	13)																	
nac	InChI=1S/C6H5NO2/c8-6(9)5-2-1-3-7-4-5/h1-4H,(H,8,9)/p-1	Organic compounds	Organoheterocyclic compounds	Pyridines and derivatives	Pyridinecarboxylic acids and derivatives	Pyridinecarboxylic acids	TRUE	TRUE	TRUE	TRUE	TRUE	FALSE	TRUE	1	2	1	FALSE	FALSE
quln	InChI=1S/C7H5NO4/c9-6(10)4-2-1-3-8-5(4)7(11)12/h1-3H,(H,9,10)(H,11,12)/p-2	Organic compounds	Organoheterocyclic compounds	Pyridines and derivatives	Pyridinecarboxylic acids and derivatives	Pyridinecarboxylic acids	TRUE	FALSE	TRUE	TRUE	FALSE	FALSE	FALSE	1	0	0	FALSE	FALSE
129397	InChI=1S/C9H16N2O2/c1-8(12)10-5-3-7-11-6-2-4-9(11)13/h2-7H2,1H3,(H,10,12)	Organic compounds	Organoheterocyclic compounds	Pyrrolidines	N-alkylpyrrolidines		FALSE	FALSE	FALSE	FALSE	TRUE	FALSE	FALSE	0	1	0	FALSE	FALSE
C02470	InChI=1S/C10H7NO4/c12-7-3-1-2-5-8(13)4-6(10(14)15)11-9(5)7/h1-4,12H,(H,13)(H,14,15)/p-1	Organic compounds	Organoheterocyclic compounds	Quinolines and derivatives	Quinoline carboxylic acids		TRUE	FALSE	TRUE	TRUE	FALSE	TRUE	FALSE	2	0	0	FALSE	FALSE
kynate	InChI=1S/C10H7NO3/c12-9-5-8(10(13)14)11-7-4-2-1-3-6(7)9/h1-5H,(H,11,12)(H,13,14)/p-1	Organic compounds	Organoheterocyclic compounds	Quinolines and derivatives	Quinoline carboxylic acids		TRUE	TRUE	TRUE	FALSE	TRUE	FALSE	FALSE	0	1	0	FALSE	FALSE
440784	InChI=1S/C33H42N4O6/c1-7-20-19(6)32(42)37-27(20)14-25-18(5)23(10-12-31(40)41)29(35-25)15-28-22(9-11-30(38)39)17(4)24(34-28)13-26-16(3)21(8-2)33(43)36-26/h8,26-27,34-35H,2,7,9-15H2,1,3-6H3,(H,36,43)(H,37,42)(H,38,39)(H,40,41)	Organic compounds	Organoheterocyclic compounds	Tetrapyrroles and derivatives	Bilirubins		FALSE	FALSE	FALSE	TRUE	FALSE	FALSE	FALSE	1	0	0	FALSE	FALSE
bilirub	InChI=1S/C33H36N4O6/c1-7-20-19(6)32(42)37-27(20)14-25-18(5)23(10-12-31(40)41)29(35-25)15-28-22(9-11-30(38)39)17(4)24(34-28)13-26-16(3)21(8-2)33(43)36-26/h7-8,13-14,34-35H,1-2,9-12,15H2,3-6H3,(H,36,43)(H,37,42)(H,38,39)(H,40,41)/p-2/b26-13-,27-14-	Organic compounds	Organoheterocyclic compounds	Tetrapyrroles and derivatives	Bilirubins		TRUE	FALSE	TRUE	TRUE	TRUE	TRUE	FALSE	2	1	1	FALSE	TRUE
bilirubin (E,Z or Z,E)		Organic compounds	Organoheterocyclic compounds	Tetrapyrroles and derivatives	Bilirubins		FALSE	FALSE	FALSE	FALSE	TRUE	FALSE	FALSE	0	1	0	FALSE	FALSE
biliverd	InChI=1S/C33H34N4O6/c1-7-20-19(6)32(42)37-27(20)14-25-18(5)23(10-12-31(40)41)29(35-25)15-28-22(9-11-30(38)39)17(4)24(34-28)13-26-16(3)21(8-2)33(43)36-26/h7-8,13-15,35H,1-2,9-12H2,3-6H3,(H,36,43)(H,37,42)(H,38,39)(H,40,41)/p-2/b26-13-,27-14-,28-15-	Organic compounds	Organoheterocyclic compounds	Tetrapyrroles and derivatives	Bilirubins		TRUE	FALSE	TRUE	TRUE	TRUE	TRUE	FALSE	2	1	1	FALSE	TRUE
pheme	InChI=1S/C34H34N4O4.Fe/c1-7-21-17(3)25-13-26-19(5)23(9-11-33(39)40)31(37-26)16-32-24(10-12-34(41)42)20(6)28(38-32)15-30-22(8-2)18(4)27(36-30)14-29(21)35-25;/h7-8,13-16H,1-2,9-12H2,3-6H3,(H4,35,36,37,38,39,40,41,42);/q;+2/p-2	Organic compounds	Organoheterocyclic compounds	Tetrapyrroles and derivatives	Metallotetrapyrroles	Metalloporphyrins	TRUE	FALSE	TRUE	FALSE	TRUE	FALSE	FALSE	0	1	0	FALSE	TRUE
443066	InChI=1S/C5H8O2S/c6-5(7)4-2-1-3-8-4/h4H,1-3H2,(H,6,7)	Organic compounds	Organoheterocyclic compounds	Thiolanes			FALSE	FALSE	FALSE	FALSE	TRUE	FALSE	FALSE	0	1	0	FALSE	FALSE
71423	InChI=1S/C17H14ClN3O2S/c1-10-7-13(21-17(23)20-15(22)9-19-21)8-11(2)16(10	Organic compounds	Organosulfur compounds	Thioethers	Aryl thioethers	Diarylthioethers	FALSE	FALSE	FALSE	FALSE	TRUE	FALSE	FALSE	0	1	0	FALSE	FALSE

	)24-14-5-3-12(18)4-6-14/h3-9H,1-2H3,(H,20,22,23)																		
131751022	InChI=1S/C35H50N8O10/c1-50-23-17-19(6-11-25(45)40-12-2-4-14-42-34(36)37)16-22-26(32(49)41-13-3-5-15-43-35(38)39)30(53-31(22)23)20-7-9-21(10-8-20)51-33-29(48)28(47)27(46)24(18-44)52-33/h6-11,16-17,24,26-30,33,44,46-48H,2-5,12-15,18H2,1H3,(H,40,45)(H,41,49)(H4,36,37,42)(H4,38,39,43)/b11-6-	Organic compounds	Phenylpropanoids and polyketides	2-arylbenzofuran flavonoids				FALSE	FALSE	FALSE	FALSE	TRUE	FALSE	FALSE	0	1	0	FALSE	FALSE
5372954	InChI=1S/C9H8O2/c10-9(11)7-6-8-4-2-1-3-5-8/h1-7H,(H,10,11)/b7-6-	Organic compounds	Phenylpropanoids and polyketides	Cinmic acids and derivatives	Cinmic acids			FALSE	FALSE	FALSE	FALSE	TRUE	FALSE	FALSE	0	1	0	FALSE	FALSE
122198225	InChI=1S/C9H8O7S/c10-7-3-1-6(2-4-9(11)12)5-8(7)16-17(13,14)15/h1-5,10H,(H,11,12)(H,13,14,15)/p-2/b4-2+	Organic compounds	Phenylpropanoids and polyketides	Cinmic acids and derivatives	Hydroxycinnamic acids and derivatives	Coumaric acids and derivatives		FALSE	FALSE	FALSE	FALSE	TRUE	FALSE	FALSE	0	1	0	FALSE	FALSE
42607894	InChI=1S/C32H40O15/c1-13(2)3-8-16-20(45-32-29(42)27(40)25(38)22(12-34)47-32)10-18(36)23-17(35)9-19(44-30(16)23)14-4-6-15(7-5-14)43-31-28(41)26(39)24(37)21(11-33)46-31/h3-7,10,19,21-22,24-29,31-34,36-42H,8-9,11-12H2,1-2H3	Organic compounds	Phenylpropanoids and polyketides	Flavonoids	Flavonoid glycosides	Flavonoid O-glycosides	Flavonoid-7-O-glycosides	FALSE	FALSE	FALSE	FALSE	TRUE	FALSE	FALSE	0	1	0	FALSE	FALSE
88881	InChI=1S/C17H14O4/c1-19-12-8-15(20-2)17-13(18)10-14(21-16(17)9-12)11-6-4-3-5-7-11/h3-10H,1-2H3	Organic compounds	Phenylpropanoids and polyketides	Flavonoids	O-methylated flavonoids	7-O-methylated flavonoids		FALSE	FALSE	FALSE	TRUE	FALSE	FALSE	FALSE	1	0	0	FALSE	FALSE
11013898	InChI=1S/C21H14O6/c1-9(2)13-4-10-3-12-16(6-14(10)25-13)27-21(22)19-11-5-17-18(24-8-23-17)7-15(11)26-20(12)19/h3,5-7,13H,1,4,8H2,2H3	Organic compounds	Phenylpropanoids and polyketides	Isoflavonoids	Coumestans			FALSE	FALSE	FALSE	TRUE	FALSE	TRUE	FALSE	2	0	0	FALSE	FALSE
2coum	InChI=1S/C9H8O3/c10-8-4-2-1-3-7(8)5-6-9(11)12/h1-6,10H,(H,11,12)/p-1/b6-5-	Organic compounds	Phenylpropanoids and polyketides	Organonitrogen compounds	Amines	Hydroxycinnamic acids		TRUE	FALSE	TRUE	TRUE	FALSE	FALSE	FALSE	1	0	0	FALSE	FALSE
34hpl	InChI=1S/C9H10O4/c10-7-3-1-6(2-4-7)5-8(11)9(12)13/h1-4,8,10-11H,5H2,(H,12,13)/p-1	Organic compounds	Phenylpropanoids and polyketides	Phenylpropanoic acids				TRUE	TRUE	TRUE	TRUE	FALSE	TRUE	FALSE	2	0	0	FALSE	FALSE
38853	InChI=1S/C10H13NO4/c1-10(11,9(14)15)5-6-2-3-7(12)8(13)4-6/h2-4,12-13H,5,11H2,1H3,(H,14,15)/t10-/m0/s1	Organic compounds	Phenylpropanoids and polyketides	Phenylpropanoic acids				FALSE	FALSE	FALSE	TRUE	TRUE	FALSE	FALSE	1	1	1	FALSE	FALSE
91	InChI=1S/C9H10O3/c10-8-3-1-2-7(6-8)4-5-9(11)12/h1-3,6,10H,4-5H2,(H,11,12)	Organic compounds	Phenylpropanoids and polyketides	Phenylpropanoic acids				FALSE	FALSE	FALSE	FALSE	TRUE	FALSE	FALSE	0	1	0	FALSE	FALSE
phlac	InChI=1S/C9H10O3/c10-8(9(11)12)6-7-4-2-1-3-5-7/h1-5,8,10H,6H2,(H,11,12)/p-1	Organic compounds	Phenylpropanoids and polyketides	Phenylpropanoic acids				TRUE	TRUE	TRUE	TRUE	FALSE	TRUE	FALSE	4	0	0	FALSE	FALSE
vanillac	InChI=1S/C10H12O5/c1-15-9-5-6(2-3-7(9)11)4-8(12)10(13)14/h2-3,5,8,11-12H,4H2,1H3,(H,13,14)	Organic compounds	Phenylpropanoids and polyketides	Phenylpropanoic acids				TRUE	FALSE	TRUE	TRUE	TRUE	TRUE	FALSE	2	1	1	FALSE	FALSE
18804	InChI=1S/C21H27NO2/c1-4-20(23)24-21(17(2)16-22-3,19-13-9-6-10-14-19)15-18-11-7-5-8-12-18/h5	Organic compounds	Phenylpropanoids and polyketides	Stilbenes				FALSE	FALSE	FALSE	TRUE	FALSE	FALSE	FALSE	1	0	0	FALSE	FALSE

	-14,17,22H,4,15-16 H2,1-3H3															
60721	InChI=1S/C27H44N2O2/c1-8-9-25(24-12-16-27(17-13-24)31-21-19-29(5,6)7)22-23-10-14-26(15-11-23)30-20-18-28(2,3)4/h10-17,25H,8-9,18-22H2,1-7H3/q+2	Organic compounds	Phenylpropanoids and polyketides	Stilbenes	FALSE	FALSE	FALSE	TRUE	FALSE	FALSE	FALSE	1	0	0	FALSE	FALSE

Supplementary Table 4: All 213 progression-related metabolites.

ID	inchistring	kingdom	superclass	class	subclass	level5	level 6	level7	level8	crossmatchWithRecon3D	crossmatchWithiDNCT	VMH	increased	decreased	increased_highfreq	decreased_highfreq	incresed_Realfrequency	decresed_Realfrequency	inconsistent	BBBcross	BBBnoncros	Replicated
10-carbon fatty aldehyde		Organic compounds	Lipids and lipid-like molecules	Fatty Acyls	Fatty acids and conjugates					FALSE	FALSE	FALSE	TRUE	FALSE	FALSE	FALSE	1	0	0	FALSE	FALSE	FALSE
100472	InChI=1S/C19H16N2O/c22-19(21-18-8-4-5-13-20-18)14-15-9-11-17(12-10-15)16-6-2-1-3-7-16/h1-13H,14H2,(H,20,21,22)	Organic compounds	Benzenoids	Benzene and substituted derivatives	Biphenyls and derivatives					FALSE	FALSE	FALSE	FALSE	TRUE	FALSE	FALSE	0	1	0	FALSE	FALSE	FALSE
104222097	InChI=1S/C17H30N4/c1-5-17(6-2)7-8-21(13-17)16-19-11-15(12-20-16)10-18-9-14(3)4/h11-12,14,18H,5-10,13H2,1-4H3	Organic compounds	Organic nitrogen compounds	Organoheterocyclic compounds	Amines	Tertiary amines	Tertiary alkylarylamines	Dialkylarylamines		FALSE	FALSE	FALSE	TRUE	FALSE	FALSE	FALSE	1	0	0	FALSE	FALSE	FALSE
1044	InChI=1S/C5H4N4/c1-4-5(8-2-6-1)9-3-7-4/h1-3H,(H,6,7,8,9)	Organic compounds	Organoheterocyclic compounds	Imidazopyrimidines	Purines and purine derivatives					FALSE	FALSE	FALSE	FALSE	TRUE	FALSE	FALSE	0	1	0	FALSE	FALSE	FALSE
10443535	InChI=1S/C13H18O3/c1-9(12(14)15)11-6-4-10(5-7-11)8-13(2,3)16/h4-7,9,16H,8H2,1-3H3,(H,14,15)	Organic compounds	Phenylpropanoids and polyketides	Phenylpropanoic acids						FALSE	FALSE	FALSE	TRUE	FALSE	FALSE	FALSE	1	0	0	FALSE	FALSE	FALSE
108009	InChI=1S/C7H13NO4S/c1-5(10)8-6(7(11)12)4-13-3-2-9/h6,9H,2-4H2,1H3,(H,8,10)(H,11,12)/t6-/m0/s1	Organic compounds	Organic acids and derivatives	Carboxylic acids and derivatives	Amino acids, peptides, and analogues	Amino acids and derivatives	Alpha amino acids and derivatives	N-acyl-alpha amino acids and derivatives	N-acyl-alpha amino acids	FALSE	FALSE	FALSE	FALSE	TRUE	FALSE	FALSE	0	1	0	FALSE	FALSE	FALSE
10871	InChI=1S/C8H8ClNO/c1-6(11)10-8-4-2-7(9)3-5-8/h2-5H,1H3,(H,10,11)	Organic compounds	Benzenoids	Benzene and substituted derivatives	Anilides	Acetanilides	Haloacetanilides	P-haloacetanilides		FALSE	FALSE	FALSE	TRUE	FALSE	FALSE	FALSE	1	0	0	FALSE	FALSE	FALSE
11-carbon fatty acid		Organic compounds	Lipids and lipid-like molecules	Fatty Acyls	Fatty acids and conjugates					FALSE	FALSE	FALSE	TRUE	FALSE	FALSE	FALSE	1	0	0	FALSE	FALSE	FALSE
11744748	InChI=1S/C8H17NO/c1-2-8(10)7-5-3-4-6-9-7/h7-10H,2-6H2,1H3/t7-,8+/m0/s1	Organic compounds	Organoheterocyclic compounds	Piperidines						FALSE	FALSE	FALSE	FALSE	TRUE	FALSE	FALSE	0	1	0	FALSE	FALSE	FALSE
11953875	InChI=1S/C33H38N4O6/c1-7-20-19(6)32(42)37-27(20)14-25-18(5)23(10-12-31(40)41)29(35-25)15-28-22(9-11-30(38)39)17(4)24(34-28)13-26-16(3)21(8-2)33(43)36-26/h7-8,14-15,19,26,34-35H,2,9-13H2,1,3-6H3,(H,36,43)(H,38,39)(H,40,41)/b20-7-,25-14-,29-15-/t19-,26-/m1/s1	Organic compounds	Organoheterocyclic compounds	Tetrapyrroles and derivatives	Bilirubins					FALSE	FALSE	FALSE	TRUE	FALSE	FALSE	FALSE	1	0	0	FALSE	FALSE	FALSE
12-carbon fatty acid		Organic compounds	Lipids and lipid-like molecules	Fatty Acyls	Fatty acids and conjugates					FALSE	FALSE	FALSE	TRUE	FALSE	FALSE	FALSE	1	0	0	FALSE	FALSE	FALSE
120773	InChI=1S/C11H21NO/c1-8-3-4-10-9(7-13)5-12(2)6-11(8)10/h8-11,13H,3-7H2,1-2H3	Organic compounds	Organoheterocyclic compounds	Piperidines						FALSE	FALSE	FALSE	FALSE	TRUE	FALSE	FALSE	0	1	0	FALSE	FALSE	FALSE
122841	InChI=1S/C12H16O4/c1-4-5-8(13)11-9(14)6-10(16-3)7(2)12(11)15/h6,14-15H,4-5H2,1-3H3	Organic compounds	Organic oxygen compounds	Organooxygen compounds	Carbonyl compounds	Ketones	Aryl ketones	Phenylketones	Alkyl-phenylketones	FALSE	FALSE	FALSE	TRUE	FALSE	FALSE	FALSE	1	0	0	FALSE	FALSE	FALSE
13-carbon fatty acid		Organic compounds	Lipids and lipid-like molecules	Fatty Acyls	Fatty acids and conjugates	Long-chain fatty acids				FALSE	FALSE	FALSE	FALSE	TRUE	FALSE	FALSE	0	1	0	FALSE	FALSE	FALSE

131750724	InChI=1S/C10H13ClN2O4S/c1-2-5-12-10(15)13-18(16,17)7-3-4-8(11)9(14)6-7/h3-4,6,14H,2,5H2,1H3,(H2,12,13,15)	Organic compounds	Benzenoids	Benzene and substituted derivatives	Benzenesulfonamides					FALSE	FALSE	FALSE	TRUE	FALSE	FALSE	FALSE	1	0	0	FALSE	FALSE	FALSE
134340	InChI=1S/C6H7NO4S/c8-5(9)3-1-12-2-4(7-3)6(10)11/h3H,1-2H2,(H,8,9)(H,10,11)	Organic compounds	Organic acids and derivatives	Carboxylic acids and derivatives	Amino acids, peptides, and alogues	Amino acids and derivatives	Alpha amino acids and derivatives			FALSE	FALSE	FALSE	FALSE	TRUE	FALSE	FALSE	0	1	0	FALSE	FALSE	FALSE
135407175	InChI=1S/C10H13N5O6/c11-9-13-6-3(7(19)14-9)12-10(20)15(6)8-5(18)4(17)2(1-16)21-8/h2,4-5,8,16-18H,1H2,(H,12,20)(H3,11,13,14,19)/t2-,4-,5-,8-/m1/s1	Organic compounds	Nucleosides, nucleotides, and alogues	Purine nucleosides						FALSE	FALSE	FALSE	FALSE	TRUE	FALSE	FALSE	0	1	0	FALSE	FALSE	FALSE
14,15-DHET	InChI=1S/C20H34O4/c1-2-3-12-15-18(21)19(22)16-13-10-8-6-4-5-7-9-11-14-17-20(23)24/h4,6-7,9-10,13,18-19,21-22H,2-3,5,8,11-12,14-17H2,1H3,(H,23,24)/b6-4-,9-7-,13-10-	Organic compounds	Lipids and lipid-like molecules	Fatty Acyls	Eicosanoids	Hydroxy eicosatrienoic acids				FALSE	FALSE	FALSE	TRUE	FALSE	FALSE	FALSE	1	0	0	FALSE	FALSE	FALSE
14-carbon fatty acid		Organic compounds	Lipids and lipid-like molecules	Fatty Acyls	Fatty acids and conjugates	Long-chain fatty acids				FALSE	FALSE	FALSE	TRUE	TRUE	FALSE	FALSE	1	1	1	FALSE	FALSE	FALSE
156003	InChI=1S/C16H12ClNO4S/c1-9(15(19)20)21-11-3-5-12(6-4-11)22-16-18-13-7-2-10(17)8-14(13)23-16/h2-9H,1H3,(H,19,20)	Organic compounds	Benzenoids	Benzene and substituted derivatives	2-phenoxypropionic acids	Aryloxyphenoxypropionic acids				FALSE	FALSE	FALSE	TRUE	FALSE	FALSE	FALSE	1	0	0	FALSE	FALSE	FALSE
15dap	InChI=1S/C5H14N2/c6-4-2-1-3-5-7/h1-7H2	Organic compounds	Organic nitrogen compounds	Organoheterocyclic compounds	Amines	Primary amines	Monoalkylamines			FALSE	FALSE	TRUE	TRUE	FALSE	FALSE	FALSE	1	0	0	FALSE	FALSE	FALSE
16 carbon fatty acid		Organic compounds	Lipids and lipid-like molecules	Fatty Acyls	Fatty acids and conjugates	Long-chain fatty acids				FALSE	FALSE	FALSE	FALSE	TRUE	FALSE	FALSE	0	1	0	FALSE	FALSE	FALSE
164838	InChI=1S/C21H32O5/c1-19-7-5-13(23)9-12(19)3-4-14-15-6-8-21(26,17(25)11-22)20(15,2)10-16(24)18(14)19/h12,14-16,18,22,24,26H,3-11H2,1-2H3/t12-,14+,15+,16+,18-,19+,20+,21+/m1/s1	Organic compounds	Lipids and lipid-like molecules	Steroids and steroid derivatives	Hydroxysteroids	21-hydroxysteroids				FALSE	FALSE	FALSE	TRUE	FALSE	FALSE	FALSE	1	0	0	FALSE	FALSE	FALSE
168871	InChI=1S/C31H41N5O5/c1-16(2)26-28(38)35-11-7-10-24(35)31(40)36(26)29(39)30(41-31,17(3)4)33-27(37)19-12-21-20-8-6-9-22-25(20)18(14-32-22)13-23(21)34(5)15-19/h6,8-9,14,16-17,19,21,23-24,26,32,40H,7,10-13,15H2,1-5H3,(H,33,37)/t19-,21-,23-,24+,26+,30-,31+/m1/s1	Organic compounds	Alkaloids and derivatives	Ergoline and derivatives	Lysergic acids and derivatives	Ergopeptines				FALSE	FALSE	FALSE	TRUE	FALSE	FALSE	FALSE	1	0	0	FALSE	FALSE	FALSE
17395965	InChI=1S/C13H17ClN6OS/c1-8(11(21)16-10-6-5-9(14)7-15-10)22-12-17-18-19-20(12)13(2,3)4/h5-8H,1-4H3,(H,15,16,21)	Organic compounds	Organic nitrogen compounds	Organonitrogen compounds	N-arylamides					FALSE	FALSE	FALSE	FALSE	TRUE	FALSE	FALSE	0	1	0	FALSE	FALSE	FALSE
17ahprgstrn	InChI=1S/C21H30O3/c1-13(22)21(24)11-8-18-16-5-4-14-12-15(23)6-9-19(14,2)17(16)7-10-20(18,21)3/h12,16-18,24H,4-11H2,1-3H3/t16-,17+,18+,19+,20+,21+/m1/s1	Organic compounds	Lipids and lipid-like molecules	Steroids and steroid derivatives	Pregne steroids	Gluco/mineralocorticoids, progestogins and derivatives				TRUE	FALSE	TRUE	TRUE	FALSE	FALSE	FALSE	1	0	0	FALSE	FALSE	FALSE
18392195	InChI=1S/C8H14O7/c1-2-14-8-5(11)3(9)4(10)6(15-8)7(12)13/h3-6,8-11H,2H2,1H3,(H,12,13)/t3-,4-,5+,6-,8+/m0/s1	Organic compounds	Organic oxygen compounds	Organooxygen compounds	Carbonyl compounds	Sugar acids and derivatives	Glucuronic acid derivatives	Glucuronides	O-glucuronides	FALSE	FALSE	FALSE	FALSE	TRUE	FALSE	FALSE	0	1	0	FALSE	FALSE	FALSE
1p2cbxl	InChI=1S/C5H7NO2/c7-5(8)4-2-1-3-6-4/h1-3H2,(H,7,8)/p-1	Organic compounds	Organic nitrogen compounds	Organoheterocyclic compounds	Pyrrolines					TRUE	FALSE	TRUE	TRUE	FALSE	FALSE	FALSE	1	0	0	FALSE	FALSE	FALSE
20908	InChI=1S/C11H10O/c12-8-10-6-3-5-9-4-1-2-7-11(9)10/h1-7,12H,8H2	Organic compounds	Benzenoids	phthalenes						FALSE	FALSE	FALSE	FALSE	TRUE	FALSE	FALSE	0	1	0	FALSE	FALSE	FALSE
21863	InChI=1S/C7H7NO4/c9-6(10)4-8-7(11)5-2-1-3-12-5/h1-3H,4H2,(H,8,11)(H,9,10)	Organic compounds	Organic acids and derivatives	Carboxylic acids and derivatives	Amino acids, peptides, and alogues	Amino acids and derivatives	Alpha amino acids and derivatives	N-acyl-alpha amino acids and derivati	N-acyl-alpha amino acids	FALSE	FALSE	FALSE	TRUE	FALSE	FALSE	FALSE	1	0	0	FALSE	FALSE	FALSE

								ves													
21976318	InChI=1S/C10H20O3/c1-2-3-4-5-6-7-9(11)8-10(12)13/h9,11H,2-8H2,1H3,(H,12,13)/p-1	Organic compounds	Organic acids and derivatives	Hydroxy acids and derivatives	Medium-chain hydroxy acids and derivatives				FALSE	FALSE	FALSE	TRUE	FALSE	FALSE	FALSE	1	0	0	FALSE	FALSE	FALSE
2249	InChI=1S/C14H22N2O3/c1-10(2)16-8-12(17)9-19-13-5-3-11(4-6-13)7-14(15)18/h3-6,10,12,16-17H,7-9H2,1-2H3,(H2,15,18)	Organic compounds	Benzenoids	Benzene and substituted derivatives	Phenylacetamides				FALSE	FALSE	FALSE	FALSE	TRUE	FALSE	FALSE	0	1	0	FALSE	FALSE	FALSE
22833544	InChI=1S/C8H15N3O5S2/c9-4(7(14)11-1-6(12)13)2-17-18-3-5(10)8(15)16/h4-5H,1-3,9-10H2,(H,11,14)(H,12,13)(H,15,16)/t4?,5-/m0/s1	Organic compounds	Organic acids and derivatives	Carboxylic acids and derivatives	Amino acids, peptides, and alogues	Peptides	Dipeptides		FALSE	FALSE	FALSE	TRUE	FALSE	FALSE	FALSE	1	0	0	FALSE	FALSE	FALSE
23670318	InChI=1S/C16H16N4O8S.Na/c1-26-19-9(8-3-2-4-27-8)12(21)18-10-13(22)20-11(15(23)24)7(5-28-16(17)25)6-29-14(10)20;/h2-4,10,14H,5-6H2,1H3,(H2,17,25)(H,18,21)(H,23,24);/q;+1/p-1/b19-9-;/t10-,14-;/m1./s1	Organic compounds	Organic acids and derivatives	Carboxylic acids and derivatives	Amino acids, peptides, and alogues	Amino acids and derivatives	Alpha amino acids	N-acyl-alpha amino acids and derivatives	FALSE	FALSE	FALSE	TRUE	FALSE	FALSE	FALSE	1	0	0	FALSE	FALSE	FALSE
24726	InChI=1S/C3H4Cl2/c4-2-1-3-5/h1-2H,3H2/b2-1+	Organic compounds	Organohalogen compounds	Vinyl halides	Vinyl chlorides				FALSE	FALSE	FALSE	FALSE	TRUE	FALSE	FALSE	0	1	0	FALSE	FALSE	FALSE
24751	InChI=1S/C3H6O.C2H4O/c1-3-2-4-3;1-2-3-1/h3H,2H2,1H3;1-2H2	Organic compounds	Organoheterocyclic compounds	Epoxides					FALSE	FALSE	FALSE	FALSE	TRUE	FALSE	FALSE	0	1	0	FALSE	FALSE	FALSE
24779473	InChI=1S/C28H58NO7P/c1-5-6-7-8-9-10-11-12-13-14-15-16-17-18-19-20-21-22-28(31)34-25-27(30)26-36-37(32,33)35-24-23-29(2,3)4/h27,30H,5-26H2,1-4H3/t27-/m1/s1	Organic compounds	Lipids and lipid-like molecules	Glycerophospholipids	Glycerophosphocholines				FALSE	FALSE	FALSE	FALSE	TRUE	FALSE	FALSE	0	1	0	FALSE	FALSE	FALSE
248425	InChI=1S/C10H12N4O4/c15-2-6-7(16)8(17)10(18-6)14-4-13-5-1-11-3-12-9(5)14/h1,3-4,6-8,10,15-17H,2H2	Organic compounds	Nucleosides, nucleotides, and alogues	Purine nucleosides					FALSE	FALSE	FALSE	FALSE	TRUE	FALSE	FALSE	0	1	0	FALSE	FALSE	FALSE
25246329	InChI=1S/C22H13Cl2N3O4/c23-13-5-1-3-9-11(7-25-17(9)13)15-16(20(22(30)31)27-19(15)21(28)29)12-8-26-18-10(12)4-2-6-14(18)24/h1-8,25-27H,(H,28,29)(H,30,31)	Organic compounds	Organoheterocyclic compounds	Indoles and derivatives	Indoles				FALSE	FALSE	FALSE	FALSE	TRUE	FALSE	FALSE	0	1	0	FALSE	FALSE	FALSE
2543	InChI=1S/C21H26O2/c1-5-6-7-8-15-12-18(22)20-16-11-14(2)9-10-17(16)21(3,4)23-19(20)13-15/h9-13,22H,5-8H2,1-4H3	Organic compounds	Organoheterocyclic compounds	Benzopyrans	1-benzopyrans	Dibenzopyrans			FALSE	FALSE	FALSE	TRUE	FALSE	FALSE	FALSE	1	0	0	FALSE	FALSE	FALSE
26576	InChI=1S/C16H18N2O3/c1-18(2)16(19)17-12-4-6-14(7-5-12)21-15-10-8-13(20-3)9-11-15/h4-11H,1-3H3,(H,17,19)	Organic compounds	Benzenoids	Benzene and substituted derivatives	Diphenylethers				FALSE	FALSE	FALSE	FALSE	TRUE	FALSE	FALSE	0	1	0	FALSE	FALSE	FALSE
2762430	InChI=1S/C9H10O3/c1-6(10)4-7-2-3-8(11)9(12)5-7/h2-3,5,11-12H,4H2,1H3	Organic compounds	Benzenoids	Benzene and substituted derivatives	Phenylpropanes				FALSE	FALSE	FALSE	TRUE	FALSE	FALSE	FALSE	1	0	0	FALSE	FALSE	FALSE
2aobut	InChI=1S/C4H7NO3/c1-2(6)3(5)4(7)8/h3H,5H2,1H3,(H,7,8)/t3-/m0/s1	Organic compounds	Organic acids and derivatives	Carboxylic acids and derivatives	Amino acids, peptides, and alogues	Amino acids and derivatives	Alpha amino acids	L-alpha-amino acids	TRUE	FALSE	TRUE	TRUE	FALSE	FALSE	FALSE	1	0	0	FALSE	FALSE	FALSE
2hb	InChI=1S/C4H8O3/c1-2-3(5)4(6)7/h3,5H,2H2,1H3,(H,6,7)/p-1	Organic compounds	Lipids and lipid-like molecules	Fatty Acyls					TRUE	TRUE	TRUE	FALSE	TRUE	FALSE	FALSE	0	1	0	FALSE	FALSE	FALSE
31209	InChI=1S/C14H18O/c1-2-3-5-10-14(12-15)11-13-8-6-4-7-9-13/h4,6-9,11-12H,2-3,5,10H2,1H3	Organic compounds	Phenylpropanoids and polyketides	Cinnamaldehydes					FALSE	FALSE	FALSE	TRUE	FALSE	FALSE	FALSE	1	0	0	FALSE	FALSE	FALSE
3344189	InChI=1S/C4H7NO4/c6-3(7)1-5-2-4(8)9/h5H,1-2H2,(H,6,7)(H,8,9)/p-2	Organic compounds	Organic acids and derivatives	Carboxylic acids and derivatives	Amino acids, peptides, and alogues	Amino acids and derivatives	Alpha amino acids and derivatives	Alpha amino acids	FALSE	FALSE	FALSE	FALSE	TRUE	FALSE	FALSE	0	1	0	FALSE	FALSE	FALSE
3444	InChI=1S/C13H12BrCl2N3O/c14-9-4-13(20-5-9,6-19-8-17-7-18-19)11-2-1-10(15)3-12(11)16/h1-3,7-9H,4-6H2	Organic compounds	Benzenoids	Benzene and substituted derivatives	Halobenzenes	Chlorobenzenes	Dichlorobenzenes		FALSE	FALSE	FALSE	TRUE	FALSE	FALSE	FALSE	1	0	0	FALSE	FALSE	FALSE

411181	InChI=1S/C14H13Cl2NO/c15-11-5-1-9(2-6-11)13(17)14(18)10-3-7-12(16)8-4-10/h1-8,13-14,18H,17H2	Organic compounds	Phenylpropanoids and polyketides	Stilbenes						FALSE	FALSE	FALSE	TRUE	FALSE	FALSE	FALSE	1	0	0	FALSE	FALSE	FALSE
440732	InChI=1S/C10H11NO4/c12-8-3-1-7(2-4-8)5-9(13)11-6-10(14)15/h1-4,12H,5-6H2,(H,11,13)(H,14,15)	Organic compounds	Organic acids and derivatives	Carboxylic acids and derivatives	Amino acids, peptides, and analogues	Amino acids and derivatives	Alpha amino acids and derivatives	N-acyl-alpha amino acids and derivatives	N-acyl-alpha amino acids	FALSE	FALSE	FALSE	TRUE	FALSE	FALSE	FALSE	1	0	0	FALSE	FALSE	FALSE
440991	InChI=1S/C13H10O5/c14-8-3-1-2-7(4-8)13(18)12-10(16)5-9(15)6-11(12)17/h1-6,14-17H	Organic compounds	Benzenoids	Benzene and substituted derivatives	Benzophenones					FALSE	FALSE	FALSE	FALSE	TRUE	FALSE	FALSE	0	1	0	FALSE	FALSE	FALSE
441996	InChI=1S/C17H18N2O/c1-4-17(3)9-11(2)19-16(17)15(20)13-10-18-14-8-6-5-7-12(13)14/h4-8,10,16,18H,1,9H2,2-3H3/t16-,17+/m1/s1	Organic compounds	Organoheterocyclic compounds	Indoles and derivatives	Indoles					FALSE	FALSE	FALSE	FALSE	TRUE	FALSE	FALSE	0	1	0	FALSE	FALSE	FALSE
442416	InChI=1S/C22H26O11/c23-8-15-17(26)18(27)19(28)22(32-15)33-21-16-11(7-14(25)13(16)5-6-30-21)9-31-20(29)10-1-3-12(24)4-2-10/h1-7,13-19,21-28H,8-9H2/t13-,14+,15+,16+,17+,18-,19+,21-,22-/m0/s1	Organic compounds	Lipids and lipid-like molecules	Prenol lipids	Terpene glycosides	Iridoid O-glycosides				FALSE	FALSE	FALSE	TRUE	FALSE	FALSE	FALSE	1	0	0	FALSE	FALSE	FALSE
44257427	InChI=1S/C18H12O9/c1-24-18(23)27-17-16-11(7-4-2-3-5-9(7)26-17)14(21)12-10(25-16)6-8(19)13(20)15(12)22/h2-6,17,19-20,22H,1H3	Organic compounds	Lipids and lipid-like molecules	Polyketides	Flavonoids					FALSE	FALSE	FALSE	TRUE	FALSE	FALSE	FALSE	1	0	0	FALSE	FALSE	FALSE
454	InChI=1S/C8H16O/c1-2-3-4-5-6-7-8-9/h8H,2-7H2,1H3	Organic compounds	Organic oxygen compounds	Organooxygen compounds	Carbonyl compounds	Aldehydes	Medium-chain aldehydes			FALSE	FALSE	FALSE	FALSE	TRUE	FALSE	FALSE	0	1	0	FALSE	FALSE	FALSE
4991	InChI=1S/C9H13N2O2/c1-10(2)9(12)13-8-5-4-6-11(3)7-8/h4-7H,1-3H3/q+1	Organic compounds	Organoheterocyclic compounds	Pyridines and derivatives	Methylpyridines	N-methylpyridinium compounds				FALSE	FALSE	FALSE	FALSE	TRUE	FALSE	FALSE	0	1	0	FALSE	FALSE	FALSE
4abut	InChI=1S/C4H9NO2/c5-3-1-2-4(6)7/h1-3,5H2,(H,6,7)	Organic compounds	Organic acids and derivatives	Carboxylic acids and derivatives	Amino acids, peptides, and analogues	Amino acids and derivatives	Gamma amino acids and derivatives			TRUE	TRUE	TRUE	TRUE	FALSE	FALSE	FALSE	1	0	0	FALSE	FALSE	FALSE
4hbz	InChI=1S/C7H6O3/c8-6-3-1-5(2-4-6)7(9)10/h1-4,8H,(H,9,10)/p-1	Organic compounds	Benzenoids	Benzene and substituted derivatives	Benzoic acids and derivatives	Benzoic acids				TRUE	FALSE	TRUE	TRUE	FALSE	FALSE	FALSE	1	0	0	FALSE	FALSE	FALSE
4hphac	InChI=1S/C8H8O3/c9-7-3-1-6(2-4-7)5-8(10)11/h1-4,9H,5H2,(H,10,11)/p-1	Organic compounds	Benzenoids	Phenols	1-hydroxy-2-unsubstituted benzenoids					TRUE	FALSE	TRUE	TRUE	FALSE	FALSE	FALSE	1	0	0	FALSE	FALSE	FALSE
4mop	InChI=1S/C6H10O3/c1-4(2)3-5(7)6(8)9/h4H,3H2,1-2H3,(H,8,9)/p-1	Organic compounds	Organic acids and derivatives	Keto acids and derivatives	Short-chain keto acids and derivatives					TRUE	TRUE	TRUE	FALSE	TRUE	FALSE	FALSE	0	1	0	FALSE	FALSE	FALSE
5280401	InChI=1S/C8H19N6O7P/c9-7(10)13-1-3(15)2(14-8(11)12)6(5(17)4(1)16)21-22(18,19)20/h1-6,15-17H,(H4,9,10,13)(H4,11,12,14)(H2,18,19,20)/t1-,2+,3-,4+,5-,6-/m0/s1	Organic compounds	Organic oxygen compounds	Organooxygen compounds	Alcohols and polyols	Secondary alcohols	Cyclohexanols			FALSE	FALSE	FALSE	FALSE	TRUE	FALSE	FALSE	0	1	0	FALSE	FALSE	FALSE
5281071	InChI=1S/C21H23NO3/c1-22(2)11-5-8-18-17-7-4-3-6-16(17)14-25-20-10-9-15(12-19(18)20)13-21(23)24/h3-4,6-10,12H,5,11,13-14H2,1-2H3,(H,23,24)/b18-8-	Organic compounds	Lipids and lipid-like molecules	Prenol lipids	Sesquiterpenoids					FALSE	FALSE	FALSE	TRUE	FALSE	FALSE	FALSE	1	0	0	FALSE	FALSE	FALSE
5281149	InChI=1S/C17H24O/c1-3-5-6-7-8-9-10-11-12-13-14-15-16-17(18)4-2/h4,10-11,17-18H,2-3,5-9,12H2,1H3/b11-10-/t17-/m1/s1	Organic compounds	Lipids and lipid-like molecules	Fatty Acyls	Fatty alcohols	Long-chain fatty alcohols				FALSE	FALSE	FALSE	FALSE	TRUE	FALSE	FALSE	0	1	0	FALSE	FALSE	FALSE
52997	InChI=1S/C15H16N4O5S/c1-9-8-10(2)17-14(16-9)18-15(21)19-25(22,23)12-7-5-4-6-11(12)13(20)24-3/h4-8H,1-3H3,(H2,16,17,18,19,21)	Organic compounds	Benzenoids	Benzene and substituted derivatives	Benzoic acids and derivatives	Benzoic acid esters				FALSE	FALSE	FALSE	FALSE	TRUE	FALSE	FALSE	0	1	0	FALSE	FALSE	FALSE

5312378	InChI=1S/C12H22O2/c1-2-3-4-5-6-7-8-9-10-11-12(13)14/h7-8H,2-6,9-11H2,1H3,(H,13,14)/b8-7-	Organic compounds	Lipids and lipid-like molecules	Fatty Acyls	Fatty acids and conjugates	Medium-chain fatty acids				FALSE	FALSE	FALSE	TRUE	FALSE	FALSE	FALSE	1	0	0	FALSE	FALSE	FALSE
5322111	InChI=1S/C15H24/c1-11-6-5-7-12(2)13-10-15(3,4)14(13)9-8-11/h6,13-14H,2,5,7-10H2,1,3-4H3/b11-6-	Organic compounds	Lipids and lipid-like molecules	Prenol lipids	Sesquiterpenoids					FALSE	FALSE	FALSE	TRUE	FALSE	FALSE	FALSE	1	0	0	FALSE	FALSE	FALSE
53471990	InChI=1S/C21H32O4/c22-20(23)18-16-14-12-10-8-6-4-2-1-3-5-7-9-11-13-15-17-19-21(24)25/h1-2,5-8,11,13H,3-4,9-10,12,14-19H2,(H,22,23)(H,24,25)/b2-1-,7-5-,8-6-,13-11-	Organic compounds	Lipids and lipid-like molecules	Fatty Acyls	Fatty acids and conjugates	Long-chain fatty acids				FALSE	FALSE	FALSE	TRUE	FALSE	FALSE	FALSE	1	0	0	FALSE	FALSE	FALSE
5429	InChI=1S/C7H8N4O2/c1-10-3-8-5-4(10)6(12)9-7(13)11(5)2/h3H,1-2H3,(H,9,12,13)	Organic compounds	Organoheterocyclic compounds	Imidazopyrimidines	Purines and purine derivatives	Xanthines				FALSE	FALSE	FALSE	TRUE	FALSE	FALSE	FALSE	1	0	0	FALSE	FALSE	FALSE
5460102	InChI=1S/C12H10O2S/c13-8-5-6-10-11(12(8)14)7-3-1-2-4-9(7)15-10/h1-6,8,12-14H/t8-,12-/m0/s1	Organic compounds	Organoheterocyclic compounds	Benzothiophenes	1-benzothiophenes					FALSE	FALSE	FALSE	TRUE	FALSE	FALSE	FALSE	1	0	0	FALSE	FALSE	FALSE
5484352	InChI=1S/C6H12N2O3/c1-3(7)5(9)8-4(2)6(10)11/h3-4H,7H2,1-2H3,(H,8,9)(H,10,11)/t3-,4-/m0/s1	Organic compounds	Organic acids and derivatives	Carboxylic acids and derivatives	Amino acids, peptides, and alogues	Peptides	Dipeptides			FALSE	FALSE	FALSE	TRUE	FALSE	FALSE	FALSE	1	0	0	FALSE	FALSE	FALSE
5810	InChI=1S/C5H9NO3/c7-3-1-4(5(8)9)6-2-3/h3-4,6-7H,1-2H2,(H,8,9)/t3-,4+/m1/s1	Organic compounds	Organic acids and derivatives	Carboxylic acids and derivatives	Amino acids, peptides, and alogues	Amino acids and derivatives	Alpha amino acids and derivatives	Proline and derivatives		FALSE	FALSE	FALSE	TRUE	FALSE	FALSE	FALSE	1	0	0	FALSE	FALSE	FALSE
5htrp	InChI=1S/C11H12N2O3/c12-9(11(15)16)3-6-5-13-10-2-1-7(14)4-8(6)10/h1-2,4-5,9,13-14H,3,12H2,(H,15,16)	Organic compounds	Organoheterocyclic compounds	Indoles and derivatives	Tryptamines and derivatives	Serotonins				TRUE	TRUE	TRUE	TRUE	FALSE	FALSE	FALSE	1	0	0	FALSE	FALSE	FALSE
60953	InChI=1S/C15H22FN3O6/c1-3-4-5-6-24-15(23)18-12-9(16)7-19(14(22)17-12)13-11(21)10(20)8(2)25-13/h7-8,10-11,13,20-21H,3-6H2,1-2H3,(H,17,18,22,23)/t8-,10-,11-,13-/m1/s1	Organic compounds	Nucleosides, nucleotides, and analogues	5'-deoxyribonucleosides						FALSE	FALSE	FALSE	TRUE	FALSE	FALSE	FALSE	1	0	0	FALSE	FALSE	FALSE
6441567	InChI=1S/C7H11NO3/c1-3-5(2)7(11)8-4-6(9)10/h3H,4H2,1-2H3,(H,8,11)(H,9,10)/b5-3+	Organic compounds	Organic acids and derivatives	Carboxylic acids and derivatives	Amino acids, peptides, and alogues	Amino acids and derivatives	Alpha amino acids and derivatives	N-acyl-alpha amino acids and derivatives	N-acyl-alpha amino acids	FALSE	FALSE	FALSE	TRUE	FALSE	FALSE	FALSE	1	0	0	FALSE	FALSE	FALSE
64956	InChI=1S/C4H9NO2/c1-3(2-5)4(6)7/h3H,2,5H2,1H3,(H,6,7)	Organic compounds	Organic acids and derivatives	Carboxylic acids and derivatives	Amino acids, peptides, and alogues	Amino acids and derivatives	Beta amino acids and derivatives			FALSE	FALSE	FALSE	FALSE	TRUE	FALSE	FALSE	0	1	0	FALSE	FALSE	FALSE
65555	InChI=1S/C21H34O4/c1-19-8-5-14(23)11-13(19)3-4-15-16(19)6-9-20(2)17(15)7-10-21(20,25)18(24)12-22/h13-17,22-23,25H,3-12H2,1-2H3/t13-,14-,15-,16+,17+,19+,20+,21+/m1/s1	Organic compounds	Lipids and lipid-like molecules	Steroids and steroid derivatives	Hydroxysteroids	21-hydroxysteroids				FALSE	FALSE	FALSE	FALSE	TRUE	FALSE	FALSE	0	1	0	FALSE	FALSE	FALSE
66886955	InChI=1S/C9H8O5/c10-6-1-3-7(4-2-6)14-9(13)5-8(11)12/h1-4,10H,5H2,(H,11,12)	Organic compounds	Benzenoids	Phenol esters						FALSE	FALSE	FALSE	TRUE	FALSE	FALSE	FALSE	1	0	0	FALSE	FALSE	FALSE
6736	InChI=1S/C9H9N/c1-7-6-10-9-5-3-2-4-8(7)9/h2-6,10H,1H3	Organic compounds	Organoheterocyclic compounds	Indoles and derivatives	Indoles	3-alkylindoles	3-methylindoles			FALSE	FALSE	FALSE	TRUE	FALSE	FALSE	FALSE	1	0	0	FALSE	FALSE	FALSE
6834	InChI=1S/C16H19BrN2/c1-19(2)12-10-15(16-5-3-4-11-18-16)13-6-8-14(17)9-7-13/h3-9,11,15H,10,12H2,1-2H3	Organic compounds	Organoheterocyclic compounds	Pyridines and derivatives	Pheniramines					FALSE	FALSE	FALSE	TRUE	FALSE	FALSE	FALSE	1	0	0	FALSE	FALSE	FALSE
68499	InChI=1S/C8H8N2O3/c11-7(12)5-10-8(13)6-2-1-3-9-4-6/h1-4H,5H2,(H,10,13)(H,11,12)	Organic compounds	Organic acids and derivatives	Carboxylic acids and derivatives	Amino acids, peptides, and alogues	Amino acids and derivatives	Alpha amino acids and derivatives	N-acyl-alpha amino acids and derivatives	N-acyl-alpha amino acids	FALSE	FALSE	FALSE	FALSE	TRUE	FALSE	FALSE	0	1	0	FALSE	FALSE	FALSE
6918732	InChI=1S/C6H13NO3/c1-3(4(2)8)5(7)6(9)10/h3-5,8H,7H2,1-2H3,(H,9,10)/t3	Organic compounds	Organic acids and derivatives	Carboxylic acids and derivatives	Amino acids, peptides, and alogues	Amino acids and	Alpha amino acids	Isoleucine and derivati		FALSE	FALSE	FALSE	TRUE	FALSE	FALSE	FALSE	1	0	0	FALSE	FALSE	FALSE

	-,4-,5-/m0/s1					derivatives		ves													
70346	InChI=1S/C7H8N4O3/c1-10-4-3(8-6(13)9-4)5(12)11(2)7(10)14/h1-2H3,(H2,8,9,13)	Organic compounds	Organoheterocyclic compounds	Imidazopyrimidines	Purines and purine derivatives	Xanthines			FALSE	FALSE	FALSE	FALSE	TRUE	FALSE	FALSE	0	1	0	FALSE	FALSE	FALSE
74839	InChI=1S/C11H13NO3/c1-8(13)12-10(11(14)15)7-9-5-3-2-4-6-9/h2-6,10H,7H2,1H3,(H,12,13)(H,14,15)/t10-/m0/s1	Organic compounds	Organic acids and derivatives	Carboxylic acids and derivatives	Amino acids, peptides, and analogues	Amino acids and derivatives	Alpha amino acids and derivatives	Phenylalanine and derivatives	FALSE	FALSE	FALSE	TRUE	TRUE	FALSE	FALSE	1	1	1	FALSE	FALSE	FALSE
7549	InChI=1S/C5H10N6O2/c12-6-10-2-8-1-9(4-10)5-11(3-8)7-13/h1-5H2	Organic compounds	Organoheterocyclic compounds	Triazines	1,3,5-triazines				FALSE	FALSE	FALSE	FALSE	TRUE	FALSE	FALSE	0	1	0	FALSE	FALSE	FALSE
75619	InChI=1S/C8H11N3O3/c1-5(12)11-7(8(13)14)2-6-3-9-4-10-6/h3-4,7H,2H2,1H3,(H,9,10)(H,11,12)(H,13,14)/t7-/m0/s1	Organic compounds	Organic acids and derivatives	Carboxylic acids and derivatives	Amino acids, peptides, and analogues	Amino acids and derivatives	Alpha amino acids and derivatives	Histidine and derivatives	FALSE	FALSE	FALSE	TRUE	FALSE	FALSE	FALSE	1	0	0	FALSE	FALSE	FALSE
76739	InChI=1S/C23H24O4/c1-15(2)21(24)26-19-11-7-17(8-12-19)23(5,6)18-9-13-20(14-10-18)27-22(25)16(3)4/h7-14H,1,3H2,2,4-6H3	Organic compounds	Benzenoids	Benzene and substituted derivatives	Diphenylmethanes				FALSE	FALSE	FALSE	FALSE	TRUE	FALSE	FALSE	0	1	0	FALSE	FALSE	FALSE
8',10'-Dihydroxydihydroergotamine		Organic compounds	Alkaloids and derivatives						FALSE	FALSE	FALSE	TRUE	FALSE	FALSE	FALSE	1	0	0	FALSE	FALSE	FALSE
80220	InChI=1S/C6H6N4O2/c1-10-5(11)3-4(8-2-7-3)9-6(10)12/h2H,1H3,(H,7,8)(H,9,12)	Organic compounds	Organoheterocyclic compounds	Imidazopyrimidines	Purines and purine derivatives	Xanthines			FALSE	FALSE	FALSE	TRUE	FALSE	FALSE	FALSE	1	0	0	FALSE	FALSE	FALSE
8404	InChI=1S/C10H12/c1-2-6-10-8-4-3-7-9(10)5-1/h1-2,5-6H,3-4,7-8H2	Organic compounds	Benzenoids	Tetralins					FALSE	FALSE	FALSE	TRUE	FALSE	FALSE	FALSE	1	0	0	FALSE	FALSE	FALSE
8897	InChI=1S/C4H7NO4/c6-3(7)1-2-4(8)9/h5H,1-2H2,(H,6,7)(H,8,9)	Organic compounds	Organic acids and derivatives	Carboxylic acids and derivatives	Amino acids, peptides, and analogues	Amino acids and derivatives	Alpha amino acids and derivatives	Alpha amino acids	FALSE	FALSE	FALSE	TRUE	FALSE	FALSE	FALSE	1	0	0	FALSE	FALSE	FALSE
90186425	InChI=1S/C19H32O8S2/c1-18-9-7-13(26-28(20,21)22)11-12(18)3-4-14-15-5-6-17(27-29(23,24)25)19(15,2)10-8-16(14)18/h12-17H,3-11H2,1-2H3,(H,20,21,22)(H,23,24,25)/t12-,13-,14-,15-,16-,17+,18-,19-/m0/s1	Organic compounds	Lipids and lipid-like molecules	Steroids and steroid derivatives	Hydroxysteroids	21-hydroxysteroids			FALSE	FALSE	FALSE	FALSE	TRUE	FALSE	FALSE	0	1	0	FALSE	FALSE	FALSE
91623	InChI=1S/C7H9NO2/c8-4-5-1-2-6(9)7(10)3-5/h1-3,9-10H,4,8H2	Organic compounds	Benzenoids	Phenols	Benzenediols	Catechols			FALSE	FALSE	FALSE	FALSE	TRUE	FALSE	FALSE	0	1	0	FALSE	FALSE	FALSE
92855	InChI=1S/C23H32O3/c1-14(24)19-7-8-20-18-6-5-16-13-17(26-15(2)25)9-11-22(16,3)21(18)10-12-23(19,20)4/h5,7,17-18,20-21H,6,8-13H2,1-4H3/t17-,18-,20-,21-,22-,23+/m0/s1	Organic compounds	Lipids and lipid-like molecules	Steroids and steroid derivatives	Steroid esters				FALSE	FALSE	FALSE	TRUE	FALSE	FALSE	FALSE	1	0	0	FALSE	FALSE	FALSE
93078	InChI=1S/C13H16N2O5/c14-9(7-11(16)17)12(18)15-10(13(19)20)6-8-4-2-1-3-5-8/h1-5,9-10H,6-7,14H2,(H,15,18)(H,16,17)(H,19,20)/t9-,10-/m0/s1	Organic compounds	Organic acids and derivatives	Carboxylic acids and derivatives	Amino acids, peptides, and analogues	Peptides			FALSE	FALSE	FALSE	FALSE	TRUE	FALSE	FALSE	0	1	0	FALSE	FALSE	FALSE
9543299	InChI=1S/C7H7ClO5/c1-3(6(10)11)5(9)2-4(8)7(12)13/h2-3H,1H3,(H,10,11)(H,12,13)/b4-2+	Organic compounds	Organic acids and derivatives	Keto acids and derivatives	Medium-chain keto acids and derivatives				FALSE	FALSE	FALSE	FALSE	TRUE	FALSE	FALSE	0	1	0	FALSE	FALSE	FALSE
APG(34:2)		Organic compounds	Lipids and lipid-like molecules	Glycerophospholipids					FALSE	FALSE	FALSE	FALSE	TRUE	FALSE	FALSE	0	1	0	FALSE	FALSE	FALSE
APG(38:1)		Organic compounds	Lipids and lipid-like molecules	Glycerophospholipids					FALSE	FALSE	FALSE	FALSE	TRUE	FALSE	FALSE	0	1	0	FALSE	FALSE	FALSE
Acetylspermidine		Organic compounds	Organic acids and derivatives	Carboximidic acids and derivatives					FALSE	FALSE	FALSE	TRUE	FALSE	FALSE	FALSE	1	0	0	FALSE	FALSE	FALSE
C01507	InChI=1S/C6H14O6/c7-1-3(9)5(11)6(12)4(10)2-8/h3-12H,1-2H2/t3-,4-,5+,6+/m0/s1	Organic compounds	Organic oxygen compounds	Organooxygen compounds	Carbohydrates and carbohydrate conjugates	Sugar alcohols			TRUE	FALSE	TRUE	TRUE	FALSE	FALSE	FALSE	1	0	0	FALSE	FALSE	FALSE

C02356	InChI=1S/C4H9NO2/c1-2-3(5)4(6)7/h3H,2,5H2,1H3,(H,6,7)/t3-/m0/s1	Organic compounds	Organic acids and derivatives	Carboxylic acids and derivatives	Amino acids, peptides, and alogues	Amino acids and derivatives	Alpha amino acids and derivatives	Alpha amino acids	L-alpha-amino acids	TRUE	TRUE	TRUE	FALSE	TRUE	FALSE	FALSE	0	1	0	FALSE	FALSE	FALSE
C02470	InChI=1S/C10H7NO4/c12-7-3-1-2-5-8(13)4-6(10(14)15)11-9(5)7/h1-4,12H,(H,11,13)(H,14,15)/p-1	Organic compounds	Organoheterocyclic compounds	Quinolines and derivatives	Quinoline carboxylic acids					TRUE	FALSE	TRUE	TRUE	FALSE	FALSE	FALSE	1	0	0	FALSE	FALSE	FALSE
C10164	InChI=1S/C6H5NO2/c8-6(9)5-3-1-2-4-7-5/h1-4H,(H,8,9)/p-1	Organic compounds	Organoheterocyclic compounds	Pyridines and derivatives	Pyridinecarboxylic acids and derivatives	Pyridinecarboxylic acids				TRUE	FALSE	TRUE	FALSE	TRUE	FALSE	FALSE	0	1	0	FALSE	FALSE	FALSE
C10:0-carnitine		Organic compounds	Lipids and lipid-like molecules	Fatty Acyls	Fatty acid esters	Acyl carnitines				FALSE	FALSE	FALSE	FALSE	TRUE	FALSE	FALSE	0	1	0	FALSE	FALSE	FALSE
C10:1-carnitine		Organic compounds	Lipids and lipid-like molecules	Fatty Acyls	Fatty acid esters	Acyl carnitines				FALSE	FALSE	FALSE	FALSE	TRUE	FALSE	FALSE	0	1	0	FALSE	FALSE	FALSE
C12:0-carnitine		Organic compounds	Lipids and lipid-like molecules	Fatty Acyls	Fatty acid esters	Acyl carnitines				FALSE	FALSE	FALSE	FALSE	TRUE	FALSE	FALSE	0	1	0	FALSE	FALSE	FALSE
C16:1-carnitine		Organic compounds	Lipids and lipid-like molecules	Fatty Acyls	Fatty acid esters	Acyl carnitines				FALSE	FALSE	FALSE	FALSE	TRUE	FALSE	FALSE	0	1	0	FALSE	FALSE	FALSE
C18:1-carnitine		Organic compounds	Lipids and lipid-like molecules	Fatty Acyls	Fatty acid esters	Acyl carnitines				FALSE	FALSE	FALSE	FALSE	TRUE	FALSE	FALSE	0	1	0	FALSE	FALSE	FALSE
C8:0-carnitine		Organic compounds	Lipids and lipid-like molecules	Fatty Acyls	Fatty acid esters	Acyl carnitines				FALSE	FALSE	FALSE	FALSE	TRUE	FALSE	FALSE	0	1	0	FALSE	FALSE	FALSE
CE1059	InChI=1S/C11H23N3O2/c1-10(15)13-8-4-3-6-12-7-5-9-14-11(2)16/h12H,3-9H2,1-2H3,(H,13,15)(H,14,16)/p+1	Organic compounds	Organic acids and derivatives	Carboxylic acids and derivatives	Carboxylic acid derivatives	Carboxylic acid amides	Acetamides			TRUE	FALSE	TRUE	TRUE	FALSE	FALSE	FALSE	1	0	0	FALSE	FALSE	FALSE
CE2176	InChI=1S/C10H13NO4/c1-15-9-5-6(2-3-8(9)12)4-7(11)10(13)14/h2-3,5,7,12H,4,11H2,1H3,(H,13,14)	Organic compounds	Organic acids and derivatives	Carboxylic acids and derivatives	Amino acids, peptides, and alogues	Amino acids and derivatives	Alpha amino acids and derivatives	Tyrosine and derivatives		TRUE	FALSE	TRUE	TRUE	FALSE	TRUE	FALSE	2	0	0	FALSE	FALSE	TRUE
Dihydro SM 18:1		Organic compounds	Lipids and lipid-like molecules	Sphingolipids						FALSE	FALSE	FALSE	FALSE	TRUE	FALSE	FALSE	0	1	0	FALSE	FALSE	FALSE
Dihydro SM 20:0		Organic compounds	Lipids and lipid-like molecules	Sphingolipids						FALSE	FALSE	FALSE	FALSE	TRUE	FALSE	TRUE	0	2	0	FALSE	FALSE	TRUE
Dihydro SM 22:0		Organic compounds	Lipids and lipid-like molecules	Sphingolipids						FALSE	FALSE	FALSE	FALSE	TRUE	FALSE	TRUE	0	2	0	FALSE	FALSE	TRUE
Dihydro SM 24: 0		Organic compounds	Lipids and lipid-like molecules	Sphingolipids						FALSE	FALSE	FALSE	FALSE	TRUE	FALSE	FALSE	0	1	0	FALSE	FALSE	FALSE
GB3(16:0)		Organic compounds	Lipids and lipid-like molecules							FALSE	FALSE	FALSE	TRUE	FALSE	FALSE	FALSE	1	0	0	FALSE	FALSE	FALSE
GB3(18:0)		Organic compounds	Lipids and lipid-like molecules							FALSE	FALSE	FALSE	TRUE	FALSE	FALSE	FALSE	1	0	0	FALSE	FALSE	FALSE
GB3(24:1)		Organic compounds	Lipids and lipid-like molecules							FALSE	FALSE	FALSE	FALSE	TRUE	FALSE	FALSE	0	1	0	FALSE	FALSE	FALSE
GlcCer(16:0)		Organic compounds	Lipids and lipid-like molecules							FALSE	FALSE	FALSE	TRUE	FALSE	FALSE	FALSE	1	0	0	FALSE	FALSE	FALSE
GlcCer(22:0)		Organic compounds	Lipids and lipid-like molecules							FALSE	FALSE	FALSE	TRUE	FALSE	FALSE	FALSE	1	0	0	FALSE	FALSE	FALSE
GlcCer(24:1)		Organic compounds	Lipids and lipid-like molecules							FALSE	FALSE	FALSE	TRUE	FALSE	FALSE	FALSE	1	0	0	FALSE	FALSE	FALSE
HC00319	InChI=1S/C3H4O4/c4-2(5)1-3(6)7/h1H2,(H,4,5)(H,6,7)/p-2	Organic compounds	Organic acids and derivatives	Carboxylic acids and derivatives	Dicarboxylic acids and derivatives					TRUE	TRUE	TRUE	TRUE	FALSE	FALSE	FALSE	1	0	0	FALSE	FALSE	FALSE
LPAC14:0		Organic compounds	Lipids and lipid-like molecules	Glycerophospholipids	Glycerophosphates	Monoacylglycerophosphates				FALSE	FALSE	FALSE	TRUE	FALSE	FALSE	FALSE	1	0	0	FALSE	FALSE	FALSE
LPAC16:0	InChI=1S/C19H39O7P/c1-2-3-4-5-6-7-8-9-10-11-12-13-14-15-19(21)25-16-18(20)17-26-27(22,23)24/h18,20H,2-17H2,1H3,(H,22,23,24)/t18-/m1/s1	Organic compounds	Lipids and lipid-like molecules	Glycerophospholipids	Glycerophosphates	Monoacylglycerophosphates	Lysophosphatidic acids	1-acylglycerol-3-phosphates		FALSE	FALSE	FALSE	TRUE	FALSE	FALSE	FALSE	1	0	0	FALSE	FALSE	FALSE
LPAC16:1		Organic compounds	Lipids and lipid-like molecules	Glycerophospholipids	Glycerophosphates	Monoacylglycerophosphates				FALSE	FALSE	FALSE	TRUE	FALSE	FALSE	FALSE	1	0	0	FALSE	FALSE	FALSE

LPAC18:3(w3w6)		Organic compounds	Lipids and lipid-like molecules	Glycerophospholipids	Glycerophosphates	Monoacylglycerophosphates				FALSE	FALSE	FALSE	TRUE	FALSE	FALSE	FALSE	1	0	0	FALSE	FALSE	FALSE
LPAC20:3		Organic compounds	Lipids and lipid-like molecules	Glycerophospholipids	Glycerophosphates	Monoacylglycerophosphates				FALSE	FALSE	FALSE	TRUE	FALSE	FALSE	FALSE	1	0	0	FALSE	FALSE	FALSE
LPI(18:0)		Organic compounds	Lipids and lipid-like molecules	Glycerophospholipids	Glycerophosphoinositols					FALSE	FALSE	FALSE	FALSE	TRUE	FALSE	FALSE	0	1	0	FALSE	FALSE	FALSE
LPI(18:1)		Organic compounds	Lipids and lipid-like molecules	Glycerophospholipids	Glycerophosphoinositols					FALSE	FALSE	FALSE	TRUE	FALSE	FALSE	FALSE	1	0	0	FALSE	FALSE	FALSE
Lcystin	InChI=1S/C6H12N2O4S2/c7-3(5(9)10)1-13-14-2-4(8)6(11)12/h3-4H,1-2,7-8H2,(H,9,10)(H,11,12)/t3-,4-/m0/s1	Organic compounds	Organic acids and derivatives	Carboxylic acids and derivatives	Amino acids, peptides, and alogues	Amino acids and derivatives	Alpha amino acids and derivatives	Cysteine and derivatives	L-cysteine-S-conjugates	TRUE	TRUE	TRUE	TRUE	FALSE	FALSE	FALSE	1	0	0	FALSE	FALSE	FALSE
Lkynr	InChI=1S/C10H12N2O3/c11-7-4-2-1-3-6(7)9(13)5-8(12)10(14)15/h1-4,8H,5,11-12H2,(H,14,15)/t8-/m0/s1	Organic compounds	Organic oxygen compounds	Organooxygen compounds	Carbonyl compounds	Aryl ketones	Phenyl ketones	Alkyl-phenylketones		TRUE	TRUE	TRUE	TRUE	FALSE	FALSE	FALSE	1	0	0	FALSE	FALSE	FALSE
M00021	InChI=1S/C22H40O2/c1-2-3-4-5-6-7-8-9-10-11-12-13-14-15-16-17-18-19-20-21-22(23)24/h6-7,9-10H,2-5,8,11-21H2,1H3,(H,23,24)/b7-6+,10-9+	Organic compounds	Lipids and lipid-like molecules	Fatty Acyls	Fatty acids and conjugates	Very long-chain fatty acids				TRUE	FALSE	TRUE	TRUE	FALSE	FALSE	FALSE	1	0	0	FALSE	FALSE	FALSE
M02723	InChI=1S/C10H11NO3/c12-9(11-7-10(13)14)6-8-4-2-1-3-5-8/h1-5H,6-7H2,(H,11,12)(H,13,14)	Organic compounds	Organic acids and derivatives	Carboxylic acids and derivatives	Amino acids, peptides, and analogues	Amino acids and derivatives	Alpha amino acids and derivatives	N-acyl-alpha amino acids and derivatives	N-acyl-alpha amino acids	TRUE	FALSE	TRUE	TRUE	FALSE	FALSE	FALSE	1	0	0	FALSE	FALSE	FALSE
M03054	InChI=1S/C3H9NO/c1-4(2,3)5/h1-3H3	Organic compounds	Organic nitrogen compounds	Organonitrogen compounds	Aminoxides	Trialkyl amine oxides				TRUE	FALSE	TRUE	TRUE	TRUE	FALSE	FALSE	1	1	1	FALSE	FALSE	FALSE
MG(16:0)		Organic compounds	Lipids and lipid-like molecules	Glycerolipids	Monoradylglycerols					FALSE	FALSE	FALSE	FALSE	TRUE	FALSE	FALSE	0	1	0	FALSE	FALSE	FALSE
PC 38:5		Organic compounds	Lipids and lipid-like molecules	Glycerophospholipids	Glycerophosphocholines					FALSE	FALSE	FALSE	FALSE	TRUE	FALSE	FALSE	0	1	0	FALSE	FALSE	FALSE
PC(22:6)		Organic compounds	Lipids and lipid-like molecules	Glycerophospholipids	Glycerophosphocholines					FALSE	FALSE	FALSE	TRUE	FALSE	FALSE	FALSE	1	0	0	FALSE	FALSE	FALSE
PC(O-16:1)		Organic compounds	Lipids and lipid-like molecules	Glycerophospholipids	Glycerophosphocholines	Phosphatidylcholines				FALSE	FALSE	FALSE	FALSE	TRUE	FALSE	FALSE	0	1	0	FALSE	FALSE	FALSE
PC38:6		Organic compounds	Lipids and lipid-like molecules	Glycerophospholipids	Glycerophosphocholines					FALSE	FALSE	FALSE	FALSE	TRUE	FALSE	FALSE	0	1	0	FALSE	FALSE	FALSE
PE(36:1)		Organic compounds	Lipids and lipid-like molecules	Glycerophospholipids	Glycerophosphoethanolamines					FALSE	FALSE	FALSE	FALSE	TRUE	FALSE	FALSE	0	1	0	FALSE	FALSE	FALSE
PE(40:6)		Organic compounds	Lipids and lipid-like molecules	Glycerophospholipids	Glycerophosphoethanolamines					FALSE	FALSE	FALSE	FALSE	TRUE	FALSE	FALSE	0	1	0	FALSE	FALSE	FALSE
PEp(36:2)		Organic compounds	Lipids and lipid-like molecules	Glycerophospholipids	Glycerophosphoethanolamines					FALSE	FALSE	FALSE	FALSE	TRUE	FALSE	FALSE	0	1	0	FALSE	FALSE	FALSE
PEp(38:5)		Organic compounds	Lipids and lipid-like molecules	Glycerophospholipids	Glycerophosphoethanolamines					FALSE	FALSE	FALSE	FALSE	TRUE	FALSE	FALSE	0	1	0	FALSE	FALSE	FALSE
PEp(38:6)		Organic compounds	Lipids and lipid-like molecules	Glycerophospholipids	Glycerophosphoethanolamines					FALSE	FALSE	FALSE	FALSE	TRUE	FALSE	TRUE	0	2	0	FALSE	FALSE	TRUE
PEp(40:6)		Organic compounds	Lipids and lipid-like molecules	Glycerophospholipids	Glycerophosphoethanolamines					FALSE	FALSE	FALSE	FALSE	TRUE	FALSE	FALSE	0	1	0	FALSE	FALSE	FALSE
PEp(40:7)		Organic compounds	Lipids and lipid-like molecules	Glycerophospholipids	Glycerophosphoethanolamines					FALSE	FALSE	FALSE	FALSE	TRUE	FALSE	FALSE	0	1	0	FALSE	FALSE	FALSE
PEp(42:7)		Organic compounds	Lipids and lipid-like molecules	Glycerophospholipids						FALSE	FALSE	FALSE	TRUE	FALSE	FALSE	FALSE	1	0	0	FALSE	FALSE	FALSE
PG(32:0)		Organic compounds	Lipids and lipid-like molecules	Glycerophospholipids	Glycerophosphoglycerols					FALSE	FALSE	FALSE	FALSE	TRUE	FALSE	FALSE	0	1	0	FALSE	FALSE	FALSE
PS(22:6)		Organic compounds	Lipids and lipid-like molecules	Glycerophospholipids	Glycerophosphoserines	Phosphatidylserines				FALSE	FALSE	FALSE	FALSE	TRUE	FALSE	FALSE	0	1	0	FALSE	FALSE	FALSE
Phenylcarnitine		?								FALSE	FALSE	FALSE	TRUE	FALSE	FALSE	FALSE	1	0	0	FALSE	FALSE	FALSE

ac	InChI=1S/C2H4O2/c1-2(3)4/h1H3,(H,3,4)/p-1	Organic compounds	Organic acids and derivatives	Carboxylic acids and derivatives	Carboxylic acids					TRUE	TRUE	TRUE	FALSE	TRUE	FALSE	FALSE	0	1	0	FALSE	FALSE	FALSE
ala_L	InChI=1S/C3H7NO2/c1-2(4)3(5)6/h2H,4H2,1H3,(H,5,6)/t2-/m0/s1	Organic compounds	Organic acids and derivatives	Carboxylic acids and derivatives	Amino acids, peptides, and alogues	Amino acids and derivatives	Alpha amino acids and derivatives	Alanine and derivatives		TRUE	TRUE	TRUE	TRUE	TRUE	FALSE	FALSE	1	1	1	TRUE	FALSE	FALSE
aprut	InChI=1S/C6H14N2O/c1-6(9)8-5-3-2-4-7/h2-5,7H2,1H3,(H,8,9)/p+1	Organic compounds	Organic acids and derivatives	Carboxylic acids and derivatives	Carboxylic acid derivatives	Carboxylic acid amides	Acetamides			TRUE	TRUE	TRUE	TRUE	FALSE	FALSE	FALSE	1	0	0	FALSE	FALSE	FALSE
arg_L	InChI=1S/C6H14N4O2/c7-4(5(11)12)2-1-3-10-6(8)9/h4H,1-3,7H2,(H,11,12)(H4,8,9,10)/p+1/t4-/m0/s1	Organic compounds	Organic acids and derivatives	Carboxylic acids and derivatives	Amino acids, peptides, and alogues	Amino acids and derivatives	Alpha amino acids and derivatives	Alpha amino acids	L-alpha-amino acids	TRUE	TRUE	TRUE	FALSE	TRUE	FALSE	FALSE	0	1	0	TRUE	FALSE	FALSE
asn_L	InChI=1S/C4H8N2O3/c5-2(4(8)9)1-3(6)7/h2H,1,5H2,(H2,6,7)(H,8,9)/t2-/m0/s1	Organic compounds	Organic acids and derivatives	Carboxylic acids and derivatives	Amino acids, peptides, and alogues	Amino acids and derivatives	Alpha amino acids and derivatives	Asparagine and derivatives		TRUE	TRUE	TRUE	FALSE	TRUE	FALSE	TRUE	0	2	0	TRUE	FALSE	TRUE
asp_D	InChI=1S/C4H7NO4/c5-2(4(8)9)1-3(6)7/h2H,1,5H2,(H,6,7)(H,8,9)/p-1/t2-/m1/s1	Organic compounds	Organic acids and derivatives	Carboxylic acids and derivatives	Amino acids, peptides, and alogues	Amino acids and derivatives	Alpha amino acids and derivatives	Alpha amino acids	Aspartic acid and derivatives	TRUE	FALSE	TRUE	FALSE	TRUE	FALSE	FALSE	0	1	0	FALSE	FALSE	FALSE
bhb	InChI=1S/C4H8O3/c1-3(5)2-4(6)7/h3,5H,2H2,1H3,(H,6,7)/p-1/t3-/m1/s1	Organic compounds	Organic acids and derivatives	Hydroxy acids and derivatives	Beta hydroxy acids and derivatives					TRUE	TRUE	TRUE	TRUE	FALSE	FALSE	FALSE	1	0	0	TRUE	FALSE	FALSE
biliverd	InChI=1S/C33H34N4O6/c1-7-20-19(6)32(42)37-27(20)14-25-18(5)23(10-12-31(40)41)29(35-25)15-28-22(9-11-30(38)39)17(4)24(34-28)13-26-16(3)21(8-2)33(43)36-26/h7-8,13-15,35H,1-2,9-12H2,3-6H3,(H,36,43)(H,37,42)(H,38,39)(H,40,41)/p-2/b26-13-,27-14-,28-15-	Organic compounds	Organoheterocyclic compounds	Tetrapyrroles and derivatives	Bilirubins					TRUE	FALSE	TRUE	FALSE	TRUE	FALSE	FALSE	0	1	0	FALSE	TRUE	FALSE
but	InChI=1S/C4H8O2/c1-2-3-4(5)6/h2-3H2,1H3,(H,5,6)/p-1	Organic compounds	Lipids and lipid-like molecules	Fatty Acyls	Fatty acids and conjugates	Straight chain fatty acids				TRUE	TRUE	TRUE	TRUE	TRUE	FALSE	FALSE	1	1	1	TRUE	FALSE	FALSE
bz	InChI=1S/C7H6O2/c8-7(9)6-4-2-1-3-5-6/h1-5H,(H,8,9)/p-1	Organic compounds	Benzenoids	Benzene and substituted derivatives	Benzoic acids and derivatives	Benzoic acids				TRUE	FALSE	TRUE	TRUE	TRUE	FALSE	FALSE	1	1	1	FALSE	FALSE	FALSE
c8crn	InChI=1S/C15H29NO4/c1-5-6-7-8-9-10-15(19)20-13(11-14(17)18)12-16(2,3)4/h13H,5-12H2,1-4H3/t13-/m1/s1	Organic compounds	Lipids and lipid-like molecules	Fatty Acyls	Fatty acid esters	Acyl carnitines				TRUE	TRUE	TRUE	TRUE	FALSE	FALSE	FALSE	1	0	0	FALSE	FALSE	FALSE
chor	InChI=1S/C10H10O6/c1-5(9(12)13)16-8-4-6(10(14)15)2-3-7(8)11/h2-4,7-8,11H,1H2,(H,12,13)(H,14,15)/t7-,8-/m1/s1	Organic compounds	Organic acids and derivatives	Carboxylic acids and derivatives	Dicarboxylic acids and derivatives					FALSE	FALSE	TRUE	TRUE	FALSE	FALSE	FALSE	1	0	0	FALSE	FALSE	FALSE
cit	InChI=1S/C6H8O7/c7-3(8)1-6(13,5(11)12)2-4(9)10/h13H,1-2H2,(H,7,8)(H,9,10)(H,11,12)/p-3	Organic compounds	Organic acids and derivatives	Carboxylic acids and derivatives	Tricarboxylic acids and derivatives					TRUE	TRUE	TRUE	TRUE	FALSE	FALSE	FALSE	1	0	0	FALSE	FALSE	FALSE
citr_L	InChI=1S/C6H13N3O3/c7-4(5(10)11)2-1-3-9-6(8)12/h4H,1-3,7H2,(H,10,11)(H3,8,9,12)/t4-/m0/s1	Organic compounds	Organic acids and derivatives	Carboxylic acids and derivatives	Amino acids, peptides, and alogues	Amino acids and derivatives	Alpha amino acids and derivatives	Alpha amino acids	L-alpha-amino acids	TRUE	TRUE	TRUE	TRUE	FALSE	FALSE	FALSE	1	0	0	FALSE	FALSE	FALSE
crtsl	InChI=1S/C21H30O5/c1-19-7-5-13(23)9-12(19)3-4-14-15-6-8-21(26,17(25)11-22)20(15,2)10-16(24)18(14)19/h9,14-16,18,22,24,26H,3-8,10-11H2,1-2H3/t14-,15-,16-,18+,19-,20-,21-/m0/s1	Organic compounds	Lipids and lipid-like molecules	Steroids and steroid derivatives	Hydroxysteroids	21-hydroxysteroids				TRUE	FALSE	TRUE	TRUE	FALSE	FALSE	FALSE	1	0	0	TRUE	FALSE	FALSE
cyst_L	InChI=1S/C7H14N2O4S/c8-4(6(10)11)1-2-14-3-5(9)7(12)13/h4-5H,1-3,8-9H2,(H,10,11)(H,12,13)	Organic compounds	Organic acids and derivatives	Carboxylic acids and derivatives	Amino acids, peptides, and alogues	Amino acids and derivatives	Alpha amino acids and derivatives	Cysteine and derivatives	L-cysteine-S-conjugates	TRUE	TRUE	TRUE	TRUE	FALSE	FALSE	FALSE	1	0	0	FALSE	FALSE	FALSE
dhGB3(16:0)		Organic compounds	Lipids and lipid-like molecules							FALSE	FALSE	FALSE	TRUE	FALSE	FALSE	FALSE	1	0	0	FALSE	FALSE	FALSE

dhGM3(16:0)		Organic compounds	Lipids and lipid-like molecules						FALSE	FALSE	FALSE	TRUE	FALSE	FALSE	FALSE	1	0	0	FALSE	FALSE	FALSE
dhGM3(22:0)		Organic compounds	Lipids and lipid-like molecules	Sphingolipids					FALSE	FALSE	FALSE	FALSE	TRUE	FALSE	FALSE	0	1	0	FALSE	FALSE	FALSE
dhGM3(22:1)		Organic compounds	Lipids and lipid-like molecules	Sphingolipids					FALSE	FALSE	FALSE	FALSE	TRUE	FALSE	FALSE	0	1	0	FALSE	FALSE	FALSE
docosdiac	InChI=1S/C22H42O4/c23-21(24)19-17-15-13-11-9-7-5-3-1-2-4-6-8-10-12-14-16-18-20-22(25)26/h1-20H2,(H,23,24)(H,25,26)/p-2	Organic compounds	Lipids and lipid-like molecules	Fatty Acyls	Fatty acids and conjugates	Very long-chain fatty acids			TRUE	FALSE	TRUE	TRUE	FALSE	FALSE	FALSE	1	0	0	FALSE	FALSE	FALSE
glu_L	InChI=1S/C5H9NO4/c6-3(5(9)10)1-2-4(7)8/h3H,1-2,6H2,(H,7,8)(H,9,10)/p-1/t3-/m0/s1	Organic compounds	Organic acids and derivatives	Carboxylic acids and derivatives	Amino acids, peptides, and alogues	Amino acids and derivatives	Alpha amino acids and derivatives	Glutamic acid and derivatives	TRUE	TRUE	TRUE	FALSE	TRUE	FALSE	FALSE	0	1	0	TRUE	FALSE	FALSE
gly	InChI=1S/C2H5NO2/c3-1-2(4)5/h1,3H2,(H,4,5)	Organic compounds	Organic acids and derivatives	Carboxylic acids and derivatives	Amino acids, peptides, and alogues	Amino acids and derivatives	Alpha amino acids and derivatives	Alpha amino acids	TRUE	TRUE	TRUE	TRUE	FALSE	FALSE	FALSE	1	0	0	FALSE	FALSE	FALSE
glyc	InChI=1S/C3H8O3/c4-1-3(6)2-5/h3-6H,1-2H2	Organic compounds	Organic oxygen compounds	Organooxygen compounds	Carbohydrates and carbohydrate conjugates	Sugar alcohols			TRUE	TRUE	TRUE	FALSE	TRUE	FALSE	FALSE	0	1	0	FALSE	FALSE	FALSE
glyc_R	InChI=1S/C3H6O4/c4-1-2(5)3(6)7/h2,4-5H,1H2,(H,6,7)/p-1/t2-/m0/s1	Organic compounds	Organic oxygen compounds	Organooxygen compounds	Carbohydrates and carbohydrate conjugates	Sugar acids and derivatives			TRUE	TRUE	TRUE	TRUE	FALSE	TRUE	FALSE	2	0	0	FALSE	FALSE	TRUE
glyclt	InChI=1S/C2H4O3/c3-1-2(4)5/h3H,1H2,(H,4,5)/p-1	Organic compounds	Organic acids and derivatives	Carboxylic acids and derivatives	Carboxylic acids				TRUE	TRUE	TRUE	TRUE	FALSE	FALSE	FALSE	1	0	0	FALSE	FALSE	FALSE
homoval	InChI=1S/C9H10O4/c1-13-8-4-6(5-9(11)12)2-3-7(8)10/h2-4,10H,5H2,1H3,(H,11,12)/p-1	Organic compounds	Benzenoids	Phenols	Methoxyphenols				TRUE	FALSE	TRUE	TRUE	FALSE	FALSE	FALSE	1	0	0	FALSE	FALSE	FALSE
icit	InChI=1S/C6H8O7/c7-3(8)1-2(5(10)11)4(9)6(12)13/h2,4,9H,1H2,(H,7,8)(H,10,11)(H,12,13)/p-3	Organic compounds	Organic acids and derivatives	Carboxylic acids and derivatives	Tricarboxylic acids and derivatives				TRUE	TRUE	TRUE	FALSE	TRUE	FALSE	FALSE	0	1	0	FALSE	FALSE	FALSE
ile_L	InChI=1S/C6H13NO2/c1-3-4(2)5(7)6(8)9/h4-5H,3,7H2,1-2H3,(H,8,9)/t4-,5-/m0/s1	Organic compounds	Organic acids and derivatives	Carboxylic acids and derivatives	Amino acids, peptides, and alogues	Amino acids and derivatives	Alpha amino acids and derivatives	Isoleucine and derivatives	TRUE	TRUE	TRUE	TRUE	TRUE	FALSE	FALSE	1	1	1	TRUE	FALSE	FALSE
ind3ac	InChI=1S/C10H9NO2/c12-10(13)5-7-6-11-9-4-2-1-3-8(7)9/h1-4,6,11H,5H2,(H,12,13)/p-1	Organic compounds	Organoheterocyclic compounds	Indoles and derivatives	Indolyl carboxylic acids and derivatives	Indole-3-acetic acid derivatives			TRUE	TRUE	TRUE	FALSE	TRUE	FALSE	FALSE	0	1	0	FALSE	FALSE	FALSE
ins	InChI=1S/C10H12N4O5/c15-1-4-6(16)7(17)10(19-4)14-3-13-5-8(14)11-2-12-9(5)18/h2-4,6-7,10,15-17H,1H2,(H,11,12,18)/t4-,6-,7-,10-/m1/s1	Organic compounds	Nucleosides, nucleotides, and analogues	Purine nucleosides					TRUE	TRUE	TRUE	FALSE	TRUE	FALSE	FALSE	0	1	0	FALSE	FALSE	FALSE
isobut	InChI=1S/C4H8O2/c1-3(2)4(5)6/h3H,1-2H3,(H,5,6)	Organic compounds	Organic acids and derivatives	Carboxylic acids and derivatives	Carboxylic acids				FALSE	FALSE	TRUE	FALSE	TRUE	FALSE	FALSE	0	1	0	FALSE	FALSE	FALSE
kynate	InChI=1S/C10H7NO3/c12-9-5-8(10(13)14)11-7-4-2-1-3-6(7)9/h1-5H,(H,11,12)(H,13,14)/p-1	Organic compounds	Organoheterocyclic compounds	Quinolines and derivatives	Quinoline carboxylic acids				TRUE	TRUE	TRUE	FALSE	TRUE	FALSE	FALSE	0	1	0	FALSE	FALSE	FALSE
lac_L	InChI=1S/C3H6O3/c1-2(4)3(5)6/h2,4H,1H3,(H,5,6)/p-1/t2-/m0/s1	Organic compounds	Organic oxygen compounds	Organooxygen compounds	Alcohols and polyols	Secondary alcohols			TRUE	TRUE	TRUE	FALSE	TRUE	FALSE	FALSE	0	1	0	TRUE	FALSE	FALSE
leu_L	InChI=1S/C6H13NO2/c1-4(2)3-5(7)6(8)9/h4-5H,3,7H2,1-2H3,(H,8,9)/t5-/m0/s1	Organic compounds	Organic acids and derivatives	Carboxylic acids and derivatives	Amino acids, peptides, and alogues	Amino acids and derivatives	Alpha amino acids and derivatives	Leucine and derivatives	TRUE	TRUE	TRUE	TRUE	TRUE	FALSE	FALSE	1	1	1	TRUE	FALSE	FALSE
man	InChI=1S/C6H12O6/c7-1-2-3(8)4(9)5(10)6(11)12-2/h2-11H,1H2/t2-,3-,4+,5+,6?/m1/s1	Organic compounds	Organic oxygen compounds	Organooxygen compounds	Carbohydrates and carbohydrate conjugates	Monosaccharides	Hexoses		TRUE	FALSE	TRUE	TRUE	FALSE	FALSE	FALSE	1	0	0	FALSE	FALSE	FALSE
met_L	InChI=1S/C5H11NO2S/c1-9-3-2-4(6)5(7)8/h4H,2-3,6H2,1H3,(H,7,8)/t4-/m0/s1	Organic compounds	Organic acids and derivatives	Carboxylic acids and derivatives	Amino acids, peptides, and alogues	Amino acids and derivatives	Alpha amino acids and derivat	Methionine and derivatives	TRUE	TRUE	TRUE	FALSE	TRUE	FALSE	FALSE	0	1	0	TRUE	FALSE	FALSE

							ives															
n8aspmd	InChI=1S/C9H21N3O/c1-9(13)12-8-3-2-6-11-7-4-5-10/h11H,2-8,10H2,1H3,(H,12,13)/p+2	Organic compounds	Organic acids and derivatives	Carboxylic acids and derivatives	Carboxylic acid derivatives	Carboxylic acid amides	Acetamides			TRUE	FALSE	TRUE	TRUE	FALSE	TRUE	FALSE	2	0	0	FALSE	FALSE	TRUE
nac	InChI=1S/C6H5NO2/c8-6(9)5-2-1-3-7-4-5/h1-4H,(H,8,9)/p-1	Organic compounds	Organoheterocyclic compounds	Pyridines and derivatives	Pyridinecarboxylic acids and derivatives	Pyridinecarboxylic acids				TRUE	TRUE	TRUE	TRUE	FALSE	FALSE	FALSE	1	0	0	FALSE	FALSE	FALSE
nrpphr	InChI=1S/C8H11NO3/c9-4-8(12)5-1-2-6(10)7(11)3-5/h1-3,8,10-12H,4,9H2/p+1/t8-/m0/s1	Organic compounds	Benzenoids	Phenols	Benzenediols	Catechols				TRUE	TRUE	TRUE	FALSE	TRUE	FALSE	FALSE	0	1	0	FALSE	FALSE	FALSE
ocdcea	InChI=1S/C18H34O2/c1-2-3-4-5-6-7-8-9-10-11-12-13-14-15-16-17-18(19)20/h9-10H,2-8,11-17H2,1H3,(H,19,20)/p-1/b10-9-	Organic compounds	Lipids and lipid-like molecules	Fatty Acyls	Fatty acids and conjugates	Long-chain fatty acids				TRUE	TRUE	TRUE	TRUE	FALSE	FALSE	FALSE	1	0	0	FALSE	FALSE	FALSE
octa	InChI=1S/C8H16O2/c1-2-3-4-5-6-7-8(9)10/h2-7H2,1H3,(H,9,10)/p-1	Organic compounds	Lipids and lipid-like molecules	Fatty Acyls	Fatty acids and conjugates	Medium-chain fatty acids				TRUE	TRUE	TRUE	FALSE	TRUE	FALSE	FALSE	0	1	0	TRUE	FALSE	FALSE
orn	InChI=1S/C5H12N2O2/c6-3-1-2-4(7)5(8)9/h4H,1-3,6-7H2,(H,8,9)/p+1/t4-/m0/s1	Organic compounds	Organic acids and derivatives	Carboxylic acids and derivatives	Amino acids, peptides, and alogues	Amino acids and derivatives	Alpha amino acids and derivatives	Alpha amino acids	L-alpha-amino acids	TRUE	TRUE	TRUE	TRUE	FALSE	TRUE	FALSE	2	0	0	TRUE	FALSE	TRUE
pcollg5hlys	InChI=1S/C6H14N2O3/c7-3-4(9)1-2-5(8)6(10)11/h4-5,9H,1-3,7-8H2,(H,10,11)/t4-,5+/m0/s1	Organic compounds	Organic acids and derivatives	Carboxylic acids and derivatives	Amino acids, peptides, and alogues	Amino acids and derivatives	Alpha amino acids and derivatives			TRUE	FALSE	TRUE	TRUE	FALSE	FALSE	FALSE	1	0	0	FALSE	FALSE	FALSE
pcs	InChI=1S/C7H8O4S/c1-6-2-4-7(5-3-6)11-12(8,9)10/h2-5H,1H3,(H,8,9,10)/p-1	Organic compounds	Organic acids and derivatives	Organic sulfuric acids and derivatives	Arylsulfates	Phenylsulfates				TRUE	FALSE	TRUE	TRUE	FALSE	FALSE	FALSE	1	0	0	FALSE	FALSE	FALSE
phe_L	InChI=1S/C9H11NO2/c10-8(9(11)12)6-7-4-2-1-3-5-7/h1-5,8H,6,10H2,(H,11,12)/t8-/m0/s1	Organic compounds	Organic acids and derivatives	Carboxylic acids and derivatives	Amino acids, peptides, and alogues	Amino acids and derivatives	Alpha amino acids and derivatives	Phenylalanine and derivatives		TRUE	TRUE	TRUE	TRUE	TRUE	TRUE	TRUE	2	3	1	TRUE	FALSE	TRUE
pheacgln	InChI=1S/C13H16N2O4/c14-11(16)7-6-10(13(18)19)15-12(17)8-9-4-2-1-3-5-9/h1-5,10H,6-8H2,(H2,14,16)(H,15,17)(H,18,19)/t10-/m0/s1	Organic compounds	Organic acids and derivatives	Carboxylic acids and derivatives	Amino acids, peptides, and alogues	Amino acids and derivatives	Alpha amino acids and derivatives	N-acyl-alpha amino acids and derivatives	N-acyl-alpha amino acids	TRUE	FALSE	TRUE	TRUE	FALSE	FALSE	FALSE	1	0	0	FALSE	FALSE	FALSE
phppa	InChI=1S/C9H10O3/c10-8-4-1-7(2-5-8)3-6-9(11)12/h1-2,4-5,10H,3,6H2,(H,11,12)	Organic compounds	Phenylpropanoids and polyketides	Phenylpropanoic acids						FALSE	FALSE	TRUE	FALSE	TRUE	FALSE	FALSE	0	1	0	FALSE	FALSE	FALSE
ppa	InChI=1S/C3H6O2/c1-2-3(4)5/h2H2,1H3,(H,4,5)/p-1	Organic compounds	Organic acids and derivatives	Carboxylic acids and derivatives	Carboxylic acids					TRUE	TRUE	TRUE	TRUE	FALSE	TRUE	FALSE	2	0	0	FALSE	FALSE	TRUE
ptrc	InChI=1S/C4H12N2/c5-3-1-2-4-6/h1-6H2/p+2	Organic compounds	Organic nitrogen compounds	Organonitrogen compounds	Amines	Primary amines	Monoalkylamines			TRUE	TRUE	TRUE	TRUE	FALSE	FALSE	FALSE	1	0	0	FALSE	FALSE	FALSE
quln	InChI=1S/C7H5NO4/c9-6(10)4-2-1-3-8-5(4)7(11)12/h1-3H,(H,9,10)(H,11,12)/p-2	Organic compounds	Organoheterocyclic compounds	Pyridines and derivatives	Pyridinecarboxylic acids and derivatives	Pyridinecarboxylic acids				TRUE	FALSE	TRUE	TRUE	FALSE	FALSE	FALSE	1	0	0	FALSE	FALSE	FALSE
sarcs	InChI=1S/C3H7NO2/c1-4-2-3(5)6/h4H,2H2,1H3,(H,5,6)	Organic compounds	Organic acids and derivatives	Carboxylic acids and derivatives	Amino acids, peptides, and alogues	Amino acids and derivatives	Alpha amino acids and derivatives	Alpha amino acids		TRUE	FALSE	TRUE	TRUE	FALSE	FALSE	FALSE	1	0	0	FALSE	FALSE	FALSE
sebacid	InChI=1S/C10H18O4/c11-9(12)7-5-3-1-2-4-6-8-10(13)14/h1-8H2,(H,11,12)(H,13,14)/p-2	Organic compounds	Lipids and lipid-like molecules	Fatty Acyls	Fatty acids and conjugates	Medium-chain fatty acids				TRUE	FALSE	TRUE	TRUE	FALSE	TRUE	FALSE	2	0	0	FALSE	FALSE	TRUE
ser_L	InChI=1S/C3H7NO3/c4-2(1-5)3(6)7/h2,5H,1,4H2,(H,6,7)/t2-/m0/s1	Organic compounds	Organic acids and derivatives	Carboxylic acids and derivatives	Amino acids, peptides, and alogues	Amino acids and derivatives	Alpha amino acids and derivatives	Serine and derivatives		TRUE	TRUE	TRUE	FALSE	TRUE	FALSE	TRUE	0	3	0	FALSE	FALSE	TRUE
succ	InChI=1S/C4H6O4/c5-3(6)1-2-4(7)8/h1-2H2,(H,5,6)(H,7,8)/p-2	Organic compounds	Organic acids and derivatives	Carboxylic acids and derivatives	Dicarboxylic acids and derivatives					TRUE	TRUE	TRUE	TRUE	FALSE	TRUE	FALSE	2	0	0	FALSE	FALSE	TRUE
taur	InChI=1S/C2H7NO3S/c3-1-2-7(4,5)6/h1-3H2,(H,4,5,6)	Organic compounds	Organic acids and derivatives	Organic sulfonic acids and	Organosulfonic acids and derivatives	Organosulfonic acids				TRUE	TRUE	TRUE	TRUE	TRUE	FALSE	FALSE	1	1	1	FALSE	FALSE	FALSE

				derivatives																		
thr_L	InChI=1S/C4H9NO3/c1-2(6)3(5)4(7)8/h2-3,6H,5H2,1H3,(H,7,8)/t2-,3+/m1/s1	Organic compounds	Organic acids and derivatives	Carboxylic acids and derivatives	Amino acids, peptides, and alogues	Amino acids and derivativ es	Alpha amino acids and derivat ives	Alpha amino acids	L-alpha-amino acids	TRUE	TRUE	TRUE	TRUE	FALSE	TRUE	FALSE	2	0	0	FALSE	FALSE	TRUE
thrnt	InChI=1S/C4H8O5/c5-1-2(6)3(7)4(8)9/h2-3,5-7H,1H2,(H,8,9)/p-1/t2-,3+/m0/s1	Organic compounds	Organic oxygen compounds	Organooxy gen compound s	Carbohydrate s and carbohydrate conjugates	Sugar acids and derivativ es				TRUE	FALSE	TRUE	TRUE	FALSE	FALSE	FALSE	1	0	0	FALSE	FALSE	FALSE
tmlys	InChI=1S/C9H20N2O2/c1-11(2,3)7-5-4-6-8(10)9(12)13/h8H,4-7,10H2,1-3H3/t8-/m0/s1	Organic compounds	Organic acids and derivatives	Carboxylic acids and derivatives	Amino acids, peptides, and alogues	Amino acids and derivativ es	Alpha amino acids and derivat ives	Alpha amino acids	L-alpha-amino acids	TRUE	FALSE	TRUE	TRUE	FALSE	FALSE	FALSE	1	0	0	FALSE	FALSE	FALSE
trypta	InChI=1S/C10H12N2/c11-6-5-8-7-12-10-4-2-1-3-9(8)10/h1-4,7,12H,5-6,11H2/p+1	Organic compounds	Organohetero cyclic compounds	Indoles and derivatives	Tryptamines and derivatives					TRUE	TRUE	TRUE	TRUE	FALSE	FALSE	FALSE	1	0	0	FALSE	FALSE	FALSE
tym	InChI=1S/C8H11NO/c9-6-5-7-1-3-8(10)4-2-7/h1-4,10H,5-6,9H2/p+1	Organic compounds	Benzenoids	Benzene and substituted derivatives	Phenethylami nes					TRUE	TRUE	TRUE	TRUE	FALSE	FALSE	FALSE	1	0	0	FALSE	FALSE	FALSE
tyr_L	InChI=1S/C9H11NO3/c10-8(9(12)13)5-6-1-3-7(11)4-2-6/h1-4,8,11H,5,10H2,(H,12,13)/t8-/m0/s1	Organic compounds	Organic acids and derivatives	Carboxylic acids and derivatives	Amino acids, peptides, and alogues	Amino acids and derivativ es	Alpha amino acids and derivat ives	Tyrosine and derivati ves		TRUE	TRUE	TRUE	TRUE	TRUE	FALSE	FALSE	1	1	1	TRUE	FALSE	FALSE
urea	InChI=1S/CH4N2O/c2-1(3)4/h(H4,2,3,4)	Organic compounds	Organic acids and derivatives	Organic carbonic acids and derivatives	Ureas					TRUE	FALSE	TRUE	FALSE	TRUE	FALSE	FALSE	0	1	0	FALSE	FALSE	FALSE
uri	InChI=1S/C9H12N2O6/c12-3-4-6(14)7(15)8(17-4)11-2-1-5(13)10-9(11)16/h1-2,4,6-8,12,14-15H,3H2,(H,10,13,16)/t4-,6-,7-,8-/m1/s1	Organic compounds	Nucleosides, nucleotides, and alogues	Pyrimidine nucleoside s						TRUE	TRUE	TRUE	TRUE	FALSE	FALSE	FALSE	1	0	0	FALSE	FALSE	FALSE
val_L	InChI=1S/C5H11NO2/c1-3(2)4(6)5(7)8/h3-4H,6H2,1-2H3,(H,7,8)/t4-/m0/s1	Organic compounds	Organic acids and derivatives	Carboxylic acids and derivatives	Amino acids, peptides, and alogues	Amino acids and derivativ es	Alpha amino acids and derivat ives	Valine and derivati ves		TRUE	TRUE	TRUE	FALSE	TRUE	FALSE	FALSE	0	1	0	TRUE	FALSE	FALSE

Supplementary Table 5: Blood brain barrier metabolites.

VMHID	Metabolite name	Metabolites crossing BBB	Matched with diagnosis-related metabolites	Matched with progression-related metabolites
glu_L	L-Glutamic acid	crossing	1	1
crn	L-Carnitine	crossing	1	0
lac_L	L-Lactic acid	crossing	1	1
lys_L	L-Lysine	crossing	1	0
arg_L	L-Arginine	crossing	1	1
orn	Ornithine	crossing	1	1
his_L	L-Histidine	crossing	1	0
gln_L	L-Glutamine	crossing	1	0
met_L	L-Methionine	crossing	1	1
leu_L	L-Leucine	crossing	1	1
ile_L	L-Isoleucine	crossing	1	1
val_L	L-Valine	crossing	1	1
phe_L	L-Phenylalanine	crossing	1	1
trp_L	L-Tryptophan	crossing	1	0
cys_L	L-Cysteine	crossing	1	0
asn_L	L-Asparagine	crossing	1	1
ala_L	L-Alanine	crossing	1	1
glc_D	D-Glucose	crossing	1	0
gal	D-Galactose	crossing	0	0
dhdascb	Dehydroascorbic acid	crossing	1	0
k	Potassium	crossing	0	0
chol	Choline	crossing	1	0
ade	Adenine	crossing	1	0
adn	Adenosine	crossing	0	0
triodthy	Liothyronine	crossing	0	0
bhb	(R)-3-Hydroxybutyric acid	crossing	0	1
thyox_L	Thyroxine	crossing	0	0
tststerone	Testosterone	crossing	1	0
estradiol	Estradiol	crossing	0	0
crtsl	Cortisol	crossing	1	1
aldstrn	Aldosterone	crossing	1	0

crtstrn	Corticosterone	crossing	1	0
cortsn	Cortisone	crossing	1	0
prgstrn	Progesterone	crossing	0	0
melatn	Melatonin	crossing	0	0
hdca	Palmitic acid	crossing	1	0
lnlc	Linoleic acid	crossing	1	0
octa	Caprylic acid	crossing	1	1
but	Butyric acid	crossing	1	1
pnto_R	Pantothenic acid	crossing	1	0
ttdca	Myristic acid	crossing	1	0
tyr_L	L-Tyrosine	crossing	1	1
asp_L	L-Aspartic acid	crossing	1	0
pchol_hs	Phosphatidylcholine	not crossing	0	0
pe_hs	Phosphatidylethanolamine	not crossing	0	0
pail_hs	1-Phosphatidyl-1D-Myo-Inositol	not crossing	0	0
tag_hs	Triglyceride	not crossing	0	0
lpchol_hs	1-Acyl-Sn-Glycero-3-Phosphocholine	not crossing	0	0
alpa_hs	Lysophosphatidic Acid	not crossing	0	0
chsterol	Cholesterol	not crossing	0	0
HC00001	Albumin	not crossing	0	0
HC01852	Fibrinogen	not crossing	0	0
HC01943	Prothrombin	not crossing	0	0
itp	Inosine triphosphate	not crossing	0	0
atp	Adenosine triphosphate	not crossing	0	0
dgtp	dGTP	not crossing	0	0
gtp	Guanosine triphosphate	not crossing	0	0
ak2lgchol_hs	LysoPC(O-18:0)	not crossing	0	0
paf_hs	PC(18:1(9Z)e/2:0)	not crossing	0	0
bglc	Beta-Glucans	not crossing	0	0
glgchlo	Beta Glucan-Glycocholate Complex	not crossing	0	0
gltdechol	Beta Glucan-Taurodeoxycholic Acid Complex	not crossing	0	0
gltcho	Beta Glucan-Taurocholic Acid Complex	not crossing	0	0
thmtp	Thiamine triphosphate	not crossing	0	0

glygn5	Glycogen, Structure 5 (Glycogenin-2[1,4-Glc])	not crossing	0	0
core8	Core 8	not crossing	0	0
triodthysuf	Triiodothyronine Sulfate	not crossing	0	0
HC02187	Reverse-triiodthyronine	not crossing	0	0
udpglcur	Uridine diphosphate glucuronic acid	not crossing	0	0
adprbp	ADP-ribose 2'-phosphate	not crossing	0	0
adprib	Adenosine diphosphate ribose	not crossing	0	0
udpg	Uridine diphosphate glucose	not crossing	0	0
udpgal	Uridine diphosphategalactose	not crossing	0	0
core5	Core 5	not crossing	0	0
core7	Core 7	not crossing	0	0
glygn4	Glycogen, Structure 4 (Glycogenin-1,6-{2[1,4-Glc], [1,4-Glc]})	not crossing	0	0
strch2	Starch, Structure 2 (1,6-{2[1,4-Glc], [1,4-Glc]})	not crossing	0	0
malttr	Maltotriose	not crossing	1	0
crmp_hs	Ceramide 1-Phosphate	not crossing	0	0
crm_hs	N-Acylsphingosine	not crossing	0	0
sTn_antigen	Sialyl-Tn Antigen	not crossing	0	0
11_cis_retfa	Fatty Acid 11-Cis-Retinol	not crossing	0	0
9_cis_retfa	Fatty Acid 9-Cis-Retinol	not crossing	0	0
retfa	Fatty Acid Retinol	not crossing	0	0
gthox	Oxidized glutathione	not crossing	1	0
nadp	NADP	not crossing	0	0
nad	NAD	not crossing	0	0
coa	Coenzyme A	not crossing	1	0
dpcoa	Dephospho-CoA	not crossing	0	0
ksi_deg1	Keratan Sulfate I, Degradation Product 1	not crossing	0	0
ksi	Keratan	not crossing	0	0
malcoa	Malonyl-CoA	not crossing	0	0
core4	Core 4	not crossing	0	0
chtn	Chitin	not crossing	0	0
maltttr	Maltotetraose	not crossing	0	0
galside_hs	Galactosylceramide (d18:1/16:0)	not crossing	0	0
pect	Pectin	not crossing	0	0

pectindchac	Pectin-Deoxycholic Acid Complex	not crossing	0	0
pectingchol	Pectin-Glycocholate Complex	not crossing	0	0
pectintchol	Pectin-Taurocholic Acid Complex	not crossing	0	0
CE5798	Neuromedin N (1-4)	not crossing	0	0
HC02198	Taurolithocholic acid 3-sulfate	not crossing	0	0
tchola	Taurocholic acid	not crossing	1	0
gum	Gums	not crossing	0	0
gumdchac	Guar Gum-Deoxyxholic Acid Complex	not crossing	0	0
gumgchol	Guar Gum-Glycocholate Complex	not crossing	0	0
gumtchol	Guar Gum-Taurocholic Acid Complex	not crossing	0	0
fad	FAD	not crossing	0	0
xolest2_hs	Cholesterol Ester	not crossing	0	0
ha	Hyaluronic acid	not crossing	0	0
leuktrF4	Leukotriene F4	not crossing	0	0
CE4723	Neocasomorphin (1-5)	not crossing	0	0
leuktrC4	Leukotriene C4	not crossing	0	0
maltpt	Maltopentaose	not crossing	0	0
digalsgalside_hs	Digalactosylceramidesulfate	not crossing	0	0
CE5797	Neuromedin N	not crossing	0	0
bilirub	Bilirubin	not crossing	1	0
pheme	Heme	not crossing	1	0
CE5791	Kinetensin 4-8	not crossing	0	0
CE2916	Neocasomorphin	not crossing	0	0
dsT_antigen	Disialyl-T Antigen	not crossing	0	0
malthx	Maltohexaose	not crossing	0	0
CE4722	Beta-Casomorphin (1-6)	not crossing	0	0
5dhf	Pentaglutamyl Folate (Dhf)	not crossing	0	0
bilglcur	Mono(Glucosyluronic Acid)Bilirubin	not crossing	0	0
5thf	Pentaglutamyl Folate (Thf)	not crossing	0	0
dag_hs	Diglyceride	not crossing	0	0
mag_hs	Monoacylglycerol 2	not crossing	0	0
HC01609	Uroporphyrinogen I	not crossing	0	0
10fthf5glu	10-Formyltetrahydrofolate-[Glu](5)	not crossing	0	0

caro	B-Carotene	not crossing	0	0
CE5788	Kinetensin 1-7	not crossing	0	0
arachcoa	Eicosanoyl-CoA	not crossing	0	0
malthp	Maltoheptaose	not crossing	0	0
CE2250	3-Oxodocosanoyl Coenzyme A	not crossing	0	0
6dhf	Hexaglutamyl Folate (Dhf)	not crossing	0	0
6thf	Hexaglutamyl Folate (Thf)	not crossing	0	0
CE2915	Beta-Casomorphin	not crossing	0	0
gbside_hs	Tetrahexosylceramide (d18:1/12:0)	not crossing	0	0
bildglcur	Bilirubin diglucuronide	not crossing	0	0
10fthf6glu	10-Formyltetrahydrofolate-[Glu](6)	not crossing	0	0
cspg_e	Chondroitin Sulfate E (GalNac4,6Dis-Glca) Proteoglycan	not crossing	0	0
cspg_d	Chondroitin Sulfate D (GlcNac6S-Glca2S) Proteoglycan	not crossing	0	0
cspg_b	Chondroitin Sulfate B / Dermatan Sulfate (Idoa2S-GalNac4S) Proteoglycan	not crossing	0	0
cspg_a	Chondroitin Sulfate A (GalNac4S-Glca) Proteoglycan	not crossing	0	0
cspg_c	Chondroitin Sulfate C (GalNac6S-Glca) Proteoglycan	not crossing	0	0
taxol	Paclitaxel	not crossing	0	0
htaxol	6 Alpha Hydroxy Taxol/ 6 Alpha Hydroxy Paclitaxel	not crossing	0	0
7dhf	Heptaglutamyl folic acid	not crossing	0	0
7thf	Heptaglutamyl Folate (Thf)	not crossing	0	0
CE5789	Kinetensin 1-8	not crossing	0	0
fuc14galacglcgalgluside_hs	Lea Glycolipid	not crossing	0	0
fucgal14acglcgalgluside_hs	Lacto-N-Fucopentaosyl Iii Ceramide	not crossing	0	0
oagd3_hs	9-O-Acetylated Gd3	not crossing	0	0
CE2838	Maltononaose	not crossing	0	0
galgalgalthcrm_hs	Gal-Gal-Gal-Gal-Gal-Glc-Cer	not crossing	0	0
CE5786	Kinetensin	not crossing	0	0
fucfuc12gal14acglcgalgluside_hs	Ley Glycolipid	not crossing	0	0
fucfucgalacglcgalgluside_hs	Leb Glycolipid	not crossing	0	0
fucgalgbside_hs	Fucosyl Galactosylgloboside	not crossing	0	0
galfuc12gal14acglcgalgluside_hs	(Gal)3 (Glc)1 (GlcNac)1 (LFuc)1 (Cer)1	not crossing	0	0
n2m2nmasn	N-Acetyl-Beta-D-Glucosaminyl-1,2-Alpha-D-Mannosyl-1,3-(N-Acetyl-Beta-D-Glucosaminyl-1,2-Alpha-D-Mannosyl-1,6)-(N-Acetyl-Beta-D-Glucos aminyl-1,4)-Beta-D-Mannosyl-1,4-N-Acetyl-Beta-D-Glucosaminyl-R	not crossing	0	0
q10	Ubiquinone-1	not crossing	1	0

q10h2	QH2	not crossing	0	0
CE2839	Maltodecaose	not crossing	0	0
CE4724	Apelin (1-12)	not crossing	0	0
fucacngal14acglcgalgluside_hs	Iv3-A-Neuac,Iii3-A-Fuc-Nlc4Cer	not crossing	0	0
fucacngalacglcgalgluside_hs	Iv3-A-Neu5Ac,Iii4-A-Fuc-Lc4Cer	not crossing	0	0
fucgalfucgalacglcgalgluside_hs	(Gal)3 (Glc)1 (GlcNac)1 (LFuc)2 (Cer)1	not crossing	0	0
fucacgalfucgalacglcgalgluside_hs	(Gal)2 (GalNac)1 (Glc)1 (GlcNac)1 (LFuc)2 (Cer)1	not crossing	0	0
fuc13galacglcgal14acglcgalgluside_hs	Iii3Fuc-Nlc6Cer	not crossing	0	0
galacglcgalgbside_hs	Gal-GlcNac-Gal Globoside	not crossing	0	0
oagt3_hs	9-O-Acetylated Gt3	not crossing	0	0
acnacngal14acglcgalgluside_hs	3',8'-LD1	not crossing	0	0
gd1b2_hs	Gd1Beta	not crossing	0	0
gd1c_hs	Gd1C	not crossing	0	0
glygn2	Glycogen, Structure 2 (Glycogenin-1,6-{7[1,4-Glc], 4[1,4-Glc]})	not crossing	0	0
strch1	Starch, Structure 1 (1,6-{7[1,4-Glc], 4[1,4-Glc]})	not crossing	0	0
CE2917	Apelin-13	not crossing	0	0
acngalacglcgal14acglcgalgluside_hs	Vi3Neuac-Nlc6Cer	not crossing	0	0
ps_hs	Phosphatidylserine	not crossing	0	0
pglyc_hs	Phosphatidylglycerol	not crossing	0	0
1glyc_hs	1 Acyl Phosphoglycerol	not crossing	0	0
fucfuc132galacglcgal14acglcgalgluside_hs	V3Fuc,Iii3Fuc-Nlc6Cer	not crossing	0	0
galfucgalacglcgal14acglcgalgluside_hs	(Gal)4 (Glc)1 (GlcNac)2 (LFuc)1 (Cer)1	not crossing	0	0
adocbl	Adenosylcobalamin	not crossing	0	0
acn13acngalgbside_hs	Sialyl-(1,3)-Sialyl-(2,6)-Galactosylgloboside	not crossing	0	0
acn23acngalgbside_hs	Salyl-(2,3)-Sialyl-(2,6)-Galactosylgloboside	not crossing	0	0
acnacngalgbside_hs	Disialyl-Galactosylgloboside	not crossing	0	0
fucfucfucgalacglcgal14acglcgalgluside_hs	(Gal)3 (Glc)1 (GlcNac)2 (LFuc)3 (Cer)1	not crossing	0	0
gt1a_hs	Gt1A	not crossing	0	0
acgalfucgalacgalfuc12gal14acglcgalgluside_hs	Type IIIA Glycolipid	not crossing	0	0
hspg	Heparan Sulfate Proteoglycan	not crossing	0	0
ksii_core2	Keratan Sulfate Ii (Core 2-Linked)	not crossing	0	0
s2l2n2m2masn	De-Fuc Form Of Pa6	not crossing	0	0
s2l2n2m2m	De-Fuc Form Of Pa6 (Wo Peptide Linkage)	not crossing	0	0

gq1b_hs	Gq1B	not crossing	0	0
gq1balpha_hs	Gq1Balpha	not crossing	0	0
ksii_core4	Keratan Sulfate Ii (Core 4-Linked)	not crossing	0	0
s2l2fn2m2masn	Pa6	not crossing	0	0
fucfucfucgalacglc13galacglcgal14acglcgalgluside_hs	(Gal)4 (Glc)1 (GlcNac)3 (LFuc)3 (Cer)1	not crossing	0	0
acgalfucgalacgalfucgalacglcgal14acglcgalgluside_hs	Type III Ab Glycan	not crossing	0	0
psyl	Psyllium	not crossing	0	0
psylchol	Psillium-Glycocholic Acid Complex	not crossing	0	0
psyltdechol	Psyllium-Taurodeoxycholic Acid Complex	not crossing	0	0
psyltchol	Psyllium-Taurocholic Acid Complex	not crossing	0	0
galgalfucfucgalacglcgalacglcgal14acglcgalgluside_hs	(Gal)6 (Glc)1 (GlcNac)3 (LFuc)2 (Cer)1	not crossing	0	0
gp1c_hs	Gp1C	not crossing	0	0
gp1calpha_hs	Gp1C Alpha	not crossing	0	0
Rtotal	R Total	not crossing	0	0
Rtotal2	R Total 2 Position	not crossing	0	0
Rtotal3	R Total 3 Position	not crossing	0	0
Tyr_ggn	Tyr-194 Of Apo-Glycogenin Protein (Primer For Glycogen Synthesis)	not crossing	0	0
aqcobal	Aquacobalamin	not crossing	0	0
10fthf7glu	10-Formyltetrahydrofolate-[Glu](7)	not crossing	0	0
mqn11	Menaquinone-11	not crossing	0	0
hxcoa	Hexanoyl-CoA	not crossing	0	0
mqn10	Menaquinone-10	not crossing	0	0
sphmyln1825_hs	Sm (D18:0/25:0), Sphingomyelin	not crossing	0	0
sphmyln1824_hs	Sm (D18:0/24:0), Sphingomyelin	not crossing	0	0
sphmyln180241_hs	Sm (D18:0/24:1), Sphingomyelin	not crossing	0	0
sphmyln18123_hs	Sm (D18:1/23:0), Sphingomyelin	not crossing	0	0
sphmyln18122_hs	Sm (D18:1/22:0), Sphingomyelin	not crossing	0	0
sphmyln181221_hs	Sm (D18:1/22:1), Sphingomyelin	not crossing	0	0
mqn9	Menaquinone-9	not crossing	0	0
sphmyln18121_hs	Sm (D18:1/21:0), Sphingomyelin	not crossing	0	0
gncore2	GlcNac-Alpha-1,4-Core 2	not crossing	0	0
sphmyln18120_hs	Sm (D18:1/20:0), Sphingomyelin	not crossing	0	0
sphmyln181201_hs	Sm (D18:1/20:1), Sphingomyelin	not crossing	0	0

sphmyln18118_hs	Sm (D18:1/18:0), Sphingomyelin	not crossing	0	0
sphmyln181181_hs	Sm (D18:1/18:1), Sphingomyelin	not crossing	0	0
sphmyln18117_hs	Sm (D18:1/17:0), Sphingomyelin	not crossing	0	0
mqn8	Menaquinone-8	not crossing	0	0
sphmyln18116_hs	Sm (D18:1/16:0), Sphingomyelin	not crossing	0	0
sphmyln181161_hs	Sm (D18:1/16:1), Sphingomyelin	not crossing	0	0
xolest226_hs	Cholesteryl Docosahexanoate, Cholesterol-Ester (22:6, Delta 4,7,10,13,16,19)	not crossing	0	0
sphmyln18115_hs	Sm (D18:1/15:0), Sphingomyelin	not crossing	0	0
sphmyln18114_hs	Sm (D18:1/14:0), Sphingomyelin	not crossing	0	0
xolest204_hs	Cholesteryl Arachidonate, Cholesterol-Ester (20:4, Delta 5,8,11,14)	not crossing	0	0
xolest205_hs	1-Timnodnoyl-Cholesterol, Cholesterol-Ester (20:5, Delta 5,8,11,14,17)	not crossing	0	0
pcholn28_hs	Lysopc A C28:0	not crossing	0	0
pcholn281_hs	Lysopc A C28:1 (Delta 5)	not crossing	0	0
cpppg1	Coproporphyrinogen I	not crossing	0	0
xolest181_hs	1-Vaccenoyl-Cholesterol, Cholesterol-Ester (18:1, Delta 11)	not crossing	0	0
xolest182_hs	1-Linoleoyl-Cholesterol, Cholesterol-Ester (18:2, Delta 9, 12)	not crossing	0	0
mqn7	Menaquinone-7	not crossing	0	0
xolest183_hs	CE(18:3(6Z,9Z,12Z))	not crossing	0	0
pcholn261_hs	Lysopc A C26:1 (Delta 5)	not crossing	0	0
pcholdoc_hs	1-Docosahexaenoylglycerophosphocholine	not crossing	0	0
pailar_hs	1-Arachidonoylglycerophosphoinositol	not crossing	1	0
pcholn24_hs	LysoPC(24:0)	not crossing	0	0
pailste_hs	1-Stearoylglycerophosphoinositol	not crossing	0	0
biliverd	Biliverdin	not crossing	1	1
pcholn224_hs	1-Docosatetraenoylglycerophosphocholine (Delta 7, 10, 13, 16), Sn1-Lpc (22:4)	not crossing	0	0
pailpalm_hs	1-Palmitoylglycerophosphoinositol	not crossing	0	0
pcholn225_hs	1-Docosapentenoylglycerophosphocholine (Delta 7, 10, 13, 16, 19), Sn1-Lpc (22:5)-W3	not crossing	0	0
pcholn2254_hs	1-Docosapentenoylglycerophosphocholine (Delta 4, 7, 10, 13, 16), Sn1-Lpc (22:5)-W6	not crossing	0	0
gncore1	GlcNac-Alpha-1,4-Core 1	not crossing	0	0
f1a	F1Alpha	not crossing	0	0
pcholn226_hs	1-Docosahexenoylglycerophosphocholine (Delta 4, 7, 10, 13, 16, 19), Sn1-Lpc (22:6)	not crossing	0	0
pcholn201_hs	1-Eicosenoylglycerophosphocholine (Delta 11) ,Sn1-Lpc (20:1)	not crossing	0	0
pcholeic_hs	1-Eicosadienoylglycerophosphocholine (Delta 11,14)	not crossing	0	0
pcholn203_hs	1-Dihomo-Linolenoylglycerophosphocholine (20:3, Delta 8, 11, 14), Lysopc A C20:3	not crossing	0	0

pcholet_hs	1-Eicosatrienoylglycerophosphocholine (Delta 11, 14, 17)	not crossing	0	0
pcholar_hs	1-Arachidonoyl-Glycero-3-Phosphocholine	not crossing	0	0
pcholn204_hs	LysoPC(20:4(8Z,11Z,14Z,17Z))	not crossing	0	0
pcholn205_hs	1-Eicosapentenoylglycerophosphocholine (Delta 5, 8, 11, 14, 17), Sn1-Lpc (20:5)	not crossing	0	0
pcholn19_hs	1-Nonadecanoylglycerophosphocholine, Sn1-Lpc (19:0)	not crossing	0	0
pe224_hs	1-Docosatetraenoyglycerophosphoethanolamine (22:4, Delta 7, 10, 13, 16)	not crossing	0	0
pe226_hs	1-Docosahexenoylglyceroethanolamine (Delta 4, 7, 10, 13, 16, 19), Lpe (22:6)	not crossing	0	0
pcholste_hs	LysoPC(18:0)	not crossing	1	0
pchol2ste_hs	2-Stearoylglycerophosphocholine	not crossing	1	0
pcholole_hs	LysoPC(18:1(9Z))	not crossing	0	0
pchol2ole_hs	2-Oleoylglycerophosphocholine	not crossing	0	0
pchol2linl_hs	2-Linoleoylglycerophosphocholine	not crossing	0	0
pchollinl_hs	1-Linoleoylglycerophosphocholine (Delta 9,12)	not crossing	0	0
pcholn183_hs	1-Octadeca-Trienoylglycerophosphocholine, Sn1-Lpc (18:3, Delta 9, 12, 15)	not crossing	0	0
pcholn1836_hs	1-Octadeca-Trienoylglycerophosphocholine, Sn1-Lpc (18:3, Delta 6, 9, 12)	not crossing	0	0
HC02197	Sulfolithocholylglycine	not crossing	1	0
pcholhep_hs	LysoPC(17:0)	not crossing	1	0
pe203_hs	1-Eicosatrienoylglycerophosphoethanolamine (Delta 11, 14, 17), Lpe (20:3)	not crossing	0	0
pedh203_hs	1-Dihomo-Linolenoylglycerophosphoethanolamine (20:3, Delta 8, 11, 14)	not crossing	0	0
pear_hs	1-Arachidonoyl-Sn-Glycero-3-Phosphoethanolamine	not crossing	0	0

Supplementary Table 6: The newly added metabolites in global model refinement.

Sources	metName	VMHId	metFormula	ChEBIID	KEGGId	PubChem ID	HMDBId	InChi	InChiKey	Charge
iDopaNeuro CT model	3,4-Dihydroxybenzaldehyde	3,4-dihydroxybenzaldehyde	C7H6O3	CHEBI:50205	C16700	8768	HMDB0059965	InChI=1S/C7H6O3/c8-4-5-1-2-6(9)7(10)3-5/h1-4,9-10H	IBGBGRVKPALMCQ-UHFFFAOYSA-N	0
	quinonemethide	quinonemethide								0
	methanimine	methanimine	CH3N	CHEBI:38220				InChI=1S/CH3N/c1-2/h2H,1H2	WDWDWGRYHDPSDS-UHFFFAOYSA-N	0
fatty acid oxidation metabolism	3E-octenoyl-CoA	3eoctecoa								0
	3Z-octenoyl-CoA	3zoctecoa								0
	2E,4Z-decadienoyl-CoA	2e4zdecadecoa								0
	5E-decenoyl-CoA	5edececoa								0
	3Z-decenoyl-CoA	3zdececoa								0
	5Z-decenoyl-CoA	5zdececoa								0
	7E-dodecenoyl-CoA	7edodeecoa								0
	7Z-dodecenoyl-CoA	7zdodeecoa								0
	9Z-tetradecenoyl-CoA	9ztdececoa								0
	3Z-tetradecenoyl-CoA	3ztdececoa								0
	5Z-hexadecenoyl-CoA	5zhexaecoa								0
	11Z-hexadecenoyl-CoA	11zhexaecoa								0
	3E,7Z-hexadecadienoyl-CoA	3e7zhexadecoa								0
	4Z,7Z-hexadecadienoyl-CoA	4z7zhexadecoa								0
	3E,4Z,7Z-hexadecatrienoyl-CoA	3e4z7zhexatecoa								0
	2E,6Z,9Z-octadecatrienoyl-CoA	2e6z9zoctatecoa								0
	3Z,6Z,9Z-octadecatrienoyl-CoA	3z6z9zoctatecoa								0
Caffeine metabolism	Caffeine	cfn1	C8H10N4O2	CHEBI:27732	C07481	2519	HMDB01847	InChI=1S/C8H10N4O2/c1-10-4-9-6-5(10)7(13)12(3)8(14)11(6)2/h4H,1-3H3	RYYVLZVUVIJVGH-UHFFFAOYSA-N	0
	Paraxanthine	pxthn1	C7H8N4O2	CHEBI:25858	C13747	4687	HMDB01860	InChI=1S/C7H8N4O2/c1-10-3-8-5-4(10)6(12)11(2)7(13)9-5/h3H,1-2H3,(H,9,13)	QUNWUDVFRNGTCO-UHFFFAOYSA-N	0
	1-Methylxanthine	1mxnt	C6H6N4O2	CHEBI:68444	C16358	80220	HMDB10738	InChI=1S/C6H6N4O2/c1-10-5(11)3-4(8-2-7-3)9-6(10)12/h2H,1H3,(H,7,8)(H,9,12)	MVOYJPOZRLFTCP-UHFFFAOYSA-N	0
	Theobromine	theobromine1	C7H8N4O2	CHEBI:28946	C07480	5429	HMDB02825	InChI=1S/C7H8N4O2/c1-10-3-8-5-4(10)6(12)9-7(13)11(5)2/h3H,1-2H3,(H,9,12,13)	YAPQBXQYLJRXSA-UHFFFAOYSA-N	0
	5-Acetylamino-6-formylamino-3-methyluracil	5acam6fam3mura	C8H10N4O4	CHEBI:32643	C16365	108214	HMDB11105	InChI=1S/C8H10N4O4/c1-4(14)10-5-6(9-3-13)11-8(16)12(2)7(5)15/h3H,1-2H3,(H,9,13)(H,10,14)(H,11,16)	RDZNZFGKEVDNPK-UHFFFAOYSA-N	0
	Theophylline	theophylline1	C7H8N4O2	CHEBI:28177	C07130	2153	HMDB01889	InChI=1S/C7H8N4O2/c1-10-5-4(8-3-9-5)6(12)11(2)7(10)13/h3H,1-2H3,(H,8,9)	ZFXYFBGIUFBOJW-UHFFFAOYSA-N	0
	5-Acetylamino-6-amino-3-methyluracil	5acam6am3mura	C7H10N4O3	CHEBI:80473	C16366	88299	HMDB04400	InChI=1S/C7H10N4O3/c1-3(12)9-4-5(8)10-7(14)11(2)6(4)13/h8H2,1-2H3,(H,9,12)(H,10,14)	POQOTWQIYYNXAT-UHFFFAOYSA-N	0
	1,7-Dimethyluric acid	17dmurt1	C7H8N4O3	CHEBI:68449	C16356	91611	HMDB11103	InChI=1S/C7H8N4O3/c1-10-3-4(8-6(10)13)9-7(14)11(2)5(3)12/h1-2H3,(H,8,13)(H,9,14)	NOFNCLGCUJJPKU-UHFFFAOYSA-N	0
	1,3,7-Trimethyluric acid	137tmurica1	C8H10N4O3	CHEBI:691622	C16361	79437	HMDB02123	InChI=1S/C8H10N4O3/c1-10-4-5(9-7(10)14)11(2)8(15)12(3)6(4)13/h1-3H3,(H,9,14)	BYXCFUMGEBZDDI-UHFFFAOYSA-N	0
	7-Methylxanthine	7mxth	C6H6N4O2	CHEBI:48991	C16353		HMDB01991	InChI=1S/C6H6N4O2/c1-10-2-7-4-3(10)5(11)9-6(12)8-4/h2H,1H3,(H2,8,9,11,12)	PFWLFWPASULGAN-UHFFFAOYSA-N	0

	1-Methyluric acid	1murt	C6H6N4O3	CHEBI:68441	C16359		HMDB03099	InChI=1S/C6H6N4O3/c1-10-4(11)2-3(9-6(10)13)8-5(12)7-2/h1H3,(H,9,13)(H2,7,8,12)	QFDRTQONISXGJA-UHFFFAOYSA-N	0
	7-Methyluric acid	7murt	C6H6N4O3		C16355	69160	HMDB11107	InChI=1S/C6H6N4O3/c1-10-2-3(8-6(10)13)7-5(12)9-4(2)11/h1H3,(H3,7,8,9,11,12,13)	YHNNPKUFPWLTOP-UHFFFAOYSA-N	0
	3,7-Dimethyluric acid	37dmur	C7H8N4O3	CHEBI:68531	C16360	83126	HMDB01982	InChI=1S/C7H8N4O3/c1-10-3-4(8-6(10)13)11(2)7(14)9-5(3)12/h1-2H3,(H,8,13)(H,9,12,14)	HMLZLHKHNBLLJD-UHFFFAOYSA-N	0
	(2R,6S,10S)-pristanate	MAM00077[x]	C19H37O2							-1
	20-hydroxy-arachidonate	MAM00591[c]	C20H31O3							-1
	20-hydroxy-arachidonate	MAM00591[e]	C20H31O3							-1
	NH3	MAM02578[c]	H3N							0
	NH3	MAM02578[e]	H3N							0
	steroids	MAM10001[e]								0
	xenobiotics	MAM10002[e]								0
	arachidonate derivatives	MAM10003[e]								0
	1-acylglycerol-3P pool	MAM10006[c]	C4H6O7PR							-2
	acyl-CoA pool	MAM10007[c]	C22H31N7O17P3SR							-4
	acyl-CoA pool	MAM10007[x]	C22H31N7O17P3SR							-4
	cofactor_pool_biomass	MAM10012[c]								0
	protein_pool_biomass	MAM10013[c]								0
	lipid_pool_biomass	MAM10014[c]								0
Human1 model	metabolite_pool_biomass	MAM10015[c]								0
	2,3-epoxy-2,3-dihydro-2-methyl-3-phytyl-1,4-naphthoquinone	MAM10016[c]	C31H46O3							0
	3-hydroxy-2-methyl-3-phytyl-2,3-dihydronaphthoquinone	MAM10017[c]	C31H48O3							0
	5-Hydroxy-2-oxo-4-ureido-2,5-dihydro-1H-imidazole-5-carboxylate	MAM10018[c]	C5H5N4O5							-1
	5alpha-Pregnan-20alpha-ol-3-one	MAM10019[c]	C21H34O2							0
	17alpha,20alpha-Dihydroxypregn-4-en-3-one	MAM10020[c]	C21H32O3							0
	alpha-muricholic acid	MAM10021[c]	C24H39O5							-1
	alpha-muricholic acid	MAM10021[e]	C24H39O5							-1
	beta-muricholic acid	MAM10022[c]	C24H39O5							-1
	beta-muricholic acid	MAM10022[e]	C24H39O5							-1
	dehydrocholic acid	MAM10023[c]	C24H33O5							-1
	dehydrocholic acid	MAM10023[e]	C24H33O5							-1
	tauro-alpha-muricholic acid	MAM10024[c]	C26H44NO7S							-1
	tauro-alpha-muricholic acid	MAM10024[e]	C26H44NO7S							-1
	omega-muricholic acid	MAM10025[c]	C24H39O5							-1

omega-muricholic acid	MAM10025[e]	C24H39O5	-1
glycohyocholic acid	MAM10027[c]	C26H42NO6	-1
glycohyocholic acid	MAM10027[e]	C26H42NO6	-1
glycohyodeoxycholic acid	MAM10028[c]	C26H42NO5	-1
glycohyodeoxycholic acid	MAM10028[e]	C26H42NO5	-1
glycodehydrocholic acid	MAM10029[c]	C26H36NO6	-1
glycodehydrocholic acid	MAM10029[e]	C26H36NO6	-1
tauro-omega-muricholic acid	MAM10031[c]	C26H44NO7S	-1
tauro-omega-muricholic acid	MAM10031[e]	C26H44NO7S	-1
tauro-beta-muricholic acid	MAM10032[c]	C26H44NO7S	-1
tauro-beta-muricholic acid	MAM10032[e]	C26H44NO7S	-1
murideoxycholic acid	MAM10033[c]	C24H39O4	-1
murideoxycholic acid	MAM10033[e]	C24H39O4	-1
taurodehydrocholic acid	MAM10034[c]	C26H38NO7S	-1
taurodehydrocholic acid	MAM10034[e]	C26H38NO7S	-1
alpha-muricholoyl-CoA	MAM10035[c]	C45H70N7O20<br>P3S	-4
beta-muricholoyl-CoA	MAM10036[c]	C45H70N7O20<br>P3S	-4
omega-muricholoyl-CoA	MAM10037[c]	C45H70N7O20<br>P3S	-4
hyocholoyl-CoA	MAM10038[c]	C45H70N7O20<br>P3S	-4
dehydrocholoyl-CoA	MAM10039[c]	C45H64N7O20<br>P3S	-4
hyodeoxycholoyl-CoA	MAM10040[c]	C45H70N7O19<br>P3S	-4
tauro-hyodeoxycholic acid	MAM10041[c]	C26H44NO6S	-1
tauro-hyodeoxycholic acid	MAM10041[e]	C26H44NO6S	-1
ursodeoxycholoyl-CoA	MAM10042[c]	C45H70N7O19<br>P3S	-4
hydroxyl radical	MAM02149[c]	HO	0

Supplementary Table 7: The newly added reactions in global model refinement.

Sources	rxnID	rxnNames/description	rxnFormular	subsystems	lb	ub	grRules	enzymes
iDopaNeuro CT model	acleua	N-Acetyl-leucine acylase	acleu_L[c] + h2o[c] --> ac[c] + leu_L[c]	Valine, leucine, and isoleucine metabolism				
	acthra	Acetyl-Threonine acylase	acthr_L[c] + h2o[c] --> ac[c] + thr_L[c]	Glycine, serine, alanine, and threonine metabolism				
	acileua	N-Acetyl-isoleucine acylase	acile_L[c] + h2o[c] --> ac[c] + ile_L[c]	Valine, leucine, and isoleucine metabolism				
	acglua	N-Acetyl-glutamate acylase	acglu[c] + h2o[c] --> ac[c] + glu_L[c]	Glutamate metabolism				
	CE1554tm	N-Acetyl-Alanine transport	CE1554[c] <==> CE1554[m]	Alanine metabolism				
	DM_ps_hs[c]	Phosphatidylserine demand	ps_hs[c] <==>	Glycerophospholipid metabolism				
	CYSTS_H2S	Cystathionine Beta-Synthase (sulfide-forming)	cys_L[c] + hcys_L[c] <==> cyst_L[c] + HC00250[c]'	Methionine and cysteine metabolism				
	DHBOX	3,4-dihydroxybenzaldehyde oxidase	quinonemethide[c] + h2o2[c] --> 3,4-dihydroxybenzaldehyde[c] + methanimine[c]	Miscellaneous				
	DM_clpn_hs[c]	Demand of cardiolipin	clpn_hs[c] -->	Exchange/demand reaction				
	EX_adocbl[e]	Exchange of adenosylcobalamin	adocbl[e] <==>	Exchange/demand reaction				
	EX_selni[c]	Exchange of selenite	selni[c] <==>	Exchange/demand reaction				
	EX_zn2[e]	Exchange of zinc (II) ion	zn2[e] <==>	Exchange/demand reaction				
	NORCON	Norsalsolinol consendation	3,4-dihydroxybenzaldehyde[c] + fald[c] + dopa[c] --> CE2172[c]	Miscellaneous				
	Q-METHRED	Quinonemethide reductase	CE5276[c] + fe2[c] --> quinonemethide[c]	Miscellaneous				
fatty acid oxidation metabolism	ACADM1		h2o[m] + nad[m] + coa[m] + hdd2coa[m] --> h[m] + nadh[m] + accoa[m] + tdcoa[m]	Fatty acid oxidation	0	1000	1892.1 and 3030.1 and 3032.1	2-enoyl CoA hydratase; 3-hydroxylacyl CoA dehydrogenase; 3-Ketoacyl-CoA thiolase
	ACADM2		h2o[m] + nad[m] + coa[m] + fad[m] + dd5ecoa[m] --> h[m] + nadh[m] + accoa[m] + 3zdececoa[m] + fadh2[m]	Fatty acid oxidation	0	1000	34.1 and 1892.1 and 3030.1 and 3032.1	Medium chain acyl CoA dehydrogenase; 2-enoyl CoA hydratase; 3-hydroxylacyl CoA dehydrogenase; 3-Ketoacyl-CoA thiolase
	ACADM3	Isomerization (C10:1), Mitochondrial	3zdececoa[m] <==> dc2coa[m]	Fatty acid oxidation	-1000	1000	1632.1	3,2- trans- Enoyl CoA isomerase
	ACADM4		h2o[m] + nad[m] + coa[m] + od2coa[m] --> h[m] + nadh[m] + accoa[m] + pmtcoa[m]	Fatty acid oxidation	0	1000	1892.1 and 3030.1 and 3032.1	2-enoyl CoA hydratase; 3-hydroxylacyl CoA dehydrogenase; 3-Ketoacyl-CoA thiolase
	ACADM5	Fatty Acid Beta Oxidation (C18:1->C16:1), Mitochondrial	h2o[m] + nad[m] + coa[m] + fad[m] + odecoa[m] --> h[m] + nadh[m] + accoa[m] + M01191[m] + fadh2[m]	Fatty acid oxidation	0	1000	37.1 and 1892.1 and 3030.1 and 3032.1	Long chain/Very long chain acyl CoA dehydrogenase; 2-enoyl CoA hydratase; 3-hydroxylacyl CoA dehydrogenase; 3-Ketoacyl-CoA thiolase
	ACADM6	Fatty Acid Beta Oxidation (C16:1->C14:1), Mitochondrial	h2o[m] + nad[m] + coa[m] + fad[m] + M01191[m] --> h[m] + nadh[m] + accoa[m] + M01141[m] + fadh2[m]	Fatty acid oxidation	0	1000	37.1 and 1892.1 and 3030.1 and 3032.1	Long chain/Very long chain acyl CoA dehydrogenase; 2-enoyl CoA hydratase; 3-hydroxylacyl CoA dehydrogenase; 3-Ketoacyl-CoA thiolase
	ACADM7	Fatty Acid Beta Oxidation (C14:1->C12:1), Mitochondrial	h2o[m] + nad[m] + coa[m] + fad[m] + M01141[m] --> h[m] + nadh[m] + accoa[m] + dd3coa[m] + fadh2[m]	Fatty acid oxidation	0	1000	37.1 and 1892.1 and 3030.1 and 3032.1	Long chain/Very long chain acyl CoA dehydrogenase; 2-enoyl CoA hydratase; 3-hydroxylacyl CoA dehydrogenase;

							3-Ketoacyl-CoA thiolase
ACADM8		h2o[m] + nad[m] + coa[m] + dd2coa[m] --> h[m] + nadh[m] + accoa[m] + dcacoa[m]	Fatty acid oxidation	0	1000	1892.1 and 3030.1 and 3032.1	2-enoyl CoA hydratase; 3-hydroxylacyl CoA dehydrogenase; 3-Ketoacyl-CoA thiolase
ACADM9		dece4coa[m] --> dece3coa[m]	Fatty acid oxidation	0	1000	34.1 and 1666.1	Medium chain acyl CoA dehydrogenase; 2,4-dienoyl reductase
ACADM10	Fatty Acid Beta Oxidation (C18:2->C16:2), Mitochondrial	h2o[m] + nad[m] + coa[m] + fad[m] + lneldccoa[m] --> h[m] + nadh[m] + accoa[m] + 4z7zhexadecoa[m] + fadh2[m]	Fatty acid oxidation	0	1000	37.1 and 1892.1 and 3030.1 and 3032.1	Long chain/Very long chain acyl CoA dehydrogenase; 2-enoyl CoA hydratase; 3-hydroxylacyl CoA dehydrogenase; 3-Ketoacyl-CoA thiolase
ACADM11		4z7zhexadecoa[m] --> 3e7zhexadecoa[m]	Fatty acid oxidation	0	1000	37.1 and 1666.1 and 1632.1	Long chain/Very long chain acyl CoA dehydrogenase; 2,4-dienoyl reductase; 3,2- trans- Enoyl CoA isomerase
ACADM12		3e7zhexadecoa[m] <==> M03019[m]	Fatty acid oxidation	-1000	1000	37.1 and 1666.1 and 1632.1	Long chain/Very long chain acyl CoA dehydrogenase; 2,4-dienoyl reductase; 3,2- trans- Enoyl CoA isomerase
ACADM13		h2o[m] + nad[m] + coa[m] + M03019[m] --> h[m] + nadh[m] + accoa[m] + M01141[m]	Fatty acid oxidation	0	1000	1892.1 and 3030.1 and 3032.1	2-enoyl CoA hydratase; 3-hydroxylacyl CoA dehydrogenase; 3-Ketoacyl-CoA thiolase
ACADM14		dec47dicoa[m] --> dectricoa[m]	Fatty acid oxidation	0	1000	34.1	Medium chain acyl CoA dehydrogenase
ACADM15		dectricoa[m] --> 3decdicoa[m]	Fatty acid oxidation	0	1000	1666.1	2,4-dienoyl reductase
ACADM16	Acyl-CoA Oxidase	h2o[m] + nad[m] + coa[m] + o2[m] + lnlncgcoa[m] --> h[m] + nadh[m] + accoa[m] + 4hexdtricoa[m] + h2o2[m]	Fatty acid oxidation	0	1000	51.1 and 1892.1 and 3030.1 and 3032.1	Acyl CoA oxidase; 2-enoyl CoA hydratase; 3-hydroxylacyl CoA dehydrogenase; 3-Ketoacyl-CoA thiolase
ACADM17		4hexdtricoa[m] --> hexdectecoa[m]	Fatty acid oxidation	0	1000	37.1	Long chain/Very long chain acyl CoA dehydrogenase
ACADM18		hexdectecoa[m] --> 3hexdtricoa[m]	Fatty acid oxidation	0	1000	1666.1	2,4-dienoyl reductase
ACADM19		dece4coa[m] --> 2e4zdecadecoa[m]	Fatty acid oxidation	0	1000	34.1	Medium chain acyl CoA dehydrogenase
ACADM20		2e4zdecadecoa[m] --> dece3coa[m]	Fatty acid oxidation	0	1000	1666.1	2,4-dienoyl reductase
ACADM21	Fatty Acid Beta Oxidation (C14:1->C12:1), Mitochondrial	h2o[m] + nad[m] + coa[m] + fad[m] + M00129[m] --> h[m] + nadh[m] + accoa[m] + 7edodeecoa[m] + fadh2[m]	Fatty acid oxidation	0	1000	37.1 and 1892.1 and 3030.1 and 3032.1	Long chain/Very long chain acyl CoA dehydrogenase; 2-enoyl CoA hydratase; 3-hydroxylacyl CoA dehydrogenase; 3-Ketoacyl-CoA thiolase
ACADM22	Fatty Acid Beta Oxidation (C12:1->C10:1), Mitochondrial	h2o[m] + nad[m] + coa[m] + fad[m] + 7edodeecoa[m] --> h[m] + nadh[m] + accoa[m] + 5edececoa[m] + fadh2[m]	Fatty acid oxidation	0	1000	34.1 and 1892.1 and 3030.1 and 3032.1	Medium chain acyl CoA dehydrogenase; 2-enoyl CoA hydratase; 3-hydroxylacyl CoA dehydrogenase; 3-Ketoacyl-CoA thiolase
ACADM23	Fatty Acid Beta Oxidation (C10:1->C8:1), Mitochondrial	h2o[m] + nad[m] + coa[m] + fad[m] + 5edececoa[m] --> h[m] + nadh[m] + accoa[m] + 3eoctecoa[m] + fadh2[m]	Fatty acid oxidation	0	1000	34.1 and 1892.1 and 3030.1 and 3032.1	Medium chain acyl CoA dehydrogenase; 2-enoyl CoA hydratase; 3-hydroxylacyl CoA dehydrogenase; 3-Ketoacyl-CoA thiolase

ACADM24	Isomerization (C8:1), Mitochondrial	3eoctecoa[m] <==> HC01415[m]	Fatty acid oxidation	-1000	1000	1632.1	3,2- trans- Enoyl CoA isomerase
ACADM25		h2o[m] + nad[m] + coa[m] + HC01415[m] --> h[m] + nadh[m] + accoa[m] + hxcoa[m]	Fatty acid oxidation	0	1000	1892.1 and 3030.1 and 3032.1	2-enoyl CoA hydratase; 3-hydroxylacyl CoA dehydrogenase; 3-Ketoacyl-CoA thiolase
ACADM26	Fatty Acid Beta Oxidation (C18:1->C16:1), Mitochondrial	h2o[m] + nad[m] + coa[m] + fad[m] + M00020[m] --> h[m] + nadh[m] + accoa[m] + 11zhexaecoa[m] + fadh2[m]	Fatty acid oxidation	0	1000	37.1 and 1892.1 and 3030.1 and 3032.1	Long chain/Very long chain acyl CoA dehydrogenase; 2-enoyl CoA hydratase; 3-hydroxylacyl CoA dehydrogenase; 3-Ketoacyl-CoA thiolase
ACADM27	Fatty Acid Beta Oxidation (C16:1->C14:1), Mitochondrial	h2o[m] + nad[m] + coa[m] + fad[m] + 11zhexaecoa[m] --> h[m] + nadh[m] + accoa[m] + 9ztdececoa[m] + fadh2[m]	Fatty acid oxidation	0	1000	37.1 and 1892.1 and 3030.1 and 3032.1	Long chain/Very long chain acyl CoA dehydrogenase; 2-enoyl CoA hydratase; 3-hydroxylacyl CoA dehydrogenase; 3-Ketoacyl-CoA thiolase
ACADM28	Fatty Acid Beta Oxidation (C14:1->C12:1), Mitochondrial	h2o[m] + nad[m] + coa[m] + fad[m] + 9ztdececoa[m] --> h[m] + nadh[m] + accoa[m] + 7zdodeecoa[m] + fadh2[m]	Fatty acid oxidation	0	1000	37.1 and 1892.1 and 3030.1 and 3032.1	Long chain/Very long chain acyl CoA dehydrogenase; 2-enoyl CoA hydratase; 3-hydroxylacyl CoA dehydrogenase; 3-Ketoacyl-CoA thiolase
ACADM29	Fatty Acid Beta Oxidation (C12:1->C10:1), Mitochondrial	h2o[m] + nad[m] + coa[m] + fad[m] + 7zdodeecoa[m] --> h[m] + nadh[m] + accoa[m] + 5zdececoa[m] + fadh2[m]	Fatty acid oxidation	0	1000	34.1 and 1892.1 and 3030.1 and 3032.1	Medium chain acyl CoA dehydrogenase; 2-enoyl CoA hydratase; 3-hydroxylacyl CoA dehydrogenase; 3-Ketoacyl-CoA thiolase
ACADM30	Fatty Acid Beta Oxidation (C10:1->C8:1), Mitochondrial	h2o[m] + nad[m] + coa[m] + fad[m] + 5zdececoa[m] --> h[m] + nadh[m] + accoa[m] + 3zoctecoa[m] + fadh2[m]	Fatty acid oxidation	0	1000	34.1 and 1892.1 and 3030.1 and 3032.1	Medium chain acyl CoA dehydrogenase; 2-enoyl CoA hydratase; 3-hydroxylacyl CoA dehydrogenase; 3-Ketoacyl-CoA thiolase
ACADM31	Isomerization (C8:1), Mitochondrial	3zoctecoa[m] <==> HC01415[m]	Fatty acid oxidation	-1000	1000	1632.1	3,2- trans- Enoyl CoA isomerase
ACADM32		M00127[m] --> ocde9ecoa[m]	Fatty acid oxidation	0	1000		Thiokinase
ACADM33	Fatty Acid Beta Oxidation (C18:1->C16:1), Mitochondrial	h2o[m] + nad[m] + coa[m] + fad[m] + M00116[m] --> h[m] + nadh[m] + accoa[m] + 5zhexaecoa[m] + fadh2[m]	Fatty acid oxidation	0	1000	37.1 and 1892.1 and 3030.1 and 3032.1	Long chain/Very long chain acyl CoA dehydrogenase; 2-enoyl CoA hydratase; 3-hydroxylacyl CoA dehydrogenase; 3-Ketoacyl-CoA thiolase
ACADM34	Fatty Acid Beta Oxidation (C16:1->C14:1), Mitochondrial	h2o[m] + nad[m] + coa[m] + fad[m] + 5zhexaecoa[m] --> h[m] + nadh[m] + accoa[m] + 3ztdececoa[m] + fadh2[m]	Fatty acid oxidation	0	1000	37.1 and 1892.1 and 3030.1 and 3032.1	Long chain/Very long chain acyl CoA dehydrogenase; 2-enoyl CoA hydratase; 3-hydroxylacyl CoA dehydrogenase; 3-Ketoacyl-CoA thiolase
ACADM35	Isomerization (C14:1), Mitochondrial	3ztdececoa[m] <==> tetdece1coa[m]	Fatty acid oxidation	-1000	1000	1632.1	3,2- trans- Enoyl CoA isomerase
ACADM36		h2o[m] + nad[m] + coa[m] + tetdece1coa[m] --> h[m] + nadh[m] + accoa[m] + ddcacoa[m]	Fatty acid oxidation	0	1000	1892.1 and 3030.1 and 3032.1	2-enoyl CoA hydratase; 3-hydroxylacyl CoA dehydrogenase; 3-Ketoacyl-CoA thiolase
ACADM37	Fatty Acid Beta Oxidation (C18:2->C16:2), Mitochondrial	h2o[m] + nad[m] + coa[m] + fad[m] + lneldccoa[m] --> h[m] + nadh[m] + accoa[m] + 4z7zhexadecoa[m] + fadh2[m]	Fatty acid oxidation	0	1000	37.1 and 1892.1 and 3030.1 and 3032.1	Long chain/Very long chain acyl CoA dehydrogenase; 2-enoyl CoA hydratase; 3-hydroxylacyl CoA dehydrogenase;

							3-Ketoacyl-CoA thiolase
ACADM38	Fatty Acid Beta Oxidation (C20:3->C18:3), Mitochondrial	h2o[m] + nad[m] + coa[m] + fad[m] + M00101[m] --> h[m] + nadh[m] + accoa[m] + 3z6z9zoctatecoa[m] + fadh2[m]	Fatty acid oxidation	0	1000	37.1 and 1892.1 and 3030.1 and 3032.1	Long chain/Very long chain acyl CoA dehydrogenase; 2-enoyl CoA hydratase; 3-hydroxylacyl CoA dehydrogenase; 3-Ketoacyl-CoA thiolase
ACADM39		h2o[m] + nad[m] + coa[m] + fad[m] + M01236[m] --> h[m] + nadh[m] + accoa[m] + M00116[m] + fadh2[m]	Fatty acid oxidation	0	1000	37.1 and 1892.1 and 3030.1 and 3032.1	Long chain/Very long chain acyl CoA dehydrogenase; 2-enoyl CoA hydratase; 3-hydroxylacyl CoA dehydrogenase; 3-Ketoacyl-CoA thiolase
ACADM40		h2o[m] + nad[m] + coa[m] + fad[m] + M00123[m] --> h[m] + nadh[m] + accoa[m] + lneldccoa[m] + fadh2[m]	Fatty acid oxidation	0	1000	37.1 and 1892.1 and 3030.1 and 3032.1	Long chain/Very long chain acyl CoA dehydrogenase; 2-enoyl CoA hydratase; 3-hydroxylacyl CoA dehydrogenase; 3-Ketoacyl-CoA thiolase
ACADM41	Isomerization (C18:3), Mitochondrial	3z6z9zoctatecoa[m] <==> 2e6z9zoctatecoa[m]	Fatty acid oxidation	-1000	1000	1632.1	3,2- trans- Enoyl CoA isomerase
ACADM42		h2o[m] + nad[m] + coa[m] + 2e6z9zoctatecoa[m] --> h[m] + nadh[m] + accoa[m] + 4z7zhexadecoa[m]	Fatty acid oxidation	0	1000	1892.1 and 3030.1 and 3032.1	2-enoyl CoA hydratase; 3-hydroxylacyl CoA dehydrogenase; 3-Ketoacyl-CoA thiolase
ACADM43		4z7zhexadecoa[m] --> 3e4z7zhexatecoa[m]	Fatty acid oxidation	0	1000	37.1 and 1666.1	Long chain/Very long chain acyl CoA dehydrogenase; 2,4-dienoyl reductase
ACADM44	Isomerization (C16:2), Mitochondrial	3e4z7zhexatecoa[m] <==> M03019[m]	Fatty acid oxidation	-1000	1000	1632.1	3,2- trans- Enoyl CoA isomerase
ACADM45	Fatty Acid Beta Oxidation (C22:1->C20:1), Mitochondrial	h2o[m] + nad[m] + coa[m] + fad[m] + M00006[m] --> h[m] + nadh[m] + accoa[m] + M01236[m] + fadh2[m]	Fatty acid oxidation	0	1000	37.1 and 1892.1 and 3030.1 and 3032.1	Long chain/Very long chain acyl CoA dehydrogenase; 2-enoyl CoA hydratase; 3-hydroxylacyl CoA dehydrogenase; 3-Ketoacyl-CoA thiolase
ACADM46		dcsptn1coa[m] --> docohexcoa[m]	Fatty acid oxidation	0	1000	37.1	Long chain/Very long chain acyl CoA dehydrogenase
ACADM47		docohexcoa[m] --> 3docopencoa[m]	Fatty acid oxidation	0	1000	1666.1	2,4-dienoyl reductase
ACADM48		2e4zdecadecoa[m] --> dece3coa[m]	Fatty acid oxidation	0	1000	1666.1	2,4-dienoyl reductase
ACADM49	Fatty Acid Beta Oxidation (C20:2->C18:2), Mitochondrial	h2o[m] + nad[m] + coa[m] + fad[m] + CE4843[m] --> h[m] + nadh[m] + accoa[m] + lnlccoa[m] + fadh2[m]	Fatty acid oxidation	0	1000	37.1 and 1892.1 and 3030.1 and 3032.1	Long chain/Very long chain acyl CoA dehydrogenase; 2-enoyl CoA hydratase; 3-hydroxylacyl CoA dehydrogenase; 3-Ketoacyl-CoA thiolase
ACADM50	Fatty Acid Beta Oxidation (C22:2->C20:2), Mitochondrial	h2o[m] + nad[m] + coa[m] + fad[m] + M00023[m] --> h[m] + nadh[m] + accoa[m] + CE4843[m] + fadh2[m]	Fatty acid oxidation	0	1000		
ACADM51	Fatty Acid Beta Oxidation (C22:3->C20:3), Mitochondrial	h2o[m] + nad[m] + coa[m] + fad[m] + CE4847[m] --> h[m] + nadh[m] + accoa[m] + dlnlcgcoa[m] + fadh2[m]	Fatty acid oxidation	0	1000	37.1 and 1892.1 and 3030.1 and 3032.1	Long chain/Very long chain acyl CoA dehydrogenase; 2-enoyl CoA hydratase; 3-hydroxylacyl CoA dehydrogenase; 3-Ketoacyl-CoA thiolase
ACADM52		4hexdtricoa[m] --> 3hexdtricoa[m]	Fatty acid oxidation	0	1000	37.1 and 1666.1	Long chain/Very long chain acyl CoA dehydrogenase; 2,4-dienoyl reductase

ACADM53		dec47dicoa[m] --> 3decdicoa[m]	Fatty acid oxidation	0	1000	34.1 and 1666.1	Medium chain acyl CoA dehydrogenase; 2,4-dienoyl reductase
ACADM54		4hexdtetcoa[m] --> hexdpencoa[m]	Fatty acid oxidation	0	1000	37.1	Long chain/Very long chain acyl CoA dehydrogenase
ACADM55		hexdpencoa[m] --> 3hexdtetcoa[m]	Fatty acid oxidation	0	1000	1666.1	2,4-dienoyl reductase
ACADM56		4hexdtetcoa[m] --> 3hexdtetcoa[m]	Fatty acid oxidation	0	1000	37.1 and 1666.1	Long chain/Very long chain acyl CoA dehydrogenase; 2,4-dienoyl reductase
ACADM57	Fatty Acid Beta Oxidation (C20:5), Mitochondrial	h2o[m] + nad[m] + coa[m] + fad[m] + clpndcoa[m] --> h[m] + nadh[m] + accoa[m] + 5eipencoa[m] + fadh2[m]	Fatty acid oxidation	0	1000	37.1 and 1892.1 and 3030.1 and 3032.1	Long chain/Very long chain acyl CoA dehydrogenase; 2-enoyl CoA hydratase; 3-hydroxylacyl CoA dehydrogenase; 3-Ketoacyl-CoA thiolase
ACADM58	Fatty Acid Beta Oxidation (C20:3->C18:3), Mitochondrial	h2o[m] + nad[m] + coa[m] + fad[m] + M00012[m] --> h[m] + nadh[m] + accoa[m] + lnlncacoa[m] + fadh2[m]	Fatty acid oxidation	0	1000	37.1 and 1892.1 and 3030.1 and 3032.1	Long chain/Very long chain acyl CoA dehydrogenase; 2-enoyl CoA hydratase; 3-hydroxylacyl CoA dehydrogenase; 3-Ketoacyl-CoA thiolase
ACADM59	Fatty Acid Beta Oxidation (C22:3->C20:3), Mitochondrial	h2o[m] + nad[m] + coa[m] + fad[m] + M00343[m] --> h[m] + nadh[m] + accoa[m] + M00012[m] + fadh2[m]	Fatty acid oxidation	0	1000	37.1 and 1892.1 and 3030.1 and 3032.1	Long chain/Very long chain acyl CoA dehydrogenase; 2-enoyl CoA hydratase; 3-hydroxylacyl CoA dehydrogenase; 3-Ketoacyl-CoA thiolase
ACADM60	Fatty Acid Beta Oxidation (C22:4->C20:4), Mitochondrial	h2o[m] + nad[m] + coa[m] + fad[m] + CE4854[m] --> h[m] + nadh[m] + accoa[m] + eicostetcoa[m] + fadh2[m]	Fatty acid oxidation	0	1000	37.1 and 1892.1 and 3030.1 and 3032.1	Long chain/Very long chain acyl CoA dehydrogenase; 2-enoyl CoA hydratase; 3-hydroxylacyl CoA dehydrogenase; 3-Ketoacyl-CoA thiolase
ACADM61	Isomerization (C12:2), Mitochondrial	3ddecdicoa[m] <==> 2ddecdicoa[m]	Fatty acid oxidation	-1000	1000	1632.1	3,2- trans- Enoyl CoA isomerase
ACADM62	Dodecenoyl Coenzyme A Isomerase	dece3coa[m] <==> dc2coa[m]	Fatty acid oxidation	-1000	1000	1632.1	3,2- trans- Enoyl CoA isomerase
ACADM63	Fatty Acid Beta Oxidation (C14:1->C12:1), Mitochondrial	h2o[m] + nad[m] + coa[m] + fad[m] + tetde5coa[m] --> h[m] + nadh[m] + accoa[m] + dd3coa[m] + fadh2[m]	Fatty acid oxidation	0	1000	37.1 and 1892.1 and 3030.1 and 3032.1	Long chain/Very long chain acyl CoA dehydrogenase; 2-enoyl CoA hydratase; 3-hydroxylacyl CoA dehydrogenase; 3-Ketoacyl-CoA thiolase
ACADM64	Isomerization (C12:3), Mitochondrial	3dodtricoa[m] <==> 2dodtricoa[m]	Fatty acid oxidation	-1000	1000	1632.1	3,2- trans- Enoyl CoA isomerase
ACADM65	Isomerization (C10:2), Mitochondrial	3decdicoa[m] <==> 2decdicoa[m]	Fatty acid oxidation	0	1000	1632.1	3,2- trans- Enoyl CoA isomerase
ACADM66	Isomerization (C16:3), Mitochondrial	3hexdtricoa[m] <==> 2hexdtricoa[m]	Fatty acid oxidation	-1000	1000	1632.1	3,2- trans- Enoyl CoA isomerase
ACADM67	Acyl-CoA Oxidase	coa[m] + h2o[m] + nad[m] + o2[m] + tetdecdicoa[m] --> 3ddecdicoa[m] + accoa[m] + h[m] + h2o2[m] + nadh[m]	Fatty acid oxidation	0	1000	51.1 and 1892.1 and 3030.1 and 3032.1	Acyl CoA oxidase; 2-enoyl CoA hydratase; 3-hydroxylacyl CoA dehydrogenase; 3-Ketoacyl-CoA thiolase
ACADM68	Isomerization of (C18:4), Mitochondrial	3octdectecoa[m] <==> 2octdectecoa[m]	Fatty acid oxidation	-1000	1000	1632.1	3,2- trans- Enoyl CoA isomerase
ACADM69	Isomerization (C22:5), Mitochondrial	3docopencoa[m] <==> 2docopencoa[m]	Fatty acid oxidation	-1000	1000	1632.1	3,2- trans- Enoyl CoA isomerase
ACADM70	Isomerization (C6:1), Mitochondrial	hexe3coa[m] <==> hx2coa[m]	Fatty acid oxidation	-1000	1000	1632.1	3,2- trans- Enoyl CoA isomerase

	ACADM71	Isomerization (C16:4), Mitochondrial	3hexdtetcoa[m] <==> 2hexdtetcoa[m]	Fatty acid oxidation	-1000	1000	1632.1	3,2- trans- Enoyl CoA isomerase
	ACADM72	Fatty Acid Beta Oxidation (C20:5->C18:5), Mitochondrial	tmndnccoa[m] + coa[m] + fad[m] + h2o[m] + nad[m] --> 3octpencoa[m] + accoa[m] + fadh2[m] + h[m] + nadh[m]	Fatty acid oxidation	0	1000	37.1 and 1892.1 and 3030.1 and 3032.1	Long chain/Very long chain acyl CoA dehydrogenase; 2-enoyl CoA hydratase; 3-hydroxylacyl CoA dehydrogenase; 3-Ketoacyl-CoA thiolase
	ACADM73	Isomerization (C18:5), Mitochondrial	3octpencoa[m] <==> 2octpencoa[m]	Fatty acid oxidation	-1000	1000	1632.1	3,2- trans- Enoyl CoA isomerase
	PXTS2	Paraxanthine Synthase Type 2, Endoplasmic reticulum	cfn1[r] + nadh[r] + h[r] + o2[r] --> pxthn1[r] + nad[r] + h2o[r] + fald[r]	Caffeine metabolism	0	1000	1544.1	
	137TMS	1,3,7-Trimethyluric acid Synthase, Endoplasmic reticulum	cfn1[r] + o2[r] + h2o[r] --> 137tmurica1[r] + h2o2[r]	Caffeine metabolism	0	1000	1544.1 or 1558.1 or 1559.1 or 1571.1 or 1576.1	
	PXTS1	Paraxanthine Synthase Type 1, Endoplasmic reticulum	cfn1[r] + nadph[r] + h[r] + o2[r] --> pxthn1[r] + nadp[r] + h2o[r] + fald[r]	Caffeine metabolism	0	1000	1544.1	
	TBMS	Theobromine Synthase, Endoplasmic reticulum	cfn1[r] + nadh[r] + h[r] + o2[r] --> theobromine1[r] + nad[r] + h2o[r] + fald[r]	Caffeine metabolism	0	1000	1544.1 or 1571.1	
	TPYS	Theophylline Synthase, Endoplasmic reticulum	cfn1[r] + nadh[r] + h[r] + o2[r] --> theophylline1[r] + nad[r] + h2o[r] + fald[r]	Caffeine metabolism	0	1000	1544.1 or 1558.1 or 1559.1 or 1571.1 or 1576.1	
	1MXO	1-Methylxanthine: NAD+ Oxidoreductase, Endoplasmic reticulum	theophylline1[r] + nadh[r] + h[r] + o2[r] --> 1mxnt[r] + nad[r] + h2o[r] + fald[r]	Caffeine metabolism	0	1000	1544.1	
	7MXO	7-Methylxanthine: NAD+ Oxidoreductase, Endoplasmic reticulum	pxthn1[r] + nadh[r] + h[r] + o2[r] --> 7mxth[r] + nad[r] + h2o[r] + fald[r]	Caffeine metabolism	0	1000	1544.1	
	17DMO	1,7-Dimethyluric acid Synthase, Endoplasmic reticulum, Endoplasmic reticulum	pxthn1[r] + o2[r] + h2o[r] --> 17dmurt1[r] + h2o2[r]	Caffeine metabolism	0	1000	1544.1 or 1548.1	
	PXTD2	Paraxanthine Demethylase, type 2, Endoplasmic reticulum	pxthn1[r] + nadh[r] + h[r] + o2[r] --> 1mxnt[r] + nad[r] + h2o[r] + fald[r]	Caffeine metabolism	0	1000	1544.1	
	PXTD1	Paraxanthine Demethylase, type 1, Endoplasmic reticulum	pxthn1[r] + nadph[r] + h[r] + o2[r] --> 1mxnt[r] + nadp[r] + h2o[r] + fald[r]	Caffeine metabolism	0	1000	1544.1	
Caffeine metabolism	1MUO	Xanthine dehydrogenase: The oxidation to 1-methyluric acid, Lysosome	1mxnt[l] + h2o[l] + o2[l] --> 1murt[l] + h2o2[l]	Caffeine metabolism	0	1000	7498.1	
	PXTAT	Paraxanthine Acetyltransferase, Cytosol	pxthn1[c] + accoa[c] --> 5acam6fam3mura[c] + coa[c]	Caffeine metabolism	0	1000	10.1	
	AAMU	Spontaneous decomposition: AFMU-AAMU, Cytosol	5acam6fam3mura[c] + h2o[c] --> 5acam6am3mura[c] + for[c] + h[c]	Caffeine metabolism	0	1000		
	37DMO	Xanthine Dehydrogenase: The oxidation to 3,7-Dimethyluric acid, Lysosome	theobromine1[l] + h2o[l] + o2[l] --> 37dmur[l] + h2o2[l]	Caffeine metabolism	0	1000	7498.1	
	7MUO	Xanthine Dehydrogenase: The oxidation to 7-methyluric acid, Lysosome	7mxth[l] + h2o[l] + o2[l] --> 7murt[l] + h2o2[l]	Caffeine metabolism	0	1000	7498.1	
	PXTtr	Paraxanthine Intracellular Transport	pxthn1[r] --> pxthn1[c]	Transport reactions, Endoplasmic reticulum	-1000	1000		
	TBMtr	Theobromine Intracellular Transport	theobromine1[r] --> theobromine1[c]	Transport reactions, Endoplasmic reticulum	-1000	1000		
	TPYtl	Theophylline Intracellular Transport	theobromine1[c] --> theobromine1[l]	Transport, lysosomal	-1000	1000		
	37DMtl	3,7-Dimethyluric acid Intracellular Transport	37dmur[l] --> 37dmur[c]	Transport, lysosomal	-1000	1000		
	37DMt	3,7-Dimethyluric acid Transport from Cytoplasm	37dmur[c] --> 37dmur[e]	Transport, extracellular	-1000	1000		
	EX_37DM[e]	Exchange of 3,7-Dimethyluric acid	37dmur[e] -->	Exchange/demand reaction	0	1000		
	17DMtr	1,7-Dimethyluric acid Intracellular Transport	17dmurt1[r] --> 17dmurt1[c]	Transport reactions, Endoplasmic reticulum	-1000	1000		
	17DMt	1,7-Dimethyluric acid Transport from Cytoplasm	17dmurt1[c] --> 17dmurt1[e]	Transport, extracellular	-1000	1000		
	EX_17DM[e]	Exchange of 1,7-Dimethyluric acid	17dmurt1[e] -->	Exchange/demand reaction	0	1000		

	1MXtr	1-Methylxanthine Intracellular Transport	1mxnt[r] --> 1mxnt[c]	Transport reactions, Endoplasmic reticulum	-1000	1000	
	1MXt	1-Methylxanthine Intracellular Transport	1mxnt[c] --> 1mxnt[l]	Transport, lysosomal	-1000	1000	
	1MUtr	1-methyluric acid Intracellular Transport	1murt[l] --> 1murt[c]	Transport, lysosomal	-1000	1000	
	1MUt	1-methyluric acid Transport from Cytoplasm	1murt[c] --> 1murt[e]	Transport, extracellular	-1000	1000	
	EX_1MU[e]	Exchange of 1-methyluric acid	1murt[e] -->	Exchange/demand reaction	0	1000	
	7MXtr	7-Methylxanthine Intracellular Transport	7mxth[r] --> 7mxth[c]	Transport reactions, Endoplasmic reticulum	-1000	1000	
	7MXt	7-Methylxanthine Intracellular Transport	7mxth[c] --> 7mxth[l]	Transport, lysosomal	-1000	1000	
	7MUtr	7-methyluric acid Intracellular Transport	7murt[l] --> 7murt[c]	Transport, lysosomal	-1000	1000	
	7MUt	7-methyluric acid Transport from Cytoplasm	7murt[c] --> 7murt[e]	Transport, extracellular	-1000	1000	
	EX_7MU[e]	Exchange of 7-methyluric acid	7murt[e] -->	Exchange/demand reaction	0	1000	
	CAFtr	Caffeine Intracellular Transport	cfn1[c] --> cfn1[r]	Transport reactions, Endoplasmic reticulum	-1000	1000	
	CAFt	Caffeine Intracellular Transport from Cytoplasm	cfn1[e] --> cfn1[c]	Transport, extracellular	-1000	1000	
	EX_CAF[e]	Exchange of Caffeine	cfn1[e] <==>	Exchange/demand reaction	-1000	1000	
	137TMtr	1,3,7-Trimethyluric acid Intracellular Transport	137tmurica1[r] --> 137tmurica1[c]	Transport reactions, Endoplasmic reticulum	-1000	1000	
	137TMt	1,3,7-Trimethyluric acid Transport from Cytoplasm	137tmurica1[c] --> 137tmurica1[e]	Transport, extracellular	-1000	1000	
	EX_137TM[e]	Exchange of 1,3,7-Trimethyluric acid	137tmurica1[e] -->	Exchange/demand reaction	0	1000	
	AAMUt	5-Acetylamino-6-amino-3-methyluracil Transport from Cytoplasm	5acam6am3mura[c] --> 5acam6am3mura[e]	Transport, extracellular	-1000	1000	
	EX_AAMU[e]	Exchange of 5-Acetylamino-6-amino-3-methyluracil	5acam6am3mura[e] -->	Exchange/demand reaction	0	1000	
	FALDtr	Formaldehyde Transport via Diffusion, Mitochondrial	fald[r] <==> fald[c]	Transport reactions, Endoplasmic reticulum	-1000	1000	
Human1 model	MAR00440		CE2414[e] --> CE2414[c]	Transport reactions	0	1000	
	MAR00494		hdcoa[c] + glyc3p[c] --> HC02030[c] + coa[c]	Glycerolipid metabolism	0	1000	57678 or 84803 or 137964 or 150763
	MAR00766		gal[e] + gluside_hs[e] --> h2o[e] + C01290[e]	Sphingolipid metabolism	0	1000	3938 or 79411 or 2720
	MAR00804		thcrm_hs[l] + h2o[l] --> gal[l] + C01290[l]	Glycosphingolipid metabolism	0	1000	3074 or 284004 or 3073
	MAR00826		gm3_hs[l] + h2o[l] --> C01290[l] + acnam[l]	Glycosphingolipid metabolism	0	1000	4759 or 10825 or 129807 or 4758
	MAR00832		HC02161[l] + h2o[l] --> gal[l] + HC02160[l]	Glycosphingolipid metabolism	0	1000	3938 and 79411 and 2720 and 643853
	MAR00838		gd1a_hs[c] + h2o[c] --> HC02161[c] + acnam[c]	Glycosphingolipid biosynthesis-ganglio series	0	1000	4759 or 10825 or 129807 or 4758
	MAR00934		arachd[c] + h[c] + nadph[c] + o2[c] --> wharachd[c] + h2o[c] + nadp[c]	Arachidonic acid metabolism	0	1000	64816 or 107987479 or 1577 or 1562 or 1571 or 1573 or 1588 or 1545 or 1559 or 1558 or 1543 or 1544 or 1580 or 113612 or 1576 or 1551 or 1557 or 29785 or 57834 or 199974 or 66002 or 260293 or 11283 or 1555 or 1572 or 1553 or 1549 or 1548 or 100861540
	MAR00941		arachd[c] + h[c] + nadph[c] + o2[c] --> MAM00591[c] + h2o[c] + nadp[c]	Arachidonic acid metabolism	0	1000	284541 or 1579

MAR00976	CE2084[c] + (2) h[c] + o2[c] --> CE7097[c] + h2o[c]	Arachidonic acid metabolism	0	1000	239
MAR01105	leuktrB4[x] + nadp[x] --> CE4990[x] + h[x] + nadph[x]	Leukotriene metabolism	0	1000	10901 or 873 or 874
MAR01152	CE5178[x] + h[x] + nadph[x] <==> CE5179[x] + nadp[x]	Leukotriene metabolism	-1000	1000	64816 or 107987479 or 1577 or 1562 or 1571 or 1573 or 1588 or 1545 or 1559 or 1558 or 1543 or 1544 or 1580 or 113612 or 1576 or 1551 or 29785 or 57834 or 199974 or 66002 or 260293 or 11283 or 1555 or 1572 or 1553 or 1549 or 1548 or 100861540
MAR01159	CE5348[r] + amp[r] + ppi[r] <==> CE5179[r] + atp[r] + coa[r]	Leukotriene metabolism	-1000	1000	2182 or 23205 or 2181 or 81616 or 11001 or 2180 or 23305 or 51703 or 728637
MAR01234	CE4987[c] + h[c] + nadph[c] + o2[c] --> CE4989[c] + h2o[c] + nadp[c]	Leukotriene metabolism	0	1000	57834 or 126410 or 8529 or 11283 or 4051
MAR01235	CE4987[r] + h[r] + nadph[r] + o2[r] --> CE4989[r] + h2o[r] + nadp[r]	Leukotriene metabolism	0	1000	57834 or 126410 or 8529 or 11283 or 4051
MAR01265	CE5969[r] + amp[r] + ppi[r] <==> CE4987[r] + atp[r] + coa[r]	Leukotriene metabolism	-1000	1000	2182 or 23205 or 2181 or 81616 or 11001 or 2180 or 23305 or 51703 or 728637
MAR01301	CE6225[x] + coa[x] + h2o[x] --> accoa[x] + (5) h[x] + CE6195[x]	Leukotriene metabolism	0	1000	30 and 4645
MAR01355	arachd[c] + o2[c] --> CE5928[c] + h[c]	Prostaglandin biosynthesis	0	1000	
MAR01358	arachd[c] + o2s[c] --> CE5535[c] + h[c]	Prostaglandin biosynthesis	0	1000	
MAR01361	arachd[c] + o2s[c] --> CE5537[c] + h[c]	Prostaglandin biosynthesis	0	1000	
MAR01364	arachd[c] + o2s[c] --> CE5925[c] + h[c]	Prostaglandin biosynthesis	0	1000	
MAR01533	chlstol[c] + h[c] + nadph[c] --> lthstrl[c] + nadp[c]	Cholesterol biosynthesis 2	0	1000	1718
MAR01570	h[c] + nadph[c] + zymst[c] --> zymstnl[c] + nadp[c]	Cholesterol metabolism	0	1000	1718
MAR01609	xoltetrol[m] + nad[m] --> thcholst[m] + h[m] + nadh[m]	Bile acid biosynthesis	0	1000	1593
MAR01613	thcholst[m] + nadh[m] + o2[m] --> thcholstoic[m] + h2o[m] + nad[m]	Bile acid biosynthesis	0	1000	1593
MAR01702	dhcholestancoa[r] + (2) atp[r] + (2) h2o[r] --> dhcholestancoa[x] + (2) adp[r] + (2) h[r] + (2) pi[r]	Transport reactions	0	1000	
MAR01729	HC00958[c] + paps[c] --> h[c] + pap[c] + HC02220[c]	Bile acid biosynthesis	0	1000	6822
MAR01803	thcholst[c] + h[c] + nadh[c] <==> M01092[c] + nad[c]	Bile acid biosynthesis	-1000	1000	1593
MAR01805	M01092[m] + h[m] + nadph[m] + o2[m] --> CE4874[m] + h2o[m] + nadp[m]	Bile acid biosynthesis	0	1000	1571 or 1593
MAR01806	CE4872[m] + h[m] + nadh[m] <==> M01092[m] + nad[m]	Bile acid biosynthesis	-1000	1000	1593
MAR01853	atp[c] + HC00958[c] + h2o[c] --> adp[c] + HC00958[e] + h[c] + pi[c]	Bile acid recycling	0	1000	1244 or 4363
MAR01854	HC00958[c] <==> HC00958[r]	Transport reactions	-1000	1000	
MAR01857	(2) atp[x] + (2) h2o[x] + tchola[x] --> (2) adp[x] + (2) h[x] + (2) pi[x] + tchola[e]	Transport reactions	0	1000	
MAR01861	(2) atp[x] + (2) h2o[x] + tdchola[x] --> (2) adp[x] + (2) h[x] + (2) pi[x] + tdchola[e]	Transport reactions	0	1000	
MAR01864	HC00958[e] + (2) na1[e] --> HC00958[c] + (2) na1[c]	Bile acid recycling	0	1000	6554 or 6555
MAR01886	HC00958[e] + HC02199[c] + hco3[c] <==> HC00958[c] + HC02199[e] + hco3[e]	Bile acid recycling	-1000	1000	11136 or 6579 or 28234

MAR01887		HC00958[e] + hco3[c] + HC02200[c] <==> HC00958[c] + hco3[e] + HC02200[e]	Bile acid recycling	-1000	1000	11136 or 6579 or 28234
MAR01888		HC00958[e] + hco3[c] + HC02201[c] <==> HC00958[c] + hco3[e] + HC02201[e]	Bile acid recycling	-1000	1000	11136 or 6579 or 28234
MAR01910		atp[r] + chsterol[r] + h2o[r] --> adp[r] + chsterol[c] + h[r] + pi[r]	Transport reactions	0	1000	
MAR01913		(2) atp[c] + (2) h2o[c] + so4[c] --> (2) adp[c] + (2) h[c] + (2) pi[c] + so4[e]	Transport reactions	0	1000	
MAR01924		chsterol[c] + M02935[c] --> M01511[c]	Steroid metabolism	0	1000	56910 or 9754 or 90627 or 6770 or 57519 or 134429 or 80765 or 147323 or 10809
MAR01926		M01511[m] --> chsterol[m] + M02935[m]	Steroid metabolism	0	1000	
MAR02001		crtsl[r] + h[r] + (2) nadph[r] + o2[r] --> C05284[r] + ac[r] + (2) h2o[r] + (2) nadp[r]	Glucocorticoid biosynthesis	0	1000	1586 or 1584 or 1585 or 83875
MAR02401		CE4830[c] + h[c] + nadph[c] --> tettet6coa[c] + nadp[c]	Omega-6 fatty acid metabolism	0	1000	9524 or 253017
MAR02411		CE4820[x] + coa[x] --> dcsptn1coa[x] + accoa[x]	Omega-6 fatty acid metabolism	0	1000	30 and 4645
MAR02449		CE5528[c] + h2o[c] --> CE6502[c] + oh1[c]	Linoleate metabolism	0	1000	
MAR02663		coa[c] + odecrn[c] <==> crn[c] + odecoa[c]	Carnitine shuttle (cytosolic)	-1000	1000	1374 or 126129 or 1375
MAR03301		lnlccoa[x] + o2[x] --> h2o2[x] + CE2434[x]	Beta oxidation of di-unsaturated fatty acids (n-6) (peroxisomal)	0	1000	8310 or 51
MAR03306		CE0849[x] + o2[x] --> h2o2[x] + CE2433[x]	Beta oxidation of di-unsaturated fatty acids (n-6) (peroxisomal)	0	1000	8310 or 51
MAR03311		CE0785[x] + o2[x] --> h2o2[x] + CE2432[x]	Beta oxidation of di-unsaturated fatty acids (n-6) (peroxisomal)	0	1000	8310 or 51
MAR03364		o2[x] + hdcoa[x] --> h2o2[x] + CE2591[x]	Beta oxidation of unsaturated fatty acids (n-9) (peroxisomal)	0	1000	8310 or 51
MAR03369		CE0784;HC10784[x] + o2[x] --> h2o2[x] + CE2594;HC12594[x]	Beta oxidation of unsaturated fatty acids (n-9) (peroxisomal)	0	1000	8310 or 51
MAR03444		arachdcoa[m] + fad[m] --> CE4795[m] + fadh2[m]	Omega-6 fatty acid metabolism	0	1000	37 or 33 or 34 or 28976
MAR03488		pristanal[x] + h2o[x] + nad[x] --> MAM00077[x] + (2) h[x] + nadh[x]	Beta oxidation of phytanic acid (peroxisomal)	0	1000	5264
MAR03489		MAM00077[x] + atp[x] + coa[x] <==> CE5125[x] + amp[x] + ppi[x]	Beta oxidation of phytanic acid (peroxisomal)	-1000	1000	2182 or 23205 or 10965 or 2181 or 81616 or 11001 or 2180 or 23305 or 122970 or 51703 or 728637
MAR03517		CE4805[c] <==> CE4805[x]	Transport reactions	-1000	1000	
MAR03554		M01489[l] + h2o[l] --> chsterol[l] + vacc[l] + h[l]	Formation and hydrolysis of cholesterol esters	0	1000	3988 or 1056
MAR03634		chsterol[r] + hdcoa[r] --> HC02021[r] + coa[r]	Formation and hydrolysis of cholesterol esters	0	1000	6646 or 8435
MAR03661		chsterol[r] + lnlncacoa[r] --> HC02025[r] + coa[r]	Formation and hydrolysis of cholesterol esters	0	1000	6646 or 8435
MAR03763		coa[m] + h[m] + HC00900[m] --> mmcoa_R[m] + h2o[m]	Valine, leucine, and isoleucine metabolism	0	1000	4329
MAR03838		1pyr5c[m] + h[m] + q10h2[m] <==> pro_L[m] + q10[m]	Arginine and proline metabolism	-1000	1000	5625 or 58510
MAR04268		atp[m] + nad[m] --> adp[m] + h[m] + nadp[m]	Nicotinate and nicotinamide metabolism	0	1000	65220 or 133686
MAR04279		atp[n] + nad[n] --> adp[n] + h[n] + nadp[n]	Nicotinate and nicotinamide metabolism	0	1000	65220 or 133686
MAR04413	Generic Human Biomass Reaction	(0.50563) ala_L[c] + (0.35926) arg_L[c] + (0.27942) asn_L[c] + (0.35261) asp_L[c] + (20.7045) atp[c] + (0.020401) chsterol[c] + (0.011658) clpn_hs[c] + (0.039036) ctp[c] + (0.046571) cys_L[c] + (0.013183) datp[n] + (0.009442) dctp[n] + (0.009898) dgtp[n] + (0.013091) dttp[n] + (0.27519) g6p[c] + (0.38587) glu_L[c] + (0.326) gln_L[c] + (0.53889) gly[c] + (0.036117) gtp[c] + (20.6508) h2o[c] + (0.12641) his_L[c] + (0.28608) ile_L[c] + (0.54554) leu_L[c] + (0.59211) lys_L[c] + (0.15302) met_L[c] + (0.15446) pchol_hs[c] +	Artificial reactions	0	0	

	(0.055374) pe_hs[c] + (0.25947) phe_L[c] + (0.023315) pail_hs[c] + (0.41248) pro_L[c] + (0.005829) ps_hs[c] + (0.39253) ser_L[c] + (0.017486) sphmyln_hs[c] + (0.31269) thr_L[c] + (0.013306) trp_L[c] + (0.15967) tyr_L[c] + (0.053446) utp[c] + (0.35261) val_L[c] + (0.002914) pglyc_hs[c] --> (20.6508) adp[c] + (20.6508) h[c] + (20.6508) pi[c] + Temp001[c]				
MAR04548	6hoxmelatn[c] + h[c] <==> oh1[c] + melatn[c]	Serotonin and melatonin biosynthesis	-1000	1000	1545 or 1543 or 1544
MAR04552	oh1[c] + melatn[c] <==> CE5982[c]	Serotonin and melatonin biosynthesis	-1000	1000	
MAR04553	CE2119[c] + h2o[c] <==> oh1[c] + CE5982[c]	Serotonin and melatonin biosynthesis	-1000	1000	
MAR04755	(3) o2[m] + pppg9[m] --> (3) h2o2[m] + ppp9[m]	Porphyrin metabolism	0	1000	5498
MAR04861	inost[c] <==> inost[e]	Transport reactions	-1000	1000	114134
MAR04911	(3) na1[e] + (2) pi[e] --> (3) na1[c] + (2) pi[c]	Transport reactions	0	1000	6574 or 6575
MAR05015	bhb[m] + h[i] --> bhb[c] + h[m]	Transport reactions	0	1000	
MAR05043	pi[c] + h[i] --> h[m] + pi[m]	Transport reactions	0	1000	5250
MAR05053	gly[l] --> gly[c]	Transport reactions	0	1000	206358
MAR05059	pro_L[l] --> pro_L[c]	Transport reactions	0	1000	206358
MAR05064	ala_L[l] --> ala_L[c]	Transport reactions	0	1000	206358
MAR05076	gly[e] --> gly[c]	Transport reactions	0	1000	(23428 and 6520) or 6542 or 8140 or 206358 or 56301 or 6541 or 8501 or 9056 or 348932 or 84889 or 124935
MAR05102	gln_L[m] + h[i] --> gln_L[c] + h[m]	Transport reactions	0	1000	
MAR05107	ile_L[c] + h[i] --> h[m] + ile_L[m]	Transport reactions	0	1000	
MAR05109	leu_L[c] + h[i] --> h[m] + leu_L[m]	Transport reactions	0	1000	
MAR05118	val_L[c] + h[i] --> h[m] + val_L[m]	Transport reactions	0	1000	
MAR05125	pro_L[c] + h[i] --> h[m] + pro_L[m]	Transport reactions	0	1000	
MAR05126	pro_L[m] + h[i] --> h[m] + pro_L[c]	Transport reactions	0	1000	
MAR05127	nh4[c] <==> h[c] + MAM02578[c]	Miscellaneous	-1000	1000	
MAR05128	nh4[e] <==> h[e] + MAM02578[e]	Miscellaneous	-1000	1000	
MAR05292	ac[c] <==> ac[m]	Transport reactions	-1000	1000	387700 or 220963 or 201232
MAR05342	h[c] + pi[c] <==> h[r] + pi[r]	Transport reactions	-1000	1000	ENSG00000104888 or 10786
MAR05429	atp[c] + h2o[c] + (2) k[e] + (3) na1[c] --> adp[c] + h[c] + (2) k[c] + (3) na1[e] + pi[c]	Transport reactions	0	1000	(23439 and 486 and 476) or (477 and 482 and 486) or (477 and 483 and 486) or (23439 and 478 and 486) or (478 and 486 and 481) or (478 and 482 and 486) or (483 and 478 and 486) or (23439 and 480 and 486) or (480 and 486 and 481) or (482 and 480 and 486) or (483 and 480 and 486) or (486 and 481

					and 476) or (482 and 486 and 476) or (483 and 486 and 476) or (477 and 23439 and 486) or (477 and 486 and 481)
MAR06059	h[e] + thm[e] --> h[c] + thm[c]	Transport reactions	0	1000	10560 or 80704
MAR06142	MAM00591[c] + HC02199[e] + hco3[e] <==> MAM00591[e] + HC02199[c] + hco3[c]	Transport reactions	-1000	1000	10599
MAR06143	MAM00591[c] + hco3[e] + HC02200[e] <==> MAM00591[e] + hco3[c] + HC02200[c]	Transport reactions	-1000	1000	10599
MAR06144	MAM00591[c] + hco3[e] + HC02201[e] <==> MAM00591[e] + hco3[c] + HC02201[c]	Transport reactions	-1000	1000	10599
MAR06220	MAM00591[c] + gthrd[e] + hco3[e] <==> MAM00591[e] + gthrd[c] + hco3[c]	Transport reactions	-1000	1000	6578
MAR06252	MAM00591[c] <==> MAM00591[e]	Transport reactions	-1000	1000	
MAR06323	arg_L[c] + h[i] --> arg_L[m] + h[m]	Transport reactions	0	1000	10166 or 83884
MAR06327	citr_L[m] + h[i] --> citr_L[c] + h[m]	Transport reactions	0	1000	10166 or 83884
MAR06381	h[c] + na1[e] + ser_L[e] --> h[e] + na1[c] + ser_L[c]	Transport reactions	0	1000	92745 or 81539 or 54407 or 55089 or 11254
MAR06382	asn_L[e] + h[c] + na1[e] --> asn_L[c] + h[e] + na1[c]	Transport reactions	0	1000	92745 or 81539 or 54407 or 55089 or 11254
MAR06383	h[c] + hom_L[e] + na1[e] --> h[e] + hom_L[c] + na1[c]	Transport reactions	0	1000	6542 or 6541 or 84889
MAR06393	oh1[c] + C01041[c] --> dhdascb[c] + h2o[c]	Ascorbate and aldarate metabolism	0	1000	
MAR06406	atp[c] + h2o[c] + lipoate[e] + (2) na1[e] --> adp[c] + h[c] + lipoate[c] + (2) na1[c] + pi[c]	Transport reactions	0	1000	8884
MAR06448	CE5846[m] + atp[m] + coa[m] + (2) h2o[m] + nad[m] + o2[m] --> CE5847[m] + accoa[m] + amp[m] + (2) h[m] + h2o2[m] + nadh[m] + ppi[m]	Vitamin E metabolism	0	1000	30 or 3028 or 3030 or 8310 or 11332 or 10005 or 1666 or 10965 or 1892 or 376497 or 3295 or 570 or 3032 or 3033 or 11001 or 11000 or 132949 or 51 or 80221 or 10999 or 10449 or 1632 or 197322 or 122970 or 641371 or 10455 or 23600 or 26063
MAR06457	CE7101[c] <==> CE7047[c] + oh1[c]	Vitamin E metabolism	-1000	1000	
MAR06587	CE5101;pail5p_hs[c] + atp[c] --> pail35p_hs[c] + adp[c] + h[c]	Inositol phosphate metabolism	0	1000	5289
MAR06589	CE5101;pail5p_hs[r] + atp[r] --> pail35p_hs[r] + adp[r] + h[r]	Inositol phosphate metabolism	0	1000	5289
MAR06608	oh1[c] <==> MAM02149[c]	ROS detoxification	-1000	1000	
MAR06680	13_cis_retn[r] + udpglcur[r] --> retnglc[r] + udp[r]	Retinol metabolism	0	1000	7365 or 79799 or 54490 or 133688 or 7363 or 54578 or 167127 or 7364 or 10941 or 7366 or 7367 or 10720 or 54579 or 54600 or 54658 or 54576 or 54575 or 54659 or 54577 or ENSG00000244474
MAR06685	CE2963[c] + udpglcur[c] --> CE2958[c] + udp[c]	Retinol metabolism	0	1000	7365 or 79799 or 54490 or 133688 or 7363 or 54578 or 167127 or 7364 or 10941 or 7366 or 7367 or 10720 or 54579 or 54600 or 54658 or 54576 or 54575 or 54659 or 54577 or ENSG00000244474

MAR06767	3mox4hpac[c] + h2o[c] + nadp[c] --> (2) h[c] + homoval[c] + nadph[c]	Phenylalanine, tyrosine and tryptophan biosynthesis	0	1000	221 or 218 or 222 or 220
MAR06783	34hpl[c] --> T4hcinnm[c] + h2o[c]	Phenylalanine, tyrosine and tryptophan biosynthesis	0	1000	
MAR06974	M00196;protein[c] + lys_L[c] --> peplys[c] + h2o[c]	Lysine metabolism	0	1000	
MAR07282	M00196;protein[c] + asn_L[c] --> Asn_X_Ser_Thr[c] + h2o[c]	Protein modification	0	1000	
MAR07301	g3m8masn[g] + (4) h2o[g] --> m7masnA[g] + (3) glc_D[g] + man[g]	N-glycan metabolism	0	1000	
MAR07302	g2m8masn[g] + (3) h2o[g] --> m7masnA[g] + (2) glc_D[g] + man[g]	N-glycan metabolism	0	1000	
MAR07303	g1m8masn[g] + (2) h2o[g] --> m7masnA[g] + glc_D[g] + man[g]	N-glycan metabolism	0	1000	
MAR07304	g1m7masnC[g] + (2) h2o[g] --> m6masnC[g] + glc_D[g] + man[g]	N-glycan metabolism	0	1000	
MAR07305	g1m7masnB[g] + (2) h2o[g] --> m6masnB2[g] + glc_D[g] + man[g]	N-glycan metabolism	0	1000	
MAR07306	g1m6masnB1[g] + (2) h2o[g] --> m5masnB1[g] + glc_D[g] + man[g]	N-glycan metabolism	0	1000	
MAR07327	n2m2masn[g] + uacgam[g] --> n5m2masn[g] + h[g] + udp[g]	N-glycan metabolism	0	1000	11320 or 11282 or 25834
MAR07436	Ser_Gly_Ala_X_Gly[g] + udpacgal[g] --> h[g] + Tn_antigen[g] + udp[g]	O-glycan metabolism	0	1000	57452 or 168391 or 51809 or 374378 or 2591 or 79695 or 26290 or 117248 or 11227 or 11226 or 2589 or 2590 or 114805 or 79623 or 55568 or 442117 or 63917 or 50614 or 64409 or 8693 or 100528030
MAR07603	h2o[c] + dak2gpe_hs[c] --> M00223[c] + h[c] + Rtotal[c]	Ether lipid metabolism	0	1000	8399 or 50487 or 8605 or 5321 or 26279 or 81579 or 5322 or 84647 or 64600 or 283748 or 151056 or 255189 or 8681 or 5319 or 11145 or 8398 or 391013 or 123745 or 5320 or 30814 or 100137049
MAR07629	atp[c] + (2) ca2[c] + h2o[c] --> adp[c] + (2) ca2[e] + h[c] + pi[c]	Transport reactions	0	1000	27032 or 493 or 9914 or 492 or 490 or 489 or 491 or 488 or 487
MAR07633	(2) atp[c] + (2) h2o[c] + (3) hista[c] --> (2) adp[c] + (2) h[c] + (3) hista[e] + (2) pi[c]	Transport reactions	0	1000	6570 or 6571
MAR07634	(3) 5htrp[c] + (2) atp[c] + (2) h2o[c] --> (3) 5htrp[e] + (2) adp[c] + (2) h[c] + (2) pi[c]	Transport reactions	0	1000	6570 or 6571
MAR07635	(3) adrnl[c] + (2) atp[c] + (2) h2o[c] --> (2) adp[c] + (3) adrnl[e] + (2) h[c] + (2) pi[c]	Transport reactions	0	1000	6570 or 6571
MAR07636	(2) atp[c] + (3) dopa[c] + (2) h2o[c] --> (2) adp[c] + (3) dopa[e] + (2) h[c] + (2) pi[c]	Transport reactions	0	1000	6570 or 6571
MAR07637	(2) atp[c] + (2) h2o[c] + (3) nrpphr[c] --> (2) adp[c] + (2) h[c] + (3) nrpphr[e] + (2) pi[c]	Transport reactions	0	1000	6570 or 6571
MAR07672	apoC_Lys_btn[c] + atp[c] + hco3[c] --> adp[c] + btn_co2[c] + h[c] + pi[c]	Biotin metabolism	0	1000	32 or 31
MAR07673	accoa[c] + btn_co2[c] --> apoC_Lys_btn[c] + malcoa[c]	Biotin metabolism	0	1000	32 or 31
MAR07681	(3) ach[c] + (2) atp[c] + (2) h2o[c] --> (3) ach[e] + (2) adp[c] + (2) h[c] + (2) pi[c]	Transport reactions	0	1000	6572
MAR07682	5fthf[c] + oh1[e] <==> 5fthf[e] + oh1[c]	Transport reactions	-1000	1000	6573
MAR07685	oh1[e] + thf[c] <==> oh1[c] + thf[e]	Transport reactions	-1000	1000	6573
MAR07686	oh1[c] + thmmp[e] <==> oh1[e] + thmmp[c]	Transport reactions	-1000	1000	6573
MAR07687	(3) oh1[e] + thmtp[c] --> (3) oh1[c] + thmtp[e]	Transport reactions	0	1000	6573

MAR07736	atp[c] + (3) ala_B[c] + h2o[c] --> adp[c] + (3) ala_B[e] + h[c] + pi[c]	Transport reactions	0	1000	140679
MAR07737	atp[c] + (3) gly[c] + h2o[c] --> adp[c] + (3) gly[e] + h[c] + pi[c]	Transport reactions	0	1000	140679
MAR07738	(3) 4abut[c] + atp[c] + h2o[c] --> (3) 4abut[e] + adp[c] + h[c] + pi[c]	Transport reactions	0	1000	140679
MAR07774	nad[c] --> nad[e]	Transport reactions	0	1000	
MAR07781	adp[c] --> adp[e]	Transport reactions	0	1000	
MAR07787	ctp[c] --> ctp[e]	Transport reactions	0	1000	
MAR07799	atp[c] + (3) h[c] + h2o[c] --> adp[c] + (4) h[l] + pi[c]	Transport reactions	0	1000	535 or 51606 or 537 or 50617 or 10312 or 533 or 245972 or 9114 or 23545 or 527
MAR07904	na1[e] + sel[e] --> na1[c] + sel[c]	Transport reactions	0	1000	6561
MAR08024	peplys[c] <==> peplys[n]	Transport reactions	-1000	1000	
MAR08064	(2) 3sala[m] + (2) h[m] + o2[m] --> (2) Lcyst[m]	Bile acid biosynthesis	0	1000	
MAR08145	C01290[c] <==> C01290[g]	Transport reactions	-1000	1000	
MAR08165	C01290[g] + udpacgal[g] --> ga2_hs[g] + h[g] + udp[g]	Sphingolipid metabolism	0	1000	2583
MAR08202	C01290[c] <==> C01290[l]	Transport reactions	-1000	1000	
MAR08245	h2o[c] + sphmyln_hs[c] --> spc_hs[c] + Rtotal[c]	Sphingolipid metabolism	0	1000	
MAR08279	acglcgal14acglcgalgluside_hs[g] + udpgal[g] --> h[g] + galacglcgal14acglcgalgluside_hs[c] + udp[g]	Blood group biosynthesis	0	1000	10331
MAR08317	acglcgalacglcgal14acglcgalgluside_hs[g] + udpgal[g] --> h[c] + M02186[c] + udp[g]	Blood group biosynthesis	0	1000	10331
MAR08406	pre_prot[r] + m3gacpail_hs[r] --> gpi_sig[r] + m3gacpail_prot_hs[r]	Glycosylphosphatidylinositol (GPI)-anchor biosynthesis	0	1000	94005 and 128869 and 51604 and 10026 and 8733
MAR08522	ak2gchol_hs[c] + h2o[c] --> ak2lgchol_hs[c] + h[c] + Rtotal[c]	Glycerophospholipid metabolism	0	1000	
MAR08561	leuktrB4woh[r] + o2[r] --> leuktrB4wcooh[r] + h[r] + h2o[r]	Eicosanoid metabolism	0	1000	
MAR08615	cbl2[m] + nadh[m] <==> cbl1[m] + h[m] + nad[m]	Vitamin B12 metabolism	-1000	1000	326625 or 166785
MAR08745	oh1[c] + thmmp[m] <==> oh1[m] + thmmp[c]	Transport reactions	-1000	1000	
MAR08747	(2) oh1[m] + thmpp[c] --> (2) oh1[c] + thmpp[m]	Transport reactions	0	1000	
MAR08782	h[c] + hpyr[c] + nadh[c] --> glyc_R[c] + nad[c]	Tricarboxylic acid cycle and glyoxylate/dicarboxylate metabolism	0	1000	9380
MAR08913	lcts[g] --> lcts[c]	Transport reactions	0	1000	
MAR08922	(2) atp[c] + (2) h2o[c] + (3) mepi[c] --> (2) adp[c] + (2) h[c] + (3) mepi[e] + (2) pi[c]	Transport reactions	0	1000	
MAR09037	CE2414[e] <==>	Exchange/demand reaction	-1000	1000	
MAR09204	pre_prot[e] <==>	Exchange/demand reaction	-1000	1000	
MAR09450	xtp[e] <==>	Exchange/demand reaction	-1000	1000	
MAR09456	adpglc[e] <==>	Exchange/demand reaction	-1000	1000	
MAR09704	dgpi_prot_hs[e] <==>	Exchange/demand reaction	-1000	1000	
MAR09705	dem2emgacpail_prot_hs[e] <==>	Exchange/demand reaction	-1000	1000	
MAR09729	M03161[e] <==>	Exchange/demand reaction	-1000	1000	

MAR09931	Biomass maintenance reaction without replication precursors	(0.50563) ala_L[c] + (0.35926) arg_L[c] + (0.27942) asn_L[c] + (0.35261) asp_L[c] + (20.7045) atp[c] + (0.020401) chsterol[c] + (0.011658) clpn_hs[c] + (0.039036) ctp[c] + (0.046571) cys_L[c] + (0.27519) g6p[c] + (0.38587) glu_L[c] + (0.326) gln_L[c] + (0.53889) gly[c] + (0.036117) gtp[c] + (20.6508) h2o[c] + (0.12641) his_L[c] + (0.28608) ile_L[c] + (0.54554) leu_L[c] + (0.59211) lys_L[c] + (0.15302) met_L[c] + (0.15446) pchol_hs[c] + (0.055374) pe_hs[c] + (0.25947) phe_L[c] + (0.023315) pail_hs[c] + (0.41248) pro_L[c] + (0.005829) ps_hs[c] + (0.39253) ser_L[c] + (0.017486) sphmyln_hs[c] + (0.31269) thr_L[c] + (0.013306) trp_L[c] + (0.15967) tyr_L[c] + (0.053446) utp[c] + (0.35261) val_L[c] + (0.002914) pglyc_hs[c] --> (20.6508) adp[c] + (20.6508) h[c] + (20.6508) pi[c] + Temp001[c]	Artificial reactions	0	0
MAR09932	Biomass maintenance reaction without replication, transcription, and translation precursors	(20.7045) atp[c] + (0.020401) chsterol[c] + (0.011658) clpn_hs[c] + (0.27519) g6p[c] + (20.6508) h2o[c] + (0.15446) pchol_hs[c] + (0.055374) pe_hs[c] + (0.023315) pail_hs[c] + (0.005829) ps_hs[c] + (0.017486) sphmyln_hs[c] + (0.002914) pglyc_hs[c] --> (20.6508) adp[c] + (20.6508) h[c] + (20.6508) pi[c] + Temp001[c]	Artificial reactions	0	0
MAR10023		Temp001[c] --> Temp001[e]	Transport reactions	0	1000
MAR10024		Temp001[e] <==>	Exchange/demand reaction	-1000	1000
MAR10025	Exchange of 20-hydroxy-arachidonate	MAM00591[e] <==>	Exchange/demand reaction	-1000	1000
MAR10026	Exchange of chenodiol	HC00958[e] <==>	Exchange/demand reaction	-1000	1000
MAR10027	Exchange of LacCer pool	C01290[e] <==>	Exchange/demand reaction	-1000	1000
MAR10029	Exchange of steroids	MAM10001[e] <==>	Exchange/demand reaction	-1000	1000
MAR10030	Exchange of xenobiotics	MAM10002[e] <==>	Exchange/demand reaction	-1000	1000
MAR10031	Exchange of arachidonate derivatives	MAM10003[e] <==>	Exchange/demand reaction	-1000	1000
MAR10033	Fatty acid pool formation and breakdown	Rtotal[c] <==> (0.0004) M00003[c] + (0.0004) M00008[c] + (0.0004) M00010[c] + (0.0004) M00017[c] + (0.0004) M00019[c] + (0.0004) M00021[c] + (0.0038) dcsptn1[c] + (0.0004) lneldc[c] + (0.0004) tetpent6[c] + (0.0004) tethex3[c] + (0.0004) M00115[c] + (0.0004) M00117[c] + (0.0004) ttdcea[c] + (0.0004) tettet6[c] + (0.0004) tetpent3[c] + (0.0004) M00260[c] + (0.0004) M00265[c] + (0.0004) M00315[c] + (0.0004) M00341[c] + (0.0004) M01197[c] + (0.0004) M01207[c] + (0.0004) M01235[c] + (0.0004) M01238[c] + (0.0014) adrn[c] + (0.1083) arachd[c] + (0.0004) docosac[c] + (0.0004) hexc[c] + (0.0004) M01582[c] + (0.0004) doco13ac[c] + (0.0004) CE2510[c] + (0.025) vacc[c] + (0.0278) crvnc[c] + (0.0215) dlnlcg[c] + (0.0059) clpnd[c] + (0.0004) arach[c] + (0.0004) elaid[c] + (0.0116) tmndnc[c] + (0.0029) lnlncg[c] + (0.0004) M02053[c] + (0.0004) ddca[c] + (0.0004) lgnc[c] + (0.1915) lnlc[c] + (0.0084) lnlnca[c] + (0.0004) hpdca[c] + (0.0004) M02457[c] + (0.0133) ttdca[c] + (0.0004) nrvnc[c] + (0.0004) M02613[c] + (0.1545) ocdcea[c] + (0.0115) eicostet[c] + (0.222) hdca[c] + (0.0219) hdcea[c] + (0.0004) ptdca[c] + (0.0004) M02745[c] + (0.1498) ocdca[c] + (0.0025) strdnc[c] + (0.0004) M03045[c] + (0.0004) M03051[c] + (0.0004) M03153[c]	Pool reactions	-1000	1000
MAR10034	Fatty acid pool formation and breakdown	Rtotal[r] <==> (0.0004) M00003[r] + (0.0004) M00008[r] + (0.0004) M00010[r] + (0.0004) M00017[r] + (0.0004) M00019[r] + (0.0004) M00021[r] + (0.0038) dcsptn1[r] + (0.0004) lneldc[r] + (0.0004) tetpent6[r] + (0.0004) tethex3[r] + (0.0004) M00115[r] + (0.0004) M00117[r] + (0.0004) ttdcea[r] + (0.0004) tettet6[r] + (0.0004) tetpent3[r] + (0.0004) M00260[r] + (0.0004) M00265[r] + (0.0004) M00315[r] + (0.0004) M00341[r] + (0.0004) M01197[r] + (0.0004) M01207[r] + (0.0004) M01235[r] + (0.0004) M01238[r] + (0.0014) adrn[r] + (0.1083) arachd[r] + (0.0004) docosac[r] + (0.0004) hexc[r] + (0.0004) M01582[r] + (0.0004) doco13ac[r] + (0.0004) CE2510[r] + (0.025) vacc[r] + (0.0278) crvnc[r] + (0.0215) dlnlcg[r] + (0.0059) clpnd[r] + (0.0004) arach[r] + (0.0004) elaid[r] + (0.0116) tmndnc[r] + (0.0029) lnlncg[r] + (0.0004) M02053[r] + (0.0004) ddca[r] + (0.0004) lgnc[r] + (0.1915) lnlc[r] + (0.0084) lnlnca[r] + (0.0004) hpdca[r] + (0.0004) M02457[r] + (0.0133) ttdca[r] + (0.0004) nrvnc[r] + (0.0004) M02613[r] + (0.1545) ocdcea[r] + (0.0115) eicostet[r] + (0.222)	Pool reactions	-1000	1000

		hdca[r] + (0.0219) hdcea[r] + (0.0004) ptdca[r] + (0.0004) M02745[r] + (0.1498) ocdca[r] + (0.0025) strdnc[r] + (0.0004) M03045[r] + (0.0004) M03051[r] + (0.0004) M03153[r]			
MAR10035	1-acylglycerol-3-phosphate pool formation and breakdown	MAM10006[c] <==> (0.0004) M00436[c] + (0.0004) M00437[c] + (0.0004) M00438[c] + (0.0004) M00439[c] + (0.0004) M00440[c] + (0.0004) M00441[c] + (0.0004) M00442[c] + (0.0004) M00443[c] + (0.0004) M00444[c] + (0.0004) M00445[c] + (0.0004) M00446[c] + (0.0004) M00447[c] + (0.0004) M00448[c] + (0.0004) M00449[c] + (0.0278) M00450[c] + (0.0038) M00451[c] + (0.0116) M00452[c] + (0.0004) M00453[c] + (0.0004) M00454[c] + (0.0004) M00455[c] + (0.0004) M00456[c] + (0.0025) M00457[c] + (0.0004) M00458[c] + (0.0059) M00459[c] + (0.0014) M00460[c] + (0.0004) M00461[c] + (0.0004) M00462[c] + (0.0004) M00463[c] + (0.0115) M00464[c] + (0.0004) M00465[c] + (0.0004) M00466[c] + (0.0004) M00467[c] + (0.0004) M00468[c] + (0.0004) M00469[c] + (0.0004) M00470[c] + (0.0004) M00471[c] + (0.1083) HC02036[c] + (0.025) M00474[c] + (0.0215) M00476[c] + (0.0004) M00477[c] + (0.0004) M00478[c] + (0.0029) M00479[c] + (0.0004) M00480[c] + (0.0004) M00481[c] + (0.0004) M00482[c] + (0.0004) M00483[c] + (0.0004) M00484[c] + (0.1915) HC02033[c] + (0.0084) M00492[c] + (0.0133) M00493[c] + (0.0004) M00494[c] + (0.1545) HC02032[c] + (0.222) HC02029[c] + (0.0219) HC02030[c] + (0.0004) M00498[c] + (0.1498) HC02031[c] + (0.0004) M00500[c] + (0.0004) M00501[c] + (0.0004) M00502[c]	Pool reactions	-1000	1000
MAR10036	acyl-CoA pool formation and breakdown	MAM10007[c] <==> (0.0004) M00004[c] + (0.0004) M00006[c] + (0.0004) CE5151[c] + (0.0004) CE4843[c] + (0.0004) M00012[c] + (0.0004) CE5155[c] + (0.0004) M00018[c] + (0.0004) M00020[c] + (0.0004) M00023[c] + (0.0004) nrvnccoa[c] + (0.0038) dcsptn1coa[c] + (0.0278) CE4827;c226coa[c] + (0.0004) M00101[c] + (0.0116) tmndnccoa[c] + (0.0004) lneldccoa[c] + (0.0025) strdnccoa[c] + (0.0004) tetpent6coa[c] + (0.0004) tethex3coa[c] + (0.0004) M00116[c] + (0.0004) CE0784;HC10784[c] + (0.0014) adrncoa[c] + (0.0059) clpndcoa[c] + (0.0004) M00123[c] + (0.0115) eicostetcoa[c] + (0.0004) M00127[c] + (0.0004) M00129[c] + (0.0004) tettet6coa[c] + (0.0004) tetpent3coa[c] + (0.0004) CE4854[c] + (0.0004) CE4847[c] + (0.0004) CE4855[c] + (0.0004) M00343[c] + (0.0004) M01141;tetde5coa[c] + (0.0004) M01191;hexde7coa[c] + (0.0004) M01236[c] + (0.0004) M01237[c] + (0.1083) arachdcoa[c] + (0.025) vacccoa[c] + (0.0215) dlnlcgcoa[c] + (0.0004) docoscoa[c] + (0.0004) arachcoa[c] + (0.0029) lnlncgcoa[c] + (0.0004) M02052[c] + (0.0004) hpdcacoa[c] + (0.0004) hexccoa[c] + (0.0004) M02112[c] + (0.0004) ddcacoa[c] + (0.0084) lnlncacoa[c] + (0.1915) lnlccoa[c] + (0.0133) tdcoa[c] + (0.0004) M02612[c] + (0.1545) odecoa[c] + (0.0219) hdcoa[c] + (0.222) pmtcoa[c] + (0.0004) ptdcacoa[c] + (0.1498) stcoa[c] + (0.0004) ttccoa[c] + (0.0004) M03047[c] + (0.0004) M03050;tridcoa[c]	Pool reactions	-1000	1000
MAR10037	cholesterol-ester pool formation and breakdown	xolest2_hs[l] <==> (0.0004) M01452[l] + (0.0004) M01453[l] + (0.0004) M01454[l] + (0.0004) M01455[l] + (0.0004) M01456[l] + (0.0004) M01457[l] + (0.0004) M01458[l] + (0.0004) M01459[l] + (0.0004) M01460[l] + (0.0004) M01461[l] + (0.0004) M01462[l] + (0.0004) M01463[l] + (0.0004) M01464[l] + (0.0004) M01465[l] + (0.0278) M01466[l] + (0.0038) M01467[l] + (0.0116) M01468[l] + (0.0004) M01469[l] + (0.0004) M01470[l] + (0.0004) M01471[l] + (0.0004) M01472[l] + (0.0025) M01473[l] + (0.0004) M01474[l] + (0.0059) M01475[l] + (0.0014) M01476[l] + (0.0004) M01477[l] + (0.0004) M01478[l] + (0.0004) M01479[l] + (0.0115) M01480[l] + (0.0004) M01481[l] + (0.0004) M01482[l] + (0.0004) M01483[l] + (0.0004) M01484[l] + (0.0004) M01485[l] + (0.0004) M01486[l] + (0.0004) M01487[l] + (0.1083) HC02027[l] + (0.025) M01489[l] + (0.0215) M01490[l] + (0.0004) M01491[l] + (0.0004) M01492[l] + (0.0029) HC02026[l] + (0.0004) M01494[l] + (0.0004) M01495[l] + (0.0004) M01496[l] + (0.0004) M01497[l] + (0.0004) M01498[l] + (0.1915) HC02024[l] + (0.0084) HC02025[l] + (0.0133) M01501[l] + (0.0004) M01502[l] + (0.1545) HC02023[l] + (0.222) HC02020[l] + (0.0219) HC02021[l] + (0.0004) M01506[l] + (0.1498) HC02022[l] + (0.0004) M01508[l] + (0.0004) M01509[l] + (0.0004) M01510[l]	Pool reactions	-1000	1000
MAR10039	fatty acid pool transport (Cytosol to Peroxisome)	Rtotal[c] <==> Rtotal[x]	Transport reactions	-1000	1000
MAR10040	acyl-CoA pool transport (Cytosol to Peroxisome)	MAM10007[c] <==> MAM10007[x]	Transport reactions	-1000	1000

MAR10041	TAG-VLDL pool transport (Cytosol to Endoplasmic reticulum)	tag_hs[c] <==> tag_hs[r]	Transport reactions	-1000	1000	
MAR10042	TAG-chylomicron pool transport (Cytosol to Extracellular)	M02956[c] --> M02956[e]	Transport reactions	0	1000	
MAR10043	TAG-chylomicron pool transport (Cytosol to Lysosome)	M02956[c] <==> M02956[l]	Transport reactions	-1000	1000	
MAR10044	1,2-diacylglycerol-LD-PC pool transport(Cytosol to Golgi apparatus)	HC02056[c] <==> HC02056[g]	Transport reactions	-1000	1000	
MAR10045	1,2-diacylglycerol-LD-PC pool transport(Cytosol to Nucleus)	HC02056[c] <==> HC02056[n]	Transport reactions	-1000	1000	
MAR10046	1,2-diacylglycerol-LD-PC pool transport(Cytosol to Extracellular)	HC02056[c] --> HC02056[e]	Transport reactions	0	1000	
MAR10047	1,2-diacylglycerol-LD-PC pool exchange	HC02056[e] <==>	Exchange/demand reaction	-1000	1000	
MAR10048	1,2-diacylglycerol-LD-PI pool transport(Cytosol to Extracellular)	HC02059[c] <==> HC02059[n]	Transport reactions	-1000	1000	
MAR10049	CDP-diacylglycerol-CL pool transport(Cytosol to Mitochondria)	HC02094[c] <==> HC02094[m]	Transport reactions	-1000	1000	
MAR10050	fatty acid-chylomicron pool transport(Cytosol to Extracellular)	M01807[c] --> M01807[e]	Transport reactions	0	1000	
MAR10051	fatty acid-VLDL pool transport(Cytosol to Extracellular)	M01820[c] --> M01820[e]	Transport reactions	0	1000	
MAR10052	phosphatidate-LD-PC pool transport(Cytosol to Mitochondria)	HC02050[c] <==> HC02050[m]	Transport reactions	-1000	1000	
MAR10053	phosphatidate-LD-PI pool transport(Cytosol to Mitochondria)	HC02053[c] <==> HC02053[m]	Transport reactions	-1000	1000	
MAR10054	phosphatidate-LD-PI pool transport(Cytosol to Endoplasmic reticulum)	HC02053[c] <==> HC02053[r]	Transport reactions	-1000	1000	
MAR10055	phosphatidate-LD-PI pool transport (Cytosol to Golgi apparatus)	HC02053[c] <==> HC02053[g]	Transport reactions	-1000	1000	
MAR10056	phosphatidate-LD-PS pool transport (Cytosol to Endoplasmic reticulum)	HC02052[c] <==> HC02052[r]	Transport reactions	-1000	1000	
MAR10057	phosphatidate-LD-PS pool transport (Cytosol to Golgi apparatus)	HC02052[c] <==> HC02052[g]	Transport reactions	-1000	1000	
MAR10058	protein transport (Cytosol to Endoplasmic reticulum)	M00196;protein[c] <==> M00196;protein[r]	Transport reactions	-1000	1000	
MAR10059	N-formyl-L-glutamate transport (Cytosol to Extracellular)	nforglu[c] --> nforglu[e]	Transport reactions	0	1000	
MAR10060	N-formyl-L-glutamate exchange	nforglu[e] <==>	Exchange/demand reaction	-1000	1000	
MAR10061	fatty acyl-CoA reduction	(2) h[x] + (2) nadph[x] + MAM10007[x] --> coa[x] + (2) nadp[x] + alkylR1oh[x]	Ether lipid metabolism	0	1000	55711 or 84188
MAR10062	Protein pool for biomass reaction	(0.0721) M02006[c] + (0.0801) M02335[c] + (0.0512) M02340[c] + (0.0375) M02341[c] + (0.0556) M02342[c] + (0.0183) M02351[c] + (0.0428) M02376[c] + (0.0783) M02377[c] + (0.0228) M02380[c] + (0.0442) M02401[c] + (0.0911) M02404[c] + (0.0719) M02405[c] + (0.0222) M02408[c] + (0.0368) M02412[c] + (0.051) M02415[c] + (0.0661) M02416[c] + (0.0535) M02419[c] + (0.0098) M02420[c] + (0.0281) M02421[c] + (0.0667) M02423[c] --> (0.0801) M03063[c] + (0.0512) M03064[c] + (0.0375) M03065[c] + (0.0556) M03066[c] + (0.0183) M03067[c] + (0.0428) M03068[c] + (0.0783) M03069[c] + (0.0721) M03070[c] + (0.0228) M03071[c] + (0.0442) M03072[c] + (0.0911) M03073[c] + (0.0719) M03074[c] + (0.0222) M03075[c] + (0.0368) M03076[c] + (0.051) M03077[c] + (0.0661) M03078[c] + (0.0535) M03079[c] + (0.0098) M03080[c] + (0.0281) M03081[c] + (0.0667) M03082[c] + MAM10013[c]	Artificial reactions	0	1000	
MAR10063	Lipid pool for biomass reaction	(0.1155) chsterol[c] + (0.0115) xolest2_hs[c] + (0.0205) clpn_hs[c] + (0.5029) pchol_hs[c] + (0.1905) pe_hs[c] + (0.0692) pail_hs[c] + (0.019) ps_hs[c] + (0.0613) sphmyln_hs[c] + (0.0096) pglyc_hs[c] --> MAM10014[c]	Artificial reactions	0	1000	
MAR10064	Small metabolite pool for biomass reaction	(0.0043) dhor_S[c] + (0.0014) 23dpg[c] + (0.0022) 3pg[c] + (0.0025) pser_L[c] + (0.0002) accoa[c] + (0.0033) adp[c] + (0.0046) akg[c] + (0.0404) ala_L[c] + (0.0002) amp[c] + (0.0015) arg_L[c] + (0.0012) asn_L[c] + (0.0865) asp_L[c] + (0.0271) atp[c] + (0.0034) cit[c] +	Artificial reactions	0	1000	

		(0.0234) coa[c] + (0.0052) ctp[c] + (0.0005) cys_L[c] + (0.0001) damp[c] + (0.0002) dcmp[c] + (0.0094) dhap[c] + (0.0088) fdp[c] + (0.0006) f6p[c] + (0.0028) fum[c] + (0.0008) g3p[c] + (0.0002) gdp[c] + (0.0039) g6p[c] + (0.3694) glu_L[c] + (0.0998) gln_L[c] + (0.0215) gly[c] + (0.0001) gmp[c] + (0.0179) gthrd[c] + (0.0001) gthox[c] + (0.0039) gtp[c] + (0.0024) his_L[c] + (0.0002) icit[c] + (0.0102) ile_L[c] + (0.0102) leu_L[c] + (0.0029) lys_L[c] + (0.0081) mal_L[c] + (0.0037) met_L[c] + (0.0029) nad[c] + (0.0004) nadh[c] + (0.0002) nadp[c] + (0.0004) nadph[c] + (0.0049) phe_L[c] + (0.0102) phpyr[c] + (0.0071) pro_L[c] + (0.034) pyr[c] + (0.0002) r5p[c] + (0.0282) ser_L[c] + (0.0388) thr_L[c] + (0.001) trp_L[c] + (0.0054) tyr_L[c] + (0.0008) udp[c] + (0.0088) udpg[c] + (0.0006) udpglcur[c] + (0.052) uacgam[c] + (0.0102) utp[c] + (0.0088) val_L[c] --> MAM10015[c]				
MAR10065	Cofactors and vitamins pool for biomass reaction	(0.0345) retinol_cis_11[c] + (0.0172) CE2957[c] + (0.0172) CE2957[r] + (0.0172) CE2207[c] + (0.0172) CE2207[m] + (0.0172) CE2204[c] + (0.0172) CE2204[m] + (0.0345) CE1925[c] + (0.0172) CE1761[c] + (0.0172) CE1761[r] + (0.0172) CE5591[c] + (0.0172) CE5591[r] + (0.0172) CE5593[c] + (0.0172) CE5593[r] + (0.0345) CE5654;oretn[r] + (0.0172) CE5652[c] + (0.0172) CE5652[r] + (0.0172) CE5757[c] + (0.0172) CE5757[r] + (0.0172) CE2954[c] + (0.0172) CE2954[r] + (0.0345) M01032[c] + (0.0345) CE2964[c] + (0.0345) retinol_9_cis[c] + (0.0345) btn[c] + (0.0345) adocbl[m] + (0.0345) fadh2[c] + (0.0345) CE5854[c] + (0.0345) pheme[m] + (0.0345) crn[c] + (0.0345) lipoate[c] + (0.0345) CE5747[c] + (0.0345) pydx5p[c] + (0.0345) ribflv[c] + (0.0345) thbpt[c] + (0.0345) thf[c] + (0.0345) thmpp[c] + (0.0345) q10h2[m] --> MAM10012[c]	Artificial reactions	0	1000	
MAR10066		aprgstrn[c] + nad[c] --> h[c] + nadh[c] + prgstrn[c]	Steroid metabolism	0	1000	1645
MAR10067		mi13456p[c] + h2o[c] --> mi1456p[c] + pi[c]	Inositol phosphate metabolism	0	1000	253430
MAR10068		mi1456p[c] + h2o[c] --> mi145p[c] + pi[c]	Inositol phosphate metabolism	0	1000	
MAR10069		CE1589[c] + gly[c] --> coa[c] + M01989[c]	Bile acid biosynthesis	0	1000	
MAR10070		dttOX[c] + MAM10017[c] --> dtt[c] + MAM10016[c]	Miscellaneous	0	1000	
MAR10071		nadp[c] + MAM10019[c] --> C03681[c] + h[c] + nadph[c]	Steroid metabolism	0	1000	8644
MAR10072		nadp[c] + MAM10020[c] --> 17ahprgstrn[c] + h[c] + nadph[c]	Steroid metabolism	0	1000	8644
MAR10073		atp[c] + h2o[c] + MAM10021[c] --> adp[c] + h[c] + pi[c] + MAM10021[e]	Transport reactions	0	1000	8647 or 8714
MAR10074		MAM10021[e] <==>	Exchange/demand reaction	-1000	1000	
MAR10075		atp[c] + h2o[c] + MAM10022[c] --> adp[c] + h[c] + pi[c] + MAM10022[e]	Transport reactions	0	1000	8647 or 8714
MAR10076		MAM10022[e] <==>	Exchange/demand reaction	-1000	1000	
MAR10077		MAM10023[e] <==>	Exchange/demand reaction	-1000	1000	
MAR10078		MAM10024[e] <==>	Exchange/demand reaction	-1000	1000	
MAR10079		atp[c] + h2o[c] + MAM10024[c] --> adp[c] + h[c] + pi[c] + MAM10024[e]	Transport reactions	0	1000	8647 or 8714
MAR10080		atp[c] + coa[c] + MAM10021[c] --> amp[c] + ppi[c] + MAM10035[c]	Bile acid biosynthesis	0	1000	
MAR10081		MAM10025[e] <==>	Exchange/demand reaction	-1000	1000	
MAR10082		atp[c] + h2o[c] + MAM10025[c] --> adp[c] + h[c] + pi[c] + MAM10025[e]	Transport reactions	0	1000	8647 or 8714
MAR10083		thyochol[e] <==>	Exchange/demand reaction	-1000	1000	
MAR10084		MAM10027[e] <==>	Exchange/demand reaction	-1000	1000	
MAR10085		MAM10028[e] <==>	Exchange/demand reaction	-1000	1000	

MAR10086	MAM10029[e] <==>	Exchange/demand reaction	-1000	1000	
MAR10087	hyochol[e] <==>	Exchange/demand reaction	-1000	1000	
MAR10088	MAM10031[e] <==>	Exchange/demand reaction	-1000	1000	
MAR10089	MAM10032[e] <==>	Exchange/demand reaction	-1000	1000	
MAR10090	MAM10033[e] <==>	Exchange/demand reaction	-1000	1000	
MAR10091	MAM10034[e] <==>	Exchange/demand reaction	-1000	1000	
MAR10092	h2o[c] + MAM10035[c] --> coa[c] + h[c] + MAM10021[c]	Bile acid biosynthesis	0	1000	
MAR10093	taur[c] + MAM10035[c] --> coa[c] + h[c] + MAM10024[c]	Bile acid biosynthesis	0	1000	
MAR10094	atp[c] + coa[c] + MAM10022[c] --> amp[c] + ppi[c] + MAM10036[c]	Bile acid biosynthesis	0	1000	
MAR10095	h2o[c] + MAM10036[c] --> coa[c] + h[c] + MAM10022[c]	Bile acid biosynthesis	0	1000	
MAR10096	taur[c] + MAM10036[c] --> coa[c] + h[c] + MAM10032[c]	Bile acid biosynthesis	0	1000	
MAR10097	atp[c] + coa[c] + MAM10025[c] --> amp[c] + ppi[c] + MAM10037[c]	Bile acid biosynthesis	0	1000	
MAR10098	h2o[c] + MAM10037[c] --> coa[c] + h[c] + MAM10025[c]	Bile acid biosynthesis	0	1000	
MAR10099	taur[c] + MAM10037[c] --> coa[c] + h[c] + MAM10031[c]	Bile acid biosynthesis	0	1000	
MAR10100	atp[c] + h2o[c] + MAM10034[c] --> adp[c] + h[c] + pi[c] + MAM10034[e]	Transport reactions	0	1000	8647 or 8714
MAR10101	atp[c] + h2o[c] + thyochol[c] --> adp[c] + h[c] + pi[c] + thyochol[e]	Transport reactions	0	1000	8647 or 8714
MAR10102	atp[c] + h2o[c] + MAM10027[c] --> adp[c] + h[c] + pi[c] + MAM10027[e]	Transport reactions	0	1000	8647 or 8714
MAR10103	atp[c] + h2o[c] + hyochol[c] --> adp[c] + h[c] + pi[c] + hyochol[e]	Transport reactions	0	1000	8647 or 8714
MAR10104	atp[c] + h2o[c] + MAM10033[c] --> adp[c] + h[c] + pi[c] + MAM10033[e]	Transport reactions	0	1000	8647 or 8714
MAR10105	atp[c] + coa[c] + hyochol[c] --> amp[c] + ppi[c] + MAM10038[c]	Bile acid biosynthesis	0	1000	
MAR10106	h2o[c] + MAM10038[c] --> coa[c] + h[c] + hyochol[c]	Bile acid biosynthesis	0	1000	
MAR10107	taur[c] + MAM10038[c] --> coa[c] + h[c] + thyochol[c]	Bile acid biosynthesis	0	1000	
MAR10108	gly[c] + MAM10038[c] --> coa[c] + h[c] + MAM10027[c]	Bile acid biosynthesis	0	1000	
MAR10109	atp[c] + h2o[c] + MAM10029[c] --> adp[c] + h[c] + pi[c] + MAM10029[e]	Transport reactions	0	1000	8647 or 8714
MAR10110	atp[c] + h2o[c] + MAM10023[c] --> adp[c] + h[c] + pi[c] + MAM10023[e]	Transport reactions	0	1000	8647 or 8714
MAR10111	atp[c] + coa[c] + MAM10023[c] --> amp[c] + ppi[c] + MAM10039[c]	Bile acid biosynthesis	0	1000	
MAR10112	h2o[c] + MAM10039[c] --> coa[c] + h[c] + MAM10023[c]	Bile acid biosynthesis	0	1000	
MAR10113	taur[c] + MAM10039[c] --> coa[c] + h[c] + MAM10034[c]	Bile acid biosynthesis	0	1000	
MAR10114	gly[c] + MAM10039[c] --> coa[c] + h[c] + MAM10029[c]	Bile acid biosynthesis	0	1000	
MAR10115	atp[c] + h2o[c] + MAM10032[c] --> adp[c] + h[c] + pi[c] + MAM10032[e]	Transport reactions	0	1000	8647 or 8714
MAR10116	h[c] + MAM10018[c] --> alltn[c] + co2[c]	Purine metabolism	0	1000	646625
MAR10117	atp[c] + h2o[c] + MAM10031[c] --> adp[c] + h[c] + pi[c] + MAM10031[e]	Transport reactions	0	1000	8647 or 8714
MAR10118	(2) na1[e] + MAM10025[e] --> (2) na1[c] + MAM10025[c]	Transport reactions	0	1000	6554

MAR10119		(2) na1[e] + hyochol[e] --> (2) na1[c] + hyochol[c]	Transport reactions	0	1000	6554
MAR10120		(2) na1[e] + MAM10023[e] --> (2) na1[c] + MAM10023[c]	Transport reactions	0	1000	6554
MAR10121		atp[c] + coa[c] + M02155[c] --> amp[c] + h[c] + ppi[c] + MAM10040[c]	Bile acid biosynthesis	0	1000	
MAR10122		h2o[c] + MAM10040[c] --> coa[c] + M02155[c]	Bile acid biosynthesis	0	1000	
MAR10123		taur[c] + MAM10040[c] --> coa[c] + h[c] + MAM10041[c]	Bile acid biosynthesis	0	1000	
MAR10124		gly[c] + MAM10040[c] --> coa[c] + h[c] + MAM10028[c]	Bile acid biosynthesis	0	1000	
MAR10125		atp[c] + h2o[c] + MAM10041[c] --> adp[c] + h[c] + pi[c] + MAM10041[e]	Transport reactions	0	1000	8647 or 8714
MAR10126		atp[c] + h2o[c] + MAM10028[c] --> adp[c] + h[c] + pi[c] + MAM10028[e]	Transport reactions	0	1000	8647 or 8714
MAR10127		(2) na1[e] + MAM10041[e] --> (2) na1[c] + MAM10041[c]	Transport reactions	0	1000	6554
MAR10128		atp[c] + coa[c] + HC02194[c] --> amp[c] + ppi[c] + MAM10042[c]	Bile acid biosynthesis	0	1000	
MAR10129		h2o[c] + MAM10042[c] --> coa[c] + h[c] + HC02194[c]	Bile acid biosynthesis	0	1000	
MAR10130		taur[c] + MAM10042[c] --> coa[c] + HC02195[c]	Bile acid biosynthesis	0	1000	
MAR10131		gly[c] + MAM10042[c] --> coa[c] + HC02196[c]	Bile acid biosynthesis	0	1000	
MAR10132		MAM10041[e] <==>	Exchange/demand reaction	-1000	1000	
MAR12140	HMR Biomass Renal Cancer Reaction	(0.0031466) dag_hs[c] + (0.0012642) lpchol_hs[c] + (0.11871) ala_L[c] + (0.0009274) arg_L[c] + (0.012056) asn_L[c] + (0.69277) asp_L[c] + (0.0082708) chsterol[c] + (0.0065713) xolest2_hs[r] + (0.00057356) clpn_hs[c] + (0.0012056) cys_L[c] + (0.007581) dna[n] + (0.16786) glu_L[c] + (0.19104) gln_L[c] + (0.13911) gly[c] + (0.028749) his_L[c] + (0.0037096) ile_L[c] + (0.011129) leu_L[c] + (0.009274) lys_L[c] + (0.0018548) met_L[c] + (0.017171) pchol_hs[c] + (0.020051) pe_hs[c] + (0.0037096) phe_L[c] + (0.016414) pa_hs[c] + (0.0030972) pail_hs[c] + (0.017992) pro_L[c] + (0.005891) ps_hs[c] + (0.030815) M02847[c] + (0.037096) ser_L[c] + (0.0024143) sphmyln_hs[c] + (0.027482) M02958[c] + (0.020403) thr_L[c] + (0.0009274) trp_L[c] + (0.0055644) tyr_L[c] + (0.012056) val_L[c] + (0.26551) M03161[c] + (0.001) M01602[c] + (0.0038601) MAM10006[c] --> Temp001[c]	Artificial reactions	0	0	
MAR13082	Generic human cell biomass reaction	(45) atp[c] + (0.0267) dna[n] + (45) h2o[c] + (0.1124) M02847[c] + (0.4062) M03161[c] + (0.0012) MAM10012[c] + (5.3375) MAM10013[c] + (0.2212) MAM10014[c] + (0.4835) MAM10015[c] --> (45) adp[c] + (45) h[c] + (45) pi[c] + Temp001[c]	Artificial reactions	0	1000	
MAR13083		h[c] + hyptaur[c] + nadh[c] + o2[c] --> h2o[c] + nad[c] + taur[c]	Metabolism of other amino acids	0	1000	2326

Supplementary Table 8: The replicated diagnosis-related metabolites.

Common Name	VMHID	cross match With Recon3D	cross match Withi DNCT	Increased Number	Decreased Number	Inconsistent	Increased Sample	Decreased Sample
Trigonelline	-	0	0	0	4	0	-	Plasma [1–4]; CSF [4]
Benzeneacetic acid	pac	1	1	2	0	0	Urine [5, 6]	-
Hydroxyanthranilic acid	-	0	0	2	0	0	Urine [5, 6]	-
Tyramine	tym	1	1	2	0	0	Plasma [7, 8]	
p-Cresol	pcresol	1	0	2	0	0	Plasma [9]; Serum [10]	-
4-hydroxyphenylacetic acid	4hphac	1	0	5	1	1	Plasma [9, 11]; Serum [12]; Urine [5, 6]	Plasma [13]
3,4-dihydroxyphenylacetic acid	34dhpha	1	1	0	2	0	-	Plasma [14]; CSF [15]
Norepinephrine	nrpphr	1	1	0	2	0	-	Plasma [8, 14]
Dopamine	dopa	1	1	2	2	1	Plasma [16]; CSF [17]	Plasma [14]; Serum [12]
Vanillylmandelic acid	3mox4hoxm	1	1	2	0	0	Plasma [2]; Serum [12]	-
Homovanillic Acid	homoval	1	0	3	2	1	Plasma [2, 3, 18]	CSF [15, 19]
Malonylcarnitine	HC10859	1	1	2	0	0	Urine [5, 6]	-
stearoylcarnitine	stcrn	1	1	2	0	0	Plasma [2]; Serum [20]	-
Linolenyl palmitate	-	0	0	0	2	0	-	Plasma [9, 11]
2-methyl-dodecanedioic acid	-	0	0	0	2	0	-	Plasma [9, 11]
methyl 8-[3,5-epidioxy-2-(3-hydroperoxy-1-pentenyl)-cyclopentyl]-octanoate	-	0	0	2	0	0	Plasma [9, 11]	-
FFA 18:0	-	0	0	0	2	0	-	Plasma [1, 16]
FFA 20:0	-	0	0	0	2	0	-	Plasma [1, 16]
Arachidonic acid	arachd	1	1	3	2	1	Plasma [21]; CSF [22, 23]	Plasma [24]; Serum [20]
Dihomo-gamma-linolenic acid	dlnlcg	1	0	3	0	0	Plasma [9, 11]; CSF [23]	-
Hexadecanoic acid	hdca	1	1	2	3	1	Plasma [2, 25]	Plasma [24, 26]; Serum [20]
palmitoleate (16:1n7)	hdcea	1	1	1	2	1	Plasma [2]	Plasma [24, 26]
Margaric acid	hpdca	1	1	1	2	1	Plasma [2]	Plasma [20, 26]
Stearic acid	ocdca	1	0	2	3	1	Plasma [2]; whole blood [27]	Plasma [20, 24, 26]
oleate (18:1n9)	ocdcea	1	1	1	4	1	Plasma [2]	Plasma [20, 24, 26]; Faecal [28]
FA C14:0	ttdca	1	0	2	0	0	Plasma [2, 29]	-
Decanoic acid	dca	1	0	3	0	0	Plasma [2]; CSF [22, 23] ;Plasma [2, 25]	-
Dodecanoic acid	ddca	1	1	2	0	0	Plasma [2, 25]	-
FA(8:0)	octa	1	1	2	0	0	Plasma [2, 29]; Sebum [30]	-
Sebacic acid	sebacid	1	0	1	2	1	CSF [31]	Serum [32]; Faecal [28]
Mycolipanolic acid (C27)	-	0	0	0	2	0	-	Plasma [9, 11]
deoxycarnitine	4tmeabutn	1	0	2	1	1	Plasma [1, 33]	Plasma [2, 29]

Pentanoic acid	M03134	1	0	1	3	1	Plasma [34]	Plasma [35]; Faecal [36]
Butanoic acid	but	1	1	2	3	1	Plasma [34]; Urine [37]	Plasma [34]; Whole blood [27]; Faecal [36]
21:3(5Z,14Z,17Z)	-	0	0	2	0	0	Plasma [9, 11]	-
10-heptadecenoate (17:1n7)	M00003	1	1	2	1	1	Plasma [2, 29]	Serum [20]
Docosapentaenoic acid	clpnd	1	0	1	2	1	Plasma [2]	Plasma [24]; Serum [20]
FA(26:0)	hexc	1	0	0	3	0	-	Plasma [9, 11]; Sebum [30]
-mannosylglycerate	-	0	0	2	0	0	CSF [22, 23]	-
3-Hydroxybutyric acid	CE5126	1	0	4	2	1	Serum [38]; CSF [17]; Urine [37]	Plasma [24]; CSF [17]
2-tridecene-4,7-diynal	-	0	0	2	0	0	Plasma [9, 11]	-
15-tetracosenal	-	0	0	0	2	0	-	Plasma [9, 11]
N-linoleoyl taurine	-	0	0	2	0	0	Plasma [9, 11]	-
C18:2	lnlc	1	1	2	4	1	Plasma [2]; Serum [39]	Plasma [24, 26]; Serum [20]; Faecal [28]
2R-HpOTrE	-	0	0	0	2	0	-	Plasma [9, 11]
DG(15:0/18:2(9Z,12Z)/0:0)	-	0	0	0	2	0	-	Plasma [9, 11]
DG(14:0/18:1(11Z)/0:0)	-	0	0	0	2	0	-	Plasma [9, 11]
DG(15:0/18:1(11Z)/0:0)	-	0	0	0	2	0	-	Plasma [9, 11]
PA(P-16:0/18:4(6Z,9Z,12Z,15Z))	-	0	0	0	2	0	-	Plasma [9, 11]
PC aa C38:1	-	0	0	0	2	0	-	Plasma [40]; Serum [39]
Glycerophosphorylcholine	g3pc	1	0	0	2	0	-	Plasma [2]; Serum [20]
PC(14:0/22:0)	-	0	0	0	2	0	-	Plasma [9, 11]
PC 34:2	-	0	0	0	2	0	-	Plasma [1, 41]
PC 34:4	-	0	0	0	2	0	-	Plasma [1, 16]
PE(24:1(15Z)/P-18:1(11Z))	-	0	0	0	2	0	-	Plasma [9, 11]
PG(19:0/22:2(13Z,16Z))	-	0	0	0	2	0	-	Plasma [9, 11]
PG(21:0/22:1(11Z))	-	0	0	0	2	0	-	Plasma [9, 11]
PI(17:0/22:2(13Z,16Z))	-	0	0	0	2	0	-	Plasma [9, 11]
PS(12:0/15:0)	-	0	0	2	0	0	Plasma [9, 11]	
PS(20:0/22:4(7Z,10Z,13Z,16Z))	-	0	0	0	2	0	-	Plasma [9, 11]
7,8-Didehydroastaxanthin	-	0	0	0	2	0	-	Plasma [9, 11]
Valerenic acid	-	0	0	2	0	0	CSF [22, 23]	-
CerP(d18:1/20:0)		0	0	0	2	0	-	Plasma [9, 11]
Aleb(d18:1/18:0)	-	0	0	0	2	0	-	Plasma [9, 11]
GlcCer(d18:1/16:0)	-	0	0	0	2	0	-	Plasma [9, 11]
N-(Hexadecanoyl)-deoxysphing-4-enine-1-sulfonate	-	0	0	0	2	0	-	Plasma [9, 11]
SM(d18:0/17:0)	-	0	0	0	2	0	-	Plasma [9, 11]

SM(d16:1/25:0)	-	0	0	0	2	0	-	Plasma [9, 11]
SM 26:0	-	0	0	0	3	0	-	Plasma [16, 40]; CSF [17]
SM(d18:0/22:0)	-	0	0	1	2	1	Plasma [7]	Plasma [9, 11]
Sphingosine-1-phosphocholine	spc_hs	1	0	0	2	0	-	Plasma [9, 11]
G-DCA	M01989	1	0	2	1	1	Plasma [1]; CSF [17]	Plasma [2]
Chenodeoxycholic acid glycine conjugate	dgchol	1	0	2	1	1	Plasma [1, 9]	CSF [17]
GCA	gchola	1	0	2	0	0	Plasma [1, 7]	
DCA	dchac	1	0	2	1	1	Plasma [1, 3]	human appendix, ileum [42]
TCA	tchola	1	0	2	1	1	Plasma [1, 9]	CSF [17]
Dihydrocortisol		0	0	2	0	0	Urine [5, 6]	-
Cortisol	crtsl	1	0	4	0	0	Plasma [1]; Serum [20]; Urine [5, 6]	-
Hydroxyprogesterone	17ahprgstrn	1	0	2	0	0	Urine [5, 6]	-
21-deoxycortisol	M00603	1	0	2	0	0	Urine [5, 6]	-
Inosine	ins	1	1	0	2	0	-	Serum [12]; CSF [31]
Uridine	uri	1	1	1	3	1	Plasma [2]	Plasma [1, 18]; CSF [31]
pyroglutamine		0	0	2	0	0	Plasma [18]; Serum [20]	-
Alanine	ala_L	1	1	5	2	1	Plasma [13, 26];	Plasma [2, 45]
Glycine	gly	1	1	6	0	0	Serum [43]; Urine [5]; Saliva [44]	
Trimethylglycine	glyb	1	0	0	3	0	Plasma [14, 16];	Serum [20]; CSF [31, 33]
homoserine	hom_L	1	1	2	0	0	Serum [20]; Urine [5, 6]; Faecal [44]	
L-Pipecolic acid	Lpipecol	1	1	0	4	0	-	Plasma [24, 47]; Serum [12]; CSF [31]
Citrulline	citr_L	1	1	0	3	0	-	Serum [12, 48, 49]
Glutamine	gln_L	1	1	8	1	1	Plasma [9, 16]; Serum [20, 38, 43]; Putamen [38]; CSF [17, 23]; Urine [37]	CSF [26]
L-lysine	lys_L	1	1	4	4	1	Plasma [40]; Serum [38]; CSF [13]; Urine [5]	Plasma [2, 40, 45, 47]
Ornithine	orn	1	1	10	1	1	Plasma [16, 18, 40]; Serum [20, 39, 46, 49, 50]; CSF [13]; Urine [37]	Serum [12]
Thr	thr_L	1	1	5	1	1	Plasma [26]; Serum [20, 43]; CSF [17]	Plasma [2]
Asn	asn_L	1	1	2	2	1	Serum [20]; CSF [17]	Plasma [2, 29]
Aspartate	asp_L	1	1	3	1	1	Plasma [14]; Urine [5]; Serum [46]	Serum [20]
cysteine	cys_L	1	1	2	0	0	Plasma [13]; Serum [20]	-
cystathionine	cyst_L	1	1	2	0	0	Plasma [16, 29]	-
Glutamate	glu_L	1	1	2	6	1	Serum [43];Brain tissue [51]	Plasma [14]; Serum [20, 38, 52]; Faecal [28, 53]
3-methylhistidine	3mhis	1	1	0	2	0	-	Plasma [2]; Serum [49]
His	his_L	1	1	5	0	0	Serum [43]; CSF [13, 17]; Urine [6]; Saliva [44]	-

Isoleucine	ile_L	1	1	6	1	1	Serum [43]; CSF [13]; Faecal [28]; Urine [5, 37]	Faecal [54]
leucine	leu_L	1	1	4	4	1	Plasma [29]; Faecal [28];Urine [5, 37]	Plasma [29, 45]; Serum [20, 52]
Met	met_L	1	1	3	2	1	Plasma [26, 29]; CSF [17]	Plasma [2, 29]
Furoylglycine	-	0	0	2	1	1	Urine [5, 6]	Plasma [2]
Phenylacetylglycine	M02723	1	0	2	0	0	Urine [5, 6]	-
N-acetylglycine	acgly	1	1	4	0	0	Plasma [2, 18]; Serum [20]; CSF [55]	-
hexanoylglycine	hexgly	1	0	3	0	0	Plasma [2];Urine [5, 6]	-
Alpha-N-Phenylacetyl-L-glutamine	pheacgln	1	0	5	0	0	Serum [10, 20]; CSF [33]; Urine [5, 6]	-
Tiglylglycine	tiggly	1	0	2	0	0	Urine [5, 6]	-
Acetylphenylalanine		0	0	2	1	1	Urine [5, 6]	Plasma [2]
Phenylalanine	phe_L	1	1	8	3	1	Plasma [29, 35]; Serum [38, 50]; Urine [5, 37]; Faecal [28]; Saliva [44]	Serum [20, 52]; Faecal [54]
5-oxoproline	5oxpro	1	1	4	2	1	Plasma [26]; CSF [13, 23]; Urine [5]	Serum [20]; Faecal [28]
Proline	pro_L	1	1	6	0	0	Plasma [1, 13]; Serum [48, 49];Urine [5, 37]	-
serine	ser_L	1	1	2	2	1	Plasma [26, 29]	Serum [20, 49]
3-OMD	CE2176	1	0	8	0	0	Plasma [1–3, 18, 24, 29]; Serum [20]; CSF [56]	
Tyrosine	tyr_L	1	1	10	3	1	Plasma [1, 8, 16]; Serum [20, 38, 39, 46]; Urine [5, 37]; Saliva [44]; CSF [39]	Plasma [45]; Faecal [53, 54]
Valine	val_L	1	1	2	1	1	Serum [43]; Saliva [44]	Faecal [54]
Aminobutyric acid	4abut	1	1	4	0	0	Plasma [14]; Urine [5, 6]; Saliva [44]	-
HWESASXX	-	0	0	0	2	0	-	Plasma [18]; Serum [20]
leucylleucine	leuleu	1	1	0	2	0	-	Plasma [18]; Serum [20]
N-Acetylputrescine	aprut	1	1	2	0	0	Plasma [47, 57]	-
Acetate	ac	1	1	4	3	1	Serum [38, 58]; Urine [37]; Saliva [44]	Plasma [59]; Faecal [34, 36]
Propanoic acid	ppa	1	1	2	2	1	Plasma [34]; Saliva [44]	Faecal [34, 36]
Glutaric acid	glutar	1	0	1	2	1	Urine [5]	Plasma [59]; Whole blood [27]
Succinate	succ	1	1	3	2	1	Plasma [40]; Urine [5, 37]	Plasma [59]; Faecal [28]
Citrate	cit	1	1	4	2	1	Plasma [3]; Serum [43, 58]; CSF [17]	Plasma [59]; Serum [20]
Malic acid	mal_L	1	1	2	2	1	Plasma [26]	Plasma [59]; Serum [20]
10-Hydroxydecanoic acid	-	0	0	2	0	0	CSF [22, 23]	-
Pyruvate	pyr	1	1	2	2	1	Plasma [59];Urine [5]	Plasma [13]; Faecal [53]
2-oxoisocaproate acid	4mop	1	1	2	0	0	Plasma [26]; CSF [13]	-
Urea	urea	1	0	2	3	1	Plasma [3, 25]	Urine [60]; CSF [61]; Brian tissue [62]
Phosphoethanolamine	ethamp	1	1	2	0	0	Serum [48, 49]	-
3-indoxyl sulfate	inds	1	0	2	0	0	Plasma [16]; Serum [20]	-

homovanillate sulfate	-	0	0	2	0	0	Plasma [2, 18]	-
catechol sulfate	-	0	0	0	2	0	-	Plasma [2, 18]
3-methoxytyramine sulfate	-	0	0	2	0	0	Plasma [2, 18]	
p-Cresol sulfate	pcs	1	0	6	0	0	Plasma [1, 9]; Serum [10, 20]; CSF [22, 23]	-
ethanolamine	etha	1	1	1	2	1	Faecal [28]	Plasma [33, 59]
Putrescine	ptrc	1	1	2	0	0	Plasma [39]; CSF [17, 39]	-
spermidine	spmd	1	1	3	0	0	Serum [39, 57]; Urine [6]	-
trimethylamine	M03053	1	0	0	2	0	-	Plasma [59]; Faecal [53]
TMAO	M03054	1	0	5	1	1	Plasma [63]; Serum [58]; Urine [5, 6]; Saliva [44]	Faecal [53]
5-acetylamino-6-amino-3-methyluracil	-	0	0	1	5	1	Plasma [18]	Plasma [2, 64]; Serum [12, 65, 66]
C0	crn	1	1	1	2	1	Plasma [40]	Plasma [35]; CSF [17]
choline	chol	1	1	1	2	1	Urine [6]	Serum [20]; Faecal [53]
Quinic acid	quint	0	0	2	1	1	CSF [22, 23]	Plasma [2]
L-Lactic acid	lac_L	1	1	3	1	1	Serum [20, 43]; CSF [17]	Plasma [45]
Pantothenic acid	pnto_R	1	0	0	5	0	-	Plasma [1, 18]; Serum [12]; Faecal [28]; Brain tissue [62]
Myo-inositol	inost	1	1	2	2	1	Plasma [59]; CSF [17]	Plasma [1]; CSF [26]
Fucose	fuc_L	1	0	2	1	1	Plasma [13]; Saliva [44]	CSF [26]
Glucose	glc_D	1	1	3	2	1	Plasma [45]; Serum [43]; CSF [61]	Plasma [18]; CSF [31]
Glyceric acid	glyc_R	1	1	1	2	1	Plasma [2]	Serum [20]; CSF [61]
Threonic acid	thrnt	1	0	2	1	1	Serum [20]; CSF [61]	Plasma [59]
Threitol	-	0	0	1	2	1	CSF [13]	Plasma [24]; Whole blood [27]
mannitol	C01507	1	0	2	0	0	Plasma [18]; CSF [13]	-
Glycerol	glyc	1	1	1	2	1	Plasma [45]	Plasma [59]; Serum [20]
glucitol	sbt_D	1	1	2	0	0	Plasma [59]; CSF [13]	-
3-ketosphingosine	-	0	0	2	0	0	Plasma [24]; CSF [33]	-
Acetone	acetone	1	1	4	0	0	Serum [38, 43, 50]; CSF [17]	-
Kynurenine	Lkynr	1	1	3	3	1	Plasma [16]; Urine [5, 6]	Plasma [1, 67]; Serum [52]
3-Hydroxykynurenine	hLkynr	1	0	2	0	0	Serum [12]; CSF [31]	-
Imidazoleacetic acid	im4ac	1	1	2	0	0	Urine [5, 6]	-
Urocanic acid	urcan	1	1	3	0	0	Plasma [2]; Urine [5, 6]	-
5-acetylamino-6-formylamino-3-methyluracil	-	0	0	0	2	0	-	Plasma [2]; Serum [66]
Hypoxanthine	hxan	1	0	2	1	1	Urine [5, 6]	Plasma [69]
theophylline	-	0	0	1	6	1	Plasma [18]	Plasma [2, 4]; Serum [12, 65, 66, 68]; CSF [4]
caffeine	-	0	0	0	5	0	-	Plasma [2, 4, 18]; Serum [65, 66]; CSF [4]
1,7-dimethylxanthine	-	0	0	0	5	0	-	Plasma [2, 4, 18]; Serum [65, 66]; CSF [4]

Theobromine	-	0	0	0	2	0	-	Serum [65, 66]
7-Methylxanthine	-	0	0	0	2	0	-	Serum [65, 66]
1,3-dimethylurate	-	0	0	0	2	0	-	Plasma [2]; Serum [65]
3-Methylxanthine	-	0	0	0	2	0	-	Serum [12, 66]
1,3,7-trimethylurate	-	0	0	0	2	0	-	Plasma [2, 18]
1-methylxanthine	-	0	0	0	5	0	-	Plasma [2, 4, 12]; Serum [65, 66]; CSF [4]
1,7-dimethylurate	-	0	0	0	4	0	-	Plasma [2, 18]; Serum [65, 66]
Urate	urate	1	0	1	3	1	Plasma [13]	Plasma [69, 70]; Serum [71]
Xanthine	xan	1	1	2	1	1	Plasma [2, 69]	CSF [17]
Tryptophan	trp_L	1	1	1	4	1	Urine [5]	Plasma [67]; CSF [26, 52]; Faecal [54]
Indole-3-acetic acid	ind3ac	1	1	1	3	1	Urine [6]	Plasma [2, 3, 18]
5-HTP	5htrp	1	1	4	0	0	Plasma [16, 56]; Urine [5, 6]	-
Serotonin	srtn	1	1	1	3	1	Plasma [40]	Plasma [8]; Serum [12, 20]
Pyridoxic acid	4pyrdx	1	0	2	2	1	Urine [5, 6]	Plasma [2, 18]
Nicotinic acid	nac	1	1	1	2	1	Urine [5]	CSF [27]; Faecal [28]
Xanthurenic acid	C02470	1	0	2	0	0	Urine [5, 6]	-
Bilirrubin	bilirub	1	0	2	1	1	Plasma [64]; Serum [20]	Plasma [18]
Biliverdin	biliverd	1	0	2	1	1	Plasma [18]; Serum [20]	Plasma [1]
Tephcalostan	-	0	0	2	0	0	Plasma [9, 11]	-
3-(4-hydroxyphenyl)lactate	34hpl	1	1	2	0	0	Plasma [18]; Serum [20]	-
L-3-Phenyllactic acid	phlac	1	1	4	0	0	Plasma [9, 11, 18]; Serum [20]	-
Vanillactic acid	vanillac	1	0	2	1	1	Plasma [2, 35]	Plasma [7]

Reference:

[1] Y. Shao et al. “Comprehensive metabolic profiling of Parkinson’s disease by liquid chromatography-mass spectrometry”. eng. In: Molecular Neurodegeneration 16.1 (Jan. 2021), p. 4.

[2] A. Okuzumi et al. “Metabolomics-based identification of metabolic alterations in PARK2”. eng. In: Annals of Clinical and Translational Neurology 6.3 (Mar. 2019), pp. 525–536.

[3] S. Saiki et al. “Decreased long-chain acylcarnitines from insufficient β-oxidation as potential early diagnostic markers for Parkinson’s disease”. eng. In: Scientific Reports 7.1 (Aug. 2017), p. 7328.

[4] G. F. Crotty et al. “Association of caffeine and related analytes with resistance to Parkinson disease among LRRK2 mutation carriers: A metabolomic study”. eng. In: Neurology 95.24 (Dec. 2020), e3428–e3437.

[5] H. Luan et al. “Comprehensive urinary metabolomic profiling and identification of potential noninvasive marker for idiopathic Parkinson’s disease”. eng. In: Scientific Reports 5 (Sept. 2015), p. 13888.

[6] H. Luan et al. “LC-MS-based urinary metabolite signatures in idiopathic Parkinson’s disease”. en. In: Journal of Proteome Research 14.1 (Jan. 2015), pp. 467–478.

[7] L. Hu et al. “Integrated Metabolomics and Proteomics Analysis Reveals Plasma Lipid Metabolic Disturbance in Patients With Parkinson’s Disease”. eng. In: Frontiers in Molecular Neuroscience 13 (2020), p. 80.

[8] G. D’Andrea et al. “Different Circulating Trace Amine Profiles in De Novo and Treated Parkinson’s Disease Patients”. eng. In: Scientific Reports 9.1 (Apr. 2019), p. 6151.

[9] M.-X. Dong et al. “Metabolomics profiling reveals altered lipid metabolism and identifies a panel of lipid metabolites as biomarkers for Parkinson’s disease related anxiety disorder”. eng. In: Neuroscience Letters 745 (Feb. 2021), p. 135626.

[10] M. S. Cirstea et al. “Microbiota Composition and Metabolism Are Associated With Gut Function in Parkinson’s Disease”. eng. In: Movement Disorders: Official Journal of the Movement Disorder Society 35.7 (July 2020), pp. 1208–1217.

[11] M.-X. Dong, Y.-D. Wei, and L. Hu. “Lipid metabolic dysregulation is involved in Parkinson’s disease dementia”. eng. In: Metabolic Brain Disease 36.3 (Mar. 2021), pp. 463–470.

[12] W. Han et al. “Profiling novel metabolic biomarkers for Parkinson’s disease using in-depth metabolomic analysis”. eng. In: Movement Disorders: Official Journal of the Movement Disorder Society 32.12 (Dec. 2017), pp. 1720–1728.

[13] A. Wuolikainen et al. “Multi-platform mass spectrometry analysis of the CSF and plasma metabolomes of rigorously matched amyotrophic lateral sclerosis, Parkinson’s disease and control subjects”. English. In: Molecular Biosystems 12.4 (2016). Place: Cambridge Publisher: Royal Soc Chemistry WOS:000372612600023, pp. 1287–1298.
[14] A. Kim et al. “Upgraded Methodology for the Development of Early Diagnosis of Parkinson’s Disease Based on Searching Blood Markers in Patients and Experimental Models”. eng. In: Molecular Neurobiology 56.5 (May 2019), pp. 3437–3450.
[15] T. Kremer et al. “Longitudinal Analysis of Multiple Neurotransmitter Metabolites in Cerebrospinal Fluid in Early Parkinson’s Disease”. eng. In: Movement Disorders: Official Journal of the Movement Disorder Society 36.8 (Aug. 2021), pp. 1972–1978.
[16] K.-H. Chang et al. “Alterations of Sphingolipid and Phospholipid Pathways and Ornithine Level in the Plasma as Biomarkers of Parkinson’s Disease”. en. In: Cells 11.3 (Jan. 2022), p. 395.
[17] A. Yilmaz et al. “Metabolic Profiling of CSF from People Suffering from Sporadic and LRRK2 Parkinson’s Disease: A Pilot Study”. eng. In: Cells 9.11 (Oct. 2020), E2394.
[18] T. Hatano et al. “Identification of novel biomarkers for Parkinson’s disease by metabolomic technologies”. eng. In: Journal of Neurology, Neurosurgery, and Psychiatry 87.3 (Mar. 2016), pp. 295–301.
[19] P. LeWitt et al. “CSF xanthine, homovanillic acid, and their ratio as biomarkers of Parkinson’s disease”. In: Brain research 1408 (Aug. 2011), pp. 88–97.
[20] E. C. Schulte et al. “Alterations in Lipid and Inositol Metabolisms in Two Dopaminergic Disorders”. English. In: Plos One 11.1 (Jan. 2016). Place: San Francisco Publisher: Public Library Science WOS:000369527800054, e0147129.
[21] J. Zhang et al. “Targeted fatty acid metabolomics to discover Parkinson’s disease associated metabolic alteration”. eng. In: Journal of mass spectrometry: JMS 56.10 (Oct. 2021), e4781.
[22] M. Lucio et al. “Integrative Metabolomic and Metallomic Analysis in a Case-Control Cohort With Parkinson’s Disease”. eng. In: Frontiers in Aging Neuroscience 11 (2019), p. 331.
[23] D. Willkommen et al. “Metabolomic investigations in cerebrospinal fluid of Parkinson’s disease”. eng. In: PloS One 13.12 (2018), e0208752.
[24] C. Gonzalez-Riano et al. “Prognostic biomarkers of Parkinson’s disease in the Spanish EPIC cohort: a multiplatform metabolomics approach”. eng. In: NPJ Parkinson’s disease 7.1 (Aug. 2021), p. 73.
[25] E. Glaab et al. “Integrative analysis of blood metabolomics and PET brain neuroimaging data for Parkinson’s disease”. eng. In: Neurobiology of Disease 124 (Apr. 2019), pp. 555–562.
[26] M. Trupp et al. “Metabolite and peptide levels in plasma and CSF differentiating healthy controls from patients with newly diagnosed Parkinson’s disease”. eng. In: Journal of Parkinson’s Disease 4.3 (2014), pp. 549–560.
[27] J. Troisi et al. “A metabolomic signature of treated and drug-naive patients with Parkinson disease: A pilot study”. English. In: Movement Disorders 33 (Oct. 2018). Place: Hoboken Publisher: Wiley WOS:000446176703014, S818–S818.
[28] S. Vascellari et al. “Gut Microbiota and Metabolome Alterations Associated with Parkinson’s Disease”. eng. In: mSystems 5.5 (Sept. 2020), e00561–20.
[29] J. Hertel et al. “Integrated Analyses of Microbiome and Longitudinal Metabolome Data Reveal Microbial-Host Interactions on Sulfur Metabolism in Parkinson’s Disease”. eng. In: Cell Reports 29.7 (Nov. 2019), 1767–1777.e8.
[30] E. Sinclair et al. “Metabolomics of sebum reveals lipid dysregulation in Parkinson’s disease”. eng. In: Nature Com- munications 12.1 (Mar. 2021), p. 1592.
[31] P. A. Lewitt et al. “3-hydroxykynurenine and other Parkinson’s disease biomarkers discovered by metabolomic analysis”. eng. In: Movement Disorders: Official Journal of the Movement Disorder Society 28.12 (Oct. 2013), pp. 1653–1660.
[32] J. R. Roede et al. “Serum metabolomics of slow vs. rapid motor progression Parkinson’s disease: a pilot study”. eng. In: PloS One 8.10 (2013), e77629.
[33] D. Stoessel et al. “Promising Metabolite Profiles in the Plasma and CSF of Early Clinical Parkinson’s Disease”. eng. In: Frontiers in Aging Neuroscience 10 (2018), p. 51.
[34] S.-J. Chen et al. “Association of Fecal and Plasma Levels of Short-Chain Fatty Acids With Gut Microbiota and Clinical Severity in Patients With Parkinson Disease”. eng. In: Neurology 98.8 (Feb. 2022), e848–e858.
[35] H. Zhao et al. “Potential biomarkers of Parkinson’s disease revealed by plasma metabolic profiling”. eng. In: Journal of Chromatography. B, Analytical Technologies in the Biomedical and Life Sciences 1081-1082 (Apr. 2018), pp. 101–108.
[36] E. De Pablo-Fernandez et al. “The faecal metabolome and mycobiome in Parkinson’s disease”. eng. In: Parkinsonism & Related Disorders 95 (Feb. 2022), pp. 65–69.
[37] S. Kumari et al. “Identification of potential urine biomarkers in idiopathic parkinson’s disease using NMR”. eng. In: Clinica Chimica Acta; International Journal of Clinical Chemistry 510 (Nov. 2020), pp. 442–449.
[38] B. Toczylowska et al. “Changes in the metabolic profiles of the serum and putamen in Parkinson’s disease patients - In vitro and in vivo NMR spectroscopy studies”. eng. In: Brain Research 1748 (Dec. 2020), p. 147118.
[39] S. Plewa et al. “The Metabolomic Approach Reveals the Alteration in Human Serum and Cerebrospinal Fluid Composition in Parkinson’s Disease Patients”. eng. In: Pharmaceuticals (Basel, Switzerland) 14.9 (Sept. 2021), p. 935.
[40] M. Pathan et al. “Plasma Metabolite Markers of Parkinson’s Disease and Atypical Parkinsonism”. eng. In: Metabolites 11.12 (Dec. 2021), p. 860.
[41] R. B. Chan et al. “Elevated GM3 plasma concentration in idiopathic Parkinson’s disease: A lipidomic analysis”. eng. In: PloS One 12.2 (2017), e0172348.
[42] P. Li et al. “Gut Microbiota Dysbiosis Is Associated with Elevated Bile Acids in Parkinson’s Disease”. eng. In: Metabolites 11.1 (Jan. 2021), p. 29.
[43] G. N. Babu et al. “Serum metabolomics study in a group of Parkinson’s disease patients from northern India”. English. In: Clinica Chimica Acta 480 (May 2018). Place: Amsterdam Publisher: Elsevier Science Bv WOS:000428826100031, pp. 214–219.
[44] S. Kumari et al. “Quantitative metabolomics of saliva using proton NMR spectroscopy in patients with Parkinson’s disease and healthy controls”. eng. In: Neurological Sciences: Official Journal of the Italian Neurological Society and of the Italian Society of Clinical Neurophysiology 41.5 (May 2020), pp. 1201–1210.
[45] C. Dong et al. “Plasma Metabolite Signature Classifies Male LRRK2 Parkinson’s Disease Patients”. eng. In: Metabolites 12.2 (Feb. 2022), p. 149.
[46] S. Klatt et al. “A six-metabolite panel as potential blood-based biomarkers for Parkinson’s disease”. eng. In: NPJ Parkinson’s disease 7.1 (Oct. 2021), p. 94.

[47] E. E. Balashova et al. “Plasma Metabolome Signature in Patients with Early-stage Parkinson Disease”. English. In: Current Metabolomics 6.1 (2018). Place: Busum Publisher: Bentham Science Publ WOS:000422962800012, pp. 75–82.
[48] R. Calvani et al. “A novel multi-marker discovery approach identifies new serum biomarkers for Parkinson’s disease in older people: an EXosomes in PArkiNson Disease (EXPAND) ancillary study”. eng. In: GeroScience 42.5 (Oct. 2020), pp. 1323–1334.
[49] A. Picca et al. “Circulating amino acid signature in older people with Parkinson’s disease: A metabolic complement to the EXosomes in PArkiNson Disease (EXPAND) study”. eng. In: Experimental Gerontology 128 (Dec. 2019), p. 110766.
[50] G. Meoni et al. “Metabolite and lipoprotein profiles reveal sex-related oxidative stress imbalance in de novo drug-naive Parkinson’s disease patients”. eng. In: NPJ Parkinson’s disease 8.1 (Feb. 2022), p. 14.
[51] H. R. Griffith et al. “Reduced brain glutamate in patients with Parkinson’s disease”. eng. In: NMR in biomedicine 21.4 (May 2008), pp. 381–387.
[52] M. S. Fiandaca et al. “Potential Metabolomic Linkage in Blood between Parkinson’s Disease and Traumatic Brain Injury”. eng. In: Metabolites 8.3 (Sept. 2018), E50.
[53] A. H. Tan et al. “Gut Microbial Ecosystem in Parkinson Disease: New Clinicobiological Insights from Multi-Omics”. eng. In: Annals of Neurology 89.3 (Mar. 2021), pp. 546–559.
[54] Z. Yan et al. “Alterations of gut microbiota and metabolome with Parkinson’s disease”. eng. In: Microbial Pathogenesis 160 (Nov. 2021), p. 105187.
[55] P. A. LeWitt et al. “Metabolomic biomarkers as strong correlates of Parkinson disease progression”. eng. In: Neurology 88.9 (Feb. 2017), pp. 862–869.
[56] J. F. Havelund et al. “Changes in kynurenine pathway metabolism in Parkinson patients with L-DOPA-induced dyskinesia”. eng. In: Journal of Neurochemistry 142.5 (Sept. 2017), pp. 756–766.
[57] S. Saiki et al. “A metabolic profile of polyamines in parkinson disease: A promising biomarker”. eng. In: Annals of Neurology 86.2 (Aug. 2019), pp. 251–263.
[58] S. Kumari et al. “Metabolomic analysis of serum using proton NMR in 6-OHDA experimental PD model and patients with PD”. eng. In: Neurochemistry International 134 (Mar. 2020), p. 104670.
[59] S. S. Ahmed et al. “Metabolic profiling of Parkinson’s disease: evidence of biomarker from gene expression analysis and rapid neural network detection”. eng. In: Journal of Biomedical Science 16 (July 2009), p. 63.
[60] A. W. Michell et al. “Metabolomic analysis of urine and serum in Parkinson’s disease”. English. In: Metabolomics 4.3 (Sept. 2008). Place: New York Publisher: Springer WOS:000259007900001, pp. 191–201.
[61] J.-P. Trezzi et al. “Distinct metabolomic signature in cerebrospinal fluid in early parkinson’s disease”. eng. In: Movement Disorders: Official Journal of the Movement Disorder Society 32.10 (Oct. 2017), pp. 1401–1408.
[62] M. Scholefield et al. “Substantively Lowered Levels of Pantothenic Acid (Vitamin B5) in Several Regions of the Human Brain in Parkinson’s Disease Dementia”. eng. In: Metabolites 11.9 (Aug. 2021), p. 569.
[63] S.-J. Chen et al. “The Gut Metabolite Trimethylamine N-oxide Is Associated With Parkinson’s Disease Severity and Progression”. eng. In: Movement Disorders: Official Journal of the Movement Disorder Society 35.11 (Nov. 2020), pp. 2115–2116.
[64] S. M. Albillos et al. “Plasma acyl-carnitines, bilirubin, tyramine and tetrahydro-21-deoxycortisol in Parkinson’s disease and essential tremor. A case control biomarker study”. eng. In: Parkinsonism & Related Disorders 91 (Oct.2021), pp. 167–172.
[65] H. Takeshige-Amano et al. “Shared Metabolic Profile of Caffeine in Parkinsonian Disorders”. eng. In: Movement Disorders: Official Journal of the Movement Disorder Society 35.8 (Aug. 2020), pp. 1438–1447.
[66] M. Fujimaki et al. “Serum caffeine and metabolites are reliable biomarkers of early Parkinson disease”. eng. In: Neurology 90.5 (Jan. 2018), e404–e411.
[67] K.-H. Chang et al. “Alternations of Metabolic Profile and Kynurenine Metabolism in the Plasma of Parkinson’s Disease”. eng. In: Molecular Neurobiology 55.8 (Aug. 2018), pp. 6319–6328.
[68] K. K. Johansen et al. “Metabolomic profiling in LRRK2-related Parkinson’s disease”. eng. In: PloS One 4.10 (Oct. 2009), e7551.
[69] T. Ohmichi et al. “Biomarker repurposing: Therapeutic drug monitoring of serum theophylline offers a potential diagnostic biomarker of Parkinson’s disease”. eng. In: PloS One 13.7 (2018), e0201260.
[70] M. Bogdanov et al. “Metabolomic profiling to develop blood biomarkers for Parkinson’s disease”. eng. In: Brain: A Journal of Neurology 131.Pt 2 (Feb. 2008), pp. 389–396.
[71] A. Bougea et al. “Serum Uric Acid in LRRK2 Related Parkinson’s Disease: Longitudinal Data from the PPMI Study”. eng. In: Journal of Parkinson’s Disease 11.2 (2021), pp. 633–640.

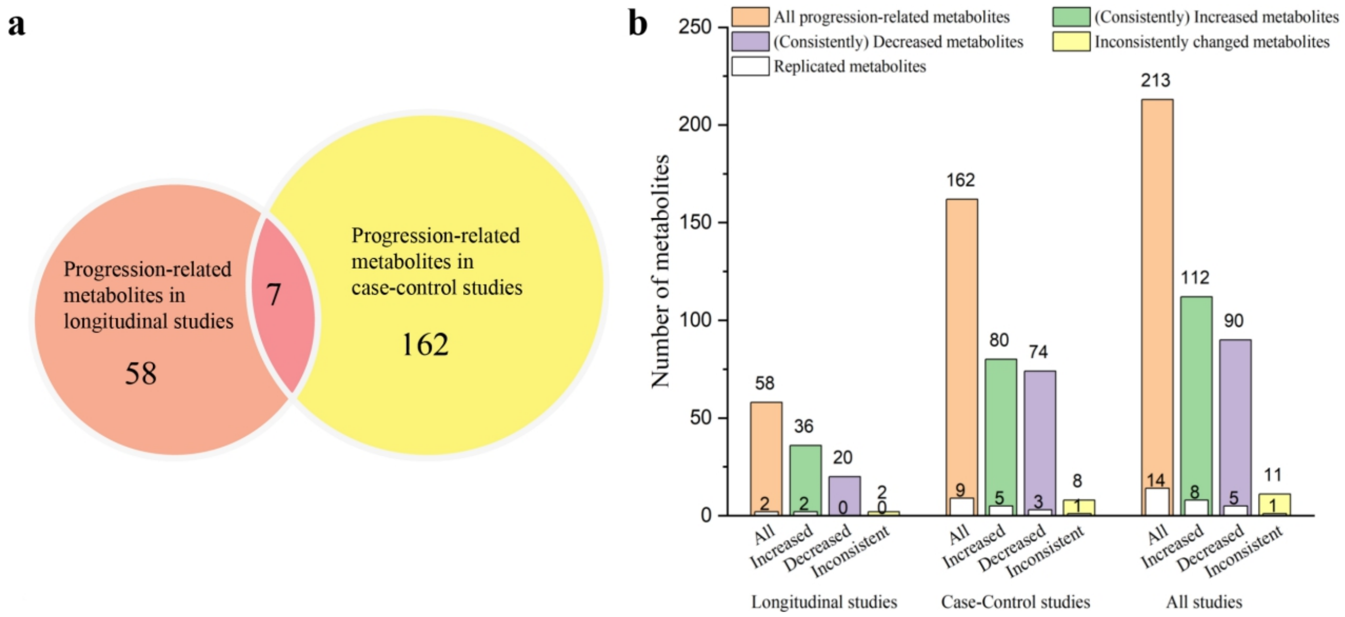

Supplementary Figure 1. PD progression-related metabolites.

a. Overlapped results of progression-related metabolites in longitudinal studies and case-control studies. b. Statistic results of PD progression-related metabolites in longitudinal studies, case-control studies, and all potential progression-related studies.

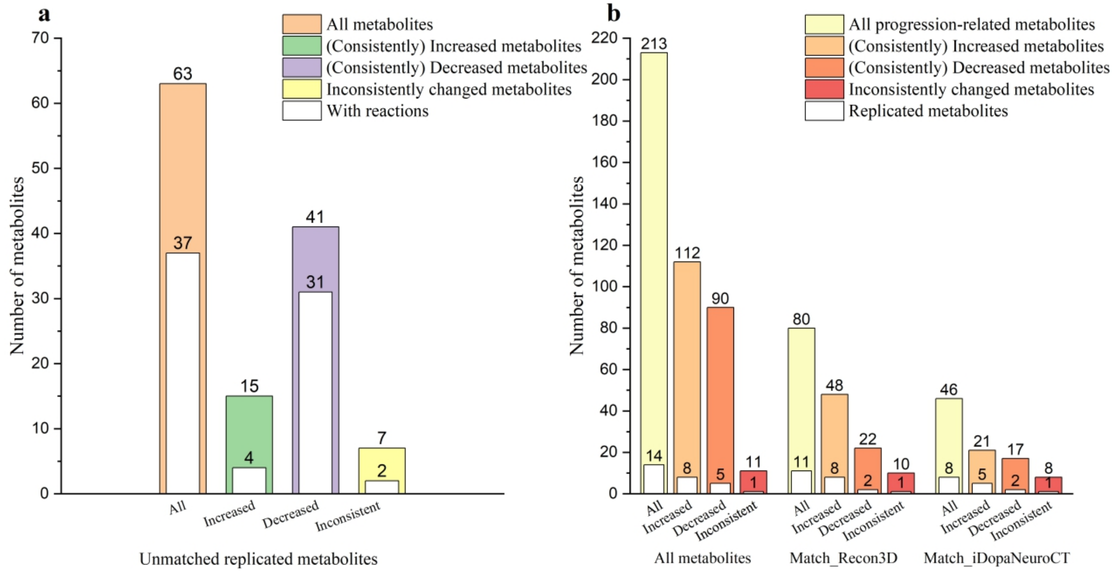

Supplementary Figure 2. Statistics of PD metabolites.

a Unmatched replicated metabolites of diagnosis-related metabolites. **b** Data statistics of progression-related metabolites of PD.

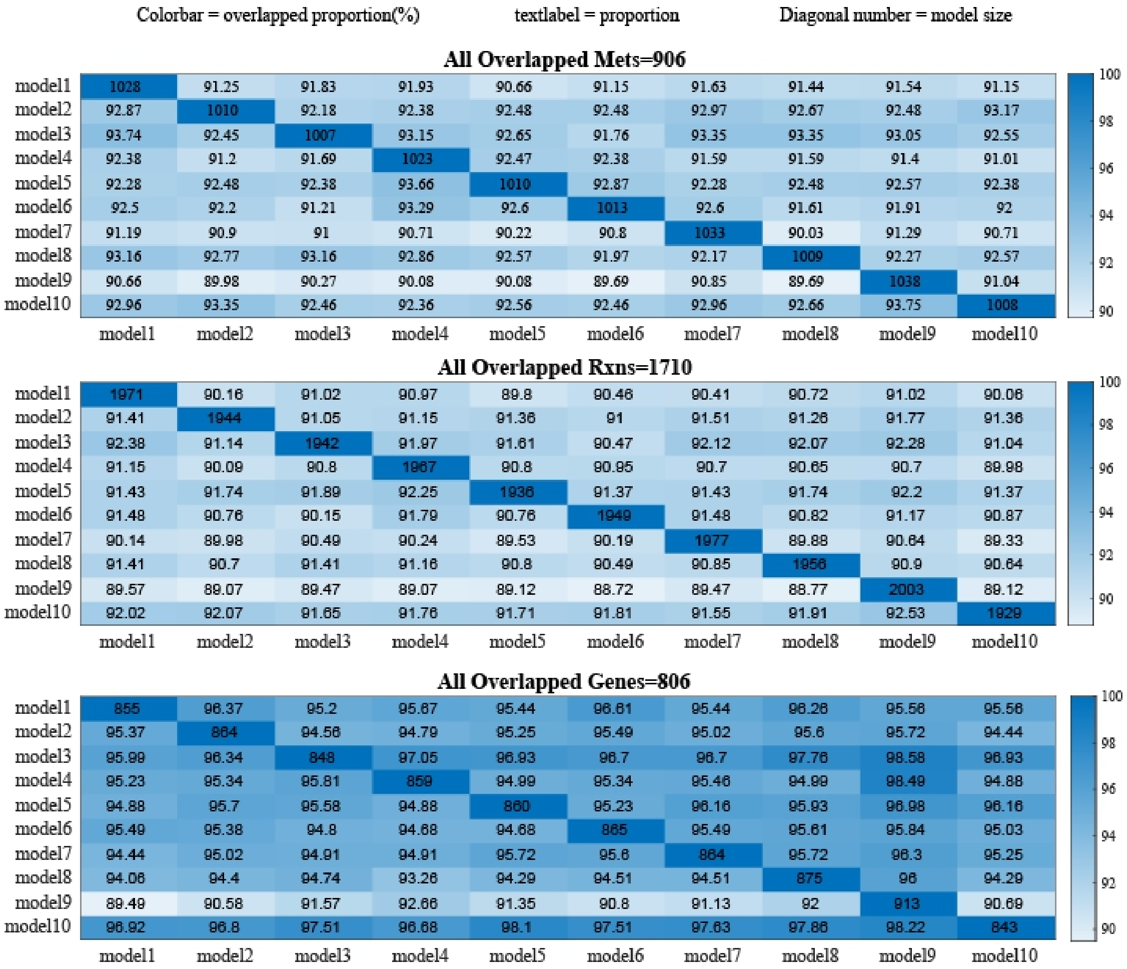

Supplementary Figure 3. The heat maps for overlapped metabolites, reactions and genes between the randomly generated 10 models. The diagonal label represents the number of metabolites, reactions and genes in each model, respectively. Other labels and the colourbar represent the shared proportion between each pair of models. All shared metabolites, reactions and genes over ten models were listed on the top of the map, respectively.

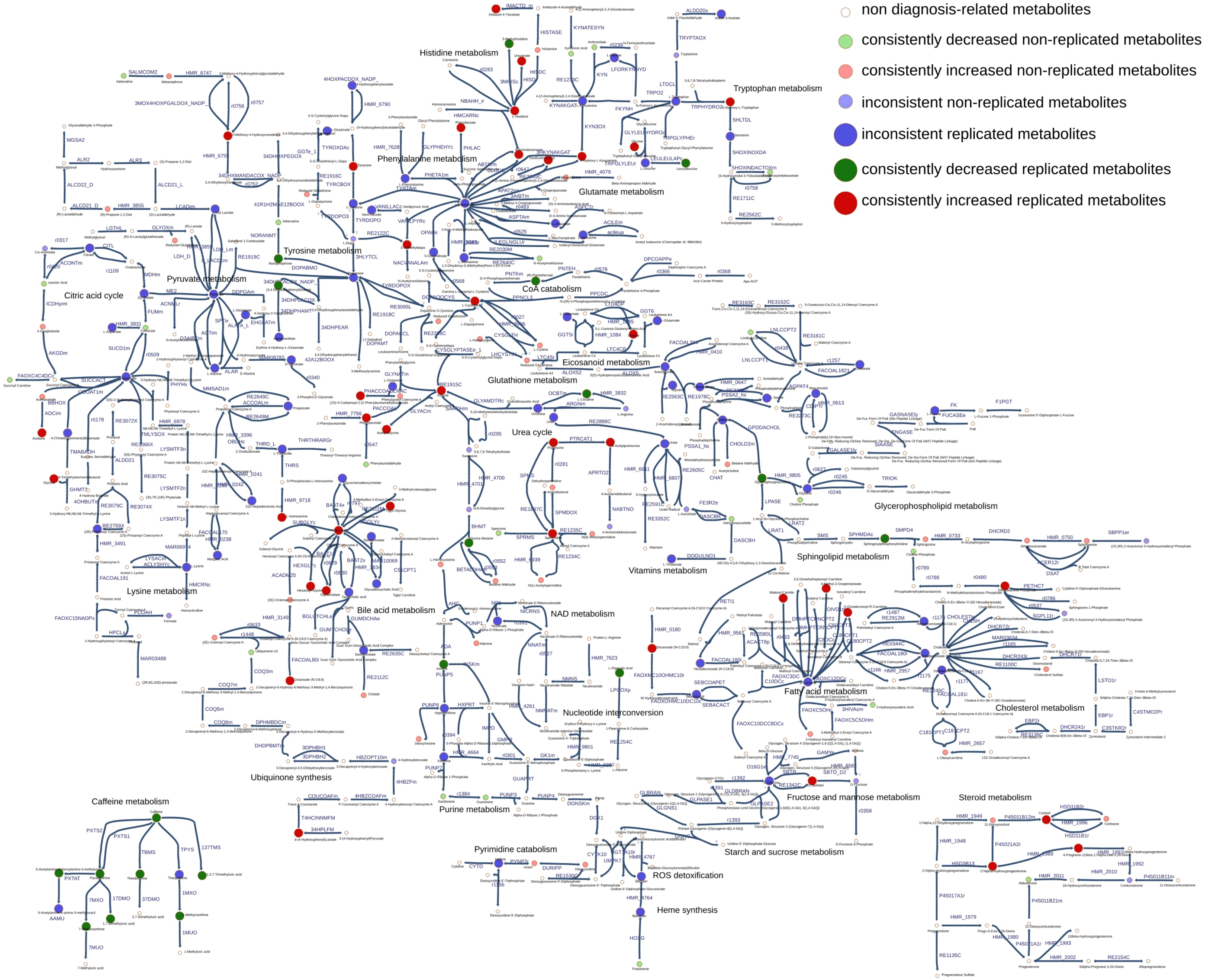

Supplementary Figure 4. The raw PD map.
